# Identification of host transcriptional networks showing concentration-dependent regulation by HPV16 E6 and E7 proteins in basal cervical squamous epithelial cells

**DOI:** 10.1038/srep29832

**Published:** 2016-07-26

**Authors:** Stephen P. Smith, Cinzia G. Scarpini, Ian J. Groves, Richard I. Odle, Nicholas Coleman

**Affiliations:** 1 Cambridge University Hospitals, Addenbrooke’s Hospital, Cambridge, CB2 2QQ, UK; 2 Department of Pathology, University of Cambridge, CB2 1QP, UK

## Abstract

Development of cervical squamous cell carcinoma requires increased expression of the major high-risk human-papillomavirus (HPV) oncogenes E6 and E7 in basal cervical epithelial cells. We used a systems biology approach to identify host transcriptional networks in such cells and study the concentration-dependent changes produced by HPV16-E6 and -E7 oncoproteins. We investigated sample sets derived from the W12 model of cervical neoplastic progression, for which high quality phenotype/genotype data were available. We defined a gene co-expression matrix containing a small number of highly-connected hub nodes that controlled large numbers of downstream genes (regulons), indicating the scale-free nature of host gene co-expression in W12. We identified a small number of ‘master regulators’ for which downstream effector genes were significantly associated with protein levels of HPV16 E6 (n = 7) or HPV16 E7 (n = 5). We validated our data by depleting E6/E7 in relevant cells and by functional analysis of selected genes *in vitro*. We conclude that the network of transcriptional interactions in HPV16-infected basal-type cervical epithelium is regulated in a concentration-dependent manner by E6/E7, via a limited number of central master-regulators. These effects are likely to be significant in cervical carcinogenesis, where there is competitive selection of cells with elevated expression of virus oncoproteins.

High-risk human papillomavirus infection (HR-HPV) is responsible for ~4.8% of the world’s cancers, including carcinoma of the cervix^1^. Most cervical malignancies are squamous cell carcinomas (SCC), which arise from precursor squamous intraepithelial lesions (SILs)^2^. A critical step in early cervical squamous carcinogenesis is deregulated expression of the major HR-HPV oncogenes E6 and E7 in the basal cell layers of infected epithelia^3^,^4^, leading to increased oncoprotein levels per cell. However, the consequences of such deregulation are difficult to study in detail using animal models or clinical samples. The latter, for example, would require micro-dissection of the basal epithelial layers of cervical SILs and, to date, such studies have not been performed. Greater insights have been gained using experimental models of cervical neoplastic progression. These include the W12 system, which is an accurate and tractable model of the early stages of cervical squamous carcinogenesis associated with HPV16, the major HR-HPV type^5^,^6^,^7^.

The W12 system was derived from ‘parental’ W12 keratinocytes (squamous epithelial cells), which were generated by explant culture of a low-grade SIL^8^. This had arisen following natural infection of the cervix with HPV16, the major type of HR-HPV. When grown in monolayer culture, W12 cells show restricted differentiation and retain the phenotype of basal squamous epithelium^6^. The cells are able to maintain HPV16 episomes stably at ~100 copies per cell, although long term passage *in vitro* eventually leads to episome clearance and selection of cells in which the HPV16 DNA is integrated into host chromatin^7^. Such events are associated with phenotypic progression of the epithelia re-formed by the W12 cells in organotypic tissue culture, from low-grade SIL to SCC^6^.

In the present study, we used multiple unique resources derived from the W12 system to identify host gene transcriptional networks in basal-type cervical keratinocytes and study the concentration-dependent changes produced by HPV16 E6 and E7 oncoproteins. First, we used fifteen representative cell clones (sample set 1) from a larger set that had been generated from the same background population, namely polyclonal early-stage W12 cells^9^,^10^. The clones were derived under non-competitive conditions, regardless of their overall selectability, and differed only by the genomic site of HPV16 integration^10^. Importantly, the different integration sites resulted in ~6-fold variation in levels of HPV16 E6 and E7 proteins per cell in monolayer culture^9^. However, across the clones the association between the levels of the two proteins was weak and statistically non-significant, allowing the effects of each virus oncoprotein to be studied individually. The E6 and E7 protein levels showed weak associations with cell growth rates, although these were again non-significant^9^.

Second, we used data from previous experiments in which we examined the effects of depleting the HPV16 oncogenes in various populations of W12 cells^11^, using siRNAs known not to cause significant off-target effects in squamous epithelial cells from the cervix and skin^12^. We identified host gene expression changes that were consistently seen in independent samples of W12 cells containing integrated HPV16 DNA (sample set 2) and were therefore suitable for direct comparison with the gene lists derived from the integrant-containing W12 clones.

Gene expression profiling of these complementary W12 sample sets has enabled us to perform massively parallel analysis of complex network interactions within the host transcriptome in HPV16-containing basal cervical squamous cells. We have identified host gene co-expression patterns and critical master regulator hubs that coordinate and regulate multiple downstream effects. Our data indicate that HPV16 E6 and E7 oncoproteins show concentration-dependent modulation of a complex network of p53-dependent and p53-independent transcriptional events in basal-type cervical keratinocytes.

## Results

### Identification of host genes showing concentration-dependent relationships with HPV16 E6 and E7 proteins in basal cervical squamous cells

We first used fifteen W12 clones containing integrated HPV16^10^, namely: 3, B, D2, F, G2, H, H2, J, J3, O2, Q, Q2, R2, S2, and Z (sample set 1). Across the clones examined, levels of HPV16 E6 and E7 proteins per cell each varied ~5-fold^9^, while none expressed full length E2 protein. Three replicate samples were examined for each clone (45 arrays in total). Differential expression of host gene transcripts across the clones was analysed using a linear model, with HPV16 E6 and E7 protein levels as predictors^9^ and cell growth rates as control covariates. After adjustment for multiple hypothesis testing, we identified genes showing a significant concentration-dependent association (adjusted p value < 0.05) with each virus protein. In total, 1,213 genes were associated with E6 levels (Supplementary Table S1), 1,527 genes with E7 levels (Supplementary Table S2) and 171 genes with both. Table 1 lists the twenty genes most significantly associated with E6 and the twenty most significantly associated with E7.

By signalling pathway impact analysis (SPIA), host genes showing concentration-dependent associations with HPV16 E6 protein were enriched significantly for ‘cell cycle’, ‘DNA replication’ and ‘evasion of apoptosis’ (Supplementary Figure S1A), while those associated with HPV16 E7 protein were enriched for ‘RNA transport’ and ‘cell cycle effects’ (Supplementary Figure S1B). Genes associated with cancer pathways annotated in the Kyoto Encyclopedia of Genes and Genomes (KEGG) are shown in Supplementary Figure S2A for HPV16 E6 and in Supplementary Figure S2B for HPV16 E7.

### Host genes are transcribed in a scale-free coordinated network in basal cervical squamous cells

**Figure 1:**
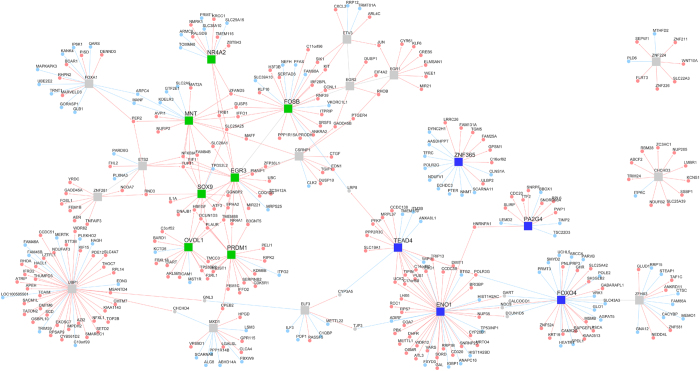
The transcriptional regulatory network of gene expression in W12. The square nodes indicate central transcriptional master regulators. Those regulators for which expression levels were directly associated with HPV16 E6 protein levels are shown in green, those associated with HPV16 E7 protein levels in red and those not directly associated with E6 or E7 levels in grey. Circles indicate the downstream effector genes, with light blue indicating an overall activating regulation and pink indicating overall repression.

The transcript level of each gene expressed in each of the fifteen W12 clones was analysed using a mutual information ARACNe algorithm, to define a gene co-expression matrix. The derived network (Fig. 1) contained a small number of central highly connected “hub” nodes controlling large numbers of downstream genes, indicating the scale-free nature of host gene co-expression in W12. Many hub nodes corresponded to TFs known to regulate cascades of downstream genes, including some TFs previously implicated in carcinogenesis.

### Central controllers of the transcriptional network are regulated by E6 and E7 in a concentration-dependent manner

**Figure 2:**
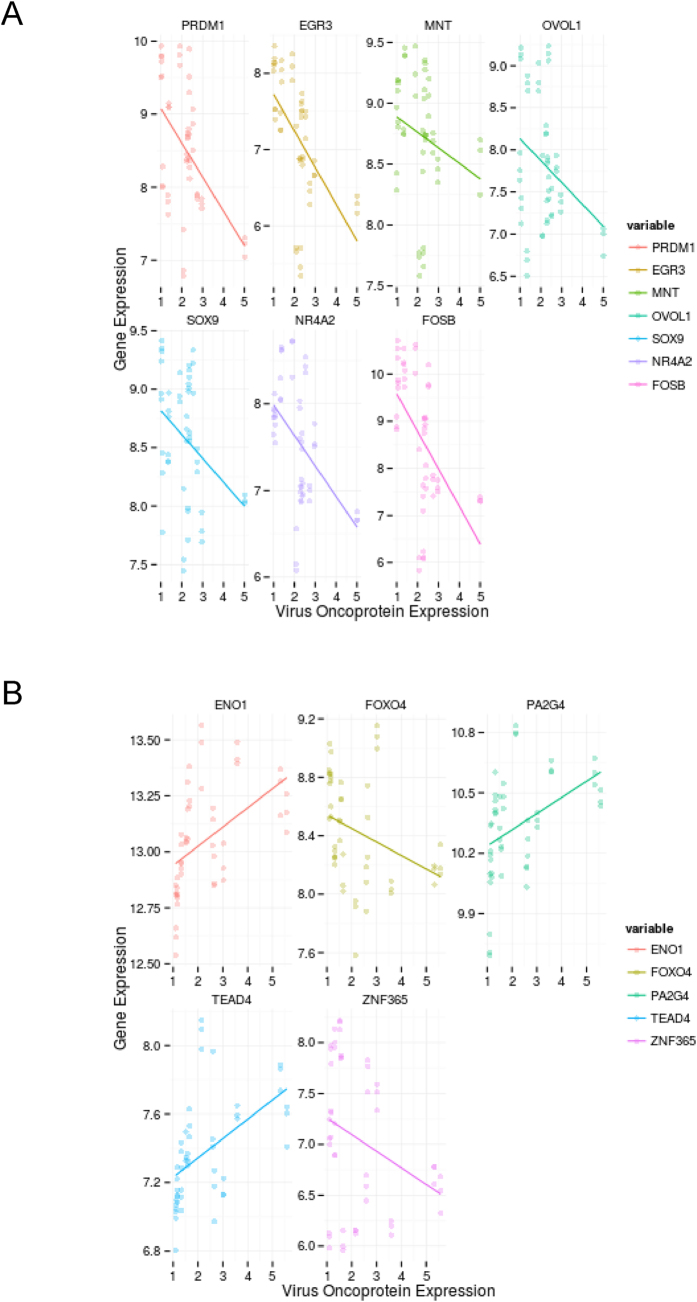
Correlations between levels of HPV16 oncoproteins and those of the master regulators. Each graph shows expression levels of a master regulator (y-axis), plotted against levels of their associated virus protein (x-axis), for HPV16 E6 (**A**) and HPV16 E7 (**B**). The p values and correlation coefficients for each analysis are given in Tables 2 and 3, respectively.

To identify putative master regulators of HPV16 oncoprotein function, we interrogated the transcriptional network for genes whose transcription levels were significantly associated with levels of either HPV16 E6 or E7 protein. Those hubs whose collection of downstream effectors (the regulon) were over-represented in the list of E6 or E7 associated genes (as identified with a Fisher’s exact test), compared with a random selection from the network, were considered “master regulators” of E6 or E7 function. We identified seven master regulators for HPV16 E6 (EGR3, FOSB, NR4A2, PRDM1, SOX9, OVOL1, and MNT) and five for HPV16 E7 (PA2G4, ENO1, TEAD4, FOXO4 and ZNF365) (Tables 2 and 3 respectively). Levels of E6 and E7 proteins correlated individually with each of the respective master regulators (Fig. 2A,B). The correlations for E6 were all negative, while E7 showed positive correlations with three master regulators (PA2G4, ENO1 and TEAD4) and negative correlations with two (FOXO4 and ZNF365). In addition, levels of E6 and E7 correlated with those of the downstream effector genes (Supplementary Figure S3A,B).

### E6-dependent regulators act via p53 targets and through p53-independent pathways

As HRHPV E6 protein is known to destabilize p53^1^, the analyses were repeated after excluding probes corresponding to known p53 target genes^13^ from the raw microarray data. Of the p53 targets (n = 2,496), 144 probesets overlapped with the genes significantly associated with HPV16 E6 protein level and 148 with E7-associated genes (22 in common). Using genelists that excluded the p53 targets (Supplementary Table S3) reduced the overall network size from 608 edges to 198 edges. The network derived from genes that were both E6- and p53-associated contained three of the E6-associated regulons (controlled by PRDM1, SOX9 and OVOL1) (Supplementary Figure S4A), while the network derived from genes that were E6-associated but not p53-associated retained two of the E6-associated regulons (controlled by EGR3 and FOSB) (Supplementary Figure S4B). The other two E6-associated regulons (controlled by MNT and NR4A2) did not appear in either of these networks.

### Depletion of HPV16 early transcripts alters expression of the E6- and E7-associated host genes, including most of the master regulators

We next analysed data from previous experiments (sample set 2) where HPV16 early genes were depleted in W12 cells using E7-141 siRNAs, which were known not to cause significant off-target effects in squamous epithelial cells from the cervix and skin^11^,^12^. The siRNAs targeted HPV16 E7 sequences, but depleted E6- and E7-containing transcripts by similar amounts (76–90%), in keeping with the polycistronic nature of HPV16 early transcripts^11^,^12^. We identified significant gene expression changes that occurred in siRNA-treated cells, compared with both non-targeting control (NTC)-treated and untreated control cells. A final list was derived of changes that were common to two independent cell samples that contained integrated HPV16 DNA, namely a representative W12 clone from our previous cell cloning work^10^ (clone G2 at p13) and cells that had emerged spontaneously (due to a selective advantage *in vitro*) during one long-term culture series of polyclonal W12 (W12 Series 4B at p83, referred to as W12Ser4Bp83). These gene expression changes were therefore common effects of HPV16 oncogene depletion in W12 cells containing integrated HPV16 DNA and were suitable for direct comparison with the gene lists derived from the integrant-containing W12 clones (sample set 1).

Using the ARACNe algorithm, we inferred a gene co-expression network from host gene expression changes in cells treated with siRNAs targeting HPV16 early transcripts versus cells treated with control siRNAs (Supplementary Figure S5). The structure of this network was again scale-free, similar to the network derived from the W12 clone dataset (sample set 1) (Fig. 1). Of 5,535 genes that were significantly differentially expressed following HPV16 early gene depletion, 937 (16.9%) also showed a concentration dependent relationship with E7 and/or E6 protein in the W12 clones (Supplementary Table S5 and Supplementary Figure S6). The transcriptional network derived from the W12 clones (Fig. 1) was re-analyzed for master regulators using these 937 genes. Regulons controlled by four of the seven E6 master regulators (FOSB, EGR3, SOX9 and NR4A2) and all five E7 master regulators (PA2G4, ENO1, TEAD4, FOXO4 and ZNF365) were again significantly enriched. No additional master regulators were identified in this analysis.

### Data validation and functional analysis of a representative novel master regulator

**Figure 3:**
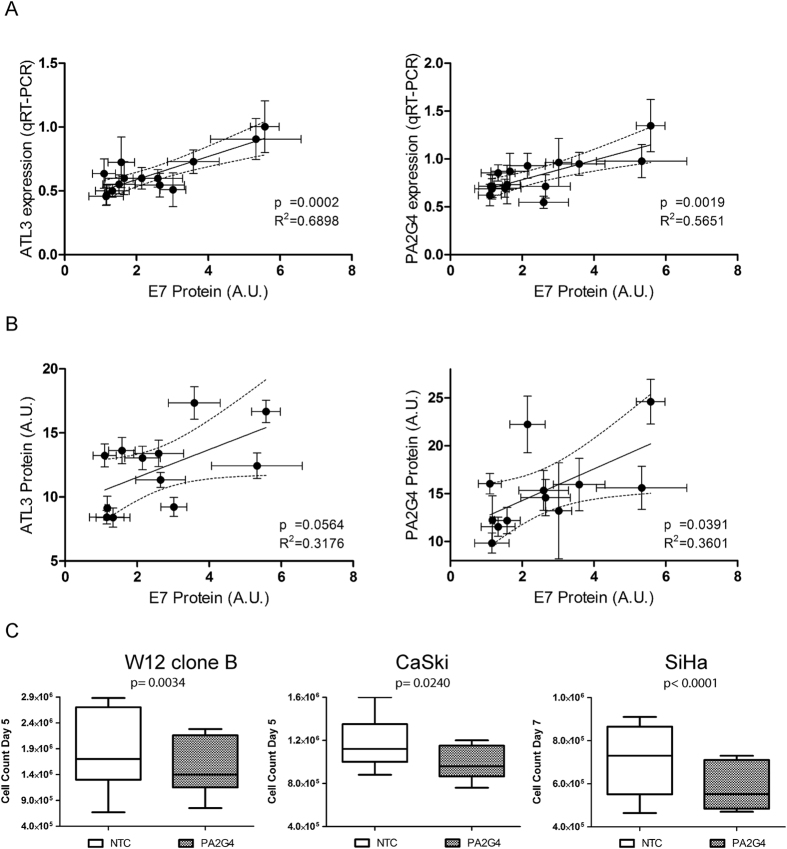
Verification of selected gene expression changes. (**A,B**) Correlations between levels of HPV16 E7 protein and those of PA2G4 and ATL3 mRNA (measured by qRT-PCR) (**A**) and protein (**B**). (**C**) Effects on cell numbers following treatment of cervical high-grade SIL (W12 clone B) or SCC (CaSki and SiHa) cells with PA2G4 siRNAs or non-targeting control siRNAs.

Data validation was performed on one master regulator (PA2G4) and one downstream effector gene (ATL3), for which microarray data showed positive correlations with HPV16 E7 protein levels. For both genes, mRNA levels determined by microarray correlated significantly with those determined by qRT-PCR (Supplementary Figure S7A), while protein levels determined by western blotting (Supplementary Figure S7B) correlated significantly with the mRNA levels determined by both microarray (data not shown) and qRT-PCR (Supplementary Figure S7C). Across the W12 clones, levels of HPV16 E7 protein correlated significantly with levels of PA2G4 and ATL3 mRNA measured by qRT-PCR (Fig. 3A), as well as with levels of PA2G4 protein measured by western blotting (Fig. 3B). There was also a near-significant correlation (p = 0.0564) between levels of HPV16 E7 protein and ATL3 protein (Fig. 3B).

We examined further the functional significance of the master regulator PA2G4. Analysis of data from our previous siRNA experiments^11^,^12^ showed that depletion of HPV16 early transcripts produced a 1.29-fold reduction in PA2G4 transcript levels (p < 0.000001). By KEGG analysis, the predicted regulon of PA2G4 in the overall transcriptional network (Fig. 1) showed enrichment (p < 0.05) for two pathways, namely ‘cell cycle’ and ‘RNA binding’. We depleted PA2G4 in cells with high-level expression, using HPV16-positive cervical keratinocytes that reformed SCCs in organotypic tissue culture (SiHa and CaSki), as well as representative W12 cells that reformed a high-grade SIL (W12 clone B). Across the cells, levels of mRNA were depleted by 80 to 90% (Supplementary Figure S8A). By western blotting only the p48 isoform of PA2G4 was seen in the cells (Supplementary Figure S8B), with 50 to 90 % reductions in protein levels following siRNA treatment (Supplementary Figure S8C). In all cells tested, PA2G4 depletion significantly reduced cell growth in monolayer culture (W12 clone B p = 0.0034; CaSki p = 0.024; SiHa p < 0.0001; unpaired t-tests) (Fig. 3C).

## Discussion

The W12 system has provided high quality phenotype and genotype data for systems biology analysis of HPV16 effects in basal-type cervical squamous cells. Our high stringency approach, which controlled for effects of cell growth rate, has identified a network of transcriptional interactions that show concentration-dependent regulation by HPV16 E6 and E7 oncoproteins. The central master regulators control groups of downstream effector genes and are important candidate targets of the major HPV16 oncoproteins. The E6/E7 effects would be greatest in the cells that are selected for during cervical carcinogenesis, as selection is associated with elevated levels of virus oncoproteins per cell, whether the virus is integrated or remains episomal^5^,^6^.

The validity of the network we identified is supported by the presence of known gene interactions, including p53 targets in the E6-associated regulons and E2F-regulated genes in the E7-associated regulons. The HR-HPV E7 protein is able to destabilise hypophosphorylated pRb and related pocket proteins in basal squamous epithelial cells, thereby releasing E2F proteins and enabling S-phase re-entry in stratified epithelia^14^,^15^. The HR-HPV E6 protein destabilises p53 via the proteasome pathway and many of the genes in the E6-associated regulons were known p53 targets^16^. We identified master regulators that are critically p53-dependent targets of HPV16 E6 (PRDM1, OVOL1 and SOX9), with E6-induced reductions in their levels leading to increased expression of multiple pro-malignant downstream target genes.

PRDM1 (BLIMP1) is a transcriptional repressor that is a master regulator of lymphoid cell differentiation and a tumour suppressor gene in lymphoma^17^,^18^. The Epstein-Barr virus oncogene latent membrane protein (LMP1) has been shown to down-regulate PRDM1 expression in primary human germinal centre B cells^19^. However, to our knowledge, this is the first report of its association with HPV16 oncoprotein effects. OVOL1 is a TF that appears to regulate mesenchymal to epithelial transition^20^ and has been implicated in a variety of carcinomas, including oral SCC^21^. SOX9 is a potential tumor suppressor in cervical carcinoma, where it trans-activates p21WAF1/CIP1 and suppresses cell growth^22^.

In addition to its effects on p53, HR-HPV E6 can destabilise other targets, such as PDZ domain-containing proteins involved in cell-cell communication, signalling, cell polarity and control of proliferation^16^,^23^. Our data indicate that p53-independent mechanisms are likely to contribute to E6-associated network alterations in HPV16-infected cells, as the E6-associated master regulators EGR3 and FOSB were retained following exclusion of p53 target genes^13^. It is interesting that HPV16 E6 showed negative correlations with all seven identified master regulators, while E7 showed positive correlations with three and negative correlations with two. Whether these differences reflect distinct modes of action of the respective virus proteins remains to be investigated.

We chose PA2G4 (EBP1) for functional validation of our findings, in view of conflicting reports in the literature concerning its significance in cancer. PA2G4 is a transcriptional and translational regulator that has recently been recognised as an E2F target gene, via non-consensus promoter response elements^24^. While in some settings the gene product appeared to cause growth inhibition^25^,^26^, in others a growth promoting effect was seen^27^,^28^. Our present data indicate that PA2G4 is up-regulated by HPV16 E7 protein in basal-type cervical squamous cells and induces pro-proliferative downstream genes. Such effects are thought to be promoted by the p48 isoform of PA2G4, which can localise to the nucleus, whereas the p42 isoform, which does not localise to the nucleus, promotes cell differentiation^27^. In the present study, only the p48 isoform was detectable in basal-like cervical squamous cells, and overall levels correlated positively with cell growth rates and HPV16 E7 protein abundance. Consistent with these observations, depletion of PA2G4 in HPV16-positive cervical squamous cells produced significant reductions in growth rates. This was seen both in cells that reformed a SIL in organotypic tissue culture (W12 clone B) and those that reformed an SCC (CaSki and SiHa).

In conclusion, our detailed analysis of multiple W12 cell populations has revealed a core gene transcriptional network in basal-type cervical squamous cells that shows concentration-dependent regulation by HPV16 E6 and E7 proteins. The master regulators identified are likely to be important cellular targets of HPV16, and potentially other HR-HPV types, during the virus life cycle and/or subsequent neoplastic transformation.

## Materials and Methods

### W12 clones and SCC cells

The W12 clones were studied at the lowest available passage (p) after cloning (generally between p3 and p8), in order to minimise any effects of genomic instability caused by deregulated HPV16 oncogenes. All W12 cells were grown in monolayer culture using irradiated 3T3J2 fibroblast feeder cells^29^, in order to restrict cell differentiation and maintain the phenotype of the basal epithelial cell layer. The feeder cells were removed before the keratinocytes were harvested. Levels of HPV16 E6 and E7 proteins per cell were calculated using quantitative Western blotting, as described in detail previously^9^. Cell population doubling times in monolayer culture were calculated previously, as described^9^. We also used the HPV16-positive cervical SCC cell lines SiHa and CaSki^30^,^31^, which did not require feeder cell support.

### Microarray expression profiling

For each replicate, probes were synthesized using the Illumina TotalPrep-96 RNA Amplification Kit and hybridized to Illumina HT-12 v4 BeadChip Arrays using standard Illumina procedures (Cambridge Genomic Services, University of Cambridge, U.K.). Three replicates were performed for each W12 clone sample, to control for variation in labeling and hybridization efficiency. The raw intensities files are available at ArrayExpress (Accession number: E-MTAB-4409).

### Quality control and normalisation

Microarray data were analysed using the R statistical programming language^32^. Initial data entry, normalisation, filtering and quality assessment were performed using the beadarray package^33^. Raw data from the BeadScan output were read with the Illumina Human v4 annotation^34^. The bead-level data were assessed for integrity at multiple levels. Raw image quality was assessed visually, by viewing image plots, and numerically, using the BASH algorithm^35^, and weights assigned to each probe.

The data were log-transformed and probes summarized to provide probe-set data for downstream analysis. Quantile normalisation was applied in order to prepare the data for between-array comparisons. Probe sets with poor or worse quality scores based on the annotation were excluded from the final data set. Normalised, summarized and filtered data were stored as an expression set object for all further downstream analysis.

### Microarray data modelling

The normalised expression data were modelled using the limma R package^36^ to investigate differential gene expression. HPV16 E6 and E7 protein levels were used as continuous covariates to characterise gene expression trends, with cell growth rates as a control co-variate. Plotting was achieved with the ggplot2 package^37^.

### Network inference

Network inference was conducted using the RTN (Reconstruction of Transcriptional Networks and Analysis of Master Regulators) R package. Transcription factors (TF) were identified from the microarray data set by selecting probes with GO annotation indicating TF activity (codes: 0003700, 0003702, 0003709, 0016563, 0016564), combined with a list of TFs identified in a previous study^38^. A mutual information network was constructed using the ARACNe algorithm^39^ on the expression matrix from all samples, and all probe sets identified as differentially expressed for any covariate (p < 0.05 with Benjamini-Hochberg (BH) correction). Significant edges were identified using p-value cutoff of 0.005 and 1000 permutations. Bootstrap filtering using 95 consensus samples from 100 bootstrap runs were selected, with subsequent filtering using data processing inequality (DPI), which removes the weakest interaction in any triangle of two TFs and a target gene, thus preserving the dominant TF-target pairs.

The network model contained 402 TFs from a potential 758 in the input probe sets. 479 targets were identified with 608 edges after DPI filtering (676 prior to filtering). The network was filtered to exclude hubs containing fewer than eight downstream nodes. The final network was stored as an iGraph object^40^ and visualised using RedeR^41^. Sub graphs of neighborhoods within 1 degree of each significant TF identified in the master regulator analysis (see below) were plotted using standard iGraph and RedeR commands.

### Master regulator analysis

The genes identified as significantly differentially expressed in the linear models were used as the input for Master Regulator Analysis (MRA)^42^ in the RTN package. The algorithm computes the hypergeometric (Fisher’s exact test) significance of the overlap between the ARACNe-inferred targets of each TF (the “regulons”) and the genes identified as differentially expressed in the E6 and E7 groups. Regulons of size 12 or greater were selected, and enrichment with an adjusted p-value of <0.01 identified. We tested for direct associations between levels of the master regulators and those of HPV16 E6 or E7 proteins, using the cor.test function in R to calculate the correlation coefficient (r) and test for significant deviation from no correlation.

### Functional enrichment analysis

Functional enrichment analysis was conducted using the signalling pathway impact analysis (SPIA) algorithm^43^. This combines standard enrichment analysis with a statistical measure of the perturbation by an input set of genes on available pathways in the Kyoto Encyclopedia of Genes and Genomes (KEGG) database. The result is a combined statistical score which identifies with specificity those pathways significantly impacted by a group of genes.

### Depletion of host gene PA2G4

The host gene PA2G4 was depleted in W12 clone B, CaSki and SiHa cells using a pool of four siRNAs (ON-TARGETplus SMARTpool L-008860, Dharmacon, GE Lifesciences, Hatfield, UK). Each siRNA was at 10 nM, producing a final concentration of 40 nM. All transfections used Lipofectamine RNAiMAX (Invitrogen, Paisley, UK), at 2 ml per well. For negative controls we used untreated cells, plus those transfected with pooled non-targeting control siRNAs (ON-TARGET plus non-targeting D-001810, Dharmacon), again at 40 nM.

### Real time quantitative PCR and Western blotting

Relative mRNA levels were measured by QuantiTect One-Step SYBR-Green qRT-PCR (Qiagen, Crawley, UK) as described^9^,^44^, using the primers and conditions listed in Supplementary Table S6. Expression ratios were calculated using the comparative threshold cycle (Ct) method^45^ and normalized using three housekeeping genes; GAPDH, YWHAZ and RPL13^46^,^47^. Western blotting and densitometry were performed as described^9^,^44^, using the primary antibodies listed in Supplementary Table S7. Levels of PA2G4 and ATL3 proteins were determined for thirteen of the clones that underwent expression profiling (omitting clones D2 and O2). Arbitrary expression values were obtained by referencing levels to those in an independent W12 clone (clone E3)^9^.

### Effects of gene depletion on cell growth

5 × 10^4^ cells were seeded per well of a 6-well plate and treated 24 hours later with PA2G4 siRNAs or NTC siRNAs. When the cells had reached sub-confluence (five days for CaSki and W12 clone B and seven days for SiHa), the numbers of PA2G4-treated cells per well were determined and referenced to the numbers of NTC-treated cells per well.

## Additional Information

**How to cite this article**: Smith, S. P. *et al.* Identification of host transcriptional networks showing concentration-dependent regulation by HPV16 E6 and E7 proteins in basal cervical squamous epithelial cells. *Sci. Rep.* **6**, 29832; doi: 10.1038/srep29832 (2016).

## Supplementary Material

Supplementary Information

## Figures and Tables

**Table 1:** Top twenty genes associated with levels of HPV16 E6 and HPV16 E7 proteins.

HPV16 E6			HPV16 E7		
Name (HUGO)	Log 2 fold change	Adjusted p	Name (HUGO)	Log 2 fold change	Adjusted p
PBDC1	−1.03	0.000000	GPX2	−0.69	0.000660
CTGF*	−0.95	0.000188	CASP14	0.60	0.000000
EGR1	−0.88	0.000062	CYBA	−0.59	0.000062
LOR	0.81	0.001949	NA	−0.58	0.003000
EGR2	−0.73	0.000411	CA9	0.57	0.000006
ALDH1A1	0.70	0.000216	FABP4	−0.56	0.044572
FOSB	−0.69	0.012565	TGM2	−0.44	0.000741
PI3	0.67	0.030491	GSTM1	−0.43	0.040663
CYR61	−0.64	0.001252	SERPINA3	0.41	0.006356
PTGER4	−0.60	0.001805	RGCC	−0.41	0.008526
CYP1B1	0.60	0.003717	SLURP1	−0.40	0.006983
DUSP5*	−0.58	0.003133	NSG1	−0.39	0.002350
ALDH1A1	0.57	0.000101	GSTM1	−0.39	0.049513
TNFSF10	−0.57	0.000050	BCAP31	−0.37	0.001684
EDN1	−0.56	0.000053	GAL	0.37	0.015374
TGM2	0.55	0.003001	ABI3BP	−0.37	0.002835
ATF3*	−0.54	0.001805	CKB	−0.37	0.000206
NCOA7	−0.52	0.010842	ECHDC3	0.34	0.001858
HBEGF*	−0.51	0.011440	ADIRF	−0.33	0.015188
IER3	−0.51	0.000006	GHR	−0.33	0.024698

Genes marked with an asterisk are identified p53 targets.

**Table 2:** Master regulators associated with HPV16 E6 protein.

Master Regulator Analysis			Correlation with E6 protein		
Master Regulator	Regulon Size	Hits	Adj. P value	Cor p-val	r
EGR3	20	15	1.3e-14	1.920077e-05	−0.5749358
FOSB	26	14	9.6e-11	8.02598e-05	−0.5379808
SOX9	15	7	1.1e-05	0.01122992	−0.3629557
PRDM1	19	7	5.8e-05	0.0002166108	−0.5095245
MNT	19	7	5.8e-05	0.09426613	−0.2442864
NR4A2	13	5	3.1e-04	0.0004620858	−0.4859879
OVOL1	16	5	1.0e-03	0.02640054	−0.320413

The left side of the Table refers to the Master Regulator Analysis. ‘Regulon Size’ is the number of ARACNe-inferred gene targets of each master regulator, while ‘Hits’ gives the numbers of those genes for which expression levels were significantly associated with levels of HPV16 E6. The p-values were determined using Fisher’s exact test. The right side of the Table refers to testing of individual correlations between levels of the master regulators and those of E6 protein, where the p-value refers to deviation from no correlation and r is the correlation coefficient.

**Table 3:** Master regulators associated with HPV16 E7 protein.

Master Regulator Analysis			Correlation with E7 protein		
Master Regulator	Regulon Size	Hits	Adj. P value	Cor p-val	r
ENO1	46	33	5.5e-26	0.0003043132	0.4991733
TEAD4	20	16	3.9e-15	0.0002321984	0.5074353
FOXO4	30	12	1.8e-06	0.01354267	−0.354097
ZNF365	17	7	1.3e-04	0.03120806	−0.3113934
PA2G4	12	4	6.6e-03	0.0017995	0.4390035

The left side of the Table refers to the Master Regulator Analysis, while the right side refers to testing of individual correlations between levels of the master regulators and those of E7 protein. Further details are as for Table 2.
