# Supplementary material for: Identification of host transcriptional networks showing concentration-dependent regulation by HPV16 E6 and E7 proteins in basal cervical squamous epithelial cells
Source: Sci Rep. 2016 Jul 26;6:29832. doi: 10.1038/srep29832 (PMC4960560; doi:10.1038/srep29832)
Supplement: Supplementary Information [file srep29832-s1.pdf]

Supplementary Material

Identification of host transcriptional networks showing concentration-dependent regulation by HPV16 E6 and E7 proteins in basal cervical squamous epithelial cells

Stephen P Smith[1], Cinzia G Scarpini[2], Ian J Groves[2], Richard I Odle[2] and Nicholas Coleman[1,2]

1. Cambridge University Hospitals, Addenbrooke's Hospital, Cambridge, CB2 2QQ, UK

2. Department of Pathology, University of Cambridge, CB2 1QP, UK

Legends to Supplementary Figures and Tables.

Supplementary Figure S1. Signalling pathway impact analysis (SPIA) evidence plots.

Each dot represents a pathway in the Kyoto Encyclopedia of Genes and Genomes (KEGG) database. The y-axis shows the degree of perturbation and the x-axis the enrichment score in the gene input list (genes associated with HPV16 E6 protein in panel A; genes associated with HPV16 E7 protein in panel B). Both axes plot $-\log_{10}$ values. For pathways above the blue line, $p<0.05$ for both parameters; while for pathways above the red line, $p<0.01$ for both parameters.

Supplementary Figure S2. HPV16 E6 and E7-associated genes in the Kyoto Encyclopedia of Genes and Genomes cancer pathways.

The red boxes show HPV16 E6-associated (A) and HPV16 E7-associated (B) genes that are present in the KEGG cancer pathways.

Supplementary Figure S3. Correlations between levels of HPV16 oncoproteins and downstream effectors of the master regulators.

The heat maps show expression levels of each gene in the regulons of the master regulators (rows), plotted against levels of their associated virus protein (columns), arranged from lowest (left) to highest (right). Each column is derived from a single W12 clone. As each clone was analysed in triplicate, there are three columns for each level of E6 or E7 expression. Data for HPV16 E6 are in panel A, while data for HPV16 E7 are in panel B.

Supplementary Figure S4. Networks derived following inclusion or exclusion of known p53 target genes.

(A) Network derived from genes that were both E6- and p53-associated. (B) Network derived from genes that were E6-associated but not p53-associated.

Supplementary Figure S5. The transcriptional regulatory network in W12 cells inferred from gene expression changes following depletion of HPV16 early genes (sample set 2).

Supplementary Figure S6. Overlap between data from sample sets 1 and 2.

Venn diagram showing the numbers of genes correlated with HPV16 E6 or E7 protein levels in sample set 1 (top left and top right sets, respectively, as well as those for which expression was significantly altered following HPV16 early gene depletion using siRNA in sample set 2 (bottom set).

Supplementary Figure S7. Data validation for the master regulator PA2G4 and the downstream effector gene ATL3.

(A) Correlations between gene expression levels determined by microarray (x-axis) and qRT-PCR (y-axis). (B) Representative Western blot used to quantify relative levels of PA2G4 and ATL3 proteins. All data were referenced to levels of the respective protein in the independent W12 clone E3. (C) Correlations between gene expression levels determined by qRT-PCR (x-axis) and those determined by quantitative western blotting (y-axis).

Supplementary Figure S8. Depletion of PA2G4.

Levels of PA2G4 mRNA following treatment of cervical high-grade SIL (W12 clone B) or SCC (CaSki and SiHa) cells with PA2G4 siRNAs or non-targetting control siRNAs. Expression levels were determined by qRT-PCR and referenced to those in NTC-treated cells. (B) Representative Western blot used to quantify relative levels of PA2G4 protein following treatment of cells treated with siPA2G4 or siNTC over seven days. (C) Quantification of relative PA2G4 protein levels. All data were referenced to PA2G4 protein levels in the independent W12 clone E3.

Supplementary Table S1. Genes showing significant concentration-dependent associations with levels of HPV16 E6 protein in the W12 clones. In the Table, the columns show the following: A, Illumina probe identifier; B, log fold change across the sample sets; C average expression levels across the sample sets; D, t-statistic. E, p-value for association with virus protein level; F, adjusted p-value; G, B-statistic. There are 1,213 genes showing significant concentration-dependent associations with HPV16 E6.

Supplementary Table S2. Genes showing significant concentration-dependent associations with levels of HPV16 E7 protein in the W12 clones. In the Table, the columns show the following: A, Illumina probe identifier; B, log fold change across the sample sets; C average expression levels across the sample sets; D, t-statistic. E, p-value for association with virus protein level; F, adjusted p-value; G, B-statistic. There are 1,527 genes showing significant concentration-dependent associations with HPV16 E7.

Supplementary Table S3. Genes showing significant concentration-dependent associations with levels of HPV16 E6 protein in the W12 clones, after exclusion of known p53 targets. In the Table, the columns show the following: A, Illumina probe identifier; B, log fold change across the sample sets; C average expression levels across the sample sets; D, t-statistic. E, p-value for association with virus protein level; F, adjusted p-value; G, B-statistic. There are 1,069 genes showing significant concentration-dependent associations with HPV16 E6 after exclusion of known p53 targets.

Supplementary Table S4. Genes showing significant concentration-dependent associations with levels of HPV16 E7 protein in the W12 clones, after exclusion of known p53 targets. In the Table, the columns show the following: A, Illumina probe identifier; B, log fold change across the sample sets; C average expression levels across the sample sets; D, t-statistic. E, p-value for association with virus protein level; F, adjusted p-value; G, B-statistic. There are 1,379 genes showing significant concentration-dependent associations with HPV16 E7 after exclusion of known p53 targets.

Supplementary Table S5. List of genes that were significantly differentially expressed following HPV16 early gene depletion in W12 cells. The list of 5,535 genes was derived from sample set 2. In the Table, the columns show the following: A, Illumina probe identifier; B, gene symbol; C, evidence of association with levels of HPV16 E6 protein in the W12 clones (sample set 1) (true or false); D, evidence of association with levels of HPV16 E7 protein in the W12 clones (sample set 1) (true or false). In total, 937 of the 5,535 genes derived from sample set 2 (16.9%) also showed a concentration dependent relationship with E7 and/or E6 protein in the W12 clones.

Supplementary Table S6. Primers and conditions used for qRT-PCR.

Supplementary Table S7. Antibodies used for western blotting.

Figure S1

A

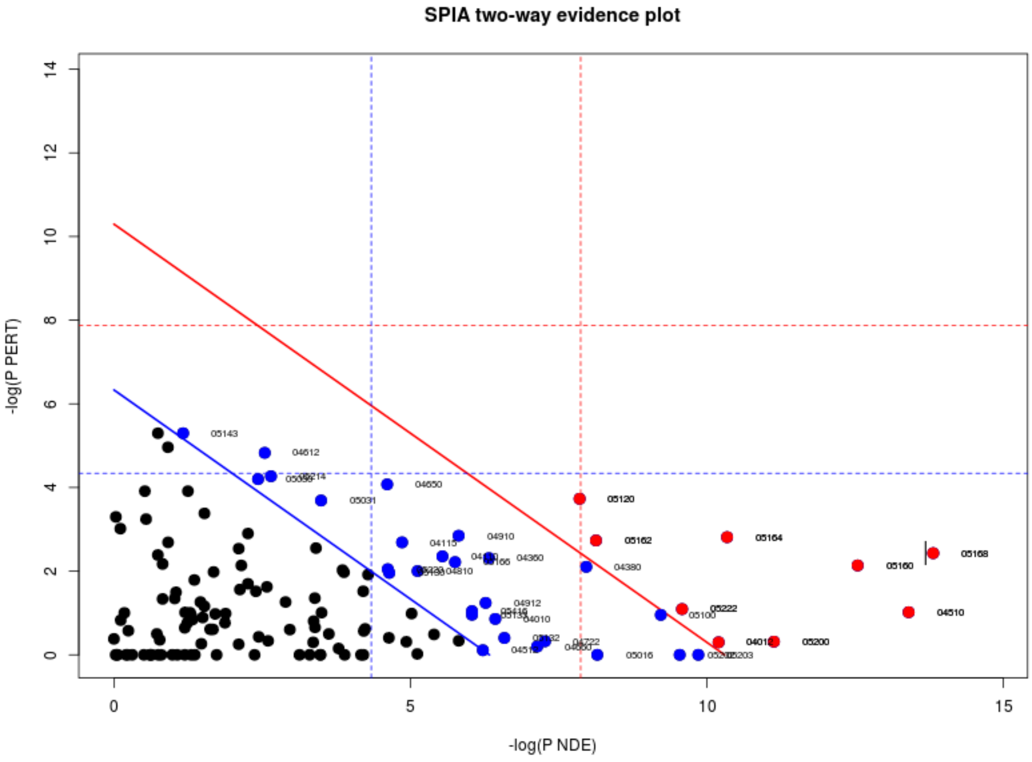
SPIA two-way evidence plot
-log(P PERT)
-log(P NDE)
05143
04612
04650
05031
05120
05162
05164
05168
05160
04510
04910
04115
04360
04380
04912
04010
05222
05100
05200
05016

B

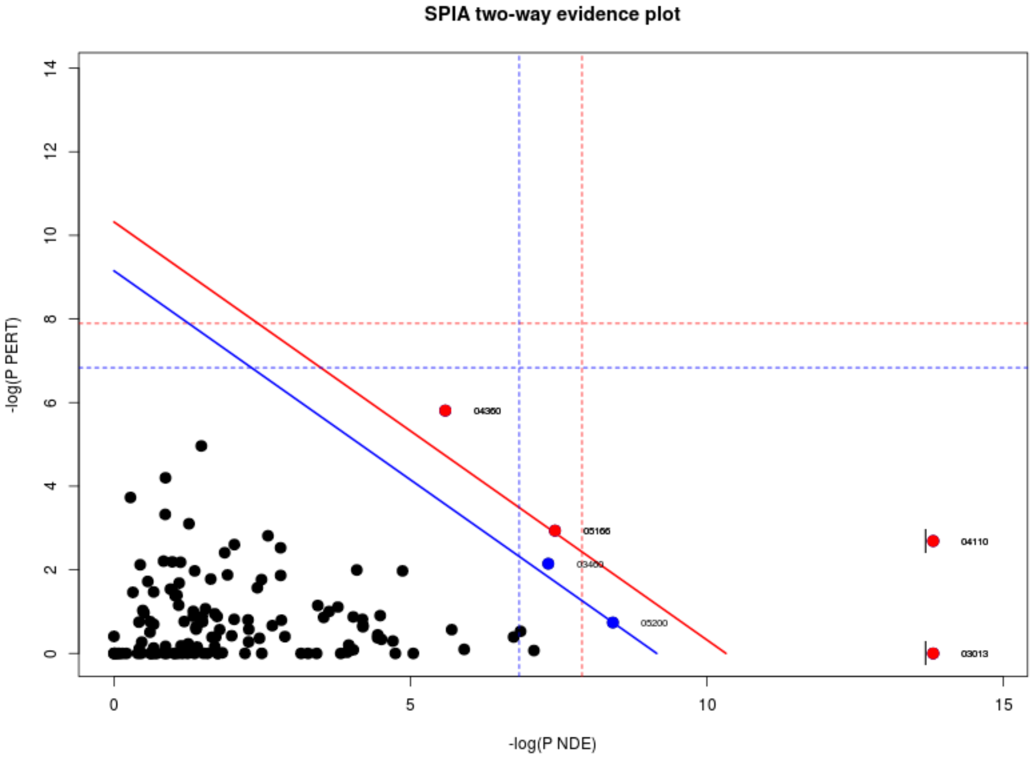
SPIA two-way evidence plot
-log(P PERT)
-log(P NDE)
04360
05166
03460
05200
04110
03013

A

Figure S2

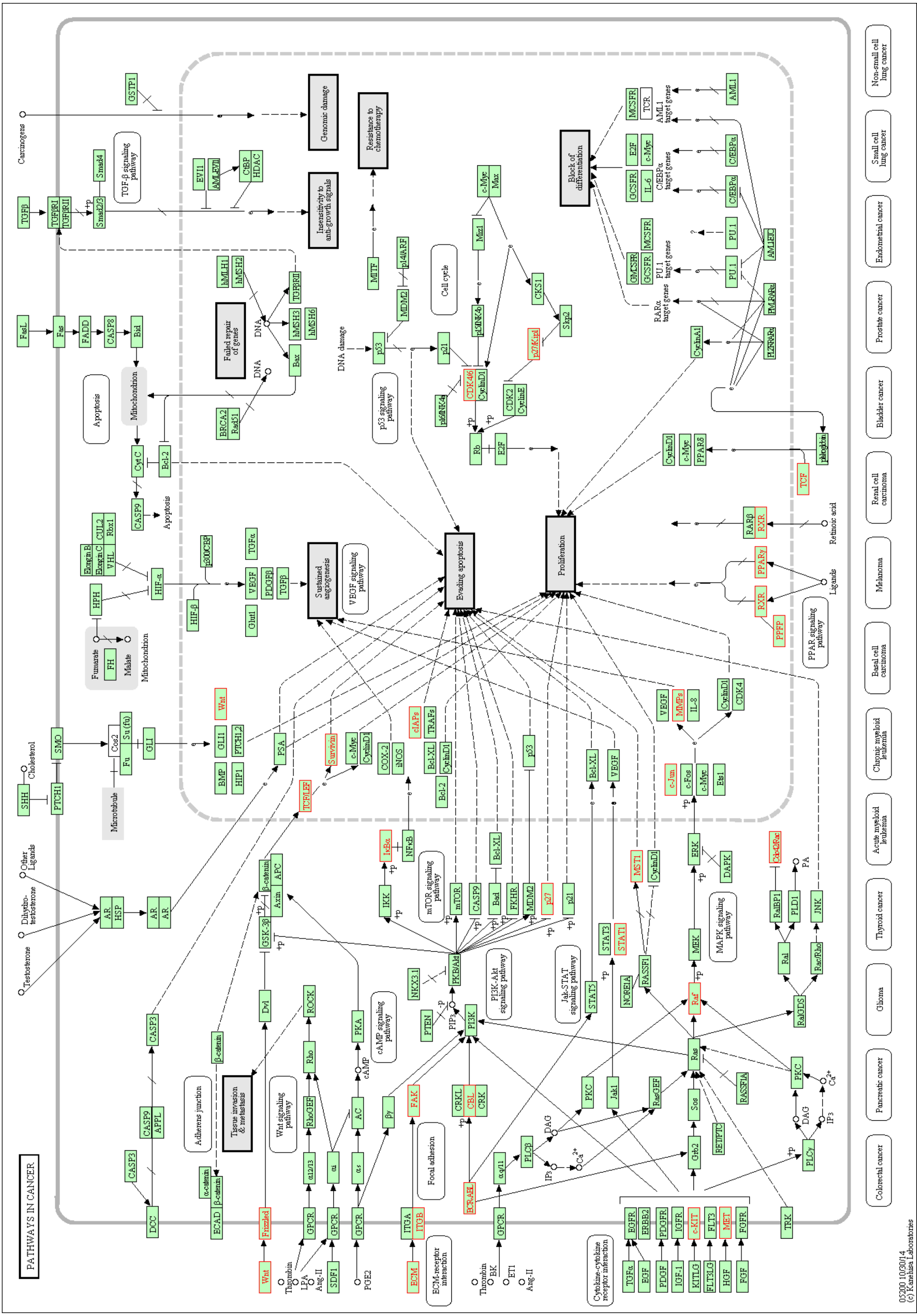

PATHWAYS IN CANCER
Genomic damage
Insensitivity to anti-growth signals
Resistance to chemotherapy
Block of differentiation
Failed repair of genes
Sustained angiogenesis
Evading apoptosis
Proliferation
Tissue invasion & metastasis
Non-small cell lung cancer
Small cell lung cancer
Endometrial cancer
Prostate cancer
Bladder cancer
Renal cell carcinoma
Melanoma
Basal cell carcinoma
Chronic myeloid leukemia
Acute myeloid leukemia
Thyroid cancer
Glioma
Pancreatic cancer
Colorectal cancer
05200 10/30/14
(c) Kanehisa Laboratories

B

Figure S2

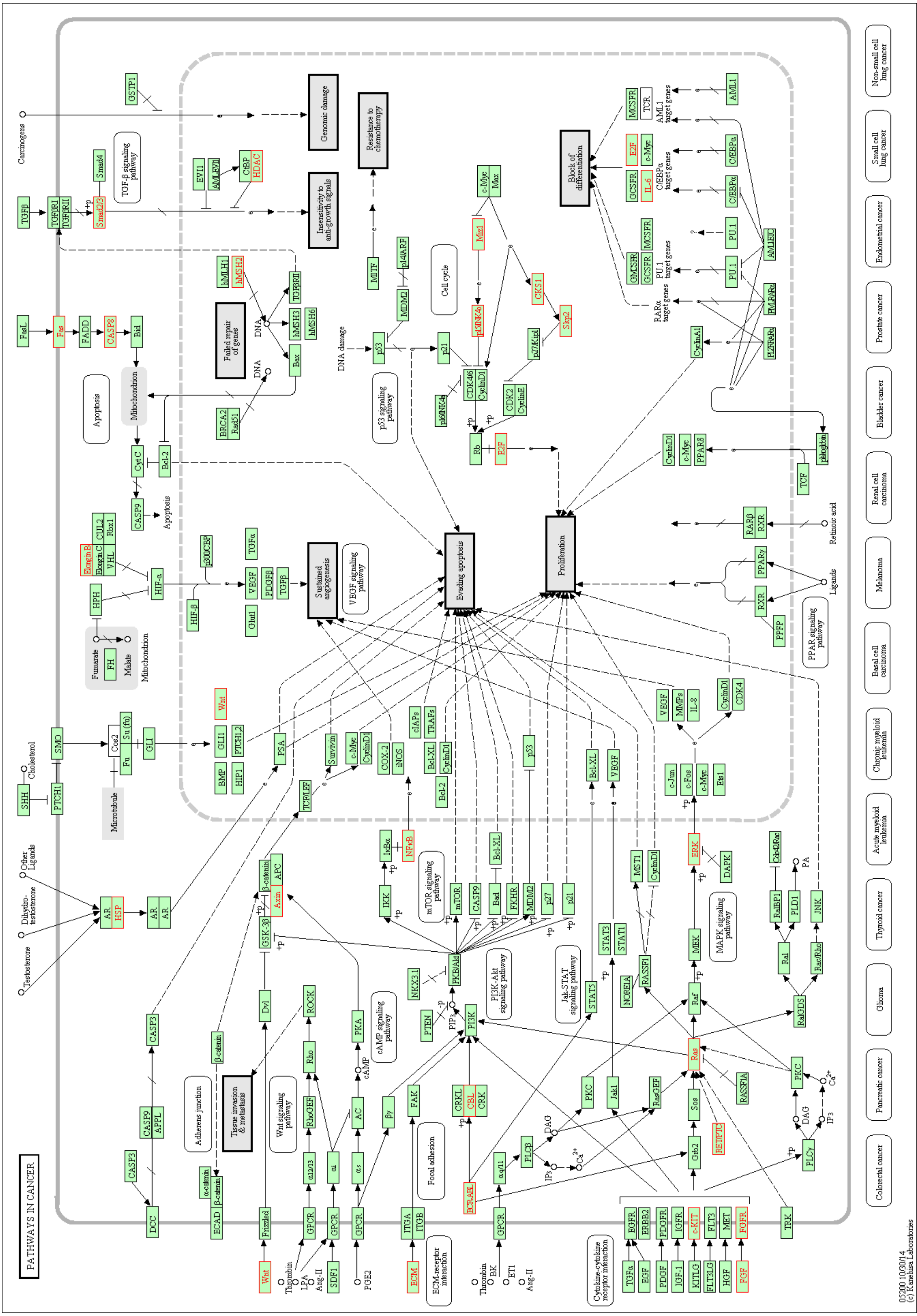
PATHWAYS IN CANCER
Colorectal cancer
Pancreatic cancer
Glioma
Thyroid cancer
Acute myeloid leukemia
Chronic myeloid leukemia
Basal cell carcinoma
Melanoma
Renal cell carcinoma
Bladder cancer
Prostate cancer
Endometrial cancer
Small cell lung cancer
Non-small cell lung cancer
05200 10/30/14
(c) Kanehisa Laboratories

Figure S3

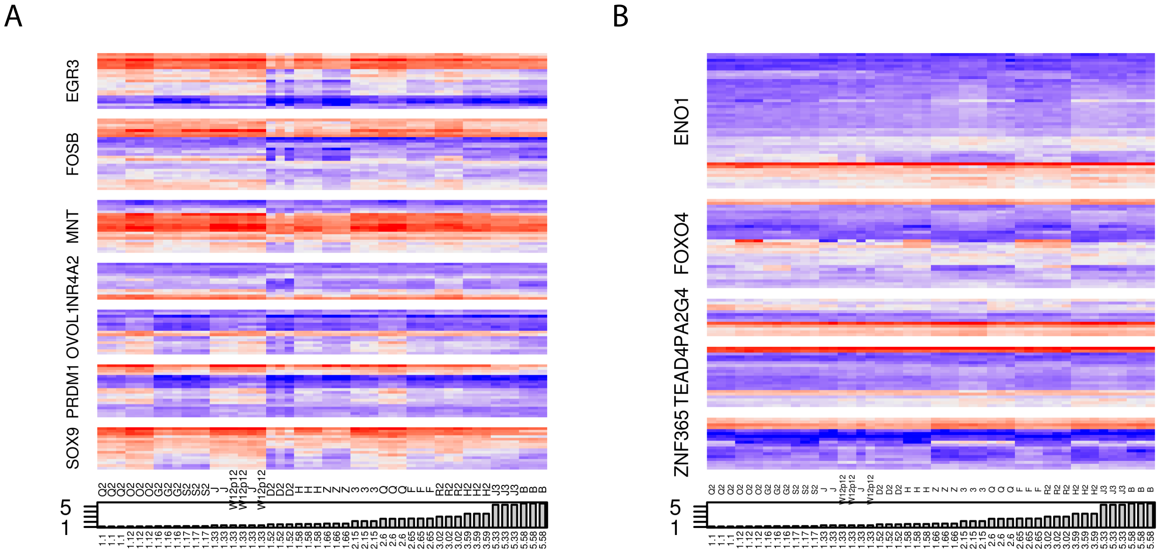

A
EGR3
FOSB
MNT
NR4A2
OVOL1
PRDM1
SOX9
5
1
B
ENO1
FOXO4
PA2G4
TEAD4
ZNF365
5
1

Figure S4

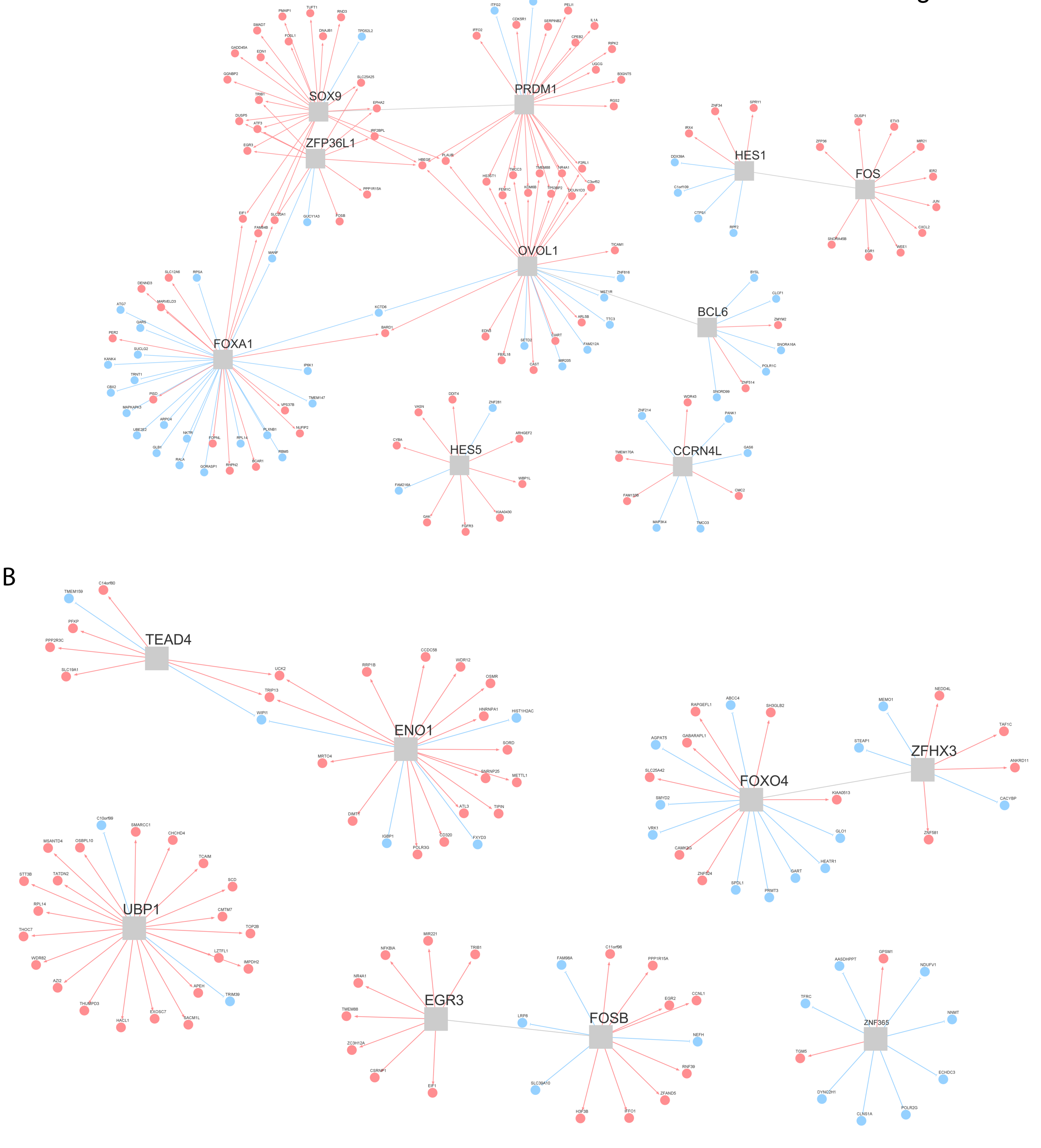

A
SOX9
PRDM1
ZFP36L1
HES1
FOS
OVOL1
FOXA1
BCL6
HES5
CCRN4L
B
TEAD4
ENO1
FOXO4
ZFHX3
UBP1
EGR3
FOSB
ZNF365

Figure S5

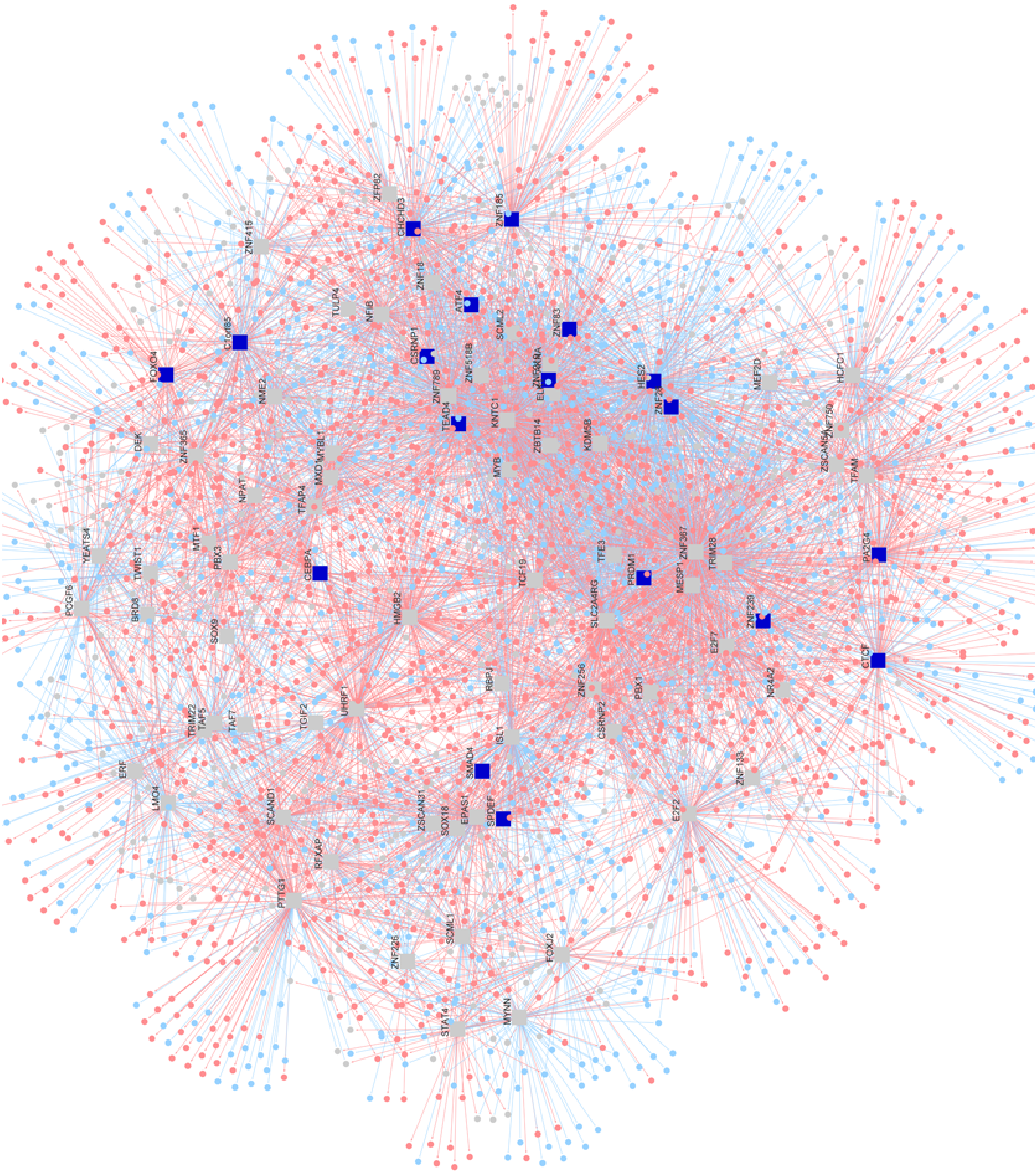

ZFP82
CHCHD3
ZNF185
ZNF415
ZNF18
TULP4
NFIB
ATF4
C1orf85
SCML2
ZNF83
CSRNP1
FOXO4
ZNF518B
HES2
MEF2D
HCFC1
NME2
ZNF789
KNTC1
ZNF750
TEAD4
DEK
ZNF365
MYBL1
ZBTB14
KDM5B
ZSCAN5A
MXD1
MYB
TFAM
NPAT
TFAP4
YEATS4
MTF1
PBX3
CEBPA
TFE3
PRDM1
ZNF367
TRIM28
PA2G4
TWIST1
TCF19
MESP1
PCGF6
BRD8
HMGB2
SLC2A4RG
ZNF239
SOX9
E2F7
CTCF
RBPJ
ZNF256
PBX1
NR4A2
TRIM22
TAF5
TAF1
TGIF2
UHRF1
ISL1
CSRNP2
ERF
SMAD4
ZNF133
LMO4
SCAND1
ZSCAN31
SOX18
EPAS1
SPDEF
E2F2
RFXAP
PTTG1
SCML1
ZNF226
FOXJ2
STAT4
MYNN

Figure S6

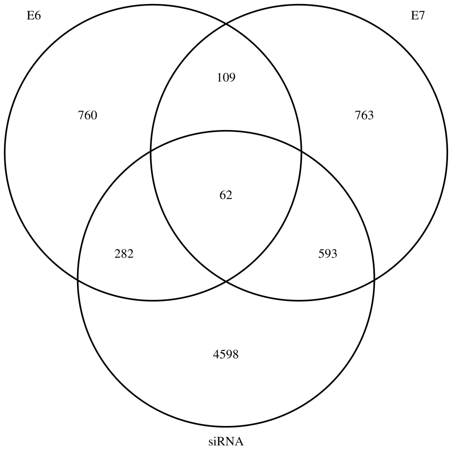

Figure S7

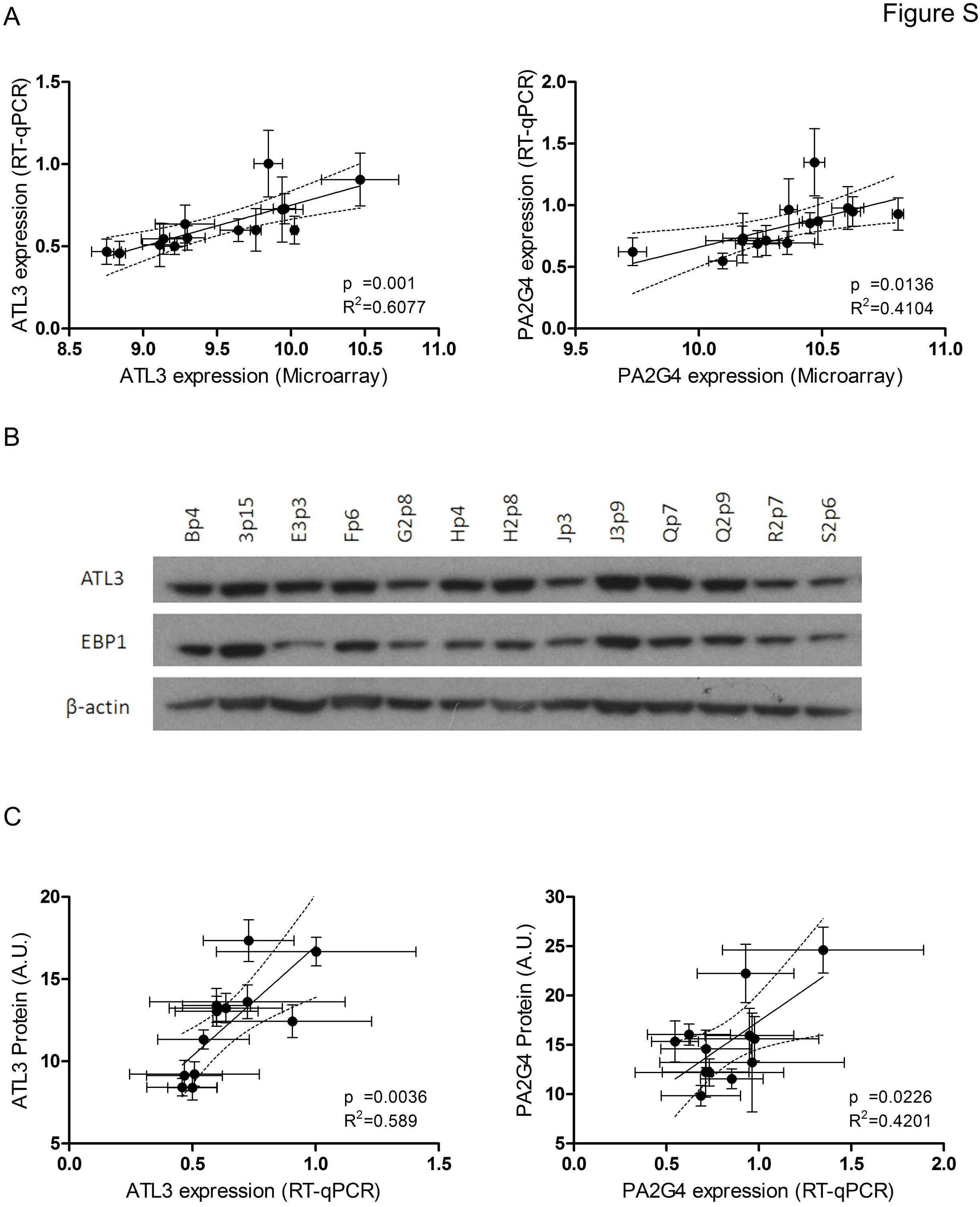
A
ATL3 expression (RT-qPCR)
ATL3 expression (Microarray)
p =0.001
R2=0.6077
PA2G4 expression (RT-qPCR)
PA2G4 expression (Microarray)
p =0.0136
R2=0.4104
B
Bp4
3p15
E3p3
Fp6
G2p8
Hp4
H2p8
Jp3
J3p9
Qp7
Q2p9
R2p7
S2p6
ATL3
EBP1
β-actin
C
ATL3 Protein (A.U.)
ATL3 expression (RT-qPCR)
p =0.0036
R2=0.589
PA2G4 Protein (A.U.)
PA2G4 expression (RT-qPCR)
p =0.0226
R2=0.4201

Figure S8

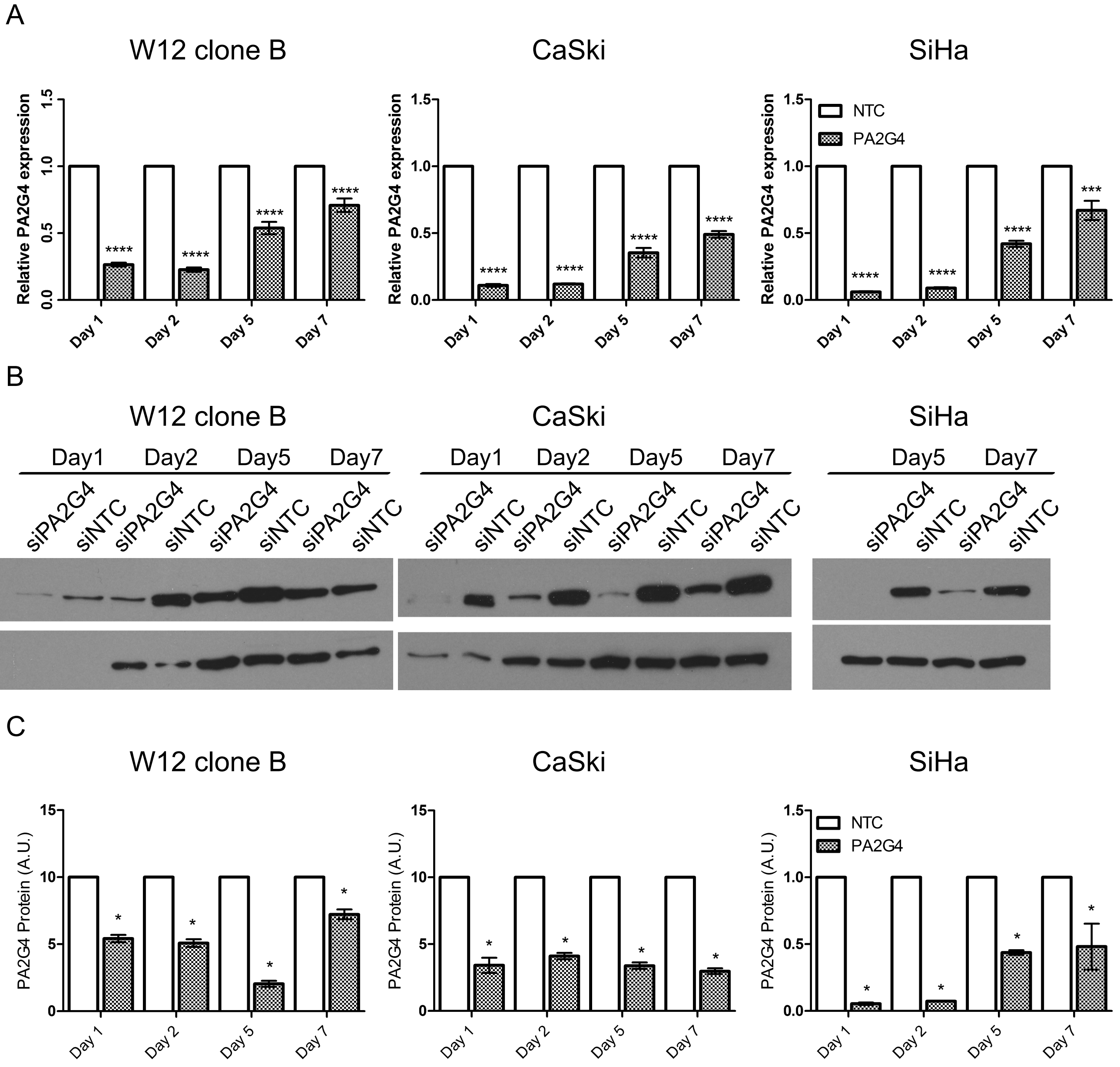
A
W12 clone B
CaSki
SiHa
Relative PA2G4 expression
NTC
PA2G4
****
***
Day 1
Day 2
Day 5
Day 7
B
W12 clone B
CaSki
SiHa
Day1
Day2
Day5
Day7
siPA2G4
siNTC
C
PA2G4 Protein (A.U.)
*

Supplementary Table S1. Genes showing significant concentration-dependent associations with levels of HPV16 E6 protein in the W12 clones.

	logFC	AveExpr	t	P.Value	adj.P.Val	B
ILMN_1768176	-1.03382	9.695149	-8.95765	5.63E-12	1.94E-07	16.72703
ILMN_2115125	-0.94747	10.6213	-5.82064	4.14E-07	0.000188	6.323643
ILMN_1762899	-0.87966	11.10572	-6.27406	8.15E-08	6.25E-05	7.840583
ILMN_1718387	0.807518	7.067937	4.841749	1.28E-05	0.001949	3.122794
ILMN_1743199	-0.73382	8.006812	-5.5079	1.26E-06	0.000411	5.286692
ILMN_2096372	0.704396	6.843431	5.770864	4.94E-07	0.000216	6.157963
ILMN_1751607	-0.69388	8.653658	-4.02154	0.000195	0.012565	0.596512
ILMN_1693192	0.668337	10.53244	3.590024	0.000752	0.030491	-0.6419
ILMN_2188264	-0.63646	9.77076	-5.06548	5.91E-06	0.001252	3.841039
ILMN_1795930	-0.60207	8.793098	-4.89311	1.07E-05	0.001805	3.286776
ILMN_1693338	0.59907	8.093769	4.561861	3.30E-05	0.003717	2.239748
ILMN_1656501	-0.58408	10.91612	-4.63926	2.54E-05	0.003133	2.482046
ILMN_1709348	0.573429	6.659982	6.073941	1.67E-07	0.000101	7.16964
ILMN_1801307	-0.56904	7.782393	-6.39114	5.35E-08	4.99E-05	8.233819
ILMN_1682775	-0.55521	9.613886	-6.35117	6.18E-08	5.32E-05	8.099527
ILMN_1705750	0.547466	9.719861	4.659485	2.38E-05	0.003001	2.545607
ILMN_2374865	-0.54036	8.426554	-4.89263	1.07E-05	0.001805	3.28525
ILMN_1687768	-0.51624	10.72609	-4.09663	0.000153	0.010842	0.819255
ILMN_2121408	-0.51066	8.642341	-4.06981	0.000167	0.01144	0.739487
ILMN_1682717	-0.50735	10.41325	-7.48282	1.05E-09	6.22E-06	11.89755
ILMN_2067656	-0.5027	11.18529	-3.69888	0.000538	0.02452	-0.33665
ILMN_1796316	0.50097	6.699623	3.60312	0.000722	0.029718	-0.60545
ILMN_1725338	-0.48667	7.689723	-5.26734	2.93E-06	0.000776	4.496938
ILMN_2166457	-0.47841	7.79733	-4.92266	9.68E-06	0.001702	3.381389
ILMN_1810835	-0.47277	11.7065	-3.40852	0.001298	0.042085	-1.13906
ILMN_1718977	-0.47091	8.552016	-6.83648	1.07E-08	1.95E-05	9.73125
ILMN_1722781	-0.46471	7.165493	-4.85394	1.23E-05	0.00192	3.16166
ILMN_1744604	0.45631	7.452409	3.464024	0.0011	0.037789	-0.98865
ILMN_1737406	-0.45431	10.1444	-6.67892	1.90E-08	2.84E-05	9.201421
ILMN_2098446	-0.44875	9.193866	-4.69937	2.08E-05	0.002763	2.671261
ILMN_1655077	-0.44437	8.529047	-3.93805	0.000254	0.015076	0.351213
ILMN_1759513	-0.44274	10.91744	-5.0875	5.48E-06	0.001202	3.912239
ILMN_1702691	-0.43746	8.552129	-3.84523	0.000341	0.018239	0.081581
ILMN_3310326	-0.43645	8.573468	-6.20962	1.03E-07	7.38E-05	7.624351
ILMN_1682636	-0.41617	7.259545	-4.82506	1.35E-05	0.00201	3.069632
ILMN_1734276	0.416054	8.66952	6.681001	1.88E-08	2.84E-05	9.208426
ILMN_1803811	-0.41345	10.1098	-4.89073	1.08E-05	0.001808	3.279161
ILMN_1791726	0.412745	7.649643	4.950285	8.80E-06	0.001614	3.469978
ILMN_1756417	-0.41226	8.04264	-6.432	4.62E-08	4.55E-05	8.371157
ILMN_1730223	-0.40347	8.039239	-5.23925	3.23E-06	0.000815	4.40528
ILMN_2404688	-0.40191	7.339713	-5.61461	8.60E-07	0.000323	5.639362
ILMN_2150851	-0.39394	11.4861	-3.98027	0.000222	0.013772	0.474933
ILMN_2372082	-0.39154	6.926074	-4.68676	2.17E-05	0.002838	2.631475
ILMN_1791728	-0.3885	8.964567	-3.79311	0.000402	0.020087	-0.06838
ILMN_2401873	-0.38456	8.387228	-7.0614	4.78E-09	1.50E-05	10.48682
ILMN_2407389	-0.384	8.461271	-3.79291	0.000402	0.020087	-0.06896
ILMN_1789400	0.375427	7.093376	4.051956	0.000177	0.011939	0.6865
ILMN_2197365	-0.37526	11.64348	-3.9879	0.000217	0.013562	0.497356
ILMN_1687978	-0.3748	9.9823	-4.05453	0.000175	0.011904	0.694121
ILMN_1810560	-0.37449	6.721196	-5.96641	2.46E-07	0.000126	6.809959

ILMN_1692056	0.374319	7.887779	3.323063	0.00167	0.048443	-1.36774
ILMN_1671891	0.372118	7.065516	6.178178	1.15E-07	8.09E-05	7.518901
ILMN_3236367	-0.36952	9.576271	-4.43486	5.05E-05	0.004975	1.845619
ILMN_1801205	-0.36816	9.373757	-3.36587	0.001472	0.044915	-1.25363
ILMN_2091375	-0.36729	7.420736	-6.09985	1.52E-07	9.55E-05	7.256412
ILMN_2150856	-0.36279	12.20645	-4.226	0.000101	0.008103	1.207578
ILMN_1703123	-0.36254	9.308187	-4.86858	1.17E-05	0.001878	3.208387
ILMN_2348268	-0.36098	7.610188	-4.39821	5.70E-05	0.005463	1.732707
ILMN_1728677	-0.35577	6.90659	-4.31564	7.50E-05	0.006573	1.479802
ILMN_1815023	-0.35031	9.555022	-6.59904	2.53E-08	3.23E-05	8.93277
ILMN_2401878	-0.34689	8.20921	-7.28301	2.15E-09	8.24E-06	11.22969
ILMN_1717706	-0.3424	8.759393	-4.72462	1.91E-05	0.002638	2.750992
ILMN_2255133	-0.34159	7.332635	-3.91723	0.000272	0.015534	0.290434
ILMN_1660000	-0.34137	7.522968	-5.25443	3.06E-06	0.000792	4.454812
ILMN_1781285	-0.34061	10.96082	-4.56122	3.31E-05	0.003717	2.237736
ILMN_1685663	0.340197	5.625189	6.886444	8.98E-09	1.76E-05	9.899184
ILMN_1772910	0.337649	8.398031	5.237567	3.25E-06	0.000815	4.399786
ILMN_1735014	-0.33475	11.25498	-5.38421	1.94E-06	0.000582	4.879647
ILMN_2410826	-0.33279	7.822715	-3.70087	0.000535	0.024402	-0.33102
ILMN_2318643	-0.33244	9.45439	-5.55689	1.06E-06	0.000368	5.448442
ILMN_2062701	0.331769	7.849424	5.899252	3.12E-07	0.00015	6.585714
ILMN_1781400	0.325712	6.351035	4.663803	2.34E-05	0.002972	2.559191
ILMN_1770612	-0.32517	10.70086	-4.47832	4.37E-05	0.004538	1.979976
ILMN_1736670	-0.32481	9.506501	-3.82372	0.000365	0.019051	0.01957
ILMN_2041190	-0.3207	10.1174	-6.13422	1.35E-07	9.11E-05	7.371555
ILMN_1745620	-0.32063	8.115913	-5.10287	5.19E-06	0.001155	3.962017
ILMN_2410145	-0.31926	6.066737	-3.90337	0.000284	0.015977	0.250072
ILMN_1782305	-0.3176	7.577045	-3.58188	0.000771	0.031037	-0.66453
ILMN_1665510	-0.31718	11.87829	-5.21583	3.50E-06	0.000863	4.328925
ILMN_1680139	-0.31587	8.145183	-4.52179	3.78E-05	0.004096	2.114932
ILMN_2318638	-0.31557	10.52254	-5.0145	7.05E-06	0.001405	3.676507
ILMN_1670841	0.313602	8.521877	6.700949	1.75E-08	2.84E-05	9.275515
ILMN_1771841	-0.31279	7.982244	-3.96966	0.00023	0.01406	0.443777
ILMN_1664922	0.310727	10.29266	4.153402	0.000127	0.009448	0.988979
ILMN_1760990	0.310301	6.710187	4.838381	1.29E-05	0.001963	3.11206
ILMN_1672295	-0.30925	8.098478	-4.56162	3.31E-05	0.003717	2.238984
ILMN_1669046	-0.30916	10.18236	-4.91511	9.93E-06	0.00173	3.357196
ILMN_1702127	0.30797	5.68991	6.32169	6.87E-08	5.64E-05	8.000517
ILMN_1800317	0.306635	6.100468	4.539929	3.56E-05	0.003963	2.171373
ILMN_1769201	-0.30486	7.974047	-3.60744	0.000713	0.02954	-0.59341
ILMN_1660067	-0.30206	7.137712	-4.27108	8.68E-05	0.007193	1.34415
ILMN_2203950	-0.30157	11.32692	-7.38567	1.49E-09	7.32E-06	11.57312
ILMN_1778401	-0.30125	7.738824	-3.57479	0.000787	0.031452	-0.68421
ILMN_1752899	-0.30078	7.611663	-4.66345	2.34E-05	0.002972	2.558078
ILMN_1806023	-0.29735	10.0989	-3.5611	0.000821	0.032122	-0.72215
ILMN_1695590	-0.29641	9.956428	-7.03107	5.33E-09	1.53E-05	10.38502
ILMN_2094776	-0.29494	8.956537	-4.36015	6.47E-05	0.005918	1.615886
ILMN_1700978	-0.29442	6.835207	-4.38882	5.89E-05	0.005605	1.703833
ILMN_1699354	-0.29011	8.363614	-4.34815	6.73E-05	0.006091	1.57914
ILMN_1735045	0.28999	7.475736	3.945307	0.000249	0.014858	0.372428
ILMN_2309156	0.289719	7.857143	5.52225	1.19E-06	0.000403	5.334043
ILMN_1680814	-0.28691	8.194471	-3.50523	0.000972	0.035267	-0.87604
ILMN_1693341	-0.28327	7.031799	-4.70825	2.01E-05	0.002728	2.699274
ILMN_1750062	0.282182	6.425835	3.937477	0.000255	0.015076	0.349533

ILMN_2404746	-0.28108	7.008499	-5.04043	6.45E-06	0.00133	3.760124
ILMN_2396020	-0.27892	10.40616	-3.37148	0.001448	0.044612	-1.23861
ILMN_2241124	-0.27586	7.144788	-3.6033	0.000722	0.029718	-0.60495
ILMN_1769520	-0.27388	8.983307	-3.53169	0.000897	0.03384	-0.80333
ILMN_1683475	0.273863	10.1368	6.880086	9.18E-09	1.76E-05	9.877815
ILMN_2148469	0.273003	8.810091	4.903693	1.03E-05	0.001773	3.32064
ILMN_1680132	0.271115	6.11318	4.746584	1.77E-05	0.002488	2.820471
ILMN_1802205	-0.27019	9.479563	-4.63939	2.54E-05	0.003133	2.482441
ILMN_1656057	-0.26939	10.21881	-6.06561	1.72E-07	0.000102	7.14174
ILMN_1710495	-0.269	7.138977	-3.96284	0.000235	0.014201	0.42378
ILMN_1683129	-0.26888	8.083049	-4.57886	3.12E-05	0.003609	2.292819
ILMN_1734929	-0.26869	9.387229	-4.28463	8.30E-05	0.006963	1.385333
ILMN_2327947	-0.26672	6.516779	-4.68176	2.20E-05	0.002867	2.615739
ILMN_1721218	-0.26379	13.55456	-5.03088	6.66E-06	0.001345	3.729314
ILMN_1740466	-0.26359	8.466511	-3.6012	0.000727	0.029821	-0.61081
ILMN_1798826	0.263082	7.326562	5.572684	9.98E-07	0.000355	5.50065
ILMN_2307025	0.262328	7.798741	5.889069	3.24E-07	0.000151	6.551741
ILMN_1675640	-0.26063	7.865586	-3.61071	0.000706	0.029332	-0.58429
ILMN_1776936	0.259812	6.817782	3.38528	0.00139	0.043808	-1.20161
ILMN_1778360	0.259386	10.85475	4.363111	6.41E-05	0.005894	1.624952
ILMN_1814215	0.258621	7.380656	3.748624	0.000461	0.022006	-0.19548
ILMN_1756469	-0.25781	6.617041	-4.71178	1.99E-05	0.002726	2.710435
ILMN_1741159	-0.25759	6.520576	-4.63418	2.59E-05	0.003165	2.466102
ILMN_1672094	0.25669	6.754803	5.644283	7.75E-07	0.0003	5.737665
ILMN_2108735	0.256349	6.308979	3.902732	0.000285	0.015983	0.24822
ILMN_1708934	-0.25553	11.66936	-3.83767	0.000349	0.018505	0.059764
ILMN_2117171	-0.25546	10.7705	-6.36585	5.86E-08	5.32E-05	8.148862
ILMN_1765860	-0.25538	6.948261	-4.13747	0.000134	0.009865	0.941244
ILMN_1794595	-0.25387	7.065567	-5.19469	3.77E-06	0.000903	4.260095
ILMN_1652580	0.253064	7.794553	8.102251	1.15E-10	1.32E-06	13.95083
ILMN_1769839	0.25285	5.604133	6.128481	1.38E-07	9.11E-05	7.352318
ILMN_1707350	0.251547	7.336674	4.707798	2.02E-05	0.002728	2.697848
ILMN_1778561	-0.25082	9.533894	-4.57055	3.21E-05	0.003674	2.266872
ILMN_2339955	-0.25069	7.058355	-3.59767	0.000734	0.030072	-0.62061
ILMN_1703180	-0.25033	7.621498	-3.922	0.000268	0.015534	0.304351
ILMN_1703891	-0.24998	8.40525	-7.06577	4.70E-09	1.50E-05	10.50148
ILMN_1767365	-0.24925	7.134126	-5.31627	2.46E-06	0.000708	4.656915
ILMN_1745471	-0.24804	9.161866	-3.3681	0.001462	0.044858	-1.24766
ILMN_1708130	-0.24728	8.245502	-5.02869	6.71E-06	0.001346	3.722271
ILMN_2189668	-0.24719	7.489654	-3.68932	0.000555	0.024798	-0.36367
ILMN_1716988	-0.24717	8.836059	-3.36154	0.001491	0.045292	-1.26521
ILMN_1671895	0.245309	6.998514	6.332541	6.61E-08	5.56E-05	8.036961
ILMN_3268403	0.244386	5.923422	8.654311	1.63E-11	2.81E-07	15.75172
ILMN_2086077	-0.2431	8.675336	-3.43538	0.001198	0.039828	-1.06647
ILMN_2326712	-0.24246	7.108977	-4.47627	4.40E-05	0.004541	1.973628
ILMN_1797236	0.241859	6.569355	4.725654	1.90E-05	0.002638	2.754254
ILMN_1742166	0.240845	8.381935	5.346771	2.21E-06	0.000652	4.756829
ILMN_1669888	-0.23976	9.58316	-4.08131	0.000161	0.011128	0.773665
ILMN_2413158	0.239509	6.450866	3.452644	0.001138	0.038458	-1.01961
ILMN_1716446	-0.23767	7.655153	-4.80927	1.43E-05	0.002095	3.019381
ILMN_1694075	-0.23588	9.968446	-3.66899	0.00059	0.025993	-0.42097
ILMN_1675616	-0.23565	6.875555	-3.89156	0.000295	0.016344	0.215735
ILMN_2130411	0.233589	9.58704	4.65257	2.43E-05	0.003061	2.523861
ILMN_1765876	-0.23238	6.010988	-4.58813	3.02E-05	0.003534	2.321806

ILMN_1706505	0.230522	7.822645	3.455239	0.001129	0.038349	-1.01256
ILMN_1744239	-0.23034	7.234171	-6.78063	1.31E-08	2.27E-05	9.543456
ILMN_3310840	-0.22884	7.508302	-3.93366	0.000258	0.015209	0.338395
ILMN_2048633	-0.2287	6.738295	-5.3857	1.93E-06	0.000582	4.88454
ILMN_1800225	0.228522	7.195323	3.5409	0.000873	0.033133	-0.77795
ILMN_1815079	-0.22815	7.332429	-3.51247	0.000951	0.034911	-0.8562
ILMN_1675992	-0.22797	7.571294	-5.50029	1.29E-06	0.000412	5.261591
ILMN_2391264	0.227488	9.11132	3.701895	0.000533	0.024369	-0.32812
ILMN_2185563	-0.22723	8.221271	-6.61973	2.35E-08	3.11E-05	9.002351
ILMN_2412922	-0.22698	6.88533	-5.93931	2.71E-07	0.000137	6.719428
ILMN_1674620	-0.22676	8.612633	-4.50185	4.04E-05	0.004285	2.052949
ILMN_1778319	0.225956	9.088586	3.50222	0.000981	0.035476	-0.8843
ILMN_1813489	0.225848	7.79926	6.313999	7.06E-08	5.66E-05	7.97469
ILMN_2229379	-0.22567	6.13363	-3.56712	0.000806	0.03191	-0.70546
ILMN_1758213	0.225133	7.772904	5.891516	3.21E-07	0.000151	6.559904
ILMN_1759184	0.22512	9.801106	4.806981	1.44E-05	0.002102	3.012107
ILMN_1736729	-0.22441	6.390611	-3.36988	0.001455	0.044742	-1.2429
ILMN_1763091	-0.22434	9.192508	-4.57329	3.18E-05	0.003665	2.275425
ILMN_1701837	-0.22393	8.208807	-6.43845	4.51E-08	4.55E-05	8.392816
ILMN_2285404	0.223459	9.479853	3.969103	0.000231	0.01406	0.442146
ILMN_3247848	0.223135	6.89447	4.776816	1.60E-05	0.002291	2.91629
ILMN_1786429	-0.22311	8.409264	-3.50608	0.000969	0.035251	-0.87372
ILMN_1720373	0.222294	11.35699	4.863951	1.18E-05	0.001881	3.193611
ILMN_1683127	-0.22173	9.525091	-3.60572	0.000717	0.029624	-0.5982
ILMN_2103397	0.221546	6.499243	5.637934	7.92E-07	0.000303	5.716625
ILMN_1805064	-0.2211	7.068874	-3.54303	0.000867	0.033102	-0.77207
ILMN_2191568	0.221059	8.435229	5.503112	1.28E-06	0.000411	5.270902
ILMN_1738759	0.220462	8.423502	7.643064	5.90E-10	5.09E-06	12.43144
ILMN_1719204	0.220104	9.238663	6.026292	1.98E-07	0.000112	7.010176
ILMN_1717765	-0.21715	7.339418	-3.44676	0.001158	0.03905	-1.0356
ILMN_1800573	0.215836	11.20054	4.014893	0.000199	0.012768	0.576886
ILMN_1669113	0.215551	7.508813	5.291409	2.69E-06	0.000748	4.575597
ILMN_1705302	0.2149	6.800363	3.743804	0.000468	0.022277	-0.2092
ILMN_3223181	0.214885	8.419154	4.188107	0.000114	0.008774	1.093256
ILMN_1762275	0.214858	7.5074	5.87017	3.47E-07	0.000159	6.488707
ILMN_1738552	-0.21463	8.427499	-4.59465	2.96E-05	0.003492	2.342214
ILMN_1844611	0.214577	6.621864	6.353854	6.12E-08	5.32E-05	8.108555
ILMN_1680453	0.214413	7.924674	4.24876	9.34E-05	0.007589	1.276446
ILMN_2168520	-0.21417	7.828221	-3.7321	0.000486	0.022942	-0.24248
ILMN_2052208	-0.2141	10.47724	-3.39234	0.001361	0.043138	-1.18262
ILMN_1702609	-0.21403	8.422294	-4.40067	5.66E-05	0.005449	1.740287
ILMN_1686555	0.213184	6.820999	4.425712	5.21E-05	0.0051	1.8174
ILMN_1717639	-0.21317	9.87896	-3.58413	0.000765	0.030898	-0.65828
ILMN_2365595	-0.21305	9.784162	-5.19802	3.73E-06	0.000899	4.270947
ILMN_1751161	0.212183	10.99825	3.339735	0.00159	0.047297	-1.32341
ILMN_1701331	0.211983	9.805817	7.313114	1.93E-09	8.24E-06	11.33046
ILMN_2383484	0.21142	8.386668	5.809561	4.30E-07	0.000193	6.286748
ILMN_2414165	-0.21125	6.673397	-3.40719	0.001303	0.042165	-1.14266
ILMN_1722634	0.211185	10.63075	4.449875	4.80E-05	0.004816	1.891981
ILMN_1668629	0.211152	9.24065	3.597223	0.000735	0.030077	-0.62187
ILMN_2396982	0.21085	9.162116	6.050179	1.82E-07	0.000106	7.090099
ILMN_1691731	-0.21033	6.980087	-3.57931	0.000777	0.031208	-0.67165
ILMN_1724181	-0.21008	6.919197	-4.97365	8.12E-06	0.001544	3.545032
ILMN_1799890	0.209114	8.428338	4.974736	8.09E-06	0.001544	3.548524

ILMN_1740429	0.208858	12.17058	3.860684	0.000325	0.017647	0.126232
ILMN_1808713	0.208271	5.671269	4.202028	0.000109	0.008524	1.135196
ILMN_2307450	0.208056	8.411141	3.997351	0.000211	0.013255	0.525175
ILMN_2178855	0.206915	7.786853	5.127087	4.77E-06	0.001087	4.040485
ILMN_1662640	0.206595	9.417851	3.974895	0.000226	0.01391	0.459146
ILMN_1700109	0.205742	9.491388	4.03934	0.000184	0.012201	0.649131
ILMN_1679093	0.205328	9.146616	3.763036	0.000441	0.021449	-0.15439
ILMN_1672004	-0.20521	9.199863	-3.83209	0.000356	0.018691	0.043664
ILMN_1808777	0.205162	8.520059	4.503022	4.02E-05	0.004281	2.056601
ILMN_1677919	-0.20408	8.942454	-5.13596	4.63E-06	0.001068	4.069263
ILMN_1772981	0.204013	10.56743	5.902338	3.09E-07	0.00015	6.596011
ILMN_1653358	-0.20323	6.534286	-3.3976	0.00134	0.042747	-1.1685
ILMN_2074748	0.202406	6.155482	5.343068	2.24E-06	0.000655	4.744693
ILMN_1754842	0.20212	7.573756	4.868569	1.17E-05	0.001878	3.208355
ILMN_1794190	-0.20197	6.986232	-4.46534	4.56E-05	0.00468	1.939801
ILMN_1752953	0.201267	8.974141	5.614121	8.62E-07	0.000323	5.637752
ILMN_1690105	-0.20106	8.328354	-3.33759	0.0016	0.047389	-1.32912
ILMN_1775962	-0.20082	7.38938	-4.45582	4.71E-05	0.00479	1.910353
ILMN_1684964	0.200763	7.346486	4.236896	9.71E-05	0.007856	1.24052
ILMN_2381197	-0.20059	7.79262	-4.9057	1.03E-05	0.001773	3.327072
ILMN_1703330	-0.20049	8.192523	-3.91776	0.000271	0.015534	0.29198
ILMN_1736575	0.200006	8.552527	4.937363	9.20E-06	0.001652	3.428517
ILMN_1770537	0.199743	6.388881	5.586075	9.52E-07	0.000342	5.544937
ILMN_1755909	0.199555	9.400336	6.416282	4.89E-08	4.68E-05	8.318317
ILMN_2285817	-0.19951	9.961391	-3.83166	0.000356	0.018691	0.042439
ILMN_1683635	-0.19937	7.995629	-3.63033	0.000665	0.028015	-0.52947
ILMN_3235325	0.199317	6.910267	3.529837	0.000902	0.03396	-0.80844
ILMN_1693421	0.1992	9.359146	6.459711	4.18E-08	4.37E-05	8.464292
ILMN_2301083	0.19913	10.92932	4.834955	1.31E-05	0.001977	3.101144
ILMN_1796074	-0.19867	8.79714	-3.79664	0.000397	0.020014	-0.05824
ILMN_2120022	-0.19867	7.027162	-4.02655	0.000192	0.012526	0.611297
ILMN_1697218	0.198196	8.347213	5.058721	6.05E-06	0.001272	3.819194
ILMN_1676515	0.197903	7.899452	3.692502	0.000549	0.024798	-0.35467
ILMN_2357438	0.19747	9.335569	4.379106	6.08E-05	0.00571	1.674016
ILMN_1658425	0.197032	8.352206	5.248425	3.13E-06	0.000798	4.435204
ILMN_1674034	-0.19643	8.200939	-4.31745	7.45E-05	0.006553	1.485324
ILMN_1675709	0.195906	7.207505	6.653702	2.08E-08	2.87E-05	9.116617
ILMN_3233388	-0.19557	8.969333	-5.22408	3.40E-06	0.000844	4.355815
ILMN_2061732	-0.19519	9.634742	-4.02326	0.000194	0.012553	0.601599
ILMN_3247018	-0.19483	8.265002	-5.99347	2.23E-07	0.000122	6.90042
ILMN_1704793	0.194785	8.555961	4.669246	2.30E-05	0.002936	2.576319
ILMN_1676631	0.194407	6.992579	3.463795	0.0011	0.037789	-0.98928
ILMN_2208777	-0.19411	7.885326	-3.53943	0.000877	0.033207	-0.78199
ILMN_2173611	0.193725	12.17228	3.807335	0.000384	0.019694	-0.02755
ILMN_1675612	0.193238	9.179098	5.125719	4.79E-06	0.001087	4.03605
ILMN_1738681	0.19294	10.25065	6.114714	1.44E-07	9.22E-05	7.306192
ILMN_1703487	-0.19257	8.843029	-4.56564	3.26E-05	0.003717	2.251535
ILMN_2392370	0.192392	6.342761	3.595252	0.00074	0.030187	-0.62736
ILMN_1691436	-0.19208	9.590865	-4.60436	2.86E-05	0.003415	2.372605
ILMN_1764571	0.191231	10.18131	3.615479	0.000696	0.028963	-0.57098
ILMN_1656933	0.191167	6.331613	4.477424	4.38E-05	0.004538	1.977217
ILMN_1669479	0.191121	7.352698	3.574264	0.000789	0.031452	-0.68566
ILMN_1665831	0.189996	8.995949	4.524969	3.74E-05	0.004072	2.124809
ILMN_2183409	0.18901	8.259481	4.319412	7.40E-05	0.006528	1.491297

ILMN_2170814	-0.1889	6.727254	-3.31166	0.001726	0.049228	-1.39799
ILMN_1671005	-0.1888	8.024776	-5.07809	5.66E-06	0.001219	3.881787
ILMN_1656670	-0.18857	6.613323	-5.73256	5.66E-07	0.000235	6.030603
ILMN_1715401	0.188487	8.08212	3.472876	0.001071	0.03739	-0.96453
ILMN_1710752	-0.18813	8.511522	-4.88736	1.09E-05	0.001811	3.268396
ILMN_1789642	0.188095	6.892939	5.311884	2.50E-06	0.000713	4.64257
ILMN_1811195	0.188049	6.387587	4.621262	2.70E-05	0.003271	2.425565
ILMN_1694111	0.1879	8.130744	5.339762	2.27E-06	0.000657	4.733859
ILMN_1810467	0.187843	10.56074	4.848859	1.25E-05	0.001927	3.145461
ILMN_1815306	0.187757	7.293171	5.737409	5.56E-07	0.000234	6.046731
ILMN_1913510	0.187665	6.677522	4.039633	0.000184	0.012201	0.65
ILMN_1679401	0.187491	7.031703	4.375481	6.15E-05	0.005732	1.662888
ILMN_2204467	0.187172	5.783277	3.472004	0.001074	0.037399	-0.96691
ILMN_1685540	0.187144	7.832534	4.866844	1.17E-05	0.00188	3.202847
ILMN_1696585	0.18682	8.641957	3.649135	0.000627	0.027177	-0.47678
ILMN_1684873	-0.18681	6.878608	-3.71091	0.000519	0.023964	-0.3026
ILMN_1704418	-0.18673	7.854408	-3.60203	0.000725	0.029781	-0.60849
ILMN_2408566	-0.1865	6.390289	-3.49429	0.001004	0.035992	-0.90602
ILMN_1706342	0.186336	7.352996	4.104384	0.000149	0.010661	0.842382
ILMN_2048478	0.186003	6.143759	4.949481	8.83E-06	0.001614	3.467399
ILMN_1658989	0.185825	6.267611	5.972922	2.40E-07	0.000125	6.831728
ILMN_2411897	-0.18571	7.291889	-3.91383	0.000275	0.015619	0.280544
ILMN_1746494	-0.18567	7.881911	-4.30253	7.83E-05	0.006729	1.439812
ILMN_1672504	0.185439	9.839358	4.481865	4.32E-05	0.004511	1.990976
ILMN_1731720	0.18528	7.562784	5.004482	7.30E-06	0.001422	3.644236
ILMN_1671191	0.185117	10.86306	4.712733	1.98E-05	0.002726	2.713431
ILMN_1907834	-0.18486	8.321906	-3.70227	0.000533	0.024369	-0.32706
ILMN_1715476	0.184802	9.924509	4.832868	1.32E-05	0.001983	3.094494
ILMN_2045994	0.184523	8.174052	5.06525	5.91E-06	0.001252	3.840292
ILMN_1676026	0.183751	8.684089	4.929238	9.46E-06	0.001673	3.402464
ILMN_2367191	0.183402	9.505748	4.685883	2.17E-05	0.002838	2.628721
ILMN_2173294	-0.18334	6.696235	-4.37644	6.13E-05	0.005729	1.665827
ILMN_1672606	-0.18324	6.181561	-3.38014	0.001411	0.044156	-1.21541
ILMN_2366041	0.183227	7.879147	4.769074	1.64E-05	0.002343	2.891731
ILMN_1701731	0.183105	9.033921	4.410899	5.47E-05	0.005297	1.771758
ILMN_1773154	-0.18285	11.15561	-3.72109	0.000503	0.023418	-0.27374
ILMN_1804822	0.182836	8.721351	3.770641	0.000431	0.021134	-0.13267
ILMN_1762284	0.182677	5.861403	6.463156	4.13E-08	4.37E-05	8.475871
ILMN_1692790	-0.18232	8.579602	-5.20682	3.61E-06	0.000884	4.299579
ILMN_1759175	-0.18216	6.883299	-5.73779	5.56E-07	0.000234	6.047989
ILMN_1699570	0.181596	9.942182	6.074689	1.67E-07	0.000101	7.172143
ILMN_2363273	0.181107	7.012101	4.304059	7.79E-05	0.006712	1.444479
ILMN_2405680	-0.18109	7.10555	-3.62337	0.000679	0.028507	-0.54893
ILMN_2372639	-0.18043	8.914962	-3.65524	0.000616	0.026807	-0.45962
ILMN_3297880	0.179973	8.630585	4.112943	0.000145	0.010435	0.86792
ILMN_1763605	0.17932	6.9534	6.890572	8.84E-09	1.76E-05	9.913057
ILMN_1751886	-0.17897	6.981708	-3.36696	0.001467	0.044915	-1.25072
ILMN_1807833	0.17857	8.049537	6.275391	8.11E-08	6.25E-05	7.845061
ILMN_1730906	-0.17854	7.259968	-3.65798	0.000611	0.02665	-0.45194
ILMN_2410772	-0.17831	8.289186	-4.96176	8.46E-06	0.001593	3.50684
ILMN_2375360	-0.17815	7.721413	-3.7974	0.000396	0.02001	-0.05608
ILMN_1790160	-0.1778	6.571739	-3.99516	0.000212	0.013299	0.51873
ILMN_1786976	0.17774	8.587619	5.100876	5.23E-06	0.001155	3.955546
ILMN_1664608	0.177569	8.508847	4.731513	1.86E-05	0.002598	2.772779

ILMN_2278653	0.177359	6.360961	5.055751	6.11E-06	0.001277	3.809601
ILMN_2186061	-0.17718	8.656237	-3.91689	0.000272	0.015534	0.28944
ILMN_1692100	0.176841	7.14129	4.398596	5.70E-05	0.005463	1.733899
ILMN_1801442	0.176668	6.102062	4.128634	0.000138	0.010087	0.914804
ILMN_1709044	0.176641	6.638544	4.415896	5.38E-05	0.005254	1.787148
ILMN_2132458	-0.17657	6.416718	-3.43916	0.001185	0.03959	-1.0562
ILMN_1680955	0.17627	9.580702	4.00823	0.000203	0.012943	0.557232
ILMN_1688154	0.175568	6.980757	3.562912	0.000816	0.032056	-0.71712
ILMN_2334350	0.175562	8.363866	4.077985	0.000163	0.011215	0.763771
ILMN_1679655	0.175524	8.886264	3.498224	0.000992	0.035741	-0.89525
ILMN_1666078	-0.17499	7.427113	-6.21834	9.96E-08	7.31E-05	7.653598
ILMN_2201347	0.174985	7.533335	4.972513	8.15E-06	0.001544	3.541379
ILMN_1745152	0.17485	8.553972	5.507517	1.26E-06	0.000411	5.285429
ILMN_2095704	0.174738	6.640483	3.422096	0.001246	0.040958	-1.10241
ILMN_1714730	0.174704	10.85933	4.168721	0.000121	0.009087	1.034957
ILMN_2358560	-0.17463	6.421356	-4.85494	1.22E-05	0.00192	3.164861
ILMN_1794643	0.174571	7.454543	5.560479	1.04E-06	0.000367	5.460306
ILMN_1771233	0.174517	8.313767	6.470291	4.02E-08	4.37E-05	8.499858
ILMN_1736112	-0.17435	7.564912	-4.94376	9.00E-06	0.001625	3.449048
ILMN_1697363	0.174309	8.470232	3.520852	0.000927	0.034592	-0.83316
ILMN_1677534	0.17419	8.546457	3.366014	0.001472	0.044915	-1.25325
ILMN_1709634	-0.17397	7.720106	-4.56121	3.31E-05	0.003717	2.237729
ILMN_1652677	-0.17391	8.342189	-3.3957	0.001348	0.042868	-1.17359
ILMN_1729225	0.173908	6.254295	3.477504	0.001056	0.036968	-0.9519
ILMN_1722811	0.173901	7.815689	3.311124	0.001729	0.049265	-1.3994
ILMN_1802615	0.173846	9.055348	3.418623	0.001259	0.041228	-1.1118
ILMN_3236061	0.173539	6.878669	4.314972	7.51E-05	0.006573	1.47775
ILMN_1654060	-0.17348	7.42097	-4.8139	1.41E-05	0.002071	3.03412
ILMN_2324056	0.173418	9.370995	3.687029	0.000558	0.024842	-0.37013
ILMN_1816342	-0.17299	7.914214	-3.83787	0.000349	0.018505	0.060324
ILMN_1806790	0.172863	7.722227	4.051389	0.000177	0.011939	0.684819
ILMN_2120340	0.172707	8.387977	5.267454	2.92E-06	0.000776	4.497322
ILMN_1660973	-0.17264	6.450034	-3.4078	0.0013	0.042135	-1.14101
ILMN_2211800	0.172642	9.738804	3.968175	0.000231	0.01406	0.439424
ILMN_2234343	0.172509	6.551563	3.954537	0.000242	0.014507	0.399445
ILMN_1791569	0.172497	7.56294	4.336619	6.99E-05	0.006264	1.543858
ILMN_2081398	0.17235	7.795199	5.985085	2.30E-07	0.000124	6.872378
ILMN_1744046	-0.17216	7.48544	-5.01294	7.09E-06	0.001405	3.671498
ILMN_1778374	-0.17183	10.17323	-4.90213	1.04E-05	0.001773	3.315629
ILMN_1735156	0.171803	6.574918	4.449823	4.81E-05	0.004816	1.891819
ILMN_2382829	0.171691	9.007533	6.150213	1.27E-07	8.77E-05	7.425149
ILMN_2105308	0.171574	10.92409	4.590978	2.99E-05	0.003524	2.330717
ILMN_2102693	-0.17083	8.757085	-3.56228	0.000818	0.032056	-0.71886
ILMN_2102960	-0.17065	8.064303	-4.38512	5.96E-05	0.005643	1.692474
ILMN_3197097	-0.17064	8.289104	-3.7302	0.000489	0.023046	-0.24787
ILMN_1790136	0.16997	8.813088	4.323791	7.30E-05	0.006451	1.504667
ILMN_1678454	-0.16991	9.454743	-5.16909	4.12E-06	0.000967	4.176853
ILMN_1745242	-0.16991	7.429544	-4.03891	0.000184	0.012201	0.647854
ILMN_1777721	0.169788	10.37908	5.607787	8.82E-07	0.000327	5.616784
ILMN_2232166	-0.16935	10.40064	-5.59252	9.31E-07	0.000338	5.566247
ILMN_1710000	-0.16934	6.626573	-6.54454	3.08E-08	3.79E-05	8.749499
ILMN_1812856	0.16923	8.03135	7.474006	1.08E-09	6.22E-06	11.86813
ILMN_1728057	-0.1692	6.695851	-5.07963	5.63E-06	0.001219	3.886771
ILMN_1732398	0.168896	5.518001	4.264737	8.86E-05	0.007286	1.324894

ILMN_1800451	-0.16878	8.17736	-5.15291	4.36E-06	0.001016	4.124275
ILMN_1719986	-0.16868	7.162071	-3.39488	0.001351	0.042894	-1.1758
ILMN_2359453	0.168482	10.66599	4.763603	1.67E-05	0.002377	2.874385
ILMN_1792679	-0.16835	7.392167	-3.46422	0.001099	0.037789	-0.98813
ILMN_1806106	0.168007	9.807794	3.83157	0.000356	0.018691	0.042166
ILMN_1784110	-0.16771	6.786576	-3.85135	0.000335	0.018029	0.099257
ILMN_1700541	0.167671	8.967071	3.439206	0.001184	0.03959	-1.05609
ILMN_2368713	0.167403	10.02075	4.563355	3.29E-05	0.003717	2.244411
ILMN_1707783	0.166958	11.66934	3.743032	0.00047	0.022299	-0.2114
ILMN_2410771	-0.16683	8.061131	-5.50997	1.25E-06	0.000411	5.293528
ILMN_1742731	0.16672	8.820646	5.112077	5.03E-06	0.001126	3.991829
ILMN_1676984	-0.16655	6.687393	-4.85233	1.23E-05	0.001922	3.156543
ILMN_2148668	-0.1663	7.46236	-3.89921	0.000288	0.016136	0.237968
ILMN_3235647	-0.16624	8.369752	-3.35258	0.001531	0.046017	-1.28916
ILMN_1664028	0.166099	9.475459	4.855172	1.22E-05	0.00192	3.165597
ILMN_1680465	-0.16545	6.953166	-3.48612	0.001029	0.036544	-0.92836
ILMN_1735658	-0.16523	9.171766	-3.96452	0.000234	0.014175	0.428704
ILMN_1652594	0.165046	6.7758	5.268598	2.91E-06	0.000776	4.50106
ILMN_1801313	-0.16471	8.011372	-3.45476	0.001131	0.038365	-1.01385
ILMN_1700168	0.164566	6.401959	3.921981	0.000268	0.015534	0.304292
ILMN_2155708	-0.16418	5.724865	-5.53255	1.15E-06	0.000393	5.368038
ILMN_1815024	-0.164	10.93684	-4.3012	7.86E-05	0.006742	1.43576
ILMN_1761941	-0.16374	6.231543	-3.39842	0.001337	0.042702	-1.16628
ILMN_1658992	0.163676	10.34544	5.267271	2.93E-06	0.000776	4.496723
ILMN_1807291	0.16353	5.682917	3.594647	0.000741	0.030188	-0.62904
ILMN_1728710	0.16342	6.954481	3.5585	0.000827	0.032266	-0.72933
ILMN_1712035	0.163416	7.517737	3.893636	0.000293	0.01629	0.221775
ILMN_1660723	0.163269	7.815519	3.470366	0.001079	0.037464	-0.97137
ILMN_3236825	-0.16319	7.840291	-3.97283	0.000228	0.013966	0.453091
ILMN_2095759	-0.16317	8.509451	-3.95432	0.000242	0.014507	0.398819
ILMN_1733667	0.163041	7.146015	5.657224	7.40E-07	0.00029	5.780561
ILMN_1791388	0.162698	8.038381	4.974707	8.09E-06	0.001544	3.548431
ILMN_1683313	0.162422	7.73199	4.878969	1.12E-05	0.001838	3.241577
ILMN_1801105	0.162318	8.112307	4.60092	2.90E-05	0.003443	2.361829
ILMN_2142117	-0.16231	9.308538	-4.11217	0.000146	0.01044	0.865606
ILMN_2394276	-0.16208	7.018932	-5.0389	6.48E-06	0.00133	3.755186
ILMN_1714741	-0.16198	7.070954	-3.80551	0.000386	0.01977	-0.03281
ILMN_3244323	0.161878	8.420129	4.988456	7.72E-06	0.001494	3.592649
ILMN_1734138	0.16177	7.228567	4.191438	0.000113	0.008743	1.103288
ILMN_1785644	0.161693	7.199323	4.169125	0.000121	0.009087	1.036173
ILMN_1705252	0.161587	6.430172	3.725341	0.000496	0.023197	-0.26168
ILMN_1751464	-0.1612	6.77298	-3.9164	0.000273	0.015534	0.288014
ILMN_2405254	-0.16075	7.769166	-3.33529	0.001611	0.047559	-1.33525
ILMN_1803856	-0.16071	6.942534	-3.62339	0.000679	0.028507	-0.54888
ILMN_1781454	0.160641	8.591168	4.377337	6.11E-05	0.005728	1.668583
ILMN_1669703	0.160472	8.590866	3.822009	0.000367	0.019072	0.014633
ILMN_1798256	-0.16026	10.07763	-3.58664	0.00076	0.030714	-0.6513
ILMN_1681326	0.160005	5.931447	6.236458	9.33E-08	6.99E-05	7.714393
ILMN_1701243	0.160004	7.875346	4.038393	0.000185	0.012201	0.646331
ILMN_1714352	0.159676	7.136768	3.320255	0.001683	0.048567	-1.37519
ILMN_2326509	-0.15948	8.811935	-3.43285	0.001207	0.040051	-1.07331
ILMN_3239217	0.159464	6.633795	4.571844	3.19E-05	0.00367	2.270912
ILMN_1670079	-0.15928	8.026146	-3.49006	0.001017	0.036364	-0.9176
ILMN_1745885	0.159247	10.33155	4.22465	0.000101	0.00812	1.203485

ILMN_1756806	-0.15912	8.925277	-3.55267	0.000842	0.032579	-0.74544
ILMN_1801833	-0.15905	6.69055	-3.43092	0.001214	0.040167	-1.07855
ILMN_1665526	0.158704	6.424292	3.417115	0.001265	0.041319	-1.11587
ILMN_1695110	0.158563	7.942509	3.876396	0.000309	0.016928	0.171736
ILMN_1719286	0.158512	7.652052	3.963292	0.000235	0.014201	0.425102
ILMN_1702211	-0.15839	6.537614	-3.74684	0.000464	0.022098	-0.20055
ILMN_1764522	0.158016	7.828939	3.384367	0.001394	0.043886	-1.20406
ILMN_1687315	0.157577	10.13046	3.466804	0.001091	0.037673	-0.98108
ILMN_1790455	0.157531	6.554547	4.143801	0.000131	0.009706	0.960202
ILMN_1700727	-0.15706	6.382961	-4.70373	2.05E-05	0.002744	2.684995
ILMN_1721022	0.156905	11.28542	4.631292	2.61E-05	0.003184	2.457029
ILMN_1683204	0.156723	8.05968	5.907633	3.03E-07	0.000149	6.613683
ILMN_1683082	0.156607	7.593366	4.525294	3.73E-05	0.004072	2.125819
ILMN_1808591	0.156492	9.949389	4.102476	0.00015	0.010705	0.836692
ILMN_1784641	0.156322	10.54662	3.456775	0.001124	0.038324	-1.00838
ILMN_1771815	0.156272	10.19336	6.654702	2.07E-08	2.87E-05	9.119979
ILMN_1750400	-0.15614	6.36807	-3.86324	0.000322	0.017561	0.13363
ILMN_1663954	0.15608	9.208176	3.94169	0.000252	0.014928	0.361851
ILMN_1695386	-0.15588	8.615201	-5.4452	1.57E-06	0.000491	5.080094
ILMN_1797698	0.155868	7.248442	6.529965	3.25E-08	3.86E-05	8.700493
ILMN_1768391	-0.15583	6.295915	-4.49123	4.19E-05	0.004399	2.020023
ILMN_1737535	0.15535	7.809927	4.879032	1.12E-05	0.001838	3.241776
ILMN_1812940	0.155224	8.171079	4.230794	9.90E-05	0.007996	1.22206
ILMN_2407464	0.155055	8.370746	3.715812	0.000511	0.023707	-0.28871
ILMN_1770505	-0.155	6.104167	-3.63683	0.000652	0.0277	-0.51127
ILMN_1658706	-0.15463	7.564188	-3.40987	0.001292	0.041956	-1.13543
ILMN_1665797	0.15461	10.52642	3.881786	0.000304	0.016722	0.187367
ILMN_2357193	-0.15455	6.711349	-4.8304	1.33E-05	0.001991	3.086621
ILMN_2125010	-0.15444	7.894929	-4.34548	6.79E-05	0.006115	1.570943
ILMN_1716821	0.154382	8.044105	3.508274	0.000963	0.035111	-0.8677
ILMN_1737426	-0.15423	9.140574	-3.6893	0.000555	0.024798	-0.3637
ILMN_1772731	-0.15403	8.037967	-3.36542	0.001474	0.044936	-1.25485
ILMN_1751743	0.153779	7.111816	4.868853	1.16E-05	0.001878	3.209261
ILMN_3244803	0.153575	6.940247	4.372629	6.21E-05	0.00577	1.654138
ILMN_1691117	0.153522	8.642115	5.032905	6.62E-06	0.001345	3.735851
ILMN_2126239	0.153477	7.785695	3.752896	0.000455	0.021837	-0.18331
ILMN_1801616	-0.1533	12.96734	-3.32389	0.001666	0.048418	-1.36554
ILMN_1722218	0.153028	7.885802	4.298708	7.93E-05	0.00678	1.428178
ILMN_1759436	0.152915	9.846954	3.697246	0.000541	0.024611	-0.34126
ILMN_1669390	0.152882	9.29823	3.364853	0.001477	0.044971	-1.25636
ILMN_1654001	-0.15274	7.716196	-4.29692	7.97E-05	0.006787	1.422735
ILMN_2183687	0.152672	6.975102	4.950134	8.81E-06	0.001614	3.469493
ILMN_1755352	0.152606	5.878212	3.466495	0.001092	0.037673	-0.98192
ILMN_1771987	0.152459	9.357103	3.754671	0.000453	0.021777	-0.17825
ILMN_2265654	0.152282	6.254246	5.048628	6.27E-06	0.001301	3.786596
ILMN_1760490	-0.1522	8.651016	-4.7446	1.78E-05	0.002495	2.814195
ILMN_1675007	0.152128	6.451682	5.030493	6.67E-06	0.001345	3.72807
ILMN_1666385	0.152057	11.11719	3.755176	0.000452	0.021773	-0.17681
ILMN_2075820	-0.15176	7.453887	-4.79889	1.48E-05	0.002143	2.986394
ILMN_1811729	0.151555	7.58875	3.796402	0.000398	0.020014	-0.05893
ILMN_2357781	0.151529	6.984022	3.488554	0.001022	0.036428	-0.92172
ILMN_1810334	0.151444	8.569222	4.051957	0.000177	0.011939	0.686501
ILMN_1809478	0.151262	11.47705	3.355385	0.001518	0.045758	-1.28167
ILMN_1782829	0.151193	6.668202	4.027037	0.000192	0.012526	0.612747

ILMN_1711383	0.151177	9.415097	5.291891	2.68E-06	0.000748	4.577174
ILMN_1735955	0.151077	8.686113	3.319045	0.001689	0.048699	-1.37841
ILMN_1729368	0.151074	6.394802	5.910464	3.00E-07	0.000149	6.623132
ILMN_1668369	-0.15095	11.22509	-3.76059	0.000445	0.02153	-0.16137
ILMN_3248857	-0.1505	6.346806	-4.03408	0.000187	0.012277	0.633578
ILMN_2323491	0.150433	8.391228	4.828619	1.34E-05	0.001995	3.08096
ILMN_1810891	0.150375	7.421438	3.953964	0.000242	0.014507	0.397768
ILMN_2283388	0.149732	10.62316	3.942651	0.000251	0.014908	0.36466
ILMN_1776375	-0.14925	9.59331	-4.89796	1.05E-05	0.001789	3.302307
ILMN_1728059	0.149219	7.675856	4.047887	0.000179	0.012019	0.674441
ILMN_1674394	0.149054	8.769143	3.465469	0.001095	0.037751	-0.98472
ILMN_1683059	-0.14904	7.756932	-3.41532	0.001272	0.041439	-1.12073
ILMN_1765684	-0.14901	10.39111	-3.42716	0.001228	0.040541	-1.08872
ILMN_1711450	0.148957	9.412244	3.927086	0.000264	0.015398	0.319188
ILMN_1779034	-0.1489	7.691803	-4.51662	3.84E-05	0.004141	2.098855
ILMN_3225784	0.148858	12.80393	3.32925	0.00164	0.047945	-1.35131
ILMN_1770653	-0.14855	11.45875	-4.36096	6.45E-05	0.005918	1.618345
ILMN_1717809	0.148416	6.697764	3.762703	0.000442	0.021449	-0.15534
ILMN_1755862	0.147969	8.186322	3.553976	0.000839	0.032546	-0.74184
ILMN_1667594	-0.14787	6.850008	-3.71985	0.000505	0.023476	-0.27725
ILMN_1770338	-0.14774	11.67637	-3.45529	0.001129	0.038349	-1.01242
ILMN_1777449	-0.14767	7.981589	-3.37235	0.001444	0.044537	-1.23628
ILMN_1725427	-0.14763	11.94789	-3.40402	0.001315	0.042371	-1.15118
ILMN_1713006	-0.14757	7.403597	-3.93071	0.000261	0.0153	0.329753
ILMN_1793203	0.147465	7.931098	4.558502	3.34E-05	0.003739	2.229268
ILMN_2332558	-0.14715	9.264805	-4.58897	3.01E-05	0.003534	2.32445
ILMN_1710844	-0.1471	6.676328	-3.89772	0.000289	0.016159	0.23364
ILMN_2359456	0.146922	8.970854	4.048175	0.000179	0.012019	0.675296
ILMN_2173451	0.146891	9.157835	3.630748	0.000664	0.028014	-0.5283
ILMN_1792748	0.14676	6.144422	4.194433	0.000112	0.008678	1.112308
ILMN_1738229	0.146561	8.189289	4.210926	0.000106	0.008375	1.162037
ILMN_1652754	0.146215	8.936248	3.558785	0.000827	0.032266	-0.72854
ILMN_3272768	-0.14617	6.624888	-6.00888	2.11E-07	0.000117	6.951944
ILMN_1737685	0.146161	9.912017	3.336211	0.001606	0.047499	-1.33279
ILMN_1769575	0.146114	5.614811	3.64837	0.000629	0.027207	-0.47892
ILMN_1695679	-0.14602	6.135872	-3.37692	0.001425	0.044289	-1.22404
ILMN_1711023	0.145942	8.144351	4.711132	1.99E-05	0.002726	2.708372
ILMN_1792825	0.145859	7.809389	3.314944	0.00171	0.04894	-1.38928
ILMN_1676197	0.145858	10.08529	3.37078	0.001451	0.044664	-1.24049
ILMN_2065783	-0.14557	7.062967	-5.69413	6.49E-07	0.000263	5.902991
ILMN_1770035	0.14557	7.938254	6.460908	4.16E-08	4.37E-05	8.468315
ILMN_1721225	0.145562	8.673909	6.047725	1.84E-07	0.000106	7.081889
ILMN_1790537	-0.14556	8.683089	-3.32668	0.001652	0.048147	-1.35814
ILMN_3227263	-0.14552	7.69568	-3.4178	0.001262	0.04129	-1.11403
ILMN_1741585	0.145515	6.201362	4.582443	3.08E-05	0.003583	2.304029
ILMN_1767658	0.14546	8.743809	4.248171	9.36E-05	0.007589	1.274661
ILMN_2144573	-0.14531	7.407936	-6.93279	7.60E-09	1.75E-05	10.05492
ILMN_1653133	-0.14513	8.267472	-5.27953	2.80E-06	0.000773	4.53677
ILMN_3265237	-0.1451	7.012253	-3.74245	0.00047	0.022309	-0.21306
ILMN_1696190	0.145057	7.341854	5.179287	3.98E-06	0.00094	4.209998
ILMN_1661484	0.144923	6.477409	3.85755	0.000328	0.017766	0.117166
ILMN_1718069	-0.14488	9.717053	-3.65846	0.00061	0.026644	-0.4506
ILMN_1753885	0.144481	8.759723	5.668451	7.11E-07	0.000282	5.81779
ILMN_3303673	-0.14438	7.409118	-3.84674	0.00034	0.018181	0.085922

ILMN_1728972	0.144304	7.406871	4.051157	0.000177	0.011939	0.68413
ILMN_1748077	-0.14429	8.373019	-4.28818	8.20E-05	0.006902	1.396141
ILMN_1654488	-0.14408	7.884099	-4.0157	0.000199	0.012768	0.579259
ILMN_1702073	-0.14407	7.020506	-5.35732	2.13E-06	0.000634	4.791413
ILMN_1688642	0.143925	6.268217	3.549667	0.00085	0.03277	-0.75375
ILMN_2050255	0.143898	8.970775	5.739448	5.52E-07	0.000234	6.053507
ILMN_1800311	-0.14389	7.430698	-4.45751	4.68E-05	0.004777	1.915581
ILMN_1733863	-0.14314	8.329745	-3.33721	0.001602	0.047401	-1.33014
ILMN_1683533	0.143067	6.642611	4.441495	4.94E-05	0.004927	1.866095
ILMN_1729142	0.143047	6.377382	3.366584	0.001469	0.044915	-1.25173
ILMN_1692754	-0.14238	9.73637	-3.57614	0.000784	0.031437	-0.68046
ILMN_2207393	0.142323	7.21087	4.884599	1.10E-05	0.00182	3.259567
ILMN_1651228	-0.14228	13.36566	-6.97261	6.58E-09	1.62E-05	10.18869
ILMN_1765770	0.14225	6.899082	3.377593	0.001422	0.044289	-1.22223
ILMN_1690610	0.142134	8.977126	4.69125	2.13E-05	0.002819	2.645638
ILMN_1745784	0.141999	6.744723	3.693038	0.000548	0.024798	-0.35316
ILMN_1687857	0.141927	7.076059	3.472706	0.001072	0.03739	-0.96499
ILMN_3235808	0.141921	9.152855	4.948784	8.85E-06	0.001614	3.465161
ILMN_1758173	-0.14191	9.107964	-4.68629	2.17E-05	0.002838	2.630005
ILMN_1700306	-0.14173	10.1864	-3.66853	0.000591	0.025996	-0.42225
ILMN_1788547	0.14165	8.061504	3.807212	0.000384	0.019694	-0.02791
ILMN_1682935	-0.1416	8.379604	-4.12118	0.000141	0.010277	0.892533
ILMN_1804329	0.141527	6.846296	4.673088	2.27E-05	0.00293	2.588413
ILMN_1821473	0.14145	6.241595	3.751173	0.000458	0.021893	-0.18822
ILMN_1662426	0.141433	11.20601	4.307395	7.70E-05	0.006655	1.454646
ILMN_1686152	-0.14141	7.40316	-4.08174	0.000161	0.011128	0.774952
ILMN_1738263	0.141127	7.787818	4.006545	0.000205	0.012965	0.552263
ILMN_1747744	0.141	7.483208	4.014795	0.000199	0.012768	0.576597
ILMN_1666727	0.14088	7.476774	4.154949	0.000127	0.009421	0.99362
ILMN_3204734	0.140815	9.880486	3.548605	0.000853	0.032839	-0.75668
ILMN_3219340	0.14081	12.83704	3.511005	0.000955	0.035028	-0.86021
ILMN_3246608	0.140682	6.307201	3.637203	0.000651	0.0277	-0.51023
ILMN_2175112	-0.14064	6.505594	-3.54106	0.000872	0.033133	-0.7775
ILMN_1761858	0.140603	8.379168	4.71024	2.00E-05	0.002726	2.705557
ILMN_1654653	0.140549	7.652321	5.538333	1.13E-06	0.000389	5.387142
ILMN_1786893	0.140339	9.273498	3.360402	0.001496	0.045325	-1.26827
ILMN_1741371	-0.14018	6.492369	-3.9852	0.000219	0.013631	0.489427
ILMN_1715273	-0.14017	9.627349	-4.16998	0.000121	0.009087	1.038755
ILMN_1669484	0.140056	9.247776	4.217714	0.000103	0.008241	1.18253
ILMN_1689327	0.139804	11.86812	5.004905	7.29E-06	0.001422	3.645601
ILMN_2407124	0.139741	6.874062	3.499223	0.00099	0.035741	-0.89251
ILMN_1737604	-0.13969	7.910983	-3.78866	0.000407	0.020302	-0.08112
ILMN_1798712	0.13965	6.692122	3.800621	0.000392	0.019868	-0.04683
ILMN_1765649	0.139577	7.80209	4.171214	0.00012	0.009073	1.04245
ILMN_2333107	-0.13944	8.586003	-4.17249	0.00012	0.009055	1.046288
ILMN_1772124	0.139399	7.905463	3.944809	0.000249	0.014858	0.370972
ILMN_1712231	-0.13929	9.444425	-3.48691	0.001027	0.036495	-0.92621
ILMN_2387599	0.139277	11.77284	4.751618	1.74E-05	0.002466	2.836411
ILMN_1804150	-0.13909	9.13232	-4.07502	0.000164	0.0113	0.754967
ILMN_3224290	0.139012	9.645384	3.322847	0.001671	0.048443	-1.36832
ILMN_1697817	-0.13895	7.647454	-3.68422	0.000563	0.024994	-0.37805
ILMN_2364131	0.138816	8.838955	5.082808	5.57E-06	0.001214	3.897064
ILMN_1690268	0.138808	9.935304	3.543485	0.000866	0.033093	-0.77082
ILMN_1660341	0.138728	8.027969	3.399388	0.001333	0.042678	-1.16367

ILMN_1702279	0.138561	7.827281	4.450664	4.79E-05	0.004816	1.894419
ILMN_1729987	0.138557	8.692391	4.74791	1.76E-05	0.002487	2.824669
ILMN_1722294	-0.13824	7.407387	-5.3076	2.54E-06	0.000718	4.628552
ILMN_1673111	0.138104	9.131493	3.709858	0.00052	0.023987	-0.30558
ILMN_1698673	-0.13809	7.12604	-5.06507	5.92E-06	0.001252	3.839722
ILMN_1701940	0.138073	7.001941	5.804244	4.38E-07	0.000194	6.269047
ILMN_1811836	-0.13804	8.139387	-3.36865	0.00146	0.044826	-1.24619
ILMN_2369785	0.138024	12.81444	5.504852	1.27E-06	0.000411	5.27664
ILMN_1727300	0.137983	7.468963	5.199442	3.71E-06	0.000899	4.275568
ILMN_1755974	0.137799	7.42346	3.402648	0.00132	0.042432	-1.15489
ILMN_1814966	0.137439	7.410336	4.212699	0.000105	0.008346	1.167388
ILMN_1747281	-0.13743	7.329939	-4.06427	0.00017	0.011592	0.723016
ILMN_2124802	0.137381	5.790428	3.68987	0.000554	0.024798	-0.36211
ILMN_1755077	-0.13733	11.37783	-3.90675	0.000281	0.015911	0.2599
ILMN_1752283	0.137263	7.497097	5.420497	1.71E-06	0.000522	4.998849
ILMN_1803988	-0.13717	10.12275	-3.4947	0.001003	0.035992	-0.90491
ILMN_1718285	0.137054	6.144673	3.667849	0.000592	0.026018	-0.42418
ILMN_1815885	0.136963	6.751586	4.165045	0.000123	0.009166	1.023919
ILMN_1676792	0.136934	12.62773	3.357984	0.001507	0.045529	-1.27473
ILMN_1718771	-0.13689	6.72125	-3.58077	0.000773	0.031105	-0.6676
ILMN_2337336	0.136858	6.485446	3.752413	0.000456	0.021839	-0.18468
ILMN_1724734	0.136772	6.443757	5.187953	3.86E-06	0.000918	4.238183
ILMN_2317730	0.1365	7.618262	5.252908	3.08E-06	0.000792	4.449832
ILMN_1795228	-0.13648	11.03292	-3.379	0.001416	0.044216	-1.21846
ILMN_1722426	-0.13628	5.769089	-4.36667	6.33E-05	0.005854	1.63585
ILMN_1809957	0.136156	12.30113	7.000736	5.95E-09	1.58E-05	10.28316
ILMN_1804419	-0.13615	6.165495	-3.50775	0.000964	0.035112	-0.86915
ILMN_1794781	0.13611	6.908979	5.276932	2.83E-06	0.000774	4.528282
ILMN_2373266	-0.13596	8.809098	-4.96026	8.50E-06	0.001593	3.502003
ILMN_1702835	-0.13592	9.257488	-4.0138	0.0002	0.012779	0.573656
ILMN_1653501	0.135578	5.587015	5.597469	9.14E-07	0.000335	5.582632
ILMN_2347068	-0.13555	9.922888	-3.80921	0.000382	0.019658	-0.02216
ILMN_2159453	-0.13552	8.276362	-3.85559	0.00033	0.017848	0.111507
ILMN_3288755	0.135497	6.588679	3.803443	0.000389	0.01978	-0.03873
ILMN_3180557	0.135472	6.933027	5.116973	4.94E-06	0.001114	4.007695
ILMN_1670322	-0.13541	7.840022	-3.88385	0.000302	0.016653	0.19335
ILMN_1752884	0.135355	6.1438	3.553784	0.000839	0.032546	-0.74237
ILMN_1796099	0.135193	7.155118	3.460599	0.001111	0.038039	-0.99798
ILMN_1900270	-0.13517	6.401475	-3.37683	0.001425	0.044289	-1.22429
ILMN_1751615	-0.13509	8.943403	-3.4785	0.001053	0.036933	-0.94918
ILMN_1794213	0.134901	6.118721	3.477899	0.001055	0.036962	-0.95083
ILMN_1657204	0.134887	11.45826	3.508431	0.000962	0.035111	-0.86727
ILMN_1758906	-0.13487	9.010846	-5.70438	6.26E-07	0.000257	5.937022
ILMN_1761069	-0.13487	7.418122	-3.54499	0.000862	0.033017	-0.76667
ILMN_1812327	-0.13486	6.350576	-4.3575	6.53E-05	0.005954	1.607751
ILMN_1731193	0.134828	6.411773	5.253807	3.07E-06	0.000792	4.452766
ILMN_1703142	-0.13471	6.363091	-4.2932	8.07E-05	0.006853	1.411394
ILMN_1658807	-0.13456	6.922589	-4.04272	0.000182	0.01217	0.659137
ILMN_2402806	0.134361	8.329239	4.605529	2.85E-05	0.003413	2.376263
ILMN_2403555	0.134145	6.899727	3.892628	0.000294	0.016316	0.218845
ILMN_1787932	-0.1339	6.155758	-3.49438	0.001004	0.035992	-0.90576
ILMN_1658015	-0.13354	7.671465	-3.86222	0.000323	0.01759	0.130678
ILMN_1653599	-0.13353	10.79695	-3.52722	0.000909	0.034118	-0.81565
ILMN_2167922	0.133517	11.34824	4.785775	1.55E-05	0.002232	2.944725

ILMN_1777444	-0.1335	7.396279	-4.67071	2.29E-05	0.002932	2.580926
ILMN_1656066	0.133497	9.395813	4.946671	8.91E-06	0.001617	3.458379
ILMN_1778803	-0.13348	8.24874	-4.93586	9.25E-06	0.001652	3.423682
ILMN_1792078	0.133274	9.804828	4.276182	8.53E-05	0.007124	1.359651
ILMN_1654064	-0.13315	7.089103	-4.65043	2.45E-05	0.003069	2.517138
ILMN_1668834	-0.13314	6.53654	-3.69165	0.000551	0.024798	-0.35708
ILMN_1808391	-0.13303	5.873943	-3.87471	0.000311	0.016991	0.166848
ILMN_1758633	0.132913	8.386412	3.498575	0.000991	0.035741	-0.89429
ILMN_1812461	0.132912	6.393518	4.848624	1.25E-05	0.001927	3.144711
ILMN_1721704	-0.13291	9.435152	-3.37876	0.001417	0.044216	-1.21911
ILMN_1757847	-0.13289	7.089486	-4.88867	1.09E-05	0.001811	3.272596
ILMN_2063925	0.132751	8.524528	4.000071	0.000209	0.013188	0.533188
ILMN_1775486	-0.1327	5.641129	-3.63525	0.000655	0.027737	-0.51569
ILMN_1806809	0.132642	6.996511	3.6185	0.000689	0.028788	-0.56254
ILMN_1808860	-0.13262	8.62686	-5.00737	7.23E-06	0.001422	3.65353
ILMN_1770977	-0.13243	8.74774	-4.79909	1.48E-05	0.002143	2.987025
ILMN_1769734	-0.13238	9.050755	-3.89813	0.000289	0.016159	0.234841
ILMN_1651886	0.132079	9.567376	4.03667	0.000186	0.012245	0.64123
ILMN_2350574	0.132019	7.019527	3.376672	0.001426	0.044289	-1.2247
ILMN_1734827	0.131914	6.450167	4.250008	9.30E-05	0.007579	1.280227
ILMN_1715969	0.131778	7.124269	4.288041	8.21E-05	0.006902	1.395708
ILMN_1793033	0.131519	7.364432	4.025448	0.000193	0.012526	0.608052
ILMN_1678052	-0.13137	7.657149	-3.31395	0.001715	0.04894	-1.39193
ILMN_2176037	-0.13126	8.145888	-6.12338	1.40E-07	9.11E-05	7.335231
ILMN_1653896	0.131024	6.707074	3.563268	0.000815	0.032056	-0.71613
ILMN_1761722	0.130575	6.721795	4.505772	3.99E-05	0.004255	2.065141
ILMN_1784436	0.130122	7.171081	3.727791	0.000492	0.023093	-0.25473
ILMN_1747223	-0.13008	8.365961	-3.82273	0.000366	0.019071	0.016703
ILMN_1760143	0.129678	10.37174	4.386412	5.93E-05	0.005634	1.696448
ILMN_2411794	0.129596	8.508565	4.641216	2.53E-05	0.003133	2.488187
ILMN_1700337	0.129543	7.679403	3.793528	0.000401	0.020087	-0.06717
ILMN_1702866	0.12942	5.750363	5.420036	1.71E-06	0.000522	4.997331
ILMN_1748894	0.129285	8.536682	3.763282	0.000441	0.021449	-0.15369
ILMN_1764323	-0.1289	9.198453	-4.58203	3.09E-05	0.003583	2.302727
ILMN_2386100	0.128884	9.046575	3.710598	0.000519	0.023964	-0.30349
ILMN_1676759	0.128789	7.911027	4.310859	7.61E-05	0.006596	1.465206
ILMN_1771824	0.128721	6.442188	5.134613	4.65E-06	0.001068	4.064897
ILMN_1678949	0.128575	7.116892	4.427449	5.18E-05	0.005085	1.822753
ILMN_2174081	0.128539	7.115872	3.350455	0.001541	0.046176	-1.29483
ILMN_1697348	0.128284	8.438112	3.838904	0.000348	0.018491	0.063311
ILMN_1693145	0.128204	11.4118	3.904125	0.000283	0.015964	0.252274
ILMN_1714364	-0.12809	11.31818	-3.66388	0.0006	0.026271	-0.43535
ILMN_1672947	-0.12781	9.153308	-3.5133	0.000948	0.034911	-0.85392
ILMN_2171295	0.127769	7.261982	3.508105	0.000963	0.035111	-0.86816
ILMN_1773002	-0.12775	7.412498	-3.40056	0.001329	0.042649	-1.1605
ILMN_1651799	-0.12774	11.70725	-3.60703	0.000714	0.029542	-0.59456
ILMN_1754727	-0.12763	6.925514	-4.21813	0.000103	0.008241	1.183775
ILMN_1747183	0.127496	6.988828	3.984996	0.000219	0.013631	0.488825
ILMN_1770911	0.127495	6.857643	4.33939	6.93E-05	0.006223	1.55233
ILMN_1697735	0.127458	9.890322	5.256236	3.04E-06	0.000792	4.460696
ILMN_1677402	-0.12735	6.360593	-3.34551	0.001563	0.0467	-1.30803
ILMN_1679195	0.127276	11.71869	4.131799	0.000137	0.010026	0.924271
ILMN_1741985	-0.12727	6.556949	-4.70614	2.03E-05	0.002732	2.692598
ILMN_1731354	0.127163	9.639258	4.179878	0.000117	0.008879	1.068496

ILMN_1802646	0.127105	5.629979	3.326603	0.001652	0.048147	-1.35834
ILMN_3233179	0.127101	7.616117	3.306308	0.001754	0.04984	-1.41216
ILMN_1750051	0.126996	6.800724	4.450784	4.79E-05	0.004816	1.89479
ILMN_1778255	0.126946	7.104808	3.749329	0.00046	0.021988	-0.19347
ILMN_1671568	-0.12678	8.992622	-3.64606	0.000633	0.027241	-0.48541
ILMN_1741942	0.126718	9.043092	3.47913	0.001051	0.036901	-0.94747
ILMN_1674421	0.126449	8.956569	3.702633	0.000532	0.024369	-0.32603
ILMN_1775522	0.126307	9.771732	3.339125	0.001593	0.047297	-1.32504
ILMN_1769158	0.126127	8.019062	3.637174	0.000651	0.0277	-0.51031
ILMN_2328378	-0.12612	7.923599	-3.30907	0.001739	0.049521	-1.40485
ILMN_1784352	-0.12607	8.794541	-3.97738	0.000225	0.013825	0.466443
ILMN_1687351	-0.12599	7.549386	-4.38386	5.98E-05	0.005651	1.688604
ILMN_1784540	-0.12578	8.812635	-3.45349	0.001135	0.038458	-1.0173
ILMN_1787280	0.125731	8.156233	3.817671	0.000372	0.019231	0.002154
ILMN_2363591	-0.12554	9.224569	-4.20682	0.000107	0.008446	1.149656
ILMN_2369221	-0.12552	5.999184	-3.50327	0.000978	0.035401	-0.88143
ILMN_1771966	0.125184	10.57052	3.479415	0.00105	0.036901	-0.94669
ILMN_1727001	0.124946	7.703579	3.795114	0.000399	0.020061	-0.06262
ILMN_1757914	0.124906	9.621177	3.327122	0.00165	0.048147	-1.35696
ILMN_2169761	-0.1249	7.763599	-3.44171	0.001176	0.039438	-1.04929
ILMN_2225061	0.124888	5.399499	4.842531	1.27E-05	0.001949	3.125284
ILMN_1733875	0.124827	7.811225	3.929631	0.000261	0.015308	0.326616
ILMN_1774589	0.124805	6.94714	3.755986	0.000451	0.021749	-0.1745
ILMN_1669268	-0.12477	7.286341	-3.51944	0.000931	0.034679	-0.83703
ILMN_2203896	-0.12468	7.439711	-3.5293	0.000904	0.033963	-0.8099
ILMN_1665095	0.124573	7.455196	3.779234	0.00042	0.020723	-0.1081
ILMN_1657631	-0.12449	6.994022	-4.51994	3.80E-05	0.004109	2.109171
ILMN_2182704	-0.12446	9.554422	-4.31169	7.59E-05	0.006596	1.467727
ILMN_1731165	0.12446	5.676588	3.369585	0.001456	0.044742	-1.24369
ILMN_1729281	0.12443	7.419073	4.537472	3.58E-05	0.003963	2.163721
ILMN_1703316	0.124408	7.538053	3.574074	0.000789	0.031452	-0.68619
ILMN_1795639	0.124236	9.320822	3.676455	0.000577	0.025468	-0.39995
ILMN_2047676	-0.12423	7.323166	-3.3661	0.001471	0.044915	-1.25303
ILMN_1694759	0.124112	7.99306	4.184746	0.000115	0.008798	1.08314
ILMN_1711823	0.124058	8.071418	3.757676	0.000449	0.021665	-0.16968
ILMN_1755664	0.123941	6.608051	5.236112	3.26E-06	0.000815	4.395042
ILMN_1673522	-0.12384	8.374233	-3.57427	0.000789	0.031452	-0.68565
ILMN_1733511	0.123742	9.79902	4.535455	3.61E-05	0.003969	2.157442
ILMN_1678966	0.123701	11.14048	3.364348	0.001479	0.044998	-1.25771
ILMN_1813657	0.123648	7.18278	4.023809	0.000194	0.012553	0.60321
ILMN_1779163	-0.12359	8.642296	-3.88734	0.000299	0.016537	0.203479
ILMN_1678054	-0.12351	7.81865	-3.37466	0.001434	0.044352	-1.23011
ILMN_1778488	-0.12336	7.39079	-4.53498	3.61E-05	0.003969	2.155951
ILMN_2167416	-0.12295	7.18592	-3.62304	0.00068	0.028507	-0.54985
ILMN_2367258	0.122871	5.760206	3.375038	0.001433	0.044346	-1.22908
ILMN_1728626	0.122818	8.320201	3.908833	0.000279	0.015832	0.265979
ILMN_1759595	0.122777	7.280271	5.690125	6.58E-07	0.000264	5.8897
ILMN_1740197	0.122742	6.46239	4.819811	1.38E-05	0.002038	3.052922
ILMN_1672843	-0.12274	8.3593	-4.0634	0.00017	0.011592	0.720448
ILMN_2219134	-0.1227	12.86068	-4.2054	0.000108	0.00845	1.145364
ILMN_1746426	0.122563	9.547126	4.438506	4.99E-05	0.004947	1.856869
ILMN_1743662	-0.12256	6.300212	-4.60933	2.82E-05	0.003382	2.388183
ILMN_1706779	0.122445	7.750245	3.382088	0.001403	0.043982	-1.21017
ILMN_1693004	0.122432	7.82199	3.322351	0.001673	0.048473	-1.36963

ILMN_1802338	-0.1224	6.528872	-4.90325	1.03E-05	0.001773	3.319217
ILMN_1661170	0.122363	11.5488	4.063984	0.00017	0.011592	0.722178
ILMN_1665300	-0.12235	7.015094	-5.46894	1.44E-06	0.000456	5.158272
ILMN_2063500	0.122297	7.628167	4.613823	2.77E-05	0.003342	2.402245
ILMN_1679483	0.122276	8.198779	3.945152	0.000249	0.014858	0.371974
ILMN_1757343	0.122275	10.7549	4.379265	6.07E-05	0.00571	1.674501
ILMN_1774196	0.122274	9.275008	3.926258	0.000264	0.015412	0.316772
ILMN_1674661	-0.12214	10.48293	-3.37518	0.001432	0.044346	-1.22869
ILMN_1778240	0.122038	8.455276	3.534691	0.000889	0.033576	-0.79507
ILMN_1663042	0.122013	9.702589	3.683455	0.000565	0.025005	-0.38021
ILMN_1670948	0.121991	8.812808	4.069337	0.000167	0.01144	0.738072
ILMN_1722089	-0.12197	7.619406	-3.68774	0.000557	0.02482	-0.36812
ILMN_2358914	0.121956	6.50809	4.187644	0.000114	0.008774	1.091863
ILMN_1704247	-0.12189	6.781117	-3.95792	0.000239	0.0144	0.409355
ILMN_1723467	-0.12185	11.41865	-3.99908	0.00021	0.013206	0.530273
ILMN_2340721	-0.12177	8.480932	-4.35288	6.63E-05	0.00603	1.593605
ILMN_1784227	0.121486	8.89894	4.863952	1.18E-05	0.001881	3.193614
ILMN_1805344	-0.12144	11.86283	-3.51803	0.000935	0.034774	-0.8409
ILMN_1823112	0.121208	6.26093	4.649588	2.46E-05	0.003069	2.514488
ILMN_1803773	0.121198	6.123491	4.453176	4.75E-05	0.004816	1.902183
ILMN_1801767	-0.12117	6.987505	-3.32824	0.001644	0.048047	-1.35399
ILMN_1711909	0.121098	7.403274	3.420775	0.001251	0.041004	-1.10598
ILMN_3240187	0.12094	9.69429	4.07302	0.000165	0.01135	0.749013
ILMN_1793743	-0.1209	8.542338	-4.04217	0.000183	0.01217	0.657517
ILMN_1686135	-0.12087	8.359989	-3.38268	0.001401	0.043982	-1.20858
ILMN_1655952	0.120472	9.16614	3.733743	0.000483	0.022857	-0.23782
ILMN_1801600	0.120299	8.147344	3.461924	0.001107	0.037926	-0.99437
ILMN_1708787	0.120235	6.52756	3.567809	0.000804	0.031909	-0.70356
ILMN_1755850	0.120167	5.583099	3.512599	0.00095	0.034911	-0.85583
ILMN_2374115	-0.12016	8.599269	-3.51413	0.000946	0.034911	-0.85164
ILMN_1706960	-0.12005	6.299473	-3.76875	0.000433	0.021229	-0.13808
ILMN_1783702	-0.11993	7.721322	-3.36173	0.00149	0.045292	-1.26471
ILMN_1768194	-0.11992	7.726417	-4.63798	2.56E-05	0.003136	2.478018
ILMN_1740430	0.119675	6.808733	4.413604	5.42E-05	0.005269	1.780086
ILMN_1665049	-0.11963	8.704278	-4.53736	3.59E-05	0.003963	2.16338
ILMN_1712944	-0.11962	9.416916	-3.82354	0.000365	0.019051	0.019052
ILMN_1734231	0.119445	9.752719	3.701724	0.000534	0.024369	-0.3286
ILMN_1815656	0.119427	8.904437	3.467802	0.001087	0.037641	-0.97836
ILMN_1754865	0.119325	9.424259	4.313832	7.54E-05	0.006581	1.474273
ILMN_2392080	-0.11932	7.594627	-3.60323	0.000722	0.029718	-0.60514
ILMN_1695797	0.119236	8.588752	3.496548	0.000998	0.03586	-0.89984
ILMN_1759991	0.11923	8.673025	4.438303	4.99E-05	0.004947	1.856242
ILMN_1745513	0.11922	7.305104	3.396457	0.001345	0.042812	-1.17156
ILMN_1707199	0.119147	7.796457	4.3319	7.10E-05	0.006329	1.529435
ILMN_1738103	0.119133	9.375674	3.375861	0.001429	0.044346	-1.22688
ILMN_1698015	0.118924	6.189212	4.514407	3.87E-05	0.004147	2.091968
ILMN_3261111	0.118895	6.0825	4.198557	0.00011	0.008582	1.124735
ILMN_1684591	0.118873	7.380275	4.005224	0.000205	0.012996	0.548372
ILMN_1692123	0.118778	6.494237	4.09672	0.000153	0.010842	0.819534
ILMN_1762439	-0.11863	7.006073	-3.75867	0.000447	0.021629	-0.16685
ILMN_3256325	0.118552	7.767774	3.36104	0.001493	0.045319	-1.26656
ILMN_1844692	-0.1182	8.90904	-3.34145	0.001582	0.047137	-1.31883
ILMN_1671619	0.117859	6.073866	4.441219	4.94E-05	0.004927	1.865243
ILMN_2405009	-0.11777	9.694206	-3.50819	0.000963	0.035111	-0.86793

ILMN_1799487	-0.11774	5.456221	-4.39653	5.74E-05	0.005479	1.727558
ILMN_1657129	-0.11769	8.061768	-3.72596	0.000495	0.023192	-0.25992
ILMN_3237209	0.117677	6.406315	4.673235	2.27E-05	0.00293	2.588875
ILMN_1681135	0.117592	6.153082	5.421089	1.71E-06	0.000522	5.000794
ILMN_1743806	-0.11757	7.173525	-3.57027	0.000798	0.031744	-0.69673
ILMN_1700733	-0.11753	8.020116	-3.83494	0.000352	0.018636	0.05187
ILMN_1720850	-0.11746	7.84599	-4.18015	0.000117	0.008879	1.069303
ILMN_1790461	-0.1173	10.46785	-3.99434	0.000213	0.01331	0.516323
ILMN_2389155	-0.11713	9.368498	-4.20725	0.000107	0.008446	1.150944
ILMN_1720838	-0.11711	10.69731	-4.52452	3.74E-05	0.004072	2.12341
ILMN_1671603	-0.11707	9.146976	-5.97435	2.39E-07	0.000125	6.836506
ILMN_2197247	0.116777	7.95462	4.181033	0.000116	0.008879	1.07197
ILMN_1702198	0.116701	7.340192	4.470886	4.48E-05	0.00461	1.956968
ILMN_2278819	-0.11652	6.91849	-4.11815	0.000143	0.010347	0.883455
ILMN_1808047	-0.1164	6.950668	-3.48362	0.001037	0.036654	-0.93521
ILMN_1705682	0.116254	5.980359	3.780066	0.000418	0.020699	-0.10572
ILMN_1688011	0.116192	7.523639	4.35094	6.67E-05	0.006053	1.58767
ILMN_2175114	-0.11611	5.816607	-3.64742	0.000631	0.027218	-0.48158
ILMN_3238269	0.116015	8.443412	4.164159	0.000123	0.009166	1.021257
ILMN_1737611	0.115991	6.66995	4.695003	2.11E-05	0.002794	2.657472
ILMN_1730077	0.115958	8.436897	4.598678	2.92E-05	0.003457	2.354813
ILMN_1781803	0.115932	6.925614	4.085971	0.000159	0.011033	0.787527
ILMN_1671932	0.11583	8.787435	4.198882	0.00011	0.008582	1.125713
ILMN_1813344	0.11573	8.433726	3.326378	0.001653	0.048147	-1.35894
ILMN_2053567	0.115699	7.403784	4.117346	0.000143	0.010352	0.881067
ILMN_3307786	0.115436	7.343689	4.093486	0.000155	0.010908	0.809901
ILMN_3225300	-0.11543	9.027645	-3.9325	0.000259	0.015239	0.335007
ILMN_2381121	0.115375	7.002543	3.833445	0.000354	0.018691	0.04757
ILMN_1722522	-0.11534	7.330681	-4.62744	2.65E-05	0.003215	2.444955
ILMN_1695311	-0.11528	6.497101	-3.64322	0.000639	0.027399	-0.49336
ILMN_1748057	-0.11523	6.078795	-4.21727	0.000104	0.008241	1.181188
ILMN_1791097	0.115081	7.561567	3.41466	0.001274	0.04148	-1.1225
ILMN_1683916	-0.11502	8.270671	-3.54254	0.000868	0.033115	-0.77343
ILMN_1710408	-0.11455	6.543896	-3.39498	0.001351	0.042894	-1.17553
ILMN_1819783	-0.11444	7.253281	-3.31477	0.001711	0.04894	-1.38973
ILMN_1768930	0.114359	10.04637	3.760925	0.000444	0.02153	-0.16041
ILMN_1654289	0.114309	8.02617	3.924092	0.000266	0.015492	0.310451
ILMN_2399264	0.114299	6.216862	4.113955	0.000145	0.010423	0.87094
ILMN_1781791	-0.11418	7.244427	-3.72503	0.000497	0.023197	-0.26258
ILMN_1737360	0.114132	5.933497	3.80403	0.000388	0.01978	-0.03704
ILMN_1809488	-0.11395	10.62056	-3.56376	0.000814	0.032056	-0.71477
ILMN_1729314	-0.11392	6.349821	-3.54129	0.000872	0.033133	-0.77686
ILMN_1692748	-0.1139	7.544309	-3.48949	0.001019	0.036364	-0.91917
ILMN_3249742	0.113839	6.037584	3.72818	0.000492	0.023093	-0.25362
ILMN_1669308	0.113745	7.241029	3.979611	0.000223	0.013776	0.472999
ILMN_1750636	0.113644	11.9311	3.359878	0.001498	0.045355	-1.26966
ILMN_2356955	-0.11327	6.470147	-3.31823	0.001693	0.048774	-1.38056
ILMN_2043109	0.113253	7.624979	4.264225	8.88E-05	0.007286	1.32334
ILMN_1659800	-0.11322	5.620496	-3.69026	0.000553	0.024798	-0.36101
ILMN_1806010	-0.11303	6.587764	-4.17866	0.000117	0.008895	1.064842
ILMN_1758339	0.113025	6.375826	3.350225	0.001542	0.046176	-1.29545
ILMN_1679324	0.113	9.648037	3.529087	0.000904	0.033963	-0.8105
ILMN_1658290	0.112868	7.569182	4.086318	0.000158	0.011033	0.788559
ILMN_1778242	0.11285	10.31384	3.483068	0.001039	0.036654	-0.93671

ILMN_1662161	0.112786	6.398688	4.035004	0.000187	0.012264	0.636302
ILMN_1693438	-0.11276	7.133441	-3.91728	0.000272	0.015534	0.290582
ILMN_1658677	0.11269	5.559248	3.803652	0.000389	0.01978	-0.03813
ILMN_1738420	0.11269	5.652472	4.185911	0.000115	0.008798	1.086646
ILMN_1657058	-0.11248	5.838526	-3.97251	0.000228	0.013966	0.452142
ILMN_1664025	0.112449	6.58128	3.455467	0.001128	0.038349	-1.01194
ILMN_1656194	-0.11224	5.805572	-4.36298	6.41E-05	0.005894	1.62456
ILMN_2397024	-0.11204	9.407902	-3.69263	0.000549	0.024798	-0.35431
ILMN_1675577	0.111843	7.180331	4.477396	4.38E-05	0.004538	1.977129
ILMN_1772677	0.111783	8.256293	4.271424	8.67E-05	0.007193	1.345196
ILMN_1811560	0.111641	8.083334	3.801635	0.000391	0.019839	-0.04392
ILMN_1812441	0.111605	8.001096	3.546789	0.000857	0.032877	-0.7617
ILMN_2198393	-0.11151	8.687471	-3.77515	0.000425	0.020898	-0.11977
ILMN_1671326	0.111497	6.730258	3.788575	0.000407	0.020302	-0.08136
ILMN_2360784	0.111433	9.320021	3.321264	0.001678	0.048546	-1.37252
ILMN_1798164	-0.1114	9.01414	-3.44927	0.001149	0.038809	-1.02878
ILMN_1683096	-0.11129	7.129923	-3.55769	0.000829	0.032309	-0.73158
ILMN_1779639	-0.11116	6.887925	-3.53881	0.000878	0.033234	-0.78372
ILMN_1676955	-0.11113	7.835196	-3.78111	0.000417	0.020661	-0.10274
ILMN_1790782	-0.11093	7.497588	-3.85222	0.000334	0.01801	0.101749
ILMN_1711102	-0.11092	7.192119	-3.75296	0.000455	0.021837	-0.18311
ILMN_2281186	-0.11091	5.81226	-4.4646	4.57E-05	0.00468	1.937528
ILMN_2352121	-0.11081	9.799415	-3.70529	0.000528	0.024295	-0.31851
ILMN_3251742	0.110777	7.084747	3.483081	0.001039	0.036654	-0.93668
ILMN_1691341	0.110749	6.484653	3.727812	0.000492	0.023093	-0.25467
ILMN_1687275	0.110584	8.405381	3.847352	0.000339	0.018174	0.087695
ILMN_2323633	0.110566	8.173019	3.895057	0.000292	0.016243	0.225905
ILMN_1803906	-0.11045	6.160431	-3.37502	0.001433	0.044346	-1.22913
ILMN_1725079	0.11042	7.7611	3.690842	0.000552	0.024798	-0.35936
ILMN_1669142	0.11004	7.797861	3.694115	0.000546	0.024785	-0.35011
ILMN_2094952	-0.11002	6.927402	-3.63515	0.000655	0.027737	-0.51597
ILMN_1806937	0.109829	8.86122	4.330333	7.14E-05	0.006341	1.524645
ILMN_2372011	0.1097	7.896012	3.3726	0.001443	0.044537	-1.23562
ILMN_1712859	-0.10944	6.573917	-4.08917	0.000157	0.010993	0.797047
ILMN_1661156	-0.10923	6.184562	-3.88361	0.000302	0.016653	0.192673
ILMN_1685022	0.109037	7.183875	3.334327	0.001615	0.047559	-1.33781
ILMN_1685286	0.108951	6.625015	3.782909	0.000415	0.020575	-0.09759
ILMN_1777528	0.108802	8.948298	3.996399	0.000211	0.013271	0.522373
ILMN_2242068	0.108732	6.378102	3.517001	0.000938	0.034825	-0.84374
ILMN_2181445	-0.10869	9.481545	-4.32975	7.15E-05	0.006341	1.522877
ILMN_1691112	-0.10861	7.70707	-3.68323	0.000565	0.025005	-0.38084
ILMN_1741736	-0.10856	8.045154	-3.84463	0.000342	0.018245	0.079826
ILMN_2352724	-0.10828	7.306223	-3.55057	0.000847	0.032718	-0.75126
ILMN_3261197	0.108269	10.5973	3.367275	0.001466	0.044915	-1.24988
ILMN_1726288	-0.10821	7.420287	-3.31397	0.001715	0.04894	-1.39185
ILMN_2058141	0.108209	11.87573	3.704362	0.000529	0.0243	-0.32114
ILMN_2144791	-0.10817	7.125175	-3.84095	0.000346	0.018403	0.069227
ILMN_2341467	0.108098	9.430058	3.773711	0.000427	0.020962	-0.1239
ILMN_1694504	0.108085	7.615096	3.590374	0.000751	0.030491	-0.64093
ILMN_1704500	-0.10804	6.769832	-3.56476	0.000812	0.032053	-0.712
ILMN_3205271	0.108002	11.52807	3.536005	0.000886	0.033479	-0.79144
ILMN_2124361	0.107929	6.170762	4.081002	0.000161	0.011128	0.772744
ILMN_1728224	0.107888	7.247527	3.320295	0.001683	0.048567	-1.37509
ILMN_2073010	0.107887	8.622636	3.416923	0.001266	0.041319	-1.11639

ILMN_1904135	0.107822	6.387132	3.634787	0.000656	0.027737	-0.517
ILMN_2346727	-0.10777	7.1026	-3.68242	0.000566	0.025036	-0.38314
ILMN_1755221	-0.10769	7.623086	-3.9651	0.000234	0.014174	0.430392
ILMN_1732053	0.107534	10.0834	3.339375	0.001592	0.047297	-1.32437
ILMN_1793267	0.107352	6.14238	3.35162	0.001535	0.046067	-1.29172
ILMN_1773066	-0.10693	7.448939	-3.39876	0.001336	0.042702	-1.16537
ILMN_1698166	-0.10678	7.116865	-4.02243	0.000194	0.012553	0.599142
ILMN_3244526	0.106686	7.229915	3.406511	0.001305	0.042165	-1.14448
ILMN_1770667	-0.10665	7.11012	-3.65104	0.000624	0.027053	-0.47144
ILMN_1761828	0.106447	8.61968	3.621738	0.000682	0.028587	-0.5535
ILMN_1702231	-0.10625	6.19729	-3.35878	0.001503	0.045462	-1.27261
ILMN_1867321	0.106126	6.243638	4.930747	9.41E-06	0.001673	3.407302
ILMN_2166686	0.105796	6.072654	4.007556	0.000204	0.012947	0.555243
ILMN_1717337	-0.10572	10.11213	-3.86899	0.000317	0.017273	0.150263
ILMN_1775753	0.10554	6.932964	3.567036	0.000806	0.03191	-0.7057
ILMN_1788095	0.105261	6.731435	3.722433	0.000501	0.023352	-0.26993
ILMN_1749345	-0.10518	9.090378	-3.41393	0.001277	0.041531	-1.12448
ILMN_1735402	0.105141	7.127156	4.278508	8.47E-05	0.007087	1.366719
ILMN_1701655	0.105136	7.201903	3.812669	0.000378	0.019476	-0.01223
ILMN_1721344	-0.10505	6.880078	-3.64175	0.000642	0.027488	-0.49747
ILMN_1713406	0.105018	8.336282	3.920207	0.000269	0.015534	0.299118
ILMN_1767766	0.104824	9.131113	3.807658	0.000384	0.019694	-0.02662
ILMN_1811178	-0.10471	7.144893	-3.63926	0.000647	0.027631	-0.50448
ILMN_2083567	0.104483	6.215335	3.334859	0.001613	0.047559	-1.33639
ILMN_1673172	0.104229	6.986877	3.637125	0.000651	0.0277	-0.51045
ILMN_2352190	0.104157	6.39338	3.688705	0.000556	0.024798	-0.36539
ILMN_2412761	-0.10414	6.307542	-3.92087	0.000269	0.015534	0.301062
ILMN_1715175	-0.10405	9.658292	-3.40015	0.00133	0.042661	-1.16162
ILMN_3309468	0.10385	6.141024	4.500567	4.06E-05	0.00429	2.04898
ILMN_1766195	-0.10384	7.173367	-3.717	0.000509	0.023652	-0.28534
ILMN_1738883	-0.10366	6.05132	-3.61945	0.000687	0.028753	-0.5599
ILMN_1732705	0.103659	9.413949	3.860027	0.000326	0.017656	0.12433
ILMN_2150284	0.103456	7.344529	4.288491	8.20E-05	0.006902	1.397077
ILMN_1764873	-0.10329	5.920363	-3.76566	0.000438	0.021343	-0.1469
ILMN_1658144	-0.10323	7.851685	-4.67203	2.28E-05	0.00293	2.585089
ILMN_1730307	-0.10317	6.961192	-3.35584	0.001516	0.045753	-1.28047
ILMN_3247906	0.103035	8.076963	3.43884	0.001186	0.03959	-1.05708
ILMN_1688526	-0.10265	11.46092	-3.33296	0.001622	0.047632	-1.34145
ILMN_3236239	-0.10261	6.713216	-3.33514	0.001612	0.047559	-1.33563
ILMN_1684755	0.102469	6.098881	3.712874	0.000516	0.023889	-0.29704
ILMN_2327276	0.102459	10.67604	4.109121	0.000147	0.010521	0.856514
ILMN_2336109	0.102059	7.715795	4.129083	0.000138	0.010087	0.916146
ILMN_1682354	0.101986	6.46123	4.919535	9.78E-06	0.001712	3.371369
ILMN_1789575	-0.10181	6.965427	-4.29027	8.15E-05	0.006902	1.402486
ILMN_1722583	0.101468	7.463456	3.504267	0.000975	0.035332	-0.87869
ILMN_1689720	-0.10139	6.837395	-3.81371	0.000377	0.019442	-0.00923
ILMN_1745421	-0.10136	7.696077	-3.40653	0.001305	0.042165	-1.14444
ILMN_1688160	-0.10136	6.519732	-3.96819	0.000231	0.01406	0.439474
ILMN_1688853	0.101327	7.418838	4.18768	0.000114	0.008774	1.091972
ILMN_1666376	0.101309	7.528633	3.316112	0.001704	0.04894	-1.38618
ILMN_2396786	-0.10131	7.295269	-4.01067	0.000202	0.012866	0.564434
ILMN_1660577	-0.10115	11.44089	-3.64364	0.000638	0.027398	-0.49218
ILMN_1660232	0.101072	9.078017	3.323601	0.001667	0.048418	-1.36631
ILMN_1773080	-0.10104	12.90952	-3.5396	0.000876	0.033207	-0.78153

ILMN_1714623	0.101015	9.620491	3.662811	0.000602	0.026324	-0.43836
ILMN_2349459	0.10088	8.499249	3.348595	0.001549	0.046318	-1.2998
ILMN_1722532	-0.10088	7.896159	-3.68538	0.000561	0.024936	-0.37478
ILMN_2370464	0.100737	7.256077	3.314346	0.001713	0.04894	-1.39087
ILMN_3249501	0.100615	6.204479	4.164066	0.000123	0.009166	1.02098
ILMN_3238560	-0.10055	8.406128	-3.33944	0.001591	0.047297	-1.32419
ILMN_1689578	-0.10031	6.207657	-3.55639	0.000833	0.0324	-0.73517
ILMN_1685275	0.100293	5.555373	3.904271	0.000283	0.015964	0.252698
ILMN_1782094	-0.10027	7.267839	-4.02281	0.000194	0.012553	0.600251
ILMN_1668426	-0.10004	6.007205	-4.09431	0.000154	0.010901	0.812354
ILMN_2095133	0.100021	8.352404	4.03933	0.000184	0.012201	0.649103
ILMN_1726107	0.099957	7.107832	4.702159	2.06E-05	0.002748	2.680049
ILMN_2357086	0.099948	5.587509	3.398261	0.001338	0.042702	-1.1667
ILMN_1818617	0.09988	6.776722	3.360539	0.001495	0.045325	-1.2679
ILMN_1753370	0.099844	6.248383	4.126648	0.000139	0.01013	0.908865
ILMN_1719988	0.099841	6.098033	3.562142	0.000818	0.032056	-0.71925
ILMN_1677452	0.099558	8.020636	3.546757	0.000857	0.032877	-0.76178
ILMN_1778032	0.099459	8.78322	3.594463	0.000742	0.030188	-0.62955
ILMN_3246801	0.099006	6.258492	4.436944	5.02E-05	0.004955	1.852047
ILMN_1753575	0.098891	6.485391	3.876786	0.000309	0.016928	0.172866
ILMN_1781672	-0.09881	6.603638	-3.392	0.001363	0.043142	-1.18355
ILMN_1672042	0.098724	7.272439	3.410424	0.00129	0.041926	-1.13393
ILMN_2380801	0.098702	6.182506	3.510175	0.000957	0.035078	-0.86249
ILMN_1691980	-0.09862	6.551937	-3.4983	0.000992	0.035741	-0.89503
ILMN_2344079	0.09857	6.244167	3.840913	0.000346	0.018403	0.069108
ILMN_1803819	-0.09856	9.694341	-4.33494	7.03E-05	0.006282	1.53871
ILMN_1798659	-0.09851	8.113755	-3.38289	0.0014	0.043982	-1.20802
ILMN_1694486	-0.09839	6.609586	-3.98166	0.000222	0.013735	0.479006
ILMN_1671516	-0.09837	7.946452	-3.76597	0.000437	0.021343	-0.14601
ILMN_1660844	-0.09829	8.236984	-3.57318	0.000791	0.031501	-0.68866
ILMN_1699987	-0.09814	6.630318	-3.58789	0.000757	0.030654	-0.64783
ILMN_2098947	0.097789	6.221145	3.98461	0.000219	0.013631	0.487689
ILMN_1760682	0.09777	6.726168	3.586475	0.00076	0.030714	-0.65176
ILMN_1729188	0.097728	6.18162	3.316423	0.001702	0.04894	-1.38536
ILMN_1713732	0.097719	7.408825	3.513201	0.000949	0.034911	-0.85418
ILMN_1687751	0.097687	6.294353	3.40336	0.001318	0.042415	-1.15297
ILMN_2367141	0.097646	6.138928	4.486616	4.25E-05	0.004454	2.005701
ILMN_3307648	0.0975	10.48895	4.152398	0.000128	0.009459	0.985967
ILMN_1811301	0.097492	7.229325	4.025661	0.000192	0.012526	0.60868
ILMN_1670377	0.097453	6.558711	3.575401	0.000786	0.031452	-0.68251
ILMN_3194087	-0.09724	6.69329	-3.39717	0.001342	0.042762	-1.16965
ILMN_1796464	-0.09707	6.732443	-3.97836	0.000224	0.013806	0.46933
ILMN_1730631	0.097025	7.288177	3.647719	0.00063	0.027218	-0.48075
ILMN_1750008	0.09699	7.387539	3.46873	0.001084	0.03761	-0.97583
ILMN_1721241	0.096955	6.310762	3.82963	0.000358	0.018776	0.036578
ILMN_1676745	0.09691	7.911071	4.256767	9.10E-05	0.007431	1.300714
ILMN_1690761	0.09686	8.819047	3.88623	0.0003	0.016569	0.200264
ILMN_2052163	-0.09682	9.130896	-3.44622	0.00116	0.03905	-1.03707
ILMN_1722981	-0.09665	6.391925	-3.48958	0.001019	0.036364	-0.9189
ILMN_1722648	0.096607	10.1849	3.55266	0.000842	0.032579	-0.74548
ILMN_1844905	-0.09641	6.367295	-3.56443	0.000813	0.032053	-0.71292
ILMN_2161556	0.096378	6.790024	4.515731	3.86E-05	0.004141	2.096081
ILMN_2049417	0.096302	5.960285	3.513685	0.000947	0.034911	-0.85285
ILMN_2156953	-0.09594	7.779871	-4.53731	3.59E-05	0.003963	2.163221

ILMN_1694385	0.095808	11.85941	3.913619	0.000275	0.015619	0.279917
ILMN_1674698	0.095779	7.212241	4.085547	0.000159	0.011033	0.786263
ILMN_1698940	0.095768	13.88241	3.498004	0.000993	0.035741	-0.89585
ILMN_1688515	0.09567	7.727903	3.595389	0.00074	0.030187	-0.62698
ILMN_1812281	0.09549	5.969044	3.69548	0.000544	0.024713	-0.34626
ILMN_1713450	0.095469	9.115566	3.437299	0.001191	0.039657	-1.06126
ILMN_1652412	-0.09542	6.524361	-3.71248	0.000516	0.023889	-0.29815
ILMN_1792518	-0.09535	8.309534	-3.55593	0.000834	0.032408	-0.73643
ILMN_1738369	0.095124	10.02442	4.042827	0.000182	0.01217	0.659455
ILMN_2222317	-0.09496	7.504694	-3.9435	0.00025	0.014894	0.36713
ILMN_1710652	0.094928	6.22486	3.381187	0.001407	0.044059	-1.21259
ILMN_2290068	-0.09481	5.847125	-4.13847	0.000134	0.009854	0.944225
ILMN_1735792	-0.09481	7.183724	-4.41332	5.43E-05	0.005269	1.779216
ILMN_1803564	-0.0948	8.155443	-3.63507	0.000655	0.027737	-0.51621
ILMN_2402805	0.094731	7.960192	3.565411	0.00081	0.032031	-0.7102
ILMN_1671374	0.09456	9.250392	3.531371	0.000898	0.03384	-0.80421
ILMN_1688070	0.094556	7.090268	3.431278	0.001213	0.040162	-1.07757
ILMN_1738589	0.094555	6.429344	3.446435	0.001159	0.03905	-1.03648
ILMN_1753980	-0.09452	7.043688	-4.49934	4.07E-05	0.004294	2.045159
ILMN_2058512	-0.09435	8.228484	-3.73553	0.000481	0.022762	-0.23273
ILMN_1698680	0.094204	6.402033	4.091813	0.000156	0.010944	0.804916
ILMN_1796900	-0.09402	7.310991	-3.77876	0.00042	0.020724	-0.10947
ILMN_2363586	-0.09381	10.02952	-4.03585	0.000186	0.012254	0.638803
ILMN_1701604	-0.09366	6.390781	-3.57887	0.000778	0.031213	-0.67289
ILMN_1684258	-0.09362	13.89482	-4.84762	1.25E-05	0.001927	3.141521
ILMN_2412214	-0.09358	5.800786	-3.87785	0.000308	0.016904	0.175947
ILMN_1692948	-0.09354	7.616537	-3.93696	0.000255	0.015076	0.348031
ILMN_1713936	-0.09346	6.452103	-3.82175	0.000367	0.019072	0.013894
ILMN_1769191	0.093225	12.14777	3.515665	0.000942	0.034873	-0.84741
ILMN_1709026	-0.09312	6.763506	-3.42673	0.001229	0.040554	-1.08988
ILMN_1727880	0.093119	5.588242	3.516194	0.00094	0.034855	-0.84596
ILMN_1663489	-0.09301	7.630736	-3.92091	0.000269	0.015534	0.301156
ILMN_1656706	0.092822	5.463017	4.34745	6.75E-05	0.006091	1.576987
ILMN_1795711	0.092655	5.933911	3.896968	0.00029	0.016171	0.231457
ILMN_2147435	-0.0926	8.722297	-3.69223	0.00055	0.024798	-0.35543
ILMN_1663772	0.092454	6.030705	3.654784	0.000617	0.026811	-0.46092
ILMN_1840934	0.092274	6.305919	3.423421	0.001241	0.040845	-1.09883
ILMN_1732049	0.092268	7.307442	3.3788	0.001417	0.044216	-1.219
ILMN_1698404	-0.09227	6.749597	-3.39032	0.00137	0.043317	-1.18805
ILMN_3240962	0.092263	6.771073	3.5193	0.000931	0.034679	-0.83742
ILMN_1682062	0.092234	6.578357	3.546765	0.000857	0.032877	-0.76176
ILMN_2389151	-0.0922	10.55855	-3.38591	0.001388	0.043766	-1.19992
ILMN_1800447	-0.09187	8.299848	-4.12091	0.000142	0.010277	0.891718
ILMN_1791884	0.091862	6.724049	3.776533	0.000423	0.020838	-0.11583
ILMN_3248057	0.091861	6.076667	4.950503	8.79E-06	0.001614	3.470677
ILMN_1681798	0.091777	6.133635	3.65202	0.000622	0.027005	-0.46868
ILMN_2296843	0.091553	7.075077	3.341793	0.00158	0.047131	-1.31793
ILMN_2225887	0.091535	12.82245	3.81848	0.000371	0.019211	0.004479
ILMN_1661409	0.091431	6.498988	3.794714	0.0004	0.020061	-0.06377
ILMN_2153485	-0.09108	6.056836	-4.26476	8.86E-05	0.007286	1.324969
ILMN_1704398	0.090493	5.50716	3.766217	0.000437	0.021343	-0.1453
ILMN_1809212	-0.09037	6.2086	-3.63104	0.000663	0.028014	-0.52748
ILMN_2274531	0.09031	6.273664	3.929466	0.000262	0.015308	0.326134
ILMN_1741391	-0.09025	7.728987	-3.82714	0.000361	0.018895	0.029415

ILMN_3262348	0.090213	6.846546	3.628027	0.000669	0.028179	-0.53591
ILMN_1767111	0.089716	6.715365	3.452807	0.001137	0.038458	-1.01917
ILMN_1666019	0.089581	6.638663	3.670931	0.000587	0.025871	-0.41551
ILMN_2342579	0.089501	5.564237	3.482111	0.001042	0.036685	-0.93932
ILMN_1652819	0.089493	6.465272	3.467778	0.001087	0.037641	-0.97843
ILMN_1665060	-0.08948	6.251033	-3.44563	0.001162	0.03908	-1.03865
ILMN_3235168	0.089228	8.770873	3.321528	0.001677	0.048546	-1.37182
ILMN_1741398	0.089212	6.951261	3.470842	0.001078	0.037448	-0.97008
ILMN_1771238	-0.08904	7.869939	-4.01338	0.0002	0.012779	0.572436
ILMN_2193498	-0.08886	6.518431	-3.61054	0.000706	0.029332	-0.58475
ILMN_1677305	0.088838	7.166174	3.452696	0.001138	0.038458	-1.01947
ILMN_1661307	0.088734	5.799211	3.905069	0.000283	0.015964	0.255021
ILMN_2098616	-0.08859	6.798537	-3.33455	0.001614	0.047559	-1.3372
ILMN_1771738	-0.08848	7.554359	-3.50964	0.000959	0.035098	-0.86396
ILMN_1789535	0.088435	6.954984	4.221496	0.000102	0.008185	1.193952
ILMN_3238402	0.088432	5.941097	3.704776	0.000529	0.0243	-0.31997
ILMN_1680703	0.08834	10.15744	4.640918	2.53E-05	0.003133	2.48725
ILMN_1781001	-0.0881	6.255321	-3.34503	0.001565	0.046724	-1.30929
ILMN_1682335	-0.0879	6.310612	-3.68856	0.000556	0.024798	-0.36582
ILMN_2111918	-0.0879	8.222654	-3.30673	0.001751	0.04982	-1.41104
ILMN_1789618	-0.08776	6.918948	-3.43868	0.001186	0.03959	-1.05751
ILMN_2089340	-0.08776	6.555807	-3.5417	0.000871	0.033133	-0.77575
ILMN_1812898	0.087652	5.747437	3.437522	0.00119	0.039657	-1.06065
ILMN_2110496	-0.08759	6.781827	-4.29689	7.97E-05	0.006787	1.422636
ILMN_2280731	-0.08733	6.640289	-3.64107	0.000643	0.027512	-0.4994
ILMN_1765060	-0.08709	8.299388	-3.39352	0.001357	0.043027	-1.17946
ILMN_1729167	-0.08709	7.546048	-4.40317	5.61E-05	0.005419	1.747976
ILMN_1675055	0.086687	7.704113	3.919883	0.00027	0.015534	0.298174
ILMN_1683044	-0.08666	8.296903	-3.32379	0.001666	0.048418	-1.36582
ILMN_1656145	0.086635	10.027	3.355723	0.001517	0.045753	-1.28077
ILMN_1688435	-0.08655	6.027814	-3.66478	0.000598	0.026232	-0.43283
ILMN_1792860	0.086297	6.301839	4.089169	0.000157	0.010993	0.797044
ILMN_1672122	0.086186	5.998331	3.425486	0.001234	0.040665	-1.09324
ILMN_1743643	0.085852	6.202437	3.547171	0.000856	0.032877	-0.76064
ILMN_1688534	0.08559	7.514029	3.420846	0.001251	0.041004	-1.10579
ILMN_3289631	-0.0849	5.915389	-3.64618	0.000633	0.027241	-0.48506
ILMN_1706553	0.084638	9.101854	3.479934	0.001049	0.036887	-0.94527
ILMN_1718042	0.084435	6.34898	3.437144	0.001192	0.039657	-1.06168
ILMN_2081682	0.084345	7.750483	3.799961	0.000393	0.01988	-0.04872
ILMN_1719763	0.084344	5.881417	4.115445	0.000144	0.010394	0.875391
ILMN_1725175	-0.08425	6.93202	-4.19078	0.000113	0.008743	1.101295
ILMN_1698715	-0.08416	6.978506	-3.65688	0.000613	0.026706	-0.45502
ILMN_1739241	-0.08378	5.446943	-3.41563	0.00127	0.041439	-1.11989
ILMN_1739454	-0.08364	7.317376	-4.36706	6.33E-05	0.005854	1.637055
ILMN_1685125	0.083364	6.304599	4.206249	0.000107	0.008446	1.147927
ILMN_1727361	0.083351	8.569409	3.523255	0.00092	0.034417	-0.82655
ILMN_1893764	0.083263	6.633854	3.45882	0.001117	0.038166	-1.00282
ILMN_1790354	-0.08288	10.19535	-3.39948	0.001333	0.042678	-1.16343
ILMN_1673944	0.082811	10.05075	3.386617	0.001385	0.043715	-1.19801
ILMN_1723846	0.082361	7.409379	3.558781	0.000827	0.032266	-0.72855
ILMN_1722742	-0.08234	6.338646	-3.33293	0.001622	0.047632	-1.34152
ILMN_1715546	0.082067	6.674725	3.374367	0.001436	0.044352	-1.23088
ILMN_1828216	0.081858	6.239727	3.471035	0.001077	0.037448	-0.96955
ILMN_1726025	0.081766	7.314399	3.608685	0.00071	0.029463	-0.58994

ILMN_1651642	0.081748	6.414432	3.383956	0.001396	0.0439	-1.20516
ILMN_2279217	-0.08169	6.027258	-4.25998	9.00E-05	0.007371	1.310469
ILMN_1700048	0.081616	5.846974	3.522609	0.000922	0.034446	-0.82833
ILMN_1742923	0.081258	6.171055	3.427659	0.001226	0.040519	-1.08736
ILMN_1806310	-0.08126	5.994995	-3.38214	0.001403	0.043982	-1.21004
ILMN_1706959	0.081203	8.660833	3.552337	0.000843	0.032579	-0.74637
ILMN_1726930	-0.08109	7.077231	-3.83218	0.000356	0.018691	0.043915
ILMN_2141157	-0.08103	6.212235	-4.31096	7.61E-05	0.006596	1.46551
ILMN_1793615	-0.08099	5.851298	-3.46684	0.001091	0.037673	-0.98099
ILMN_1715181	0.080834	6.690597	3.483711	0.001037	0.036654	-0.93495
ILMN_1678627	0.080782	5.3752	4.266372	8.81E-05	0.007286	1.329856
ILMN_1692896	0.080746	6.625689	3.568255	0.000803	0.031902	-0.70232
ILMN_1735415	0.080736	7.315729	3.314233	0.001713	0.04894	-1.39117
ILMN_3297317	0.080418	13.90607	3.783083	0.000415	0.020575	-0.09709
ILMN_3245143	-0.08022	5.815494	-3.80461	0.000388	0.01978	-0.03537
ILMN_1776352	-0.08009	7.781603	-3.44382	0.001168	0.039253	-1.04356
ILMN_1774281	-0.08001	6.209842	-3.33091	0.001632	0.047848	-1.34688
ILMN_2197128	0.079973	6.431742	3.330231	0.001635	0.047848	-1.3487
ILMN_2177460	0.079857	6.285313	3.316432	0.001702	0.04894	-1.38534
ILMN_1769503	-0.07962	5.950628	-3.33759	0.0016	0.047389	-1.32912
ILMN_1698725	0.079448	6.157744	3.917272	0.000272	0.015534	0.290564
ILMN_3245893	-0.0793	6.436412	-3.46023	0.001112	0.038043	-0.99897
ILMN_1662334	0.079128	9.877035	4.099661	0.000152	0.010781	0.828299
ILMN_2366714	-0.07908	7.945501	-3.51268	0.00095	0.034911	-0.8556
ILMN_2049642	-0.07889	10.4411	-3.91907	0.00027	0.015534	0.2958
ILMN_1718898	0.078805	6.109315	3.590075	0.000752	0.030491	-0.64176
ILMN_1773716	0.078737	7.94473	3.423365	0.001242	0.040845	-1.09898
ILMN_2134224	0.078722	6.828101	3.848606	0.000338	0.018131	0.091317
ILMN_1782730	0.078428	6.297205	4.27458	8.58E-05	0.007145	1.354782
ILMN_1692486	0.078346	8.511366	3.801548	0.000391	0.019839	-0.04417
ILMN_1722771	-0.07816	5.778356	-3.54389	0.000865	0.033089	-0.76969
ILMN_3268590	0.077823	5.775323	3.516837	0.000938	0.034825	-0.84419
ILMN_1721628	0.077529	5.548135	3.353768	0.001526	0.045896	-1.28599
ILMN_2388965	0.077408	5.253054	4.08695	0.000158	0.011033	0.790439
ILMN_2124425	0.077408	5.376862	3.615942	0.000695	0.028957	-0.56968
ILMN_1676159	-0.07724	9.37641	-3.31593	0.001705	0.04894	-1.38667
ILMN_2390586	-0.07637	7.047253	-3.81925	0.00037	0.019194	0.006685
ILMN_1689029	-0.07601	6.564634	-3.43452	0.001201	0.03989	-1.06878
ILMN_1809094	-0.07597	6.7153	-3.46461	0.001098	0.037789	-0.98707
ILMN_1713454	0.07573	6.15932	3.432302	0.001209	0.040078	-1.07479
ILMN_1803094	0.075213	6.214764	3.5056	0.000971	0.035265	-0.87504
ILMN_1745668	-0.07454	5.787069	-3.78351	0.000414	0.020575	-0.09587
ILMN_1696826	0.074385	5.909971	4.184926	0.000115	0.008798	1.083682
ILMN_1737818	0.074303	7.595981	3.330799	0.001632	0.047848	-1.34719
ILMN_1806782	0.074235	6.27442	3.377609	0.001422	0.044289	-1.22219
ILMN_1705330	-0.0737	6.392187	-3.34973	0.001544	0.046203	-1.29675
ILMN_1761181	0.073293	5.857509	3.562204	0.000818	0.032056	-0.71908
ILMN_1792242	0.073257	5.673458	3.315421	0.001707	0.04894	-1.38802
ILMN_1712751	0.073132	7.449822	3.354747	0.001521	0.045804	-1.28337
ILMN_1801395	-0.07282	5.985178	-3.33039	0.001634	0.047848	-1.34828
ILMN_1699836	0.072332	5.569219	3.441598	0.001176	0.039438	-1.0496
ILMN_1715654	0.072229	6.159278	3.493423	0.001007	0.036049	-0.9084
ILMN_1748607	-0.07213	5.781067	-3.52367	0.000919	0.03441	-0.82539
ILMN_1713247	-0.07186	5.57309	-3.48722	0.001026	0.036495	-0.92537

ILMN_1689651	0.07114	5.329057	3.727953	0.000492	0.023093	-0.25427
ILMN_1799669	-0.07091	6.169469	-3.60346	0.000722	0.029718	-0.60451
ILMN_1676594	-0.07084	5.782597	-3.51472	0.000944	0.034911	-0.85001
ILMN_1789955	-0.07046	5.498019	-3.40629	0.001306	0.042165	-1.14506
ILMN_2410905	-0.07006	5.267522	-3.38672	0.001384	0.043715	-1.19773
ILMN_1696767	0.069905	5.26613	3.404329	0.001314	0.042371	-1.15036
ILMN_1815570	0.069839	6.374613	3.476571	0.001059	0.037034	-0.95445
ILMN_1767441	0.069765	6.13581	3.68844	0.000556	0.024798	-0.36614
ILMN_2209766	-0.06973	6.632917	-3.48457	0.001034	0.036639	-0.9326
ILMN_3300358	0.069564	6.250778	3.482501	0.00104	0.036679	-0.93826
ILMN_1728802	-0.06932	5.97848	-3.48819	0.001023	0.036431	-0.92272
ILMN_1887128	-0.06927	6.128285	-3.8509	0.000335	0.018029	0.097944
ILMN_1759312	-0.06919	5.296898	-3.40259	0.001321	0.042432	-1.15504
ILMN_2085446	0.06817	6.516636	3.3381	0.001598	0.047389	-1.32776
ILMN_1661002	-0.06753	9.235879	-3.52587	0.000913	0.034221	-0.81936
ILMN_1651498	0.066353	5.48707	3.320379	0.001683	0.048567	-1.37487
ILMN_3225014	0.066123	5.747072	3.42179	0.001247	0.040958	-1.10324
ILMN_1885908	-0.06612	5.936269	-3.58262	0.000769	0.031004	-0.66247
ILMN_1761844	0.066018	8.75912	3.485764	0.00103	0.036546	-0.92934
ILMN_1693319	-0.06332	5.331141	-3.46317	0.001103	0.037822	-0.99097
ILMN_2338849	-0.06286	5.81064	-3.59854	0.000733	0.030029	-0.61822
ILMN_1759801	0.062286	7.855688	3.456994	0.001123	0.038324	-1.00778
ILMN_2038773	-0.06124	14.04246	-3.33314	0.001621	0.047632	-1.34095
ILMN_2084912	-0.05984	5.261935	-3.4399	0.001182	0.03959	-1.05419
ILMN_3211262	0.059778	5.588309	3.351735	0.001535	0.046067	-1.29142
ILMN_1673042	0.059436	6.00835	3.618257	0.00069	0.028788	-0.56322
ILMN_2232084	-0.05891	6.062851	-3.64592	0.000634	0.027241	-0.48578
ILMN_2331501	-0.0583	14.14271	-3.45588	0.001127	0.038349	-1.01083
ILMN_1736234	0.055856	8.677606	3.471953	0.001074	0.037399	-0.96705

Supplementary Table S2. Genes showing significant concentration-dependent associations with levels of HPV16 E7 protein in the W12 clones.

	logFC	AveExpr	t	P.Value	adj.P.Val	B
ILMN_2133205	-0.69082	7.992169	-5.43423	1.63E-06	0.00066	5.034165
ILMN_1739513	0.604786	7.219708	8.736298	1.22E-11	4.21E-07	16.21005
ILMN_1744604	-0.58674	7.452409	-6.36858	5.80E-08	6.22E-05	8.197195
ILMN_2391861	-0.58146	8.151042	-4.66289	2.35E-05	0.003	2.51143
ILMN_1725139	0.565743	6.326501	7.513792	9.38E-10	6.47E-06	12.10954
ILMN_1773006	-0.55724	10.33058	-3.28438	0.001869	0.044572	-1.56481
ILMN_1705750	-0.44125	9.719861	-5.36966	2.04E-06	0.000741	4.818896
ILMN_1668134	-0.42785	6.914501	-3.33398	0.001617	0.040663	-1.43198
ILMN_1788874	0.411575	7.706951	4.320598	7.37E-05	0.006356	1.436105
ILMN_1658494	-0.40806	8.771834	-4.16641	0.000122	0.008526	0.963249
ILMN_1705080	-0.40105	8.187943	-4.27217	8.65E-05	0.006983	1.286749
ILMN_1772627	-0.38929	8.074501	-4.80845	1.43E-05	0.00235	2.978126
ILMN_1762255	-0.38743	7.106344	-3.23094	0.002183	0.049513	-1.70653
ILMN_1812403	-0.37152	9.949197	-4.95218	8.74E-06	0.001684	3.44371
ILMN_1682015	0.370871	8.023019	3.887879	0.000298	0.015374	0.130089
ILMN_1673639	-0.36746	7.225538	-4.70704	2.02E-05	0.002835	2.652426
ILMN_1671478	-0.36556	8.875751	-5.90923	3.01E-07	0.000206	6.633196
ILMN_2072178	0.33632	7.401153	4.915263	9.93E-06	0.001858	3.323705
ILMN_1680110	-0.33061	10.90965	-3.89325	0.000293	0.015188	0.145878
ILMN_1775814	-0.33047	8.011236	-3.61178	0.000703	0.024698	-0.66571
ILMN_1806754	0.328635	8.880934	3.417994	0.001262	0.034602	-1.20413
ILMN_1722670	-0.32843	7.524326	-3.53337	0.000893	0.028185	-0.88569
ILMN_3249032	0.323525	7.755069	4.622503	2.69E-05	0.00328	2.382873
ILMN_1699989	-0.3222	8.280026	-4.83682	1.30E-05	0.002206	3.069658
ILMN_1754241	0.320601	7.183669	3.920537	0.000269	0.014425	0.226278
ILMN_1705107	-0.30974	8.0672	-3.82376	0.000365	0.017052	-0.05756
ILMN_1790227	-0.30943	8.273254	-3.76294	0.000441	0.018974	-0.23398
ILMN_1693338	0.303947	8.093769	3.309336	0.001738	0.042499	-1.49813
ILMN_1766054	-0.30186	7.521938	-5.00198	7.36E-06	0.001515	3.606065
ILMN_1744765	-0.30137	11.11599	-3.4151	0.001273	0.03479	-1.21205
ILMN_2242463	0.29533	10.18413	5.304838	2.57E-06	0.00082	4.603435
ILMN_2096372	-0.2941	6.843431	-3.44507	0.001164	0.033106	-1.12997
ILMN_1690017	-0.29005	8.585352	-3.29798	0.001797	0.043525	-1.52852
ILMN_1735045	-0.28713	7.475736	-5.58536	9.54E-07	0.000439	5.540149
ILMN_1805665	-0.28221	9.899024	-4.77299	1.62E-05	0.002454	2.863952
ILMN_1737561	-0.28016	7.754604	-4.79629	1.49E-05	0.00235	2.93895
ILMN_2138765	0.279582	7.772094	6.150264	1.27E-07	0.000102	7.452398
ILMN_1666893	0.278934	6.466164	8.28675	5.95E-11	1.03E-06	14.7173
ILMN_2390919	-0.26961	7.823265	-4.84969	1.24E-05	0.002198	3.11126
ILMN_2304512	0.263785	8.426435	3.512194	0.000952	0.029398	-0.9446
ILMN_1713829	-0.26237	9.390348	-4.03236	0.000188	0.011595	0.558717
ILMN_2376723	-0.2572	9.075359	-5.29087	2.69E-06	0.00084	4.557095
ILMN_1651496	-0.25572	9.321274	-3.47469	0.001065	0.031169	-1.04844
ILMN_1696347	0.254268	9.758519	4.311322	7.60E-05	0.00644	1.407441
ILMN_1706015	-0.25198	7.100224	-3.33518	0.001611	0.0406	-1.42874
ILMN_1711087	-0.24569	8.94067	-5.58842	9.44E-07	0.000439	5.550431
ILMN_1774602	-0.24503	8.368134	-4.83402	1.31E-05	0.002206	3.060615
ILMN_1808590	0.244658	7.188088	3.810764	0.00038	0.017435	-0.09537
ILMN_1773337	-0.24373	11.82517	-4.07271	0.000165	0.010504	0.679826
ILMN_1789400	-0.23944	7.093376	-3.69495	0.000545	0.021487	-0.4294

ILMN_2214197	-0.23937	7.404584	-5.38227	1.95E-06	0.000724	4.860907
ILMN_1660067	0.239114	7.137712	4.83418	1.31E-05	0.002206	3.061129
ILMN_1667966	-0.23757	7.760319	-5.39507	1.87E-06	0.000716	4.903541
ILMN_1760990	-0.23727	6.710187	-5.28976	2.70E-06	0.00084	4.553421
ILMN_1776121	-0.23717	7.409774	-3.69834	0.000539	0.021346	-0.41968
ILMN_1798006	-0.23447	7.928236	-3.73458	0.000482	0.019833	-0.31574
ILMN_3244348	0.232543	8.046509	6.477717	3.92E-08	5.36E-05	8.570171
ILMN_1723123	-0.23142	10.92282	-5.38191	1.96E-06	0.000724	4.859691
ILMN_1751086	0.230311	9.493877	5.759145	5.15E-07	0.000306	6.125371
ILMN_3227023	-0.23002	9.465506	-5.6941	6.49E-07	0.000367	5.905962
ILMN_3234089	-0.22814	7.081691	-4.22294	0.000102	0.00739	1.135726
ILMN_1661599	-0.22804	11.56727	-4.24551	9.44E-05	0.007245	1.204875
ILMN_1743103	-0.2251	10.09195	-4.85508	1.22E-05	0.002169	3.128682
ILMN_3242586	-0.2251	6.789435	-3.67337	0.000582	0.022311	-0.49099
ILMN_1800130	-0.22448	7.260117	-4.68204	2.20E-05	0.002875	2.572544
ILMN_1799098	-0.22444	10.47357	-6.66432	2.00E-08	3.45E-05	9.208465
ILMN_3243156	-0.22431	10.01686	-5.94739	2.63E-07	0.000193	6.762596
ILMN_1800317	-0.2235	6.100468	-4.73142	1.86E-05	0.002686	2.730501
ILMN_3305614	-0.22337	6.399203	-3.95793	0.000239	0.013337	0.336928
ILMN_1695945	-0.2213	6.886069	-6.48123	3.87E-08	5.36E-05	8.582193
ILMN_1790100	0.219813	8.226173	5.730934	5.69E-07	0.000327	6.030154
ILMN_2160209	0.219491	6.731097	4.859042	1.20E-05	0.002151	3.141487
ILMN_1699651	0.21889	6.608253	3.239464	0.00213	0.048563	-1.68402
ILMN_1794742	-0.21841	8.365627	-4.45683	4.69E-05	0.004731	1.860089
ILMN_1700583	0.218227	9.328275	3.818623	0.000371	0.017189	-0.07251
ILMN_1721876	-0.21636	8.700419	-5.12289	4.84E-06	0.001192	4.002272
ILMN_1714108	-0.21597	7.661412	-5.62776	8.21E-07	0.000399	5.682634
ILMN_1777233	0.215541	8.912187	6.414197	4.92E-08	5.66E-05	8.353047
ILMN_1856480	-0.21465	7.171697	-3.95397	0.000242	0.01341	0.32518
ILMN_1749096	0.212726	6.532685	3.97445	0.000227	0.013051	0.385978
ILMN_1708934	-0.21218	11.66936	-4.5563	3.37E-05	0.00377	2.173058
ILMN_1693471	0.21188	8.402875	3.659053	0.000609	0.022886	-0.53176
ILMN_1781285	-0.20738	10.96082	-3.97081	0.000229	0.013128	0.375172
ILMN_1711069	-0.20737	9.757086	-4.36535	6.36E-05	0.005842	1.574779
ILMN_2095610	-0.20575	9.971736	-6.18361	1.13E-07	9.26E-05	7.566012
ILMN_1810725	-0.20455	7.339041	-4.12605	0.000139	0.009322	0.840803
ILMN_1799105	-0.20401	9.237549	-5.03218	6.63E-06	0.001469	3.704784
ILMN_2186983	-0.20334	10.29542	-5.87973	3.35E-07	0.000218	6.533227
ILMN_1678655	-0.20262	9.884598	-3.29778	0.001798	0.043525	-1.52904
ILMN_3224926	0.20202	8.871704	4.83587	1.30E-05	0.002206	3.066589
ILMN_3242004	-0.20153	9.102102	-5.68	6.82E-07	0.000373	5.858434
ILMN_3236858	-0.20108	6.435354	-3.82872	0.000359	0.01695	-0.04308
ILMN_1739450	-0.20096	9.157353	-6.31927	6.93E-08	6.64E-05	8.028776
ILMN_1788955	-0.20086	10.90429	-5.13789	4.60E-06	0.001173	4.051633
ILMN_1772910	-0.19989	8.398031	-4.43332	5.08E-05	0.004903	1.786511
ILMN_3306730	0.198534	8.639006	4.871314	1.15E-05	0.002095	3.181203
ILMN_1722834	-0.19831	8.939424	-4.69948	2.08E-05	0.002859	2.628261
ILMN_1759513	0.198056	10.91744	3.254032	0.002042	0.047308	-1.64547
ILMN_1675616	0.197984	6.875555	4.674844	2.26E-05	0.002935	2.549559
ILMN_1751161	-0.19614	10.99825	-4.41417	5.41E-05	0.00517	1.726716
ILMN_1770085	-0.19605	8.319791	-3.26564	0.001974	0.046396	-1.61468
ILMN_1657766	-0.19599	10.09657	-4.69623	2.10E-05	0.002859	2.617875
ILMN_1691476	-0.1953	7.248167	-3.41374	0.001278	0.034875	-1.21575
ILMN_1793017	-0.19524	7.235809	-6.33936	6.45E-08	6.35E-05	8.097384

ILMN_1696270	-0.1952	8.109972	-6.44449	4.42E-08	5.49E-05	8.456579
ILMN_2413816	0.194739	6.652781	4.318761	7.42E-05	0.006379	1.430427
ILMN_1705213	-0.19343	8.975996	-6.43922	4.50E-08	5.49E-05	8.438583
ILMN_1749109	-0.19245	11.15245	-6.46916	4.04E-08	5.36E-05	8.54091
ILMN_1790962	0.191401	6.6506	4.07715	0.000163	0.010374	0.693189
ILMN_1663080	-0.19126	8.308611	-5.00278	7.34E-06	0.001515	3.60869
ILMN_1791580	-0.19116	7.516767	-4.13949	0.000133	0.009067	0.881531
ILMN_1792679	0.190885	7.392167	5.616248	8.55E-07	0.00041	5.643927
ILMN_1672908	-0.19003	6.931672	-3.88584	0.0003	0.015385	0.124108
ILMN_1788192	-0.18993	6.735082	-4.17144	0.00012	0.008436	0.97857
ILMN_1729217	-0.18965	6.33715	-3.90235	0.000285	0.014932	0.172648
ILMN_1773959	0.189189	5.740913	7.359984	1.63E-09	9.37E-06	11.58599
ILMN_1731640	-0.18888	8.258374	-3.58377	0.000766	0.025767	-0.74462
ILMN_1686555	-0.18878	6.820999	-5.60347	8.95E-07	0.000423	5.600991
ILMN_1799106	0.188677	7.097389	5.035171	6.56E-06	0.001469	3.714553
ILMN_1685540	0.188063	7.832534	6.992856	6.12E-09	1.81E-05	10.3325
ILMN_1775114	-0.188	7.008211	-4.75929	1.69E-05	0.002506	2.819925
ILMN_1771841	0.187995	7.982244	3.41137	0.001287	0.035066	-1.22222
ILMN_2355559	-0.18768	11.20146	-5.51953	1.20E-06	0.000513	5.319404
ILMN_1678669	0.186617	7.8982	4.43909	4.98E-05	0.004864	1.804564
ILMN_1791912	-0.18606	8.240175	-5.52796	1.17E-06	0.000513	5.347627
ILMN_2365686	0.185661	9.254504	4.583386	3.07E-05	0.003615	2.258766
ILMN_1775883	-0.18527	6.818445	-3.78039	0.000418	0.018383	-0.1835
ILMN_1741755	-0.1847	9.953249	-5.34893	2.20E-06	0.000773	4.749915
ILMN_1709307	-0.18441	7.171161	-3.70652	0.000526	0.020981	-0.39628
ILMN_1651282	-0.18413	11.50699	-4.17091	0.00012	0.008436	0.97696
ILMN_2180371	0.183056	7.825553	4.364052	6.39E-05	0.005842	1.570734
ILMN_1778360	-0.18193	10.85475	-4.37546	6.15E-05	0.005671	1.606189
ILMN_1676361	-0.18146	7.368993	-5.37935	1.97E-06	0.000724	4.85116
ILMN_3248857	0.180974	6.346806	6.936005	7.51E-09	1.85E-05	10.13807
ILMN_1692938	0.180921	8.625168	5.423327	1.69E-06	0.000678	4.997764
ILMN_1669433	-0.1808	8.764554	-5.3225	2.41E-06	0.000807	4.662078
ILMN_1787265	-0.17995	8.776492	-4.12445	0.00014	0.009352	0.835972
ILMN_1886515	-0.17976	8.070173	-4.52877	3.69E-05	0.003964	2.086157
ILMN_2342437	0.179462	7.164944	5.106722	5.12E-06	0.001243	3.949136
ILMN_2330307	0.179246	7.789457	4.218577	0.000103	0.007459	1.122367
ILMN_1657111	-0.17843	10.45706	-4.69733	2.09E-05	0.002859	2.621369
ILMN_2125346	-0.17823	6.785924	-3.74013	0.000474	0.019682	-0.29977
ILMN_3251691	0.177897	7.470628	4.456043	4.71E-05	0.004731	1.857622
ILMN_1711994	0.177382	7.334314	5.234759	3.28E-06	0.000942	4.371233
ILMN_3233388	0.176453	8.969333	6.739198	1.53E-08	3.00E-05	9.464694
ILMN_2390974	-0.17632	9.158101	-5.10866	5.09E-06	0.001243	3.955506
ILMN_1704537	0.174733	9.658634	3.558423	0.000828	0.026893	-0.8157
ILMN_2376050	-0.17442	8.043913	-3.95766	0.000239	0.013337	0.336136
ILMN_1692026	0.17425	7.547477	7.133646	3.68E-09	1.59E-05	10.8137
ILMN_1671365	-0.17389	8.105262	-3.51116	0.000955	0.029437	-0.94748
ILMN_1749118	0.17385	6.735765	3.493218	0.001008	0.030417	-0.99722
ILMN_1680018	-0.17329	8.648107	-3.8779	0.000308	0.015586	0.100767
ILMN_1724139	0.172771	11.04195	5.130282	4.72E-06	0.001179	4.026586
ILMN_2326712	0.17267	7.108977	4.557986	3.35E-05	0.00377	2.178398
ILMN_3241441	-0.17242	6.992044	-4.47995	4.35E-05	0.004446	1.932592
ILMN_1809695	-0.17203	8.243369	-4.09578	0.000154	0.010009	0.749326
ILMN_1698732	0.171964	9.841955	4.398668	5.70E-05	0.005349	1.678395
ILMN_1664861	-0.17179	10.51662	-4.25603	9.12E-05	0.007161	1.237156

ILMN_3201453	0.17156	9.408963	4.976452	8.04E-06	0.001575	3.522787
ILMN_2131177	0.170944	6.861966	3.579755	0.000776	0.025861	-0.75589
ILMN_1690289	-0.17082	8.508461	-4.13944	0.000133	0.009067	0.881354
ILMN_1811195	-0.17078	6.387587	-6.00066	2.17E-07	0.000163	6.943471
ILMN_1732609	-0.17036	7.328232	-4.30722	7.71E-05	0.00648	1.394789
ILMN_1903021	-0.17026	6.660383	-4.92803	9.50E-06	0.00181	3.365175
ILMN_1754842	-0.17009	7.573756	-5.85803	3.62E-07	0.000231	6.459745
ILMN_1723412	-0.16989	8.099925	-4.09827	0.000152	0.009966	0.756831
ILMN_1737283	-0.16984	8.270082	-3.43375	0.001204	0.033496	-1.16102
ILMN_1797822	-0.16982	7.703849	-3.59817	0.000733	0.025232	-0.7041
ILMN_1788931	-0.16947	6.978091	-3.7668	0.000436	0.018842	-0.22282
ILMN_2087941	-0.16922	7.976439	-4.33884	6.94E-05	0.00617	1.492556
ILMN_1838942	0.168967	7.333424	3.626232	0.000673	0.024223	-0.62487
ILMN_2151281	-0.1688	7.544569	-3.64088	0.000644	0.023685	-0.58337
ILMN_1673522	0.168706	8.374233	6.962167	6.83E-09	1.81E-05	10.22755
ILMN_1685413	0.168322	9.058596	5.340976	2.26E-06	0.000787	4.723477
ILMN_1700310	-0.16806	6.956644	-3.32055	0.001682	0.04148	-1.46807
ILMN_1800091	0.167428	6.191857	6.115847	1.44E-07	0.000113	7.335192
ILMN_1865764	-0.16725	7.97774	-5.24706	3.14E-06	0.000933	4.411935
ILMN_1789775	0.167218	8.644515	4.804303	1.45E-05	0.00235	2.96475
ILMN_1801077	0.167033	6.694603	6.73163	1.57E-08	3.00E-05	9.438796
ILMN_1673543	0.16702	8.339837	6.300342	7.42E-08	6.91E-05	7.964174
ILMN_3305273	0.166488	7.434981	6.485871	3.80E-08	5.36E-05	8.59805
ILMN_2176502	-0.1662	6.956946	-4.09827	0.000152	0.009966	0.756842
ILMN_2404065	-0.16515	9.856242	-6.21103	1.02E-07	8.81E-05	7.659478
ILMN_2219466	0.16488	6.618042	4.044641	0.000181	0.011207	0.595524
ILMN_1706051	-0.16481	6.646429	-3.40728	0.001302	0.03535	-1.23338
ILMN_1673673	0.164412	7.439336	4.492771	4.16E-05	0.004324	1.972855
ILMN_2217935	0.164017	8.484241	6.213782	1.01E-07	8.81E-05	7.668869
ILMN_1738955	-0.1635	8.396592	-5.78486	4.70E-07	0.000284	6.212235
ILMN_1682402	0.163494	6.554808	7.152685	3.44E-09	1.59E-05	10.87874
ILMN_1679093	-0.16337	9.146616	-4.28094	8.40E-05	0.006881	1.313749
ILMN_3200421	0.163289	7.335807	5.135903	4.63E-06	0.001173	4.045081
ILMN_1781400	-0.1632	6.351035	-3.34121	0.001583	0.040178	-1.4125
ILMN_2209578	-0.16293	8.965183	-6.48365	3.83E-08	5.36E-05	8.590467
ILMN_3238707	0.162413	7.42062	5.662761	7.25E-07	0.000373	5.800397
ILMN_2158705	-0.16184	7.68826	-4.01645	0.000198	0.012009	0.511149
ILMN_2128967	-0.16154	9.231968	-3.88292	0.000303	0.015432	0.115507
ILMN_1669888	-0.16147	9.58316	-3.92999	0.000261	0.014176	0.254189
ILMN_1769451	0.16129	8.506642	6.994714	6.08E-09	1.81E-05	10.33885
ILMN_2401878	0.161226	8.20921	4.839888	1.29E-05	0.002206	3.079567
ILMN_3242883	-0.16121	8.583161	-5.66718	7.14E-07	0.000373	5.815265
ILMN_1781386	-0.1611	7.357152	-3.75741	0.000449	0.019184	-0.24994
ILMN_1683450	0.160863	9.802312	5.193307	3.79E-06	0.001045	4.234263
ILMN_2134039	0.159434	9.220499	3.585713	0.000762	0.025703	-0.73915
ILMN_1658426	-0.15894	6.566176	-3.84897	0.000337	0.016315	0.016023
ILMN_1707350	-0.1589	7.336674	-4.25202	9.24E-05	0.007221	1.224839
ILMN_1756541	-0.1586	8.567317	-5.03776	6.51E-06	0.001469	3.723021
ILMN_1772036	0.158129	6.536796	4.559152	3.33E-05	0.00377	2.182082
ILMN_1654262	-0.15806	8.417803	-4.54855	3.45E-05	0.003854	2.148588
ILMN_1667295	-0.15754	7.862332	-3.47766	0.001056	0.031069	-1.04024
ILMN_1748352	-0.15739	11.41013	-3.79721	0.000397	0.017953	-0.13477
ILMN_1775405	-0.15729	9.581759	-4.54356	3.51E-05	0.003881	2.132827
ILMN_1778543	0.157284	8.784348	4.260866	8.97E-05	0.007096	1.25201

ILMN_2214678	-0.15721	7.753444	-3.24548	0.002093	0.048075	-1.66812
ILMN_2072622	0.157172	9.12022	4.198378	0.00011	0.007858	1.060656
ILMN_2357134	0.156518	7.806797	3.527337	0.000909	0.028593	-0.90249
ILMN_2401873	0.156332	8.387228	4.104447	0.000149	0.009825	0.775486
ILMN_1671404	0.156071	9.585143	3.870029	0.000316	0.015838	0.077687
ILMN_1715684	-0.15592	9.932007	-3.56071	0.000822	0.026754	-0.80929
ILMN_1655622	0.155651	9.877114	4.797425	1.49E-05	0.00235	2.94259
ILMN_1712888	-0.15557	10.95199	-3.3531	0.001529	0.0393	-1.38043
ILMN_1802205	-0.15557	9.479563	-3.81933	0.00037	0.017174	-0.07044
ILMN_3235404	0.155367	7.050826	5.382649	1.95E-06	0.000724	4.862154
ILMN_2121816	-0.1549	6.828963	-5.92802	2.82E-07	0.000202	6.696901
ILMN_1663866	-0.15472	12.16059	-3.51728	0.000937	0.029134	-0.93048
ILMN_1716026	-0.15454	7.359713	-5.03064	6.67E-06	0.001469	3.699736
ILMN_1678922	-0.15433	8.85559	-4.4271	5.18E-05	0.004991	1.767087
ILMN_2413898	0.153064	8.123873	4.091501	0.000156	0.010072	0.736422
ILMN_1669703	-0.15303	8.590866	-5.21128	3.56E-06	0.000997	4.29362
ILMN_1682038	0.152626	8.374774	5.274188	2.86E-06	0.000871	4.501779
ILMN_1685580	-0.15249	7.69523	-5.92334	2.87E-07	0.000202	6.681021
ILMN_1665775	0.152172	6.339835	3.715604	0.000511	0.020688	-0.37024
ILMN_2413899	0.151897	6.61334	4.608783	2.82E-05	0.0034	2.339299
ILMN_1778087	-0.15175	12.66377	-4.56805	3.23E-05	0.003718	2.21022
ILMN_1708672	0.15109	9.817585	3.752235	0.000456	0.019328	-0.26488
ILMN_1696713	0.150861	7.818523	5.154864	4.33E-06	0.00114	4.107506
ILMN_1712305	-0.15068	9.841136	-3.33095	0.001631	0.040776	-1.44011
ILMN_1710284	-0.15068	8.130457	-3.43157	0.001212	0.033552	-1.167
ILMN_1729225	-0.1505	6.254295	-4.30285	7.82E-05	0.00651	1.381284
ILMN_2229379	-0.15049	6.13363	-3.40129	0.001326	0.035568	-1.2497
ILMN_3247848	-0.15014	6.89447	-4.59558	2.95E-05	0.003505	2.297401
ILMN_1694840	-0.15003	7.088939	-3.65641	0.000614	0.022995	-0.53928
ILMN_3250268	0.149967	7.791705	4.080781	0.000161	0.010291	0.70412
ILMN_1763907	0.149787	10.09857	5.881885	3.32E-07	0.000218	6.540521
ILMN_1774685	0.149769	6.321316	4.344314	6.82E-05	0.006106	1.509511
ILMN_1810942	-0.1497	6.842908	-3.49241	0.00101	0.030438	-0.99947
ILMN_1682165	-0.14956	9.81225	-4.01714	0.000198	0.012004	0.513196
ILMN_1805828	0.148963	8.975599	4.704549	2.04E-05	0.002847	2.644465
ILMN_1657708	-0.14885	6.507068	-3.34318	0.001574	0.040075	-1.40718
ILMN_2217809	0.14768	9.865297	3.739592	0.000475	0.019688	-0.30131
ILMN_1760620	0.147482	7.638822	5.459609	1.49E-06	0.000611	5.118913
ILMN_2380588	0.147387	8.905956	5.521962	1.19E-06	0.000513	5.327534
ILMN_1668629	-0.14715	9.24065	-3.58448	0.000765	0.025761	-0.74263
ILMN_1809866	0.146879	8.354651	4.989749	7.68E-06	0.001531	3.566154
ILMN_1664369	0.146224	6.806388	5.00212	7.36E-06	0.001515	3.606532
ILMN_1800412	-0.14598	7.86545	-7.84265	2.89E-10	2.49E-06	13.22439
ILMN_1682738	-0.14562	8.942544	-4.35279	6.63E-05	0.005984	1.535802
ILMN_1669479	0.145339	7.352698	3.886307	0.0003	0.015385	0.125467
ILMN_1759419	0.145267	8.841508	6.045602	1.85E-07	0.000142	7.0962
ILMN_1794914	-0.14514	7.949059	-4.30191	7.84E-05	0.00651	1.378372
ILMN_1711314	0.145106	9.227916	4.43504	5.05E-05	0.004896	1.791903
ILMN_1731433	-0.14429	6.346302	-3.85397	0.000332	0.016239	0.030659
ILMN_1726839	0.144183	10.39428	4.06322	0.000171	0.01075	0.651294
ILMN_1725193	-0.144	10.1647	-6.36167	5.95E-08	6.22E-05	8.173573
ILMN_1782403	0.143901	7.30588	4.576613	3.14E-05	0.00367	2.237319
ILMN_1685722	-0.14378	11.69208	-5.02326	6.84E-06	0.001483	3.675585
ILMN_1693630	-0.14376	6.773813	-3.63516	0.000655	0.023869	-0.59958

ILMN_1810392	0.143717	7.699965	4.916121	9.90E-06	0.001858	3.32649
ILMN_1681249	-0.1436	8.864681	-5.66919	7.09E-07	0.000373	5.822042
ILMN_1744211	-0.14349	6.010109	-3.92264	0.000267	0.014374	0.23249
ILMN_1683127	0.143341	9.525091	3.332825	0.001623	0.040674	-1.43508
ILMN_1733519	0.143145	7.128569	5.31953	2.44E-06	0.000808	4.652212
ILMN_1796417	0.143136	8.302821	4.688061	2.16E-05	0.00287	2.591759
ILMN_1656482	-0.14267	8.367446	-4.64741	2.48E-05	0.003081	2.462092
ILMN_1719938	0.142397	6.741639	4.097019	0.000153	0.009988	0.753065
ILMN_2371055	-0.14236	10.52178	-4.91392	9.97E-06	0.001858	3.319338
ILMN_2166524	0.142351	7.416898	4.842202	1.28E-05	0.002206	3.087044
ILMN_1765109	-0.14229	8.299028	-3.85024	0.000336	0.016288	0.019744
ILMN_2224657	0.141857	6.722118	3.499253	0.000989	0.030107	-0.9805
ILMN_2124352	0.141684	10.23923	4.270792	8.69E-05	0.006983	1.282523
ILMN_1695386	0.141533	8.615201	7.069179	4.65E-09	1.78E-05	10.59342
ILMN_2214910	-0.1415	8.086097	-3.96892	0.000231	0.013128	0.369559
ILMN_1809344	-0.14144	8.703744	-6.18943	1.10E-07	9.26E-05	7.585849
ILMN_2287653	-0.14067	6.690452	-4.69041	2.14E-05	0.00287	2.599276
ILMN_1784847	0.140526	7.592238	5.158274	4.28E-06	0.001135	4.118739
ILMN_1773906	-0.14038	11.75633	-4.76022	1.69E-05	0.002506	2.822902
ILMN_2203588	-0.14027	6.67429	-4.69825	2.08E-05	0.002859	2.624321
ILMN_1651346	-0.13997	6.717753	-4.52618	3.72E-05	0.003986	2.078007
ILMN_1678671	-0.13987	7.812516	-3.60326	0.000722	0.025018	-0.68974
ILMN_2134974	0.139836	10.82826	3.239887	0.002127	0.048536	-1.6829
ILMN_2348050	0.139666	9.946905	3.96914	0.000231	0.013128	0.370199
ILMN_2062701	-0.13922	7.849424	-3.53939	0.000877	0.027932	-0.8689
ILMN_1775522	-0.13888	9.771732	-5.24958	3.11E-06	0.000933	4.420277
ILMN_3306482	0.138855	10.27699	3.644426	0.000637	0.023549	-0.57332
ILMN_3233229	-0.13879	7.717482	-4.75466	1.72E-05	0.002529	2.80505
ILMN_1803194	0.138674	8.461656	5.353379	2.16E-06	0.000769	4.764725
ILMN_1779677	-0.13867	7.562899	-3.44063	0.001179	0.033218	-1.14216
ILMN_2404063	-0.13852	9.331987	-6.68381	1.86E-08	3.38E-05	9.275155
ILMN_1732967	-0.13843	8.497603	-5.03532	6.56E-06	0.001469	3.715027
ILMN_1770206	0.138378	9.242454	6.593717	2.58E-08	4.23E-05	8.966905
ILMN_1731349	-0.13828	6.896797	-3.36411	0.00148	0.038388	-1.35065
ILMN_2414533	-0.13819	7.051803	-5.19975	3.70E-06	0.00103	4.255521
ILMN_1719344	-0.13802	8.761622	-4.33206	7.10E-05	0.006248	1.47157
ILMN_2409220	0.137836	9.171432	3.376778	0.001426	0.03743	-1.31634
ILMN_2297626	0.137796	7.303479	3.294644	0.001814	0.043772	-1.53743
ILMN_1803018	-0.13777	8.220185	-4.37754	6.11E-05	0.005647	1.612654
ILMN_1744835	0.137732	11.05111	4.597184	2.93E-05	0.003499	2.302496
ILMN_2103397	-0.13764	6.499243	-5.00804	7.21E-06	0.001515	3.625858
ILMN_1723971	0.137599	8.306865	3.794034	0.000401	0.01801	-0.14397
ILMN_2079285	-0.13755	9.10756	-3.57024	0.000798	0.026314	-0.78259
ILMN_3237385	-0.13732	8.080546	-5.10481	5.16E-06	0.001243	3.94287
ILMN_1730824	0.137295	7.513409	4.136144	0.000135	0.009129	0.871375
ILMN_1742577	0.13725	10.20435	4.865068	1.18E-05	0.002118	3.160985
ILMN_2334760	-0.13708	6.781816	-3.49614	0.000999	0.03026	-0.98914
ILMN_1711005	0.136866	7.497246	3.885578	0.000301	0.015385	0.123327
ILMN_1797236	-0.13677	6.569355	-3.821	0.000368	0.017154	-0.06557
ILMN_1667319	-0.1366	6.22959	-4.53741	3.59E-05	0.003912	2.113413
ILMN_1903914	0.136455	7.610041	3.330797	0.001632	0.040776	-1.44053
ILMN_2074044	0.136381	7.061313	4.131127	0.000137	0.009224	0.856174
ILMN_1739805	-0.13634	8.701651	-4.80755	1.44E-05	0.00235	2.975208
ILMN_1694106	0.135857	7.791195	3.65844	0.00061	0.022901	-0.53351

ILMN_1796013	0.135659	7.230507	5.029747	6.69E-06	0.001469	3.69681
ILMN_1736327	0.135512	7.254208	3.43222	0.001209	0.033515	-1.16521
ILMN_1716869	-0.13548	6.670323	-4.56076	3.32E-05	0.00377	2.187161
ILMN_1770641	-0.13528	6.005746	-4.93417	9.30E-06	0.001782	3.385125
ILMN_3246206	0.135101	7.060914	3.334752	0.001613	0.0406	-1.42989
ILMN_2082324	0.135069	7.349485	6.780015	1.32E-08	2.84E-05	9.604368
ILMN_2045994	-0.13465	8.174052	-5.28473	2.75E-06	0.000847	4.536723
ILMN_1766658	0.134502	8.098891	4.480062	4.34E-05	0.004446	1.932937
ILMN_2225718	0.13449	7.499277	3.923445	0.000267	0.014374	0.234865
ILMN_2061565	-0.1344	8.34587	-4.45298	4.75E-05	0.004752	1.848028
ILMN_2140700	-0.13424	6.757813	-5.32258	2.41E-06	0.000807	4.662343
ILMN_1726104	0.134232	8.323975	4.414014	5.41E-05	0.00517	1.726236
ILMN_1718972	0.134151	8.220588	4.912736	1.00E-05	0.001858	3.315499
ILMN_1698189	0.13412	8.283864	5.123158	4.84E-06	0.001192	4.003157
ILMN_2344850	-0.13386	10.71219	-6.34387	6.34E-08	6.35E-05	8.112808
ILMN_1660341	-0.13386	8.027969	-4.69	2.14E-05	0.00287	2.59794
ILMN_1666376	-0.13345	7.528633	-6.24549	9.03E-08	8.20E-05	7.77701
ILMN_1795336	0.13325	5.682205	3.744821	0.000467	0.019597	-0.28625
ILMN_1714170	-0.13286	7.992532	-3.76435	0.000439	0.018915	-0.22992
ILMN_1724493	0.132792	8.164774	3.661776	0.000604	0.022767	-0.52402
ILMN_2135984	0.132714	6.588567	3.865031	0.000321	0.015997	0.063041
ILMN_1717326	-0.13227	6.720387	-3.93917	0.000254	0.013842	0.28135
ILMN_1666599	0.132237	7.265358	4.581722	3.09E-05	0.003623	2.253495
ILMN_1832208	0.132046	9.540907	4.738401	1.82E-05	0.002634	2.752887
ILMN_1806809	-0.13201	6.996511	-5.14908	4.42E-06	0.001146	4.088467
ILMN_3274596	-0.13199	10.58903	-4.51368	3.88E-05	0.004079	2.03864
ILMN_2326509	0.131876	8.811935	4.058724	0.000173	0.010808	0.637788
ILMN_1717990	0.131724	7.262928	3.503694	0.000976	0.029813	-0.96819
ILMN_1671791	0.131708	7.469116	3.957201	0.000239	0.013337	0.334761
ILMN_3298716	0.131705	6.642078	3.854134	0.000332	0.016239	0.031134
ILMN_1712400	0.131687	8.876375	3.973425	0.000227	0.013051	0.382931
ILMN_1756595	-0.13147	7.482138	-3.24862	0.002074	0.047831	-1.65981
ILMN_1710622	-0.13124	7.914403	-3.60441	0.000719	0.024955	-0.6865
ILMN_1655876	-0.13119	7.946876	-3.87916	0.000307	0.01557	0.104485
ILMN_1773567	-0.13111	11.08087	-3.30075	0.001782	0.043302	-1.5211
ILMN_3263702	0.131029	6.359064	4.364794	6.37E-05	0.005842	1.573039
ILMN_1709483	0.130638	7.580603	5.078289	5.65E-06	0.001335	3.8558
ILMN_1669323	0.130574	7.255136	3.90671	0.000281	0.014795	0.185501
ILMN_1680579	-0.13049	8.39294	-4.43574	5.04E-05	0.004896	1.794088
ILMN_1788166	0.130487	8.675036	4.409128	5.50E-05	0.005225	1.710996
ILMN_1797332	0.130393	8.23721	3.752921	0.000455	0.019311	-0.2629
ILMN_2414399	0.130313	7.24565	5.019535	6.93E-06	0.001483	3.663423
ILMN_1745034	0.130045	8.710431	4.412001	5.45E-05	0.00519	1.719958
ILMN_2150402	0.129949	6.642874	4.787714	1.54E-05	0.002396	2.911323
ILMN_1655557	-0.12926	8.6097	-3.71113	0.000518	0.020853	-0.38305
ILMN_1669831	0.129228	7.643488	6.779613	1.32E-08	2.84E-05	9.602993
ILMN_2404182	-0.12898	7.265849	-4.84378	1.27E-05	0.002206	3.09215
ILMN_1678678	-0.12889	8.497363	-3.32119	0.001679	0.04148	-1.46633
ILMN_1815121	0.128888	6.009127	4.223343	0.000101	0.00739	1.136949
ILMN_1759023	-0.12882	6.831852	-3.73925	0.000475	0.019688	-0.3023
ILMN_2285375	0.128498	8.553091	3.588041	0.000756	0.025664	-0.73261
ILMN_2151056	-0.12847	7.826808	-4.75403	1.72E-05	0.002529	2.803054
ILMN_2148150	0.128045	6.811128	3.578095	0.00078	0.025917	-0.76055
ILMN_3246538	-0.12801	6.830278	-3.84652	0.00034	0.01641	0.008878

ILMN_1665372	-0.12798	6.998521	-4.19295	0.000112	0.007949	1.044087
ILMN_1748476	0.127974	10.8424	5.14922	4.42E-06	0.001146	4.088917
ILMN_1657697	-0.12791	9.858753	-4.83498	1.31E-05	0.002206	3.063705
ILMN_1717674	-0.12761	9.362655	-3.96807	0.000231	0.013138	0.367015
ILMN_1796589	0.127568	9.182448	4.265463	8.84E-05	0.007039	1.266136
ILMN_1735052	-0.12751	8.102006	-3.6614	0.000604	0.022769	-0.5251
ILMN_1759818	-0.12736	7.741005	-3.54826	0.000853	0.027472	-0.84413
ILMN_1667893	-0.12724	8.518279	-3.32169	0.001676	0.04148	-1.46499
ILMN_1657373	-0.12706	9.93256	-3.31317	0.001719	0.042205	-1.48787
ILMN_2398107	0.126453	7.265734	5.561625	1.04E-06	0.000471	5.460498
ILMN_1777564	0.126416	10.34518	4.686447	2.17E-05	0.00287	2.586605
ILMN_1670353	0.126202	9.008403	3.895258	0.000291	0.015163	0.151785
ILMN_1799128	0.126116	9.232999	5.018451	6.96E-06	0.001483	3.659881
ILMN_1758915	0.12603	8.708471	4.801578	1.47E-05	0.00235	2.955968
ILMN_1713764	-0.12573	6.910495	-4.31311	7.56E-05	0.006438	1.412951
ILMN_2305112	0.125701	6.280871	6.979482	6.42E-09	1.81E-05	10.28676
ILMN_1815723	0.125541	8.40545	4.804234	1.45E-05	0.00235	2.964527
ILMN_1704793	-0.12528	8.555961	-4.2939	8.05E-05	0.006641	1.353671
ILMN_1703324	0.125235	7.840373	3.850882	0.000335	0.016288	0.021622
ILMN_1784871	0.125199	10.97356	3.416083	0.001269	0.034716	-1.20936
ILMN_2085525	0.125178	7.593519	4.701681	2.06E-05	0.002852	2.635294
ILMN_1776337	0.125156	8.179413	3.534365	0.00089	0.028141	-0.88291
ILMN_1811472	0.124959	8.848215	3.522106	0.000924	0.028843	-0.91705
ILMN_1700507	-0.12492	9.003335	-3.22733	0.002206	0.049867	-1.71604
ILMN_1789510	0.124793	9.487129	3.317421	0.001697	0.041801	-1.47646
ILMN_2367753	-0.12462	8.28046	-3.61961	0.000687	0.024514	-0.64359
ILMN_1749930	0.124601	7.864206	5.18529	3.90E-06	0.001066	4.20781
ILMN_1669981	-0.12436	6.921259	-4.79146	1.52E-05	0.002378	2.923371
ILMN_1725366	0.124297	6.520463	3.67845	0.000573	0.022063	-0.47652
ILMN_1801939	0.12393	9.923411	3.578857	0.000778	0.025882	-0.75841
ILMN_1772702	0.123745	9.274788	4.542376	3.53E-05	0.003884	2.129089
ILMN_3300198	-0.12362	7.088251	-3.5916	0.000748	0.025573	-0.72259
ILMN_1735762	-0.12357	6.867633	-4.23122	9.89E-05	0.007317	1.161052
ILMN_1698209	0.12352	8.592883	4.820632	1.37E-05	0.002288	3.017399
ILMN_1659888	0.123509	10.85665	3.615337	0.000696	0.024558	-0.65567
ILMN_1808777	-0.12342	8.520059	-3.87314	0.000313	0.015707	0.086811
ILMN_1759595	-0.12311	7.280271	-8.15814	9.39E-11	1.08E-06	14.28667
ILMN_1897310	-0.12299	8.603914	-5.06404	5.94E-06	0.001384	3.809071
ILMN_2246510	-0.12298	7.998613	-3.63245	0.00066	0.02392	-0.60726
ILMN_2408645	0.122937	8.748486	5.837429	3.90E-07	0.000244	6.389998
ILMN_1810228	0.122751	8.282954	4.765739	1.66E-05	0.002483	2.84065
ILMN_2377185	-0.12267	8.479248	-3.48743	0.001025	0.030581	-1.01324
ILMN_1709085	0.122619	6.344561	4.248314	9.35E-05	0.007224	1.213468
ILMN_1791002	0.122602	7.781896	3.424377	0.001238	0.03413	-1.18668
ILMN_3245983	-0.12257	7.955008	-3.33142	0.001629	0.040776	-1.43886
ILMN_1748438	0.122513	10.48076	4.605514	2.85E-05	0.003425	2.328924
ILMN_2411139	0.122289	6.792351	4.104767	0.000149	0.009825	0.776452
ILMN_1787815	0.122248	7.582959	3.751744	0.000457	0.019334	-0.2663
ILMN_2324056	0.122215	9.370995	3.715225	0.000512	0.020688	-0.37133
ILMN_1651347	-0.12219	9.178162	-5.40189	1.82E-06	0.000707	4.926274
ILMN_2143795	0.122172	10.18029	3.955019	0.000241	0.0134	0.328291
ILMN_1759252	-0.12198	10.14331	-4.9109	1.01E-05	0.001858	3.309549
ILMN_1768662	0.12194	7.893578	4.270014	8.71E-05	0.006983	1.28013
ILMN_1672022	-0.12182	6.385237	-3.41009	0.001292	0.035145	-1.22573

ILMN_3240370	0.121802	7.789924	3.650199	0.000625	0.023311	-0.55693
ILMN_2111187	0.121653	8.370281	3.875261	0.00031	0.01567	0.093035
ILMN_1794132	0.121519	11.12478	3.962679	0.000235	0.01326	0.351014
ILMN_1710752	0.121472	8.511522	4.511958	3.90E-05	0.004079	2.033204
ILMN_1712803	0.12145	9.548288	5.181403	3.95E-06	0.001072	4.194985
ILMN_2090782	0.121389	8.613408	3.337136	0.001602	0.040464	-1.42347
ILMN_2195821	-0.12131	7.004528	-3.4791	0.001051	0.031057	-1.03626
ILMN_3251737	-0.12125	8.954101	-3.48891	0.001021	0.030581	-1.00914
ILMN_1798288	-0.12077	7.041444	-5.04271	6.40E-06	0.001469	3.739217
ILMN_1801934	-0.12061	7.979874	-3.25541	0.002034	0.047268	-1.64183
ILMN_1695475	-0.12053	8.116216	-3.59914	0.000731	0.025207	-0.70136
ILMN_1689059	-0.12047	7.465566	-3.7792	0.00042	0.018405	-0.18697
ILMN_1770665	-0.12034	6.553793	-5.04003	6.45E-06	0.001469	3.73044
ILMN_2080611	0.120276	8.138755	3.803647	0.000389	0.017716	-0.11606
ILMN_1764729	-0.12027	8.524975	-4.24911	9.33E-05	0.007224	1.215926
ILMN_1740185	-0.12025	8.250801	-3.5081	0.000963	0.029551	-0.95596
ILMN_1770692	0.120115	9.212045	4.262789	8.92E-05	0.007073	1.25792
ILMN_1751776	0.119999	9.023057	4.538712	3.57E-05	0.003907	2.117527
ILMN_1791568	0.119972	7.620488	4.241564	9.56E-05	0.007252	1.192764
ILMN_2243308	-0.11992	8.15554	-4.76939	1.64E-05	0.002474	2.852374
ILMN_1779353	0.119768	8.683327	3.434112	0.001202	0.033496	-1.16003
ILMN_2356955	0.119737	6.470147	5.015326	7.03E-06	0.001487	3.649667
ILMN_2319910	-0.11973	7.843609	-4.65401	2.42E-05	0.003035	2.483141
ILMN_1717809	-0.11972	6.697764	-4.33978	6.92E-05	0.006167	1.495464
ILMN_1684108	-0.11969	9.051949	-3.24986	0.002067	0.047755	-1.65652
ILMN_2235137	0.119676	8.759065	3.483268	0.001038	0.030827	-1.02475
ILMN_1790577	0.119657	8.636033	3.252131	0.002053	0.047505	-1.65051
ILMN_1805271	-0.11965	8.035721	-4.86795	1.17E-05	0.002108	3.170315
ILMN_1677098	-0.11938	6.765281	-4.26875	8.75E-05	0.006995	1.276255
ILMN_1838313	0.119202	7.675533	3.631866	0.000662	0.02392	-0.60892
ILMN_1696601	0.119145	8.966241	5.306681	2.55E-06	0.00082	4.609549
ILMN_1732772	0.11908	8.220642	4.999578	7.42E-06	0.001515	3.598232
ILMN_1744138	-0.119	9.242957	-5.46305	1.47E-06	0.000611	5.13042
ILMN_1682197	0.118915	7.729661	4.837027	1.30E-05	0.002206	3.070325
ILMN_1814282	0.118868	8.293627	3.68659	0.000559	0.021735	-0.45328
ILMN_1654385	0.118742	6.037032	4.482789	4.30E-05	0.00443	1.941499
ILMN_1692665	-0.11857	6.993973	-4.33262	7.09E-05	0.006248	1.473303
ILMN_1665526	-0.1184	6.424292	-3.64518	0.000635	0.02352	-0.57116
ILMN_1754149	-0.11832	8.468935	-3.81494	0.000375	0.017296	-0.08322
ILMN_1673721	0.118146	7.484523	3.885196	0.000301	0.015385	0.122203
ILMN_1728714	0.118075	6.99978	3.322234	0.001674	0.04148	-1.46354
ILMN_2077094	0.11805	8.705853	3.461759	0.001107	0.031917	-1.08408
ILMN_1768004	-0.11801	8.515974	-5.792	4.58E-07	0.000282	6.236372
ILMN_2285996	0.117685	10.78016	4.49281	4.16E-05	0.004324	1.972977
ILMN_1712413	0.117647	8.579679	3.974109	0.000227	0.013051	0.384962
ILMN_1667361	-0.11759	6.543923	-5.25559	3.05E-06	0.000922	4.440172
ILMN_1695271	0.117399	8.107913	4.96635	8.33E-06	0.001622	3.489867
ILMN_1656463	0.117398	8.129104	4.800408	1.47E-05	0.00235	2.9522
ILMN_2112460	0.117384	7.469196	5.680739	6.80E-07	0.000373	5.860937
ILMN_1805737	0.117376	9.318393	3.234463	0.002161	0.049076	-1.69723
ILMN_2351638	-0.11712	6.269081	-3.62042	0.000685	0.024488	-0.64129
ILMN_1714352	-0.11702	7.136768	-3.47916	0.001051	0.031057	-1.03611
ILMN_1761946	-0.117	10.03847	-3.3667	0.001469	0.03824	-1.34364
ILMN_1733094	0.11696	7.83028	3.425213	0.001235	0.034084	-1.1844

ILMN_2317730	-0.1169	7.618262	-6.43217	4.62E-08	5.49E-05	8.414484
ILMN_2061732	0.116856	9.634742	3.443856	0.001168	0.033144	-1.1333
ILMN_1773369	0.11685	8.324905	4.153616	0.000127	0.008816	0.924383
ILMN_3239771	0.116848	9.107844	3.573817	0.00079	0.026205	-0.77256
ILMN_1712985	0.116482	8.016082	5.525005	1.18E-06	0.000513	5.337727
ILMN_2108357	0.116327	9.510669	3.710022	0.00052	0.020881	-0.38624
ILMN_2055156	-0.11628	7.259109	-3.70521	0.000528	0.020994	-0.40002
ILMN_1741133	0.116236	12.2059	3.393987	0.001355	0.03607	-1.2696
ILMN_1787879	0.116224	8.903315	4.14458	0.000131	0.008953	0.896956
ILMN_1702322	-0.11621	6.437328	-3.40441	0.001314	0.035472	-1.2412
ILMN_1813775	-0.11607	8.530452	-4.7635	1.67E-05	0.002491	2.833464
ILMN_1652008	0.115689	7.747766	5.658295	7.37E-07	0.000374	5.785363
ILMN_1756550	0.115667	7.648422	4.799325	1.48E-05	0.00235	2.948709
ILMN_1655126	-0.11564	7.973174	-3.70007	0.000536	0.021256	-0.41474
ILMN_1715616	0.115548	9.021957	5.026576	6.76E-06	0.001476	3.686442
ILMN_1700515	0.115507	7.735061	4.270028	8.71E-05	0.006983	1.280173
ILMN_1697317	-0.11544	5.879137	-3.57052	0.000798	0.026314	-0.78182
ILMN_2311537	0.115407	11.27813	3.584224	0.000765	0.025761	-0.74334
ILMN_1704702	0.115406	7.341246	3.706683	0.000526	0.020981	-0.39581
ILMN_1786379	0.115383	6.061254	3.973287	0.000228	0.013051	0.382521
ILMN_2253648	0.115334	7.430884	4.145973	0.000131	0.00893	0.901182
ILMN_1720819	0.115311	10.45168	4.688557	2.15E-05	0.00287	2.593345
ILMN_1689774	-0.11494	9.864237	-3.99189	0.000214	0.012592	0.437883
ILMN_1755862	0.114935	8.186322	3.947055	0.000247	0.013621	0.304689
ILMN_1795704	-0.11476	6.093777	-5.1662	4.16E-06	0.001113	4.144853
ILMN_1676846	0.114759	9.847006	4.556069	3.37E-05	0.00377	2.172337
ILMN_2227968	0.114522	7.650799	5.058441	6.06E-06	0.001401	3.790738
ILMN_2371053	-0.11445	7.79892	-4.32376	7.30E-05	0.006322	1.445878
ILMN_1760802	0.114397	6.722676	4.302284	7.83E-05	0.00651	1.379541
ILMN_2357781	-0.11404	6.984022	-3.75407	0.000454	0.019291	-0.25958
ILMN_1703041	-0.11403	5.96196	-4.55998	3.32E-05	0.00377	2.184688
ILMN_1674421	-0.11387	8.956569	-4.7674	1.65E-05	0.00248	2.845999
ILMN_1686906	-0.11373	6.61997	-3.37615	0.001428	0.037443	-1.31805
ILMN_1743763	-0.11365	8.513739	-3.88483	0.000301	0.015385	0.121121
ILMN_1732750	0.11365	7.464403	4.282679	8.35E-05	0.006858	1.319107
ILMN_1749829	0.113574	9.291547	3.474094	0.001067	0.031176	-1.05008
ILMN_1731070	0.113384	8.203436	4.091641	0.000156	0.010072	0.736843
ILMN_3273047	0.113	6.289677	3.737058	0.000478	0.019733	-0.3086
ILMN_2398926	0.112973	8.502134	5.133474	4.67E-06	0.001174	4.037086
ILMN_3282768	0.112921	8.48882	3.229775	0.00219	0.049614	-1.7096
ILMN_1737728	0.112817	8.578394	3.641547	0.000642	0.023682	-0.58148
ILMN_1780382	0.112814	9.142404	4.439297	4.98E-05	0.004864	1.805212
ILMN_1683598	0.112783	8.471196	3.479071	0.001051	0.031057	-1.03634
ILMN_1722642	-0.11275	7.247962	-3.62991	0.000666	0.024002	-0.61447
ILMN_1676600	-0.11253	9.628447	-3.60772	0.000712	0.024854	-0.67716
ILMN_1663716	-0.11247	6.101307	-4.27622	8.53E-05	0.006955	1.299218
ILMN_1662658	0.112444	9.094335	3.730064	0.000489	0.019993	-0.32872
ILMN_1684217	0.112443	8.660205	4.247815	9.37E-05	0.007224	1.211938
ILMN_3246754	0.112063	6.658273	5.328206	2.36E-06	0.000807	4.681035
ILMN_1730291	-0.11175	8.709407	-3.32636	0.001654	0.041191	-1.45246
ILMN_1796180	-0.11173	7.129997	-5.52294	1.19E-06	0.000513	5.330821
ILMN_2338963	0.111666	7.851581	3.340519	0.001586	0.040212	-1.41436
ILMN_1781039	-0.11153	8.572214	-4.78186	1.57E-05	0.002407	2.892493
ILMN_2382505	-0.11151	6.686503	-4.01562	0.000199	0.01202	0.508654

ILMN_1810069	0.111505	7.707762	4.251394	9.26E-05	0.007221	1.222922
ILMN_1795839	-0.11144	7.585747	-3.88372	0.000302	0.015416	0.117863
ILMN_1725791	0.111393	7.771939	3.873377	0.000312	0.015707	0.087507
ILMN_1794740	-0.11131	8.186942	-3.65901	0.000609	0.022886	-0.53188
ILMN_1719870	0.11131	8.18048	3.894629	0.000292	0.015163	0.149935
ILMN_2070349	0.111135	6.622781	3.935295	0.000257	0.013983	0.269884
ILMN_1689800	0.111079	8.066726	4.447194	4.85E-05	0.004789	1.829917
ILMN_1757230	-0.11106	6.625259	-5.41655	1.73E-06	0.000687	4.97517
ILMN_2316540	0.111024	10.15592	3.827472	0.000361	0.016989	-0.04673
ILMN_1709484	0.110996	6.939209	4.713942	1.98E-05	0.002792	2.674523
ILMN_1727740	0.110981	9.924555	3.839066	0.000348	0.016593	-0.0129
ILMN_1740960	0.110966	7.273288	3.341093	0.001584	0.040178	-1.41282
ILMN_1671933	0.110868	10.0399	3.55597	0.000834	0.027039	-0.82257
ILMN_1721868	0.110856	9.594541	3.492663	0.001009	0.030438	-0.99876
ILMN_1745152	-0.11059	8.553972	-4.98072	7.92E-06	0.001561	3.5367
ILMN_1757697	0.110488	7.179199	4.037381	0.000185	0.01143	0.573767
ILMN_1668996	0.110448	10.9741	4.356257	6.56E-05	0.005933	1.546542
ILMN_1781198	-0.11034	7.040246	-4.66136	2.36E-05	0.003004	2.506555
ILMN_1776788	-0.11028	6.904601	-3.70026	0.000536	0.021256	-0.41421
ILMN_3249406	0.11024	7.633908	4.439635	4.97E-05	0.004864	1.806268
ILMN_1769839	-0.11022	5.604133	-3.81964	0.00037	0.017174	-0.06955
ILMN_1661589	-0.11014	9.039459	-3.56256	0.000817	0.02668	-0.80412
ILMN_1769091	0.11013	10.25775	3.891653	0.000295	0.015237	0.141183
ILMN_1670931	0.109982	8.344569	4.645969	2.49E-05	0.003085	2.457516
ILMN_1757415	0.109799	7.754264	4.346003	6.78E-05	0.006104	1.514745
ILMN_2390609	-0.10968	7.612099	-3.72343	0.000499	0.020264	-0.34777
ILMN_1882000	0.109602	7.486805	4.826209	1.35E-05	0.002255	3.035396
ILMN_1805481	0.109383	8.210688	3.248913	0.002072	0.047831	-1.65903
ILMN_1799667	0.109126	7.599248	4.779955	1.58E-05	0.002407	2.886358
ILMN_1668498	0.109033	8.076128	4.450891	4.79E-05	0.004771	1.841489
ILMN_1686097	0.108979	10.63697	3.782293	0.000416	0.018352	-0.178
ILMN_1745813	-0.10872	9.210892	-3.562	0.000819	0.0267	-0.8057
ILMN_1853876	0.108707	6.888697	4.46005	4.64E-05	0.004695	1.870175
ILMN_2409298	0.108432	9.038152	3.586094	0.000761	0.025703	-0.73808
ILMN_1789123	0.108427	9.000204	4.189343	0.000113	0.008026	1.033093
ILMN_1798690	-0.10832	6.199461	-3.47808	0.001054	0.031069	-1.03909
ILMN_1688295	-0.1083	7.923774	-4.98471	7.82E-06	0.001549	3.549704
ILMN_1739083	-0.10825	7.948388	-4.11232	0.000146	0.009652	0.799266
ILMN_1788211	-0.10823	7.375675	-3.31061	0.001732	0.0424	-1.4947
ILMN_1798459	0.108047	6.719283	3.443016	0.001171	0.033169	-1.13561
ILMN_1656057	-0.10798	10.21881	-3.47636	0.00106	0.031124	-1.04384
ILMN_1753502	-0.10797	5.861393	-5.35768	2.13E-06	0.000765	4.779045
ILMN_2263144	0.107941	7.580538	3.761822	0.000443	0.018993	-0.23721
ILMN_2048700	0.107848	9.063857	3.859505	0.000326	0.016137	0.046854
ILMN_1743620	0.107591	6.116472	4.715564	1.96E-05	0.002788	2.679715
ILMN_1809495	0.10753	12.22347	4.222304	0.000102	0.00739	1.133772
ILMN_2072391	0.107484	8.267508	3.789415	0.000406	0.018103	-0.15737
ILMN_2326512	0.107427	8.422317	3.66896	0.00059	0.022452	-0.50357
ILMN_1770454	-0.10737	9.212353	-3.94522	0.000249	0.013644	0.299254
ILMN_2048507	0.107324	6.934677	3.791896	0.000403	0.018034	-0.15017
ILMN_1734316	0.107294	7.968123	4.556741	3.36E-05	0.00377	2.174463
ILMN_2219712	0.107292	10.62312	4.379147	6.08E-05	0.005632	1.617636
ILMN_2155172	0.107287	9.350678	3.585605	0.000762	0.025703	-0.73945
ILMN_1677843	-0.10718	8.415776	-4.07777	0.000163	0.010372	0.695048

ILMN_2214713	0.107108	6.93969	5.307674	2.54E-06	0.00082	4.612846
ILMN_1683096	0.106993	7.129923	4.890468	1.08E-05	0.001983	3.243258
ILMN_1729987	-0.10698	8.692391	-5.24174	3.20E-06	0.000935	4.394326
ILMN_2401436	0.106948	6.733042	4.685924	2.17E-05	0.00287	2.584932
ILMN_1714710	-0.10689	7.440477	-4.58599	3.05E-05	0.003596	2.267028
ILMN_1672094	-0.10679	6.754803	-3.3576	0.001508	0.038903	-1.36826
ILMN_1776674	0.106736	9.301239	3.512384	0.000951	0.029398	-0.94407
ILMN_1792986	0.106734	7.065098	4.642651	2.52E-05	0.003109	2.446952
ILMN_2405521	0.106707	9.709857	4.181024	0.000116	0.00823	1.007743
ILMN_1761131	0.106698	9.413357	4.454722	4.73E-05	0.004738	1.853485
ILMN_1728262	0.106644	5.633907	3.463121	0.001103	0.031813	-1.08033
ILMN_1786125	0.106629	9.585444	3.532614	0.000895	0.028223	-0.88778
ILMN_1664912	-0.1066	6.625302	-3.43235	0.001209	0.033515	-1.16485
ILMN_1751708	-0.10649	12.29033	-3.59729	0.000735	0.025249	-0.70657
ILMN_2048478	-0.10645	6.143759	-4.04993	0.000178	0.011038	0.611381
ILMN_2344971	0.10637	7.690906	4.399734	5.68E-05	0.005347	1.681715
ILMN_1705301	0.106353	7.378087	3.759485	0.000446	0.019085	-0.24396
ILMN_2347592	0.106341	7.731768	3.390092	0.00137	0.036422	-1.28019
ILMN_3244521	-0.10622	8.874954	-3.37132	0.001449	0.037866	-1.33114
ILMN_1719543	-0.10619	6.558594	-3.81115	0.00038	0.017435	-0.09425
ILMN_1823231	-0.10615	6.043474	-3.33565	0.001609	0.0406	-1.42748
ILMN_1775939	0.10613	9.109342	3.597828	0.000734	0.025233	-0.70506
ILMN_3243248	0.106091	6.648627	4.570821	3.20E-05	0.003708	2.218985
ILMN_2077550	0.106059	9.13064	3.785144	0.000412	0.018281	-0.16975
ILMN_1670542	0.105891	9.474264	3.469227	0.001083	0.031474	-1.0635
ILMN_1773470	-0.10585	7.148702	-3.97793	0.000224	0.013032	0.396337
ILMN_1705907	0.105821	8.640687	4.665055	2.33E-05	0.002989	2.51833
ILMN_1701374	0.105685	8.063289	4.666492	2.32E-05	0.002985	2.522914
ILMN_1700257	0.105617	7.445881	4.305883	7.74E-05	0.006493	1.390647
ILMN_2048633	0.105414	6.738295	3.549354	0.000851	0.027457	-0.84107
ILMN_2063114	0.10541	7.51076	3.475328	0.001063	0.031167	-1.04668
ILMN_1716279	0.105329	8.575244	3.434629	0.001201	0.033496	-1.15861
ILMN_1787415	-0.10531	7.716049	-4.01418	0.0002	0.012033	0.50436
ILMN_1814122	0.105296	8.830415	4.381442	6.03E-05	0.005614	1.624773
ILMN_2404407	-0.10515	6.015306	-4.01841	0.000197	0.011976	0.516995
ILMN_1700633	-0.10507	6.483025	-3.8453	0.000341	0.016419	0.005302
ILMN_2202948	0.105045	8.434508	3.940033	0.000253	0.01384	0.283902
ILMN_1799604	0.105021	10.66305	5.214172	3.52E-06	0.000996	4.303172
ILMN_3256325	-0.10484	7.767774	-4.24989	9.30E-05	0.007224	1.218313
ILMN_1809488	0.104793	10.62056	4.685903	2.17E-05	0.00287	2.584865
ILMN_1700546	0.104696	7.170142	3.633675	0.000658	0.02392	-0.6038
ILMN_1698996	0.104681	6.57796	4.059204	0.000173	0.010808	0.639228
ILMN_1663454	-0.10463	10.93109	-4.79672	1.49E-05	0.00235	2.940333
ILMN_1716524	-0.10462	10.43269	-4.91242	1.00E-05	0.001858	3.31446
ILMN_1765258	-0.10462	9.497473	-4.14715	0.00013	0.00893	0.904765
ILMN_1653134	-0.10451	7.862712	-3.75557	0.000452	0.01927	-0.25525
ILMN_1761069	0.104378	7.418122	3.922834	0.000267	0.014374	0.23306
ILMN_1706817	-0.10421	8.933568	-4.15297	0.000128	0.008817	0.922409
ILMN_1688103	-0.10408	7.79265	-3.86453	0.000321	0.015999	0.061572
ILMN_1714473	-0.10401	6.555985	-4.70191	2.06E-05	0.002852	2.636015
ILMN_2249920	-0.10398	6.030495	-3.69416	0.000546	0.021487	-0.43166
ILMN_1671895	-0.10387	6.998514	-3.83394	0.000354	0.016789	-0.02788
ILMN_1728083	-0.10378	7.764653	-3.96157	0.000236	0.01326	0.347732
ILMN_1703477	-0.10357	8.719924	-4.53361	3.63E-05	0.003925	2.101437

ILMN_1773760	0.103461	10.87738	3.501903	0.000982	0.029895	-0.97316
ILMN_1704253	-0.10344	7.390644	-4.1774	0.000118	0.008294	0.996697
ILMN_1767260	0.103353	7.203757	4.302961	7.82E-05	0.00651	1.381631
ILMN_1690802	0.103281	11.86294	4.060458	0.000172	0.010802	0.642994
ILMN_2306189	-0.10323	8.775216	-4.30795	7.69E-05	0.00648	1.397042
ILMN_1803312	0.10315	10.1723	4.228339	9.98E-05	0.007338	1.152244
ILMN_1744308	0.103132	8.517396	5.650338	7.58E-07	0.000379	5.758581
ILMN_1777853	-0.10299	6.925167	-3.61642	0.000694	0.024558	-0.6526
ILMN_1709634	0.102976	7.720106	3.860199	0.000326	0.016125	0.048887
ILMN_1679177	0.102748	7.84153	3.612938	0.000701	0.024636	-0.66244
ILMN_1815184	0.102649	9.900279	3.369615	0.001456	0.038028	-1.33576
ILMN_1750518	0.102646	9.345884	3.671996	0.000585	0.022356	-0.49492
ILMN_1654493	0.102595	7.359278	4.133932	0.000136	0.009176	0.864672
ILMN_1721022	-0.10247	11.28542	-4.32474	7.27E-05	0.006317	1.4489
ILMN_1655117	0.102468	7.807416	3.655305	0.000616	0.023048	-0.54242
ILMN_1671257	0.102428	10.84111	4.684228	2.19E-05	0.002875	2.579517
ILMN_3245893	0.102396	6.436412	6.38856	5.40E-08	6.01E-05	8.265446
ILMN_3251423	0.102381	6.070937	5.404967	1.80E-06	0.000707	4.936527
ILMN_1802251	-0.10237	9.784165	-4.30952	7.65E-05	0.006463	1.401883
ILMN_1785413	0.102282	6.579484	4.312501	7.57E-05	0.006438	1.411085
ILMN_2350574	-0.10223	7.019527	-3.73853	0.000476	0.019708	-0.30435
ILMN_1678095	0.10218	6.22752	3.816297	0.000374	0.017268	-0.07928
ILMN_1798256	0.102102	10.07763	3.267273	0.001965	0.04627	-1.61034
ILMN_1815885	-0.10199	6.751586	-4.43454	5.06E-05	0.004896	1.790343
ILMN_1744239	0.1018	7.234171	4.28478	8.30E-05	0.006827	1.325577
ILMN_1682095	-0.10171	7.826887	-4.72945	1.87E-05	0.002693	2.724178
ILMN_1688526	0.101658	11.46092	4.71937	1.94E-05	0.002763	2.691902
ILMN_1788416	0.101648	10.07322	3.796697	0.000397	0.017953	-0.13624
ILMN_1734483	-0.10156	8.371628	-4.9943	7.56E-06	0.001524	3.58099
ILMN_1690371	0.101477	9.478241	3.997697	0.00021	0.012381	0.455175
ILMN_1748923	0.101456	7.985495	3.484668	0.001034	0.03075	-1.02087
ILMN_1801118	0.101293	9.449759	3.240504	0.002123	0.048513	-1.68127
ILMN_1804988	-0.10127	8.036671	-3.58706	0.000759	0.025703	-0.73536
ILMN_2198515	-0.10119	6.781701	-3.29003	0.001839	0.044149	-1.54974
ILMN_2319913	-0.10104	8.392875	-3.34509	0.001565	0.03991	-1.40205
ILMN_1682226	-0.10102	6.776447	-4.448	4.83E-05	0.004789	1.832443
ILMN_1691578	0.100982	8.624275	3.502073	0.000981	0.029895	-0.97269
ILMN_1661293	-0.10098	6.006452	-3.80834	0.000383	0.01754	-0.10243
ILMN_1777895	-0.10096	7.506303	-4.70793	2.02E-05	0.002835	2.655279
ILMN_1798654	0.10085	10.43062	4.195555	0.000111	0.007898	1.052039
ILMN_2205999	0.100568	6.563454	3.924201	0.000266	0.014374	0.237098
ILMN_1797307	0.100501	7.472941	3.848809	0.000337	0.016315	0.015561
ILMN_2278636	-0.1005	8.453397	-4.63963	2.54E-05	0.003118	2.437344
ILMN_1723625	0.100414	8.226137	3.366764	0.001468	0.03824	-1.34348
ILMN_1752988	-0.10039	8.508428	-4.27211	8.65E-05	0.006983	1.286581
ILMN_2051373	0.100314	9.100051	3.476034	0.001061	0.031127	-1.04473
ILMN_2312719	0.100212	9.234985	3.783765	0.000414	0.018307	-0.17374
ILMN_2124816	-0.1002	6.47506	-3.47044	0.001079	0.031439	-1.06015
ILMN_1798177	-0.1002	9.656604	-4.67241	2.27E-05	0.002948	2.541781
ILMN_1743911	0.100016	10.10492	3.523956	0.000918	0.02876	-0.9119
ILMN_1712122	0.099963	5.943253	5.641958	7.81E-07	0.000385	5.730385
ILMN_1754234	0.099932	8.750927	3.968838	0.000231	0.013128	0.369301
ILMN_1721741	-0.09988	8.567651	-3.63581	0.000654	0.023869	-0.59775
ILMN_1758633	-0.09975	8.386412	-3.75403	0.000454	0.019291	-0.25969

ILMN_2364357	0.099685	9.160358	3.693608	0.000547	0.021487	-0.43323
ILMN_3241979	0.099636	8.52377	3.54764	0.000855	0.027497	-0.84586
ILMN_1730825	0.099588	7.212852	3.447849	0.001154	0.032859	-1.12234
ILMN_2226304	-0.09956	7.526339	-3.38114	0.001407	0.037092	-1.30452
ILMN_1656670	0.099524	6.613323	4.325975	7.24E-05	0.006307	1.452733
ILMN_1659364	0.099514	9.227493	4.065616	0.000169	0.010687	0.658494
ILMN_1676548	0.099426	10.23764	3.456283	0.001125	0.032201	-1.09916
ILMN_1665205	-0.09931	6.758934	-3.64579	0.000634	0.023517	-0.56943
ILMN_1797298	-0.09923	6.449559	-4.54127	3.54E-05	0.003886	2.12559
ILMN_1737611	-0.09913	6.66995	-5.73728	5.57E-07	0.000325	6.051582
ILMN_3196019	0.099109	7.911907	3.523552	0.00092	0.028769	-0.91302
ILMN_1674302	0.098995	8.246591	3.666252	0.000595	0.022553	-0.51128
ILMN_1672504	-0.09887	9.839358	-3.41672	0.001266	0.034677	-1.2076
ILMN_1765858	0.09884	11.0411	4.465214	4.56E-05	0.004629	1.886361
ILMN_1705201	-0.09881	6.145961	-3.52643	0.000912	0.028625	-0.90502
ILMN_1805104	-0.0987	5.880778	-3.54501	0.000862	0.027588	-0.8532
ILMN_1733956	0.098658	9.365749	3.550935	0.000847	0.027378	-0.83665
ILMN_1741736	0.098593	8.045154	4.992303	7.61E-06	0.001526	3.574486
ILMN_1780937	0.098485	7.910153	4.655286	2.41E-05	0.003035	2.487193
ILMN_1753482	0.098299	6.52185	3.730084	0.000489	0.019993	-0.32866
ILMN_2376667	-0.0983	7.41598	-4.7843	1.55E-05	0.002404	2.900339
ILMN_2041046	0.098247	10.23294	4.331498	7.11E-05	0.006248	1.46982
ILMN_1712320	-0.09825	8.823535	-4.56954	3.22E-05	0.003711	2.21492
ILMN_1657797	0.0982	8.498498	3.549637	0.00085	0.027457	-0.84028
ILMN_1655177	-0.09818	8.835963	-3.80126	0.000392	0.017767	-0.123
ILMN_2320906	0.098165	8.239528	3.781758	0.000416	0.018352	-0.17955
ILMN_2336781	0.098163	9.712383	3.624482	0.000677	0.024328	-0.62982
ILMN_1778059	0.098098	6.052982	4.522756	3.77E-05	0.004007	2.067213
ILMN_1685005	-0.09809	9.236945	-5.08275	5.57E-06	0.001323	3.87045
ILMN_1721713	0.098067	8.741853	3.771376	0.00043	0.018729	-0.2096
ILMN_1792078	-0.0979	9.804828	-4.4912	4.19E-05	0.004333	1.967914
ILMN_2371700	0.097897	7.614581	4.014896	0.000199	0.012026	0.506503
ILMN_2212909	0.097885	10.08082	3.307815	0.001746	0.042537	-1.5022
ILMN_3306997	0.09787	8.643979	4.267601	8.78E-05	0.007006	1.27271
ILMN_2214355	0.09787	6.543851	3.778687	0.00042	0.018411	-0.18845
ILMN_1798705	0.0978	8.716211	3.975846	0.000226	0.013051	0.390127
ILMN_1713406	-0.09778	8.336282	-5.21867	3.47E-06	0.000988	4.31802
ILMN_2210713	0.097758	6.990189	5.141904	4.53E-06	0.001166	4.064831
ILMN_1728934	0.097548	10.36563	3.743649	0.000469	0.019597	-0.28963
ILMN_1723481	-0.0975	7.711944	-4.07113	0.000166	0.010538	0.675082
ILMN_2377862	0.097461	7.906293	3.721934	0.000501	0.020334	-0.35207
ILMN_1682336	0.097406	7.018467	3.631373	0.000663	0.02392	-0.61032
ILMN_3231638	-0.09728	7.788437	-3.24606	0.00209	0.048026	-1.66658
ILMN_1791569	-0.09725	7.56294	-3.49582	0.001	0.03026	-0.99002
ILMN_1675130	0.097201	7.106587	3.365542	0.001474	0.038313	-1.34679
ILMN_1708787	-0.09716	6.52756	-4.12218	0.000141	0.009403	0.829077
ILMN_1701269	0.097152	10.9199	3.826684	0.000362	0.016989	-0.04902
ILMN_1741219	-0.09714	7.24337	-3.747	0.000464	0.019525	-0.27996
ILMN_1704571	-0.09711	8.266542	-3.6743	0.000581	0.022272	-0.48834
ILMN_2327994	0.096959	7.877695	3.396451	0.001345	0.035999	-1.26289
ILMN_1716445	0.096808	9.710095	3.753548	0.000454	0.019297	-0.26109
ILMN_1667977	0.096763	7.875987	5.017969	6.97E-06	0.001483	3.658303
ILMN_1694514	-0.09673	6.798094	-3.70869	0.000522	0.020939	-0.39007
ILMN_2367215	0.096733	8.953831	3.39149	0.001365	0.036306	-1.27639

ILMN_1710064	-0.09641	6.052733	-3.46402	0.0011	0.031813	-1.07786
ILMN_1915076	0.096396	6.374949	3.247043	0.002084	0.047921	-1.66398
ILMN_2061768	0.096374	5.620463	4.723059	1.92E-05	0.00274	2.703717
ILMN_1797522	-0.09637	9.338909	-3.83901	0.000348	0.016593	-0.01308
ILMN_3282436	-0.09637	9.636965	-3.45963	0.001114	0.03204	-1.08993
ILMN_1658685	-0.09633	5.723769	-3.63552	0.000654	0.023869	-0.59858
ILMN_2411116	0.096269	6.521317	4.331472	7.11E-05	0.006248	1.469741
ILMN_1788462	0.096224	9.76744	3.850651	0.000335	0.016288	0.020946
ILMN_1685774	0.096196	11.23213	3.463243	0.001102	0.031813	-1.08
ILMN_1743711	0.096185	8.799743	3.906867	0.000281	0.014795	0.185965
ILMN_1685679	-0.09617	7.037924	-4.99556	7.53E-06	0.001524	3.585116
ILMN_1764970	-0.09612	9.031092	-4.13856	0.000134	0.009075	0.878706
ILMN_1746561	-0.09596	9.355075	-4.87971	1.12E-05	0.002046	3.20838
ILMN_1797318	0.095937	7.588091	3.631908	0.000662	0.02392	-0.6088
ILMN_1671906	0.095869	8.231716	3.530466	0.000901	0.028381	-0.89377
ILMN_1786718	0.095789	10.08506	3.519825	0.00093	0.029016	-0.92339
ILMN_2045729	0.095752	9.020965	3.668218	0.000592	0.022468	-0.50568
ILMN_1701289	0.095593	8.383383	3.254162	0.002041	0.047308	-1.64513
ILMN_1743241	-0.09556	7.429253	-3.54617	0.000859	0.027517	-0.84997
ILMN_1780667	0.095546	8.102367	3.376152	0.001428	0.037443	-1.31804
ILMN_1708059	0.095456	6.612322	3.952913	0.000243	0.013412	0.32205
ILMN_2129273	0.095342	6.794774	3.457072	0.001123	0.032179	-1.09698
ILMN_1741942	-0.09529	9.043092	-3.74056	0.000473	0.019679	-0.29852
ILMN_1772124	-0.09525	7.905463	-3.85405	0.000332	0.016239	0.030892
ILMN_1815190	0.095252	7.766689	4.230031	9.93E-05	0.007326	1.157424
ILMN_1691418	-0.09525	6.015237	-3.74168	0.000472	0.019645	-0.29528
ILMN_1787923	-0.09516	7.990188	-3.7929	0.000402	0.018024	-0.14725
ILMN_1657983	-0.09512	8.205449	-4.57586	3.15E-05	0.00367	2.234931
ILMN_1698715	0.095059	6.978506	5.905654	3.05E-07	0.000206	6.621067
ILMN_1802646	-0.095	5.629979	-3.55504	0.000836	0.02709	-0.82518
ILMN_1810418	0.094944	6.766562	4.27089	8.68E-05	0.006983	1.282823
ILMN_3276822	0.094915	6.700761	3.916589	0.000272	0.014527	0.21463
ILMN_2049766	0.094885	7.162144	3.496235	0.000998	0.03026	-0.98886
ILMN_1734830	-0.09486	6.273341	-3.67968	0.000571	0.022053	-0.473
ILMN_1797005	-0.09473	9.38135	-3.89314	0.000293	0.015188	0.145559
ILMN_1718271	-0.09465	8.508077	-3.50847	0.000962	0.029544	-0.95493
ILMN_1695362	-0.09464	7.64827	-3.58078	0.000773	0.02583	-0.75301
ILMN_1740505	-0.0946	6.756571	-5.23829	3.24E-06	0.000938	4.382928
ILMN_1737195	0.094552	9.058671	3.886178	0.0003	0.015385	0.12509
ILMN_2403965	0.094488	7.315688	3.688651	0.000556	0.021646	-0.4474
ILMN_1786108	0.094484	6.541234	3.626279	0.000673	0.024223	-0.62474
ILMN_1701882	0.09448	7.89375	3.905601	0.000282	0.014802	0.182236
ILMN_1747935	-0.09446	8.156604	-4.23297	9.83E-05	0.00731	1.166431
ILMN_3187852	-0.09442	9.630297	-4.14941	0.000129	0.008902	0.911612
ILMN_1743204	-0.09436	6.696148	-4.24123	9.57E-05	0.007252	1.191753
ILMN_1717173	0.09423	9.055678	3.44296	0.001171	0.033169	-1.13576
ILMN_1751773	0.094187	7.174382	4.092573	0.000155	0.010072	0.739654
ILMN_1710150	0.094138	7.049956	4.745963	1.77E-05	0.002589	2.777145
ILMN_1775759	0.094075	7.746172	3.371686	0.001447	0.037853	-1.33015
ILMN_1695946	-0.09399	8.595334	-3.44139	0.001177	0.03317	-1.14007
ILMN_1781281	0.093987	6.702514	4.226582	0.0001	0.007349	1.146864
ILMN_2399310	0.093945	7.050559	3.272325	0.001936	0.045721	-1.59691
ILMN_1699737	0.093791	8.90879	4.236222	9.73E-05	0.00731	1.176391
ILMN_1734290	-0.09364	6.382182	-3.34755	0.001554	0.039681	-1.39542

ILMN_1705403	-0.09364	5.593937	-4.40523	5.57E-05	0.005264	1.698849
ILMN_1771738	0.093527	7.554359	5.304366	2.57E-06	0.00082	4.601869
ILMN_2413644	0.093413	5.945823	3.306161	0.001754	0.042713	-1.50663
ILMN_1765523	-0.09331	7.115895	-3.60158	0.000726	0.025096	-0.69448
ILMN_2392546	0.093287	10.91639	3.350509	0.00154	0.039483	-1.38742
ILMN_3307930	0.093205	12.3474	3.910949	0.000277	0.01471	0.197997
ILMN_1790625	0.093125	9.523981	3.954719	0.000241	0.0134	0.327401
ILMN_1678300	0.093042	9.174469	3.433834	0.001203	0.033496	-1.16079
ILMN_2097185	-0.09302	7.903364	-3.50973	0.000959	0.029485	-0.95145
ILMN_3305871	0.092912	6.858199	3.846031	0.00034	0.016412	0.00744
ILMN_1745217	-0.09284	8.509883	-4.25599	9.12E-05	0.007161	1.237045
ILMN_1759175	0.092759	6.883299	4.177675	0.000118	0.008294	0.997543
ILMN_2100458	0.092743	6.719361	3.785811	0.000411	0.018281	-0.16781
ILMN_1670821	0.092722	6.174755	4.235259	9.76E-05	0.00731	1.173439
ILMN_3206242	0.092663	7.382247	3.328579	0.001643	0.040977	-1.4465
ILMN_1663390	0.092612	10.3255	3.286513	0.001858	0.04448	-1.55913
ILMN_1746948	-0.09256	7.200818	-3.46426	0.001099	0.031813	-1.0772
ILMN_1755749	0.092555	10.96564	4.331074	7.12E-05	0.006248	1.468508
ILMN_1722089	0.092493	7.619406	3.998623	0.00021	0.012375	0.457937
ILMN_1746699	0.092477	7.767765	3.491013	0.001014	0.030512	-1.00333
ILMN_1675038	-0.0924	8.060328	-4.06004	0.000172	0.010802	0.641731
ILMN_3185161	0.092346	6.903838	4.781019	1.57E-05	0.002407	2.889779
ILMN_2347917	0.092292	8.30568	4.08584	0.000159	0.010162	0.719359
ILMN_1739103	0.092155	6.763199	3.85382	0.000332	0.016239	0.030216
ILMN_3305304	0.092123	9.246339	4.243859	9.49E-05	0.007245	1.199804
ILMN_1703946	0.092018	8.504249	3.253801	0.002043	0.047308	-1.64608
ILMN_1745904	-0.09197	10.72794	-3.35112	0.001538	0.039441	-1.38578
ILMN_1651433	0.091867	8.086261	3.607912	0.000712	0.024854	-0.67663
ILMN_1669424	0.091807	11.80404	3.567757	0.000804	0.026488	-0.78956
ILMN_1898692	0.091592	6.279318	4.844599	1.27E-05	0.002206	3.094789
ILMN_1723124	-0.09154	6.993052	-3.842	0.000345	0.016505	-0.00433
ILMN_2311089	0.091511	7.284059	3.636934	0.000651	0.023869	-0.59456
ILMN_1754178	0.091488	10.43995	3.816932	0.000373	0.017257	-0.07743
ILMN_1685258	0.09147	10.63855	4.098623	0.000152	0.009966	0.757903
ILMN_2384536	0.091353	8.082153	3.705597	0.000527	0.020993	-0.39892
ILMN_1658695	0.091323	8.046687	3.428205	0.001224	0.033808	-1.17621
ILMN_1815012	-0.09132	8.664127	-3.77116	0.00043	0.018729	-0.21023
ILMN_2048636	0.091279	7.645362	3.790072	0.000406	0.01809	-0.15546
ILMN_2400874	0.091208	9.201289	3.748326	0.000462	0.019469	-0.27615
ILMN_1677906	0.09119	9.433026	4.524611	3.74E-05	0.003995	2.07306
ILMN_1733937	0.090994	7.646218	3.362135	0.001488	0.038554	-1.35601
ILMN_1900270	0.090849	6.401475	3.245125	0.002095	0.048092	-1.66905
ILMN_1765558	-0.09084	7.628369	-3.45972	0.001114	0.03204	-1.08969
ILMN_1722674	0.090791	6.699466	3.96716	0.000232	0.013155	0.364318
ILMN_2261784	0.09073	8.13697	3.844571	0.000342	0.016419	0.003176
ILMN_1783546	-0.09066	8.003033	-3.27819	0.001903	0.045121	-1.5813
ILMN_2379469	0.090616	11.18543	4.40824	5.52E-05	0.005226	1.708228
ILMN_1754179	-0.09059	8.305198	-4.73948	1.81E-05	0.002634	2.756361
ILMN_1768969	0.090542	7.446226	3.649083	0.000628	0.023365	-0.5601
ILMN_2128750	-0.09043	10.87379	-4.65768	2.39E-05	0.003031	2.49482
ILMN_3234756	0.090421	7.0363	3.744771	0.000467	0.019597	-0.28639
ILMN_3179620	0.090407	6.460925	4.56534	3.26E-05	0.003739	2.201648
ILMN_1692790	0.090383	8.579602	3.690737	0.000552	0.021556	-0.44144
ILMN_1756542	-0.09034	6.823393	-3.81182	0.000379	0.017435	-0.0923

ILMN_2178226	0.090328	5.761471	3.980397	0.000222	0.012952	0.403659
ILMN_1805827	-0.0903	12.81968	-3.82692	0.000361	0.016989	-0.04833
ILMN_3307266	0.090183	7.157899	4.624264	2.68E-05	0.003272	2.38847
ILMN_1673991	0.090175	10.46295	3.59007	0.000752	0.025581	-0.7269
ILMN_2325394	0.090164	6.413411	4.202392	0.000109	0.007788	1.072909
ILMN_1781419	0.090137	8.999329	3.552638	0.000842	0.027262	-0.83189
ILMN_3272768	0.09012	6.624888	5.296918	2.64E-06	0.000834	4.577153
ILMN_2110829	0.090066	9.06234	4.546465	3.48E-05	0.003868	2.142001
ILMN_2350114	-0.09006	6.47765	-3.76727	0.000435	0.018838	-0.22149
ILMN_1766637	0.089845	8.528574	3.965148	0.000233	0.013196	0.358345
ILMN_1733757	0.089843	10.38152	3.234662	0.00216	0.049076	-1.6967
ILMN_2141259	0.089827	6.809435	5.095506	5.33E-06	0.001275	3.912299
ILMN_1761531	-0.08974	7.048732	-4.95667	8.61E-06	0.001667	3.458328
ILMN_2179837	0.089665	10.82075	3.737278	0.000478	0.019733	-0.30797
ILMN_1766185	0.089639	8.20693	3.771329	0.00043	0.018729	-0.20974
ILMN_1788149	-0.08958	5.95601	-3.54624	0.000859	0.027517	-0.84978
ILMN_1664167	0.089514	10.25886	4.340535	6.90E-05	0.006167	1.4978
ILMN_2059797	-0.08946	7.313495	-3.68317	0.000565	0.021901	-0.46306
ILMN_1815882	0.089406	6.880175	3.952919	0.000243	0.013412	0.322067
ILMN_1736130	0.089314	7.127001	3.620793	0.000684	0.024488	-0.64025
ILMN_1703005	-0.0892	10.44196	-3.24864	0.002074	0.047831	-1.65977
ILMN_1690352	-0.08915	8.411519	-3.25943	0.00201	0.046877	-1.63116
ILMN_1744046	0.08911	7.48544	3.709958	0.00052	0.020881	-0.38643
ILMN_2359453	-0.0891	10.66599	-3.6021	0.000725	0.025082	-0.69303
ILMN_1807243	0.08904	7.320598	3.680125	0.00057	0.022048	-0.47174
ILMN_1808354	0.089028	6.543877	4.786569	1.54E-05	0.002396	2.907637
ILMN_2298936	0.08898	10.25468	3.754698	0.000453	0.019291	-0.25777
ILMN_1812441	-0.08894	8.001096	-4.04156	0.000183	0.011298	0.586289
ILMN_1764323	0.088928	9.198453	4.519734	3.80E-05	0.004029	2.057692
ILMN_1658027	0.088786	7.183461	3.841573	0.000345	0.016505	-0.00558
ILMN_2222880	-0.08878	6.836397	-3.33813	0.001597	0.040435	-1.4208
ILMN_1807540	-0.08865	7.603404	-3.44223	0.001174	0.033169	-1.13777
ILMN_1815024	0.0886	10.93684	3.322375	0.001673	0.04148	-1.46316
ILMN_1722276	-0.08855	8.935431	-3.94592	0.000248	0.013644	0.301327
ILMN_1774890	0.088423	7.438108	4.614848	2.76E-05	0.003354	2.358555
ILMN_1799367	0.088394	10.52339	3.311507	0.001727	0.04235	-1.49231
ILMN_2395240	0.088353	7.737161	3.692482	0.000549	0.021538	-0.43645
ILMN_2334204	0.088293	6.883614	3.596601	0.000737	0.025277	-0.70852
ILMN_1752270	0.088241	6.838127	4.589765	3.01E-05	0.003562	2.278978
ILMN_3280667	0.088137	6.652168	5.066729	5.88E-06	0.00138	3.817897
ILMN_3228585	-0.08805	8.422743	-3.43591	0.001196	0.033496	-1.15511
ILMN_1801600	-0.08803	8.147344	-3.62212	0.000682	0.024479	-0.63651
ILMN_2396956	-0.088	8.230168	-3.45983	0.001114	0.03204	-1.08939
ILMN_1658437	0.087994	8.967506	3.24452	0.002099	0.048112	-1.67065
ILMN_1687971	-0.08797	6.275943	-3.5352	0.000888	0.02811	-0.88058
ILMN_3248758	0.087866	6.366765	4.01892	0.000197	0.011976	0.518527
ILMN_1693771	0.087761	7.766399	3.313234	0.001718	0.042205	-1.48768
ILMN_1738767	-0.08773	8.400323	-4.01149	0.000201	0.012095	0.496342
ILMN_2332558	0.087685	9.264805	3.909933	0.000278	0.014712	0.195001
ILMN_2168992	0.087567	7.43552	4.613289	2.78E-05	0.00336	2.353603
ILMN_1813240	0.087538	10.31536	3.338601	0.001595	0.040409	-1.41953
ILMN_1664608	-0.08753	8.508847	-3.33482	0.001613	0.0406	-1.42969
ILMN_2054145	0.087506	8.440683	3.414394	0.001275	0.034835	-1.21397
ILMN_1732725	0.087491	7.960482	3.489471	0.001019	0.030581	-1.00759

ILMN_1671809	-0.0874	8.4693	-3.60512	0.000718	0.024926	-0.6845
ILMN_1797367	-0.08727	7.058893	-3.74307	0.00047	0.019597	-0.29128
ILMN_1693221	0.087214	7.609533	3.588494	0.000755	0.025654	-0.73133
ILMN_2386100	0.08713	9.046575	3.586667	0.000759	0.025703	-0.73647
ILMN_1669308	0.087104	7.241029	4.357398	6.53E-05	0.005933	1.550082
ILMN_1672759	-0.08708	8.162983	-3.74382	0.000468	0.019597	-0.28914
ILMN_1846771	-0.087	6.375979	-3.34968	0.001544	0.03952	-1.38965
ILMN_1689968	-0.08693	6.296841	-3.5166	0.000939	0.02914	-0.93234
ILMN_1730504	-0.08683	6.258881	-4.35997	6.48E-05	0.005906	1.558051
ILMN_2127477	0.08656	7.228714	3.279431	0.001897	0.045001	-1.578
ILMN_1653553	0.086531	6.729792	3.919256	0.00027	0.014439	0.222498
ILMN_1672553	0.086432	5.859542	3.662729	0.000602	0.022725	-0.52131
ILMN_1801378	0.086427	8.348091	3.494354	0.001004	0.030366	-0.99407
ILMN_1727300	-0.08639	7.468963	-4.65465	2.42E-05	0.003035	2.485162
ILMN_1702231	-0.08628	6.19729	-3.89986	0.000287	0.015027	0.165338
ILMN_1688089	0.086247	10.74743	4.027276	0.000191	0.011744	0.543514
ILMN_2129388	-0.08624	7.4324	-3.57103	0.000796	0.026314	-0.78038
ILMN_1786976	-0.08622	8.587619	-3.53794	0.00088	0.027977	-0.87294
ILMN_1679881	0.086126	7.188561	3.3374	0.001601	0.040464	-1.42276
ILMN_2333687	-0.08611	6.597974	-3.85092	0.000335	0.016288	0.021745
ILMN_1665219	-0.0861	7.18645	-4.30143	7.85E-05	0.00651	1.376917
ILMN_1668605	0.086063	7.037081	4.094621	0.000154	0.010027	0.745832
ILMN_1751984	-0.08598	7.97319	-5.17162	4.09E-06	0.001101	4.162726
ILMN_2055165	-0.08588	11.24432	-3.5241	0.000918	0.02876	-0.91149
ILMN_1730799	-0.08588	7.340899	-3.23454	0.00216	0.049076	-1.69703
ILMN_1733690	0.085881	6.916004	3.768766	0.000433	0.018774	-0.21715
ILMN_2393544	-0.08584	7.652228	-4.42374	5.24E-05	0.005033	1.756601
ILMN_1802377	-0.08582	6.874295	-3.63528	0.000655	0.023869	-0.59924
ILMN_2043828	0.085792	6.634683	3.606668	0.000715	0.024876	-0.68014
ILMN_1668465	0.085735	6.090794	4.112777	0.000145	0.009652	0.800651
ILMN_3263329	0.085579	8.816942	3.275277	0.00192	0.045423	-1.58906
ILMN_2399523	-0.08557	7.83665	-3.50468	0.000973	0.029778	-0.96546
ILMN_1784367	0.085471	7.22628	3.645566	0.000634	0.023517	-0.57008
ILMN_3182422	-0.0854	7.415648	-3.40974	0.001293	0.035153	-1.22667
ILMN_1770053	0.085396	10.21354	4.210583	0.000106	0.007599	1.097927
ILMN_1733875	-0.08539	7.811225	-3.84374	0.000343	0.016439	0.000749
ILMN_1672389	0.08537	9.105181	4.397957	5.71E-05	0.005349	1.67618
ILMN_1740005	0.085322	6.181646	3.253961	0.002042	0.047308	-1.64566
ILMN_2061405	0.08531	9.813282	3.439962	0.001182	0.03323	-1.14399
ILMN_1814074	-0.08523	7.334847	-3.26108	0.002	0.046758	-1.62677
ILMN_1755822	0.085191	6.523157	4.233023	9.83E-05	0.00731	1.166587
ILMN_1759954	0.085123	11.03952	4.471564	4.47E-05	0.004545	1.906273
ILMN_1811104	-0.08511	9.073405	-3.77376	0.000427	0.018648	-0.20271
ILMN_2206344	-0.08506	6.519048	-4.64749	2.47E-05	0.003081	2.462371
ILMN_2412172	-0.08505	6.932103	-3.69369	0.000547	0.021487	-0.43299
ILMN_1772713	-0.08504	9.535364	-3.45053	0.001145	0.032625	-1.11498
ILMN_2174380	0.084822	6.2976	3.844757	0.000342	0.016419	0.003718
ILMN_1694752	-0.0848	7.069329	-4.24355	9.50E-05	0.007245	1.198861
ILMN_1782439	-0.08466	10.19497	-4.27804	8.48E-05	0.00693	1.304826
ILMN_2201533	0.084621	9.821846	3.636454	0.000652	0.023869	-0.59592
ILMN_3307700	0.084519	8.613779	3.320574	0.001682	0.04148	-1.468
ILMN_1701308	-0.08438	6.375542	-3.61922	0.000688	0.024517	-0.64469
ILMN_1731612	0.084301	9.136684	3.285415	0.001864	0.04455	-1.56205
ILMN_2048822	0.084167	7.98774	3.775983	0.000424	0.018543	-0.19627

ILMN_1761058	-0.0841	7.335681	-4.32297	7.32E-05	0.006322	1.44345
ILMN_1786016	0.084098	7.078864	4.449472	4.81E-05	0.00478	1.837049
ILMN_1677747	0.084084	6.347556	3.488612	0.001022	0.030581	-1.00997
ILMN_1655206	-0.08401	6.955883	-4.51284	3.89E-05	0.004079	2.035988
ILMN_1805658	0.083964	9.531575	4.212122	0.000105	0.007592	1.102632
ILMN_1709479	0.083826	10.3083	3.352089	0.001533	0.039387	-1.38316
ILMN_1792748	-0.08379	6.144422	-3.42422	0.001238	0.03413	-1.18712
ILMN_2333865	-0.0837	7.367843	-3.62065	0.000685	0.024488	-0.64065
ILMN_1766000	0.083672	8.079881	3.614132	0.000698	0.024597	-0.65907
ILMN_1724907	0.083636	8.172697	3.396137	0.001346	0.036004	-1.26374
ILMN_2383419	0.083612	6.691453	3.76531	0.000438	0.018882	-0.22714
ILMN_1728059	0.083584	7.675856	3.241936	0.002115	0.048409	-1.67749
ILMN_1719256	0.083579	10.98633	3.541397	0.000871	0.027789	-0.86329
ILMN_1744268	-0.08358	8.45024	-3.47446	0.001066	0.031169	-1.04908
ILMN_1781001	0.083556	6.255321	4.536037	3.60E-05	0.003915	2.109088
ILMN_1727281	0.083553	7.373109	3.730228	0.000489	0.019993	-0.32824
ILMN_2389810	0.08355	7.036976	4.440151	4.96E-05	0.004864	1.807882
ILMN_3309694	0.083544	6.60877	4.24541	9.44E-05	0.007245	1.20456
ILMN_3258795	-0.08343	8.134018	-3.59475	0.000741	0.025369	-0.71371
ILMN_1693004	-0.08337	7.82199	-3.23461	0.00216	0.049076	-1.69683
ILMN_1734153	-0.08336	7.901925	-3.82568	0.000363	0.017019	-0.05196
ILMN_1684771	0.083242	9.93225	5.324702	2.39E-06	0.000807	4.669393
ILMN_1793033	0.083234	7.364432	3.642537	0.00064	0.023635	-0.57868
ILMN_2184640	0.08322	8.438208	3.455299	0.001129	0.032269	-1.10186
ILMN_1712894	-0.08297	5.769308	-4.54417	3.51E-05	0.003881	2.134767
ILMN_2332691	-0.08297	5.726569	-3.86732	0.000318	0.015905	0.069734
ILMN_1758173	0.082933	9.107964	3.915868	0.000273	0.014527	0.212501
ILMN_1737360	-0.08292	5.933497	-3.95177	0.000244	0.013439	0.318665
ILMN_2181125	-0.08282	7.1485	-3.97332	0.000227	0.013051	0.382629
ILMN_1758672	0.082822	6.513335	3.491768	0.001012	0.03047	-1.00123
ILMN_1813836	0.08282	11.55781	4.296619	7.98E-05	0.006598	1.362066
ILMN_1787280	0.082812	8.156233	3.595238	0.00074	0.025357	-0.71235
ILMN_1806705	-0.08279	6.603107	-3.48101	0.001045	0.030982	-1.03097
ILMN_2341952	0.082774	9.93769	3.671564	0.000586	0.022361	-0.49615
ILMN_1802894	0.082693	8.105851	3.58096	0.000773	0.02583	-0.75251
ILMN_3265365	0.082645	7.30924	3.728909	0.000491	0.020041	-0.33204
ILMN_2149053	0.082626	8.052889	3.832433	0.000355	0.01681	-0.03226
ILMN_1682818	-0.08259	6.036895	-3.65471	0.000617	0.023065	-0.5441
ILMN_1658110	0.082474	7.626379	3.824949	0.000364	0.017034	-0.05408
ILMN_2081335	0.082468	9.44052	4.129928	0.000138	0.009242	0.852542
ILMN_2364131	-0.08242	8.838955	-4.31496	7.51E-05	0.006438	1.418687
ILMN_2189406	0.082413	6.306606	3.473026	0.00107	0.03123	-1.05303
ILMN_1760635	0.082271	8.191383	3.695128	0.000545	0.021487	-0.42888
ILMN_1813275	-0.0822	10.06034	-3.2861	0.00186	0.044502	-1.56022
ILMN_2313730	-0.08211	11.72703	-3.33504	0.001612	0.0406	-1.42911
ILMN_1767747	0.082073	11.16732	4.061661	0.000171	0.010785	0.646609
ILMN_2224143	0.082045	11.33141	3.600033	0.000729	0.025189	-0.69885
ILMN_1719010	-0.08192	6.77102	-3.68483	0.000562	0.021828	-0.4583
ILMN_1666078	0.081842	7.427113	4.15825	0.000125	0.008702	0.938459
ILMN_1750088	0.081809	8.257719	3.484047	0.001036	0.030781	-1.02259
ILMN_1728975	0.081758	9.406542	4.804829	1.45E-05	0.00235	2.966445
ILMN_2407799	0.081753	6.374703	4.356171	6.56E-05	0.005933	1.546274
ILMN_1667577	0.081696	8.29085	4.330351	7.14E-05	0.006248	1.46627
ILMN_1762529	0.081631	7.058434	3.359072	0.001502	0.038788	-1.36429

ILMN_1731184	0.081574	9.208038	3.325029	0.00166	0.041263	-1.45604
ILMN_1704842	0.081547	6.070801	3.226334	0.002212	0.049948	-1.71867
ILMN_1808047	0.081517	6.950668	3.488103	0.001023	0.030581	-1.01138
ILMN_1678037	0.081462	7.767013	3.527094	0.00091	0.028593	-0.90316
ILMN_2357193	0.0814	6.711349	3.637578	0.00065	0.023869	-0.59274
ILMN_1659343	0.081338	10.95083	4.247954	9.36E-05	0.007224	1.212365
ILMN_1663383	0.08133	6.846656	3.617404	0.000692	0.024553	-0.64983
ILMN_3207933	0.081306	9.839309	3.3181	0.001694	0.041748	-1.47464
ILMN_2043306	-0.08129	5.970853	-3.43437	0.001202	0.033496	-1.15931
ILMN_1745593	0.081288	7.46409	3.724303	0.000498	0.020244	-0.34527
ILMN_1660844	0.081264	8.236984	4.224095	0.000101	0.00739	1.139251
ILMN_1805863	-0.08117	7.372344	-3.44022	0.001181	0.03323	-1.14328
ILMN_2054362	0.08103	7.511284	4.051363	0.000177	0.011007	0.615689
ILMN_1784328	0.080957	6.665213	3.694721	0.000545	0.021487	-0.43005
ILMN_1680196	0.080947	10.99316	3.706282	0.000526	0.020981	-0.39696
ILMN_1815306	-0.08091	7.293171	-3.53515	0.000888	0.02811	-0.88071
ILMN_3246391	0.080854	7.146863	3.265377	0.001976	0.046399	-1.61538
ILMN_1749709	-0.08083	10.32535	-3.30099	0.001781	0.043302	-1.52046
ILMN_1690252	0.080797	7.900762	3.404312	0.001314	0.035472	-1.24148
ILMN_1787511	0.080774	7.670593	4.312161	7.58E-05	0.006438	1.410034
ILMN_1739199	-0.08075	7.359269	-3.60801	0.000712	0.024854	-0.67636
ILMN_1682449	-0.0807	8.461128	-3.7436	0.000469	0.019597	-0.28977
ILMN_2179726	0.080692	6.727532	4.682542	2.20E-05	0.002875	2.574133
ILMN_1811592	0.080626	8.778322	3.240936	0.002121	0.048485	-1.68013
ILMN_2411559	0.08057	7.212475	4.535305	3.61E-05	0.003915	2.106779
ILMN_2381603	0.080472	6.52753	4.326199	7.24E-05	0.006307	1.453427
ILMN_1791186	0.080464	6.060227	3.566311	0.000808	0.026563	-0.79361
ILMN_1675186	0.080395	7.954593	3.741507	0.000472	0.019645	-0.29579
ILMN_1659564	-0.08036	9.782527	-3.78092	0.000417	0.018376	-0.18198
ILMN_1670948	-0.08034	8.812808	-3.83179	0.000356	0.01681	-0.03413
ILMN_1769751	-0.08026	7.054067	-4.00363	0.000206	0.012278	0.472882
ILMN_1791508	-0.08026	6.284753	-3.71453	0.000513	0.020708	-0.37332
ILMN_1680386	0.080169	6.946894	4.805962	1.44E-05	0.00235	2.970095
ILMN_1696133	0.080143	6.850996	3.267319	0.001965	0.04627	-1.61022
ILMN_2221014	0.080079	6.324599	3.71165	0.000518	0.020845	-0.38158
ILMN_3248701	0.080074	5.966318	3.557982	0.000829	0.0269	-0.81694
ILMN_1802157	0.080044	8.26044	5.000485	7.40E-06	0.001515	3.601193
ILMN_2313821	0.080043	8.017746	5.242097	3.20E-06	0.000935	4.39551
ILMN_1772894	0.080042	6.317472	3.677669	0.000575	0.022073	-0.47874
ILMN_3239130	-0.07999	6.88769	-3.98914	0.000216	0.012681	0.429689
ILMN_1730523	0.079994	7.930678	3.322259	0.001674	0.04148	-1.46347
ILMN_3246353	0.079957	7.169918	3.480361	0.001047	0.031017	-1.03278
ILMN_3228700	0.079955	6.753811	3.868023	0.000318	0.015893	0.071807
ILMN_1772703	0.079913	7.706179	3.583369	0.000767	0.025767	-0.74574
ILMN_1664030	0.079805	7.490503	3.432207	0.001209	0.033515	-1.16525
ILMN_1710756	0.079783	13.051	3.496795	0.000997	0.03026	-0.98731
ILMN_3295874	-0.0797	6.768968	-3.28323	0.001876	0.044659	-1.56787
ILMN_1753613	-0.07968	7.234789	-3.38911	0.001374	0.036444	-1.28286
ILMN_3248263	-0.07964	7.573761	-3.89703	0.00029	0.015117	0.156992
ILMN_1669598	0.079614	7.075846	4.670027	2.29E-05	0.002961	2.53419
ILMN_1778238	0.079583	9.702043	4.272094	8.65E-05	0.006983	1.286527
ILMN_1734826	0.07951	10.01109	3.572528	0.000793	0.02627	-0.77618
ILMN_1674706	0.079505	8.760991	3.636429	0.000652	0.023869	-0.59599
ILMN_1708513	-0.07948	5.683599	-3.8151	0.000375	0.017296	-0.08275

ILMN_1793476	-0.07947	8.059651	-3.54726	0.000856	0.027503	-0.8469
ILMN_1793371	-0.07947	8.352824	-3.58083	0.000773	0.02583	-0.75287
ILMN_2350801	-0.07937	6.091863	-4.23287	9.84E-05	0.00731	1.166133
ILMN_2395236	0.079362	7.819576	3.452316	0.001139	0.032531	-1.11006
ILMN_1782954	0.07932	9.122717	3.395489	0.001349	0.036018	-1.26551
ILMN_2391512	0.079107	6.565384	3.268316	0.001959	0.046193	-1.60757
ILMN_1737416	-0.07906	7.319151	-3.2631	0.001989	0.046643	-1.62141
ILMN_2405009	-0.07903	9.694206	-3.36608	0.001471	0.038281	-1.34533
ILMN_1750130	0.079019	11.2397	4.002689	0.000207	0.01229	0.470062
ILMN_1690282	0.079003	7.509177	4.086933	0.000158	0.010162	0.722653
ILMN_1796968	0.078969	7.273116	3.678854	0.000573	0.022063	-0.47536
ILMN_1654518	-0.07871	6.571465	-3.36108	0.001493	0.038607	-1.35886
ILMN_1745110	-0.07867	9.549561	-3.85344	0.000333	0.016239	0.029104
ILMN_3212373	0.078631	9.858828	3.535547	0.000887	0.02811	-0.87961
ILMN_1690138	0.078628	7.195188	4.477663	4.38E-05	0.004467	1.925408
ILMN_2347298	-0.07858	6.47501	-3.37399	0.001437	0.037626	-1.32392
ILMN_1893633	0.078527	6.969511	3.474988	0.001064	0.031169	-1.04761
ILMN_1662905	0.078507	12.26727	3.749181	0.000461	0.019441	-0.27369
ILMN_2083243	0.078502	8.487235	4.251653	9.25E-05	0.007221	1.223716
ILMN_2246894	-0.07849	10.12191	-3.69036	0.000553	0.021556	-0.44251
ILMN_2414007	0.078437	6.757321	4.019993	0.000196	0.011957	0.521734
ILMN_1755758	0.078412	6.846843	3.333351	0.00162	0.040674	-1.43366
ILMN_1653822	0.078242	6.814945	4.02839	0.000191	0.011723	0.546847
ILMN_3306388	0.078231	6.678257	4.010053	0.000202	0.012129	0.492039
ILMN_1668657	0.078209	6.905298	4.59906	2.91E-05	0.003489	2.308447
ILMN_2182531	0.078112	9.98976	3.762551	0.000442	0.018974	-0.23511
ILMN_1721977	0.078102	8.948922	3.86279	0.000323	0.01604	0.056475
ILMN_1700232	0.078096	8.155792	3.928519	0.000262	0.014219	0.249853
ILMN_1653836	-0.07809	6.280264	-3.72412	0.000498	0.020244	-0.34579
ILMN_1842850	-0.07808	6.399931	-3.50399	0.000975	0.029813	-0.96736
ILMN_2221673	0.078025	9.902959	3.240027	0.002126	0.048536	-1.68253
ILMN_1795865	-0.07799	7.208199	-3.64819	0.000629	0.023404	-0.56263
ILMN_2406501	0.077905	7.147169	3.50994	0.000958	0.029485	-0.95086
ILMN_1811574	-0.07789	8.301684	-3.27289	0.001933	0.045678	-1.59542
ILMN_1755910	-0.07783	6.575995	-3.83266	0.000355	0.01681	-0.0316
ILMN_3255144	0.077718	6.471177	3.61538	0.000696	0.024558	-0.65555
ILMN_2286024	0.077714	5.747321	5.488709	1.34E-06	0.000565	5.216214
ILMN_1736176	0.077657	7.014995	3.85363	0.000332	0.016239	0.02966
ILMN_1798940	0.077607	6.881157	4.087774	0.000158	0.010155	0.725188
ILMN_2113362	0.0775	6.918771	3.585963	0.000761	0.025703	-0.73845
ILMN_3234884	0.077488	8.666052	3.378191	0.00142	0.037331	-1.31251
ILMN_1702384	-0.07739	7.422851	-3.2846	0.001868	0.044572	-1.56424
ILMN_2050617	0.077363	10.63245	3.231026	0.002182	0.049513	-1.7063
ILMN_1753265	-0.07733	6.468278	-3.64727	0.000631	0.023445	-0.56524
ILMN_2352934	0.07731	6.588773	3.63185	0.000662	0.02392	-0.60897
ILMN_2181992	-0.07729	6.769124	-3.39566	0.001348	0.036018	-1.26503
ILMN_1688848	-0.07717	6.092466	-3.32156	0.001677	0.04148	-1.46535
ILMN_1720965	-0.07713	7.884281	-3.28967	0.001841	0.044165	-1.55071
ILMN_1805148	-0.07703	6.428121	-4.53149	3.66E-05	0.00394	2.09476
ILMN_1813246	0.077022	9.378718	3.28526	0.001865	0.04455	-1.56247
ILMN_1798543	0.076998	7.458454	3.615543	0.000695	0.024558	-0.65509
ILMN_1723843	-0.07696	8.337517	-3.97432	0.000227	0.013051	0.385604
ILMN_1755620	-0.07688	6.813174	-3.41062	0.00129	0.035117	-1.22428
ILMN_1698015	-0.07683	6.189212	-4.16998	0.000121	0.008445	0.974109

ILMN_1655952	-0.07679	9.16614	-3.40274	0.00132	0.035549	-1.24577
ILMN_1749243	0.076612	7.439673	3.620291	0.000685	0.024488	-0.64167
ILMN_1653283	-0.07654	6.972005	-3.29923	0.00179	0.043403	-1.52517
ILMN_2162799	0.076527	7.112451	4.571098	3.20E-05	0.003708	2.219862
ILMN_1775823	-0.0764	7.617423	-3.29532	0.001811	0.04375	-1.53562
ILMN_1856634	0.076278	6.904764	3.388604	0.001377	0.036444	-1.28423
ILMN_1794122	-0.07625	5.774116	-4.19694	0.000111	0.007879	1.05627
ILMN_1782633	0.076245	10.44958	3.779809	0.000419	0.018393	-0.1852
ILMN_1663042	-0.07605	9.702589	-3.28277	0.001878	0.044661	-1.5691
ILMN_1700378	0.075958	7.794192	3.401631	0.001324	0.035568	-1.24878
ILMN_1772743	0.075933	8.542248	3.466927	0.00109	0.031665	-1.06985
ILMN_1760933	0.075921	7.729166	3.706151	0.000526	0.020981	-0.39734
ILMN_1662417	0.075882	8.315637	4.021399	0.000195	0.011925	0.525937
ILMN_2389528	0.07578	6.108071	4.519325	3.81E-05	0.004029	2.056405
ILMN_1782377	0.075657	8.15753	4.111194	0.000146	0.009669	0.795867
ILMN_1686835	0.075618	6.816222	3.564635	0.000812	0.026563	-0.79831
ILMN_3247906	-0.07555	8.076963	-3.6052	0.000718	0.024926	-0.68429
ILMN_2411794	-0.07554	8.508565	-3.86833	0.000317	0.015893	0.072697
ILMN_3268403	-0.07553	5.923422	-3.82454	0.000364	0.017034	-0.05527
ILMN_3242462	0.075357	6.881228	3.796151	0.000398	0.017959	-0.13783
ILMN_3238452	-0.07516	7.509117	-4.23607	9.73E-05	0.00731	1.175915
ILMN_2397571	0.075105	6.875508	3.262733	0.001991	0.046662	-1.6224
ILMN_1672547	-0.07507	7.394584	-3.39409	0.001354	0.03607	-1.2693
ILMN_2410742	-0.075	8.216274	-3.48709	0.001026	0.030581	-1.01418
ILMN_1758827	0.074967	7.92998	3.889707	0.000297	0.015308	0.13546
ILMN_1724333	-0.07495	7.319627	-4.25663	9.10E-05	0.007161	1.239001
ILMN_1678454	0.074936	9.454743	3.259581	0.002009	0.046877	-1.63076
ILMN_1729868	0.074827	6.96272	4.090434	0.000156	0.010087	0.733206
ILMN_1783337	0.074779	6.847807	3.672166	0.000585	0.022356	-0.49443
ILMN_2224990	0.074776	6.33814	4.000827	0.000208	0.012321	0.464508
ILMN_2191313	-0.07467	6.159919	-4.22676	0.0001	0.007349	1.147393
ILMN_1780292	0.074581	6.06516	4.514634	3.87E-05	0.004079	2.041629
ILMN_1771903	0.074547	10.2093	3.666718	0.000594	0.022547	-0.50995
ILMN_2377430	-0.07448	7.648643	-4.00432	0.000206	0.012278	0.474927
ILMN_1708029	-0.07443	5.796382	-3.73598	0.00048	0.01977	-0.3117
ILMN_1695962	-0.07437	7.735068	-3.87548	0.00031	0.01567	0.093666
ILMN_1797499	0.074313	7.334307	3.368553	0.001461	0.038118	-1.33864
ILMN_1662364	0.074304	9.771747	3.484838	0.001033	0.03075	-1.0204
ILMN_1721034	-0.07426	6.996372	-3.66974	0.000589	0.022452	-0.50134
ILMN_1691131	0.074258	8.694918	3.854193	0.000332	0.016239	0.031306
ILMN_1662161	-0.07413	6.398688	-3.79196	0.000403	0.018034	-0.14999
ILMN_3289262	0.074085	9.609777	3.998395	0.00021	0.012375	0.457257
ILMN_1778611	0.074009	8.430149	3.564996	0.000811	0.026563	-0.79729
ILMN_1765923	0.073932	6.748245	3.806041	0.000386	0.017643	-0.1091
ILMN_1849941	-0.07391	6.628758	-3.42869	0.001222	0.033786	-1.17487
ILMN_1790461	0.073742	10.46785	3.590287	0.000751	0.025581	-0.72629
ILMN_1710979	0.073673	7.122279	3.725241	0.000496	0.020222	-0.34258
ILMN_1810838	0.073652	10.17688	3.945015	0.000249	0.013644	0.298648
ILMN_1772706	-0.07358	7.77093	-3.26154	0.001998	0.046728	-1.62557
ILMN_1761858	-0.07347	8.379168	-3.51936	0.000931	0.02902	-0.92467
ILMN_1654861	-0.07345	7.784018	-3.29967	0.001788	0.043394	-1.524
ILMN_1789627	-0.07337	6.247496	-3.61834	0.00069	0.024533	-0.64719
ILMN_1774990	0.073274	8.209856	3.349791	0.001544	0.03952	-1.38936
ILMN_1682996	0.073103	6.480388	3.505524	0.000971	0.029728	-0.96311

ILMN_1662184	0.073051	6.902236	3.419456	0.001256	0.034507	-1.20014
ILMN_1726512	-0.07297	5.750646	-3.63339	0.000659	0.02392	-0.60461
ILMN_1807074	0.072952	12.4116	3.988597	0.000217	0.012682	0.428063
ILMN_1697544	-0.07286	6.54915	-3.45162	0.001141	0.032558	-1.11197
ILMN_1781257	0.072847	7.109016	3.665912	0.000596	0.022553	-0.51225
ILMN_1814247	-0.07284	8.133792	-3.65235	0.000621	0.023182	-0.55081
ILMN_1748916	0.07278	9.077889	4.057512	0.000174	0.010831	0.634146
ILMN_1758895	-0.07277	5.959237	-3.43292	0.001207	0.033515	-1.16328
ILMN_1707534	0.072741	7.520501	5.668298	7.11E-07	0.000373	5.819039
ILMN_3236239	-0.07266	6.713216	-3.37689	0.001425	0.03743	-1.31605
ILMN_1655654	0.072525	7.681589	3.542391	0.000869	0.027756	-0.86051
ILMN_1727479	-0.07248	9.198404	-3.54618	0.000859	0.027517	-0.84994
ILMN_1667222	0.072463	7.845548	3.247599	0.00208	0.047878	-1.66251
ILMN_1765644	0.072454	9.64981	4.001319	0.000208	0.012321	0.465976
ILMN_1783695	0.072385	10.06995	4.106225	0.000148	0.009806	0.780855
ILMN_1777483	0.072325	6.793192	4.63971	2.54E-05	0.003118	2.437595
ILMN_1676233	0.072315	6.56562	4.126677	0.000139	0.009321	0.842699
ILMN_1794781	-0.07223	6.908979	-4.00409	0.000206	0.012278	0.474228
ILMN_1677440	-0.07222	10.45174	-3.53898	0.000878	0.027941	-0.87004
ILMN_1660579	0.072202	7.033727	3.393951	0.001355	0.03607	-1.26969
ILMN_1769634	0.072184	9.424811	3.463424	0.001102	0.031813	-1.0795
ILMN_1720442	-0.07213	9.711777	-3.7334	0.000484	0.019881	-0.31911
ILMN_1787199	0.072111	5.75609	3.38485	0.001392	0.036743	-1.29443
ILMN_1734553	-0.07207	6.505856	-4.48471	4.28E-05	0.004415	1.947528
ILMN_1666206	-0.07207	6.701845	-3.38839	0.001377	0.036444	-1.28482
ILMN_2375557	0.071972	6.017795	3.652389	0.000621	0.023182	-0.55071
ILMN_2061452	0.071961	8.406673	3.83644	0.000351	0.016681	-0.02057
ILMN_1708345	0.071837	6.267427	3.283498	0.001874	0.044656	-1.56716
ILMN_1815479	0.071832	11.76915	3.62177	0.000682	0.024479	-0.63749
ILMN_1694799	0.071807	7.960521	3.278044	0.001904	0.045121	-1.58169
ILMN_1761262	0.071738	6.502508	3.477031	0.001058	0.031087	-1.04197
ILMN_1740319	-0.07173	8.118393	-3.26573	0.001974	0.046396	-1.61445
ILMN_1700975	0.071665	5.799969	3.894653	0.000292	0.015163	0.150006
ILMN_1741148	0.07157	11.2415	4.231465	9.88E-05	0.007317	1.161817
ILMN_2361807	-0.07156	6.954242	-3.4425	0.001173	0.033169	-1.13703
ILMN_1711543	0.071485	7.71451	3.510306	0.000957	0.029485	-0.94984
ILMN_1709623	-0.07135	6.769465	-3.90905	0.000279	0.014731	0.192388
ILMN_1793146	0.071344	7.836729	3.389288	0.001374	0.036444	-1.28237
ILMN_1804479	0.071343	10.40331	4.210683	0.000106	0.007599	1.098234
ILMN_1680659	-0.07133	6.070394	-3.33364	0.001619	0.040673	-1.43287
ILMN_1783973	-0.07126	6.213538	-3.80345	0.000389	0.017716	-0.11664
ILMN_1746252	0.07119	10.81671	3.477606	0.001056	0.031069	-1.04039
ILMN_1654690	0.071123	8.92939	3.364143	0.00148	0.038388	-1.35057
ILMN_1727761	0.07107	6.848968	3.750506	0.000459	0.019384	-0.26986
ILMN_1748831	-0.07104	7.036402	-3.69443	0.000546	0.021487	-0.43087
ILMN_1793966	0.070915	6.391729	3.796669	0.000397	0.017953	-0.13632
ILMN_1716400	0.070903	6.972341	3.388638	0.001376	0.036444	-1.28414
ILMN_1706839	0.070831	8.051776	3.690384	0.000553	0.021556	-0.44245
ILMN_1651936	-0.0708	8.362008	-3.52449	0.000917	0.02876	-0.91041
ILMN_2278819	0.070792	6.91849	3.577364	0.000781	0.025949	-0.76261
ILMN_2130838	0.07077	9.563752	3.286953	0.001855	0.044454	-1.55795
ILMN_1698846	-0.07077	5.446632	-3.27662	0.001912	0.045277	-1.58548
ILMN_1739283	0.070708	8.105886	3.570973	0.000797	0.026314	-0.78054
ILMN_1769849	0.070592	5.956987	3.96614	0.000233	0.013176	0.361289

ILMN_1765459	-0.07059	9.787609	-3.38581	0.001388	0.036668	-1.29184
ILMN_1808163	0.070583	6.654041	4.237847	9.68E-05	0.007301	1.181371
ILMN_2194627	0.070511	7.630318	3.451487	0.001142	0.032558	-1.11234
ILMN_1760714	0.070499	13.40672	3.933565	0.000258	0.014038	0.264768
ILMN_1775742	0.07043	6.149598	3.308378	0.001743	0.042537	-1.5007
ILMN_1756220	0.070272	9.88412	3.919346	0.00027	0.014439	0.222763
ILMN_1696503	0.070166	5.995005	3.341055	0.001584	0.040178	-1.41292
ILMN_3238797	0.070108	6.616323	3.988046	0.000217	0.012683	0.426422
ILMN_1700628	0.070023	8.914394	3.496834	0.000997	0.03026	-0.98721
ILMN_3237241	-0.0699	8.244827	-3.67852	0.000573	0.022063	-0.4763
ILMN_1712639	-0.06987	5.972284	-3.6175	0.000691	0.024553	-0.64955
ILMN_3235477	0.069853	7.23899	3.310182	0.001734	0.042424	-1.49586
ILMN_1766010	0.069786	9.713606	3.406058	0.001307	0.0354	-1.23672
ILMN_1761722	-0.06965	6.721795	-3.43633	0.001195	0.033496	-1.15394
ILMN_1737205	0.069609	10.47446	3.328387	0.001644	0.040977	-1.44701
ILMN_2396947	0.069554	6.802564	3.312092	0.001724	0.042307	-1.49075
ILMN_2095133	-0.06951	8.352404	-4.01346	0.0002	0.01204	0.50221
ILMN_1769503	0.06949	5.950628	4.165079	0.000123	0.008545	0.959216
ILMN_1755710	-0.06945	6.44396	-3.58004	0.000775	0.025861	-0.75508
ILMN_3276859	0.06936	6.856227	3.36189	0.00149	0.038554	-1.35667
ILMN_1734895	0.069347	10.45557	3.969444	0.00023	0.013128	0.371101
ILMN_1713732	-0.06931	7.408825	-3.56285	0.000816	0.02668	-0.80331
ILMN_2407168	-0.0692	9.364091	-3.24467	0.002098	0.048112	-1.67024
ILMN_1774427	-0.06903	6.664536	-3.31107	0.001729	0.042373	-1.49347
ILMN_2400583	-0.06898	6.221054	-3.69094	0.000552	0.021556	-0.44086
ILMN_1722798	-0.06897	6.920934	-3.57944	0.000776	0.025861	-0.75678
ILMN_3240365	-0.06896	6.127374	-3.48836	0.001022	0.030581	-1.01066
ILMN_1662232	-0.06891	10.57958	-3.61523	0.000696	0.024558	-0.65598
ILMN_2414366	0.06864	6.52836	4.147882	0.00013	0.008928	0.906976
ILMN_1740197	-0.06863	6.46239	-3.8535	0.000332	0.016239	0.029292
ILMN_1700549	0.068579	6.885617	3.923691	0.000266	0.014374	0.235592
ILMN_1757956	-0.06847	7.737848	-3.22631	0.002212	0.049948	-1.71873
ILMN_1677953	0.068394	8.517315	3.228722	0.002197	0.049732	-1.71238
ILMN_1733511	-0.06836	9.79902	-3.58231	0.00077	0.025798	-0.74872
ILMN_3251567	-0.0683	6.775704	-3.51746	0.000937	0.029134	-0.92996
ILMN_1660186	-0.06827	8.751922	-3.44386	0.001168	0.033144	-1.13328
ILMN_1686668	0.068208	7.342006	4.232814	9.84E-05	0.00731	1.165949
ILMN_1706571	-0.06811	6.719259	-3.3518	0.001534	0.039392	-1.38394
ILMN_1693559	-0.06805	6.12679	-3.73697	0.000478	0.019733	-0.30884
ILMN_3249963	0.068032	6.239203	3.490496	0.001016	0.030533	-1.00476
ILMN_1724009	0.068014	7.799939	3.790907	0.000405	0.018066	-0.15304
ILMN_1755235	0.068013	8.331261	3.899223	0.000288	0.015035	0.163454
ILMN_1746368	-0.06797	7.297648	-4.05503	0.000175	0.010898	0.626681
ILMN_1752027	-0.06787	7.845224	-3.32551	0.001658	0.041235	-1.45475
ILMN_1748481	0.067857	7.970756	3.743123	0.000469	0.019597	-0.29114
ILMN_2415949	-0.0678	8.011595	-3.56137	0.00082	0.026726	-0.80746
ILMN_2182198	0.067751	9.117461	3.960582	0.000237	0.01328	0.344791
ILMN_1678546	-0.06772	9.135787	-3.78208	0.000416	0.018352	-0.17862
ILMN_1805992	-0.06768	9.205907	-3.90573	0.000282	0.014802	0.182629
ILMN_1694486	0.067608	6.609586	3.911816	0.000277	0.014692	0.200552
ILMN_1651504	-0.06758	7.020903	-3.51676	0.000939	0.02914	-0.93191
ILMN_1722953	-0.0675	6.095272	-3.72586	0.000495	0.020207	-0.3408
ILMN_1770978	0.067482	5.619437	3.227474	0.002205	0.049867	-1.71566
ILMN_1880012	-0.06734	6.180205	-4.02192	0.000195	0.011925	0.527483

ILMN_2387599	-0.06725	11.77284	-3.28037	0.001891	0.044909	-1.5755
ILMN_2285112	-0.06724	9.45944	-3.76572	0.000438	0.018882	-0.22597
ILMN_1702124	-0.06722	8.13866	-4.21817	0.000103	0.007459	1.121123
ILMN_1875342	-0.06722	7.489629	-4.26255	8.92E-05	0.007073	1.257193
ILMN_1669635	0.067171	9.458683	4.201346	0.000109	0.007798	1.069713
ILMN_1800958	0.066951	9.531794	3.97515	0.000226	0.013051	0.388056
ILMN_1787477	0.066855	6.784701	3.519178	0.000932	0.02902	-0.92519
ILMN_1736863	-0.06672	5.676027	-3.54181	0.00087	0.02778	-0.86214
ILMN_1670796	0.066715	9.651498	3.921822	0.000268	0.014389	0.230073
ILMN_2322935	0.066679	8.922015	3.851064	0.000335	0.016288	0.022156
ILMN_1742922	0.066633	8.151503	3.68717	0.000558	0.02172	-0.45163
ILMN_2298511	0.06663	8.457234	3.438549	0.001187	0.033343	-1.14787
ILMN_1678268	-0.06659	7.226246	-3.96163	0.000236	0.01326	0.347898
ILMN_3241985	0.066558	6.365159	3.784659	0.000413	0.018281	-0.17115
ILMN_1665065	-0.06653	6.807666	-3.77027	0.000431	0.018733	-0.2128
ILMN_1792489	0.066525	12.35525	4.008216	0.000203	0.01218	0.486554
ILMN_1809086	-0.06652	7.998576	-3.79433	0.0004	0.01801	-0.14311
ILMN_1767541	0.066478	6.062374	3.810671	0.00038	0.017435	-0.09565
ILMN_1669772	-0.06646	5.496418	-4.31398	7.54E-05	0.006438	1.41566
ILMN_1750178	-0.06644	7.241327	-3.67758	0.000575	0.022073	-0.47899
ILMN_1773148	-0.0663	6.855625	-3.35731	0.00151	0.038903	-1.36906
ILMN_2402806	-0.06626	8.329239	-3.24758	0.00208	0.047878	-1.66255
ILMN_1687546	-0.06626	6.590849	-3.33123	0.00163	0.040776	-1.43937
ILMN_2407308	-0.0662	6.087804	-3.71324	0.000515	0.020766	-0.37702
ILMN_1713875	0.066194	6.984571	3.314247	0.001713	0.042131	-1.48497
ILMN_1674128	0.066169	7.295817	4.083939	0.00016	0.010205	0.713632
ILMN_1789732	-0.06612	7.190357	-3.80275	0.00039	0.017731	-0.11867
ILMN_1770732	0.066019	10.41735	3.259167	0.002012	0.046877	-1.63186
ILMN_1738819	0.065922	9.110635	3.469998	0.00108	0.031455	-1.06138
ILMN_3248575	0.065908	6.366202	4.380921	6.04E-05	0.005614	1.623154
ILMN_1745954	0.065766	9.440604	3.529841	0.000902	0.028408	-0.89551
ILMN_3188099	0.065714	7.34998	3.863033	0.000323	0.01604	0.057186
ILMN_2046024	0.065683	7.334315	3.916187	0.000273	0.014527	0.213442
ILMN_1744914	0.065605	7.926975	3.690574	0.000552	0.021556	-0.4419
ILMN_1795893	-0.06557	7.453639	-4.14633	0.00013	0.00893	0.902257
ILMN_1736340	-0.06557	8.446269	-4.08618	0.000158	0.010162	0.72038
ILMN_1659156	0.065536	6.381475	4.229499	9.95E-05	0.007326	1.155795
ILMN_1665554	-0.06547	6.731716	-3.48731	0.001026	0.030581	-1.01358
ILMN_2163790	-0.06546	5.714307	-3.30814	0.001744	0.042537	-1.50134
ILMN_2069821	0.065419	6.303886	3.88202	0.000304	0.015453	0.112876
ILMN_1779184	0.065413	6.575723	4.06933	0.000167	0.01058	0.669659
ILMN_1700896	0.065364	8.699259	3.769369	0.000433	0.018762	-0.21541
ILMN_1740165	-0.06524	7.584426	-3.3791	0.001416	0.03726	-1.31006
ILMN_1691333	-0.06519	6.389422	-3.63152	0.000662	0.02392	-0.60989
ILMN_1799488	-0.06498	6.664421	-3.59818	0.000733	0.025232	-0.70407
ILMN_1663493	0.064712	7.638221	3.294173	0.001817	0.043802	-1.53868
ILMN_1698365	0.064513	5.905556	3.507773	0.000964	0.029554	-0.95687
ILMN_1810474	0.064498	10.47726	3.70732	0.000525	0.020981	-0.39399
ILMN_2369682	0.064426	12.64903	3.854843	0.000331	0.016239	0.033209
ILMN_2231020	0.064348	8.699813	3.269324	0.001953	0.04609	-1.60489
ILMN_2139827	0.064279	6.49442	3.320888	0.00168	0.04148	-1.46715
ILMN_1666332	-0.06426	6.90698	-3.33724	0.001602	0.040464	-1.42319
ILMN_2319544	0.064242	6.139729	3.463722	0.001101	0.031813	-1.07868
ILMN_1654598	-0.06423	6.784462	-3.73085	0.000488	0.019993	-0.32647

ILMN_1747162	0.064149	9.333039	3.329654	0.001638	0.040884	-1.4436
ILMN_1665601	0.064001	7.153769	3.683017	0.000565	0.021901	-0.46348
ILMN_1774281	0.063911	6.209842	3.804382	0.000388	0.017711	-0.11392
ILMN_1670377	-0.06375	6.558711	-3.34428	0.001569	0.039976	-1.40423
ILMN_1791896	0.06369	9.982601	3.420645	0.001252	0.034412	-1.19689
ILMN_1714461	-0.06369	8.581909	-3.58855	0.000755	0.025654	-0.73117
ILMN_1764398	0.063515	6.95288	4.115434	0.000144	0.009592	0.808682
ILMN_1695917	-0.06342	9.13386	-3.5658	0.000809	0.026563	-0.79505
ILMN_1785177	0.063391	7.1096	3.292554	0.001825	0.043917	-1.54301
ILMN_1791302	0.063347	5.413727	4.132604	0.000136	0.009198	0.860651
ILMN_1689720	0.063267	6.837395	3.402436	0.001321	0.035549	-1.24659
ILMN_1658830	-0.06324	7.297197	-3.34922	0.001546	0.039545	-1.39091
ILMN_1787308	-0.0632	7.499074	-3.35696	0.001511	0.038913	-1.37
ILMN_1741564	-0.0632	8.096878	-3.54332	0.000866	0.027705	-0.85793
ILMN_1709162	0.063189	6.238882	3.590109	0.000752	0.025581	-0.72679
ILMN_1788377	-0.06318	5.678743	-3.43235	0.001209	0.033515	-1.16486
ILMN_1699082	0.062966	7.642711	3.961959	0.000236	0.01326	0.348878
ILMN_3188196	0.062908	5.65451	3.255248	0.002035	0.047268	-1.64225
ILMN_1679731	0.062822	7.707593	3.823294	0.000366	0.017054	-0.0589
ILMN_2090786	0.062746	6.199522	3.548545	0.000853	0.027472	-0.84333
ILMN_3262936	0.062673	7.718663	3.832143	0.000356	0.01681	-0.03311
ILMN_1670532	0.062658	7.726652	3.513801	0.000947	0.029309	-0.94013
ILMN_1776347	0.062628	11.11013	3.591046	0.000749	0.025581	-0.72415
ILMN_1720838	-0.06259	10.69731	-3.45741	0.001122	0.032173	-1.09605
ILMN_1673944	-0.06213	10.05075	-3.63269	0.00066	0.02392	-0.60658
ILMN_1781099	-0.06211	8.614731	-3.46597	0.001093	0.031729	-1.07248
ILMN_1670440	0.061995	5.638456	3.609197	0.000709	0.024854	-0.67301
ILMN_1705266	0.061939	7.110534	3.636466	0.000652	0.023869	-0.59589
ILMN_1805606	-0.06186	6.257124	-3.51159	0.000953	0.029425	-0.94628
ILMN_1750511	0.061781	7.313128	3.434558	0.001201	0.033496	-1.15881
ILMN_1667260	0.061702	8.447507	3.299543	0.001789	0.043394	-1.52433
ILMN_1667580	0.061651	6.044111	3.469596	0.001082	0.031466	-1.06249
ILMN_1787541	-0.06164	6.610779	-3.23057	0.002185	0.049532	-1.70749
ILMN_1814589	0.061504	7.199963	3.403528	0.001317	0.035527	-1.24361
ILMN_2230672	0.061464	10.81509	4.241311	9.57E-05	0.007252	1.191988
ILMN_2075892	0.061406	5.774836	3.616828	0.000693	0.024558	-0.65146
ILMN_1817545	-0.06114	6.080008	-3.37456	0.001435	0.037591	-1.32237
ILMN_1730660	-0.06106	6.511666	-3.60644	0.000715	0.024876	-0.68078
ILMN_1830985	-0.06098	5.98513	-3.26616	0.001971	0.046388	-1.61329
ILMN_1700238	-0.06096	6.866117	-3.29526	0.001811	0.04375	-1.53577
ILMN_2372698	-0.06093	6.023205	-3.64363	0.000638	0.023582	-0.57559
ILMN_3248057	-0.06093	6.076667	-4.69511	2.11E-05	0.002859	2.614286
ILMN_1744147	0.06092	10.12962	3.558384	0.000828	0.026893	-0.81581
ILMN_1739164	0.060837	5.841739	3.640808	0.000644	0.023685	-0.58358
ILMN_2357382	0.060778	6.740875	3.538402	0.000879	0.027964	-0.87165
ILMN_1737413	0.060707	7.554731	3.235142	0.002157	0.049076	-1.69544
ILMN_3203098	0.060493	6.27157	3.56542	0.00081	0.026563	-0.79611
ILMN_2038772	-0.06038	11.82935	-3.5371	0.000883	0.028023	-0.87528
ILMN_1813641	-0.06028	7.114735	-3.26189	0.001996	0.046713	-1.62464
ILMN_1674658	0.06016	5.345732	3.40227	0.001322	0.035549	-1.24704
ILMN_2354649	0.060028	7.613953	3.801668	0.000391	0.017767	-0.12181
ILMN_1675542	0.05991	9.312797	3.514212	0.000946	0.029299	-0.93899
ILMN_1656378	0.059787	7.057486	3.34782	0.001553	0.039678	-1.39468
ILMN_1750092	-0.0597	6.27336	-3.41801	0.001262	0.034602	-1.2041

ILMN_3242638	0.059596	6.533512	3.669249	0.00059	0.022452	-0.50274
ILMN_1672390	0.059505	6.340332	3.618653	0.000689	0.024533	-0.6463
ILMN_3234735	0.059402	6.842951	4.158921	0.000125	0.008701	0.940497
ILMN_1674908	-0.05909	6.39054	-3.65721	0.000612	0.022963	-0.537
ILMN_1786426	0.059071	6.398607	3.572366	0.000793	0.02627	-0.77663
ILMN_1756352	-0.05902	8.736713	-3.61498	0.000697	0.024558	-0.65667
ILMN_1668408	0.059008	6.832079	3.435293	0.001198	0.033496	-1.15679
ILMN_1747629	0.058983	5.737485	3.383939	0.001396	0.036814	-1.29691
ILMN_1837298	0.058946	6.160308	3.534183	0.000891	0.028141	-0.88341
ILMN_1679782	-0.05892	6.257648	-3.95873	0.000238	0.013337	0.33929
ILMN_2377733	0.05887	6.007639	3.362009	0.001489	0.038554	-1.35635
ILMN_3239162	-0.05884	5.864807	-3.32469	0.001662	0.041274	-1.45693
ILMN_1688753	0.058734	10.48571	3.456392	0.001125	0.032201	-1.09885
ILMN_1737144	0.058718	5.966924	3.98237	0.000221	0.012893	0.40953
ILMN_1785107	0.058678	7.490235	3.523131	0.000921	0.02878	-0.91419
ILMN_3247222	0.058449	5.894238	3.441937	0.001175	0.03317	-1.13857
ILMN_1776147	0.058319	8.433	3.717791	0.000508	0.020572	-0.36396
ILMN_1721337	0.058212	7.576043	3.784639	0.000413	0.018281	-0.17121
ILMN_1742962	0.058205	6.073157	3.405197	0.00131	0.035435	-1.23906
ILMN_1899940	0.058004	6.250459	3.591477	0.000748	0.025573	-0.72294
ILMN_1759789	0.057898	6.664458	3.341057	0.001584	0.040178	-1.41291
ILMN_1761474	0.057796	7.49856	3.58215	0.00077	0.025798	-0.74916
ILMN_1811301	-0.05768	7.229325	-3.40541	0.00131	0.035435	-1.23847
ILMN_2255310	0.057612	11.02611	3.819959	0.000369	0.017174	-0.06862
ILMN_3246944	0.057554	6.035575	3.665058	0.000598	0.022588	-0.51468
ILMN_3248562	0.057426	6.903549	3.459268	0.001115	0.032048	-1.09094
ILMN_1836218	0.057407	7.240125	3.357336	0.00151	0.038903	-1.36898
ILMN_1743131	-0.05733	7.581142	-3.68259	0.000566	0.021905	-0.4647
ILMN_1746893	0.057004	6.199842	3.305168	0.001759	0.042807	-1.50929
ILMN_3309754	-0.05694	5.824769	-3.29326	0.001822	0.043875	-1.54113
ILMN_1680403	-0.05694	10.4819	-3.23572	0.002153	0.049059	-1.69391
ILMN_1739876	-0.05694	9.891474	-3.59945	0.00073	0.025207	-0.70048
ILMN_1809883	0.05694	5.617203	3.793456	0.000401	0.018016	-0.14565
ILMN_1761721	0.056727	10.42	3.290282	0.001838	0.044148	-1.54907
ILMN_1737805	0.056591	5.529744	3.250966	0.00206	0.047634	-1.65359
ILMN_1676159	0.056459	9.37641	3.465393	0.001095	0.031757	-1.07407
ILMN_2105573	0.056319	6.023866	3.548289	0.000853	0.027472	-0.84404
ILMN_1748607	0.055987	5.781067	3.910426	0.000278	0.014712	0.196453
ILMN_1693843	0.055789	5.993364	3.760444	0.000445	0.019051	-0.24119
ILMN_1724504	0.055783	9.781237	3.566191	0.000808	0.026563	-0.79395
ILMN_3309869	0.05471	5.800399	3.860619	0.000325	0.016125	0.050115
ILMN_1771815	-0.05455	10.19336	-3.32116	0.001679	0.04148	-1.46642
ILMN_2395092	0.054458	5.953096	3.570663	0.000797	0.026314	-0.78141
ILMN_1654365	0.054374	5.770407	3.668802	0.000591	0.022452	-0.50402
ILMN_2247296	-0.05434	5.921464	-4.24445	9.47E-05	0.007245	1.201616
ILMN_1873261	-0.05433	5.92997	-3.30795	0.001745	0.042537	-1.50183
ILMN_1701940	-0.05432	7.001941	-3.26515	0.001977	0.046399	-1.61599
ILMN_1703949	0.054319	11.56885	3.254818	0.002037	0.047295	-1.64339
ILMN_1656427	-0.05427	6.98163	-3.41742	0.001264	0.034633	-1.2057
ILMN_1810608	0.054159	6.838954	3.437756	0.00119	0.033395	-1.15004
ILMN_1801905	-0.05416	6.675453	-3.25844	0.002016	0.046928	-1.63379
ILMN_1655052	0.054124	6.102045	3.365268	0.001475	0.038315	-1.34753
ILMN_3298462	0.053894	5.557262	4.237946	9.67E-05	0.007301	1.181674
ILMN_2173740	-0.05387	7.362773	-3.2411	0.00212	0.048485	-1.67968

ILMN_1738497	-0.0538	5.905921	-3.44157	0.001176	0.03317	-1.13958
ILMN_1765532	0.053798	7.495265	3.326105	0.001655	0.041192	-1.45314
ILMN_1749212	-0.05379	7.075685	-3.42347	0.001241	0.034179	-1.18918
ILMN_1806122	-0.05369	8.218411	-3.26197	0.001995	0.046713	-1.62441
ILMN_1738656	0.053567	9.319114	4.344388	6.82E-05	0.006106	1.50974
ILMN_3310401	-0.05348	5.717793	-3.4778	0.001055	0.031069	-1.03985
ILMN_2308689	0.053456	7.681956	3.341467	0.001582	0.040178	-1.41181
ILMN_2049642	0.053413	10.4411	3.793976	0.000401	0.01801	-0.14414
ILMN_1680682	0.053383	6.391218	3.503379	0.000977	0.029815	-0.96907
ILMN_3240117	-0.05315	10.03713	-3.87388	0.000312	0.015707	0.088973
ILMN_1719857	-0.05309	5.749521	-3.24832	0.002076	0.04784	-1.6606
ILMN_1808132	0.052797	6.072595	3.704148	0.00053	0.021039	-0.40307
ILMN_1739454	0.052762	7.317376	3.938978	0.000254	0.013842	0.28078
ILMN_1755911	0.052438	6.392163	4.003544	0.000207	0.012278	0.472613
ILMN_2221336	0.051708	6.184475	3.607915	0.000712	0.024854	-0.67662
ILMN_2187746	0.051644	6.165204	3.36806	0.001463	0.038145	-1.33997
ILMN_2044645	-0.05159	6.215112	-3.37913	0.001416	0.03726	-1.30996
ILMN_1663024	0.051512	5.277935	4.388275	5.90E-05	0.005509	1.646034
ILMN_2065299	-0.05151	7.599916	-3.27362	0.001929	0.045612	-1.59347
ILMN_2311873	-0.05126	5.907227	-3.58319	0.000768	0.025767	-0.74625
ILMN_1907966	0.051222	6.077735	3.613084	0.000701	0.024636	-0.66203
ILMN_1656327	-0.05113	5.591198	-3.43496	0.001199	0.033496	-1.1577
ILMN_1757825	-0.05106	5.640296	-3.31474	0.001711	0.042101	-1.48366
ILMN_1683798	0.051004	6.135779	3.837325	0.00035	0.016658	-0.01799
ILMN_1861270	-0.05097	6.065112	-3.40706	0.001303	0.03535	-1.23399
ILMN_2048477	-0.05061	5.872809	-3.25684	0.002025	0.047115	-1.63804
ILMN_1801572	-0.05051	5.635066	-3.87846	0.000307	0.015581	0.102431
ILMN_1766247	0.049839	6.790934	3.477519	0.001056	0.031069	-1.04063
ILMN_1714527	-0.04968	9.882828	-3.42876	0.001222	0.033786	-1.17469
ILMN_1794987	0.049666	6.293463	3.48748	0.001025	0.030581	-1.0131
ILMN_2415572	-0.0494	5.916209	-3.38746	0.001381	0.036517	-1.28734
ILMN_1777060	0.049396	6.197064	3.493753	0.001006	0.030395	-0.99574
ILMN_1662021	0.049054	6.452101	3.38989	0.001371	0.036422	-1.28074
ILMN_2261099	0.048482	5.366453	3.412996	0.00128	0.034925	-1.21779
ILMN_1776325	-0.04844	7.337166	-3.43524	0.001198	0.033496	-1.15693
ILMN_3248069	-0.04828	13.81881	-3.51549	0.000942	0.029212	-0.93544
ILMN_3235148	0.048138	12.98194	3.29506	0.001812	0.04375	-1.53631
ILMN_1748407	0.048123	6.545728	3.332647	0.001623	0.040674	-1.43556
ILMN_1802766	0.048106	6.196487	3.496112	0.000999	0.03026	-0.98921
ILMN_1787193	-0.048	5.464581	-3.39399	0.001355	0.03607	-1.26958
ILMN_2395981	-0.04793	6.196733	-3.45747	0.001122	0.032173	-1.0959
ILMN_2382558	0.047558	6.634568	3.421302	0.001249	0.034372	-1.19509
ILMN_1680703	0.047456	10.15744	3.564625	0.000812	0.026563	-0.79833
ILMN_1651310	0.047223	5.64281	3.354328	0.001523	0.039187	-1.37711
ILMN_2144352	0.047113	6.173444	3.282606	0.001879	0.044661	-1.56954
ILMN_1730791	0.047039	6.350792	3.48274	0.00104	0.030849	-1.0262
ILMN_1750469	-0.04698	5.396838	-3.3328	0.001623	0.040674	-1.43515
ILMN_1677815	-0.04677	5.463739	-3.28251	0.00188	0.044661	-1.5698
ILMN_3263072	-0.04675	5.719879	-3.29313	0.001822	0.043875	-1.54148
ILMN_1670715	-0.04635	5.830224	-3.29621	0.001806	0.043695	-1.53324
ILMN_2388090	0.046143	5.945175	3.509002	0.000961	0.029523	-0.95346
ILMN_1791078	0.045855	5.184899	3.259839	0.002008	0.046877	-1.63007
ILMN_2163723	0.045843	5.592985	3.407223	0.001303	0.03535	-1.23354
ILMN_1697512	-0.04549	5.89182	-3.30871	0.001741	0.042537	-1.49981

ILMN_1694414	0.04496	5.429101	3.401469	0.001325	0.035568	-1.24922
ILMN_1712530	0.044705	8.099304	3.401023	0.001327	0.035569	-1.25044
ILMN_1737498	0.044274	6.296438	3.360911	0.001494	0.038607	-1.35932
ILMN_2049672	-0.0441	5.240343	-3.60686	0.000714	0.024876	-0.6796
ILMN_2408764	-0.04356	5.412849	-3.60787	0.000712	0.024854	-0.67676
ILMN_1840894	0.043502	6.436331	3.243958	0.002102	0.048158	-1.67214
ILMN_1767433	0.042732	6.231968	3.259041	0.002012	0.046877	-1.63219
ILMN_1827968	-0.04266	6.000498	-3.77051	0.000431	0.018733	-0.2121
ILMN_1765729	0.042419	6.196997	3.284876	0.001867	0.044569	-1.56349
ILMN_1887128	0.042084	6.128285	3.345266	0.001564	0.03991	-1.40157
ILMN_1748141	0.042007	6.114728	3.28873	0.001846	0.044255	-1.55321
ILMN_1788625	0.041414	6.60099	3.290554	0.001836	0.044143	-1.54834
ILMN_1817251	0.041404	5.987984	3.442482	0.001173	0.033169	-1.13708
ILMN_1706758	-0.04137	5.513889	-3.44413	0.001167	0.033144	-1.13256
ILMN_2163187	0.040297	5.70899	3.472954	0.001071	0.03123	-1.05323
ILMN_1736234	0.039261	8.677606	3.489328	0.001019	0.030581	-1.00799
ILMN_1766565	-0.03923	5.405056	-3.39722	0.001342	0.035945	-1.26079
ILMN_3265439	-0.03847	5.761817	-3.40664	0.001305	0.035367	-1.23513
ILMN_3246292	0.035704	13.61235	3.402413	0.001321	0.035549	-1.24665

Supplementary Table S3. Genes showing significant concentration-dependent associations with levels of HPV16 E6 protein in the W12 clones, after exclusion of known p53 targets.

	logFC	AveExpr	t	P.Value	adj.P.Val	B
ILMN_1768176	-1.03382	9.695149	-8.95765	5.63E-12	1.94E-07	16.72703
ILMN_1762899	-0.87966	11.10572	-6.27406	8.15E-08	6.25E-05	7.840583
ILMN_1718387	0.807518	7.067937	4.841749	1.28E-05	0.001949	3.122794
ILMN_1743199	-0.73382	8.006812	-5.5079	1.26E-06	0.000411	5.286692
ILMN_2096372	0.704396	6.843431	5.770864	4.94E-07	0.000216	6.157963
ILMN_1751607	-0.69388	8.653658	-4.02154	0.000195	0.012565	0.596512
ILMN_1693192	0.668337	10.53244	3.590024	0.000752	0.030491	-0.6419
ILMN_2188264	-0.63646	9.77076	-5.06548	5.91E-06	0.001252	3.841039
ILMN_1795930	-0.60207	8.793098	-4.89311	1.07E-05	0.001805	3.286776
ILMN_1693338	0.59907	8.093769	4.561861	3.30E-05	0.003717	2.239748
ILMN_1709348	0.573429	6.659982	6.073941	1.67E-07	0.000101	7.16964
ILMN_1801307	-0.56904	7.782393	-6.39114	5.35E-08	4.99E-05	8.233819
ILMN_1682775	-0.55521	9.613886	-6.35117	6.18E-08	5.32E-05	8.099527
ILMN_1705750	0.547466	9.719861	4.659485	2.38E-05	0.003001	2.545607
ILMN_1687768	-0.51624	10.72609	-4.09663	0.000153	0.010842	0.819255
ILMN_1682717	-0.50735	10.41325	-7.48282	1.05E-09	6.22E-06	11.89755
ILMN_1796316	0.50097	6.699623	3.60312	0.000722	0.029718	-0.60545
ILMN_1725338	-0.48667	7.689723	-5.26734	2.93E-06	0.000776	4.496938
ILMN_2166457	-0.47841	7.79733	-4.92266	9.68E-06	0.001702	3.381389
ILMN_1810835	-0.47277	11.7065	-3.40852	0.001298	0.042085	-1.13906
ILMN_1718977	-0.47091	8.552016	-6.83648	1.07E-08	1.95E-05	9.73125
ILMN_1722781	-0.46471	7.165493	-4.85394	1.23E-05	0.00192	3.16166
ILMN_1744604	0.45631	7.452409	3.464024	0.0011	0.037789	-0.98865
ILMN_1737406	-0.45431	10.1444	-6.67892	1.90E-08	2.84E-05	9.201421
ILMN_1702691	-0.43746	8.552129	-3.84523	0.000341	0.018239	0.081581
ILMN_3310326	-0.43645	8.573468	-6.20962	1.03E-07	7.38E-05	7.624351
ILMN_1682636	-0.41617	7.259545	-4.82506	1.35E-05	0.00201	3.069632
ILMN_1803811	-0.41345	10.1098	-4.89073	1.08E-05	0.001808	3.279161
ILMN_1791726	0.412745	7.649643	4.950285	8.80E-06	0.001614	3.469978
ILMN_1756417	-0.41226	8.04264	-6.432	4.62E-08	4.55E-05	8.371157
ILMN_1730223	-0.40347	8.039239	-5.23925	3.23E-06	0.000815	4.40528
ILMN_2150851	-0.39394	11.4861	-3.98027	0.000222	0.013772	0.474933
ILMN_2372082	-0.39154	6.926074	-4.68676	2.17E-05	0.002838	2.631475
ILMN_2401873	-0.38456	8.387228	-7.0614	4.78E-09	1.50E-05	10.48682
ILMN_2407389	-0.384	8.461271	-3.79291	0.000402	0.020087	-0.06896
ILMN_2197365	-0.37526	11.64348	-3.9879	0.000217	0.013562	0.497356
ILMN_1687978	-0.3748	9.9823	-4.05453	0.000175	0.011904	0.694121
ILMN_1692056	0.374319	7.887779	3.323063	0.00167	0.048443	-1.36774
ILMN_1671891	0.372118	7.065516	6.178178	1.15E-07	8.09E-05	7.518901
ILMN_3236367	-0.36952	9.576271	-4.43486	5.05E-05	0.004975	1.845619
ILMN_1801205	-0.36816	9.373757	-3.36587	0.001472	0.044915	-1.25363
ILMN_2091375	-0.36729	7.420736	-6.09985	1.52E-07	9.55E-05	7.256412
ILMN_2150856	-0.36279	12.20645	-4.226	0.000101	0.008103	1.207578
ILMN_1703123	-0.36254	9.308187	-4.86858	1.17E-05	0.001878	3.208387
ILMN_2348268	-0.36098	7.610188	-4.39821	5.70E-05	0.005463	1.732707
ILMN_1728677	-0.35577	6.90659	-4.31564	7.50E-05	0.006573	1.479802
ILMN_1815023	-0.35031	9.555022	-6.59904	2.53E-08	3.23E-05	8.93277
ILMN_2401878	-0.34689	8.20921	-7.28301	2.15E-09	8.24E-06	11.22969
ILMN_2255133	-0.34159	7.332635	-3.91723	0.000272	0.015534	0.290434
ILMN_1660000	-0.34137	7.522968	-5.25443	3.06E-06	0.000792	4.454812

ILMN_1781285	-0.34061	10.96082	-4.56122	3.31E-05	0.003717	2.237736
ILMN_1685663	0.340197	5.625189	6.886444	8.98E-09	1.76E-05	9.899184
ILMN_1772910	0.337649	8.398031	5.237567	3.25E-06	0.000815	4.399786
ILMN_1735014	-0.33475	11.25498	-5.38421	1.94E-06	0.000582	4.879647
ILMN_2318643	-0.33244	9.45439	-5.55689	1.06E-06	0.000368	5.448442
ILMN_2062701	0.331769	7.849424	5.899252	3.12E-07	0.00015	6.585714
ILMN_1781400	0.325712	6.351035	4.663803	2.34E-05	0.002972	2.559191
ILMN_2041190	-0.3207	10.1174	-6.13422	1.35E-07	9.11E-05	7.371555
ILMN_1745620	-0.32063	8.115913	-5.10287	5.19E-06	0.001155	3.962017
ILMN_2410145	-0.31926	6.066737	-3.90337	0.000284	0.015977	0.250072
ILMN_1782305	-0.3176	7.577045	-3.58188	0.000771	0.031037	-0.66453
ILMN_1665510	-0.31718	11.87829	-5.21583	3.50E-06	0.000863	4.328925
ILMN_1680139	-0.31587	8.145183	-4.52179	3.78E-05	0.004096	2.114932
ILMN_2318638	-0.31557	10.52254	-5.0145	7.05E-06	0.001405	3.676507
ILMN_1664922	0.310727	10.29266	4.153402	0.000127	0.009448	0.988979
ILMN_1760990	0.310301	6.710187	4.838381	1.29E-05	0.001963	3.11206
ILMN_1672295	-0.30925	8.098478	-4.56162	3.31E-05	0.003717	2.238984
ILMN_1669046	-0.30916	10.18236	-4.91511	9.93E-06	0.00173	3.357196
ILMN_1702127	0.30797	5.68991	6.32169	6.87E-08	5.64E-05	8.000517
ILMN_1800317	0.306635	6.100468	4.539929	3.56E-05	0.003963	2.171373
ILMN_1769201	-0.30486	7.974047	-3.60744	0.000713	0.02954	-0.59341
ILMN_1660067	-0.30206	7.137712	-4.27108	8.68E-05	0.007193	1.34415
ILMN_2203950	-0.30157	11.32692	-7.38567	1.49E-09	7.32E-06	11.57312
ILMN_1752899	-0.30078	7.611663	-4.66345	2.34E-05	0.002972	2.558078
ILMN_1806023	-0.29735	10.0989	-3.5611	0.000821	0.032122	-0.72215
ILMN_2094776	-0.29494	8.956537	-4.36015	6.47E-05	0.005918	1.615886
ILMN_1700978	-0.29442	6.835207	-4.38882	5.89E-05	0.005605	1.703833
ILMN_1693341	-0.28327	7.031799	-4.70825	2.01E-05	0.002728	2.699274
ILMN_1750062	0.282182	6.425835	3.937477	0.000255	0.015076	0.349533
ILMN_2404746	-0.28108	7.008499	-5.04043	6.45E-06	0.00133	3.760124
ILMN_2396020	-0.27892	10.40616	-3.37148	0.001448	0.044612	-1.23861
ILMN_2241124	-0.27586	7.144788	-3.6033	0.000722	0.029718	-0.60495
ILMN_1769520	-0.27388	8.983307	-3.53169	0.000897	0.03384	-0.80333
ILMN_1683475	0.273863	10.1368	6.880086	9.18E-09	1.76E-05	9.877815
ILMN_2148469	0.273003	8.810091	4.903693	1.03E-05	0.001773	3.32064
ILMN_1680132	0.271115	6.11318	4.746584	1.77E-05	0.002488	2.820471
ILMN_1802205	-0.27019	9.479563	-4.63939	2.54E-05	0.003133	2.482441
ILMN_1656057	-0.26939	10.21881	-6.06561	1.72E-07	0.000102	7.14174
ILMN_1683129	-0.26888	8.083049	-4.57886	3.12E-05	0.003609	2.292819
ILMN_1734929	-0.26869	9.387229	-4.28463	8.30E-05	0.006963	1.385333
ILMN_1721218	-0.26379	13.55456	-5.03088	6.66E-06	0.001345	3.729314
ILMN_1740466	-0.26359	8.466511	-3.6012	0.000727	0.029821	-0.61081
ILMN_1798826	0.263082	7.326562	5.572684	9.98E-07	0.000355	5.50065
ILMN_1776936	0.259812	6.817782	3.38528	0.00139	0.043808	-1.20161
ILMN_1778360	0.259386	10.85475	4.363111	6.41E-05	0.005894	1.624952
ILMN_1814215	0.258621	7.380656	3.748624	0.000461	0.022006	-0.19548
ILMN_1741159	-0.25759	6.520576	-4.63418	2.59E-05	0.003165	2.466102
ILMN_1672094	0.25669	6.754803	5.644283	7.75E-07	0.0003	5.737665
ILMN_2108735	0.256349	6.308979	3.902732	0.000285	0.015983	0.24822
ILMN_1708934	-0.25553	11.66936	-3.83767	0.000349	0.018505	0.059764
ILMN_2117171	-0.25546	10.7705	-6.36585	5.86E-08	5.32E-05	8.148862
ILMN_1765860	-0.25538	6.948261	-4.13747	0.000134	0.009865	0.941244
ILMN_1652580	0.253064	7.794553	8.102251	1.15E-10	1.32E-06	13.95083
ILMN_1769839	0.25285	5.604133	6.128481	1.38E-07	9.11E-05	7.352318

ILMN_1778561	-0.25082	9.533894	-4.57055	3.21E-05	0.003674	2.266872
ILMN_2339955	-0.25069	7.058355	-3.59767	0.000734	0.030072	-0.62061
ILMN_1703180	-0.25033	7.621498	-3.922	0.000268	0.015534	0.304351
ILMN_1703891	-0.24998	8.40525	-7.06577	4.70E-09	1.50E-05	10.50148
ILMN_1767365	-0.24925	7.134126	-5.31627	2.46E-06	0.000708	4.656915
ILMN_1745471	-0.24804	9.161866	-3.3681	0.001462	0.044858	-1.24766
ILMN_1708130	-0.24728	8.245502	-5.02869	6.71E-06	0.001346	3.722271
ILMN_1716988	-0.24717	8.836059	-3.36154	0.001491	0.045292	-1.26521
ILMN_1671895	0.245309	6.998514	6.332541	6.61E-08	5.56E-05	8.036961
ILMN_3268403	0.244386	5.923422	8.654311	1.63E-11	2.81E-07	15.75172
ILMN_2086077	-0.2431	8.675336	-3.43538	0.001198	0.039828	-1.06647
ILMN_2326712	-0.24246	7.108977	-4.47627	4.40E-05	0.004541	1.973628
ILMN_1797236	0.241859	6.569355	4.725654	1.90E-05	0.002638	2.754254
ILMN_1742166	0.240845	8.381935	5.346771	2.21E-06	0.000652	4.756829
ILMN_1716446	-0.23767	7.655153	-4.80927	1.43E-05	0.002095	3.019381
ILMN_1675616	-0.23565	6.875555	-3.89156	0.000295	0.016344	0.215735
ILMN_2130411	0.233589	9.58704	4.65257	2.43E-05	0.003061	2.523861
ILMN_1765876	-0.23238	6.010988	-4.58813	3.02E-05	0.003534	2.321806
ILMN_1744239	-0.23034	7.234171	-6.78063	1.31E-08	2.27E-05	9.543456
ILMN_3310840	-0.22884	7.508302	-3.93366	0.000258	0.015209	0.338395
ILMN_2048633	-0.2287	6.738295	-5.3857	1.93E-06	0.000582	4.88454
ILMN_1800225	0.228522	7.195323	3.5409	0.000873	0.033133	-0.77795
ILMN_1815079	-0.22815	7.332429	-3.51247	0.000951	0.034911	-0.8562
ILMN_1675992	-0.22797	7.571294	-5.50029	1.29E-06	0.000412	5.261591
ILMN_2391264	0.227488	9.11132	3.701895	0.000533	0.024369	-0.32812
ILMN_1674620	-0.22676	8.612633	-4.50185	4.04E-05	0.004285	2.052949
ILMN_1778319	0.225956	9.088586	3.50222	0.000981	0.035476	-0.8843
ILMN_1813489	0.225848	7.79926	6.313999	7.06E-08	5.66E-05	7.97469
ILMN_2229379	-0.22567	6.13363	-3.56712	0.000806	0.03191	-0.70546
ILMN_1758213	0.225133	7.772904	5.891516	3.21E-07	0.000151	6.559904
ILMN_1759184	0.22512	9.801106	4.806981	1.44E-05	0.002102	3.012107
ILMN_1736729	-0.22441	6.390611	-3.36988	0.001455	0.044742	-1.2429
ILMN_1701837	-0.22393	8.208807	-6.43845	4.51E-08	4.55E-05	8.392816
ILMN_2285404	0.223459	9.479853	3.969103	0.000231	0.01406	0.442146
ILMN_3247848	0.223135	6.89447	4.776816	1.60E-05	0.002291	2.91629
ILMN_1786429	-0.22311	8.409264	-3.50608	0.000969	0.035251	-0.87372
ILMN_1720373	0.222294	11.35699	4.863951	1.18E-05	0.001881	3.193611
ILMN_1683127	-0.22173	9.525091	-3.60572	0.000717	0.029624	-0.5982
ILMN_2103397	0.221546	6.499243	5.637934	7.92E-07	0.000303	5.716625
ILMN_1805064	-0.2211	7.068874	-3.54303	0.000867	0.033102	-0.77207
ILMN_2191568	0.221059	8.435229	5.503112	1.28E-06	0.000411	5.270902
ILMN_1738759	0.220462	8.423502	7.643064	5.90E-10	5.09E-06	12.43144
ILMN_1719204	0.220104	9.238663	6.026292	1.98E-07	0.000112	7.010176
ILMN_1800573	0.215836	11.20054	4.014893	0.000199	0.012768	0.576886
ILMN_1669113	0.215551	7.508813	5.291409	2.69E-06	0.000748	4.575597
ILMN_1705302	0.2149	6.800363	3.743804	0.000468	0.022277	-0.2092
ILMN_3223181	0.214885	8.419154	4.188107	0.000114	0.008774	1.093256
ILMN_1762275	0.214858	7.5074	5.87017	3.47E-07	0.000159	6.488707
ILMN_1844611	0.214577	6.621864	6.353854	6.12E-08	5.32E-05	8.108555
ILMN_1680453	0.214413	7.924674	4.24876	9.34E-05	0.007589	1.276446
ILMN_2168520	-0.21417	7.828221	-3.7321	0.000486	0.022942	-0.24248
ILMN_1702609	-0.21403	8.422294	-4.40067	5.66E-05	0.005449	1.740287
ILMN_1686555	0.213184	6.820999	4.425712	5.21E-05	0.0051	1.8174
ILMN_2365595	-0.21305	9.784162	-5.19802	3.73E-06	0.000899	4.270947

ILMN_1701331	0.211983	9.805817	7.313114	1.93E-09	8.24E-06	11.33046
ILMN_2383484	0.21142	8.386668	5.809561	4.30E-07	0.000193	6.286748
ILMN_1722634	0.211185	10.63075	4.449875	4.80E-05	0.004816	1.891981
ILMN_1668629	0.211152	9.24065	3.597223	0.000735	0.030077	-0.62187
ILMN_2396982	0.21085	9.162116	6.050179	1.82E-07	0.000106	7.090099
ILMN_1691731	-0.21033	6.980087	-3.57931	0.000777	0.031208	-0.67165
ILMN_1724181	-0.21008	6.919197	-4.97365	8.12E-06	0.001544	3.545032
ILMN_1799890	0.209114	8.428338	4.974736	8.09E-06	0.001544	3.548524
ILMN_1740429	0.208858	12.17058	3.860684	0.000325	0.017647	0.126232
ILMN_1808713	0.208271	5.671269	4.202028	0.000109	0.008524	1.135196
ILMN_2307450	0.208056	8.411141	3.997351	0.000211	0.013255	0.525175
ILMN_2178855	0.206915	7.786853	5.127087	4.77E-06	0.001087	4.040485
ILMN_1662640	0.206595	9.417851	3.974895	0.000226	0.01391	0.459146
ILMN_1700109	0.205742	9.491388	4.03934	0.000184	0.012201	0.649131
ILMN_1679093	0.205328	9.146616	3.763036	0.000441	0.021449	-0.15439
ILMN_1808777	0.205162	8.520059	4.503022	4.02E-05	0.004281	2.056601
ILMN_1677919	-0.20408	8.942454	-5.13596	4.63E-06	0.001068	4.069263
ILMN_1772981	0.204013	10.56743	5.902338	3.09E-07	0.00015	6.596011
ILMN_1653358	-0.20323	6.534286	-3.3976	0.00134	0.042747	-1.1685
ILMN_2074748	0.202406	6.155482	5.343068	2.24E-06	0.000655	4.744693
ILMN_1754842	0.20212	7.573756	4.868569	1.17E-05	0.001878	3.208355
ILMN_1794190	-0.20197	6.986232	-4.46534	4.56E-05	0.00468	1.939801
ILMN_1752953	0.201267	8.974141	5.614121	8.62E-07	0.000323	5.637752
ILMN_1690105	-0.20106	8.328354	-3.33759	0.0016	0.047389	-1.32912
ILMN_1775962	-0.20082	7.38938	-4.45582	4.71E-05	0.00479	1.910353
ILMN_1684964	0.200763	7.346486	4.236896	9.71E-05	0.007856	1.24052
ILMN_2381197	-0.20059	7.79262	-4.9057	1.03E-05	0.001773	3.327072
ILMN_1703330	-0.20049	8.192523	-3.91776	0.000271	0.015534	0.29198
ILMN_1736575	0.200006	8.552527	4.937363	9.20E-06	0.001652	3.428517
ILMN_1770537	0.199743	6.388881	5.586075	9.52E-07	0.000342	5.544937
ILMN_1755909	0.199555	9.400336	6.416282	4.89E-08	4.68E-05	8.318317
ILMN_2285817	-0.19951	9.961391	-3.83166	0.000356	0.018691	0.042439
ILMN_1683635	-0.19937	7.995629	-3.63033	0.000665	0.028015	-0.52947
ILMN_3235325	0.199317	6.910267	3.529837	0.000902	0.03396	-0.80844
ILMN_1693421	0.1992	9.359146	6.459711	4.18E-08	4.37E-05	8.464292
ILMN_2301083	0.19913	10.92932	4.834955	1.31E-05	0.001977	3.101144
ILMN_2120022	-0.19867	7.027162	-4.02655	0.000192	0.012526	0.611297
ILMN_1697218	0.198196	8.347213	5.058721	6.05E-06	0.001272	3.819194
ILMN_1676515	0.197903	7.899452	3.692502	0.000549	0.024798	-0.35467
ILMN_1658425	0.197032	8.352206	5.248425	3.13E-06	0.000798	4.435204
ILMN_1674034	-0.19643	8.200939	-4.31745	7.45E-05	0.006553	1.485324
ILMN_1675709	0.195906	7.207505	6.653702	2.08E-08	2.87E-05	9.116617
ILMN_2061732	-0.19519	9.634742	-4.02326	0.000194	0.012553	0.601599
ILMN_3247018	-0.19483	8.265002	-5.99347	2.23E-07	0.000122	6.90042
ILMN_1704793	0.194785	8.555961	4.669246	2.30E-05	0.002936	2.576319
ILMN_1676631	0.194407	6.992579	3.463795	0.0011	0.037789	-0.98928
ILMN_2173611	0.193725	12.17228	3.807335	0.000384	0.019694	-0.02755
ILMN_1738681	0.19294	10.25065	6.114714	1.44E-07	9.22E-05	7.306192
ILMN_1703487	-0.19257	8.843029	-4.56564	3.26E-05	0.003717	2.251535
ILMN_2392370	0.192392	6.342761	3.595252	0.00074	0.030187	-0.62736
ILMN_1691436	-0.19208	9.590865	-4.60436	2.86E-05	0.003415	2.372605
ILMN_1764571	0.191231	10.18131	3.615479	0.000696	0.028963	-0.57098
ILMN_1656933	0.191167	6.331613	4.477424	4.38E-05	0.004538	1.977217
ILMN_1669479	0.191121	7.352698	3.574264	0.000789	0.031452	-0.68566

ILMN_1665831	0.189996	8.995949	4.524969	3.74E-05	0.004072	2.124809
ILMN_2183409	0.18901	8.259481	4.319412	7.40E-05	0.006528	1.491297
ILMN_1656670	-0.18857	6.613323	-5.73256	5.66E-07	0.000235	6.030603
ILMN_1715401	0.188487	8.08212	3.472876	0.001071	0.03739	-0.96453
ILMN_1710752	-0.18813	8.511522	-4.88736	1.09E-05	0.001811	3.268396
ILMN_1789642	0.188095	6.892939	5.311884	2.50E-06	0.000713	4.64257
ILMN_1811195	0.188049	6.387587	4.621262	2.70E-05	0.003271	2.425565
ILMN_1694111	0.1879	8.130744	5.339762	2.27E-06	0.000657	4.733859
ILMN_1810467	0.187843	10.56074	4.848859	1.25E-05	0.001927	3.145461
ILMN_1815306	0.187757	7.293171	5.737409	5.56E-07	0.000234	6.046731
ILMN_1913510	0.187665	6.677522	4.039633	0.000184	0.012201	0.65
ILMN_1679401	0.187491	7.031703	4.375481	6.15E-05	0.005732	1.662888
ILMN_2204467	0.187172	5.783277	3.472004	0.001074	0.037399	-0.96691
ILMN_1685540	0.187144	7.832534	4.866844	1.17E-05	0.00188	3.202847
ILMN_1684873	-0.18681	6.878608	-3.71091	0.000519	0.023964	-0.3026
ILMN_1704418	-0.18673	7.854408	-3.60203	0.000725	0.029781	-0.60849
ILMN_2408566	-0.1865	6.390289	-3.49429	0.001004	0.035992	-0.90602
ILMN_1706342	0.186336	7.352996	4.104384	0.000149	0.010661	0.842382
ILMN_2048478	0.186003	6.143759	4.949481	8.83E-06	0.001614	3.467399
ILMN_1658989	0.185825	6.267611	5.972922	2.40E-07	0.000125	6.831728
ILMN_2411897	-0.18571	7.291889	-3.91383	0.000275	0.015619	0.280544
ILMN_1746494	-0.18567	7.881911	-4.30253	7.83E-05	0.006729	1.439812
ILMN_1672504	0.185439	9.839358	4.481865	4.32E-05	0.004511	1.990976
ILMN_1731720	0.18528	7.562784	5.004482	7.30E-06	0.001422	3.644236
ILMN_1671191	0.185117	10.86306	4.712733	1.98E-05	0.002726	2.713431
ILMN_1907834	-0.18486	8.321906	-3.70227	0.000533	0.024369	-0.32706
ILMN_1715476	0.184802	9.924509	4.832868	1.32E-05	0.001983	3.094494
ILMN_2045994	0.184523	8.174052	5.06525	5.91E-06	0.001252	3.840292
ILMN_1676026	0.183751	8.684089	4.929238	9.46E-06	0.001673	3.402464
ILMN_2367191	0.183402	9.505748	4.685883	2.17E-05	0.002838	2.628721
ILMN_2173294	-0.18334	6.696235	-4.37644	6.13E-05	0.005729	1.665827
ILMN_2366041	0.183227	7.879147	4.769074	1.64E-05	0.002343	2.891731
ILMN_1701731	0.183105	9.033921	4.410899	5.47E-05	0.005297	1.771758
ILMN_1773154	-0.18285	11.15561	-3.72109	0.000503	0.023418	-0.27374
ILMN_1804822	0.182836	8.721351	3.770641	0.000431	0.021134	-0.13267
ILMN_1762284	0.182677	5.861403	6.463156	4.13E-08	4.37E-05	8.475871
ILMN_1692790	-0.18232	8.579602	-5.20682	3.61E-06	0.000884	4.299579
ILMN_1759175	-0.18216	6.883299	-5.73779	5.56E-07	0.000234	6.047989
ILMN_1699570	0.181596	9.942182	6.074689	1.67E-07	0.000101	7.172143
ILMN_2363273	0.181107	7.012101	4.304059	7.79E-05	0.006712	1.444479
ILMN_2405680	-0.18109	7.10555	-3.62337	0.000679	0.028507	-0.54893
ILMN_2372639	-0.18043	8.914962	-3.65524	0.000616	0.026807	-0.45962
ILMN_3297880	0.179973	8.630585	4.112943	0.000145	0.010435	0.86792
ILMN_1763605	0.17932	6.9534	6.890572	8.84E-09	1.76E-05	9.913057
ILMN_1751886	-0.17897	6.981708	-3.36696	0.001467	0.044915	-1.25072
ILMN_1807833	0.17857	8.049537	6.275391	8.11E-08	6.25E-05	7.845061
ILMN_1730906	-0.17854	7.259968	-3.65798	0.000611	0.02665	-0.45194
ILMN_2410772	-0.17831	8.289186	-4.96176	8.46E-06	0.001593	3.50684
ILMN_2375360	-0.17815	7.721413	-3.7974	0.000396	0.02001	-0.05608
ILMN_1790160	-0.1778	6.571739	-3.99516	0.000212	0.013299	0.51873
ILMN_1786976	0.17774	8.587619	5.100876	5.23E-06	0.001155	3.955546
ILMN_1664608	0.177569	8.508847	4.731513	1.86E-05	0.002598	2.772779
ILMN_2278653	0.177359	6.360961	5.055751	6.11E-06	0.001277	3.809601
ILMN_1692100	0.176841	7.14129	4.398596	5.70E-05	0.005463	1.733899

ILMN_1801442	0.176668	6.102062	4.128634	0.000138	0.010087	0.914804
ILMN_1709044	0.176641	6.638544	4.415896	5.38E-05	0.005254	1.787148
ILMN_2132458	-0.17657	6.416718	-3.43916	0.001185	0.03959	-1.0562
ILMN_1688154	0.175568	6.980757	3.562912	0.000816	0.032056	-0.71712
ILMN_2334350	0.175562	8.363866	4.077985	0.000163	0.011215	0.763771
ILMN_1679655	0.175524	8.886264	3.498224	0.000992	0.035741	-0.89525
ILMN_1666078	-0.17499	7.427113	-6.21834	9.96E-08	7.31E-05	7.653598
ILMN_2201347	0.174985	7.533335	4.972513	8.15E-06	0.001544	3.541379
ILMN_2095704	0.174738	6.640483	3.422096	0.001246	0.040958	-1.10241
ILMN_1714730	0.174704	10.85933	4.168721	0.000121	0.009087	1.034957
ILMN_1794643	0.174571	7.454543	5.560479	1.04E-06	0.000367	5.460306
ILMN_1771233	0.174517	8.313767	6.470291	4.02E-08	4.37E-05	8.499858
ILMN_1697363	0.174309	8.470232	3.520852	0.000927	0.034592	-0.83316
ILMN_1677534	0.17419	8.546457	3.366014	0.001472	0.044915	-1.25325
ILMN_1652677	-0.17391	8.342189	-3.3957	0.001348	0.042868	-1.17359
ILMN_1729225	0.173908	6.254295	3.477504	0.001056	0.036968	-0.9519
ILMN_1722811	0.173901	7.815689	3.311124	0.001729	0.049265	-1.3994
ILMN_1802615	0.173846	9.055348	3.418623	0.001259	0.041228	-1.1118
ILMN_3236061	0.173539	6.878669	4.314972	7.51E-05	0.006573	1.47775
ILMN_2324056	0.173418	9.370995	3.687029	0.000558	0.024842	-0.37013
ILMN_1816342	-0.17299	7.914214	-3.83787	0.000349	0.018505	0.060324
ILMN_1806790	0.172863	7.722227	4.051389	0.000177	0.011939	0.684819
ILMN_2120340	0.172707	8.387977	5.267454	2.92E-06	0.000776	4.497322
ILMN_1660973	-0.17264	6.450034	-3.4078	0.0013	0.042135	-1.14101
ILMN_2234343	0.172509	6.551563	3.954537	0.000242	0.014507	0.399445
ILMN_2081398	0.17235	7.795199	5.985085	2.30E-07	0.000124	6.872378
ILMN_1744046	-0.17216	7.48544	-5.01294	7.09E-06	0.001405	3.671498
ILMN_1778374	-0.17183	10.17323	-4.90213	1.04E-05	0.001773	3.315629
ILMN_2382829	0.171691	9.007533	6.150213	1.27E-07	8.77E-05	7.425149
ILMN_2105308	0.171574	10.92409	4.590978	2.99E-05	0.003524	2.330717
ILMN_2102693	-0.17083	8.757085	-3.56228	0.000818	0.032056	-0.71886
ILMN_2102960	-0.17065	8.064303	-4.38512	5.96E-05	0.005643	1.692474
ILMN_3197097	-0.17064	8.289104	-3.7302	0.000489	0.023046	-0.24787
ILMN_1790136	0.16997	8.813088	4.323791	7.30E-05	0.006451	1.504667
ILMN_1678454	-0.16991	9.454743	-5.16909	4.12E-06	0.000967	4.176853
ILMN_1745242	-0.16991	7.429544	-4.03891	0.000184	0.012201	0.647854
ILMN_1777721	0.169788	10.37908	5.607787	8.82E-07	0.000327	5.616784
ILMN_1710000	-0.16934	6.626573	-6.54454	3.08E-08	3.79E-05	8.749499
ILMN_1812856	0.16923	8.03135	7.474006	1.08E-09	6.22E-06	11.86813
ILMN_1728057	-0.1692	6.695851	-5.07963	5.63E-06	0.001219	3.886771
ILMN_1732398	0.168896	5.518001	4.264737	8.86E-05	0.007286	1.324894
ILMN_1719986	-0.16868	7.162071	-3.39488	0.001351	0.042894	-1.1758
ILMN_2359453	0.168482	10.66599	4.763603	1.67E-05	0.002377	2.874385
ILMN_1792679	-0.16835	7.392167	-3.46422	0.001099	0.037789	-0.98813
ILMN_1806106	0.168007	9.807794	3.83157	0.000356	0.018691	0.042166
ILMN_1784110	-0.16771	6.786576	-3.85135	0.000335	0.018029	0.099257
ILMN_1700541	0.167671	8.967071	3.439206	0.001184	0.03959	-1.05609
ILMN_2368713	0.167403	10.02075	4.563355	3.29E-05	0.003717	2.244411
ILMN_1707783	0.166958	11.66934	3.743032	0.00047	0.022299	-0.2114
ILMN_2410771	-0.16683	8.061131	-5.50997	1.25E-06	0.000411	5.293528
ILMN_1742731	0.16672	8.820646	5.112077	5.03E-06	0.001126	3.991829
ILMN_1676984	-0.16655	6.687393	-4.85233	1.23E-05	0.001922	3.156543
ILMN_2148668	-0.1663	7.46236	-3.89921	0.000288	0.016136	0.237968
ILMN_1664028	0.166099	9.475459	4.855172	1.22E-05	0.00192	3.165597

ILMN_1680465	-0.16545	6.953166	-3.48612	0.001029	0.036544	-0.92836
ILMN_1735658	-0.16523	9.171766	-3.96452	0.000234	0.014175	0.428704
ILMN_1652594	0.165046	6.7758	5.268598	2.91E-06	0.000776	4.50106
ILMN_1801313	-0.16471	8.011372	-3.45476	0.001131	0.038365	-1.01385
ILMN_1700168	0.164566	6.401959	3.921981	0.000268	0.015534	0.304292
ILMN_1815024	-0.164	10.93684	-4.3012	7.86E-05	0.006742	1.43576
ILMN_1658992	0.163676	10.34544	5.267271	2.93E-06	0.000776	4.496723
ILMN_1807291	0.16353	5.682917	3.594647	0.000741	0.030188	-0.62904
ILMN_1728710	0.16342	6.954481	3.5585	0.000827	0.032266	-0.72933
ILMN_1712035	0.163416	7.517737	3.893636	0.000293	0.01629	0.221775
ILMN_1660723	0.163269	7.815519	3.470366	0.001079	0.037464	-0.97137
ILMN_1733667	0.163041	7.146015	5.657224	7.40E-07	0.00029	5.780561
ILMN_1791388	0.162698	8.038381	4.974707	8.09E-06	0.001544	3.548431
ILMN_1683313	0.162422	7.73199	4.878969	1.12E-05	0.001838	3.241577
ILMN_2142117	-0.16231	9.308538	-4.11217	0.000146	0.01044	0.865606
ILMN_2394276	-0.16208	7.018932	-5.0389	6.48E-06	0.00133	3.755186
ILMN_1714741	-0.16198	7.070954	-3.80551	0.000386	0.01977	-0.03281
ILMN_3244323	0.161878	8.420129	4.988456	7.72E-06	0.001494	3.592649
ILMN_1734138	0.16177	7.228567	4.191438	0.000113	0.008743	1.103288
ILMN_1785644	0.161693	7.199323	4.169125	0.000121	0.009087	1.036173
ILMN_1705252	0.161587	6.430172	3.725341	0.000496	0.023197	-0.26168
ILMN_1751464	-0.1612	6.77298	-3.9164	0.000273	0.015534	0.288014
ILMN_1803856	-0.16071	6.942534	-3.62339	0.000679	0.028507	-0.54888
ILMN_1781454	0.160641	8.591168	4.377337	6.11E-05	0.005728	1.668583
ILMN_1669703	0.160472	8.590866	3.822009	0.000367	0.019072	0.014633
ILMN_1798256	-0.16026	10.07763	-3.58664	0.00076	0.030714	-0.6513
ILMN_1681326	0.160005	5.931447	6.236458	9.33E-08	6.99E-05	7.714393
ILMN_1701243	0.160004	7.875346	4.038393	0.000185	0.012201	0.646331
ILMN_1714352	0.159676	7.136768	3.320255	0.001683	0.048567	-1.37519
ILMN_2326509	-0.15948	8.811935	-3.43285	0.001207	0.040051	-1.07331
ILMN_3239217	0.159464	6.633795	4.571844	3.19E-05	0.00367	2.270912
ILMN_1670079	-0.15928	8.026146	-3.49006	0.001017	0.036364	-0.9176
ILMN_1745885	0.159247	10.33155	4.22465	0.000101	0.00812	1.203485
ILMN_1756806	-0.15912	8.925277	-3.55267	0.000842	0.032579	-0.74544
ILMN_1801833	-0.15905	6.69055	-3.43092	0.001214	0.040167	-1.07855
ILMN_1665526	0.158704	6.424292	3.417115	0.001265	0.041319	-1.11587
ILMN_1695110	0.158563	7.942509	3.876396	0.000309	0.016928	0.171736
ILMN_1719286	0.158512	7.652052	3.963292	0.000235	0.014201	0.425102
ILMN_1702211	-0.15839	6.537614	-3.74684	0.000464	0.022098	-0.20055
ILMN_1764522	0.158016	7.828939	3.384367	0.001394	0.043886	-1.20406
ILMN_1687315	0.157577	10.13046	3.466804	0.001091	0.037673	-0.98108
ILMN_1790455	0.157531	6.554547	4.143801	0.000131	0.009706	0.960202
ILMN_1700727	-0.15706	6.382961	-4.70373	2.05E-05	0.002744	2.684995
ILMN_1683204	0.156723	8.05968	5.907633	3.03E-07	0.000149	6.613683
ILMN_1683082	0.156607	7.593366	4.525294	3.73E-05	0.004072	2.125819
ILMN_1808591	0.156492	9.949389	4.102476	0.00015	0.010705	0.836692
ILMN_1784641	0.156322	10.54662	3.456775	0.001124	0.038324	-1.00838
ILMN_1771815	0.156272	10.19336	6.654702	2.07E-08	2.87E-05	9.119979
ILMN_1750400	-0.15614	6.36807	-3.86324	0.000322	0.017561	0.13363
ILMN_1663954	0.15608	9.208176	3.94169	0.000252	0.014928	0.361851
ILMN_1797698	0.155868	7.248442	6.529965	3.25E-08	3.86E-05	8.700493
ILMN_1768391	-0.15583	6.295915	-4.49123	4.19E-05	0.004399	2.020023
ILMN_1737535	0.15535	7.809927	4.879032	1.12E-05	0.001838	3.241776
ILMN_1812940	0.155224	8.171079	4.230794	9.90E-05	0.007996	1.22206

ILMN_2407464	0.155055	8.370746	3.715812	0.000511	0.023707	-0.28871
ILMN_1770505	-0.155	6.104167	-3.63683	0.000652	0.0277	-0.51127
ILMN_1658706	-0.15463	7.564188	-3.40987	0.001292	0.041956	-1.13543
ILMN_1665797	0.15461	10.52642	3.881786	0.000304	0.016722	0.187367
ILMN_2125010	-0.15444	7.894929	-4.34548	6.79E-05	0.006115	1.570943
ILMN_1716821	0.154382	8.044105	3.508274	0.000963	0.035111	-0.8677
ILMN_1737426	-0.15423	9.140574	-3.6893	0.000555	0.024798	-0.3637
ILMN_1772731	-0.15403	8.037967	-3.36542	0.001474	0.044936	-1.25485
ILMN_1751743	0.153779	7.111816	4.868853	1.16E-05	0.001878	3.209261
ILMN_3244803	0.153575	6.940247	4.372629	6.21E-05	0.00577	1.654138
ILMN_1691117	0.153522	8.642115	5.032905	6.62E-06	0.001345	3.735851
ILMN_2126239	0.153477	7.785695	3.752896	0.000455	0.021837	-0.18331
ILMN_1722218	0.153028	7.885802	4.298708	7.93E-05	0.00678	1.428178
ILMN_1759436	0.152915	9.846954	3.697246	0.000541	0.024611	-0.34126
ILMN_1669390	0.152882	9.29823	3.364853	0.001477	0.044971	-1.25636
ILMN_1654001	-0.15274	7.716196	-4.29692	7.97E-05	0.006787	1.422735
ILMN_2183687	0.152672	6.975102	4.950134	8.81E-06	0.001614	3.469493
ILMN_1755352	0.152606	5.878212	3.466495	0.001092	0.037673	-0.98192
ILMN_1771987	0.152459	9.357103	3.754671	0.000453	0.021777	-0.17825
ILMN_2265654	0.152282	6.254246	5.048628	6.27E-06	0.001301	3.786596
ILMN_1760490	-0.1522	8.651016	-4.7446	1.78E-05	0.002495	2.814195
ILMN_1675007	0.152128	6.451682	5.030493	6.67E-06	0.001345	3.72807
ILMN_1666385	0.152057	11.11719	3.755176	0.000452	0.021773	-0.17681
ILMN_2075820	-0.15176	7.453887	-4.79889	1.48E-05	0.002143	2.986394
ILMN_1811729	0.151555	7.58875	3.796402	0.000398	0.020014	-0.05893
ILMN_2357781	0.151529	6.984022	3.488554	0.001022	0.036428	-0.92172
ILMN_1810334	0.151444	8.569222	4.051957	0.000177	0.011939	0.686501
ILMN_1782829	0.151193	6.668202	4.027037	0.000192	0.012526	0.612747
ILMN_1711383	0.151177	9.415097	5.291891	2.68E-06	0.000748	4.577174
ILMN_1735955	0.151077	8.686113	3.319045	0.001689	0.048699	-1.37841
ILMN_1729368	0.151074	6.394802	5.910464	3.00E-07	0.000149	6.623132
ILMN_1668369	-0.15095	11.22509	-3.76059	0.000445	0.02153	-0.16137
ILMN_3248857	-0.1505	6.346806	-4.03408	0.000187	0.012277	0.633578
ILMN_2323491	0.150433	8.391228	4.828619	1.34E-05	0.001995	3.08096
ILMN_1810891	0.150375	7.421438	3.953964	0.000242	0.014507	0.397768
ILMN_2283388	0.149732	10.62316	3.942651	0.000251	0.014908	0.36466
ILMN_1776375	-0.14925	9.59331	-4.89796	1.05E-05	0.001789	3.302307
ILMN_1728059	0.149219	7.675856	4.047887	0.000179	0.012019	0.674441
ILMN_1674394	0.149054	8.769143	3.465469	0.001095	0.037751	-0.98472
ILMN_1683059	-0.14904	7.756932	-3.41532	0.001272	0.041439	-1.12073
ILMN_1765684	-0.14901	10.39111	-3.42716	0.001228	0.040541	-1.08872
ILMN_1711450	0.148957	9.412244	3.927086	0.000264	0.015398	0.319188
ILMN_3225784	0.148858	12.80393	3.32925	0.00164	0.047945	-1.35131
ILMN_1770653	-0.14855	11.45875	-4.36096	6.45E-05	0.005918	1.618345
ILMN_1717809	0.148416	6.697764	3.762703	0.000442	0.021449	-0.15534
ILMN_1755862	0.147969	8.186322	3.553976	0.000839	0.032546	-0.74184
ILMN_1667594	-0.14787	6.850008	-3.71985	0.000505	0.023476	-0.27725
ILMN_1770338	-0.14774	11.67637	-3.45529	0.001129	0.038349	-1.01242
ILMN_1777449	-0.14767	7.981589	-3.37235	0.001444	0.044537	-1.23628
ILMN_1725427	-0.14763	11.94789	-3.40402	0.001315	0.042371	-1.15118
ILMN_1713006	-0.14757	7.403597	-3.93071	0.000261	0.0153	0.329753
ILMN_1793203	0.147465	7.931098	4.558502	3.34E-05	0.003739	2.229268
ILMN_2332558	-0.14715	9.264805	-4.58897	3.01E-05	0.003534	2.32445
ILMN_1710844	-0.1471	6.676328	-3.89772	0.000289	0.016159	0.23364

ILMN_2359456	0.146922	8.970854	4.048175	0.000179	0.012019	0.675296
ILMN_2173451	0.146891	9.157835	3.630748	0.000664	0.028014	-0.5283
ILMN_1792748	0.14676	6.144422	4.194433	0.000112	0.008678	1.112308
ILMN_1738229	0.146561	8.189289	4.210926	0.000106	0.008375	1.162037
ILMN_1652754	0.146215	8.936248	3.558785	0.000827	0.032266	-0.72854
ILMN_3272768	-0.14617	6.624888	-6.00888	2.11E-07	0.000117	6.951944
ILMN_1737685	0.146161	9.912017	3.336211	0.001606	0.047499	-1.33279
ILMN_1769575	0.146114	5.614811	3.64837	0.000629	0.027207	-0.47892
ILMN_1695679	-0.14602	6.135872	-3.37692	0.001425	0.044289	-1.22404
ILMN_1711023	0.145942	8.144351	4.711132	1.99E-05	0.002726	2.708372
ILMN_1792825	0.145859	7.809389	3.314944	0.00171	0.04894	-1.38928
ILMN_1676197	0.145858	10.08529	3.37078	0.001451	0.044664	-1.24049
ILMN_2065783	-0.14557	7.062967	-5.69413	6.49E-07	0.000263	5.902991
ILMN_1770035	0.14557	7.938254	6.460908	4.16E-08	4.37E-05	8.468315
ILMN_1721225	0.145562	8.673909	6.047725	1.84E-07	0.000106	7.081889
ILMN_1790537	-0.14556	8.683089	-3.32668	0.001652	0.048147	-1.35814
ILMN_3227263	-0.14552	7.69568	-3.4178	0.001262	0.04129	-1.11403
ILMN_1741585	0.145515	6.201362	4.582443	3.08E-05	0.003583	2.304029
ILMN_1767658	0.14546	8.743809	4.248171	9.36E-05	0.007589	1.274661
ILMN_2144573	-0.14531	7.407936	-6.93279	7.60E-09	1.75E-05	10.05492
ILMN_1653133	-0.14513	8.267472	-5.27953	2.80E-06	0.000773	4.53677
ILMN_3265237	-0.1451	7.012253	-3.74245	0.00047	0.022309	-0.21306
ILMN_1696190	0.145057	7.341854	5.179287	3.98E-06	0.00094	4.209998
ILMN_1661484	0.144923	6.477409	3.85755	0.000328	0.017766	0.117166
ILMN_1718069	-0.14488	9.717053	-3.65846	0.00061	0.026644	-0.4506
ILMN_1753885	0.144481	8.759723	5.668451	7.11E-07	0.000282	5.81779
ILMN_3303673	-0.14438	7.409118	-3.84674	0.00034	0.018181	0.085922
ILMN_1728972	0.144304	7.406871	4.051157	0.000177	0.011939	0.68413
ILMN_1654488	-0.14408	7.884099	-4.0157	0.000199	0.012768	0.579259
ILMN_1702073	-0.14407	7.020506	-5.35732	2.13E-06	0.000634	4.791413
ILMN_1688642	0.143925	6.268217	3.549667	0.00085	0.03277	-0.75375
ILMN_2050255	0.143898	8.970775	5.739448	5.52E-07	0.000234	6.053507
ILMN_1800311	-0.14389	7.430698	-4.45751	4.68E-05	0.004777	1.915581
ILMN_1733863	-0.14314	8.329745	-3.33721	0.001602	0.047401	-1.33014
ILMN_1683533	0.143067	6.642611	4.441495	4.94E-05	0.004927	1.866095
ILMN_1729142	0.143047	6.377382	3.366584	0.001469	0.044915	-1.25173
ILMN_1692754	-0.14238	9.73637	-3.57614	0.000784	0.031437	-0.68046
ILMN_2207393	0.142323	7.21087	4.884599	1.10E-05	0.00182	3.259567
ILMN_1651228	-0.14228	13.36566	-6.97261	6.58E-09	1.62E-05	10.18869
ILMN_1765770	0.14225	6.899082	3.377593	0.001422	0.044289	-1.22223
ILMN_1690610	0.142134	8.977126	4.69125	2.13E-05	0.002819	2.645638
ILMN_1745784	0.141999	6.744723	3.693038	0.000548	0.024798	-0.35316
ILMN_1687857	0.141927	7.076059	3.472706	0.001072	0.03739	-0.96499
ILMN_3235808	0.141921	9.152855	4.948784	8.85E-06	0.001614	3.465161
ILMN_1758173	-0.14191	9.107964	-4.68629	2.17E-05	0.002838	2.630005
ILMN_1700306	-0.14173	10.1864	-3.66853	0.000591	0.025996	-0.42225
ILMN_1788547	0.14165	8.061504	3.807212	0.000384	0.019694	-0.02791
ILMN_1682935	-0.1416	8.379604	-4.12118	0.000141	0.010277	0.892533
ILMN_1804329	0.141527	6.846296	4.673088	2.27E-05	0.00293	2.588413
ILMN_1821473	0.14145	6.241595	3.751173	0.000458	0.021893	-0.18822
ILMN_1662426	0.141433	11.20601	4.307395	7.70E-05	0.006655	1.454646
ILMN_1686152	-0.14141	7.40316	-4.08174	0.000161	0.011128	0.774952
ILMN_1747744	0.141	7.483208	4.014795	0.000199	0.012768	0.576597
ILMN_1666727	0.14088	7.476774	4.154949	0.000127	0.009421	0.99362

ILMN_3204734	0.140815	9.880486	3.548605	0.000853	0.032839	-0.75668
ILMN_3219340	0.14081	12.83704	3.511005	0.000955	0.035028	-0.86021
ILMN_3246608	0.140682	6.307201	3.637203	0.000651	0.0277	-0.51023
ILMN_2175112	-0.14064	6.505594	-3.54106	0.000872	0.033133	-0.7775
ILMN_1654653	0.140549	7.652321	5.538333	1.13E-06	0.000389	5.387142
ILMN_1786893	0.140339	9.273498	3.360402	0.001496	0.045325	-1.26827
ILMN_1741371	-0.14018	6.492369	-3.9852	0.000219	0.013631	0.489427
ILMN_1715273	-0.14017	9.627349	-4.16998	0.000121	0.009087	1.038755
ILMN_1669484	0.140056	9.247776	4.217714	0.000103	0.008241	1.18253
ILMN_1689327	0.139804	11.86812	5.004905	7.29E-06	0.001422	3.645601
ILMN_2407124	0.139741	6.874062	3.499223	0.00099	0.035741	-0.89251
ILMN_1737604	-0.13969	7.910983	-3.78866	0.000407	0.020302	-0.08112
ILMN_1798712	0.13965	6.692122	3.800621	0.000392	0.019868	-0.04683
ILMN_1765649	0.139577	7.80209	4.171214	0.00012	0.009073	1.04245
ILMN_2333107	-0.13944	8.586003	-4.17249	0.00012	0.009055	1.046288
ILMN_1772124	0.139399	7.905463	3.944809	0.000249	0.014858	0.370972
ILMN_1712231	-0.13929	9.444425	-3.48691	0.001027	0.036495	-0.92621
ILMN_2387599	0.139277	11.77284	4.751618	1.74E-05	0.002466	2.836411
ILMN_1804150	-0.13909	9.13232	-4.07502	0.000164	0.0113	0.754967
ILMN_3224290	0.139012	9.645384	3.322847	0.001671	0.048443	-1.36832
ILMN_1697817	-0.13895	7.647454	-3.68422	0.000563	0.024994	-0.37805
ILMN_2364131	0.138816	8.838955	5.082808	5.57E-06	0.001214	3.897064
ILMN_1660341	0.138728	8.027969	3.399388	0.001333	0.042678	-1.16367
ILMN_1702279	0.138561	7.827281	4.450664	4.79E-05	0.004816	1.894419
ILMN_1729987	0.138557	8.692391	4.74791	1.76E-05	0.002487	2.824669
ILMN_1722294	-0.13824	7.407387	-5.3076	2.54E-06	0.000718	4.628552
ILMN_1673111	0.138104	9.131493	3.709858	0.00052	0.023987	-0.30558
ILMN_1698673	-0.13809	7.12604	-5.06507	5.92E-06	0.001252	3.839722
ILMN_1701940	0.138073	7.001941	5.804244	4.38E-07	0.000194	6.269047
ILMN_1811836	-0.13804	8.139387	-3.36865	0.00146	0.044826	-1.24619
ILMN_2369785	0.138024	12.81444	5.504852	1.27E-06	0.000411	5.27664
ILMN_1727300	0.137983	7.468963	5.199442	3.71E-06	0.000899	4.275568
ILMN_1755974	0.137799	7.42346	3.402648	0.00132	0.042432	-1.15489
ILMN_1814966	0.137439	7.410336	4.212699	0.000105	0.008346	1.167388
ILMN_1747281	-0.13743	7.329939	-4.06427	0.00017	0.011592	0.723016
ILMN_2124802	0.137381	5.790428	3.68987	0.000554	0.024798	-0.36211
ILMN_1755077	-0.13733	11.37783	-3.90675	0.000281	0.015911	0.2599
ILMN_1752283	0.137263	7.497097	5.420497	1.71E-06	0.000522	4.998849
ILMN_1803988	-0.13717	10.12275	-3.4947	0.001003	0.035992	-0.90491
ILMN_1718285	0.137054	6.144673	3.667849	0.000592	0.026018	-0.42418
ILMN_1815885	0.136963	6.751586	4.165045	0.000123	0.009166	1.023919
ILMN_1676792	0.136934	12.62773	3.357984	0.001507	0.045529	-1.27473
ILMN_1718771	-0.13689	6.72125	-3.58077	0.000773	0.031105	-0.6676
ILMN_2337336	0.136858	6.485446	3.752413	0.000456	0.021839	-0.18468
ILMN_2317730	0.1365	7.618262	5.252908	3.08E-06	0.000792	4.449832
ILMN_1795228	-0.13648	11.03292	-3.379	0.001416	0.044216	-1.21846
ILMN_1722426	-0.13628	5.769089	-4.36667	6.33E-05	0.005854	1.63585
ILMN_1809957	0.136156	12.30113	7.000736	5.95E-09	1.58E-05	10.28316
ILMN_1804419	-0.13615	6.165495	-3.50775	0.000964	0.035112	-0.86915
ILMN_1794781	0.13611	6.908979	5.276932	2.83E-06	0.000774	4.528282
ILMN_2373266	-0.13596	8.809098	-4.96026	8.50E-06	0.001593	3.502003
ILMN_1702835	-0.13592	9.257488	-4.0138	0.0002	0.012779	0.573656
ILMN_2159453	-0.13552	8.276362	-3.85559	0.00033	0.017848	0.111507
ILMN_3288755	0.135497	6.588679	3.803443	0.000389	0.01978	-0.03873

ILMN_3180557	0.135472	6.933027	5.116973	4.94E-06	0.001114	4.007695
ILMN_1752884	0.135355	6.1438	3.553784	0.000839	0.032546	-0.74237
ILMN_1900270	-0.13517	6.401475	-3.37683	0.001425	0.044289	-1.22429
ILMN_1751615	-0.13509	8.943403	-3.4785	0.001053	0.036933	-0.94918
ILMN_1794213	0.134901	6.118721	3.477899	0.001055	0.036962	-0.95083
ILMN_1657204	0.134887	11.45826	3.508431	0.000962	0.035111	-0.86727
ILMN_1761069	-0.13487	7.418122	-3.54499	0.000862	0.033017	-0.76667
ILMN_1812327	-0.13486	6.350576	-4.3575	6.53E-05	0.005954	1.607751
ILMN_1731193	0.134828	6.411773	5.253807	3.07E-06	0.000792	4.452766
ILMN_1703142	-0.13471	6.363091	-4.2932	8.07E-05	0.006853	1.411394
ILMN_1658807	-0.13456	6.922589	-4.04272	0.000182	0.01217	0.659137
ILMN_2402806	0.134361	8.329239	4.605529	2.85E-05	0.003413	2.376263
ILMN_2403555	0.134145	6.899727	3.892628	0.000294	0.016316	0.218845
ILMN_1787932	-0.1339	6.155758	-3.49438	0.001004	0.035992	-0.90576
ILMN_1658015	-0.13354	7.671465	-3.86222	0.000323	0.01759	0.130678
ILMN_1653599	-0.13353	10.79695	-3.52722	0.000909	0.034118	-0.81565
ILMN_2167922	0.133517	11.34824	4.785775	1.55E-05	0.002232	2.944725
ILMN_1777444	-0.1335	7.396279	-4.67071	2.29E-05	0.002932	2.580926
ILMN_1656066	0.133497	9.395813	4.946671	8.91E-06	0.001617	3.458379
ILMN_1778803	-0.13348	8.24874	-4.93586	9.25E-06	0.001652	3.423682
ILMN_1792078	0.133274	9.804828	4.276182	8.53E-05	0.007124	1.359651
ILMN_1654064	-0.13315	7.089103	-4.65043	2.45E-05	0.003069	2.517138
ILMN_1808391	-0.13303	5.873943	-3.87471	0.000311	0.016991	0.166848
ILMN_1758633	0.132913	8.386412	3.498575	0.000991	0.035741	-0.89429
ILMN_1812461	0.132912	6.393518	4.848624	1.25E-05	0.001927	3.144711
ILMN_1721704	-0.13291	9.435152	-3.37876	0.001417	0.044216	-1.21911
ILMN_1757847	-0.13289	7.089486	-4.88867	1.09E-05	0.001811	3.272596
ILMN_2063925	0.132751	8.524528	4.000071	0.000209	0.013188	0.533188
ILMN_1775486	-0.1327	5.641129	-3.63525	0.000655	0.027737	-0.51569
ILMN_1806809	0.132642	6.996511	3.6185	0.000689	0.028788	-0.56254
ILMN_1808860	-0.13262	8.62686	-5.00737	7.23E-06	0.001422	3.65353
ILMN_1769734	-0.13238	9.050755	-3.89813	0.000289	0.016159	0.234841
ILMN_1651886	0.132079	9.567376	4.03667	0.000186	0.012245	0.64123
ILMN_2350574	0.132019	7.019527	3.376672	0.001426	0.044289	-1.2247
ILMN_1734827	0.131914	6.450167	4.250008	9.30E-05	0.007579	1.280227
ILMN_1715969	0.131778	7.124269	4.288041	8.21E-05	0.006902	1.395708
ILMN_1678052	-0.13137	7.657149	-3.31395	0.001715	0.04894	-1.39193
ILMN_1653896	0.131024	6.707074	3.563268	0.000815	0.032056	-0.71613
ILMN_1761722	0.130575	6.721795	4.505772	3.99E-05	0.004255	2.065141
ILMN_1784436	0.130122	7.171081	3.727791	0.000492	0.023093	-0.25473
ILMN_1747223	-0.13008	8.365961	-3.82273	0.000366	0.019071	0.016703
ILMN_1760143	0.129678	10.37174	4.386412	5.93E-05	0.005634	1.696448
ILMN_2411794	0.129596	8.508565	4.641216	2.53E-05	0.003133	2.488187
ILMN_1700337	0.129543	7.679403	3.793528	0.000401	0.020087	-0.06717
ILMN_1702866	0.12942	5.750363	5.420036	1.71E-06	0.000522	4.997331
ILMN_1748894	0.129285	8.536682	3.763282	0.000441	0.021449	-0.15369
ILMN_1764323	-0.1289	9.198453	-4.58203	3.09E-05	0.003583	2.302727
ILMN_2386100	0.128884	9.046575	3.710598	0.000519	0.023964	-0.30349
ILMN_1676759	0.128789	7.911027	4.310859	7.61E-05	0.006596	1.465206
ILMN_1771824	0.128721	6.442188	5.134613	4.65E-06	0.001068	4.064897
ILMN_1678949	0.128575	7.116892	4.427449	5.18E-05	0.005085	1.822753
ILMN_2174081	0.128539	7.115872	3.350455	0.001541	0.046176	-1.29483
ILMN_1697348	0.128284	8.438112	3.838904	0.000348	0.018491	0.063311
ILMN_1693145	0.128204	11.4118	3.904125	0.000283	0.015964	0.252274

ILMN_1714364	-0.12809	11.31818	-3.66388	0.0006	0.026271	-0.43535
ILMN_1672947	-0.12781	9.153308	-3.5133	0.000948	0.034911	-0.85392
ILMN_1773002	-0.12775	7.412498	-3.40056	0.001329	0.042649	-1.1605
ILMN_1754727	-0.12763	6.925514	-4.21813	0.000103	0.008241	1.183775
ILMN_1770911	0.127495	6.857643	4.33939	6.93E-05	0.006223	1.55233
ILMN_1697735	0.127458	9.890322	5.256236	3.04E-06	0.000792	4.460696
ILMN_1677402	-0.12735	6.360593	-3.34551	0.001563	0.0467	-1.30803
ILMN_1679195	0.127276	11.71869	4.131799	0.000137	0.010026	0.924271
ILMN_1741985	-0.12727	6.556949	-4.70614	2.03E-05	0.002732	2.692598
ILMN_1731354	0.127163	9.639258	4.179878	0.000117	0.008879	1.068496
ILMN_1802646	0.127105	5.629979	3.326603	0.001652	0.048147	-1.35834
ILMN_3233179	0.127101	7.616117	3.306308	0.001754	0.04984	-1.41216
ILMN_1778255	0.126946	7.104808	3.749329	0.00046	0.021988	-0.19347
ILMN_1671568	-0.12678	8.992622	-3.64606	0.000633	0.027241	-0.48541
ILMN_1674421	0.126449	8.956569	3.702633	0.000532	0.024369	-0.32603
ILMN_1775522	0.126307	9.771732	3.339125	0.001593	0.047297	-1.32504
ILMN_1769158	0.126127	8.019062	3.637174	0.000651	0.0277	-0.51031
ILMN_2328378	-0.12612	7.923599	-3.30907	0.001739	0.049521	-1.40485
ILMN_1784540	-0.12578	8.812635	-3.45349	0.001135	0.038458	-1.0173
ILMN_1787280	0.125731	8.156233	3.817671	0.000372	0.019231	0.002154
ILMN_2363591	-0.12554	9.224569	-4.20682	0.000107	0.008446	1.149656
ILMN_2369221	-0.12552	5.999184	-3.50327	0.000978	0.035401	-0.88143
ILMN_1771966	0.125184	10.57052	3.479415	0.00105	0.036901	-0.94669
ILMN_1727001	0.124946	7.703579	3.795114	0.000399	0.020061	-0.06262
ILMN_1757914	0.124906	9.621177	3.327122	0.00165	0.048147	-1.35696
ILMN_2169761	-0.1249	7.763599	-3.44171	0.001176	0.039438	-1.04929
ILMN_2225061	0.124888	5.399499	4.842531	1.27E-05	0.001949	3.125284
ILMN_1733875	0.124827	7.811225	3.929631	0.000261	0.015308	0.326616
ILMN_1774589	0.124805	6.94714	3.755986	0.000451	0.021749	-0.1745
ILMN_1669268	-0.12477	7.286341	-3.51944	0.000931	0.034679	-0.83703
ILMN_1665095	0.124573	7.455196	3.779234	0.00042	0.020723	-0.1081
ILMN_1657631	-0.12449	6.994022	-4.51994	3.80E-05	0.004109	2.109171
ILMN_2182704	-0.12446	9.554422	-4.31169	7.59E-05	0.006596	1.467727
ILMN_1731165	0.12446	5.676588	3.369585	0.001456	0.044742	-1.24369
ILMN_1729281	0.12443	7.419073	4.537472	3.58E-05	0.003963	2.163721
ILMN_1703316	0.124408	7.538053	3.574074	0.000789	0.031452	-0.68619
ILMN_1795639	0.124236	9.320822	3.676455	0.000577	0.025468	-0.39995
ILMN_2047676	-0.12423	7.323166	-3.3661	0.001471	0.044915	-1.25303
ILMN_1694759	0.124112	7.99306	4.184746	0.000115	0.008798	1.08314
ILMN_1711823	0.124058	8.071418	3.757676	0.000449	0.021665	-0.16968
ILMN_1755664	0.123941	6.608051	5.236112	3.26E-06	0.000815	4.395042
ILMN_1673522	-0.12384	8.374233	-3.57427	0.000789	0.031452	-0.68565
ILMN_1733511	0.123742	9.79902	4.535455	3.61E-05	0.003969	2.157442
ILMN_1678966	0.123701	11.14048	3.364348	0.001479	0.044998	-1.25771
ILMN_1813657	0.123648	7.18278	4.023809	0.000194	0.012553	0.60321
ILMN_1779163	-0.12359	8.642296	-3.88734	0.000299	0.016537	0.203479
ILMN_1678054	-0.12351	7.81865	-3.37466	0.001434	0.044352	-1.23011
ILMN_1778488	-0.12336	7.39079	-4.53498	3.61E-05	0.003969	2.155951
ILMN_2367258	0.122871	5.760206	3.375038	0.001433	0.044346	-1.22908
ILMN_1728626	0.122818	8.320201	3.908833	0.000279	0.015832	0.265979
ILMN_1759595	0.122777	7.280271	5.690125	6.58E-07	0.000264	5.8897
ILMN_1740197	0.122742	6.46239	4.819811	1.38E-05	0.002038	3.052922
ILMN_1672843	-0.12274	8.3593	-4.0634	0.00017	0.011592	0.720448
ILMN_2219134	-0.1227	12.86068	-4.2054	0.000108	0.00845	1.145364

ILMN_1746426	0.122563	9.547126	4.438506	4.99E-05	0.004947	1.856869
ILMN_1743662	-0.12256	6.300212	-4.60933	2.82E-05	0.003382	2.388183
ILMN_1706779	0.122445	7.750245	3.382088	0.001403	0.043982	-1.21017
ILMN_1802338	-0.1224	6.528872	-4.90325	1.03E-05	0.001773	3.319217
ILMN_1661170	0.122363	11.5488	4.063984	0.00017	0.011592	0.722178
ILMN_1665300	-0.12235	7.015094	-5.46894	1.44E-06	0.000456	5.158272
ILMN_2063500	0.122297	7.628167	4.613823	2.77E-05	0.003342	2.402245
ILMN_1679483	0.122276	8.198779	3.945152	0.000249	0.014858	0.371974
ILMN_1757343	0.122275	10.7549	4.379265	6.07E-05	0.00571	1.674501
ILMN_1774196	0.122274	9.275008	3.926258	0.000264	0.015412	0.316772
ILMN_1674661	-0.12214	10.48293	-3.37518	0.001432	0.044346	-1.22869
ILMN_1778240	0.122038	8.455276	3.534691	0.000889	0.033576	-0.79507
ILMN_1670948	0.121991	8.812808	4.069337	0.000167	0.01144	0.738072
ILMN_1722089	-0.12197	7.619406	-3.68774	0.000557	0.02482	-0.36812
ILMN_2358914	0.121956	6.50809	4.187644	0.000114	0.008774	1.091863
ILMN_1723467	-0.12185	11.41865	-3.99908	0.00021	0.013206	0.530273
ILMN_1784227	0.121486	8.89894	4.863952	1.18E-05	0.001881	3.193614
ILMN_1823112	0.121208	6.26093	4.649588	2.46E-05	0.003069	2.514488
ILMN_1803773	0.121198	6.123491	4.453176	4.75E-05	0.004816	1.902183
ILMN_1801767	-0.12117	6.987505	-3.32824	0.001644	0.048047	-1.35399
ILMN_1711909	0.121098	7.403274	3.420775	0.001251	0.041004	-1.10598
ILMN_3240187	0.12094	9.69429	4.07302	0.000165	0.01135	0.749013
ILMN_1793743	-0.1209	8.542338	-4.04217	0.000183	0.01217	0.657517
ILMN_1686135	-0.12087	8.359989	-3.38268	0.001401	0.043982	-1.20858
ILMN_1655952	0.120472	9.16614	3.733743	0.000483	0.022857	-0.23782
ILMN_1801600	0.120299	8.147344	3.461924	0.001107	0.037926	-0.99437
ILMN_1708787	0.120235	6.52756	3.567809	0.000804	0.031909	-0.70356
ILMN_1755850	0.120167	5.583099	3.512599	0.00095	0.034911	-0.85583
ILMN_1706960	-0.12005	6.299473	-3.76875	0.000433	0.021229	-0.13808
ILMN_1783702	-0.11993	7.721322	-3.36173	0.00149	0.045292	-1.26471
ILMN_1768194	-0.11992	7.726417	-4.63798	2.56E-05	0.003136	2.478018
ILMN_1740430	0.119675	6.808733	4.413604	5.42E-05	0.005269	1.780086
ILMN_1665049	-0.11963	8.704278	-4.53736	3.59E-05	0.003963	2.16338
ILMN_1712944	-0.11962	9.416916	-3.82354	0.000365	0.019051	0.019052
ILMN_1734231	0.119445	9.752719	3.701724	0.000534	0.024369	-0.3286
ILMN_1815656	0.119427	8.904437	3.467802	0.001087	0.037641	-0.97836
ILMN_1754865	0.119325	9.424259	4.313832	7.54E-05	0.006581	1.474273
ILMN_2392080	-0.11932	7.594627	-3.60323	0.000722	0.029718	-0.60514
ILMN_1695797	0.119236	8.588752	3.496548	0.000998	0.03586	-0.89984
ILMN_1759991	0.11923	8.673025	4.438303	4.99E-05	0.004947	1.856242
ILMN_1745513	0.11922	7.305104	3.396457	0.001345	0.042812	-1.17156
ILMN_1707199	0.119147	7.796457	4.3319	7.10E-05	0.006329	1.529435
ILMN_1738103	0.119133	9.375674	3.375861	0.001429	0.044346	-1.22688
ILMN_1698015	0.118924	6.189212	4.514407	3.87E-05	0.004147	2.091968
ILMN_1684591	0.118873	7.380275	4.005224	0.000205	0.012996	0.548372
ILMN_1762439	-0.11863	7.006073	-3.75867	0.000447	0.021629	-0.16685
ILMN_3256325	0.118552	7.767774	3.36104	0.001493	0.045319	-1.26656
ILMN_1844692	-0.1182	8.90904	-3.34145	0.001582	0.047137	-1.31883
ILMN_1671619	0.117859	6.073866	4.441219	4.94E-05	0.004927	1.865243
ILMN_2405009	-0.11777	9.694206	-3.50819	0.000963	0.035111	-0.86793
ILMN_1657129	-0.11769	8.061768	-3.72596	0.000495	0.023192	-0.25992
ILMN_3237209	0.117677	6.406315	4.673235	2.27E-05	0.00293	2.588875
ILMN_1681135	0.117592	6.153082	5.421089	1.71E-06	0.000522	5.000794
ILMN_1743806	-0.11757	7.173525	-3.57027	0.000798	0.031744	-0.69673

ILMN_1700733	-0.11753	8.020116	-3.83494	0.000352	0.018636	0.05187
ILMN_1720850	-0.11746	7.84599	-4.18015	0.000117	0.008879	1.069303
ILMN_1790461	-0.1173	10.46785	-3.99434	0.000213	0.01331	0.516323
ILMN_2389155	-0.11713	9.368498	-4.20725	0.000107	0.008446	1.150944
ILMN_1671603	-0.11707	9.146976	-5.97435	2.39E-07	0.000125	6.836506
ILMN_2197247	0.116777	7.95462	4.181033	0.000116	0.008879	1.07197
ILMN_1702198	0.116701	7.340192	4.470886	4.48E-05	0.00461	1.956968
ILMN_2278819	-0.11652	6.91849	-4.11815	0.000143	0.010347	0.883455
ILMN_1808047	-0.1164	6.950668	-3.48362	0.001037	0.036654	-0.93521
ILMN_1705682	0.116254	5.980359	3.780066	0.000418	0.020699	-0.10572
ILMN_1688011	0.116192	7.523639	4.35094	6.67E-05	0.006053	1.58767
ILMN_2175114	-0.11611	5.816607	-3.64742	0.000631	0.027218	-0.48158
ILMN_3238269	0.116015	8.443412	4.164159	0.000123	0.009166	1.021257
ILMN_1737611	0.115991	6.66995	4.695003	2.11E-05	0.002794	2.657472
ILMN_1730077	0.115958	8.436897	4.598678	2.92E-05	0.003457	2.354813
ILMN_1781803	0.115932	6.925614	4.085971	0.000159	0.011033	0.787527
ILMN_1813344	0.11573	8.433726	3.326378	0.001653	0.048147	-1.35894
ILMN_2053567	0.115699	7.403784	4.117346	0.000143	0.010352	0.881067
ILMN_3307786	0.115436	7.343689	4.093486	0.000155	0.010908	0.809901
ILMN_3225300	-0.11543	9.027645	-3.9325	0.000259	0.015239	0.335007
ILMN_1722522	-0.11534	7.330681	-4.62744	2.65E-05	0.003215	2.444955
ILMN_1695311	-0.11528	6.497101	-3.64322	0.000639	0.027399	-0.49336
ILMN_1791097	0.115081	7.561567	3.41466	0.001274	0.04148	-1.1225
ILMN_1683916	-0.11502	8.270671	-3.54254	0.000868	0.033115	-0.77343
ILMN_1819783	-0.11444	7.253281	-3.31477	0.001711	0.04894	-1.38973
ILMN_1768930	0.114359	10.04637	3.760925	0.000444	0.02153	-0.16041
ILMN_1654289	0.114309	8.02617	3.924092	0.000266	0.015492	0.310451
ILMN_2399264	0.114299	6.216862	4.113955	0.000145	0.010423	0.87094
ILMN_1781791	-0.11418	7.244427	-3.72503	0.000497	0.023197	-0.26258
ILMN_1737360	0.114132	5.933497	3.80403	0.000388	0.01978	-0.03704
ILMN_1809488	-0.11395	10.62056	-3.56376	0.000814	0.032056	-0.71477
ILMN_1729314	-0.11392	6.349821	-3.54129	0.000872	0.033133	-0.77686
ILMN_1692748	-0.1139	7.544309	-3.48949	0.001019	0.036364	-0.91917
ILMN_3249742	0.113839	6.037584	3.72818	0.000492	0.023093	-0.25362
ILMN_1669308	0.113745	7.241029	3.979611	0.000223	0.013776	0.472999
ILMN_1750636	0.113644	11.9311	3.359878	0.001498	0.045355	-1.26966
ILMN_2043109	0.113253	7.624979	4.264225	8.88E-05	0.007286	1.32334
ILMN_1659800	-0.11322	5.620496	-3.69026	0.000553	0.024798	-0.36101
ILMN_1806010	-0.11303	6.587764	-4.17866	0.000117	0.008895	1.064842
ILMN_1758339	0.113025	6.375826	3.350225	0.001542	0.046176	-1.29545
ILMN_1679324	0.113	9.648037	3.529087	0.000904	0.033963	-0.8105
ILMN_1658290	0.112868	7.569182	4.086318	0.000158	0.011033	0.788559
ILMN_1778242	0.11285	10.31384	3.483068	0.001039	0.036654	-0.93671
ILMN_1662161	0.112786	6.398688	4.035004	0.000187	0.012264	0.636302
ILMN_1693438	-0.11276	7.133441	-3.91728	0.000272	0.015534	0.290582
ILMN_1658677	0.11269	5.559248	3.803652	0.000389	0.01978	-0.03813
ILMN_1738420	0.11269	5.652472	4.185911	0.000115	0.008798	1.086646
ILMN_1657058	-0.11248	5.838526	-3.97251	0.000228	0.013966	0.452142
ILMN_1664025	0.112449	6.58128	3.455467	0.001128	0.038349	-1.01194
ILMN_2397024	-0.11204	9.407902	-3.69263	0.000549	0.024798	-0.35431
ILMN_1675577	0.111843	7.180331	4.477396	4.38E-05	0.004538	1.977129
ILMN_1772677	0.111783	8.256293	4.271424	8.67E-05	0.007193	1.345196
ILMN_1811560	0.111641	8.083334	3.801635	0.000391	0.019839	-0.04392
ILMN_1812441	0.111605	8.001096	3.546789	0.000857	0.032877	-0.7617

ILMN_2198393	-0.11151	8.687471	-3.77515	0.000425	0.020898	-0.11977
ILMN_1671326	0.111497	6.730258	3.788575	0.000407	0.020302	-0.08136
ILMN_2360784	0.111433	9.320021	3.321264	0.001678	0.048546	-1.37252
ILMN_1798164	-0.1114	9.01414	-3.44927	0.001149	0.038809	-1.02878
ILMN_1683096	-0.11129	7.129923	-3.55769	0.000829	0.032309	-0.73158
ILMN_1779639	-0.11116	6.887925	-3.53881	0.000878	0.033234	-0.78372
ILMN_1676955	-0.11113	7.835196	-3.78111	0.000417	0.020661	-0.10274
ILMN_1711102	-0.11092	7.192119	-3.75296	0.000455	0.021837	-0.18311
ILMN_2281186	-0.11091	5.81226	-4.4646	4.57E-05	0.00468	1.937528
ILMN_2352121	-0.11081	9.799415	-3.70529	0.000528	0.024295	-0.31851
ILMN_3251742	0.110777	7.084747	3.483081	0.001039	0.036654	-0.93668
ILMN_1691341	0.110749	6.484653	3.727812	0.000492	0.023093	-0.25467
ILMN_1687275	0.110584	8.405381	3.847352	0.000339	0.018174	0.087695
ILMN_2323633	0.110566	8.173019	3.895057	0.000292	0.016243	0.225905
ILMN_1803906	-0.11045	6.160431	-3.37502	0.001433	0.044346	-1.22913
ILMN_1725079	0.11042	7.7611	3.690842	0.000552	0.024798	-0.35936
ILMN_1669142	0.11004	7.797861	3.694115	0.000546	0.024785	-0.35011
ILMN_2094952	-0.11002	6.927402	-3.63515	0.000655	0.027737	-0.51597
ILMN_1806937	0.109829	8.86122	4.330333	7.14E-05	0.006341	1.524645
ILMN_1712859	-0.10944	6.573917	-4.08917	0.000157	0.010993	0.797047
ILMN_1685286	0.108951	6.625015	3.782909	0.000415	0.020575	-0.09759
ILMN_1777528	0.108802	8.948298	3.996399	0.000211	0.013271	0.522373
ILMN_2242068	0.108732	6.378102	3.517001	0.000938	0.034825	-0.84374
ILMN_2181445	-0.10869	9.481545	-4.32975	7.15E-05	0.006341	1.522877
ILMN_1691112	-0.10861	7.70707	-3.68323	0.000565	0.025005	-0.38084
ILMN_1741736	-0.10856	8.045154	-3.84463	0.000342	0.018245	0.079826
ILMN_2352724	-0.10828	7.306223	-3.55057	0.000847	0.032718	-0.75126
ILMN_3261197	0.108269	10.5973	3.367275	0.001466	0.044915	-1.24988
ILMN_2058141	0.108209	11.87573	3.704362	0.000529	0.0243	-0.32114
ILMN_2144791	-0.10817	7.125175	-3.84095	0.000346	0.018403	0.069227
ILMN_2341467	0.108098	9.430058	3.773711	0.000427	0.020962	-0.1239
ILMN_1694504	0.108085	7.615096	3.590374	0.000751	0.030491	-0.64093
ILMN_1704500	-0.10804	6.769832	-3.56476	0.000812	0.032053	-0.712
ILMN_2124361	0.107929	6.170762	4.081002	0.000161	0.011128	0.772744
ILMN_1728224	0.107888	7.247527	3.320295	0.001683	0.048567	-1.37509
ILMN_2073010	0.107887	8.622636	3.416923	0.001266	0.041319	-1.11639
ILMN_1904135	0.107822	6.387132	3.634787	0.000656	0.027737	-0.517
ILMN_2346727	-0.10777	7.1026	-3.68242	0.000566	0.025036	-0.38314
ILMN_1755221	-0.10769	7.623086	-3.9651	0.000234	0.014174	0.430392
ILMN_1732053	0.107534	10.0834	3.339375	0.001592	0.047297	-1.32437
ILMN_1793267	0.107352	6.14238	3.35162	0.001535	0.046067	-1.29172
ILMN_1773066	-0.10693	7.448939	-3.39876	0.001336	0.042702	-1.16537
ILMN_1698166	-0.10678	7.116865	-4.02243	0.000194	0.012553	0.599142
ILMN_3244526	0.106686	7.229915	3.406511	0.001305	0.042165	-1.14448
ILMN_1770667	-0.10665	7.11012	-3.65104	0.000624	0.027053	-0.47144
ILMN_1761828	0.106447	8.61968	3.621738	0.000682	0.028587	-0.5535
ILMN_1702231	-0.10625	6.19729	-3.35878	0.001503	0.045462	-1.27261
ILMN_1867321	0.106126	6.243638	4.930747	9.41E-06	0.001673	3.407302
ILMN_1717337	-0.10572	10.11213	-3.86899	0.000317	0.017273	0.150263
ILMN_1775753	0.10554	6.932964	3.567036	0.000806	0.03191	-0.7057
ILMN_1788095	0.105261	6.731435	3.722433	0.000501	0.023352	-0.26993
ILMN_1749345	-0.10518	9.090378	-3.41393	0.001277	0.041531	-1.12448
ILMN_1735402	0.105141	7.127156	4.278508	8.47E-05	0.007087	1.366719
ILMN_1701655	0.105136	7.201903	3.812669	0.000378	0.019476	-0.01223

ILMN_1721344	-0.10505	6.880078	-3.64175	0.000642	0.027488	-0.49747
ILMN_1713406	0.105018	8.336282	3.920207	0.000269	0.015534	0.299118
ILMN_1767766	0.104824	9.131113	3.807658	0.000384	0.019694	-0.02662
ILMN_1811178	-0.10471	7.144893	-3.63926	0.000647	0.027631	-0.50448
ILMN_2083567	0.104483	6.215335	3.334859	0.001613	0.047559	-1.33639
ILMN_1673172	0.104229	6.986877	3.637125	0.000651	0.0277	-0.51045
ILMN_2352190	0.104157	6.39338	3.688705	0.000556	0.024798	-0.36539
ILMN_2412761	-0.10414	6.307542	-3.92087	0.000269	0.015534	0.301062
ILMN_3309468	0.10385	6.141024	4.500567	4.06E-05	0.00429	2.04898
ILMN_1766195	-0.10384	7.173367	-3.717	0.000509	0.023652	-0.28534
ILMN_1738883	-0.10366	6.05132	-3.61945	0.000687	0.028753	-0.5599
ILMN_1732705	0.103659	9.413949	3.860027	0.000326	0.017656	0.12433
ILMN_2150284	0.103456	7.344529	4.288491	8.20E-05	0.006902	1.397077
ILMN_1764873	-0.10329	5.920363	-3.76566	0.000438	0.021343	-0.1469
ILMN_1658144	-0.10323	7.851685	-4.67203	2.28E-05	0.00293	2.585089
ILMN_3247906	0.103035	8.076963	3.43884	0.001186	0.03959	-1.05708
ILMN_1688526	-0.10265	11.46092	-3.33296	0.001622	0.047632	-1.34145
ILMN_1684755	0.102469	6.098881	3.712874	0.000516	0.023889	-0.29704
ILMN_2327276	0.102459	10.67604	4.109121	0.000147	0.010521	0.856514
ILMN_2336109	0.102059	7.715795	4.129083	0.000138	0.010087	0.916146
ILMN_1682354	0.101986	6.46123	4.919535	9.78E-06	0.001712	3.371369
ILMN_1789575	-0.10181	6.965427	-4.29027	8.15E-05	0.006902	1.402486
ILMN_1722583	0.101468	7.463456	3.504267	0.000975	0.035332	-0.87869
ILMN_1689720	-0.10139	6.837395	-3.81371	0.000377	0.019442	-0.00923
ILMN_1745421	-0.10136	7.696077	-3.40653	0.001305	0.042165	-1.14444
ILMN_1688160	-0.10136	6.519732	-3.96819	0.000231	0.01406	0.439474
ILMN_1688853	0.101327	7.418838	4.18768	0.000114	0.008774	1.091972
ILMN_1666376	0.101309	7.528633	3.316112	0.001704	0.04894	-1.38618
ILMN_2396786	-0.10131	7.295269	-4.01067	0.000202	0.012866	0.564434
ILMN_1660577	-0.10115	11.44089	-3.64364	0.000638	0.027398	-0.49218
ILMN_1660232	0.101072	9.078017	3.323601	0.001667	0.048418	-1.36631
ILMN_1773080	-0.10104	12.90952	-3.5396	0.000876	0.033207	-0.78153
ILMN_1714623	0.101015	9.620491	3.662811	0.000602	0.026324	-0.43836
ILMN_2349459	0.10088	8.499249	3.348595	0.001549	0.046318	-1.2998
ILMN_1722532	-0.10088	7.896159	-3.68538	0.000561	0.024936	-0.37478
ILMN_2370464	0.100737	7.256077	3.314346	0.001713	0.04894	-1.39087
ILMN_3249501	0.100615	6.204479	4.164066	0.000123	0.009166	1.02098
ILMN_3238560	-0.10055	8.406128	-3.33944	0.001591	0.047297	-1.32419
ILMN_1782094	-0.10027	7.267839	-4.02281	0.000194	0.012553	0.600251
ILMN_1668426	-0.10004	6.007205	-4.09431	0.000154	0.010901	0.812354
ILMN_2095133	0.100021	8.352404	4.03933	0.000184	0.012201	0.649103
ILMN_1726107	0.099957	7.107832	4.702159	2.06E-05	0.002748	2.680049
ILMN_2357086	0.099948	5.587509	3.398261	0.001338	0.042702	-1.1667
ILMN_1818617	0.09988	6.776722	3.360539	0.001495	0.045325	-1.2679
ILMN_1719988	0.099841	6.098033	3.562142	0.000818	0.032056	-0.71925
ILMN_1677452	0.099558	8.020636	3.546757	0.000857	0.032877	-0.76178
ILMN_1778032	0.099459	8.78322	3.594463	0.000742	0.030188	-0.62955
ILMN_3246801	0.099006	6.258492	4.436944	5.02E-05	0.004955	1.852047
ILMN_1753575	0.098891	6.485391	3.876786	0.000309	0.016928	0.172866
ILMN_1672042	0.098724	7.272439	3.410424	0.00129	0.041926	-1.13393
ILMN_2380801	0.098702	6.182506	3.510175	0.000957	0.035078	-0.86249
ILMN_1691980	-0.09862	6.551937	-3.4983	0.000992	0.035741	-0.89503
ILMN_2344079	0.09857	6.244167	3.840913	0.000346	0.018403	0.069108
ILMN_1803819	-0.09856	9.694341	-4.33494	7.03E-05	0.006282	1.53871

ILMN_1798659	-0.09851	8.113755	-3.38289	0.0014	0.043982	-1.20802
ILMN_1694486	-0.09839	6.609586	-3.98166	0.000222	0.013735	0.479006
ILMN_1671516	-0.09837	7.946452	-3.76597	0.000437	0.021343	-0.14601
ILMN_1660844	-0.09829	8.236984	-3.57318	0.000791	0.031501	-0.68866
ILMN_1699987	-0.09814	6.630318	-3.58789	0.000757	0.030654	-0.64783
ILMN_2098947	0.097789	6.221145	3.98461	0.000219	0.013631	0.487689
ILMN_1760682	0.09777	6.726168	3.586475	0.00076	0.030714	-0.65176
ILMN_1729188	0.097728	6.18162	3.316423	0.001702	0.04894	-1.38536
ILMN_1713732	0.097719	7.408825	3.513201	0.000949	0.034911	-0.85418
ILMN_1687751	0.097687	6.294353	3.40336	0.001318	0.042415	-1.15297
ILMN_2367141	0.097646	6.138928	4.486616	4.25E-05	0.004454	2.005701
ILMN_3307648	0.0975	10.48895	4.152398	0.000128	0.009459	0.985967
ILMN_1811301	0.097492	7.229325	4.025661	0.000192	0.012526	0.60868
ILMN_1670377	0.097453	6.558711	3.575401	0.000786	0.031452	-0.68251
ILMN_3194087	-0.09724	6.69329	-3.39717	0.001342	0.042762	-1.16965
ILMN_1796464	-0.09707	6.732443	-3.97836	0.000224	0.013806	0.46933
ILMN_1730631	0.097025	7.288177	3.647719	0.00063	0.027218	-0.48075
ILMN_1750008	0.09699	7.387539	3.46873	0.001084	0.03761	-0.97583
ILMN_1721241	0.096955	6.310762	3.82963	0.000358	0.018776	0.036578
ILMN_1676745	0.09691	7.911071	4.256767	9.10E-05	0.007431	1.300714
ILMN_1690761	0.09686	8.819047	3.88623	0.0003	0.016569	0.200264
ILMN_2052163	-0.09682	9.130896	-3.44622	0.00116	0.03905	-1.03707
ILMN_1722981	-0.09665	6.391925	-3.48958	0.001019	0.036364	-0.9189
ILMN_1844905	-0.09641	6.367295	-3.56443	0.000813	0.032053	-0.71292
ILMN_2161556	0.096378	6.790024	4.515731	3.86E-05	0.004141	2.096081
ILMN_2049417	0.096302	5.960285	3.513685	0.000947	0.034911	-0.85285
ILMN_2156953	-0.09594	7.779871	-4.53731	3.59E-05	0.003963	2.163221
ILMN_1694385	0.095808	11.85941	3.913619	0.000275	0.015619	0.279917
ILMN_1674698	0.095779	7.212241	4.085547	0.000159	0.011033	0.786263
ILMN_1698940	0.095768	13.88241	3.498004	0.000993	0.035741	-0.89585
ILMN_1812281	0.09549	5.969044	3.69548	0.000544	0.024713	-0.34626
ILMN_1713450	0.095469	9.115566	3.437299	0.001191	0.039657	-1.06126
ILMN_1652412	-0.09542	6.524361	-3.71248	0.000516	0.023889	-0.29815
ILMN_1792518	-0.09535	8.309534	-3.55593	0.000834	0.032408	-0.73643
ILMN_1738369	0.095124	10.02442	4.042827	0.000182	0.01217	0.659455
ILMN_2222317	-0.09496	7.504694	-3.9435	0.00025	0.014894	0.36713
ILMN_1710652	0.094928	6.22486	3.381187	0.001407	0.044059	-1.21259
ILMN_2290068	-0.09481	5.847125	-4.13847	0.000134	0.009854	0.944225
ILMN_1803564	-0.0948	8.155443	-3.63507	0.000655	0.027737	-0.51621
ILMN_2402805	0.094731	7.960192	3.565411	0.00081	0.032031	-0.7102
ILMN_1671374	0.09456	9.250392	3.531371	0.000898	0.03384	-0.80421
ILMN_1688070	0.094556	7.090268	3.431278	0.001213	0.040162	-1.07757
ILMN_1753980	-0.09452	7.043688	-4.49934	4.07E-05	0.004294	2.045159
ILMN_2058512	-0.09435	8.228484	-3.73553	0.000481	0.022762	-0.23273
ILMN_1698680	0.094204	6.402033	4.091813	0.000156	0.010944	0.804916
ILMN_1796900	-0.09402	7.310991	-3.77876	0.00042	0.020724	-0.10947
ILMN_2363586	-0.09381	10.02952	-4.03585	0.000186	0.012254	0.638803
ILMN_1701604	-0.09366	6.390781	-3.57887	0.000778	0.031213	-0.67289
ILMN_1684258	-0.09362	13.89482	-4.84762	1.25E-05	0.001927	3.141521
ILMN_2412214	-0.09358	5.800786	-3.87785	0.000308	0.016904	0.175947
ILMN_1713936	-0.09346	6.452103	-3.82175	0.000367	0.019072	0.013894
ILMN_1769191	0.093225	12.14777	3.515665	0.000942	0.034873	-0.84741
ILMN_1727880	0.093119	5.588242	3.516194	0.00094	0.034855	-0.84596
ILMN_1663489	-0.09301	7.630736	-3.92091	0.000269	0.015534	0.301156

ILMN_1656706	0.092822	5.463017	4.34745	6.75E-05	0.006091	1.576987
ILMN_1795711	0.092655	5.933911	3.896968	0.00029	0.016171	0.231457
ILMN_2147435	-0.0926	8.722297	-3.69223	0.00055	0.024798	-0.35543
ILMN_1663772	0.092454	6.030705	3.654784	0.000617	0.026811	-0.46092
ILMN_1840934	0.092274	6.305919	3.423421	0.001241	0.040845	-1.09883
ILMN_1732049	0.092268	7.307442	3.3788	0.001417	0.044216	-1.219
ILMN_1698404	-0.09227	6.749597	-3.39032	0.00137	0.043317	-1.18805
ILMN_3240962	0.092263	6.771073	3.5193	0.000931	0.034679	-0.83742
ILMN_1682062	0.092234	6.578357	3.546765	0.000857	0.032877	-0.76176
ILMN_2389151	-0.0922	10.55855	-3.38591	0.001388	0.043766	-1.19992
ILMN_1800447	-0.09187	8.299848	-4.12091	0.000142	0.010277	0.891718
ILMN_1791884	0.091862	6.724049	3.776533	0.000423	0.020838	-0.11583
ILMN_3248057	0.091861	6.076667	4.950503	8.79E-06	0.001614	3.470677
ILMN_1681798	0.091777	6.133635	3.65202	0.000622	0.027005	-0.46868
ILMN_2296843	0.091553	7.075077	3.341793	0.00158	0.047131	-1.31793
ILMN_2225887	0.091535	12.82245	3.81848	0.000371	0.019211	0.004479
ILMN_1661409	0.091431	6.498988	3.794714	0.0004	0.020061	-0.06377
ILMN_2153485	-0.09108	6.056836	-4.26476	8.86E-05	0.007286	1.324969
ILMN_1704398	0.090493	5.50716	3.766217	0.000437	0.021343	-0.1453
ILMN_1809212	-0.09037	6.2086	-3.63104	0.000663	0.028014	-0.52748
ILMN_2274531	0.09031	6.273664	3.929466	0.000262	0.015308	0.326134
ILMN_1741391	-0.09025	7.728987	-3.82714	0.000361	0.018895	0.029415
ILMN_3262348	0.090213	6.846546	3.628027	0.000669	0.028179	-0.53591
ILMN_1767111	0.089716	6.715365	3.452807	0.001137	0.038458	-1.01917
ILMN_1666019	0.089581	6.638663	3.670931	0.000587	0.025871	-0.41551
ILMN_2342579	0.089501	5.564237	3.482111	0.001042	0.036685	-0.93932
ILMN_1652819	0.089493	6.465272	3.467778	0.001087	0.037641	-0.97843
ILMN_1665060	-0.08948	6.251033	-3.44563	0.001162	0.03908	-1.03865
ILMN_3235168	0.089228	8.770873	3.321528	0.001677	0.048546	-1.37182
ILMN_1741398	0.089212	6.951261	3.470842	0.001078	0.037448	-0.97008
ILMN_1771238	-0.08904	7.869939	-4.01338	0.0002	0.012779	0.572436
ILMN_2193498	-0.08886	6.518431	-3.61054	0.000706	0.029332	-0.58475
ILMN_1677305	0.088838	7.166174	3.452696	0.001138	0.038458	-1.01947
ILMN_1661307	0.088734	5.799211	3.905069	0.000283	0.015964	0.255021
ILMN_2098616	-0.08859	6.798537	-3.33455	0.001614	0.047559	-1.3372
ILMN_1771738	-0.08848	7.554359	-3.50964	0.000959	0.035098	-0.86396
ILMN_1789535	0.088435	6.954984	4.221496	0.000102	0.008185	1.193952
ILMN_3238402	0.088432	5.941097	3.704776	0.000529	0.0243	-0.31997
ILMN_1680703	0.08834	10.15744	4.640918	2.53E-05	0.003133	2.48725
ILMN_1781001	-0.0881	6.255321	-3.34503	0.001565	0.046724	-1.30929
ILMN_1682335	-0.0879	6.310612	-3.68856	0.000556	0.024798	-0.36582
ILMN_2111918	-0.0879	8.222654	-3.30673	0.001751	0.04982	-1.41104
ILMN_1789618	-0.08776	6.918948	-3.43868	0.001186	0.03959	-1.05751
ILMN_2089340	-0.08776	6.555807	-3.5417	0.000871	0.033133	-0.77575
ILMN_1812898	0.087652	5.747437	3.437522	0.00119	0.039657	-1.06065
ILMN_2110496	-0.08759	6.781827	-4.29689	7.97E-05	0.006787	1.422636
ILMN_2280731	-0.08733	6.640289	-3.64107	0.000643	0.027512	-0.4994
ILMN_1765060	-0.08709	8.299388	-3.39352	0.001357	0.043027	-1.17946
ILMN_1729167	-0.08709	7.546048	-4.40317	5.61E-05	0.005419	1.747976
ILMN_1675055	0.086687	7.704113	3.919883	0.00027	0.015534	0.298174
ILMN_1683044	-0.08666	8.296903	-3.32379	0.001666	0.048418	-1.36582
ILMN_1656145	0.086635	10.027	3.355723	0.001517	0.045753	-1.28077
ILMN_1688435	-0.08655	6.027814	-3.66478	0.000598	0.026232	-0.43283
ILMN_1792860	0.086297	6.301839	4.089169	0.000157	0.010993	0.797044

ILMN_1672122	0.086186	5.998331	3.425486	0.001234	0.040665	-1.09324
ILMN_1743643	0.085852	6.202437	3.547171	0.000856	0.032877	-0.76064
ILMN_1688534	0.08559	7.514029	3.420846	0.001251	0.041004	-1.10579
ILMN_3289631	-0.0849	5.915389	-3.64618	0.000633	0.027241	-0.48506
ILMN_1706553	0.084638	9.101854	3.479934	0.001049	0.036887	-0.94527
ILMN_1718042	0.084435	6.34898	3.437144	0.001192	0.039657	-1.06168
ILMN_2081682	0.084345	7.750483	3.799961	0.000393	0.01988	-0.04872
ILMN_1719763	0.084344	5.881417	4.115445	0.000144	0.010394	0.875391
ILMN_1725175	-0.08425	6.93202	-4.19078	0.000113	0.008743	1.101295
ILMN_1698715	-0.08416	6.978506	-3.65688	0.000613	0.026706	-0.45502
ILMN_1739454	-0.08364	7.317376	-4.36706	6.33E-05	0.005854	1.637055
ILMN_1685125	0.083364	6.304599	4.206249	0.000107	0.008446	1.147927
ILMN_1727361	0.083351	8.569409	3.523255	0.00092	0.034417	-0.82655
ILMN_1893764	0.083263	6.633854	3.45882	0.001117	0.038166	-1.00282
ILMN_1790354	-0.08288	10.19535	-3.39948	0.001333	0.042678	-1.16343
ILMN_1673944	0.082811	10.05075	3.386617	0.001385	0.043715	-1.19801
ILMN_1723846	0.082361	7.409379	3.558781	0.000827	0.032266	-0.72855
ILMN_1722742	-0.08234	6.338646	-3.33293	0.001622	0.047632	-1.34152
ILMN_1715546	0.082067	6.674725	3.374367	0.001436	0.044352	-1.23088
ILMN_1828216	0.081858	6.239727	3.471035	0.001077	0.037448	-0.96955
ILMN_1726025	0.081766	7.314399	3.608685	0.00071	0.029463	-0.58994
ILMN_1651642	0.081748	6.414432	3.383956	0.001396	0.0439	-1.20516
ILMN_2279217	-0.08169	6.027258	-4.25998	9.00E-05	0.007371	1.310469
ILMN_1700048	0.081616	5.846974	3.522609	0.000922	0.034446	-0.82833
ILMN_1742923	0.081258	6.171055	3.427659	0.001226	0.040519	-1.08736
ILMN_1806310	-0.08126	5.994995	-3.38214	0.001403	0.043982	-1.21004
ILMN_1706959	0.081203	8.660833	3.552337	0.000843	0.032579	-0.74637
ILMN_1726930	-0.08109	7.077231	-3.83218	0.000356	0.018691	0.043915
ILMN_2141157	-0.08103	6.212235	-4.31096	7.61E-05	0.006596	1.46551
ILMN_1793615	-0.08099	5.851298	-3.46684	0.001091	0.037673	-0.98099
ILMN_1715181	0.080834	6.690597	3.483711	0.001037	0.036654	-0.93495
ILMN_1678627	0.080782	5.3752	4.266372	8.81E-05	0.007286	1.329856
ILMN_1692896	0.080746	6.625689	3.568255	0.000803	0.031902	-0.70232
ILMN_1735415	0.080736	7.315729	3.314233	0.001713	0.04894	-1.39117
ILMN_3297317	0.080418	13.90607	3.783083	0.000415	0.020575	-0.09709
ILMN_3245143	-0.08022	5.815494	-3.80461	0.000388	0.01978	-0.03537
ILMN_1776352	-0.08009	7.781603	-3.44382	0.001168	0.039253	-1.04356
ILMN_1774281	-0.08001	6.209842	-3.33091	0.001632	0.047848	-1.34688
ILMN_2197128	0.079973	6.431742	3.330231	0.001635	0.047848	-1.3487
ILMN_2177460	0.079857	6.285313	3.316432	0.001702	0.04894	-1.38534
ILMN_1769503	-0.07962	5.950628	-3.33759	0.0016	0.047389	-1.32912
ILMN_1698725	0.079448	6.157744	3.917272	0.000272	0.015534	0.290564
ILMN_3245893	-0.0793	6.436412	-3.46023	0.001112	0.038043	-0.99897
ILMN_1662334	0.079128	9.877035	4.099661	0.000152	0.010781	0.828299
ILMN_2366714	-0.07908	7.945501	-3.51268	0.00095	0.034911	-0.8556
ILMN_2049642	-0.07889	10.4411	-3.91907	0.00027	0.015534	0.2958
ILMN_1718898	0.078805	6.109315	3.590075	0.000752	0.030491	-0.64176
ILMN_1773716	0.078737	7.94473	3.423365	0.001242	0.040845	-1.09898
ILMN_2134224	0.078722	6.828101	3.848606	0.000338	0.018131	0.091317
ILMN_1782730	0.078428	6.297205	4.27458	8.58E-05	0.007145	1.354782
ILMN_1692486	0.078346	8.511366	3.801548	0.000391	0.019839	-0.04417
ILMN_1722771	-0.07816	5.778356	-3.54389	0.000865	0.033089	-0.76969
ILMN_3268590	0.077823	5.775323	3.516837	0.000938	0.034825	-0.84419
ILMN_1721628	0.077529	5.548135	3.353768	0.001526	0.045896	-1.28599

ILMN_2388965	0.077408	5.253054	4.08695	0.000158	0.011033	0.790439
ILMN_2124425	0.077408	5.376862	3.615942	0.000695	0.028957	-0.56968
ILMN_1676159	-0.07724	9.37641	-3.31593	0.001705	0.04894	-1.38667
ILMN_2390586	-0.07637	7.047253	-3.81925	0.00037	0.019194	0.006685
ILMN_1809094	-0.07597	6.7153	-3.46461	0.001098	0.037789	-0.98707
ILMN_1713454	0.07573	6.15932	3.432302	0.001209	0.040078	-1.07479
ILMN_1803094	0.075213	6.214764	3.5056	0.000971	0.035265	-0.87504
ILMN_1696826	0.074385	5.909971	4.184926	0.000115	0.008798	1.083682
ILMN_1737818	0.074303	7.595981	3.330799	0.001632	0.047848	-1.34719
ILMN_1806782	0.074235	6.27442	3.377609	0.001422	0.044289	-1.22219
ILMN_1705330	-0.0737	6.392187	-3.34973	0.001544	0.046203	-1.29675
ILMN_1761181	0.073293	5.857509	3.562204	0.000818	0.032056	-0.71908
ILMN_1792242	0.073257	5.673458	3.315421	0.001707	0.04894	-1.38802
ILMN_1712751	0.073132	7.449822	3.354747	0.001521	0.045804	-1.28337
ILMN_1801395	-0.07282	5.985178	-3.33039	0.001634	0.047848	-1.34828
ILMN_1699836	0.072332	5.569219	3.441598	0.001176	0.039438	-1.0496
ILMN_1715654	0.072229	6.159278	3.493423	0.001007	0.036049	-0.9084
ILMN_1748607	-0.07213	5.781067	-3.52367	0.000919	0.03441	-0.82539
ILMN_1713247	-0.07186	5.57309	-3.48722	0.001026	0.036495	-0.92537
ILMN_1689651	0.07114	5.329057	3.727953	0.000492	0.023093	-0.25427
ILMN_1799669	-0.07091	6.169469	-3.60346	0.000722	0.029718	-0.60451
ILMN_1676594	-0.07084	5.782597	-3.51472	0.000944	0.034911	-0.85001
ILMN_1789955	-0.07046	5.498019	-3.40629	0.001306	0.042165	-1.14506
ILMN_2410905	-0.07006	5.267522	-3.38672	0.001384	0.043715	-1.19773
ILMN_1696767	0.069905	5.26613	3.404329	0.001314	0.042371	-1.15036
ILMN_1815570	0.069839	6.374613	3.476571	0.001059	0.037034	-0.95445
ILMN_1767441	0.069765	6.13581	3.68844	0.000556	0.024798	-0.36614
ILMN_2209766	-0.06973	6.632917	-3.48457	0.001034	0.036639	-0.9326
ILMN_3300358	0.069564	6.250778	3.482501	0.00104	0.036679	-0.93826
ILMN_1728802	-0.06932	5.97848	-3.48819	0.001023	0.036431	-0.92272
ILMN_1887128	-0.06927	6.128285	-3.8509	0.000335	0.018029	0.097944
ILMN_1759312	-0.06919	5.296898	-3.40259	0.001321	0.042432	-1.15504
ILMN_2085446	0.06817	6.516636	3.3381	0.001598	0.047389	-1.32776
ILMN_1661002	-0.06753	9.235879	-3.52587	0.000913	0.034221	-0.81936
ILMN_1651498	0.066353	5.48707	3.320379	0.001683	0.048567	-1.37487
ILMN_3225014	0.066123	5.747072	3.42179	0.001247	0.040958	-1.10324
ILMN_1885908	-0.06612	5.936269	-3.58262	0.000769	0.031004	-0.66247
ILMN_1761844	0.066018	8.75912	3.485764	0.00103	0.036546	-0.92934
ILMN_1693319	-0.06332	5.331141	-3.46317	0.001103	0.037822	-0.99097
ILMN_2338849	-0.06286	5.81064	-3.59854	0.000733	0.030029	-0.61822
ILMN_1759801	0.062286	7.855688	3.456994	0.001123	0.038324	-1.00778
ILMN_2038773	-0.06124	14.04246	-3.33314	0.001621	0.047632	-1.34095
ILMN_2084912	-0.05984	5.261935	-3.4399	0.001182	0.03959	-1.05419
ILMN_3211262	0.059778	5.588309	3.351735	0.001535	0.046067	-1.29142
ILMN_1673042	0.059436	6.00835	3.618257	0.00069	0.028788	-0.56322
ILMN_2331501	-0.0583	14.14271	-3.45588	0.001127	0.038349	-1.01083
ILMN_1736234	0.055856	8.677606	3.471953	0.001074	0.037399	-0.96705

Supplementary Table S4. Genes showing significant concentration-dependent associations with levels of HPV16 E7 protein in the W12 clones, after exclusion of known p53 targets.

	logFC	AveExpr	t	P.Value	adj.P.Val	B
ILMN_2133205	-0.69081606	7.99216877	-5.434234286	1.62786E-06	0.000660259	5.034164943
ILMN_1739513	0.604786058	7.219707756	8.73629807	1.22083E-11	4.20894E-07	16.21004505
ILMN_1744604	-0.586736389	7.452409232	-6.368581534	5.80282E-08	6.21511E-05	8.197194939
ILMN_2391861	-0.58146284	8.151042478	-4.662890606	2.34913E-05	0.002999582	2.511430461
ILMN_1725139	0.565743007	6.326501386	7.51379229	9.38235E-10	6.46932E-06	12.10954146
ILMN_1773006	-0.55724265	10.33058279	-3.284381713	0.001869435	0.044571665	-1.564808264
ILMN_1705750	-0.441252203	9.719860872	-5.369658152	2.0432E-06	0.000741489	4.818896084
ILMN_1668134	-0.427845205	6.914500691	-3.333975919	0.001617036	0.040662965	-1.431979315
ILMN_1788874	0.411575486	7.706951017	4.32059777	7.37427E-05	0.006355887	1.4361046
ILMN_1705080	-0.40105173	8.187943267	-4.27216586	8.64784E-05	0.006982696	1.286748685
ILMN_1772627	-0.38929497	8.074500632	-4.808453546	1.43184E-05	0.002349659	2.97812617
ILMN_1762255	-0.387429166	7.106343715	-3.230938793	0.002182939	0.04951251	-1.706528162
ILMN_1812403	-0.371523466	9.949196794	-4.952175936	8.74355E-06	0.001684037	3.443709792
ILMN_1682015	0.370871001	8.023019495	3.887879387	0.000298325	0.015373789	0.130088999
ILMN_1673639	-0.367455572	7.225538082	-4.707037288	2.02263E-05	0.002834636	2.652426128
ILMN_1671478	-0.365558346	8.875750533	-5.909231965	3.01483E-07	0.000206424	6.633195965
ILMN_2072178	0.336319958	7.401153344	4.91526345	9.92829E-06	0.001858065	3.323705116
ILMN_1775814	-0.330473923	8.011236094	-3.611781457	0.000703495	0.024698252	-0.665710356
ILMN_3249032	0.32352468	7.755068973	4.622502857	2.69274E-05	0.00328038	2.382872901
ILMN_1699989	-0.322204513	8.280025947	-4.836820403	1.29946E-05	0.002206412	3.069657724
ILMN_1754241	0.320600998	7.183668741	3.920536532	0.000269039	0.014425195	0.22627846
ILMN_1705107	-0.309738213	8.067200163	-3.823755691	0.000365025	0.017052307	-0.057557178
ILMN_1790227	-0.309427044	8.273253594	-3.76294215	0.000441397	0.018973996	-0.233977083
ILMN_1693338	0.303946945	8.093768737	3.309335964	0.001738105	0.042498505	-1.498129471
ILMN_1744765	-0.301370098	11.11599086	-3.415095266	0.001272501	0.034790444	-1.212054595
ILMN_2242463	0.295329948	10.18413434	5.304838316	2.56523E-06	0.000820235	4.603434503
ILMN_2096372	-0.294100034	6.843430779	-3.445070773	0.001163826	0.033105678	-1.129969727
ILMN_1690017	-0.290045091	8.585351717	-3.297978687	0.001796754	0.043525466	-1.528516101
ILMN_1805665	-0.282212258	9.899023581	-4.772988676	1.6161E-05	0.002454475	2.86395239
ILMN_1737561	-0.280160022	7.754603778	-4.796294912	1.49256E-05	0.002349659	2.938950315
ILMN_2138765	0.27958152	7.772093849	6.15026449	1.27172E-07	0.000101962	7.452398073
ILMN_2390919	-0.269610984	7.823265094	-4.84969472	1.24341E-05	0.002198351	3.111259521
ILMN_2304512	0.263785142	8.426434592	3.512193909	0.000951626	0.029398089	-0.944599546
ILMN_2376723	-0.257203244	9.075358902	-5.290871944	2.69391E-06	0.000839969	4.557095056
ILMN_1651496	-0.255719192	9.321274314	-3.474688831	0.001065162	0.03116883	-1.048438888
ILMN_1696347	0.254268017	9.758518681	4.311321696	7.60315E-05	0.006440445	1.407441359
ILMN_1706015	-0.251978895	7.10022439	-3.335179203	0.001611333	0.040600074	-1.428741032
ILMN_1711087	-0.245690545	8.940669647	-5.58841911	9.44144E-07	0.000438736	5.550430731
ILMN_1774602	-0.245031785	8.368134171	-4.834020371	1.31197E-05	0.002206412	3.060614701
ILMN_1808590	0.244657643	7.18808754	3.810764404	0.000380188	0.017435104	-0.095372325
ILMN_1660067	0.239113589	7.137711934	4.834179622	1.31125E-05	0.002206412	3.061128974
ILMN_1667966	-0.237567824	7.760318553	-5.395071302	1.86853E-06	0.000715772	4.903541006
ILMN_1760990	-0.237268838	6.710186996	-5.289764156	2.70439E-06	0.000839969	4.553420812
ILMN_1798006	-0.234468968	7.928235922	-3.734576189	0.000482068	0.019832651	-0.315741353
ILMN_3244348	0.232542783	8.046508909	6.477717038	3.91743E-08	5.35713E-05	8.570171447
ILMN_1751086	0.230310703	9.493877108	5.759145075	5.14908E-07	0.000306068	6.125371429
ILMN_3227023	-0.23001724	9.465506347	-5.6941025	6.48928E-07	0.000366761	5.905962054
ILMN_3234089	-0.228138307	7.081691467	-4.222943136	0.000101606	0.007390097	1.135726128
ILMN_1743103	-0.225103715	10.09195226	-4.85508309	1.22067E-05	0.002169262	3.12868241
ILMN_3242586	-0.225102888	6.789435012	-3.673373877	0.00058243	0.022310943	-0.490990257

ILMN_1800130	-0.224479568	7.260116812	-4.682044429	2.20158E-05	0.002875057	2.572544029
ILMN_1799098	-0.224440989	10.47357299	-6.664318918	1.99966E-08	3.44702E-05	9.208464827
ILMN_1800317	-0.22350407	6.100467508	-4.73141825	1.86183E-05	0.002685707	2.730500877
ILMN_3305614	-0.223368093	6.399203071	-3.957931005	0.000238909	0.013337338	0.336927568
ILMN_1695945	-0.221304142	6.886069381	-6.481233056	3.86814E-08	5.35713E-05	8.582192824
ILMN_1790100	0.219813055	8.226172551	5.730934118	5.69281E-07	0.000327109	6.030153859
ILMN_2160209	0.219490692	6.731097227	4.859041812	1.20422E-05	0.002151118	3.141486766
ILMN_1699651	0.218890296	6.608252975	3.239463627	0.002129806	0.048562949	-1.684021809
ILMN_1700583	0.218227029	9.328274748	3.818622526	0.000370945	0.017189122	-0.072507039
ILMN_1721876	-0.216364977	8.700419317	-5.122888993	4.84206E-06	0.001192391	4.002271707
ILMN_1777233	0.215540938	8.912187296	6.414196826	4.92422E-08	5.65891E-05	8.353046659
ILMN_1856480	-0.214646444	7.171697048	-3.953969429	0.00024194	0.013410175	0.325180073
ILMN_1749096	0.212726388	6.532684661	3.974450438	0.000226663	0.01305066	0.385977694
ILMN_1708934	-0.21218095	11.66936382	-4.556297003	3.3652E-05	0.003769727	2.173058244
ILMN_1693471	0.211880179	8.40287487	3.659052725	0.000608656	0.022886172	-0.531764293
ILMN_1781285	-0.207384102	10.9608232	-3.970814137	0.000229305	0.013127837	0.375171805
ILMN_1711069	-0.207365644	9.757086349	-4.365354615	6.36097E-05	0.005842125	1.574779422
ILMN_2095610	-0.205748074	9.971736434	-6.183609185	1.12826E-07	9.26141E-05	7.566011811
ILMN_1810725	-0.20454778	7.339040559	-4.126050766	0.000139254	0.009322172	0.840803468
ILMN_2186983	-0.203337649	10.29542032	-5.879732506	3.3498E-07	0.000217901	6.533227358
ILMN_1678655	-0.202616789	9.884597969	-3.297783238	0.00179778	0.043525466	-1.529038451
ILMN_3224926	0.202020378	8.871704037	4.835870289	1.30369E-05	0.002206412	3.066589024
ILMN_3242004	-0.20152848	9.102102216	-5.679996016	6.82279E-07	0.000372997	5.858434477
ILMN_3236858	-0.201080371	6.435354039	-3.828722864	0.000359383	0.016949503	-0.04308058
ILMN_1739450	-0.200962318	9.157352887	-6.319265215	6.92932E-08	6.63598E-05	8.028776021
ILMN_1772910	-0.199887607	8.398031431	-4.433315319	5.07675E-05	0.004902692	1.786510553
ILMN_3306730	0.198533654	8.639005981	4.871313943	1.1546E-05	0.002095043	3.181202652
ILMN_1675616	0.197984324	6.875555063	4.674844139	2.25595E-05	0.002934946	2.549559417
ILMN_1657766	-0.195994708	10.09657349	-4.696233253	2.09816E-05	0.002858752	2.617875261
ILMN_1793017	-0.195238893	7.235809229	-6.339357904	6.44619E-08	6.34968E-05	8.097383583
ILMN_1696270	-0.195203211	8.109972371	-6.4444893	4.41541E-08	5.48717E-05	8.456579329
ILMN_2413816	0.194739056	6.652781464	4.318761034	7.41905E-05	0.006378536	1.430426911
ILMN_1749109	-0.192449327	11.15245326	-6.469158372	4.04007E-08	5.35713E-05	8.54091029
ILMN_1791580	-0.191163585	7.516766911	-4.139493976	0.000133319	0.009067373	0.8815308
ILMN_1792679	0.190884687	7.392166643	5.616247963	8.55478E-07	0.000409631	5.643927145
ILMN_1672908	-0.190029403	6.931672228	-3.88584416	0.000300249	0.015384685	0.124108179
ILMN_1788192	-0.189931105	6.735081722	-4.171442236	0.000120181	0.008435971	0.97857037
ILMN_1729217	-0.189654983	6.337149941	-3.90234596	0.000284991	0.014932161	0.172648018
ILMN_1731640	-0.188879701	8.258373683	-3.583765928	0.000766212	0.025766729	-0.744623495
ILMN_1686555	-0.188776926	6.82099883	-5.603471483	8.95111E-07	0.000422737	5.600990798
ILMN_1799106	0.188677261	7.097389027	5.035170511	6.56441E-06	0.001468809	3.714552519
ILMN_1685540	0.188063075	7.832534157	6.99285609	6.11633E-09	1.81181E-05	10.33249881
ILMN_1775114	-0.18800111	7.008211087	-4.759288223	1.69334E-05	0.002505568	2.819924898
ILMN_2355559	-0.187680333	11.20146333	-5.519534969	1.20472E-06	0.000512766	5.31940396
ILMN_1678669	0.186617129	7.898199559	4.439089744	4.98012E-05	0.004863867	1.804563809
ILMN_2365686	0.185660958	9.254504264	4.583386116	3.07222E-05	0.003614948	2.258765943
ILMN_1741755	-0.184704068	9.953249213	-5.348926663	2.19758E-06	0.000773098	4.749915185
ILMN_1709307	-0.184410067	7.171161366	-3.706517537	0.000525825	0.020981412	-0.396284954
ILMN_2180371	0.183056046	7.825553085	4.364051922	6.38845E-05	0.005842125	1.570734456
ILMN_1778360	-0.181926162	10.85475199	-4.375464462	6.15161E-05	0.005670668	1.606188858
ILMN_1676361	-0.181459616	7.368993026	-5.379348197	1.97477E-06	0.00072428	4.851160093
ILMN_3248857	0.180973672	6.346805873	6.936004619	7.50766E-09	1.84881E-05	10.13806579
ILMN_1692938	0.180920967	8.625168167	5.423327133	1.69162E-06	0.000678144	4.997763526
ILMN_1669433	-0.180796705	8.76455444	-5.322499946	2.41117E-06	0.000807064	4.662078245

ILMN_1787265	-0.179947208	8.776492154	-4.124454541	0.000139975	0.009352316	0.835971736
ILMN_1886515	-0.179759802	8.070173077	-4.52876612	3.69091E-05	0.003964103	2.086157012
ILMN_2342437	0.179461728	7.164943946	5.106722261	5.12191E-06	0.001243056	3.94913595
ILMN_2330307	0.179245561	7.789457065	4.218576517	0.000103066	0.00745919	1.122367006
ILMN_2125346	-0.178228373	6.785924135	-3.740125974	0.000473837	0.019681937	-0.299770861
ILMN_3251691	0.177896819	7.470628025	4.456042621	4.70667E-05	0.004730816	1.857621624
ILMN_1711994	0.17738201	7.334313561	5.234759307	3.27838E-06	0.000941879	4.371232742
ILMN_2390974	-0.176315262	9.158101172	-5.108661255	5.08752E-06	0.001243056	3.95550637
ILMN_1704537	0.174733415	9.658633574	3.558422992	0.000827522	0.026892586	-0.815702295
ILMN_2376050	-0.174418352	8.043913138	-3.957664218	0.000239112	0.013337338	0.336136263
ILMN_1692026	0.174249718	7.547477453	7.133646458	3.68248E-09	1.58696E-05	10.81370447
ILMN_1749118	0.173849678	6.735765352	3.493217695	0.001007547	0.030416973	-0.997221387
ILMN_1724139	0.172771062	11.04195193	5.130281889	4.71916E-06	0.001178969	4.02658611
ILMN_2326712	0.172670154	7.108977109	4.557986414	3.34616E-05	0.003769727	2.17839761
ILMN_1809695	-0.172032373	8.243369376	-4.09577975	0.000153572	0.010008618	0.749325765
ILMN_1698732	0.171963635	9.84195521	4.398668037	5.69618E-05	0.005349064	1.678394828
ILMN_1664861	-0.171785172	10.51661568	-4.256030437	9.11785E-05	0.007161366	1.237156045
ILMN_3201453	0.171559671	9.408963236	4.976451759	8.0414E-06	0.001575201	3.522786754
ILMN_2131177	0.170944003	6.861965588	3.57975505	0.000775617	0.025860693	-0.755892192
ILMN_1811195	-0.170777634	6.387586903	-6.000661858	2.17402E-07	0.000162938	6.943470744
ILMN_1732609	-0.170355087	7.328232052	-4.307224194	7.70644E-05	0.006480173	1.394788601
ILMN_1903021	-0.170261062	6.660382587	-4.928029357	9.50171E-06	0.001809839	3.365175324
ILMN_1754842	-0.170090714	7.573755813	-5.858034943	3.61955E-07	0.000231088	6.459744675
ILMN_1723412	-0.169892927	8.099925286	-4.098267393	0.000152344	0.00996623	0.756831168
ILMN_1737283	-0.169839612	8.270081685	-3.433751064	0.001203785	0.033496112	-1.161018718
ILMN_1797822	-0.169819001	7.703848732	-3.598168462	0.00073333	0.025231822	-0.704099099
ILMN_2087941	-0.169218725	7.976438825	-4.338841335	6.94355E-05	0.006169738	1.492555873
ILMN_1838942	0.168967176	7.333424158	3.62623211	0.000673096	0.024223018	-0.624868682
ILMN_2151281	-0.168798031	7.544568822	-3.640881204	0.000643565	0.023684694	-0.583371392
ILMN_1673522	0.168705945	8.374232556	6.962167447	6.83186E-09	1.81181E-05	10.22755008
ILMN_1685413	0.168321935	9.058596142	5.340975739	2.25979E-06	0.000786956	4.723476787
ILMN_1700310	-0.16805555	6.956644075	-3.32054578	0.001681997	0.041479641	-1.468072959
ILMN_1800091	0.167428076	6.191857456	6.115846563	1.43886E-07	0.000112741	7.335191582
ILMN_1789775	0.167217551	8.644514677	4.804303276	1.45229E-05	0.002349659	2.964749906
ILMN_1801077	0.167032943	6.694602711	6.731630223	1.56876E-08	3.0047E-05	9.438796451
ILMN_1673543	0.167020085	8.339836748	6.300341597	7.4173E-08	6.91132E-05	7.964174301
ILMN_3305273	0.166487504	7.434980595	6.485870956	3.80406E-08	5.35713E-05	8.598050381
ILMN_2404065	-0.165148456	9.856242498	-6.211028258	1.02246E-07	8.81259E-05	7.659478477
ILMN_2219466	0.164879559	6.618041515	4.044640504	0.000181063	0.011207062	0.595523839
ILMN_1706051	-0.164812187	6.646428759	-3.407280997	0.001302379	0.035350425	-1.23338142
ILMN_1673673	0.16441195	7.43933552	4.492771099	4.16356E-05	0.004323582	1.972855084
ILMN_2217935	0.164017365	8.484240684	6.213782351	1.0124E-07	8.81259E-05	7.668868738
ILMN_1738955	-0.163501544	8.396592065	-5.784860585	4.69851E-07	0.000284185	6.212234602
ILMN_1682402	0.163494202	6.554807944	7.152685197	3.43839E-09	1.58696E-05	10.87873602
ILMN_1679093	-0.163369146	9.14661576	-4.280939092	8.40226E-05	0.006880673	1.313748584
ILMN_3200421	0.163289431	7.3358073	5.135903179	4.62778E-06	0.001173141	4.045080537
ILMN_1781400	-0.163199598	6.351035103	-3.341210803	0.001583034	0.040177859	-1.412497766
ILMN_2209578	-0.162928791	8.965182798	-6.483652944	3.83457E-08	5.35713E-05	8.590466661
ILMN_3238707	0.162412664	7.420619876	5.662761382	7.2534E-07	0.000373236	5.800396698
ILMN_2128967	-0.161538612	9.231967674	-3.882916224	0.000303037	0.015432062	0.115506859
ILMN_1769451	0.161289634	8.506642461	6.994714177	6.0755E-09	1.81181E-05	10.33885251
ILMN_2401878	0.161225706	8.209210494	4.839888051	1.28588E-05	0.002206412	3.079567074
ILMN_3242883	-0.161205999	8.583161327	-5.667177408	7.14057E-07	0.000372997	5.815264617
ILMN_1781386	-0.161096662	7.357152482	-3.757414509	0.000449054	0.019184124	-0.249936876

ILMN_1683450	0.160862649	9.802312178	5.193306531	3.78897E-06	0.001045028	4.234263393
ILMN_2134039	0.159433681	9.220499199	3.58571311	0.000761685	0.025703058	-0.739150192
ILMN_1658426	-0.158942288	6.566176358	-3.84896685	0.000337245	0.016315041	0.016022569
ILMN_1756541	-0.15859699	8.567317396	-5.037758648	6.50585E-06	0.001468809	3.723020664
ILMN_1772036	0.158129245	6.536795991	4.559152045	3.33308E-05	0.003769727	2.182082023
ILMN_1748352	-0.157393569	11.4101258	-3.797205911	0.000396658	0.017952992	-0.134765049
ILMN_1775405	-0.15729259	9.58175858	-4.543559767	3.51224E-05	0.003881022	2.132827361
ILMN_1778543	0.1572839	8.784347679	4.260866042	8.97444E-05	0.007096396	1.252009579
ILMN_2214678	-0.157210629	7.753443547	-3.245479044	0.00209305	0.048074616	-1.668117636
ILMN_2072622	0.157172039	9.120219576	4.198378494	0.000110089	0.007858011	1.060656016
ILMN_2357134	0.156517706	7.806796626	3.527336874	0.000909139	0.028592897	-0.90248659
ILMN_2401873	0.156332014	8.387227745	4.104447232	0.000149333	0.009825188	0.775485649
ILMN_1671404	0.156070665	9.585143281	3.870028606	0.000315609	0.015838348	0.077687462
ILMN_1715684	-0.155924291	9.932006939	-3.560712266	0.000821797	0.026753799	-0.809293661
ILMN_1655622	0.155651196	9.877113686	4.797424893	1.48681E-05	0.002349659	2.942589739
ILMN_1712888	-0.15557024	10.95199369	-3.353099236	0.001528629	0.03929978	-1.380428388
ILMN_1802205	-0.15556641	9.479562597	-3.819331257	0.000370122	0.017174075	-0.070443562
ILMN_3235404	0.155366503	7.050826139	5.3826492	1.95198E-06	0.00072428	4.862154273
ILMN_2121816	-0.15490423	6.828962555	-5.928019578	2.81907E-07	0.000201694	6.696900848
ILMN_1663866	-0.154716466	12.1605902	-3.517276977	0.000937157	0.02913385	-0.930475332
ILMN_1716026	-0.154543997	7.359713491	-5.030641181	6.66812E-06	0.001468809	3.699736121
ILMN_1678922	-0.154325442	8.855589712	-4.427099176	5.18282E-05	0.004991147	1.76708721
ILMN_2413898	0.153063577	8.123873246	4.091501176	0.000155708	0.010071639	0.736422113
ILMN_1669703	-0.153029137	8.590866258	-5.211281143	3.55859E-06	0.000997446	4.293619832
ILMN_1682038	0.152626201	8.37477388	5.274187959	2.85601E-06	0.000871363	4.501779322
ILMN_1685580	-0.152486775	7.695230185	-5.923337273	2.86664E-07	0.000201694	6.681021494
ILMN_1665775	0.152172029	6.339835111	3.71560422	0.000511253	0.020687748	-0.37023796
ILMN_2413899	0.151897125	6.613339801	4.608783474	2.82029E-05	0.003399736	2.339299082
ILMN_1778087	-0.151745302	12.66377147	-4.56805032	3.23487E-05	0.003717517	2.210220487
ILMN_1708672	0.151090049	9.817585384	3.752234828	0.000456345	0.019327963	-0.264880414
ILMN_1696713	0.150861164	7.818523367	5.154864322	4.33222E-06	0.001140133	4.107505891
ILMN_1712305	-0.150682223	9.841135588	-3.330954656	0.001631439	0.040776416	-1.440106937
ILMN_1729225	-0.150497387	6.254295319	-4.302849011	7.81823E-05	0.006509608	1.381284225
ILMN_2229379	-0.150492034	6.133629685	-3.401294511	0.001325717	0.03556843	-1.249699635
ILMN_3247848	-0.150138198	6.894470153	-4.595576993	2.94866E-05	0.003505451	2.297400988
ILMN_1694840	-0.150028314	7.088939309	-3.656411111	0.000613616	0.022994577	-0.539275488
ILMN_3250268	0.149967121	7.791705366	4.080780897	0.000161186	0.010290814	0.704119589
ILMN_1763907	0.149786741	10.09856606	5.881885369	3.32415E-07	0.000217901	6.540520595
ILMN_1774685	0.149768742	6.321316435	4.344314134	6.81919E-05	0.006106451	1.509510537
ILMN_1810942	-0.149702983	6.842908302	-3.492406357	0.001010006	0.030437914	-0.999467488
ILMN_1682165	-0.149563248	9.812250366	-4.017136362	0.000197761	0.012003526	0.513195915
ILMN_1805828	0.148962681	8.975598563	4.704548716	2.03978E-05	0.00284711	2.644465216
ILMN_2217809	0.147680466	9.865297437	3.739592192	0.000474623	0.019688161	-0.301307477
ILMN_1760620	0.147481626	7.638821681	5.459608585	1.48857E-06	0.000610953	5.1189126
ILMN_2380588	0.147387481	8.905956084	5.521962125	1.19443E-06	0.000512766	5.327534192
ILMN_1668629	-0.147154939	9.240650478	-3.584475911	0.000764559	0.02576088	-0.742628014
ILMN_1809866	0.14687859	8.35465094	4.989749339	7.68063E-06	0.00153062	3.566153912
ILMN_1664369	0.146223738	6.806387544	5.002120449	7.35931E-06	0.001514546	3.606531683
ILMN_1800412	-0.145983881	7.865449707	-7.84264695	2.88908E-10	2.4901E-06	13.22438735
ILMN_1682738	-0.145622455	8.942543553	-4.35279491	6.63075E-05	0.00598434	1.535802108
ILMN_1669479	0.145338647	7.352697752	3.886306668	0.000299811	0.015384685	0.125467185
ILMN_1759419	0.14526734	8.841508239	6.045601742	1.85086E-07	0.000141801	7.096199979
ILMN_1794914	-0.145136958	7.94905921	-4.301905309	7.84255E-05	0.006509608	1.378372198
ILMN_1711314	0.145106064	9.227915869	4.43504036	5.04769E-05	0.004896446	1.791902728

ILMN_1726839	0.144182595	10.39428272	4.063220354	0.000170565	0.010750255	0.651294455
ILMN_1725193	-0.143995836	10.16470107	-6.361666166	5.94903E-08	6.21511E-05	8.173573313
ILMN_1782403	0.14390058	7.305880123	4.576613226	3.14305E-05	0.003670097	2.237318585
ILMN_1685722	-0.143783374	11.69207743	-5.023255569	6.84072E-06	0.001482674	3.675584849
ILMN_1693630	-0.143757426	6.773813424	-3.635164416	0.000654939	0.023868572	-0.599576963
ILMN_1810392	0.143717491	7.699964909	4.916121205	9.89905E-06	0.001858065	3.326490455
ILMN_1681249	-0.14360394	8.864681078	-5.669190274	7.08971E-07	0.000372997	5.82204226
ILMN_1744211	-0.143491817	6.010109406	-3.922640544	0.000267251	0.01437401	0.232489902
ILMN_1683127	0.143340695	9.525091049	3.33282504	0.001622508	0.040673606	-1.435075885
ILMN_1733519	0.143144613	7.128569209	5.319529648	2.43643E-06	0.000807676	4.652212275
ILMN_1796417	0.143135703	8.30282101	4.688060649	2.15713E-05	0.00287032	2.591758846
ILMN_1656482	-0.142672658	8.367445998	-4.647405892	2.47547E-05	0.003081022	2.462091812
ILMN_1719938	0.142397173	6.741638783	4.097019176	0.000152959	0.009987528	0.753064934
ILMN_2371055	-0.142360181	10.52178493	-4.913918351	9.97432E-06	0.001858065	3.319337564
ILMN_2166524	0.142350673	7.416897998	4.842202109	1.27573E-05	0.002206412	3.087043526
ILMN_1765109	-0.142287738	8.299027525	-3.850239636	0.000335898	0.016287503	0.019744024
ILMN_2224657	0.141857498	6.722117736	3.499253293	0.000989429	0.030107298	-0.980502767
ILMN_2124352	0.141683905	10.239231	4.270791896	8.68692E-05	0.006982696	1.282522502
ILMN_2287653	-0.140669072	6.690451728	-4.690413601	2.13999E-05	0.00287032	2.599276247
ILMN_1784847	0.140526138	7.592238302	5.15827421	4.28107E-06	0.001135341	4.118738996
ILMN_1773906	-0.140375608	11.75632911	-4.760215023	1.68801E-05	0.002505568	2.822901838
ILMN_2203588	-0.140272836	6.674290419	-4.698249637	2.08386E-05	0.002858752	2.62432138
ILMN_1651346	-0.139968425	6.717753434	-4.526180709	3.72303E-05	0.003986188	2.078006861
ILMN_1678671	-0.139868972	7.812515614	-3.603262141	0.000722027	0.025017701	-0.68974463
ILMN_2134974	0.139836124	10.82826205	3.239887182	0.002127198	0.048535586	-1.68290259
ILMN_2348050	0.139666228	9.946905243	3.969140194	0.000230532	0.013127837	0.370199071
ILMN_2062701	-0.139215712	7.849424132	-3.539385865	0.000876631	0.027932299	-0.868901913
ILMN_1775522	-0.138880116	9.771732377	-5.249581104	3.11282E-06	0.000933198	4.420277207
ILMN_3306482	0.138854965	10.27698718	3.644425863	0.000636608	0.023549047	-0.573315977
ILMN_3233229	-0.138791968	7.717481802	-4.754656389	1.72027E-05	0.002529095	2.805050219
ILMN_1803194	0.138673993	8.461655941	5.353379316	2.16348E-06	0.000768949	4.764725301
ILMN_1779677	-0.138671702	7.56289937	-3.440629931	0.00117935	0.03321835	-1.142157945
ILMN_2404063	-0.138518499	9.331986562	-6.683808647	1.86398E-08	3.38223E-05	9.275154541
ILMN_1732967	-0.13842616	8.497603366	-5.035315602	6.56111E-06	0.001468809	3.71502721
ILMN_1770206	0.138378143	9.242453848	6.593716941	2.5792E-08	4.23431E-05	8.966905328
ILMN_1731349	-0.138277816	6.896796949	-3.364114179	0.001479808	0.038388161	-1.350652164
ILMN_2414533	-0.138187778	7.051802978	-5.199745711	3.70479E-06	0.001030052	4.255520742
ILMN_1719344	-0.138021685	8.761622237	-4.332063248	7.10064E-05	0.006248444	1.471570285
ILMN_2409220	0.137835765	9.171432101	3.376778425	0.001425506	0.03743013	-1.316342808
ILMN_2297626	0.137795828	7.303478771	3.2946442	0.001814327	0.043772377	-1.537425071
ILMN_1803018	-0.137765616	8.220185437	-4.377544234	6.10938E-05	0.00564684	1.612654173
ILMN_1744835	0.137732232	11.05111462	4.597183659	2.93275E-05	0.003498592	2.302495741
ILMN_2103397	-0.137635955	6.499243191	-5.00803853	7.21032E-06	0.001514546	3.625858424
ILMN_1723971	0.137598534	8.30686531	3.794033904	0.000400609	0.01801027	-0.14397009
ILMN_2079285	-0.137553426	9.107559641	-3.570242018	0.000798368	0.02631408	-0.782590146
ILMN_3237385	-0.137317691	8.080546148	-5.104814744	5.15596E-06	0.001243056	3.942869619
ILMN_1730824	0.137295269	7.513409392	4.136143547	0.000134774	0.009128652	0.871374536
ILMN_1742577	0.137250494	10.20435451	4.865067923	1.17959E-05	0.002118109	3.160984704
ILMN_2334760	-0.137084795	6.781816131	-3.496135651	0.000998749	0.030259518	-0.989140785
ILMN_1711005	0.136865906	7.497245812	3.885578433	0.000300501	0.015384685	0.123327421
ILMN_1797236	-0.136772365	6.569355035	-3.821004784	0.000368186	0.0171535	-0.065570266
ILMN_1667319	-0.136599128	6.22959011	-4.537408151	3.58548E-05	0.003911806	2.113413204
ILMN_1903914	0.136454991	7.610041268	3.330797365	0.001632192	0.040776416	-1.440529948
ILMN_2074044	0.136380516	7.061313224	4.131126604	0.000136983	0.009223903	0.85617378

ILMN_1739805	-0.136336689	8.701650521	-4.807548135	1.43628E-05	0.002349659	2.975207704
ILMN_1796013	0.135658981	7.230506607	5.029746634	6.6888E-06	0.001468809	3.696810342
ILMN_1716869	-0.135482093	6.670322582	-4.560758567	3.31513E-05	0.003769727	2.187160644
ILMN_1770641	-0.135281249	6.005745844	-4.934166822	9.30309E-06	0.001781851	3.385125245
ILMN_3246206	0.13510085	7.060913533	3.334751648	0.001613357	0.040600074	-1.429891753
ILMN_2082324	0.135069489	7.349484567	6.780015062	1.31761E-08	2.84323E-05	9.604367932
ILMN_2045994	-0.134645968	8.174051829	-5.28472887	2.75251E-06	0.000847283	4.536722534
ILMN_1766658	0.134501684	8.098890705	4.480061918	4.34418E-05	0.004445842	1.932937237
ILMN_2225718	0.134490032	7.499276731	3.923444925	0.00026657	0.01437401	0.23486504
ILMN_2061565	-0.134398759	8.345870025	-4.452979364	4.75497E-05	0.004751659	1.848028311
ILMN_2140700	-0.134239212	6.757812663	-5.322579708	2.4105E-06	0.000807064	4.662343199
ILMN_1726104	0.134232299	8.323975493	4.414013642	5.41323E-05	0.005169713	1.726236489
ILMN_1718972	0.134151401	8.220587667	4.912736061	1.0015E-05	0.001858065	3.315498974
ILMN_1698189	0.134120129	8.283863773	5.123158075	4.83753E-06	0.001192391	4.003156513
ILMN_2344850	-0.133863845	10.71218979	-6.343874506	6.34229E-08	6.34968E-05	8.112807788
ILMN_1660341	-0.133861417	8.027968608	-4.689995466	2.14303E-05	0.00287032	2.597940255
ILMN_1666376	-0.133446973	7.528632543	-6.24549166	9.03388E-08	8.19611E-05	7.777009607
ILMN_1795336	0.133249892	5.682204554	3.744820931	0.00046698	0.019597123	-0.286250138
ILMN_1724493	0.132792171	8.164774122	3.661775981	0.000603583	0.022767109	-0.524017755
ILMN_2135984	0.132714325	6.588567296	3.86503149	0.000320618	0.015996571	0.063040856
ILMN_1666599	0.132236703	7.265357528	4.581722066	3.08948E-05	0.003622886	2.253495347
ILMN_1832208	0.132046084	9.540906978	4.738400622	1.81813E-05	0.002633698	2.75288707
ILMN_1806809	-0.132009218	6.996510918	-5.149083599	4.42029E-06	0.00114582	4.088467328
ILMN_3274596	-0.131988436	10.58902558	-4.51368444	3.88219E-05	0.004079352	2.038640221
ILMN_2326509	0.131875834	8.811934897	4.058724461	0.00017305	0.010808082	0.637787827
ILMN_1671791	0.131707646	7.46911622	3.95720059	0.000239465	0.013337338	0.334761178
ILMN_1712400	0.131686898	8.876374823	3.973425412	0.000227404	0.01305066	0.382931149
ILMN_1655876	-0.131192124	7.946875505	-3.879162623	0.000306648	0.015569955	0.104484945
ILMN_1773567	-0.131106867	11.08087231	-3.300752785	0.001782258	0.043301717	-1.521100023
ILMN_3263702	0.131029339	6.359063635	4.364794148	6.37278E-05	0.005842125	1.573039062
ILMN_1709483	0.130637789	7.580602966	5.078288626	5.6533E-06	0.001334954	3.855799638
ILMN_1669323	0.130573864	7.255135811	3.906709518	0.000281084	0.014794866	0.18550117
ILMN_1788166	0.130487452	8.67503595	4.409127715	5.5018E-05	0.005225347	1.710996463
ILMN_1797332	0.130392724	8.237209661	3.75292074	0.000455373	0.019310517	-0.262902184
ILMN_2414399	0.130313078	7.245649804	5.019535229	6.92932E-06	0.001482674	3.66342324
ILMN_1745034	0.130044991	8.710431443	4.412001127	5.44954E-05	0.005190014	1.719958259
ILMN_1655557	-0.129260021	8.609699708	-3.711134996	0.000518371	0.020853402	-0.383053414
ILMN_1669831	0.129227966	7.643488292	6.779613252	1.31952E-08	2.84323E-05	9.602992966
ILMN_1678678	-0.128890436	8.497363089	-3.321194383	0.001678804	0.041479641	-1.46633193
ILMN_1759023	-0.128823437	6.831852282	-3.739248696	0.000475129	0.019688161	-0.302296248
ILMN_2285375	0.128498482	8.553090667	3.588040675	0.000756308	0.025663857	-0.732605426
ILMN_2151056	-0.128474727	7.826808254	-4.754034688	1.72392E-05	0.002529095	2.803054078
ILMN_2148150	0.12804483	6.811128089	3.578094744	0.000779543	0.025916599	-0.760554739
ILMN_1665372	-0.127976771	6.998520963	-4.19294782	0.000112055	0.007948977	1.044086908
ILMN_1748476	0.127973869	10.84240498	5.149220128	4.41819E-06	0.00114582	4.088916909
ILMN_1657697	-0.12791406	9.85875281	-4.834977222	1.30768E-05	0.002206412	3.063704763
ILMN_1717674	-0.127613098	9.362654833	-3.968068231	0.00023132	0.013138388	0.36701518
ILMN_1796589	0.127567824	9.182447848	4.265462566	8.84016E-05	0.007038648	1.266135738
ILMN_1735052	-0.127511934	8.102006174	-3.661395562	0.000604289	0.022768834	-0.525100084
ILMN_1667893	-0.12724145	8.518278757	-3.321693723	0.001676349	0.041479641	-1.464991416
ILMN_2398107	0.126452904	7.265734244	5.561625004	1.0381E-06	0.000470915	5.460497944
ILMN_1777564	0.126416461	10.34517603	4.686447172	2.16896E-05	0.00287032	2.586604779
ILMN_1670353	0.126202046	9.008402824	3.895257577	0.000291451	0.015162742	0.151784555
ILMN_1799128	0.126115937	9.232999206	5.018451454	6.95534E-06	0.001482674	3.659880949

ILMN_1758915	0.126029809	8.708471252	4.80157781	1.46587E-05	0.002349659	2.955967933
ILMN_1713764	-0.125727973	6.910494514	-4.313105262	7.55861E-05	0.006438484	1.412950516
ILMN_2305112	0.125701018	6.280870741	6.979481827	6.41845E-09	1.81181E-05	10.28676377
ILMN_1815723	0.125541374	8.405450161	4.804234253	1.45263E-05	0.002349659	2.964527482
ILMN_1704793	-0.12527972	8.555960612	-4.29389654	8.05193E-05	0.00664111	1.353670504
ILMN_1703324	0.125235208	7.840373041	3.850881741	0.00033522	0.016287503	0.021621695
ILMN_2085525	0.125177817	7.593519229	4.701681307	2.05973E-05	0.002851861	2.635294307
ILMN_1776337	0.125156293	8.17941259	3.534364951	0.00089004	0.028141074	-0.8829051
ILMN_1811472	0.124958552	8.848214771	3.522106286	0.000923606	0.028842595	-0.917045062
ILMN_1700507	-0.124920839	9.003335212	-3.227332625	0.00220579	0.049866774	-1.716037293
ILMN_1789510	0.124792528	9.487128892	3.317420975	0.001697463	0.041801229	-1.476457813
ILMN_1669981	-0.124360908	6.921258888	-4.791456723	1.51742E-05	0.002377933	2.923370904
ILMN_1725366	0.12429741	6.5204632	3.678449817	0.000573397	0.022062985	-0.476516991
ILMN_1801939	0.123929796	9.923410899	3.578857424	0.000777737	0.025881529	-0.758413101
ILMN_3300198	-0.123622977	7.088250882	-3.591602155	0.00074815	0.025572898	-0.722586329
ILMN_1698209	0.12351993	8.592883328	4.820631604	1.37346E-05	0.002287507	3.017398721
ILMN_1659888	0.123509478	10.85665344	3.615336731	0.000695896	0.024558061	-0.655670781
ILMN_1808777	-0.123417893	8.520059408	-3.873139511	0.000312529	0.015706645	0.086810562
ILMN_1759595	-0.123113566	7.28027052	-8.158142375	9.38903E-11	1.07899E-06	14.28666816
ILMN_1897310	-0.122985531	8.603913822	-5.064035827	5.93976E-06	0.001383643	3.809070619
ILMN_2246510	-0.122975266	7.998612814	-3.63245248	0.000660401	0.023919797	-0.60725952
ILMN_2408645	0.122937063	8.748485732	5.837429442	3.89566E-07	0.000244194	6.389998245
ILMN_1810228	0.122751038	8.282954419	4.765739032	1.65653E-05	0.002483069	2.840649544
ILMN_2377185	-0.122673652	8.479247604	-3.4874295	0.001025218	0.030580526	-1.013238569
ILMN_1709085	0.122619109	6.344561293	4.248313781	9.35132E-05	0.007224229	1.213468482
ILMN_1791002	0.122602198	7.7818959	3.424376856	0.00123786	0.034129975	-1.186684466
ILMN_3245983	-0.12257454	7.95500833	-3.331418938	0.001629218	0.040776416	-1.438858255
ILMN_1748438	0.122512976	10.48075732	4.60551449	2.85155E-05	0.003425437	2.328923817
ILMN_2411139	0.122288548	6.792350952	4.10476712	0.000149179	0.009825188	0.776451629
ILMN_1787815	0.122247788	7.582959281	3.751743689	0.000457043	0.019333742	-0.266296782
ILMN_2324056	0.122214788	9.370994725	3.715225012	0.000511853	0.020687757	-0.371325666
ILMN_1651347	-0.122189357	9.178162101	-5.401891838	1.82422E-06	0.00070665	4.92627448
ILMN_2143795	0.12217211	10.18028915	3.955018571	0.000241134	0.01339979	0.328290575
ILMN_1759252	-0.121980201	10.14330626	-4.910903246	1.00783E-05	0.001858065	3.309548893
ILMN_1768662	0.121939828	7.89357845	4.270013961	8.70913E-05	0.006982696	1.280129918
ILMN_1672022	-0.121822376	6.385237268	-3.410086869	0.001291575	0.035144692	-1.225727023
ILMN_3240370	0.121802105	7.789923862	3.650199462	0.000625431	0.023310659	-0.556925692
ILMN_2111187	0.121652782	8.370280836	3.875261395	0.000310445	0.01567043	0.09303544
ILMN_1794132	0.121519239	11.12478003	3.962678826	0.000235326	0.01326011	0.351014409
ILMN_1710752	0.121472439	8.511522427	4.511957613	3.90471E-05	0.004079352	2.033203661
ILMN_1712803	0.121449897	9.548288256	5.181402868	3.94957E-06	0.00107217	4.194985244
ILMN_2090782	0.121388893	8.613407829	3.337135894	0.0016021	0.040464478	-1.423473615
ILMN_2195821	-0.121306263	7.004528065	-3.479098304	0.001051173	0.031056754	-1.036264968
ILMN_3251737	-0.121253818	8.954101382	-3.488912726	0.001020662	0.030580526	-1.00913567
ILMN_1798288	-0.12076731	7.041443625	-5.042707507	6.39532E-06	0.001468809	3.739216503
ILMN_1801934	-0.120607179	7.979873922	-3.25540782	0.002033691	0.04726824	-1.64182559
ILMN_1689059	-0.120469941	7.465566472	-3.779196159	0.0004196	0.018404739	-0.186973589
ILMN_1770665	-0.120337567	6.553792707	-5.040025816	6.45499E-06	0.001468809	3.730439697
ILMN_2080611	0.120276339	8.138754828	3.803647067	0.00038875	0.017715823	-0.116060355
ILMN_1764729	-0.120270755	8.524975381	-4.24911469	9.32682E-05	0.007224229	1.215926104
ILMN_1740185	-0.120245918	8.25080104	-3.508102417	0.000963427	0.029550814	-0.955959714
ILMN_1770692	0.120115128	9.212045189	4.262789356	8.91802E-05	0.007073475	1.25791953
ILMN_1791568	0.119972104	7.620487626	4.241563774	9.5603E-05	0.007251799	1.192764099
ILMN_2243308	-0.119920158	8.155540478	-4.769386942	1.63606E-05	0.0024739	2.852373625

ILMN_1779353	0.119768202	8.683326861	3.434112218	0.00120249	0.033496112	-1.160029059
ILMN_1717809	-0.119720567	6.697764307	-4.339780157	6.92206E-05	0.006166537	1.495463675
ILMN_1684108	-0.119694623	9.051949167	-3.249860441	0.002066657	0.047754736	-1.65652175
ILMN_2235137	0.119675823	8.759065116	3.483267799	0.001038106	0.030826657	-1.024745106
ILMN_1790577	0.119656662	8.636032548	3.252130776	0.002053105	0.047505258	-1.650509097
ILMN_1805271	-0.119647686	8.035720829	-4.867950651	1.16799E-05	0.002108254	3.170314829
ILMN_1677098	-0.119383546	6.765280901	-4.268753833	8.74522E-05	0.006995362	1.276254733
ILMN_1838313	0.119201553	7.675533308	3.631865521	0.000661589	0.023919797	-0.608921868
ILMN_1696601	0.119145331	8.966240656	5.306680515	2.54872E-06	0.000820235	4.609549082
ILMN_1732772	0.119079508	8.22064226	4.999578192	7.42424E-06	0.001514546	3.598231559
ILMN_1682197	0.118914937	7.729660919	4.837026921	1.29854E-05	0.002206412	3.070324769
ILMN_1654385	0.118742083	6.037031665	4.482788948	4.30479E-05	0.004430207	1.941498615
ILMN_1665526	-0.118404939	6.424291649	-3.645184646	0.000635129	0.023519542	-0.571162757
ILMN_1754149	-0.11831732	8.468934902	-3.814940332	0.000375249	0.017295556	-0.083224489
ILMN_1673721	0.118145598	7.484523037	3.885195769	0.000300864	0.015384685	0.122203126
ILMN_2077094	0.118050009	8.705852975	3.461758634	0.001107214	0.031916643	-1.084083959
ILMN_1768004	-0.118013334	8.51597427	-5.792003072	4.58046E-07	0.000281993	6.236372127
ILMN_2285996	0.117684779	10.7801623	4.492809771	4.16302E-05	0.004323582	1.972976618
ILMN_1712413	0.117647405	8.579679455	3.974108613	0.00022691	0.01305066	0.38496169
ILMN_1667361	-0.117585881	6.543923284	-5.255590517	3.04808E-06	0.000921803	4.440172312
ILMN_1695271	0.1173988	8.107912829	4.966350125	8.32657E-06	0.001621847	3.489866581
ILMN_1656463	0.117398385	8.129104427	4.800408241	1.47174E-05	0.002349659	2.952199882
ILMN_2112460	0.11738372	7.469196009	5.680738992	6.80481E-07	0.000372997	5.860937182
ILMN_1805737	0.117376259	9.318393293	3.234462554	0.002160826	0.049075527	-1.69722973
ILMN_2351638	-0.117120259	6.26908067	-3.620424172	0.000685159	0.024488264	-0.641294768
ILMN_1714352	-0.117020964	7.136768015	-3.479156096	0.001050991	0.031056754	-1.03610535
ILMN_1733094	0.116959599	7.830280234	3.425212742	0.001234785	0.034083618	-1.184397613
ILMN_2317730	-0.116899678	7.618262486	-6.4321735	4.61562E-08	5.48717E-05	8.414483744
ILMN_2061732	0.116856246	9.634741661	3.443856162	0.001168053	0.033143858	-1.133304262
ILMN_1773369	0.116850026	8.324905122	4.153616215	0.000127348	0.008816158	0.924382533
ILMN_3239771	0.116847509	9.107843621	3.573816949	0.000789744	0.026205225	-0.77256207
ILMN_1712985	0.116482184	8.01608163	5.525004814	1.18166E-06	0.000512766	5.337727331
ILMN_2108357	0.116326695	9.510669231	3.710021855	0.000520159	0.020880692	-0.386244004
ILMN_1741133	0.116236495	12.20590476	3.393986887	0.001354746	0.036070429	-1.269595242
ILMN_1787879	0.116224441	8.903314854	4.144580202	0.000131138	0.00895267	0.89695625
ILMN_1702322	-0.116212027	6.437327887	-3.404411975	0.001313515	0.035472325	-1.241204112
ILMN_1813775	-0.116068779	8.530452061	-4.763502801	1.6692E-05	0.002491232	2.833464038
ILMN_1652008	0.115689282	7.747765968	5.65829535	7.36932E-07	0.000373624	5.785362596
ILMN_1655126	-0.115644397	7.973173718	-3.700072849	0.000536401	0.021256287	-0.414737224
ILMN_1715616	0.115547604	9.021956762	5.026576292	6.76258E-06	0.001475612	3.686442429
ILMN_1700515	0.115506883	7.735061101	4.270027969	8.70873E-05	0.006982696	1.280172998
ILMN_1697317	-0.11544122	5.879137264	-3.570517083	0.000797701	0.02631408	-0.781818765
ILMN_2311537	0.115407475	11.27813446	3.584223883	0.000765145	0.02576088	-0.743336393
ILMN_1704702	0.11540649	7.341246033	3.706682727	0.000525557	0.020981412	-0.395811752
ILMN_1786379	0.115382687	6.061254446	3.973287373	0.000227505	0.01305066	0.382520906
ILMN_2253648	0.11533359	7.430883504	4.145972957	0.000130546	0.008929994	0.901181738
ILMN_1720819	0.115311169	10.45167592	4.688557094	2.1535E-05	0.00287032	2.593344812
ILMN_1689774	-0.114940158	9.864237144	-3.991893862	0.00021439	0.012591653	0.437882855
ILMN_1755862	0.114934672	8.186321901	3.947054532	0.000247319	0.01362074	0.304689233
ILMN_1795704	-0.114760925	6.093777319	-5.166198862	4.16451E-06	0.00111299	4.144852925
ILMN_1676846	0.114758878	9.847005999	4.556068868	3.36778E-05	0.003769727	2.172337285
ILMN_2227968	0.114521511	7.650799301	5.05844093	6.05607E-06	0.00140127	3.790737853
ILMN_2371053	-0.114449769	7.798919946	-4.323758695	7.29783E-05	0.006321605	1.445878068
ILMN_1760802	0.114397377	6.722675556	4.302283958	7.83279E-05	0.006509608	1.379540579

ILMN_2357781	-0.114044211	6.984021562	-3.754072299	0.000453746	0.019291319	-0.259580541
ILMN_1703041	-0.114034624	5.961960475	-4.559976452	3.32386E-05	0.003769727	2.184688093
ILMN_1674421	-0.113869894	8.956568774	-4.767403545	1.64716E-05	0.002479805	2.84599876
ILMN_1686906	-0.113728603	6.619970145	-3.376147355	0.001428167	0.037442953	-1.318054355
ILMN_1743763	-0.11365086	8.513738595	-3.884827437	0.000301214	0.015384685	0.121120996
ILMN_1732750	0.113650115	7.464403489	4.282679178	8.35437E-05	0.006857741	1.31910668
ILMN_1749829	0.113573624	9.291546732	3.474093622	0.001067064	0.031176358	-1.050081466
ILMN_1731070	0.113383872	8.203436146	4.091640639	0.000155638	0.010071639	0.736842614
ILMN_3273047	0.113000352	6.289676893	3.73705839	0.00047837	0.019732794	-0.308599989
ILMN_2398926	0.112973331	8.502134182	5.133473613	4.66706E-06	0.001174463	4.037086398
ILMN_3282768	0.112920632	8.488820036	3.229774988	0.002190289	0.049613941	-1.709597755
ILMN_1737728	0.112816606	8.578393675	3.641546968	0.000642253	0.023681622	-0.581483191
ILMN_1780382	0.112813887	9.142404109	4.439297021	4.97668E-05	0.004863867	1.805212027
ILMN_1683598	0.112782877	8.471195899	3.479070768	0.00105126	0.031056754	-1.036341019
ILMN_1722642	-0.112751112	7.24796175	-3.629907064	0.000665568	0.02400222	-0.61446738
ILMN_1676600	-0.112530622	9.628447257	-3.607723882	0.000712264	0.024854261	-0.677161445
ILMN_1663716	-0.112470797	6.101306713	-4.276218713	8.53354E-05	0.006955134	1.299218402
ILMN_1662658	0.112443675	9.094334708	3.730064461	0.00048886	0.019992792	-0.328715054
ILMN_1684217	0.112442672	8.660205276	4.247815117	9.3666E-05	0.007224229	1.211938416
ILMN_3246754	0.112062568	6.658273383	5.3282061	2.36338E-06	0.000807064	4.681035293
ILMN_1730291	-0.111754638	8.709407451	-3.326360568	0.001653573	0.04119118	-1.452456848
ILMN_1796180	-0.111733681	7.129997011	-5.522943343	1.1903E-06	0.000512766	5.330821186
ILMN_2338963	0.111665615	7.851580883	3.340519274	0.001586254	0.04021154	-1.414361003
ILMN_1781039	-0.111528208	8.572213576	-4.781862454	1.56792E-05	0.002407453	2.892492627
ILMN_2382505	-0.111513532	6.686503073	-4.015616286	0.000198726	0.012019778	0.508653974
ILMN_1810069	0.11150471	7.707761534	4.251394147	9.25743E-05	0.007220796	1.22292186
ILMN_1795839	-0.111435682	7.585746898	-3.883718248	0.000302271	0.015415813	0.117862608
ILMN_1725791	0.111392868	7.771938699	3.873376895	0.000312295	0.015706645	0.087506876
ILMN_1794740	-0.111314023	8.18694178	-3.659012455	0.000608731	0.022886172	-0.531878819
ILMN_1719870	0.111309716	8.180480194	3.894628924	0.000292031	0.015162742	0.149935171
ILMN_2070349	0.111135329	6.622780832	3.935294789	0.000256734	0.013982871	0.269883651
ILMN_1689800	0.111079357	8.066725921	4.447193756	4.84752E-05	0.004788625	1.829916719
ILMN_1757230	-0.111063295	6.625258517	-5.416554877	1.73245E-06	0.000686528	4.975170272
ILMN_2316540	0.111024394	10.15592311	3.827471962	0.000360796	0.016988542	-0.046727219
ILMN_1709484	0.110995857	6.939208813	4.713942254	1.97575E-05	0.002791642	2.674523014
ILMN_1727740	0.110981004	9.924555301	3.839066457	0.000347902	0.016592727	-0.012902645
ILMN_1671933	0.110868409	10.03989744	3.555969899	0.0008337	0.027039163	-0.822566863
ILMN_1721868	0.110856354	9.594540838	3.492662579	0.001009229	0.030437914	-0.998758199
ILMN_1757697	0.110488038	7.179199199	4.037381014	0.000185333	0.011430293	0.573767223
ILMN_1668996	0.110447744	10.9740981	4.356257228	6.55529E-05	0.005933454	1.546542117
ILMN_1781198	-0.110344572	7.040246259	-4.661361281	2.36132E-05	0.003004023	2.506554882
ILMN_1776788	-0.110283692	6.904600806	-3.700256847	0.000536096	0.021256287	-0.414210651
ILMN_3249406	0.110239838	7.633908106	4.439634523	4.97109E-05	0.004863867	1.80626752
ILMN_1769839	-0.11021839	5.604133267	-3.819638523	0.000369766	0.017174075	-0.069548892
ILMN_1661589	-0.110142689	9.039459339	-3.562561506	0.0008172	0.026679717	-0.804115112
ILMN_1769091	0.110130474	10.25774921	3.891653206	0.00029479	0.015237156	0.14118324
ILMN_1670931	0.109982408	8.344569045	4.645968795	2.48753E-05	0.003084892	2.457515921
ILMN_1757415	0.109798743	7.754264264	4.346003319	6.78124E-05	0.006104183	1.514745478
ILMN_2390609	-0.1096809	7.612098975	-3.723432303	0.000499011	0.020263719	-0.347770486
ILMN_1882000	0.109602426	7.486804543	4.826208903	1.34751E-05	0.002255179	3.035396058
ILMN_1805481	0.109382783	8.210687973	3.24891342	0.002072335	0.047830793	-1.659029
ILMN_1799667	0.109126434	7.599247815	4.779955478	1.57815E-05	0.002407453	2.886357763
ILMN_1668498	0.109033111	8.076128406	4.450890769	4.78818E-05	0.004771017	1.841488936
ILMN_1686097	0.108979349	10.63696681	3.782293407	0.000415566	0.018351643	-0.1780045

ILMN_1745813	-0.108717332	9.21089245	-3.561996592	0.000818602	0.026700197	-0.805697245
ILMN_2409298	0.108432472	9.038152177	3.586093906	0.000760803	0.025703058	-0.73807962
ILMN_1789123	0.108426667	9.000204231	4.189342607	0.000113379	0.008026385	1.033092785
ILMN_1798690	-0.108315309	6.199460696	-3.478075568	0.001054402	0.03106853	-1.039089417
ILMN_1739083	-0.10824969	7.948387784	-4.112318676	0.000145581	0.009652032	0.799265869
ILMN_1788211	-0.108230377	7.375675481	-3.310614511	0.001731617	0.042400013	-1.494704572
ILMN_1798459	0.10804744	6.719282624	3.443015763	0.001170986	0.033168616	-1.135611035
ILMN_1656057	-0.107983713	10.21880809	-3.476355443	0.001059854	0.031123962	-1.043838699
ILMN_1753502	-0.107965539	5.861392599	-5.357683691	2.13101E-06	0.000765299	4.779045051
ILMN_2263144	0.107941111	7.580538206	3.761821749	0.000442938	0.018993463	-0.237213014
ILMN_1743620	0.107590924	6.116471599	4.715564207	1.9649E-05	0.00278773	2.679715194
ILMN_1809495	0.107530362	12.22347304	4.222304395	0.000101819	0.007390097	1.133771588
ILMN_2072391	0.107484488	8.267507843	3.789414697	0.000406431	0.018103496	-0.157367445
ILMN_2326512	0.107427063	8.422317328	3.668960444	0.000590394	0.022452432	-0.503565369
ILMN_2048507	0.107324466	6.934676879	3.79189636	0.000403293	0.018033631	-0.150170825
ILMN_1734316	0.107294264	7.968123315	4.55674145	3.36018E-05	0.003769727	2.174462838
ILMN_2219712	0.107291688	10.6231244	4.379146596	6.07704E-05	0.005632046	1.61763627
ILMN_2155172	0.107286873	9.350677952	3.585604782	0.000761937	0.025703058	-0.739454735
ILMN_2214713	0.107107885	6.939689865	5.307673797	2.53986E-06	0.000820235	4.612846176
ILMN_1683096	0.106992877	7.12992344	4.890468319	1.08113E-05	0.001982616	3.243257519
ILMN_1729987	-0.106984707	8.692391089	-5.241739825	3.19936E-06	0.000934754	4.394326346
ILMN_2401436	0.106948257	6.733041756	4.685923618	2.17282E-05	0.00287032	2.584932485
ILMN_1714710	-0.106891542	7.440476788	-4.585994124	3.04537E-05	0.003595624	2.267027858
ILMN_1672094	-0.106794829	6.75480312	-3.357602659	0.001508487	0.038902714	-1.368261796
ILMN_1792986	0.106733964	7.065097729	4.642650536	2.51559E-05	0.003108508	2.446952215
ILMN_2405521	0.106707457	9.70985749	4.181024432	0.000116492	0.008229868	1.007743142
ILMN_1761131	0.106697847	9.413357388	4.454721982	4.72743E-05	0.004737875	1.853485397
ILMN_1728262	0.106644301	5.633907349	3.463121336	0.001102709	0.031813375	-1.080331118
ILMN_1786125	0.106629104	9.58544411	3.53261434	0.000894761	0.028223047	-0.887784759
ILMN_1664912	-0.10659609	6.625301862	-3.432352287	0.001208812	0.033515316	-1.164851146
ILMN_1751708	-0.106485841	12.29033155	-3.597291252	0.000735294	0.025248984	-0.706569981
ILMN_2048478	-0.106445995	6.143759477	-4.049927436	0.000178014	0.011038134	0.61138077
ILMN_2344971	0.106370364	7.690905689	4.399733744	5.67608E-05	0.005346678	1.681715027
ILMN_1705301	0.106352558	7.378086814	3.759484864	0.000446171	0.01908461	-0.243960699
ILMN_2347592	0.106340975	7.731767718	3.390091556	0.001370465	0.036422403	-1.280189875
ILMN_3244521	-0.106218996	8.874954366	-3.371319231	0.001448678	0.037865528	-1.331142355
ILMN_1719543	-0.10618849	6.558594015	-3.811150591	0.000379728	0.017435104	-0.094249199
ILMN_1823231	-0.106154613	6.043474121	-3.335647945	0.001609117	0.040600074	-1.427479353
ILMN_1775939	0.106129895	9.10934211	3.597827939	0.000734092	0.025232845	-0.705058307
ILMN_3243248	0.106090879	6.648626861	4.570820577	3.20488E-05	0.003707769	2.21898506
ILMN_2077550	0.106059401	9.130640182	3.785144042	0.000411885	0.01828095	-0.169746051
ILMN_1670542	0.105890642	9.474264184	3.469227443	0.001082735	0.031474169	-1.063504183
ILMN_1773470	-0.1058481	7.148702109	-3.977934931	0.000224158	0.013032152	0.39633713
ILMN_1705907	0.10582135	8.640686952	4.665054611	2.33199E-05	0.00298876	2.518330446
ILMN_1701374	0.105685252	8.063288812	4.666492055	2.32067E-05	0.002985349	2.522914431
ILMN_1700257	0.105617109	7.44588105	4.305882683	7.74055E-05	0.006493021	1.39064727
ILMN_2048633	0.105414215	6.738295177	3.549354414	0.000850579	0.027457456	-0.84106541
ILMN_2063114	0.105409827	7.510759563	3.475327703	0.001063124	0.031166904	-1.046675628
ILMN_1716279	0.105328891	8.575243615	3.434628518	0.001200641	0.033496112	-1.158614147
ILMN_1787415	-0.105314288	7.716048692	-4.014179095	0.000199642	0.012032997	0.504360473
ILMN_1814122	0.105295986	8.830414732	4.381441546	6.03101E-05	0.005614129	1.624773129
ILMN_2404407	-0.105147323	6.015306341	-4.018407689	0.000196957	0.011975832	0.516995258
ILMN_2202948	0.105045018	8.434508364	3.940033341	0.000252899	0.013839621	0.283902084
ILMN_1799604	0.105020783	10.66304612	5.214172146	3.52285E-06	0.000995522	4.30317175

ILMN_3256325	-0.104841766	7.767774337	-4.249892624	9.30308E-05	0.007224229	1.218313428
ILMN_1809488	0.104793375	10.62056369	4.685902594	2.17297E-05	0.00287032	2.584865333
ILMN_1700546	0.104695806	7.170142048	3.633674665	0.000657934	0.023919797	-0.603797635
ILMN_1698996	0.104681151	6.577959557	4.059203893	0.000172783	0.010808082	0.639227797
ILMN_1716524	-0.104623689	10.43269012	-4.912416184	1.0026E-05	0.001858065	3.31446047
ILMN_1765258	-0.104618718	9.497473414	-4.14715399	0.000130047	0.008929994	0.904765403
ILMN_1653134	-0.104513062	7.862712129	-3.75557476	0.000451631	0.019270331	-0.255245896
ILMN_1761069	0.104377687	7.418121548	3.922833775	0.000267087	0.01437401	0.233060444
ILMN_1706817	-0.104207745	8.933567806	-4.152966325	0.000127617	0.008817074	0.922409038
ILMN_1714473	-0.104006181	6.555985411	-4.701906762	2.05816E-05	0.002851861	2.636015311
ILMN_2249920	-0.103982212	6.030495244	-3.694156577	0.00054629	0.021487249	-0.431660826
ILMN_1671895	-0.103872918	6.998514485	-3.83393585	0.000353552	0.016789339	-0.027876837
ILMN_1728083	-0.103775293	7.764652866	-3.961572813	0.000236156	0.01326011	0.347732092
ILMN_1703477	-0.10356919	8.719924018	-4.533611788	3.63143E-05	0.003924673	2.101437281
ILMN_1773760	0.103461457	10.87738174	3.501903363	0.000981573	0.029894623	-0.973156653
ILMN_1704253	-0.103438779	7.390643803	-4.17739727	0.000117875	0.008293606	0.996696677
ILMN_1690802	0.10328138	11.86293949	4.060457733	0.000172088	0.010801677	0.642994087
ILMN_2306189	-0.103225095	8.775216014	-4.307953936	7.68794E-05	0.006480173	1.397041599
ILMN_1803312	0.103150136	10.1723036	4.228339368	9.98298E-05	0.007338448	1.152243918
ILMN_1744308	0.103131928	8.517396068	5.650338046	7.58042E-07	0.000378757	5.758581223
ILMN_1777853	-0.102989695	6.925166684	-3.616424857	0.000693586	0.024558061	-0.652596942
ILMN_1679177	0.102748392	7.841529653	3.612938143	0.000701014	0.024636243	-0.662444664
ILMN_1815184	0.102649319	9.900279107	3.36961523	0.001455984	0.038027652	-1.335758771
ILMN_1750518	0.102645677	9.345884188	3.671996176	0.000584905	0.022356075	-0.494916626
ILMN_1654493	0.102595153	7.359277742	4.133931735	0.000135744	0.009176294	0.864671927
ILMN_1655117	0.102468039	7.80741635	3.655304988	0.000615704	0.023047779	-0.542419742
ILMN_1671257	0.10242845	10.84110977	4.684228012	2.18534E-05	0.002875057	2.579516987
ILMN_3245893	0.102396214	6.436412395	6.388559721	5.40026E-08	6.00579E-05	8.265445551
ILMN_3251423	0.102381138	6.070936542	5.404967091	1.80458E-06	0.00070665	4.936526765
ILMN_1802251	-0.102365574	9.78416461	-4.309521898	7.64835E-05	0.006462855	1.401883077
ILMN_1785413	0.102281946	6.579484474	4.312501463	7.57366E-05	0.006438484	1.411085365
ILMN_2350574	-0.102227862	7.01952651	-3.738533764	0.000476184	0.019708206	-0.304354059
ILMN_1678095	0.102179595	6.227519664	3.816296574	0.000373658	0.017268413	-0.079277631
ILMN_1798256	0.102102179	10.07763073	3.267272767	0.001964842	0.04627043	-1.610339165
ILMN_1815885	-0.101989378	6.751585841	-4.434541414	5.05608E-05	0.004896446	1.790343022
ILMN_1744239	0.101799523	7.234171366	4.284780058	8.29689E-05	0.00682682	1.325577028
ILMN_1682095	-0.101710121	7.826886844	-4.729445447	1.87436E-05	0.002692517	2.72417801
ILMN_1688526	0.101658469	11.46092375	4.719370495	1.93965E-05	0.002763285	2.691902383
ILMN_1788416	0.101648297	10.07321808	3.796696573	0.00039729	0.017952992	-0.136243405
ILMN_1734483	-0.101560847	8.371627796	-4.994295856	7.56097E-06	0.001524397	3.580989602
ILMN_1690371	0.101476765	9.478240738	3.997696529	0.000210451	0.012381439	0.455174651
ILMN_1748923	0.101455685	7.985495475	3.484667982	0.001033753	0.03075036	-1.020874704
ILMN_1801118	0.10129261	9.449758515	3.240503941	0.002123406	0.048513279	-1.681272675
ILMN_1804988	-0.101266749	8.036671177	-3.587061413	0.000758566	0.025703058	-0.735359265
ILMN_2198515	-0.101190742	6.781700792	-3.290032434	0.001838898	0.044148923	-1.549737243
ILMN_1682226	-0.101024286	6.776447359	-4.448001022	4.8345E-05	0.004788625	1.832443247
ILMN_1691578	0.10098201	8.624275051	3.502073176	0.000981072	0.029894623	-0.97268581
ILMN_1661293	-0.100978705	6.006452296	-3.808336773	0.000383087	0.0175396	-0.102431068
ILMN_1777895	-0.100955108	7.506302808	-4.707928858	2.01651E-05	0.002834636	2.65527862
ILMN_1798654	0.100850085	10.43061867	4.195554523	0.000111107	0.007897983	1.052038783
ILMN_2205999	0.100567506	6.563454149	3.924201188	0.000265932	0.01437401	0.237098327
ILMN_1797307	0.100500719	7.472940786	3.84880911	0.000337412	0.016315041	0.015561403
ILMN_2278636	-0.100496028	8.45339744	-4.63963174	2.54138E-05	0.003118034	2.437344314
ILMN_1723625	0.100413602	8.226136828	3.366764234	0.001468285	0.038239954	-1.343479359

ILMN_1752988	-0.100389889	8.508428044	-4.272111209	8.64939E-05	0.006982696	1.286580571
ILMN_2051373	0.100313749	9.100051492	3.47603434	0.001060875	0.031127423	-1.04472511
ILMN_2312719	0.100211617	9.234985365	3.783764902	0.000413662	0.018307329	-0.173741916
ILMN_2124816	-0.100202759	6.475060161	-3.470442192	0.001078802	0.031439387	-1.060154513
ILMN_1798177	-0.100198997	9.656603651	-4.672406464	2.27465E-05	0.002948155	2.54178088
ILMN_1743911	0.100016016	10.10491784	3.523956448	0.000918463	0.028760166	-0.911896899
ILMN_1712122	0.099963232	5.943253436	5.641958129	7.80922E-07	0.000384615	5.730385119
ILMN_1754234	0.099931879	8.750926766	3.968837865	0.000230754	0.013127837	0.369301066
ILMN_1721741	-0.099880409	8.567651102	-3.635809623	0.000653646	0.023868572	-0.597748698
ILMN_1758633	-0.099746357	8.386412058	-3.754032877	0.000453801	0.019291319	-0.259694262
ILMN_2364357	0.099685017	9.160358027	3.693607794	0.000547216	0.021487249	-0.433229867
ILMN_3241979	0.099636405	8.523769598	3.547640196	0.000855006	0.02749737	-0.845855509
ILMN_1730825	0.099587965	7.212852178	3.447848997	0.001154214	0.032859361	-1.122339861
ILMN_2226304	-0.099562289	7.526338806	-3.381135084	0.001407267	0.037092467	-1.304521571
ILMN_1656670	0.099523586	6.613323418	4.3259752	7.24468E-05	0.006307263	1.452733281
ILMN_1659364	0.099514478	9.22749337	4.065615932	0.000169255	0.010687232	0.658494258
ILMN_1676548	0.099426108	10.23763896	3.456282523	0.001125496	0.032201321	-1.099156064
ILMN_1665205	-0.099313355	6.758934227	-3.645793666	0.000633943	0.023517303	-0.56943434
ILMN_1797298	-0.09923338	6.449558603	-4.541266957	3.53936E-05	0.003886087	2.125590168
ILMN_1737611	-0.099132363	6.669950183	-5.737284991	5.56564E-07	0.000325222	6.051582352
ILMN_3196019	0.099108976	7.91190747	3.5235516	0.000919586	0.028769196	-0.913023545
ILMN_1674302	0.09899494	8.246590724	3.666251651	0.000595334	0.022553434	-0.511279281
ILMN_1672504	-0.098872082	9.839357917	-3.416724878	0.001266353	0.034677354	-1.207603278
ILMN_1765858	0.098839649	11.04109625	4.465214308	4.56489E-05	0.004628802	1.886361105
ILMN_1705201	-0.098808357	6.14596149	-3.526426249	0.000911642	0.028624549	-0.905022078
ILMN_1805104	-0.0986951	5.880777654	-3.545012021	0.000861836	0.027588339	-0.853196881
ILMN_1733956	0.098658309	9.365748601	3.550935371	0.000846516	0.02737756	-0.83664649
ILMN_1741736	0.098593406	8.045154438	4.992302847	7.61319E-06	0.001526003	3.57448572
ILMN_1780937	0.098485109	7.910152734	4.655286052	2.41037E-05	0.003034838	2.487192575
ILMN_1753482	0.098298673	6.521849759	3.730084351	0.000488829	0.019992792	-0.328657877
ILMN_2376667	-0.098296982	7.415980103	-4.784301133	1.55493E-05	0.002403936	2.900339251
ILMN_2041046	0.098246719	10.23293506	4.331497728	7.1139E-05	0.006248444	1.469820027
ILMN_1712320	-0.098245882	8.823535485	-4.569535801	3.21876E-05	0.003711367	2.214920012
ILMN_1657797	0.098199715	8.498497899	3.54963681	0.000849852	0.027457456	-0.840276171
ILMN_1655177	-0.098178945	8.835962518	-3.801257836	0.000391665	0.017767168	-0.123000511
ILMN_2320906	0.098165167	8.239527803	3.781758081	0.00041626	0.018351643	-0.179554993
ILMN_2336781	0.098162947	9.712383469	3.624481855	0.00067671	0.024327676	-0.629820358
ILMN_1778059	0.098097957	6.052982091	4.522755737	3.76601E-05	0.004007313	2.067212955
ILMN_1685005	-0.098089677	9.236945181	-5.082754721	5.56638E-06	0.001323493	3.87045003
ILMN_1721713	0.09806712	8.74185298	3.77137613	0.000429954	0.018728635	-0.209601333
ILMN_1792078	-0.097898063	9.804828113	-4.491198712	4.1855E-05	0.004333315	1.967913945
ILMN_2371700	0.097897091	7.614581369	4.014896193	0.000199185	0.012026426	0.506502653
ILMN_3306997	0.097870416	8.643978954	4.267601117	8.77836E-05	0.007005617	1.272710311
ILMN_2214355	0.097869593	6.543851181	3.778686537	0.000420267	0.01841059	-0.188448986
ILMN_1798705	0.097800414	8.716211037	3.975846425	0.000225656	0.01305066	0.390127431
ILMN_1713406	-0.097777052	8.336282087	-5.218665346	3.468E-06	0.000988122	4.318020158
ILMN_2210713	0.097758339	6.990188826	5.14190432	4.53214E-06	0.001166046	4.064830989
ILMN_1728934	0.097548201	10.36563148	3.743648959	0.000468683	0.019597123	-0.289626097
ILMN_2377862	0.097461321	7.906293065	3.721934397	0.000501331	0.020334002	-0.352071662
ILMN_1682336	0.097405863	7.018466589	3.631372527	0.000662589	0.023919797	-0.610317977
ILMN_3231638	-0.097282649	7.788437121	-3.246059237	0.002089537	0.048025917	-1.666582663
ILMN_1675130	0.097201396	7.106587477	3.365542387	0.001473587	0.038313275	-1.346786921
ILMN_1708787	-0.097157534	6.527560153	-4.122176179	0.000141012	0.009403324	0.829076734
ILMN_1701269	0.097151578	10.91990176	3.826683664	0.000361689	0.016988542	-0.049024942

ILMN_1741219	-0.097142671	7.243370215	-3.747002852	0.000463826	0.019524851	-0.279963395
ILMN_2327994	0.096958564	7.877694774	3.396450785	0.001344891	0.035998816	-1.262890008
ILMN_1716445	0.096808043	9.710094884	3.753547617	0.000454487	0.019296654	-0.26109404
ILMN_1667977	0.096763496	7.87598747	5.017968723	6.96697E-06	0.001482674	3.658303228
ILMN_1694514	-0.096734481	6.798093916	-3.708686193	0.000522312	0.020938623	-0.390071707
ILMN_2367215	0.096733243	8.953831256	3.391490293	0.0013648	0.03630622	-1.27638641
ILMN_1710064	-0.096412025	6.052733143	-3.464019407	0.001099749	0.031813375	-1.07785737
ILMN_1915076	0.096395757	6.374949152	3.247043477	0.00208359	0.047921176	-1.66397833
ILMN_2061768	0.096374248	5.620462668	4.723059332	1.91549E-05	0.002740187	2.703716891
ILMN_1797522	-0.096373323	9.338908942	-3.839006399	0.000347968	0.016592727	-0.013077994
ILMN_3282436	-0.096372474	9.636964824	-3.459634436	0.001114272	0.032039724	-1.089932172
ILMN_1658685	-0.096330707	5.72376934	-3.635517162	0.000654231	0.023868572	-0.598577441
ILMN_1788462	0.096224239	9.767439541	3.85065055	0.000335464	0.016287503	0.020945618
ILMN_1685774	0.0961959	11.2321337	3.463242668	0.001102308	0.031813375	-1.07999693
ILMN_1743711	0.096185424	8.799743019	3.906867016	0.000280943	0.014794866	0.185965228
ILMN_1685679	-0.096167768	7.037923805	-4.995560188	7.52802E-06	0.001524397	3.585115969
ILMN_1764970	-0.096122811	9.031091731	-4.138562095	0.000133722	0.009075194	0.878705569
ILMN_1746561	-0.095957567	9.35507481	-4.879705695	1.12183E-05	0.002046353	3.208379719
ILMN_1797318	0.095936934	7.588091256	3.631907673	0.000661504	0.023919797	-0.608802493
ILMN_1671906	0.095869271	8.23171575	3.530465944	0.000900588	0.028380862	-0.893771267
ILMN_1786718	0.095788841	10.08506444	3.519824759	0.000929984	0.029015515	-0.923391326
ILMN_2045729	0.095751769	9.02096458	3.668218007	0.000591744	0.022468038	-0.505679947
ILMN_1701289	0.095592709	8.383383115	3.254161666	0.002041053	0.047307602	-1.645128306
ILMN_1743241	-0.095559383	7.429252584	-3.546168846	0.000858823	0.027517453	-0.849965875
ILMN_1780667	0.09554555	8.102366742	3.376151516	0.001428149	0.037442953	-1.318043071
ILMN_1708059	0.09545611	6.612322187	3.952913396	0.000242754	0.013412175	0.322049566
ILMN_2129273	0.095342249	6.794773694	3.457072256	0.001122842	0.032178793	-1.096983339
ILMN_1772124	-0.095251892	7.905462812	-3.854051083	0.000331895	0.016238578	0.030892054
ILMN_1815190	0.09525165	7.766688732	4.23003086	9.92792E-05	0.007326296	1.157423524
ILMN_1691418	-0.095248988	6.015237344	-3.741684374	0.00047155	0.019645076	-0.29528395
ILMN_1787923	-0.095155903	7.990188311	-3.792904316	0.000402025	0.018023696	-0.147247112
ILMN_1657983	-0.095121782	8.205448842	-4.575859098	3.15103E-05	0.003670097	2.234931288
ILMN_1698715	0.095059013	6.978505793	5.905653966	3.05361E-07	0.000206424	6.621066928
ILMN_1802646	-0.095001082	5.62997933	-3.555037436	0.000836059	0.027090208	-0.825175478
ILMN_1810418	0.094943596	6.766561836	4.270889691	8.68413E-05	0.006982696	1.28282329
ILMN_3276822	0.0949146	6.700760752	3.91658928	0.000272426	0.014527246	0.214630043
ILMN_2049766	0.094884875	7.162144079	3.496235336	0.000998449	0.030259518	-0.98886466
ILMN_1734830	-0.094855911	6.273340911	-3.679682891	0.000571223	0.022053177	-0.47299938
ILMN_1797005	-0.094730986	9.381350127	-3.893141257	0.000293407	0.015188451	0.145559335
ILMN_1718271	-0.094653927	8.50807691	-3.508474905	0.000962347	0.029543969	-0.954925813
ILMN_1695362	-0.094637103	7.648269942	-3.580781565	0.0007732	0.025830261	-0.753008858
ILMN_1737195	0.094551571	9.0586709	3.886178255	0.000299932	0.015384685	0.125089855
ILMN_2403965	0.094487647	7.315687917	3.688651258	0.000555648	0.021645781	-0.447395341
ILMN_1786108	0.094483756	6.541233892	3.626279089	0.000672999	0.024223018	-0.624735754
ILMN_1701882	0.094480434	7.893749686	3.905601357	0.000282071	0.014801647	0.182236303
ILMN_1747935	-0.094457507	8.156604102	-4.232971595	9.8329E-05	0.007309774	1.166430744
ILMN_3187852	-0.094421077	9.630296933	-4.149410026	0.000129099	0.008901603	0.911612336
ILMN_1743204	-0.094355993	6.696148495	-4.24123408	9.57063E-05	0.007251799	1.191753206
ILMN_1717173	0.094230148	9.055677698	3.44295969	0.001171181	0.033168616	-1.135764934
ILMN_1751773	0.094187445	7.174381759	4.092573154	0.00015517	0.010071639	0.739654459
ILMN_1710150	0.09413793	7.049956004	4.745962788	1.77195E-05	0.002588545	2.77714542
ILMN_1775759	0.094075394	7.746171966	3.371685722	0.001447112	0.037853276	-1.330149284
ILMN_1695946	-0.093987687	8.595334158	-3.441389297	0.001176681	0.033170292	-1.140074483
ILMN_1781281	0.09398673	6.702514257	4.226582131	0.000100405	0.007349386	1.146863985

ILMN_2399310	0.093944797	7.05055913	3.272324671	0.001936202	0.045720888	-1.596910599
ILMN_1699737	0.093791042	8.908790451	4.236222444	9.72888E-05	0.007309774	1.176391119
ILMN_1771738	0.093527262	7.554359386	5.304366494	2.56948E-06	0.000820235	4.601868533
ILMN_2413644	0.093412964	5.945822603	3.306160687	0.001754317	0.042713153	-1.506631617
ILMN_1765523	-0.093308047	7.1158952	-3.601582641	0.000725735	0.025095744	-0.694478909
ILMN_2392546	0.093287363	10.91638617	3.350509303	0.001540328	0.03948278	-1.387420839
ILMN_3307930	0.093205049	12.34740456	3.910949243	0.000277336	0.014709914	0.197996666
ILMN_1790625	0.093125247	9.523980712	3.954718685	0.000241364	0.01339979	0.327401431
ILMN_1678300	0.093042325	9.174469051	3.433833563	0.001203489	0.033496112	-1.160792653
ILMN_2097185	-0.093015141	7.90336422	-3.509725198	0.00095873	0.029485439	-0.951454945
ILMN_3305871	0.092911538	6.858198686	3.846030619	0.000340372	0.016412138	0.007439905
ILMN_1745217	-0.092843025	8.509882894	-4.255994427	9.11892E-05	0.007161366	1.237045461
ILMN_1759175	0.092758674	6.883298574	4.177675359	0.000117769	0.008293606	0.997543436
ILMN_2100458	0.092742651	6.719360877	3.785811469	0.000411028	0.01828095	-0.167811992
ILMN_1670821	0.092721981	6.17475528	4.235259158	9.75959E-05	0.007309774	1.173439315
ILMN_3206242	0.092662631	7.38224721	3.328578588	0.001642852	0.040976769	-1.44649566
ILMN_1663390	0.092612458	10.32550312	3.286512675	0.001857863	0.044480336	-1.55912671
ILMN_1746948	-0.09255967	7.200817563	-3.464258254	0.001098963	0.031813375	-1.077199397
ILMN_1755749	0.092555118	10.965638	4.331073632	7.12386E-05	0.006248444	1.468507533
ILMN_1722089	0.092493309	7.619406197	3.99862311	0.000209829	0.012374946	0.457936999
ILMN_1746699	0.092477373	7.76776525	3.491012588	0.001014245	0.030512298	-1.003325259
ILMN_1675038	-0.092396465	8.060327569	-4.060037185	0.000172321	0.010801677	0.64173078
ILMN_3185161	0.092346418	6.903837656	4.781019095	1.57244E-05	0.002407453	2.889779382
ILMN_2347917	0.092291927	8.305680315	4.085839968	0.000158578	0.010161946	0.719358577
ILMN_1739103	0.09215513	6.763199341	3.853819884	0.000332136	0.016238578	0.030215659
ILMN_3305304	0.092123228	9.246338618	4.243859487	9.48872E-05	0.007244749	1.199804103
ILMN_1703946	0.092018041	8.504248876	3.253800691	0.00204319	0.047307602	-1.646084859
ILMN_1745904	-0.091969083	10.72793863	-3.351118687	0.001537567	0.039441351	-1.385775891
ILMN_1651433	0.091867166	8.086261462	3.607911724	0.000711856	0.024854261	-0.67663149
ILMN_1669424	0.091806685	11.80404463	3.567757382	0.000804414	0.026488041	-0.789556392
ILMN_1898692	0.091592105	6.279318107	4.844599041	1.2653E-05	0.002206412	3.094789
ILMN_1723124	-0.091535932	6.99305156	-3.842002886	0.000344708	0.01650513	-0.004327614
ILMN_2311089	0.091511021	7.284058783	3.636934457	0.000651397	0.023868572	-0.594560913
ILMN_1754178	0.09148847	10.43994563	3.816932207	0.000372915	0.017257196	-0.077427591
ILMN_1685258	0.09146999	10.63855008	4.098622611	0.000152169	0.00996623	0.757903068
ILMN_2384536	0.091352614	8.082153293	3.705596628	0.000527324	0.020993104	-0.398922768
ILMN_1658695	0.091322993	8.046686856	3.428204984	0.001223835	0.033808453	-1.176208535
ILMN_1815012	-0.091320754	8.664126614	-3.771160096	0.000430244	0.018728635	-0.210226081
ILMN_2048636	0.09127943	7.645361708	3.790072237	0.000405597	0.018089735	-0.155460881
ILMN_2400874	0.091207885	9.201289233	3.748325981	0.000461923	0.019468525	-0.276150104
ILMN_1677906	0.091189518	9.433026339	4.524611098	3.74267E-05	0.003994805	2.07305978
ILMN_1733937	0.09099438	7.646217725	3.362134865	0.00148847	0.038553688	-1.35600722
ILMN_1900270	0.090848739	6.401474752	3.24512498	0.002095197	0.048091883	-1.66905427
ILMN_1765558	-0.090838935	7.628369439	-3.459723191	0.001113976	0.032039724	-1.089687861
ILMN_1722674	0.090790745	6.699466109	3.96716017	0.000231991	0.013154782	0.36431844
ILMN_2261784	0.090729782	8.136969936	3.844571365	0.000341937	0.016418694	0.003175762
ILMN_1783546	-0.090657319	8.003032723	-3.278191269	0.001903438	0.045121052	-1.581299921
ILMN_2379469	0.090616491	11.18543483	4.408239747	5.51805E-05	0.005226381	1.708227504
ILMN_1754179	-0.090591428	8.30519787	-4.73948392	1.81145E-05	0.002633698	2.756361293
ILMN_1768969	0.090542321	7.446225794	3.649082994	0.000627578	0.023365408	-0.560096305
ILMN_2128750	-0.090427545	10.87378624	-4.657679626	2.39093E-05	0.0030305	2.494819989
ILMN_3234756	0.090421022	7.036300446	3.744771329	0.000467052	0.019597123	-0.286393031
ILMN_3179620	0.090406793	6.460924823	4.565340232	3.26448E-05	0.003739076	2.201648276
ILMN_1692790	0.090382604	8.579601874	3.690736751	0.000552085	0.021556245	-0.441436423

ILMN_1756542	-0.090344356	6.823393305	-3.811821267	0.000378932	0.017435104	-0.092298567
ILMN_2178226	0.090327671	5.761470798	3.980396684	0.000222404	0.012952056	0.403658701
ILMN_1805827	-0.090301517	12.81968424	-3.826923345	0.000361417	0.016988542	-0.04832635
ILMN_3307266	0.090183389	7.157898806	4.624264151	2.67677E-05	0.003272499	2.388470466
ILMN_1673991	0.090175209	10.46295498	3.590069897	0.00075165	0.025581318	-0.726897556
ILMN_2325394	0.09016368	6.413411088	4.202392481	0.000108657	0.007788072	1.072909125
ILMN_1781419	0.090137194	8.999328921	3.552638388	0.00084216	0.027262249	-0.831885113
ILMN_3272768	0.090119915	6.624887584	5.296918294	2.63744E-06	0.000834206	4.577152653
ILMN_2110829	0.090066063	9.062340334	4.546465496	3.47815E-05	0.003868152	2.142001281
ILMN_2350114	-0.090064528	6.477650175	-3.767265157	0.000435495	0.018838317	-0.2214865
ILMN_1766637	0.089844997	8.528574068	3.965148231	0.000233482	0.013195954	0.358344537
ILMN_1733757	0.089843143	10.38151971	3.234661605	0.002159584	0.049075527	-1.696704282
ILMN_2141259	0.089827241	6.809435194	5.095505811	5.32538E-06	0.001274985	3.912298783
ILMN_1761531	-0.089744642	7.048732444	-4.956666345	8.60928E-06	0.001667491	3.458327851
ILMN_2179837	0.08966518	10.82074589	3.73727799	0.000478044	0.019732794	-0.307968067
ILMN_1766185	0.089639124	8.206930228	3.771329395	0.000430017	0.018728635	-0.209736489
ILMN_1788149	-0.089582974	5.956010188	-3.546235147	0.000858651	0.027517453	-0.849780678
ILMN_1664167	0.089514164	10.25886212	4.340534548	6.90484E-05	0.006166537	1.497800437
ILMN_1815882	0.089405633	6.880174958	3.952919171	0.00024275	0.013412175	0.322066682
ILMN_1736130	0.089314035	7.127000602	3.620793364	0.000684386	0.024488264	-0.640251061
ILMN_1703005	-0.089199912	10.44196314	-3.24863521	0.002074006	0.047830793	-1.659765478
ILMN_1690352	-0.089152147	8.411518814	-3.259429671	0.002010102	0.046877323	-1.631160853
ILMN_1744046	0.089110426	7.48543954	3.70995841	0.000520261	0.020880692	-0.386425842
ILMN_2359453	-0.089103389	10.66598828	-3.602097305	0.000724597	0.02508154	-0.693028276
ILMN_1807243	0.089040384	7.320598484	3.68012476	0.000570446	0.022047865	-0.471738694
ILMN_1808354	0.089027635	6.543876776	4.786568787	1.54295E-05	0.002396152	2.907636842
ILMN_2298936	0.088980224	10.25467939	3.754698445	0.000452863	0.019291319	-0.257774205
ILMN_1812441	-0.088944345	8.001096042	-4.041560011	0.000182863	0.011298192	0.586289293
ILMN_1764323	0.088928388	9.198452706	4.519733703	3.80433E-05	0.004028765	2.057691625
ILMN_1658027	0.088785504	7.18346088	3.841573369	0.000345173	0.01650513	-0.005582117
ILMN_2222880	-0.088784192	6.836397064	-3.338129963	0.001597429	0.040435363	-1.420796842
ILMN_1807540	-0.08865247	7.603404083	-3.442229516	0.001173736	0.033168616	-1.137768862
ILMN_1815024	0.088600117	10.93683771	3.32237522	0.001673005	0.041479641	-1.463161687
ILMN_1722276	-0.088549179	8.935430807	-3.945919185	0.000248214	0.013643953	0.301326612
ILMN_1774890	0.088423146	7.438108109	4.614848182	2.76319E-05	0.003354361	2.358554991
ILMN_1799367	0.08839355	10.52338823	3.311506821	0.001727102	0.042349633	-1.492313811
ILMN_2395240	0.088352862	7.737161267	3.692481639	0.000549121	0.021537522	-0.43644928
ILMN_2334204	0.08829312	6.883614053	3.59660062	0.000736843	0.025277012	-0.708515076
ILMN_1752270	0.088241482	6.838127405	4.589765236	3.00695E-05	0.003562459	2.278977573
ILMN_3280667	0.088137089	6.65216831	5.066729003	5.88456E-06	0.001380109	3.817897466
ILMN_3228585	-0.088052264	8.42274286	-3.435908755	0.001196069	0.033496112	-1.155105127
ILMN_1801600	-0.08802934	8.147343777	-3.622116034	0.000681624	0.024478822	-0.636511372
ILMN_2396956	-0.088000963	8.230168174	-3.459831401	0.001113615	0.032039724	-1.089389992
ILMN_1658437	0.087993713	8.967506283	3.244519943	0.00209887	0.04811213	-1.670654673
ILMN_1687971	-0.087973652	6.275942845	-3.535200031	0.000887797	0.02811042	-0.880576893
ILMN_3248758	0.087866124	6.366764829	4.018920027	0.000196634	0.011975832	0.518526541
ILMN_1693771	0.087760792	7.76639878	3.313234073	0.001718396	0.042204627	-1.487684841
ILMN_1738767	-0.08772946	8.400322766	-4.011494315	0.000201366	0.012094573	0.496341933
ILMN_2332558	0.08768534	9.264804547	3.909933026	0.00027823	0.014712052	0.19500099
ILMN_2168992	0.087567305	7.435519657	4.613288937	2.77776E-05	0.003360218	2.353603348
ILMN_1813240	0.08753795	10.31536467	3.338601393	0.001595218	0.04040907	-1.419527225
ILMN_1664608	-0.087531002	8.508846577	-3.334824786	0.001613011	0.040600074	-1.429694916
ILMN_2054145	0.08750574	8.440683457	3.414394209	0.001275155	0.034835369	-1.213969148
ILMN_1732725	0.087491159	7.960481739	3.489470534	0.001018954	0.030580526	-1.007592395

ILMN_1671809	-0.087398657	8.469299951	-3.605120418	0.000717945	0.024926372	-0.684504928
ILMN_1797367	-0.087268098	7.058893365	-3.743073078	0.000469522	0.019597123	-0.291284759
ILMN_1693221	0.087213622	7.609533438	3.588493506	0.000755266	0.025653751	-0.731331846
ILMN_2386100	0.087129741	9.046575082	3.586667157	0.000759477	0.025703058	-0.736467852
ILMN_1669308	0.08710429	7.241028612	4.357398269	6.5306E-05	0.005933454	1.550082399
ILMN_1672759	-0.087079689	8.162982829	-3.743816307	0.000468439	0.019597123	-0.289144074
ILMN_1846771	-0.086997304	6.375978592	-3.349683403	0.001544076	0.039520098	-1.389649944
ILMN_1689968	-0.086933012	6.296840589	-3.516604704	0.000939059	0.02914041	-0.932344054
ILMN_2127477	0.086559898	7.228713871	3.279430926	0.001896582	0.045001081	-1.577998994
ILMN_1653553	0.086530761	6.729792388	3.919255789	0.000270134	0.014438959	0.222498303
ILMN_1672553	0.086431767	5.859541859	3.662729221	0.000601817	0.022725358	-0.521305414
ILMN_1801378	0.08642688	8.348091427	3.494354377	0.001004111	0.030366423	-0.994074087
ILMN_1727300	-0.086392428	7.468962948	-4.654648893	2.41557E-05	0.003034838	2.485162432
ILMN_1702231	-0.086279148	6.197290273	-3.899863161	0.000287238	0.015027045	0.16533808
ILMN_1688089	0.086247268	10.74742542	4.027275899	0.000191439	0.011743844	0.543514293
ILMN_2129388	-0.08624141	7.432400372	-3.571030498	0.000796458	0.02631408	-0.780378875
ILMN_1786976	-0.086220771	8.587619334	-3.537939804	0.000880473	0.027977144	-0.872936131
ILMN_1679881	0.086126327	7.188560734	3.337400327	0.001600856	0.040464478	-1.422761613
ILMN_2333687	-0.086109714	6.597974131	-3.850923957	0.000335176	0.016287503	0.021745153
ILMN_1665219	-0.086102396	7.186449874	-4.301433834	7.85473E-05	0.006509608	1.37691745
ILMN_1668605	0.086063104	7.037080861	4.094621492	0.000154148	0.010027156	0.745831965
ILMN_1751984	-0.085978064	7.973189984	-5.171620877	4.08657E-06	0.001100692	4.162726345
ILMN_2055165	-0.085881512	11.24431997	-3.524102343	0.000918059	0.028760166	-0.911490871
ILMN_1730799	-0.085880924	7.340899208	-3.234536776	0.002160363	0.049075527	-1.697033805
ILMN_1733690	0.085880907	6.91600418	3.76876645	0.000433464	0.018773994	-0.217146953
ILMN_2393544	-0.085836583	7.652228369	-4.423741807	5.24101E-05	0.005033123	1.756601287
ILMN_1802377	-0.085820805	6.874295034	-3.635283221	0.0006547	0.023868572	-0.599240331
ILMN_2043828	0.085792034	6.634682857	3.606667776	0.000714563	0.024876348	-0.680140727
ILMN_1668465	0.085734569	6.090793778	4.112777008	0.000145366	0.009652032	0.800651193
ILMN_3263329	0.085578594	8.816942479	3.275276863	0.001919648	0.045423334	-1.589057206
ILMN_2399523	-0.085565157	7.836649947	-3.504677722	0.000973412	0.029777598	-0.965462469
ILMN_1784367	0.08547098	7.226279907	3.645566049	0.000634386	0.023517303	-0.570080344
ILMN_3182422	-0.085396062	7.415648336	-3.409741155	0.001292901	0.035153044	-1.226670336
ILMN_1770053	0.085395849	10.21354195	4.210582682	0.000105792	0.007598501	1.097927228
ILMN_1733875	-0.085394882	7.811224776	-3.843740769	0.000342831	0.016438711	0.000749026
ILMN_1672389	0.085369632	9.105181039	4.397956934	5.70964E-05	0.005349064	1.676179586
ILMN_1740005	0.085322335	6.181645512	3.253960576	0.002042243	0.047307602	-1.645661186
ILMN_2061405	0.085310432	9.813281903	3.439962436	0.0011817	0.03323025	-1.143989111
ILMN_1814074	-0.085231063	7.334846786	-3.26108275	0.002000481	0.046758372	-1.62677495
ILMN_1755822	0.085191211	6.52315749	4.233022685	9.83126E-05	0.007309774	1.166587254
ILMN_1759954	0.085123149	11.03951657	4.471564181	4.46919E-05	0.004545129	1.906272592
ILMN_1811104	-0.085108884	9.073405271	-3.773757157	0.000426776	0.018648314	-0.202714371
ILMN_2206344	-0.085063405	6.519048067	-4.647493663	2.47474E-05	0.003081022	2.4623713
ILMN_2412172	-0.085052138	6.932102583	-3.693692526	0.000547073	0.021487249	-0.432987615
ILMN_1772713	-0.085038775	9.535363942	-3.450527553	0.001145018	0.032624501	-1.114980198
ILMN_2174380	0.084822232	6.297600137	3.844757005	0.000341738	0.016418694	0.003718181
ILMN_1694752	-0.084803384	7.069329397	-4.243551984	9.49828E-05	0.007244749	1.198861017
ILMN_1782439	-0.084661013	10.19497366	-4.27804065	8.48263E-05	0.006930029	1.304825809
ILMN_2201533	0.084621143	9.821846228	3.636453788	0.000652357	0.023868572	-0.595923199
ILMN_3307700	0.084519082	8.613779351	3.320573717	0.00168186	0.041479641	-1.467997973
ILMN_1701308	-0.084379387	6.375542286	-3.619223933	0.000687678	0.024517462	-0.644687426
ILMN_2048822	0.084166848	7.987739817	3.775982867	0.000423825	0.018542887	-0.196274522
ILMN_1761058	-0.084102987	7.335681071	-4.322973605	7.31674E-05	0.006322105	1.443450305
ILMN_1786016	0.084097975	7.078864237	4.449472421	4.81086E-05	0.004779802	1.837048822

ILMN_1677747	0.084084024	6.347556495	3.488611996	0.001021584	0.030580526	-1.009967633
ILMN_1655206	-0.084007166	6.955883342	-4.51284218	3.89316E-05	0.004079352	2.035988434
ILMN_1805658	0.083963599	9.531574837	4.212122215	0.000105262	0.007592044	1.102632437
ILMN_1709479	0.083825884	10.30830298	3.352089428	0.00153318	0.039387419	-1.383155124
ILMN_1792748	-0.083794857	6.144422072	-3.42421833	0.001238444	0.034129975	-1.187118128
ILMN_2333865	-0.083702334	7.367843472	-3.620651165	0.000684684	0.024488264	-0.640653066
ILMN_1766000	0.083671628	8.079880563	3.614131536	0.000698463	0.024596745	-0.659074711
ILMN_1724907	0.083636344	8.172696565	3.396137036	0.001346142	0.036004348	-1.263744007
ILMN_2383419	0.083611606	6.691453077	3.765309745	0.000438155	0.018882297	-0.227137291
ILMN_1728059	0.08358392	7.675855752	3.24193592	0.002114626	0.048408933	-1.677487599
ILMN_1719256	0.083579125	10.98632788	3.541396725	0.000871316	0.027788599	-0.863290411
ILMN_1744268	-0.083575133	8.450240022	-3.474457088	0.001065902	0.03116883	-1.049078443
ILMN_1781001	0.083556422	6.255321047	4.536037094	3.60201E-05	0.00391472	2.109087642
ILMN_1727281	0.083553484	7.373108918	3.730228033	0.000488612	0.019992792	-0.328244845
ILMN_2389810	0.083549919	7.036975926	4.440150829	4.96256E-05	0.004863867	1.807882262
ILMN_3309694	0.083543705	6.608769909	4.24541012	9.44067E-05	0.007244749	1.204560226
ILMN_3258795	-0.083428689	8.134017625	-3.594754224	0.000741001	0.025369155	-0.713714206
ILMN_1734153	-0.083359672	7.901925119	-3.825677733	0.000362832	0.017019032	-0.051956659
ILMN_1684771	0.083242366	9.932249583	5.324702001	2.39262E-06	0.000807064	4.669393344
ILMN_2184640	0.083220373	8.438207727	3.455299386	0.001128808	0.032269314	-1.101860467
ILMN_1712894	-0.082972218	5.769307687	-4.544174057	3.505E-05	0.003881022	2.134766593
ILMN_2332691	-0.082970114	5.726568903	-3.86731534	0.000318319	0.015904902	0.069733618
ILMN_1758173	0.0829329	9.107964049	3.915867563	0.00027305	0.014527246	0.212500895
ILMN_1737360	-0.0829233	5.933497061	-3.951771588	0.000243638	0.013439436	0.318665261
ILMN_2181125	-0.082822778	7.148499539	-3.973323648	0.000227478	0.01305066	0.382628711
ILMN_1758672	0.082821609	6.513335482	3.491767999	0.001011945	0.030469717	-1.001234492
ILMN_1813836	0.082819839	11.55781151	4.296619403	7.98014E-05	0.006597683	1.362066435
ILMN_1787280	0.082811665	8.156232987	3.59523754	0.00073991	0.025357001	-0.712353419
ILMN_1806705	-0.0827874	6.603106889	-3.481014098	0.00104515	0.030982443	-1.030972858
ILMN_2341952	0.082774316	9.937689774	3.671564491	0.000585682	0.022361007	-0.496146734
ILMN_1802894	0.082692769	8.105850678	3.580960138	0.00077278	0.025830261	-0.752507222
ILMN_3265365	0.082644807	7.309240054	3.728908513	0.000490615	0.020040789	-0.332037652
ILMN_2149053	0.082626204	8.052889189	3.832433463	0.000355223	0.016810019	-0.032259695
ILMN_1682818	-0.082587423	6.036895035	-3.654713941	0.000616822	0.02306461	-0.544099624
ILMN_1658110	0.082474356	7.626379394	3.824949013	0.000363662	0.01703351	-0.054080207
ILMN_2081335	0.082468277	9.440519939	4.129927583	0.000137516	0.009241749	0.852542179
ILMN_2364131	-0.082420442	8.838954643	-4.314961953	7.51252E-05	0.006438484	1.418686608
ILMN_2189406	0.082412999	6.306606026	3.473026455	0.001070482	0.031230055	-1.053026073
ILMN_1813275	-0.082195498	10.06033962	-3.286104266	0.001860075	0.044502403	-1.560215788
ILMN_1767747	0.082072801	11.16732194	4.061661035	0.000171423	0.010784612	0.646609103
ILMN_2224143	0.082044854	11.33140974	3.600033166	0.000729173	0.02518934	-0.69884554
ILMN_1719010	-0.081923695	6.77102049	-3.684834702	0.000562226	0.021828028	-0.45829559
ILMN_1666078	0.081842125	7.427112998	4.158250298	0.000125446	0.008701955	0.93845886
ILMN_1750088	0.081809059	8.257719251	3.484046872	0.001035682	0.03078118	-1.022591698
ILMN_1728975	0.081757633	9.406541507	4.804829197	1.44968E-05	0.002349659	2.966444723
ILMN_2407799	0.081752504	6.374703257	4.356170924	6.55716E-05	0.005933454	1.546274361
ILMN_1667577	0.081696008	8.290849641	4.330350735	7.14087E-05	0.006248444	1.46627044
ILMN_1762529	0.081630602	7.058433989	3.359071905	0.00150197	0.038787956	-1.364290243
ILMN_1704842	0.081546983	6.070801416	3.226333716	0.00221216	0.049948438	-1.718670116
ILMN_1808047	0.081516946	6.950667834	3.48810268	0.001023148	0.030580526	-1.011376546
ILMN_1678037	0.081461881	7.767012798	3.527094493	0.000909804	0.028592897	-0.903161498
ILMN_1659343	0.081338253	10.95082878	4.247954276	9.36234E-05	0.007224229	1.212365394
ILMN_1663383	0.081330231	6.846656051	3.617404226	0.000691513	0.024552645	-0.649829878
ILMN_3207933	0.081305585	9.839309498	3.318100357	0.001694089	0.041747966	-1.474635234

ILMN_2043306	-0.081293709	5.970853322	-3.434372839	0.001201556	0.033496112	-1.159314848
ILMN_1745593	0.081288177	7.464089698	3.724302883	0.000497667	0.020244413	-0.345270212
ILMN_1660844	0.08126418	8.236984184	4.224095075	0.000101224	0.007390097	1.139251391
ILMN_1805863	-0.081167071	7.372344362	-3.440220028	0.001180792	0.03323025	-1.143282474
ILMN_2054362	0.081030312	7.511284142	4.05136318	0.000177194	0.011007117	0.615688698
ILMN_1784328	0.080956568	6.665213299	3.694720538	0.000545339	0.021487249	-0.430048257
ILMN_1680196	0.080946933	10.993156	3.706282072	0.000526208	0.020981412	-0.396959445
ILMN_1815306	-0.08091138	7.293170646	-3.535150635	0.000887929	0.02811042	-0.880714619
ILMN_3246391	0.080854173	7.146862906	3.265376607	0.001975695	0.046398779	-1.615375975
ILMN_1749709	-0.080828156	10.32534891	-3.300991238	0.001781017	0.043301717	-1.520462376
ILMN_1690252	0.080797463	7.900762356	3.404312026	0.001313904	0.035472325	-1.24147656
ILMN_1787511	0.080773747	7.67059321	4.312161052	7.58216E-05	0.006438484	1.410033876
ILMN_1739199	-0.080746408	7.359268692	-3.608009398	0.000711643	0.024854261	-0.676355919
ILMN_1682449	-0.080700334	8.461127668	-3.74359928	0.000468755	0.019597123	-0.28976919
ILMN_2179726	0.080691536	6.727532067	4.682542113	2.19786E-05	0.002875057	2.574133207
ILMN_2411559	0.080570292	7.212475437	4.535305179	3.61086E-05	0.00391472	2.106778724
ILMN_2381603	0.080472477	6.527529949	4.326199317	7.23933E-05	0.006307263	1.453426517
ILMN_1675186	0.080395238	7.954592689	3.74150702	0.00047181	0.019645076	-0.295794634
ILMN_1659564	-0.080360013	9.7825274	-3.78092081	0.000417349	0.018376139	-0.18197979
ILMN_1670948	-0.080339147	8.812807803	-3.831792917	0.000355938	0.016810019	-0.034128058
ILMN_1769751	-0.080255768	7.054066529	-4.003634303	0.000206494	0.012277821	0.472882031
ILMN_1791508	-0.080255236	6.284752762	-3.714530057	0.000512955	0.020708017	-0.37331889
ILMN_1680386	0.080168706	6.946893529	4.805961899	1.44408E-05	0.002349659	2.97009515
ILMN_1696133	0.080143027	6.850995571	3.267318665	0.00196458	0.04627043	-1.610217222
ILMN_2221014	0.080078787	6.324598604	3.71165017	0.000517546	0.020844522	-0.381576593
ILMN_3248701	0.080073891	5.966318015	3.557982061	0.000828629	0.026900028	-0.81693637
ILMN_1802157	0.080044194	8.260439835	5.000485312	7.40101E-06	0.001514546	3.601193035
ILMN_2313821	0.080042996	8.017745795	5.24209744	3.19536E-06	0.000934754	4.395509657
ILMN_1772894	0.080041765	6.317471789	3.677669301	0.000574777	0.022072726	-0.478743241
ILMN_3239130	-0.079994887	6.887690391	-3.989142757	0.000216282	0.012681172	0.429689018
ILMN_1730523	0.079993571	7.930678436	3.322259282	0.001673574	0.041479641	-1.463472982
ILMN_3246353	0.079957349	7.169918263	3.480361033	0.001047199	0.03101653	-1.032777053
ILMN_3228700	0.079954994	6.753810593	3.868022844	0.000317611	0.015892516	0.071807358
ILMN_1772703	0.079912572	7.706179104	3.583369031	0.000767138	0.025766729	-0.745738916
ILMN_1664030	0.079804849	7.490503007	3.432206943	0.001209336	0.033515316	-1.16524931
ILMN_1710756	0.07978289	13.05099861	3.496795347	0.00099677	0.030259518	-0.987313355
ILMN_3295874	-0.079700619	6.768968205	-3.283234735	0.001875691	0.044659066	-1.56786536
ILMN_1753613	-0.079675303	7.234789364	-3.389108357	0.00137446	0.036444246	-1.282862826
ILMN_3248263	-0.079643072	7.57376143	-3.89702745	0.000289825	0.015116513	0.156992039
ILMN_1669598	0.079614454	7.075845759	4.670027137	2.29306E-05	0.002960876	2.534189972
ILMN_1778238	0.079583496	9.702042835	4.27209377	8.64988E-05	0.006982696	1.286526928
ILMN_1734826	0.079510422	10.01109247	3.572527827	0.000792844	0.026270447	-0.776178872
ILMN_1674706	0.079505111	8.760990694	3.63642907	0.000652406	0.023868572	-0.595993251
ILMN_1708513	-0.079481667	5.683599156	-3.815103284	0.000375057	0.017295556	-0.082750314
ILMN_1793476	-0.079474787	8.059651438	-3.547264644	0.000855979	0.027502998	-0.846904747
ILMN_1793371	-0.079473564	8.352824034	-3.580829517	0.000773087	0.025830261	-0.752874157
ILMN_2350801	-0.079374804	6.091863246	-4.2328743	9.83603E-05	0.007309774	1.166132692
ILMN_2395236	0.079362261	7.819575602	3.452315992	0.001138917	0.032531321	-1.110064325
ILMN_1782954	0.079319562	9.122717301	3.395489146	0.001348729	0.036017653	-1.26550736
ILMN_2391512	0.079107315	6.565383725	3.268316089	0.001958895	0.046193477	-1.607566971
ILMN_1737416	-0.079064985	7.319151182	-3.263103706	0.001988779	0.04664296	-1.621411074
ILMN_2405009	-0.079034251	9.694206071	-3.366079523	0.001471254	0.038281484	-1.345332976
ILMN_1750130	0.079019059	11.23969926	4.002688978	0.000207119	0.012290255	0.470062048
ILMN_1690282	0.079003338	7.509177298	4.086933116	0.00015802	0.010161946	0.722652565

ILMN_1796968	0.078969099	7.273116102	3.67885387	0.000572684	0.022062985	-0.475364416
ILMN_1654518	-0.078707086	6.571464652	-3.361081296	0.001493101	0.038607137	-1.358856865
ILMN_1745110	-0.078672361	9.549561246	-3.853439782	0.000332534	0.016238578	0.02910368
ILMN_3212373	0.078631255	9.858827987	3.53554663	0.000886867	0.02811042	-0.87961048
ILMN_1690138	0.078628357	7.195187762	4.47766294	4.37912E-05	0.004466704	1.925407513
ILMN_2347298	-0.078580952	6.475009641	-3.373985322	0.001437318	0.037625635	-1.323916589
ILMN_1893633	0.078527366	6.969511068	3.474988466	0.001064206	0.03116883	-1.047611931
ILMN_1662905	0.078507361	12.26726838	3.749181064	0.000460697	0.019440632	-0.273685343
ILMN_2083243	0.078502149	8.48723459	4.251652748	9.24959E-05	0.007220796	1.223715623
ILMN_2246894	-0.078488641	10.1219053	-3.690361576	0.000552724	0.021556245	-0.442508555
ILMN_2414007	0.078437045	6.757321203	4.019993001	0.00019596	0.011957347	0.521733771
ILMN_1755758	0.078411645	6.84684349	3.333350717	0.001620006	0.040673606	-1.433661576
ILMN_1653822	0.078242257	6.814944733	4.028389585	0.000190756	0.011722844	0.546846641
ILMN_3306388	0.078231077	6.678256697	4.010053244	0.000202297	0.012129354	0.492039037
ILMN_1668657	0.078208555	6.905298391	4.59906024	2.91426E-05	0.003488612	2.30844726
ILMN_1721977	0.078101769	8.948922115	3.862790095	0.00032289	0.016040262	0.056474523
ILMN_1700232	0.078095523	8.155791901	3.928518997	0.000262315	0.014219428	0.249853271
ILMN_1653836	-0.078089211	6.280263901	-3.724120461	0.000497948	0.020244413	-0.345794146
ILMN_1842850	-0.078081729	6.399931117	-3.503993666	0.000975418	0.029812516	-0.967359911
ILMN_2221673	0.078024548	9.902959077	3.240026998	0.002126338	0.048535586	-1.682533116
ILMN_1795865	-0.077990646	7.208198689	-3.648192174	0.000629296	0.023404091	-0.562625713
ILMN_2406501	0.077905052	7.147168897	3.509940134	0.00095811	0.029485439	-0.950858199
ILMN_1811574	-0.077887257	8.301683555	-3.272886764	0.00193304	0.045677505	-1.595415671
ILMN_1755910	-0.077829811	6.575995019	-3.832659753	0.000354971	0.016810019	-0.031599605
ILMN_3255144	0.077718035	6.47117696	3.615380029	0.000695804	0.024558061	-0.655548479
ILMN_2286024	0.077713749	5.747320821	5.488709304	1.34331E-06	0.000564779	5.216213614
ILMN_1736176	0.077656769	7.014994795	3.853629949	0.000332335	0.016238578	0.029660002
ILMN_1798940	0.077607289	6.881157459	4.087774497	0.000157591	0.010155363	0.725188191
ILMN_3234884	0.077488033	8.666051852	3.37819099	0.001419568	0.037331073	-1.312511023
ILMN_1702384	-0.077393663	7.422851444	-3.284596349	0.001868266	0.044571665	-1.564236109
ILMN_2050617	0.077362511	10.63245265	3.231025828	0.002182391	0.04951251	-1.706298574
ILMN_1753265	-0.077333261	6.468277715	-3.647270094	0.000631078	0.023445107	-0.565243515
ILMN_2352934	0.077310439	6.5887728	3.631849704	0.000661621	0.023919797	-0.608966662
ILMN_2181992	-0.077286229	6.76912353	-3.39566305	0.001348034	0.036017653	-1.265034069
ILMN_1688848	-0.077165856	6.092465537	-3.321559491	0.001677009	0.041479641	-1.465351785
ILMN_1720965	-0.07713421	7.884281005	-3.289667319	0.001840857	0.044165193	-1.550711536
ILMN_1805148	-0.077031954	6.428120905	-4.53149468	3.6573E-05	0.003940281	2.094760422
ILMN_1813246	0.077021741	9.378718176	3.285259996	0.001864657	0.044550185	-1.562466874
ILMN_1798543	0.076997558	7.45845359	3.615543209	0.000695457	0.024558061	-0.655087544
ILMN_1723843	-0.076957245	8.337516591	-3.974324774	0.000226753	0.01305066	0.385604178
ILMN_1755620	-0.076878492	6.813173541	-3.410615364	0.001289549	0.035117296	-1.224284862
ILMN_1698015	-0.076828392	6.189212296	-4.169976026	0.000120755	0.008444549	0.974109275
ILMN_1655952	-0.076787833	9.166140473	-3.40273713	0.001320057	0.035548673	-1.245768889
ILMN_1749243	0.076611613	7.439673282	3.620290968	0.000685438	0.024488264	-0.641671321
ILMN_1653283	-0.076537693	6.972005154	-3.299229545	0.001790204	0.043403012	-1.525172633
ILMN_2162799	0.076526978	7.112451481	4.571097589	3.2019E-05	0.003707769	2.219861587
ILMN_1775823	-0.076403162	7.61742292	-3.295319552	0.001810755	0.043749924	-1.535621149
ILMN_1782633	0.076245244	10.44958047	3.779808701	0.000418799	0.018393013	-0.185200089
ILMN_1700378	0.075958014	7.794192441	3.401631011	0.001324395	0.03556843	-1.248782855
ILMN_1772743	0.075933398	8.542248212	3.466927038	0.001090219	0.031665027	-1.069845623
ILMN_1760933	0.075920704	7.729166437	3.706150672	0.000526422	0.020981412	-0.397335833
ILMN_1662417	0.075881508	8.315637427	4.021398827	0.000195079	0.011924716	0.525936573
ILMN_2389528	0.075779668	6.108071362	4.519325317	3.80954E-05	0.004028765	2.056405143
ILMN_1782377	0.075656596	8.157530126	4.111193984	0.000146112	0.009668604	0.795866757

ILMN_1686835	0.075618458	6.816222489	3.564634579	0.000812075	0.026563495	-0.79830789
ILMN_3247906	-0.075548023	8.076962899	-3.605195665	0.000717781	0.024926372	-0.684292723
ILMN_2411794	-0.075544527	8.508564921	-3.868326224	0.000317307	0.015892516	0.072696645
ILMN_3268403	-0.075534312	5.923421506	-3.824539936	0.000364129	0.01703351	-0.055272195
ILMN_3242462	0.075356502	6.881228331	3.796150935	0.000397968	0.017958559	-0.137827007
ILMN_3238452	-0.075161788	7.509117079	-4.236067062	9.73383E-05	0.007309774	1.17591496
ILMN_2397571	0.075104507	6.875507995	3.262732555	0.001990923	0.046661507	-1.622396317
ILMN_1672547	-0.075070434	7.394584235	-3.394094186	0.001354315	0.036070429	-1.2693033
ILMN_2410742	-0.074999109	8.216273899	-3.487088168	0.00102627	0.030580526	-1.014182612
ILMN_1758827	0.074966957	7.929980088	3.889706682	0.000296608	0.015308195	0.135460166
ILMN_1724333	-0.074953178	7.319626576	-4.256631339	9.09991E-05	0.007161366	1.239001423
ILMN_1678454	0.074936405	9.454743154	3.259581246	0.002009218	0.046877323	-1.630758758
ILMN_1729868	0.074827121	6.962719886	4.090434356	0.000156245	0.010087444	0.733205725
ILMN_1783337	0.074778737	6.847806658	3.672166234	0.000584599	0.022356075	-0.494432014
ILMN_2224990	0.074775767	6.33814044	4.000826942	0.000208356	0.012321234	0.464508405
ILMN_2191313	-0.074668205	6.159919208	-4.226755049	0.000100348	0.007349386	1.147393343
ILMN_1780292	0.074580965	6.065160059	4.514633693	3.86987E-05	0.004079352	2.0416291
ILMN_1771903	0.074546562	10.20930277	3.666718037	0.000594481	0.022547111	-0.509951369
ILMN_2377430	-0.074481234	7.648642656	-4.00431985	0.000206042	0.012277821	0.474927283
ILMN_1708029	-0.074426632	5.796381971	-3.735981547	0.00047997	0.019769968	-0.311698418
ILMN_1695962	-0.074365482	7.735067839	-3.875476195	0.000310235	0.01567043	0.093665688
ILMN_1797499	0.074312538	7.334307376	3.368553077	0.001460555	0.038118174	-1.338635588
ILMN_1662364	0.074304219	9.77174734	3.484838032	0.001033225	0.03075036	-1.020404588
ILMN_1721034	-0.07425843	6.996372494	-3.669741748	0.000588977	0.022452432	-0.501339833
ILMN_1691131	0.074257533	8.694917948	3.85419261	0.000331747	0.016238578	0.031306115
ILMN_1662161	-0.0741303	6.398687841	-3.79195855	0.000403215	0.018033631	-0.149990446
ILMN_3289262	0.074084771	9.609777337	3.998395003	0.000209982	0.012374946	0.457256933
ILMN_1778611	0.074008831	8.430149024	3.564996361	0.000811184	0.026563495	-0.797294243
ILMN_1765923	0.073932226	6.748244899	3.806041458	0.000385849	0.017642597	-0.109102869
ILMN_1849941	-0.073913635	6.628758361	-3.428694394	0.001222053	0.033786296	-1.17486872
ILMN_1790461	0.073741875	10.46785016	3.590286738	0.000751154	0.025581318	-0.726287508
ILMN_1710979	0.073672673	7.122279356	3.725240628	0.000496223	0.020221971	-0.342576681
ILMN_1810838	0.073652107	10.17687728	3.945014729	0.000248928	0.013643953	0.298648188
ILMN_1772706	-0.073579121	7.77093023	-3.261538631	0.001997836	0.046728219	-1.625565168
ILMN_1654861	-0.073450877	7.784018319	-3.299669829	0.001787904	0.043393782	-1.523995589
ILMN_1789627	-0.073367226	6.247496269	-3.6183371	0.000689545	0.024533273	-0.647193782
ILMN_1774990	0.073273731	8.209855864	3.349791178	0.001543587	0.039520098	-1.38935908
ILMN_1682996	0.073103161	6.48038797	3.50552416	0.000970935	0.029728203	-0.963114305
ILMN_1662184	0.073050845	6.902235724	3.419455755	0.001256114	0.034506589	-1.200140932
ILMN_1726512	-0.072974831	5.750645936	-3.633386546	0.000658515	0.023919797	-0.604613802
ILMN_1807074	0.072952446	12.41159786	3.988596549	0.000216659	0.012681742	0.42806254
ILMN_1697544	-0.072862688	6.549149501	-3.45162357	0.001141276	0.032558021	-1.111967765
ILMN_1781257	0.072847446	7.109016083	3.665912256	0.000595956	0.022553434	-0.512245559
ILMN_1814247	-0.072844848	8.133791503	-3.652352728	0.000621311	0.023182152	-0.550809175
ILMN_1758895	-0.072766367	5.959236993	-3.432924918	0.001206752	0.033515316	-1.163282341
ILMN_1707534	0.072741445	7.520500785	5.668298295	7.1122E-07	0.000372997	5.819038767
ILMN_1655654	0.072525469	7.681589028	3.542391223	0.000868698	0.027756468	-0.860514479
ILMN_1727479	-0.072480954	9.198403553	-3.546176507	0.000858803	0.027517453	-0.849944474
ILMN_1667222	0.07246303	7.845547765	3.247599066	0.00208024	0.047878028	-1.662507998
ILMN_1765644	0.072453772	9.649809853	4.001319067	0.000208028	0.012321234	0.465976074
ILMN_1783695	0.072384702	10.06994638	4.106225315	0.000148477	0.009806326	0.780855467
ILMN_1777483	0.072324863	6.793191658	4.639710407	2.54071E-05	0.003118034	2.437594657
ILMN_1676233	0.072314795	6.565619719	4.12667674	0.000138972	0.009321395	0.842698517
ILMN_1794781	-0.072232542	6.90897944	-4.004085549	0.000206196	0.012277821	0.474228253

ILMN_1677440	-0.072219376	10.45174419	-3.538976204	0.000877718	0.027941103	-0.870044881
ILMN_1660579	0.072201843	7.033726994	3.393950765	0.001354891	0.036070429	-1.26969352
ILMN_1769634	0.072184337	9.424811436	3.463424422	0.001101709	0.031813375	-1.079496307
ILMN_1720442	-0.072133105	9.711776661	-3.73340339	0.000483824	0.019881202	-0.319114625
ILMN_1787199	0.072110734	5.756090017	3.384849936	0.00139189	0.036743347	-1.294434385
ILMN_1734553	-0.072074825	6.505855862	-4.4847091	4.27726E-05	0.004415059	1.947528105
ILMN_1666206	-0.072068082	6.701845349	-3.388389198	0.001377389	0.036444246	-1.28481765
ILMN_2375557	0.071971528	6.017795188	3.65238927	0.000621241	0.023182152	-0.550705356
ILMN_2061452	0.071960666	8.406672671	3.836439813	0.000350784	0.016680856	-0.020570094
ILMN_1708345	0.071837161	6.267427086	3.283497913	0.001874254	0.044655683	-1.567163959
ILMN_1815479	0.071831885	11.76915255	3.621769806	0.000682346	0.024479253	-0.637490366
ILMN_1694799	0.071806996	7.960521392	3.278043653	0.001904256	0.045121052	-1.581692936
ILMN_1761262	0.07173785	6.502507771	3.477030716	0.001057711	0.031087493	-1.041974431
ILMN_1740319	-0.071728894	8.118392901	-3.265725136	0.001973696	0.046395634	-1.61445031
ILMN_1741148	0.071569816	11.24149657	4.23146548	9.88145E-05	0.007316553	1.161817289
ILMN_2361807	-0.071563319	6.954242049	-3.442498004	0.001172796	0.033168616	-1.137032039
ILMN_1711543	0.071484988	7.714510099	3.510305956	0.000957054	0.029485439	-0.949842486
ILMN_1709623	-0.071345022	6.769464895	-3.909046409	0.000279012	0.014730813	0.192387687
ILMN_1793146	0.071343647	7.836729142	3.389288419	0.001373727	0.036444246	-1.282373342
ILMN_1804479	0.071343236	10.40331021	4.210683215	0.000105757	0.007598501	1.098234459
ILMN_1680659	-0.071326691	6.070393958	-3.333644904	0.001618608	0.040672832	-1.432870017
ILMN_1783973	-0.071256747	6.213538021	-3.803447998	0.000388992	0.017715823	-0.116638692
ILMN_1746252	0.07118992	10.81670588	3.477605573	0.001055889	0.03106853	-1.040387217
ILMN_1654690	0.071123418	8.92939	3.364143146	0.001479682	0.038388161	-1.35057378
ILMN_1727761	0.071070073	6.848967664	3.750506299	0.000458804	0.019384459	-0.269864769
ILMN_1748831	-0.071040491	7.036402371	-3.694434297	0.000545821	0.021487249	-0.43086674
ILMN_1793966	0.070915432	6.39172861	3.796669249	0.000397324	0.017952992	-0.136322711
ILMN_1716400	0.070902639	6.972341492	3.388637789	0.001376376	0.036444246	-1.284141956
ILMN_1706839	0.070830869	8.051775505	3.690383631	0.000552687	0.021556245	-0.442445533
ILMN_1651936	-0.070801328	8.362008434	-3.524490777	0.000916983	0.028760166	-0.910409808
ILMN_2278819	0.070791707	6.918490101	3.577363785	0.000781277	0.025949231	-0.762607057
ILMN_2130838	0.07076992	9.563751763	3.286952737	0.001855482	0.044454198	-1.55795313
ILMN_1698846	-0.070767851	5.446632417	-3.276621394	0.001912154	0.045277069	-1.585479007
ILMN_1739283	0.070708168	8.105886125	3.570972946	0.000796597	0.02631408	-0.780540286
ILMN_1769849	0.07059188	5.956986653	3.966139938	0.000232746	0.013175935	0.361288948
ILMN_1808163	0.070583135	6.654040639	4.237847404	9.67729E-05	0.007300534	1.181371182
ILMN_2194627	0.070510605	7.63031781	3.451487202	0.001141741	0.032558021	-1.112342606
ILMN_1760714	0.070498924	13.40671696	3.933564909	0.000258148	0.014037694	0.264768157
ILMN_1756220	0.070271588	9.88412041	3.919345629	0.000270057	0.014438959	0.222763447
ILMN_1696503	0.070165836	5.995005351	3.341054698	0.00158376	0.040177859	-1.412918393
ILMN_3238797	0.070107528	6.616323015	3.988045626	0.000217041	0.012682538	0.426422134
ILMN_1700628	0.070022878	8.914394282	3.49683435	0.000996653	0.030259518	-0.987205306
ILMN_3237241	-0.069896106	8.244826704	-3.678524915	0.000573264	0.022062985	-0.476302778
ILMN_3235477	0.069853259	7.238990491	3.310182107	0.001733808	0.042423546	-1.495862964
ILMN_1766010	0.06978552	9.713605628	3.40605828	0.001307113	0.035399878	-1.23671578
ILMN_1761722	-0.069647612	6.721795399	-3.436334504	0.001194552	0.033496112	-1.153938011
ILMN_1737205	0.069608684	10.47445695	3.328386508	0.001643777	0.040976769	-1.447011996
ILMN_2396947	0.069554487	6.802563637	3.312091545	0.00172415	0.042307335	-1.490746942
ILMN_2095133	-0.069505762	8.352404301	-4.013459089	0.000200103	0.012039718	0.502209796
ILMN_1769503	0.069489701	5.950627897	4.165079332	0.000122693	0.008545386	0.959215871
ILMN_1755710	-0.069453797	6.443959721	-3.580044535	0.000774935	0.025860693	-0.755079117
ILMN_3276859	0.069359623	6.856226775	3.361890367	0.001489544	0.038553688	-1.356668577
ILMN_1734895	0.069346762	10.45557333	3.969443944	0.000230309	0.013127837	0.371101334
ILMN_1713732	-0.069309661	7.408824728	-3.562849718	0.000816486	0.026679717	-0.803307871

ILMN_1774427	-0.069030164	6.664536029	-3.311073532	0.001729293	0.042373212	-1.493474771
ILMN_2400583	-0.06897792	6.221053913	-3.690939625	0.000551739	0.021556245	-0.440856647
ILMN_1722798	-0.068965038	6.920933643	-3.579439883	0.000776361	0.025860693	-0.756777355
ILMN_3240365	-0.068959015	6.127373729	-3.488360454	0.001022356	0.030580526	-1.010663484
ILMN_1662232	-0.068907479	10.57957775	-3.615225853	0.000696132	0.024558061	-0.655983969
ILMN_2414366	0.068640082	6.528360207	4.147882288	0.00012974	0.008927988	0.906975552
ILMN_1740197	-0.06863397	6.462390215	-3.853504321	0.000332466	0.016238578	0.029292481
ILMN_1700549	0.068578717	6.885616731	3.923690946	0.000266362	0.01437401	0.235591529
ILMN_1757956	-0.068471819	7.737848	-3.226311745	0.0022123	0.049948438	-1.718728019
ILMN_1677953	0.068393927	8.51731532	3.228721753	0.002196961	0.049732396	-1.712375103
ILMN_1733511	-0.068356624	9.799019618	-3.582308757	0.000769616	0.025797973	-0.74871831
ILMN_3251567	-0.068297069	6.775703698	-3.517460695	0.000936638	0.02913385	-0.929964613
ILMN_1660186	-0.068272689	8.751922223	-3.443864718	0.001168023	0.033143858	-1.133280775
ILMN_1686668	0.068207863	7.342005763	4.232814258	9.83796E-05	0.007309774	1.165948762
ILMN_1706571	-0.068105933	6.719258621	-3.351798057	0.001534496	0.039391865	-1.383941806
ILMN_1693559	-0.068050617	6.126789969	-3.736973464	0.000478496	0.019732794	-0.308844364
ILMN_3249963	0.068031667	6.239203324	3.490495537	0.001015821	0.030533085	-1.004756154
ILMN_1724009	0.068013831	7.799938942	3.790907489	0.00040454	0.018065982	-0.153038775
ILMN_1755235	0.068012934	8.331260537	3.899223111	0.00028782	0.015034677	0.163454015
ILMN_1746368	-0.0679695	7.297648285	-4.05502549	0.00017512	0.010897928	0.626680787
ILMN_1752027	-0.067874745	7.845224331	-3.325508197	0.001657711	0.04123467	-1.454747034
ILMN_1748481	0.067856719	7.970755997	3.743123012	0.000469449	0.019597123	-0.291140944
ILMN_2415949	-0.067798209	8.011595316	-3.561368357	0.000820163	0.026725846	-0.807456545
ILMN_2182198	0.067750644	9.117460601	3.960581575	0.000236902	0.013280394	0.344790788
ILMN_1678546	-0.067721067	9.135787099	-3.782079514	0.000415843	0.018351643	-0.178624024
ILMN_1805992	-0.067676799	9.205907086	-3.905734708	0.000281952	0.014801647	0.182629156
ILMN_1694486	0.067608095	6.609585853	3.911815908	0.000276576	0.014692204	0.200551797
ILMN_1651504	-0.067584794	7.020902607	-3.516760564	0.000938618	0.02914041	-0.931910829
ILMN_1722953	-0.06749813	6.095271562	-3.725857432	0.000495276	0.020207247	-0.340804802
ILMN_1770978	0.067482312	5.619437108	3.227474112	0.00220489	0.049866774	-1.715664334
ILMN_1880012	-0.067342753	6.180205205	-4.02191591	0.000194756	0.011924716	0.527482605
ILMN_2387599	-0.067248337	11.77283695	-3.280369855	0.001891405	0.044909149	-1.575498309
ILMN_2285112	-0.067240464	9.45943972	-3.765715201	0.000437602	0.018882077	-0.225965726
ILMN_1702124	-0.06722401	8.138659758	-4.218169796	0.000103203	0.00745919	1.121123015
ILMN_1875342	-0.067215229	7.489628569	-4.262552794	8.92494E-05	0.007073475	1.257192559
ILMN_1669635	0.067171463	9.458682579	4.201345524	0.000109029	0.007798497	1.069712661
ILMN_1800958	0.066950527	9.531793648	3.975149631	0.000226158	0.01305066	0.388056035
ILMN_1787477	0.06685524	6.784700874	3.51917848	0.000931799	0.029019604	-0.925188564
ILMN_1736863	-0.066715781	5.676026796	-3.541808378	0.000870231	0.027779712	-0.862141424
ILMN_1670796	0.066714974	9.651497843	3.921821999	0.000267945	0.014388912	0.230073201
ILMN_2322935	0.066679489	8.922014958	3.851064358	0.000335028	0.016287503	0.022155745
ILMN_1742922	0.066632881	8.151503348	3.68716983	0.000558192	0.021720357	-0.45162711
ILMN_2298511	0.066630408	8.45723373	3.438548891	0.001186692	0.033343425	-1.14786624
ILMN_1678268	-0.066594283	7.226246459	-3.961628684	0.000236114	0.01326011	0.34789789
ILMN_3241985	0.066558068	6.365158977	3.784659162	0.000412509	0.01828095	-0.171151013
ILMN_1665065	-0.066532619	6.807666396	-3.770268318	0.000431441	0.018733435	-0.212804802
ILMN_1809086	-0.066521775	7.998576443	-3.794330345	0.000400238	0.01801027	-0.143110005
ILMN_1767541	0.066477693	6.062373741	3.810670511	0.000380299	0.017435104	-0.095645378
ILMN_1750178	-0.066442806	7.241326729	-3.677582626	0.000574931	0.022072726	-0.478990446
ILMN_1773148	-0.06629515	6.855625053	-3.357307527	0.001509799	0.038902714	-1.369059445
ILMN_2402806	-0.066263692	8.329239167	-3.247584924	0.002080325	0.047878028	-1.662545426
ILMN_1687546	-0.066263565	6.590848609	-3.331229932	0.001630122	0.040776416	-1.439366599
ILMN_2407308	-0.066200194	6.087804109	-3.713240551	0.000515006	0.020766484	-0.37701682
ILMN_1713875	0.066194151	6.984570751	3.314247286	0.001713308	0.042131238	-1.484968764

ILMN_1674128	0.066168538	7.295817123	4.083939144	0.000159553	0.01020546	0.713631825
ILMN_1789732	-0.066119036	7.190356831	-3.802748021	0.000389844	0.017731226	-0.118672146
ILMN_1770732	0.066018617	10.41735191	3.25916731	0.002011633	0.046877323	-1.631856813
ILMN_1738819	0.06592204	9.110634952	3.469998001	0.001080239	0.031454657	-1.061379452
ILMN_3248575	0.065907533	6.366202355	4.380920876	6.04143E-05	0.005614129	1.623153805
ILMN_1745954	0.065765503	9.440604124	3.529841457	0.000902288	0.02840848	-0.895510997
ILMN_3188099	0.065713751	7.349979962	3.863032869	0.000322643	0.016040262	0.05718565
ILMN_2046024	0.06568304	7.334315061	3.916186598	0.000272774	0.014527246	0.213442061
ILMN_1744914	0.065604741	7.926975297	3.690573914	0.000552362	0.021556245	-0.441901767
ILMN_1795893	-0.065568175	7.453638816	-4.146327254	0.000130396	0.008929994	0.902256748
ILMN_1736340	-0.065567117	8.446268738	-4.086178945	0.000158404	0.010161946	0.720379973
ILMN_1659156	0.065535999	6.381475056	4.229499092	9.9452E-05	0.007326296	1.155795068
ILMN_1665554	-0.065472814	6.731716216	-3.487305581	0.0010256	0.030580526	-1.013581306
ILMN_2163790	-0.065462639	5.714307291	-3.308135315	0.001744218	0.042537113	-1.501344943
ILMN_2069821	0.065418714	6.303885667	3.882020412	0.000303895	0.015452932	0.112875929
ILMN_1779184	0.06541314	6.575723137	4.069329569	0.000167243	0.010579602	0.669659496
ILMN_1700896	0.065363639	8.699258885	3.769368929	0.000432651	0.018762366	-0.215405198
ILMN_1740165	-0.065241613	7.584426065	-3.379095377	0.001415779	0.037259839	-1.310057226
ILMN_1691333	-0.065194564	6.389421929	-3.631523613	0.000662282	0.023919797	-0.609890129
ILMN_1663493	0.064712462	7.638220506	3.294173259	0.001816822	0.043801916	-1.538682856
ILMN_1698365	0.06451328	5.905556162	3.507772725	0.000964384	0.029553873	-0.956874774
ILMN_1810474	0.064498365	10.47726291	3.70731952	0.000524523	0.020981412	-0.393987489
ILMN_2369682	0.064425606	12.64902512	3.854843127	0.000331069	0.016238578	0.033209415
ILMN_2231020	0.064347605	8.699812758	3.269323656	0.001953167	0.04608994	-1.604889248
ILMN_2139827	0.064278633	6.494419722	3.320888096	0.001680311	0.041479641	-1.467154115
ILMN_1666332	-0.064255908	6.906979958	-3.337242455	0.001601599	0.040464478	-1.423186697
ILMN_2319544	0.064242177	6.139728567	3.463722129	0.001100728	0.031813375	-1.078676268
ILMN_1654598	-0.064232514	6.784461684	-3.730847059	0.000487675	0.019992792	-0.326465271
ILMN_1747162	0.064148987	9.333038767	3.329654374	0.001637675	0.040883768	-1.443603461
ILMN_1665601	0.064001475	7.153769478	3.683017062	0.000565384	0.02190134	-0.463484633
ILMN_1774281	0.063911006	6.20984184	3.80438245	0.000387856	0.01771091	-0.113923771
ILMN_1670377	-0.063752102	6.558710872	-3.344276641	0.00156883	0.039975606	-1.404234367
ILMN_1791896	0.063689695	9.98260125	3.420645339	0.001251678	0.034412155	-1.196889162
ILMN_1714461	-0.063686207	8.581908686	-3.588551506	0.000755133	0.025653751	-0.731168718
ILMN_1764398	0.063514873	6.952880119	4.115433504	0.000144122	0.00959218	0.808681997
ILMN_1695917	-0.063416836	9.133860271	-3.565796128	0.000809218	0.026563495	-0.795053228
ILMN_1785177	0.063390611	7.109600281	3.29255409	0.001825424	0.04391718	-1.543006454
ILMN_1791302	0.063347114	5.413727231	4.132604445	0.000136329	0.009197809	0.860650562
ILMN_1689720	0.063266856	6.837395461	3.40243568	0.001321238	0.035548673	-1.246590344
ILMN_1658830	-0.063236906	7.297197159	-3.349218154	0.001546192	0.039544884	-1.390905504
ILMN_1787308	-0.063199182	7.499074379	-3.356960792	0.001511342	0.038913391	-1.369996502
ILMN_1741564	-0.063195444	8.096878492	-3.543315508	0.000866272	0.027704626	-0.857934121
ILMN_1709162	0.063189432	6.238882198	3.590109476	0.000751559	0.025581318	-0.726786207
ILMN_1699082	0.062966325	7.642711274	3.96195883	0.000235866	0.01326011	0.348877624
ILMN_3188196	0.062908124	5.65451028	3.255247814	0.002034635	0.04726824	-1.642249702
ILMN_1679731	0.062822108	7.707593445	3.823293767	0.000365554	0.017053911	-0.058902927
ILMN_2090786	0.062745705	6.199522487	3.548545395	0.000852666	0.027471698	-0.843326248
ILMN_3262936	0.062672977	7.718662859	3.832143215	0.000355547	0.016810019	-0.033106318
ILMN_1670532	0.062658366	7.726652012	3.513801245	0.000947028	0.029308566	-0.940134581
ILMN_1776347	0.06262765	11.11012971	3.591045522	0.00074942	0.025581185	-0.724152619
ILMN_1673944	-0.062125394	10.05074973	-3.632691362	0.000659918	0.023919797	-0.606582929
ILMN_1781099	-0.062107173	8.614731154	-3.465969994	0.001093347	0.031729152	-1.072483129
ILMN_1670440	0.061995471	5.63845625	3.609196551	0.000709069	0.024854261	-0.673006204
ILMN_1705266	0.061939396	7.110534272	3.63646631	0.000652332	0.023868572	-0.595887713

ILMN_1750511	0.061781182	7.313128224	3.434557949	0.001200894	0.033496112	-1.158807548
ILMN_1667260	0.061701641	8.447506948	3.299543281	0.001788565	0.043393782	-1.524333908
ILMN_1667580	0.06165061	6.044111338	3.469596421	0.001081539	0.031465937	-1.0624868
ILMN_1787541	-0.061639776	6.610778665	-3.230573133	0.002185246	0.049532246	-1.707492686
ILMN_1814589	0.061504323	7.199962661	3.403528264	0.001316963	0.035527081	-1.243612829
ILMN_2230672	0.061463747	10.8150929	4.241310577	9.56823E-05	0.007251799	1.191987756
ILMN_2075892	0.061406469	5.774835782	3.616827896	0.000692733	0.024558061	-0.651458268
ILMN_1817545	-0.061144837	6.080008482	-3.374556944	0.001434893	0.037590703	-1.322366891
ILMN_1730660	-0.061056162	6.511666053	-3.606439452	0.000715062	0.024876348	-0.680784764
ILMN_1830985	-0.06097738	5.98512989	-3.266162674	0.001971189	0.046388207	-1.613288156
ILMN_1700238	-0.060956778	6.866116623	-3.295262093	0.001811058	0.043749924	-1.535774636
ILMN_2372698	-0.060934223	6.02320511	-3.64362513	0.000638174	0.023581644	-0.575587965
ILMN_3248057	-0.060932438	6.076666863	-4.695110324	2.10617E-05	0.002858752	2.614285839
ILMN_1744147	0.060919673	10.12961878	3.558384003	0.00082762	0.026892586	-0.815811422
ILMN_1739164	0.060837479	5.841738987	3.64080765	0.00064371	0.023684694	-0.58357999
ILMN_2357382	0.060777858	6.740874752	3.538402406	0.000879243	0.027963809	-0.87164567
ILMN_2038772	-0.060378485	11.82934813	-3.53709808	0.000882717	0.028022611	-0.875283925
ILMN_1813641	-0.060280979	7.114734822	-3.261886048	0.001995822	0.046712808	-1.624643148
ILMN_1674658	0.060159527	5.345731816	3.402269943	0.001321888	0.035548673	-1.247041958
ILMN_2354649	0.060028274	7.613953219	3.801667789	0.000391163	0.017767168	-0.12180986
ILMN_1675542	0.059909657	9.312797444	3.514212191	0.000945856	0.029298589	-0.938992832
ILMN_1656378	0.059787176	7.057486148	3.347820309	0.001552564	0.039678426	-1.394677186
ILMN_1750092	-0.05969603	6.273360073	-3.418005876	0.00126154	0.034601695	-1.204103297
ILMN_3242638	0.059596482	6.533511503	3.66924947	0.00058987	0.022452432	-0.502742115
ILMN_3234735	0.059402138	6.842951011	4.158921099	0.000125173	0.008700521	0.940497071
ILMN_1674908	-0.059085041	6.390540277	-3.657210725	0.00061211	0.022963126	-0.537002177
ILMN_1786426	0.059070702	6.398607261	3.572365824	0.000793234	0.026270447	-0.77663334
ILMN_1756352	-0.059023421	8.736712577	-3.614981275	0.000696652	0.024558061	-0.656674792
ILMN_1668408	0.059008	6.832078705	3.435293104	0.001198266	0.033496112	-1.156792672
ILMN_1747629	0.05898299	5.737485163	3.383938832	0.001395647	0.036814323	-1.296908997
ILMN_1837298	0.058946035	6.160308233	3.534182947	0.00089053	0.028141074	-0.883412483
ILMN_2377733	0.058870054	6.00763885	3.362008578	0.001489025	0.038553688	-1.356348824
ILMN_3239162	-0.058835341	5.864807066	-3.324694519	0.00166167	0.041273595	-1.456932916
ILMN_1688753	0.058734302	10.48570961	3.456392411	0.001125126	0.032201321	-1.098853756
ILMN_1737144	0.058718438	5.966923819	3.982370424	0.000221008	0.012892524	0.409530498
ILMN_1785107	0.058678442	7.490235155	3.523131468	0.000920753	0.028779579	-0.914192641
ILMN_3247222	0.058448773	5.894238494	3.441937347	0.001174759	0.033170292	-1.138570632
ILMN_1776147	0.058319174	8.432999532	3.717791165	0.000507804	0.020572336	-0.363963813
ILMN_1721337	0.058211788	7.576043369	3.784638614	0.000412536	0.01828095	-0.171210548
ILMN_1742962	0.058204573	6.073156708	3.405196753	0.00131046	0.035434825	-1.239064731
ILMN_1899940	0.058003612	6.250458606	3.591477152	0.000748435	0.025572898	-0.722938085
ILMN_1759789	0.057898448	6.664458115	3.341057295	0.001583748	0.040177859	-1.412911396
ILMN_1761474	0.057796317	7.498559688	3.582149905	0.000769988	0.025797973	-0.749164644
ILMN_1811301	-0.057679918	7.229325132	-3.405414172	0.001309614	0.035434825	-1.238471972
ILMN_2255310	0.05761203	11.02611257	3.819958839	0.000369395	0.017174075	-0.068616183
ILMN_3246944	0.057554365	6.035575315	3.66505768	0.000597524	0.022587987	-0.51467837
ILMN_3248562	0.057426328	6.903548659	3.459268052	0.001115493	0.032048122	-1.090940659
ILMN_1836218	0.057406946	7.240124504	3.357336264	0.001509671	0.038902714	-1.368981779
ILMN_1743131	-0.057329413	7.581141544	-3.68259217	0.000566125	0.021905426	-0.464697418
ILMN_1746893	0.057003747	6.199842473	3.30516784	0.001759415	0.042807059	-1.50928902
ILMN_3309754	-0.056942306	5.824769429	-3.293257695	0.001821681	0.043874554	-1.541127812
ILMN_1680403	-0.056941972	10.48190051	-3.235720315	0.002152985	0.049059024	-1.693909195
ILMN_1739876	-0.056941353	9.891474369	-3.599453616	0.000730462	0.025207406	-0.700478513
ILMN_1761721	0.056727118	10.41999883	3.290281914	0.001837561	0.044147562	-1.54907148

ILMN_1737805	0.056590976	5.529743714	3.25096648	0.002060044	0.047633858	-1.653592903
ILMN_1676159	0.056458594	9.376409984	3.465393168	0.001095237	0.031757254	-1.074072583
ILMN_2105573	0.056318651	6.023865854	3.548288987	0.000853328	0.027471698	-0.844042729
ILMN_1748607	0.05598653	5.781067065	3.91042576	0.000277796	0.014711683	0.196453455
ILMN_1693843	0.055789452	5.993364047	3.760444012	0.000444842	0.019051376	-0.241191465
ILMN_1724504	0.05578274	9.781236948	3.566191315	0.000808247	0.026563495	-0.793945774
ILMN_3309869	0.054710282	5.800399428	3.860618604	0.000325105	0.016125186	0.050114887
ILMN_1771815	-0.054546176	10.19335595	-3.321161647	0.001678965	0.041479641	-1.466419808
ILMN_2395092	0.054458201	5.9530958	3.570663008	0.000797348	0.02631408	-0.781409526
ILMN_1654365	0.054373604	5.770406688	3.668802044	0.000590682	0.022452432	-0.504016538
ILMN_2247296	-0.054335486	5.921463754	-4.244450319	9.47038E-05	0.007244749	1.201616217
ILMN_1873261	-0.054329967	5.929969504	-3.307952726	0.00174515	0.042537113	-1.501833872
ILMN_1701940	-0.054323418	7.001941146	-3.265145516	0.001977022	0.046398779	-1.615989701
ILMN_1703949	0.054318789	11.56885388	3.254818054	0.002037172	0.047295313	-1.643388764
ILMN_1810608	0.054159353	6.838954293	3.437755944	0.001189501	0.033395135	-1.150040744
ILMN_1801905	-0.054158522	6.675453208	-3.25843916	0.002015887	0.046927571	-1.633788172
ILMN_1655052	0.054123621	6.102045191	3.365268163	0.00147478	0.038315384	-1.347529148
ILMN_3298462	0.05389442	5.557262389	4.237946235	9.67416E-05	0.007300534	1.1816741
ILMN_2173740	-0.053868762	7.362773253	-3.241104955	0.002119717	0.048484857	-1.679684178
ILMN_1738497	-0.053804066	5.905921033	-3.441571253	0.001176043	0.033170292	-1.139575211
ILMN_1765532	0.053797577	7.495264903	3.326105259	0.001654812	0.041192266	-1.453142861
ILMN_1749212	-0.05378794	7.075684735	-3.423465047	0.001241223	0.034179237	-1.189178643
ILMN_1806122	-0.053686862	8.218411426	-3.261973484	0.001995316	0.046712808	-1.624411089
ILMN_1738656	0.053566903	9.319114388	4.344388293	6.81752E-05	0.006106451	1.509740343
ILMN_3310401	-0.053483613	5.717793192	-3.477800991	0.00105527	0.03106853	-1.039847619
ILMN_2308689	0.053455792	7.681956463	3.341467032	0.001581842	0.040177859	-1.411807331
ILMN_2049642	0.053413419	10.44110211	3.793976459	0.000400681	0.01801027	-0.144136755
ILMN_1680682	0.053382657	6.391217677	3.503378692	0.000977225	0.02981488	-0.969065542
ILMN_3240117	-0.053152688	10.03712504	-3.87387671	0.000311804	0.015706645	0.088973048
ILMN_1719857	-0.053091607	5.749521468	-3.248318664	0.002075908	0.047840254	-1.660603388
ILMN_1739454	0.052761802	7.317376461	3.938978126	0.000253749	0.013842142	0.280779613
ILMN_1755911	0.05243764	6.392163217	4.003544135	0.000206553	0.012277821	0.47261304
ILMN_2221336	0.051708064	6.184475327	3.607915249	0.000711848	0.024854261	-0.676621545
ILMN_2187746	0.051644004	6.165204253	3.36805953	0.001462684	0.038144858	-1.339972158
ILMN_2044645	-0.051587307	6.215111729	-3.379131603	0.001415627	0.037259839	-1.309958928
ILMN_1663024	0.051512303	5.277934841	4.388275129	5.89595E-05	0.005508642	1.646033677
ILMN_2311873	-0.051255788	5.907226564	-3.58318778	0.000767561	0.025766729	-0.746248271
ILMN_1907966	0.051222434	6.077735411	3.613084296	0.000700701	0.024636243	-0.662031987
ILMN_1656327	-0.051128544	5.591197549	-3.434960924	0.001199452	0.033496112	-1.157703127
ILMN_1757825	-0.051063924	5.640295739	-3.314736078	0.001710858	0.042101033	-1.483658294
ILMN_1683798	0.051004201	6.135779362	3.837324566	0.000349811	0.016657556	-0.017987719
ILMN_1861270	-0.050970799	6.065111977	-3.407059075	0.001303237	0.035350425	-1.233986657
ILMN_2048477	-0.050613337	5.872809379	-3.256836024	0.002025284	0.047114511	-1.638039387
ILMN_1801572	-0.050506336	5.635066496	-3.878462867	0.000307326	0.015581419	0.102430826
ILMN_1766247	0.049839251	6.790933682	3.477518671	0.001056164	0.03106853	-1.040627169
ILMN_1714527	-0.049684048	9.882827743	-3.428761146	0.00122181	0.033786296	-1.17468597
ILMN_1794987	0.049666037	6.293462547	3.487480262	0.001025062	0.030580526	-1.013098166
ILMN_2415572	-0.049400291	5.916208828	-3.387460493	0.00138118	0.036516535	-1.287341686
ILMN_1777060	0.049396003	6.197063807	3.493753091	0.001005927	0.030394687	-0.995739033
ILMN_1662021	0.049054491	6.45210051	3.389890481	0.001371281	0.036422403	-1.280736562
ILMN_2261099	0.048481807	5.366453395	3.412995593	0.001280464	0.034925074	-1.217787985
ILMN_1776325	-0.04844122	7.33716615	-3.43524349	0.001198443	0.033496112	-1.15692866
ILMN_3248069	-0.048280436	13.8188129	-3.515489602	0.000942221	0.029212243	-0.93544325
ILMN_1748407	0.048122896	6.545727881	3.332646665	0.001623358	0.040673606	-1.435555763

ILMN_1802766	0.048106066	6.196486709	3.496111771	0.00099882	0.030259518	-0.989206932
ILMN_2395981	-0.047927549	6.196732747	-3.457466279	0.00112152	0.032172773	-1.095899184
ILMN_2382558	0.047557563	6.634568082	3.421302099	0.001249235	0.034372413	-1.195093592
ILMN_1680703	0.047455947	10.15743726	3.564625019	0.000812099	0.026563495	-0.798334675
ILMN_1651310	0.047222886	5.642809825	3.354328408	0.001523106	0.039187017	-1.377108621
ILMN_2144352	0.047112501	6.173444441	3.282605992	0.001879129	0.044661002	-1.569540894
ILMN_1730791	0.047039316	6.350791687	3.482740016	0.001039752	0.030848947	-1.026203767
ILMN_1750469	-0.046984998	5.39683752	-3.332797395	0.00162264	0.040673606	-1.435150258
ILMN_1677815	-0.04676853	5.463738532	-3.282509254	0.001879659	0.044661002	-1.56979867
ILMN_3263072	-0.046752082	5.719879313	-3.293126278	0.00182238	0.043874554	-1.541478718
ILMN_1670715	-0.046352623	5.830224425	-3.296211153	0.001806049	0.043694978	-1.533239248
ILMN_2388090	0.046142575	5.945175386	3.509002009	0.000960821	0.029523397	-0.953462639
ILMN_1791078	0.045854784	5.184898556	3.259839411	0.002007713	0.046877323	-1.630073876
ILMN_1697512	-0.045486105	5.891820155	-3.308706744	0.001741306	0.042537113	-1.499814682
ILMN_1694414	0.04495986	5.429101362	3.401469119	0.001325031	0.03556843	-1.249223931
ILMN_1712530	0.044705186	8.099304294	3.401023338	0.001326784	0.035569364	-1.250438395
ILMN_1737498	0.044274481	6.296437908	3.360911479	0.001493849	0.038607137	-1.359316128
ILMN_2049672	-0.044102568	5.240342546	-3.606860484	0.000714143	0.024876348	-0.679597134
ILMN_2408764	-0.043561931	5.412848638	-3.6078665	0.000711954	0.024854261	-0.676759081
ILMN_1840894	0.043501707	6.43633132	3.243957774	0.002102288	0.048158469	-1.672141512
ILMN_1767433	0.042732354	6.231968029	3.259041184	0.002012369	0.046877323	-1.632191371
ILMN_1827968	-0.042660657	6.000497664	-3.770512431	0.000431113	0.018733435	-0.212098944
ILMN_1765729	0.042418539	6.196997197	3.284875923	0.001866745	0.04456918	-1.563490814
ILMN_1887128	0.042083821	6.128285112	3.345265505	0.001564275	0.039910084	-1.40156806
ILMN_1748141	0.042006859	6.114728227	3.288730076	0.001845894	0.044255244	-1.553212215
ILMN_1788625	0.041413945	6.600990258	3.29055447	0.001836101	0.04414325	-1.548344098
ILMN_1817251	0.041404039	5.987983834	3.442482185	0.001172851	0.033168616	-1.137075452
ILMN_1706758	-0.041366859	5.513888897	-3.444125662	0.001167114	0.033143858	-1.132564449
ILMN_2163187	0.040296549	5.708989621	3.472954277	0.001070714	0.031230055	-1.053225211
ILMN_1736234	0.039261134	8.67760602	3.489328395	0.001019389	0.030580526	-1.007985662
ILMN_1766565	-0.039234073	5.405055651	-3.397221108	0.001341825	0.035944632	-1.260793046
ILMN_3265439	-0.038471676	5.761816542	-3.406640019	0.001304859	0.035366595	-1.235129464
ILMN_3246292	0.03570426	13.61234894	3.402412885	0.001321327	0.035548673	-1.246652457

Supplementary Table S5. List of genes that were significantly differentially expressed following HPV16 early gene depletion in W12 cells.

DEGs.siRNA.probeset	DEGs.siRNA.symbol	E6.associated	E7.associated
ILMN_2301083	UBE2C	FALSE	TRUE
ILMN_1663390	CDC20	TRUE	FALSE
ILMN_1686097	TOP2A	TRUE	FALSE
ILMN_1801939	CCNB2	TRUE	FALSE
ILMN_1751444	NCAPG	FALSE	FALSE
ILMN_1747911	CDK1	FALSE	FALSE
ILMN_1751776	CKAP2L	TRUE	FALSE
ILMN_1684217	AURKB	TRUE	FALSE
ILMN_1714730	UBE2C	FALSE	TRUE
ILMN_1654268	HMGB2	FALSE	FALSE
ILMN_1747016	CEP55	FALSE	FALSE
ILMN_1726720	NUSAP1	FALSE	FALSE
ILMN_2409220	HMMR	TRUE	FALSE
ILMN_3239771	DLGAP5	TRUE	FALSE
ILMN_1670238	CDC45	FALSE	FALSE
ILMN_1683450	CDCA5	TRUE	FALSE
ILMN_1786125	CCNA2	TRUE	FALSE
ILMN_1749829	DLGAP5	TRUE	FALSE
ILMN_1695658	KIF20A	FALSE	FALSE
ILMN_1815184	ASPM	TRUE	FALSE
ILMN_1728934	PRC1	TRUE	FALSE
ILMN_1777564	MAD2L1	TRUE	FALSE
ILMN_1781942	HMMR	FALSE	FALSE
ILMN_2349459	BIRC5	FALSE	TRUE
ILMN_1666305	CDKN3	FALSE	FALSE
ILMN_1801257	CENPA	FALSE	FALSE
ILMN_2075334	HIST1H4C	FALSE	FALSE
ILMN_2196984	OIP5	FALSE	FALSE
ILMN_1789123	PLK4	TRUE	FALSE
ILMN_1678669	RRM2	TRUE	FALSE
ILMN_2202948	BUB1	TRUE	FALSE
ILMN_1670353	RAD51AP1	TRUE	FALSE
ILMN_1809590	GINS2	FALSE	FALSE
ILMN_1802819	DEPDC1	FALSE	FALSE
ILMN_1788166	TTK	TRUE	FALSE
ILMN_2210129	PRIM1	FALSE	FALSE
ILMN_1703906	HJURP	FALSE	FALSE
ILMN_1737728	CDCA3	TRUE	FALSE
ILMN_2357438	AURKA	FALSE	TRUE
ILMN_2409298	NUSAP1	TRUE	FALSE
ILMN_1796949	TPX2	FALSE	FALSE
ILMN_1737195	CENPK	TRUE	FALSE

ILMN_2368718	CENPM	FALSE	FALSE
ILMN_1739645	ANLN	FALSE	FALSE
ILMN_2212909	MELK	TRUE	FALSE
ILMN_1693471	KLHL35	TRUE	FALSE
ILMN_1712803	CCNB1	TRUE	FALSE
ILMN_1680955	AURKA	FALSE	TRUE
ILMN_3232696	DHFR	FALSE	FALSE
ILMN_2051373	NEK2	TRUE	FALSE
ILMN_2222008	KIFC1	FALSE	FALSE
ILMN_2219712	HMGB2	TRUE	FALSE
ILMN_2041046	CKS1B	TRUE	FALSE
ILMN_2413898	MCM10	TRUE	FALSE
ILMN_2042771	PTTG1	FALSE	FALSE
ILMN_2049021	PTTG3P	FALSE	FALSE
ILMN_1694502	PRIM1	FALSE	FALSE
ILMN_1719256	CKS1B	TRUE	FALSE
ILMN_2148796	MND1	FALSE	FALSE
ILMN_1806037	TK1	FALSE	FALSE
ILMN_1753196	PTTG1	FALSE	FALSE
ILMN_1731184	MELK	TRUE	FALSE
ILMN_1664516	CENPF	FALSE	FALSE
ILMN_2285996	KIAA0101	TRUE	FALSE
ILMN_1651237	CDT1	FALSE	FALSE
ILMN_1786065	UHRF1	FALSE	FALSE
ILMN_1664511	NDC80	FALSE	FALSE
ILMN_1781943	FAM83D	FALSE	FALSE
ILMN_1761903	KCNS1	FALSE	FALSE
ILMN_2181432	PAPL	FALSE	FALSE
ILMN_1710676	FBXO5	FALSE	FALSE
ILMN_1796589	TRIP13	TRUE	FALSE
ILMN_1704537	PHGDH	TRUE	FALSE
ILMN_1673721	EXO1	TRUE	FALSE
ILMN_1790100	DDIAS	TRUE	FALSE
ILMN_1763907	CENPW	TRUE	FALSE
ILMN_2160929	FEN1	FALSE	FALSE
ILMN_1700337	TROAP	FALSE	TRUE
ILMN_1794539	KIF11	FALSE	FALSE
ILMN_1724489	RFC4	FALSE	FALSE
ILMN_2330861	SMC4	FALSE	FALSE
ILMN_2362549	ZWINT	FALSE	FALSE
ILMN_1712452	KIF20B	FALSE	FALSE
ILMN_1780667	POC1A	TRUE	FALSE
ILMN_1756326	CKS2	FALSE	FALSE
ILMN_1658143	RFC3	FALSE	FALSE
ILMN_1759277	OIP5	FALSE	FALSE
ILMN_1711470	UBE2T	FALSE	FALSE

ILMN_1778543	DHFR	TRUE	FALSE
ILMN_1724407	TACC3	FALSE	FALSE
ILMN_1727055	PARPBP	FALSE	FALSE
ILMN_1685916	KIF2C	FALSE	FALSE
ILMN_2157240	MNS1	FALSE	FALSE
ILMN_1673673	PBK	TRUE	FALSE
ILMN_1671906	MND1	TRUE	FALSE
ILMN_1716279	CENPE	TRUE	FALSE
ILMN_1720526	CENPN	FALSE	FALSE
ILMN_2330307	SLC43A3	TRUE	FALSE
ILMN_2108357	RPL39L	TRUE	FALSE
ILMN_1771593	RRM1	FALSE	FALSE
ILMN_2143155	KIF11	FALSE	FALSE
ILMN_1811472	KIF23	TRUE	FALSE
ILMN_1773119	CCNF	FALSE	FALSE
ILMN_1754241	MEIOB	TRUE	FALSE
ILMN_2370365	RFC4	FALSE	FALSE
ILMN_1814281	SPC25	FALSE	FALSE
ILMN_1808071	KIF14	FALSE	FALSE
ILMN_2048700	ATAD2	TRUE	FALSE
ILMN_1655642	FANCI	FALSE	FALSE
ILMN_1808591	UBE2S	FALSE	TRUE
ILMN_3220769	CKS1B	FALSE	FALSE
ILMN_1663195	MCM7	FALSE	FALSE
ILMN_1737184	CDCA7	FALSE	FALSE
ILMN_2077550	RACGAP1	TRUE	FALSE
ILMN_1710428	CDK1	FALSE	FALSE
ILMN_1737205	MCM4	TRUE	FALSE
ILMN_2225718	CENPE	TRUE	FALSE
ILMN_1758728	FANCG	FALSE	FALSE
ILMN_1701882	NA	TRUE	FALSE
ILMN_1709294	CDCA8	FALSE	FALSE
ILMN_1671843	PSRC1	FALSE	FALSE
ILMN_2392472	CENPA	FALSE	FALSE
ILMN_2072296	CKS2	FALSE	FALSE
ILMN_2396982	BCL2L12	FALSE	TRUE
ILMN_1775008	NCAPD2	FALSE	FALSE
ILMN_1657796	STMN1	FALSE	FALSE
ILMN_1776490	C17orf53	FALSE	FALSE
ILMN_1712413	RPL39L	TRUE	FALSE
ILMN_1740291	POLQ	FALSE	FALSE
ILMN_1757697	NEIL3	TRUE	FALSE
ILMN_2235137	FANCD2	TRUE	FALSE
ILMN_1774336	POLE2	FALSE	FALSE
ILMN_1777233	E2F2	TRUE	FALSE
ILMN_1659364	RFC5	TRUE	FALSE

ILMN_1705153	NEFH	FALSE	FALSE
ILMN_2412384	CCNE2	FALSE	FALSE
ILMN_1765701	TUBA1B	FALSE	FALSE
ILMN_1752953	BCL2L12	FALSE	TRUE
ILMN_1725260	CDC25C	FALSE	FALSE
ILMN_1716445	DTYMK	TRUE	FALSE
ILMN_2355665	MTFP1	FALSE	FALSE
ILMN_1798654	MCM6	TRUE	FALSE
ILMN_1772910	GAS1	TRUE	TRUE
ILMN_1665559	CDK2	FALSE	FALSE
ILMN_2072622	ERVMER34-1	TRUE	FALSE
ILMN_2368721	CENPM	FALSE	FALSE
ILMN_2338323	CDC25B	FALSE	FALSE
ILMN_1781479	SUV39H1	FALSE	FALSE
ILMN_1728972	FAM64A	FALSE	TRUE
ILMN_1695414	ASF1B	FALSE	FALSE
ILMN_1768816	TMPO	FALSE	FALSE
ILMN_1683120	UNG	FALSE	FALSE
ILMN_3228822	TMEM194A	FALSE	FALSE
ILMN_1711005	CDC25A	TRUE	FALSE
ILMN_1731070	ORC6	TRUE	FALSE
ILMN_2413650	STIL	FALSE	FALSE
ILMN_1814122	MDC1	TRUE	FALSE
ILMN_1732688	DUT	FALSE	FALSE
ILMN_2362545	ZWINT	FALSE	FALSE
ILMN_1771039	NA	FALSE	FALSE
ILMN_3234884	KIF22	TRUE	FALSE
ILMN_1776577	DSCC1	FALSE	FALSE
ILMN_1806040	TYMS	FALSE	FALSE
ILMN_1729142	CENPV	FALSE	TRUE
ILMN_2407619	CDC25C	FALSE	FALSE
ILMN_2311089	BRCA1	TRUE	FALSE
ILMN_1745420	PHF19	FALSE	FALSE
ILMN_1658027	RAD54L	TRUE	FALSE
ILMN_1668814	CENPM	FALSE	FALSE
ILMN_1799667	KIF4A	TRUE	FALSE
ILMN_2344971	FOXM1	TRUE	FALSE
ILMN_1702197	SAPCD2	FALSE	FALSE
ILMN_1710962	TMEM97	FALSE	FALSE
ILMN_3306440	TMEM194A	FALSE	FALSE
ILMN_1713249	PHF19	FALSE	FALSE
ILMN_2412860	MCM4	FALSE	FALSE
ILMN_1658407	SLC43A3	FALSE	FALSE
ILMN_1799516	DNAJC9	FALSE	FALSE
ILMN_1669479	MEST	TRUE	TRUE
ILMN_2384807	LRRCC1	FALSE	FALSE

ILMN_2410038	FAM111A	FALSE	FALSE
ILMN_1719870	FAM72D	TRUE	FALSE
ILMN_1790537	RMI2	FALSE	TRUE
ILMN_1717173	ECT2	TRUE	FALSE
ILMN_2413899	MCM10	TRUE	FALSE
ILMN_1696713	POLA2	TRUE	FALSE
ILMN_2251804	SMC4	FALSE	FALSE
ILMN_2330243	NUDT1	FALSE	FALSE
ILMN_1782403	PRR11	TRUE	FALSE
ILMN_1805828	VRK1	TRUE	FALSE
ILMN_1742981	TUBA1A	FALSE	FALSE
ILMN_1742922	PRIM2	TRUE	FALSE
ILMN_1656452	C16orf59	FALSE	FALSE
ILMN_1746359	RERG	FALSE	FALSE
ILMN_1653553	C14orf80	TRUE	FALSE
ILMN_2148012	NA	FALSE	FALSE
ILMN_1730825	SGOL1	TRUE	FALSE
ILMN_2074258	BARD1	FALSE	FALSE
ILMN_1779711	DTL	FALSE	FALSE
ILMN_1735093	TIMELESS	FALSE	FALSE
ILMN_1753063	KIF15	FALSE	FALSE
ILMN_1736176	PLK1	TRUE	FALSE
ILMN_1673820	HLTF	FALSE	FALSE
ILMN_1779448	EFHD1	FALSE	FALSE
ILMN_1658695	NUF2	TRUE	FALSE
ILMN_1652008	KNSTRN	TRUE	FALSE
ILMN_1681503	MCM2	FALSE	FALSE
ILMN_1666208	MIS18BP1	FALSE	FALSE
ILMN_1715616	LRR1	TRUE	FALSE
ILMN_1674231	CHAF1B	FALSE	FALSE
ILMN_1700896	SAP30	TRUE	FALSE
ILMN_1747630	DEK	FALSE	FALSE
ILMN_2194828	PAGR1	FALSE	FALSE
ILMN_1795822	DIS3L	FALSE	FALSE
ILMN_2224143	MCM3	TRUE	FALSE
ILMN_1738027	BRCA1	FALSE	FALSE
ILMN_1678493	CHN1	FALSE	FALSE
ILMN_1704702	MCM7	TRUE	FALSE
ILMN_1716895	RPA3	FALSE	FALSE
ILMN_1727540	C1orf112	FALSE	FALSE
ILMN_1815169	MCM5	FALSE	FALSE
ILMN_1747078	HYLS1	FALSE	FALSE
ILMN_1776052	HMGN2	FALSE	FALSE
ILMN_2066756	NCAPG2	FALSE	FALSE
ILMN_1721868	KPNA2	TRUE	FALSE
ILMN_1746699	SGOL2	TRUE	FALSE

ILMN_1751656	KLF11	FALSE	FALSE
ILMN_1736816	SKA3	FALSE	FALSE
ILMN_1712389	NA	FALSE	FALSE
ILMN_1767665	GPX8	FALSE	FALSE
ILMN_1784860	RFC3	FALSE	FALSE
ILMN_1732516	KNTC1	FALSE	FALSE
ILMN_1741801	CDC7	FALSE	FALSE
ILMN_1785756	H2AFX	FALSE	FALSE
ILMN_2141807	KNSTRN	FALSE	FALSE
ILMN_1761486	BORA	FALSE	FALSE
ILMN_1678037	HIRIP3	TRUE	FALSE
ILMN_1657547	CCDC34	FALSE	FALSE
ILMN_2058141	HMGN2	FALSE	TRUE
ILMN_2355738	INCENP	FALSE	FALSE
ILMN_1787280	AUNIP	TRUE	TRUE
ILMN_1664630	CHEK1	FALSE	FALSE
ILMN_3251592	SAPCD2	FALSE	FALSE
ILMN_2167922	TRMT5	FALSE	TRUE
ILMN_1755834	FEN1	FALSE	FALSE
ILMN_1705301	TEAD4	TRUE	FALSE
ILMN_2374633	ZWILCH	FALSE	FALSE
ILMN_1689327	HMGN2	FALSE	TRUE
ILMN_1712386	MIS18A	FALSE	FALSE
ILMN_1767523	IL17RB	FALSE	FALSE
ILMN_1658010	CENPI	FALSE	FALSE
ILMN_1754272	GINS3	FALSE	FALSE
ILMN_1806818	MCM3	FALSE	FALSE
ILMN_3176989	HAUS8	FALSE	FALSE
ILMN_1777691	ARL9	FALSE	FALSE
ILMN_2374778	DUT	FALSE	FALSE
ILMN_1709484	BLM	TRUE	FALSE
ILMN_3243291	HNRNPA1	FALSE	FALSE
ILMN_2288784	CCDC34	FALSE	FALSE
ILMN_1678300	NA	TRUE	FALSE
ILMN_1766658	PKMYT1	TRUE	FALSE
ILMN_1729051	MSH6	FALSE	FALSE
ILMN_1689001	CDK4	FALSE	FALSE
ILMN_1753183	CDCA4	FALSE	FALSE
ILMN_2132161	KIF18A	FALSE	FALSE
ILMN_2193315	EFCAB11	FALSE	FALSE
ILMN_3274929	GPAT2	FALSE	FALSE
ILMN_1707493	RCC1	FALSE	FALSE
ILMN_2101375	CCDC77	FALSE	FALSE
ILMN_3253579	HAUS8	FALSE	FALSE
ILMN_1813028	CBX5	FALSE	FALSE
ILMN_1708516	PRTFDC1	FALSE	FALSE

ILMN_2202423	HELLS	FALSE	FALSE
ILMN_1715189	LHX6	FALSE	FALSE
ILMN_2411190	SMC2	FALSE	FALSE
ILMN_1651557	KDELC2	FALSE	FALSE
ILMN_1756550	NUP62CL	TRUE	FALSE
ILMN_1736242	PLEKHG4	FALSE	FALSE
ILMN_1662184	C5orf34	TRUE	FALSE
ILMN_3239785	NA	FALSE	FALSE
ILMN_1756162	EXOSC8	FALSE	FALSE
ILMN_1682336	MASTL	TRUE	FALSE
ILMN_1696975	USP1	FALSE	FALSE
ILMN_1801913	PPIH	FALSE	FALSE
ILMN_1748923	SMC2	TRUE	FALSE
ILMN_1701603	ALPL	FALSE	FALSE
ILMN_3246608	CENPV	FALSE	TRUE
ILMN_1810901	RNASEH2A	FALSE	FALSE
ILMN_2414027	NA	FALSE	FALSE
ILMN_2043452	FANCE	FALSE	FALSE
ILMN_2395240	CHEK2	TRUE	FALSE
ILMN_2215881	ARHGAP11B	FALSE	FALSE
ILMN_1698252	FANCB	FALSE	FALSE
ILMN_1793360	NA	FALSE	FALSE
ILMN_1681754	GGH	FALSE	FALSE
ILMN_1704987	ZFR2	FALSE	FALSE
ILMN_1709085	GSG2	TRUE	FALSE
ILMN_1695357	SPDL1	FALSE	FALSE
ILMN_1653822	NEK2	TRUE	FALSE
ILMN_1651433	DCK	TRUE	FALSE
ILMN_2371700	HAUS7	TRUE	FALSE
ILMN_1686871	PARP1	FALSE	FALSE
ILMN_3297996	NA	FALSE	FALSE
ILMN_1672536	FBLN1	FALSE	FALSE
ILMN_1721457	RANBP1	FALSE	FALSE
ILMN_2180371	FAM216A	TRUE	FALSE
ILMN_1778845	FAM111A	FALSE	FALSE
ILMN_1745946	HAUS1	FALSE	FALSE
ILMN_1874530	DIAPH3	FALSE	FALSE
ILMN_3243945	MZT1	FALSE	FALSE
ILMN_1652580	POLD1	FALSE	TRUE
ILMN_2103685	DEPDC1B	FALSE	FALSE
ILMN_1771652	BAIAP2L2	FALSE	FALSE
ILMN_2151488	RMI1	FALSE	FALSE
ILMN_1747303	DDX39A	FALSE	FALSE
ILMN_1668910	CIDEB	FALSE	FALSE
ILMN_1760201	DNMT1	FALSE	FALSE
ILMN_1787259	RNF212	FALSE	FALSE

ILMN_1711462	MNS1	FALSE	FALSE
ILMN_1797964	ARL6IP6	FALSE	FALSE
ILMN_1788701	PSIP1	FALSE	FALSE
ILMN_2372040	MTFP1	FALSE	FALSE
ILMN_1688642	LAMC3	FALSE	TRUE
ILMN_3265797	HMGB1	FALSE	FALSE
ILMN_1721901	CTNNAL1	FALSE	FALSE
ILMN_1658607	DLEU2L	FALSE	FALSE
ILMN_1666553	SLC25A19	FALSE	FALSE
ILMN_1786433	BCCIP	FALSE	FALSE
ILMN_1708101	LMNB2	FALSE	FALSE
ILMN_2109156	RANBP1	FALSE	FALSE
ILMN_1663447	HNRNPA1	FALSE	FALSE
ILMN_1715947	NA	FALSE	FALSE
ILMN_1749930	NDC1	TRUE	FALSE
ILMN_1787477	CENPO	TRUE	FALSE
ILMN_1814600	DEPDC1B	FALSE	FALSE
ILMN_2297626	PEG10	TRUE	FALSE
ILMN_1660222	MTBP	FALSE	FALSE
ILMN_2139816	GPSM2	FALSE	FALSE
ILMN_3215712	NA	FALSE	FALSE
ILMN_3212373	NA	TRUE	FALSE
ILMN_1660654	CDCA2	FALSE	FALSE
ILMN_1749875	NA	FALSE	FALSE
ILMN_1723412	ASCL2	TRUE	FALSE
ILMN_1719906	HADH	FALSE	FALSE
ILMN_1697703	HPDL	FALSE	FALSE
ILMN_1791002	SKP2	TRUE	FALSE
ILMN_1757467	H1F0	FALSE	FALSE
ILMN_1732150	KIAA0101	FALSE	FALSE
ILMN_1679134	NSMCE4A	FALSE	FALSE
ILMN_1694177	PCNA	FALSE	FALSE
ILMN_2135984	MASTL	TRUE	FALSE
ILMN_1797307	BUB1B	TRUE	FALSE
ILMN_1732074	NA	FALSE	FALSE
ILMN_1697998	MTFR2	FALSE	FALSE
ILMN_1754051	RMI1	FALSE	FALSE
ILMN_1715437	CASC5	FALSE	FALSE
ILMN_1742779	CENPL	FALSE	FALSE
ILMN_2131336	TMEM194A	FALSE	FALSE
ILMN_1667893	TNS3	TRUE	FALSE
ILMN_1707858	H2AFZ	FALSE	FALSE
ILMN_1771224	SKA1	FALSE	FALSE
ILMN_1679438	CENPU	FALSE	FALSE
ILMN_1778890	LRR1	FALSE	FALSE
ILMN_1716400	FOXM1	TRUE	FALSE

ILMN_3265365	CEP78	TRUE	FALSE
ILMN_1756572	COQ2	FALSE	FALSE
ILMN_1796923	LOC81691	FALSE	FALSE
ILMN_1674411	CKAP2	FALSE	FALSE
ILMN_1670134	FADS1	FALSE	FALSE
ILMN_1705231	SLCO2A1	FALSE	FALSE
ILMN_1803124	BIRC5	FALSE	FALSE
ILMN_1780699	THAP11	FALSE	FALSE
ILMN_2130635	FOXRED2	FALSE	FALSE
ILMN_1758629	DONSON	FALSE	FALSE
ILMN_1750711	MYO19	FALSE	FALSE
ILMN_1723971	SLC29A1	TRUE	FALSE
ILMN_3191227	DBF4	FALSE	FALSE
ILMN_2265654	UBE2C	FALSE	TRUE
ILMN_1783610	HELLS	FALSE	FALSE
ILMN_1720114	GMNN	FALSE	FALSE
ILMN_1721713	EXOSC9	TRUE	FALSE
ILMN_1677906	NA	TRUE	FALSE
ILMN_1665583	TUBB	FALSE	FALSE
ILMN_1692790	ITGB3BP	TRUE	TRUE
ILMN_2191436	POLA1	FALSE	FALSE
ILMN_2136446	CTNNAL1	FALSE	FALSE
ILMN_3305273	PSAT1	TRUE	FALSE
ILMN_1802951	NUF2	FALSE	FALSE
ILMN_1810228	TTF2	TRUE	FALSE
ILMN_3304898	TUBB	FALSE	FALSE
ILMN_1726574	CACYBP	FALSE	FALSE
ILMN_2126706	LMNB1	FALSE	FALSE
ILMN_1756705	CHTF18	FALSE	FALSE
ILMN_1771903	NUP37	TRUE	FALSE
ILMN_2171183	MIS18A	FALSE	FALSE
ILMN_3307266	PSMC3IP	TRUE	FALSE
ILMN_1729112	CHPT1	FALSE	FALSE
ILMN_1685928	WDR34	FALSE	FALSE
ILMN_2338963	SLC29A1	TRUE	FALSE
ILMN_1765332	TIMM10	FALSE	FALSE
ILMN_2169089	C18orf54	FALSE	FALSE
ILMN_1755862	PFAS	TRUE	TRUE
ILMN_1653443	CDK2	FALSE	FALSE
ILMN_1686920	CCDC58	FALSE	FALSE
ILMN_1671554	LPIN1	FALSE	FALSE
ILMN_2137084	LIN9	FALSE	FALSE
ILMN_1696266	XRCC3	FALSE	FALSE
ILMN_3297455	OIP5-AS1	FALSE	FALSE
ILMN_1777066	NIF3L1	FALSE	FALSE
ILMN_2404906	SGOL1	FALSE	FALSE

ILMN_3298582	PTMA	FALSE	FALSE
ILMN_2155272	PIF1	FALSE	FALSE
ILMN_3300972	SIVA1	FALSE	FALSE
ILMN_1743065	PTPLAD1	FALSE	FALSE
ILMN_2396948	PSMC3IP	FALSE	FALSE
ILMN_1699623	FAM81A	FALSE	FALSE
ILMN_1700728	KRTCAP3	FALSE	FALSE
ILMN_1729115	UBE2S	FALSE	FALSE
ILMN_3290261	CACYBP	FALSE	FALSE
ILMN_1814589	KIF22	TRUE	FALSE
ILMN_1671933	GPSM2	TRUE	FALSE
ILMN_1671766	F12	FALSE	FALSE
ILMN_1798210	E2F7	FALSE	FALSE
ILMN_1724062	LIN54	FALSE	FALSE
ILMN_1670769	CENPQ	FALSE	FALSE
ILMN_3251423	CHDH	TRUE	FALSE
ILMN_2202915	FAR2	FALSE	FALSE
ILMN_1738681	NUP62	FALSE	TRUE
ILMN_3240003	CHEK2P2	FALSE	FALSE
ILMN_1709953	DONSON	FALSE	FALSE
ILMN_1672503	DPYSL2	FALSE	FALSE
ILMN_3215759	HAUS1	FALSE	FALSE
ILMN_3179891	NA	FALSE	FALSE
ILMN_1773200	CCP110	FALSE	FALSE
ILMN_1715508	NNMT	FALSE	FALSE
ILMN_1682774	TEX30	FALSE	FALSE
ILMN_2199676	CEP152	FALSE	FALSE
ILMN_1696046	SIVA1	FALSE	FALSE
ILMN_3237584	CHEK2	FALSE	FALSE
ILMN_2091590	ANKLE1	FALSE	FALSE
ILMN_2200331	H2AFX	FALSE	FALSE
ILMN_1737398	PTPLAD1	FALSE	FALSE
ILMN_2334204	ERCC6L	TRUE	FALSE
ILMN_1692056	HS3ST3A1	FALSE	TRUE
ILMN_1691611	HNRNPA1P10	FALSE	FALSE
ILMN_1760635	RAD51C	TRUE	FALSE
ILMN_2356578	TH	FALSE	FALSE
ILMN_3246401	AIF1L	FALSE	FALSE
ILMN_1684802	TAF5	FALSE	FALSE
ILMN_1751368	HNRNPD	FALSE	FALSE
ILMN_2238928	RAD51C	FALSE	FALSE
ILMN_1800619	BRI3BP	FALSE	FALSE
ILMN_1801766	CCDC109B	FALSE	FALSE
ILMN_2401436	PKMYT1	TRUE	FALSE
ILMN_1682494	RSRC1	FALSE	FALSE
ILMN_1704750	TUBB	FALSE	FALSE

ILMN_1722127	RAD54B	FALSE	FALSE
ILMN_1656574	PCGF6	FALSE	FALSE
ILMN_1751963	ZCWPW1	FALSE	FALSE
ILMN_2117904	ZNF22	FALSE	FALSE
ILMN_1753582	RPA2	FALSE	FALSE
ILMN_1657898	MTFP1	FALSE	FALSE
ILMN_1706779	LIG1	FALSE	TRUE
ILMN_1815723	NUP35	TRUE	FALSE
ILMN_3291472	NA	FALSE	FALSE
ILMN_1715991	SDPR	FALSE	FALSE
ILMN_1880052	NA	FALSE	FALSE
ILMN_1671603	MED30	FALSE	TRUE
ILMN_1806483	ZWILCH	FALSE	FALSE
ILMN_1719205	FBL	FALSE	FALSE
ILMN_3205271	HMGB1	FALSE	TRUE
ILMN_1652913	EZH2	FALSE	FALSE
ILMN_1727459	ORC1	FALSE	FALSE
ILMN_1748427	ZNF239	FALSE	FALSE
ILMN_2351916	EXO1	FALSE	FALSE
ILMN_1803005	MMACHC	FALSE	FALSE
ILMN_2179726	C16orf93	TRUE	FALSE
ILMN_1660599	GPAT2	FALSE	FALSE
ILMN_1665538	SKP2	FALSE	FALSE
ILMN_1810725	FAM129A	TRUE	FALSE
ILMN_2141259	SPAG5	TRUE	FALSE
ILMN_1695386	RAD51C	TRUE	TRUE
ILMN_3244640	SNORD96A	FALSE	FALSE
ILMN_1737947	LSM5	FALSE	FALSE
ILMN_1676091	NA	FALSE	FALSE
ILMN_2202940	CHPT1	FALSE	FALSE
ILMN_1683441	NCAPD3	FALSE	FALSE
ILMN_1737254	USP1	FALSE	FALSE
ILMN_1731044	NA	FALSE	FALSE
ILMN_1798588	HLTF	FALSE	FALSE
ILMN_3213568	NA	FALSE	FALSE
ILMN_1734734	ZNF519	FALSE	FALSE
ILMN_1768969	LBR	TRUE	FALSE
ILMN_1674620	SGCE	FALSE	TRUE
ILMN_2117171	LMO4	FALSE	TRUE
ILMN_1692938	PSAT1	TRUE	FALSE
ILMN_1764362	LYAR	FALSE	FALSE
ILMN_1781257	ERVMER34-1	TRUE	FALSE
ILMN_2211800	HMGB1	FALSE	TRUE
ILMN_1785198	POLE3	FALSE	FALSE
ILMN_1700546	ELOVL6	TRUE	FALSE
ILMN_1677747	TMPO	TRUE	FALSE

ILMN_1798270	SMCO4	FALSE	FALSE
ILMN_1684293	ANP32B	FALSE	FALSE
ILMN_2105923	RHNO1	FALSE	FALSE
ILMN_1703487	LMO4	FALSE	TRUE
ILMN_1739582	NA	FALSE	FALSE
ILMN_1679880	THOC6	FALSE	FALSE
ILMN_1675219	WDHD1	FALSE	FALSE
ILMN_1810418	LBR	TRUE	FALSE
ILMN_1669550	MAD2L2	FALSE	FALSE
ILMN_1654421	MPHOSPH9	FALSE	FALSE
ILMN_1717403	ARHGEF39	FALSE	FALSE
ILMN_3294365	HMGB3	FALSE	FALSE
ILMN_1708672	ACAT2	TRUE	FALSE
ILMN_1801387	YEATS4	FALSE	FALSE
ILMN_1690523	LRRC20	FALSE	FALSE
ILMN_3234436	HNRNPA1P33	FALSE	FALSE
ILMN_1661776	CENPJ	FALSE	FALSE
ILMN_1672553	SLC43A3	TRUE	FALSE
ILMN_1768293	NUP155	FALSE	FALSE
ILMN_1756043	WDHD1	FALSE	FALSE
ILMN_2056975	HPRT1	FALSE	FALSE
ILMN_1677273	TH	FALSE	FALSE
ILMN_1743402	SIX4	FALSE	FALSE
ILMN_1810100	PBX3	FALSE	FALSE
ILMN_1748770	CKAP5	FALSE	FALSE
ILMN_2137625	GPAT2	FALSE	FALSE
ILMN_1697736	EXOSC2	FALSE	FALSE
ILMN_1764861	ISOC1	FALSE	FALSE
ILMN_1751028	SERPINH1	FALSE	FALSE
ILMN_1667839	UBR7	FALSE	FALSE
ILMN_1745593	STMN1	TRUE	FALSE
ILMN_1749297	ACTL6A	FALSE	FALSE
ILMN_1731193	BCL2L12	FALSE	TRUE
ILMN_1799106	Mar-01	TRUE	FALSE
ILMN_1695271	RPP25	TRUE	FALSE
ILMN_2176768	SEPHS1	FALSE	FALSE
ILMN_2135798	NR2C2AP	FALSE	FALSE
ILMN_1748093	PAFAH1B3	FALSE	FALSE
ILMN_1669842	CHAF1A	FALSE	FALSE
ILMN_1669635	NUP85	TRUE	FALSE
ILMN_1696183	HBQ1	FALSE	FALSE
ILMN_2081335	COA1	TRUE	FALSE
ILMN_2395236	CHEK2	TRUE	FALSE
ILMN_1915076	NA	TRUE	FALSE
ILMN_1703754	CEP192	FALSE	FALSE
ILMN_3304022	NA	FALSE	FALSE

ILMN_2407124	MCM8	FALSE	TRUE
ILMN_2364062	ALYREF	FALSE	FALSE
ILMN_1681340	HSPB11	FALSE	FALSE
ILMN_1771003	HAUS4	FALSE	FALSE
ILMN_1762275	NA	FALSE	TRUE
ILMN_1687107	RFWD3	FALSE	FALSE
ILMN_1671905	SFR1	FALSE	FALSE
ILMN_1772292	PDCD6IPP2	FALSE	FALSE
ILMN_1757877	HCFC1R1	FALSE	FALSE
ILMN_1698185	WDR90	FALSE	FALSE
ILMN_1784467	NUP210	FALSE	FALSE
ILMN_1726928	TCEA3	FALSE	FALSE
ILMN_1813938	CHCHD4	FALSE	FALSE
ILMN_2101885	TUBB	FALSE	FALSE
ILMN_1720745	NA	FALSE	FALSE
ILMN_1715661	TFAM	FALSE	FALSE
ILMN_1811933	SHMT1	FALSE	FALSE
ILMN_1728199	POLE	FALSE	FALSE
ILMN_1738147	NES	FALSE	FALSE
ILMN_1869109	NUCKS1	FALSE	FALSE
ILMN_1748241	CENPP	FALSE	FALSE
ILMN_3242205	GMPS	FALSE	FALSE
ILMN_1756676	PHF19	FALSE	FALSE
ILMN_1703092	RECQL4	FALSE	FALSE
ILMN_1727444	PAGR1	FALSE	FALSE
ILMN_1709483	MRE11A	TRUE	FALSE
ILMN_1802157	THOC1	TRUE	FALSE
ILMN_1778561	WEE1	FALSE	TRUE
ILMN_1726756	FANCB	FALSE	FALSE
ILMN_1740234	GSTO2	FALSE	FALSE
ILMN_2111187	ELOVL6	TRUE	FALSE
ILMN_2321451	HNRNPD	FALSE	FALSE
ILMN_1684929	TOPBP1	FALSE	FALSE
ILMN_1767481	XRCC6BP1	FALSE	FALSE
ILMN_1757210	CALML4	FALSE	FALSE
ILMN_1690371	MRPL11	TRUE	FALSE
ILMN_1684982	PDK4	FALSE	FALSE
ILMN_1804735	CBS	FALSE	FALSE
ILMN_2349393	MDK	FALSE	FALSE
ILMN_1779264	PSMG1	FALSE	FALSE
ILMN_1690586	NA	FALSE	FALSE
ILMN_1750518	ALYREF	TRUE	FALSE
ILMN_1662658	PUS1	TRUE	FALSE
ILMN_2309534	RDM1	FALSE	FALSE
ILMN_2396947	PSMC3IP	TRUE	FALSE
ILMN_1738093	RNFT2	FALSE	FALSE

ILMN_1718807	SMC3	FALSE	FALSE
ILMN_1750062	PPARGC1A	FALSE	TRUE
ILMN_1658464	GTF3A	FALSE	FALSE
ILMN_2383484	C19orf48	FALSE	TRUE
ILMN_1709044	TGIF2	FALSE	TRUE
ILMN_1665797	CSE1L	FALSE	TRUE
ILMN_1705032	SEH1L	FALSE	FALSE
ILMN_3249807	SKA2	FALSE	FALSE
ILMN_1684045	CDCA4	FALSE	FALSE
ILMN_2399431	PRPS2	FALSE	FALSE
ILMN_2296843	GCDH	FALSE	TRUE
ILMN_1659257	TNFRSF8	FALSE	FALSE
ILMN_1694268	HES6	FALSE	FALSE
ILMN_2215656	MAGOH	FALSE	FALSE
ILMN_1660793	PAQR4	FALSE	FALSE
ILMN_1682428	HENMT1	FALSE	FALSE
ILMN_2124951	RBMX	FALSE	FALSE
ILMN_3181695	LSM3	FALSE	FALSE
ILMN_1753353	SLBP	FALSE	FALSE
ILMN_2067370	SNRPF	FALSE	FALSE
ILMN_1664682	DNA2	FALSE	FALSE
ILMN_1716006	C18orf54	FALSE	FALSE
ILMN_1688953	ARHGAP19	FALSE	FALSE
ILMN_3251404	NUCKS1	FALSE	FALSE
ILMN_2312719	EXOSC9	TRUE	FALSE
ILMN_3276822	TMPO	TRUE	FALSE
ILMN_3185161	CEP78	TRUE	FALSE
ILMN_1674662	TICRR	FALSE	FALSE
ILMN_1658678	SAAL1	FALSE	FALSE
ILMN_1736441	PDXP	FALSE	FALSE
ILMN_3292224	NA	FALSE	FALSE
ILMN_1770725	AIF1L	FALSE	FALSE
ILMN_1767260	CCDC138	TRUE	FALSE
ILMN_1785191	TMEM14A	FALSE	FALSE
ILMN_2376953	KCNK2	FALSE	FALSE
ILMN_1747924	NA	FALSE	FALSE
ILMN_1668850	PCSK9	FALSE	FALSE
ILMN_1667050	PRPS1	FALSE	FALSE
ILMN_1787324	ENKD1	FALSE	FALSE
ILMN_2134176	TXLNG	FALSE	FALSE
ILMN_1768020	HAUS8	FALSE	FALSE
ILMN_3206242	GPAT2	TRUE	FALSE
ILMN_1734486	TSEN15	FALSE	FALSE
ILMN_3267670	G2E3	FALSE	FALSE
ILMN_1758293	UBR7	FALSE	FALSE
ILMN_2220283	HNRNPA1P10	FALSE	FALSE

ILMN_1686835	ATAD5	TRUE	FALSE
ILMN_1724493	LYSMD2	TRUE	FALSE
ILMN_3177271	RBMX	FALSE	FALSE
ILMN_3241262	PABPC4L	FALSE	FALSE
ILMN_1767542	THAP10	FALSE	FALSE
ILMN_3227811	HNRNPA1	FALSE	FALSE
ILMN_2043918	DLEU1	FALSE	FALSE
ILMN_2286334	U2SURP	FALSE	FALSE
ILMN_1806825	CEP128	FALSE	FALSE
ILMN_1776582	PDK3	FALSE	FALSE
ILMN_1788213	FRAT2	FALSE	FALSE
ILMN_1706238	CSE1L	FALSE	FALSE
ILMN_2306066	FUS	FALSE	FALSE
ILMN_2062701	GAS1	TRUE	TRUE
ILMN_1693221	CENPH	TRUE	FALSE
ILMN_2322996	EYA2	FALSE	FALSE
ILMN_3251691	POLR3G	TRUE	FALSE
ILMN_1709634	CMBL	TRUE	TRUE
ILMN_1812297	CYP26B1	FALSE	FALSE
ILMN_1800958	TMEM237	TRUE	FALSE
ILMN_3275590	LSM3	FALSE	FALSE
ILMN_2404385	REPIN1	FALSE	FALSE
ILMN_2221564	LYAR	FALSE	FALSE
ILMN_1690252	ALKBH2	TRUE	FALSE
ILMN_1792389	RNF165	FALSE	FALSE
ILMN_1672908	TWIST1	TRUE	FALSE
ILMN_2176251	MGME1	FALSE	FALSE
ILMN_1801391	SKP2	FALSE	FALSE
ILMN_2334205	ERCC6L	FALSE	FALSE
ILMN_2162253	NMU	FALSE	FALSE
ILMN_2325347	B3GALNT1	FALSE	FALSE
ILMN_1708416	ARL6IP1	FALSE	FALSE
ILMN_1757995	PARP2	FALSE	FALSE
ILMN_2369682	HNRNPA2B1	TRUE	FALSE
ILMN_2230683	CDCA7L	FALSE	FALSE
ILMN_1787248	SIVA1	FALSE	FALSE
ILMN_1761939	TIPIN	FALSE	FALSE
ILMN_1708105	EZH2	FALSE	FALSE
ILMN_1736015	JADE1	FALSE	FALSE
ILMN_2169490	TDRD9	FALSE	FALSE
ILMN_1759184	C19orf48	FALSE	TRUE
ILMN_1798164	PHF3	FALSE	TRUE
ILMN_2316540	MRPL11	TRUE	FALSE
ILMN_1678962	DFFB	FALSE	FALSE
ILMN_2266948	SLC38A1	FALSE	FALSE
ILMN_2298936	NA	TRUE	FALSE

ILMN_1753393	OSGEP	FALSE	FALSE
ILMN_2096654	COMMD4	FALSE	FALSE
ILMN_1811110	TDRD9	FALSE	FALSE
ILMN_1659285	PSMG1	FALSE	FALSE
ILMN_2376108	PSMB9	FALSE	FALSE
ILMN_1751773	POLD3	TRUE	FALSE
ILMN_1771734	GMPS	FALSE	FALSE
ILMN_2116714	SLC39A1	FALSE	FALSE
ILMN_1706645	MB21D1	FALSE	FALSE
ILMN_1734827	MKI67	FALSE	TRUE
ILMN_1654690	CECR5	TRUE	FALSE
ILMN_3214052	CACYBP	FALSE	FALSE
ILMN_1783676	CCDC15	FALSE	FALSE
ILMN_1703692	TUBB	FALSE	FALSE
ILMN_1667966	FAM129A	TRUE	FALSE
ILMN_1798533	ZNF22	FALSE	FALSE
ILMN_1664863	CTRL	FALSE	FALSE
ILMN_2120273	AP1S2	FALSE	FALSE
ILMN_1787628	NOP56	FALSE	FALSE
ILMN_3192791	HNRNPM	FALSE	FALSE
ILMN_3208233	NA	FALSE	FALSE
ILMN_1747160	SYT15	FALSE	FALSE
ILMN_1782745	RBL1	FALSE	FALSE
ILMN_2275533	DIAPH3	FALSE	FALSE
ILMN_1652237	CBR3	FALSE	FALSE
ILMN_1674706	MTHFD2	TRUE	FALSE
ILMN_1743397	PIGW	FALSE	FALSE
ILMN_2405521	MTHFD2	TRUE	FALSE
ILMN_1798886	NUDT21	FALSE	FALSE
ILMN_1728225	KIAA1524	FALSE	FALSE
ILMN_1678966	SNRPF	FALSE	TRUE
ILMN_1805395	LTBP3	FALSE	FALSE
ILMN_2044832	NOP56	FALSE	FALSE
ILMN_3197767	NA	FALSE	FALSE
ILMN_2354237	PARP2	FALSE	FALSE
ILMN_1790136	MRGBP	FALSE	TRUE
ILMN_1748894	GTPBP3	FALSE	TRUE
ILMN_2226415	MZT1	FALSE	FALSE
ILMN_1757278	HSPD1	FALSE	FALSE
ILMN_2363621	RBBP8	FALSE	FALSE
ILMN_1651886	CWF19L1	FALSE	TRUE
ILMN_1755677	EMC9	FALSE	FALSE
ILMN_2115633	CD320	FALSE	FALSE
ILMN_1716382	C12orf75	FALSE	FALSE
ILMN_1711799	C9orf40	FALSE	FALSE
ILMN_3242405	RMRP	FALSE	FALSE

ILMN_3256004	SNRPG	FALSE	FALSE
ILMN_2392546	PAICS	TRUE	FALSE
ILMN_1805985	ANKRD32	FALSE	FALSE
ILMN_1807501	GINS4	FALSE	FALSE
ILMN_3249261	RPA1	FALSE	FALSE
ILMN_2184640	NOLC1	TRUE	FALSE
ILMN_1745623	CRACR2B	FALSE	FALSE
ILMN_1893697	NA	FALSE	FALSE
ILMN_2229242	LSM3	FALSE	FALSE
ILMN_1758778	CNTRL	FALSE	FALSE
ILMN_2061768	HES7	TRUE	FALSE
ILMN_1770044	CHRNA5	FALSE	FALSE
ILMN_1678678	SLC37A4	TRUE	FALSE
ILMN_3242459	DCTPP1	FALSE	FALSE
ILMN_2097546	NUDC	FALSE	FALSE
ILMN_1743205	ABCA7	FALSE	FALSE
ILMN_1758104	PRPS2	FALSE	FALSE
ILMN_1692511	TMEM106C	FALSE	FALSE
ILMN_3243268	ALG10	FALSE	FALSE
ILMN_1766000	PM20D2	TRUE	FALSE
ILMN_1797813	SUZ12	FALSE	FALSE
ILMN_1812489	CEP85	FALSE	FALSE
ILMN_3282503	CBX3	FALSE	FALSE
ILMN_1757387	UCHL1	FALSE	FALSE
ILMN_3247064	SNRNP40	FALSE	FALSE
ILMN_1719032	LSM3	FALSE	FALSE
ILMN_2365528	HAUS8	FALSE	FALSE
ILMN_1792712	C4orf46	FALSE	FALSE
ILMN_3197652	ERI2	FALSE	FALSE
ILMN_1744240	TBC1D31	FALSE	FALSE
ILMN_1715908	NCAPH2	FALSE	FALSE
ILMN_2110252	NPM3	FALSE	FALSE
ILMN_2344002	GEMIN2	FALSE	FALSE
ILMN_1800390	ZNF511	FALSE	FALSE
ILMN_1682799	STAMBPL1	FALSE	FALSE
ILMN_1754045	FANCL	FALSE	FALSE
ILMN_1657204	SAE1	FALSE	TRUE
ILMN_1652333	FN3KRP	FALSE	FALSE
ILMN_1738530	ATAD3A	FALSE	FALSE
ILMN_1717707	PSTK	FALSE	FALSE
ILMN_1810922	PCNT	FALSE	FALSE
ILMN_1795719	RPA1	FALSE	FALSE
ILMN_2344007	GEMIN2	FALSE	FALSE
ILMN_1728512	YWHAH	FALSE	FALSE
ILMN_1801664	POLR3K	FALSE	FALSE
ILMN_2310296	C21orf58	FALSE	FALSE

ILMN_2347234	PRMT1	FALSE	FALSE
ILMN_1686458	CEP152	FALSE	FALSE
ILMN_2221006	RAD21	FALSE	FALSE
ILMN_1784946	ORC3	FALSE	FALSE
ILMN_1770678	CBX2	FALSE	FALSE
ILMN_1672094	DLX1	TRUE	TRUE
ILMN_2117987	TFDP1	FALSE	FALSE
ILMN_1654920	HNRNPH3	FALSE	FALSE
ILMN_1700378	ZWILCH	TRUE	FALSE
ILMN_1804953	CCDC18	FALSE	FALSE
ILMN_1783333	CMC2	FALSE	FALSE
ILMN_3289172	KDM4A-AS1	FALSE	FALSE
ILMN_1797318	HSPA14	TRUE	FALSE
ILMN_1736940	HPRT1	FALSE	FALSE
ILMN_1653711	FZD2	FALSE	FALSE
ILMN_1655733	RFC2	FALSE	FALSE
ILMN_1694233	ACYP1	FALSE	FALSE
ILMN_1710937	IFI16	FALSE	FALSE
ILMN_1694126	KIF24	FALSE	FALSE
ILMN_1797181	LOC93622	FALSE	FALSE
ILMN_1694780	GCHFR	FALSE	FALSE
ILMN_1760802	MRE11A	TRUE	FALSE
ILMN_1744830	ARHGAP11A	FALSE	FALSE
ILMN_2357361	THYN1	FALSE	FALSE
ILMN_1736096	DLL3	FALSE	FALSE
ILMN_1782813	DHFR	FALSE	FALSE
ILMN_1776080	GTPBP6	FALSE	FALSE
ILMN_1749410	PKP4	FALSE	FALSE
ILMN_1760849	NETO2	FALSE	FALSE
ILMN_1686968	ZNF362	FALSE	FALSE
ILMN_1652989	NUP160	FALSE	FALSE
ILMN_2255133	BCL11A	FALSE	TRUE
ILMN_1705407	NOP56	FALSE	FALSE
ILMN_1664028	CENPB	FALSE	TRUE
ILMN_1757497	VGF	FALSE	FALSE
ILMN_1670218	EXOSC6	FALSE	FALSE
ILMN_1651262	HNRNPAB	FALSE	FALSE
ILMN_2053281	L3HYPDH	FALSE	FALSE
ILMN_1739946	VKORC1	FALSE	FALSE
ILMN_1714438	MUTYH	FALSE	FALSE
ILMN_1743367	FZD4	FALSE	FALSE
ILMN_1761981	FAM96A	FALSE	FALSE
ILMN_3283742	ANP32B	FALSE	FALSE
ILMN_3290340	CHEK2P2	FALSE	FALSE
ILMN_1716480	ACD	FALSE	FALSE
ILMN_1673962	NUP205	FALSE	FALSE

ILMN_1799103	SNRPB	FALSE	FALSE
ILMN_1693905	HAT1	FALSE	FALSE
ILMN_1671260	WLS	FALSE	FALSE
ILMN_1715905	DSN1	FALSE	FALSE
ILMN_1734205	RASSF1	FALSE	FALSE
ILMN_2188374	XPOT	FALSE	FALSE
ILMN_1803476	KCTD20	FALSE	FALSE
ILMN_1815154	MYH10	FALSE	FALSE
ILMN_2345908	DDX11	FALSE	FALSE
ILMN_3236244	C1orf174	FALSE	FALSE
ILMN_3224758	TUBB	FALSE	FALSE
ILMN_1734096	DCLRE1A	FALSE	FALSE
ILMN_1760280	NXT1	FALSE	FALSE
ILMN_1757384	NA	FALSE	FALSE
ILMN_2391750	SFMBT1	FALSE	FALSE
ILMN_1800261	TUBA1B	FALSE	FALSE
ILMN_2055700	SLBP	FALSE	FALSE
ILMN_1716336	CKAP5	FALSE	FALSE
ILMN_1691097	HSP90AA1	FALSE	FALSE
ILMN_3258321	SNRPG	FALSE	FALSE
ILMN_1803192	WDR76	FALSE	FALSE
ILMN_1653001	CABLES1	FALSE	FALSE
ILMN_1759419	ILVBL	TRUE	FALSE
ILMN_1713875	NA	TRUE	FALSE
ILMN_3272768	LINC00339	TRUE	TRUE
ILMN_1725244	HAT1	FALSE	FALSE
ILMN_3220861	HMGB3	FALSE	FALSE
ILMN_1770244	CBX1	FALSE	FALSE
ILMN_1673207	KLF11	FALSE	FALSE
ILMN_1746888	PCOLCE2	FALSE	FALSE
ILMN_2200880	ZNF367	FALSE	FALSE
ILMN_3305949	NA	FALSE	FALSE
ILMN_1695034	NA	FALSE	FALSE
ILMN_1690307	TPRKB	FALSE	FALSE
ILMN_1778991	NFIB	FALSE	FALSE
ILMN_2411781	RYR1	FALSE	FALSE
ILMN_2196569	NUP93	FALSE	FALSE
ILMN_2325168	ARRB1	FALSE	FALSE
ILMN_2413331	TMEM107	FALSE	FALSE
ILMN_2089458	SASS6	FALSE	FALSE
ILMN_1661695	IRAK3	FALSE	FALSE
ILMN_1748538	ALDH1A2	FALSE	FALSE
ILMN_3245659	ERI1	FALSE	FALSE
ILMN_1729973	ZC3HAV1	FALSE	FALSE
ILMN_1720266	ZGRF1	FALSE	FALSE
ILMN_1681221	NA	FALSE	FALSE

ILMN_2227968	NTHL1	TRUE	FALSE
ILMN_1664369	DHTKD1	TRUE	FALSE
ILMN_2133187	PRSS53	FALSE	FALSE
ILMN_2391231	SORD	FALSE	FALSE
ILMN_2401352	UHRF1	FALSE	FALSE
ILMN_1768110	ZAK	FALSE	FALSE
ILMN_1693145	BUB3	FALSE	TRUE
ILMN_1708059	USP13	TRUE	FALSE
ILMN_1773760	PAICS	TRUE	FALSE
ILMN_1762312	FOXRED1	FALSE	FALSE
ILMN_1784367	HSPD1	TRUE	FALSE
ILMN_1666325	ALG10B	FALSE	FALSE
ILMN_1778152	FIGNL1	FALSE	FALSE
ILMN_1788099	LSM4	FALSE	FALSE
ILMN_2328280	ACTL6A	FALSE	FALSE
ILMN_1682008	TCF19	FALSE	FALSE
ILMN_2112460	MAD2L1	TRUE	FALSE
ILMN_1805973	GPR19	FALSE	FALSE
ILMN_1731612	UCHL5	TRUE	FALSE
ILMN_1797184	PKN3	FALSE	FALSE
ILMN_1754969	LMCD1	FALSE	FALSE
ILMN_1788689	PHIP	FALSE	FALSE
ILMN_1673543	PGM2	TRUE	FALSE
ILMN_2367428	FAM96A	FALSE	FALSE
ILMN_2112402	PHF5A	FALSE	FALSE
ILMN_3240586	PLD6	FALSE	FALSE
ILMN_1673352	IFITM2	FALSE	FALSE
ILMN_1755758	RIF1	TRUE	FALSE
ILMN_1797693	BRI3BP	FALSE	FALSE
ILMN_1748578	RAD21	FALSE	FALSE
ILMN_2323491	NUP62	FALSE	TRUE
ILMN_1670809	NRM	FALSE	FALSE
ILMN_2348975	NASP	FALSE	FALSE
ILMN_1741632	RAB3IL1	FALSE	FALSE
ILMN_1791483	PDE4D	FALSE	FALSE
ILMN_1799015	PXMP2	FALSE	FALSE
ILMN_1759883	SRP9	FALSE	FALSE
ILMN_1766125	LONP1	FALSE	FALSE
ILMN_1713143	MRPL3	FALSE	FALSE
ILMN_3244117	STMN3	FALSE	FALSE
ILMN_1730622	EVL	FALSE	FALSE
ILMN_1716368	PRIMPOL	FALSE	FALSE
ILMN_2210482	MRPS34	FALSE	FALSE
ILMN_2173891	C19orf40	FALSE	FALSE
ILMN_1706958	PCNA	FALSE	FALSE
ILMN_1801118	SNRNP25	TRUE	FALSE

ILMN_1683562	SNRPG	FALSE	FALSE
ILMN_1682567	CCDC106	FALSE	FALSE
ILMN_1790781	DHRS13	FALSE	FALSE
ILMN_1720270	CDR2	FALSE	FALSE
ILMN_1719627	SLC27A3	FALSE	FALSE
ILMN_1665428	GSDMD	FALSE	FALSE
ILMN_2191568	NPRL2	FALSE	TRUE
ILMN_2415583	HS6ST2	FALSE	FALSE
ILMN_1763129	DCTPP1	FALSE	FALSE
ILMN_1708147	TBPL1	FALSE	FALSE
ILMN_1808566	TMEM180	FALSE	FALSE
ILMN_1805996	SIN3A	FALSE	FALSE
ILMN_2061405	NUP54	TRUE	FALSE
ILMN_1808999	ARHGEF19	FALSE	FALSE
ILMN_1717229	AAED1	FALSE	FALSE
ILMN_1673936	KHSRP	FALSE	FALSE
ILMN_2233099	SSRP1	FALSE	FALSE
ILMN_2411559	PUS1	TRUE	FALSE
ILMN_2388547	EPSTI1	FALSE	FALSE
ILMN_2283325	WLS	FALSE	FALSE
ILMN_2219466	APOBEC3B	TRUE	FALSE
ILMN_1663383	GEMIN5	TRUE	FALSE
ILMN_1766411	AP1S2	FALSE	FALSE
ILMN_1672128	ATF4	FALSE	FALSE
ILMN_1683475	TOMM40	FALSE	TRUE
ILMN_3219806	UNC93B1	FALSE	FALSE
ILMN_1808861	METTL15	FALSE	FALSE
ILMN_2073604	EBP	FALSE	FALSE
ILMN_2257432	RAD51	FALSE	FALSE
ILMN_1703324	PDSS1	TRUE	FALSE
ILMN_1800993	CLUAP1	FALSE	FALSE
ILMN_1705753	CMSS1	FALSE	FALSE
ILMN_1712122	FANCD2	TRUE	FALSE
ILMN_3211857	HMGN2	FALSE	FALSE
ILMN_2044085	RQCD1	FALSE	FALSE
ILMN_1689652	RNMTL1	FALSE	FALSE
ILMN_1660806	CSRP2	FALSE	FALSE
ILMN_1762262	PKIA	FALSE	FALSE
ILMN_1793220	GART	FALSE	FALSE
ILMN_1809866	WDR74	TRUE	FALSE
ILMN_2080611	PDSS1	TRUE	FALSE
ILMN_1733690	AKAP7	TRUE	FALSE
ILMN_1799814	SNRNP40	FALSE	FALSE
ILMN_1693340	RAC3	FALSE	FALSE
ILMN_1661432	NUP43	FALSE	FALSE
ILMN_1782504	MTERF3	FALSE	FALSE

ILMN_1680831	BAZ1B	FALSE	FALSE
ILMN_1657347	PODXL2	FALSE	FALSE
ILMN_1702783	SNRPA1	FALSE	FALSE
ILMN_1739210	NSL1	FALSE	FALSE
ILMN_1691949	NA	FALSE	FALSE
ILMN_2166865	ENY2	FALSE	FALSE
ILMN_1789338	SORBS3	FALSE	FALSE
ILMN_1726306	HMBS	FALSE	FALSE
ILMN_1771019	MTMR4	FALSE	FALSE
ILMN_1667825	MLKL	FALSE	FALSE
ILMN_1804834	OARD1	FALSE	FALSE
ILMN_1748697	LIN28B	FALSE	FALSE
ILMN_3211935	SRSF3	FALSE	FALSE
ILMN_1743711	XPOT	TRUE	FALSE
ILMN_3256868	RBMX	FALSE	FALSE
ILMN_1751743	XRCC1	FALSE	TRUE
ILMN_3236021	NA	FALSE	FALSE
ILMN_1681437	DCXR	FALSE	FALSE
ILMN_1795454	CPSF6	FALSE	FALSE
ILMN_1782045	FKBP4	FALSE	FALSE
ILMN_3246353	SNORD65	TRUE	FALSE
ILMN_1723212	SRSF3	FALSE	FALSE
ILMN_3241046	MYBL1	FALSE	FALSE
ILMN_2355042	CLUAP1	FALSE	FALSE
ILMN_1680246	MAT2B	FALSE	FALSE
ILMN_1683023	PDGFC	FALSE	FALSE
ILMN_2049642	RPA1	TRUE	TRUE
ILMN_2070300	LSM2	FALSE	FALSE
ILMN_1782305	NR4A2	FALSE	TRUE
ILMN_1810181	PCGF6	FALSE	FALSE
ILMN_2110167	POLR1E	FALSE	FALSE
ILMN_1751744	ANKLE1	FALSE	FALSE
ILMN_1661886	APEX1	FALSE	FALSE
ILMN_1770732	COPS3	TRUE	FALSE
ILMN_2232157	SLMO1	FALSE	FALSE
ILMN_3251482	ALG10B	FALSE	FALSE
ILMN_1771835	NUP54	FALSE	FALSE
ILMN_2364529	EZH2	FALSE	FALSE
ILMN_3270641	HNRNPH3	FALSE	FALSE
ILMN_1692473	PRMT1	FALSE	FALSE
ILMN_1673450	DDN	FALSE	FALSE
ILMN_1761083	HNRNPA3	FALSE	FALSE
ILMN_1693669	WRAP53	FALSE	FALSE
ILMN_1747870	CD3EAP	FALSE	FALSE
ILMN_1693985	JPH1	FALSE	FALSE
ILMN_1778836	SRSF7	FALSE	FALSE

ILMN_1674302	PPAT	TRUE	FALSE
ILMN_1788135	NA	FALSE	FALSE
ILMN_1697363	C20orf27	FALSE	TRUE
ILMN_1679640	FXR1	FALSE	FALSE
ILMN_1739576	CYB5R2	FALSE	FALSE
ILMN_2134555	KCTD3	FALSE	FALSE
ILMN_2184966	ZHX2	FALSE	FALSE
ILMN_1798827	SRBD1	FALSE	FALSE
ILMN_1705570	H2AFY2	FALSE	FALSE
ILMN_1788363	MLH1	FALSE	FALSE
ILMN_1703926	PTGER2	FALSE	FALSE
ILMN_1882000	TXLNG	TRUE	FALSE
ILMN_1770206	GEMIN4	TRUE	FALSE
ILMN_1658373	PAN2	FALSE	FALSE
ILMN_1722811	CDKN1B	FALSE	TRUE
ILMN_2077758	CTDSPL2	FALSE	FALSE
ILMN_1669878	GUSB	FALSE	FALSE
ILMN_2155172	BRIX1	TRUE	FALSE
ILMN_2213199	CEP44	FALSE	FALSE
ILMN_3239426	GPN3	FALSE	FALSE
ILMN_3201453	PRKRIR	TRUE	FALSE
ILMN_1693410	NA	FALSE	FALSE
ILMN_1730888	ZNF680	FALSE	FALSE
ILMN_1765641	SEMA3A	FALSE	FALSE
ILMN_2179837	BANF1	TRUE	FALSE
ILMN_1666894	CSPG4	FALSE	FALSE
ILMN_3246409	HNRNPH1	FALSE	FALSE
ILMN_1778764	BUB3	FALSE	FALSE
ILMN_1652486	THAP7	FALSE	FALSE
ILMN_1654545	CPSF1	FALSE	FALSE
ILMN_2415926	THOC3	FALSE	FALSE
ILMN_2401701	PCGF6	FALSE	FALSE
ILMN_2096191	AASDHPPT	FALSE	FALSE
ILMN_3298829	HMGN2	FALSE	FALSE
ILMN_1751086	ATL3	TRUE	FALSE
ILMN_1685703	ACOX2	FALSE	FALSE
ILMN_2337974	PKIA	FALSE	FALSE
ILMN_1789171	EEF2K	FALSE	FALSE
ILMN_3176746	TMPO-AS1	FALSE	FALSE
ILMN_1689720	PPP2R3B	TRUE	TRUE
ILMN_1658416	MRPS18C	FALSE	FALSE
ILMN_1735719	UNG	FALSE	FALSE
ILMN_1730612	NA	FALSE	FALSE
ILMN_1659462	DUSP23	FALSE	FALSE
ILMN_1746206	CEP131	FALSE	FALSE
ILMN_2318568	HCFC1R1	FALSE	FALSE

ILMN_1750102	EME1	FALSE	FALSE
ILMN_1736575	TRIM28	FALSE	TRUE
ILMN_1724907	NA	TRUE	FALSE
ILMN_1695959	EVA1C	FALSE	FALSE
ILMN_1893633	PRKCQ-AS1	TRUE	FALSE
ILMN_1655694	NA	FALSE	FALSE
ILMN_1745172	ILF2	FALSE	FALSE
ILMN_2105983	XRCC5	FALSE	FALSE
ILMN_2335718	HNRNPAB	FALSE	FALSE
ILMN_3233091	UTY	FALSE	FALSE
ILMN_1696311	IMPAD1	FALSE	FALSE
ILMN_1813834	PRMT6	FALSE	FALSE
ILMN_1661717	TFDP1	FALSE	FALSE
ILMN_2045729	WDR12	TRUE	FALSE
ILMN_1752075	MYBPC1	FALSE	FALSE
ILMN_2182531	TIMM21	TRUE	FALSE
ILMN_2139351	ZNF232	FALSE	FALSE
ILMN_2070349	POLR3G	TRUE	FALSE
ILMN_1770053	RBBP7	TRUE	FALSE
ILMN_2389114	FIGNL1	FALSE	FALSE
ILMN_3199647	CBX1	FALSE	FALSE
ILMN_2205935	SFXN1	FALSE	FALSE
ILMN_2217935	RFC1	TRUE	FALSE
ILMN_1724837	ZC3HAV1	FALSE	FALSE
ILMN_1711894	MYB	FALSE	FALSE
ILMN_1733390	LARP1B	FALSE	FALSE
ILMN_1806603	MESP1	FALSE	FALSE
ILMN_1730260	N6AMT2	FALSE	FALSE
ILMN_3260070	RAN	FALSE	FALSE
ILMN_1768662	UCK2	TRUE	FALSE
ILMN_1667510	C12orf65	FALSE	FALSE
ILMN_1753498	COASY	FALSE	FALSE
ILMN_3233388	RELL1	TRUE	TRUE
ILMN_1766713	HSPD1	FALSE	FALSE
ILMN_1693766	CEP135	FALSE	FALSE
ILMN_1769369	ELAVL2	FALSE	FALSE
ILMN_2047676	OSGEPL1	FALSE	TRUE
ILMN_1761961	MCUR1	FALSE	FALSE
ILMN_1807304	MBNL1	FALSE	FALSE
ILMN_1705594	NAT10	FALSE	FALSE
ILMN_1665655	CTDSPL2	FALSE	FALSE
ILMN_2354211	RCC1	FALSE	FALSE
ILMN_1669881	TSPAN13	FALSE	FALSE
ILMN_1676458	MRPL11	FALSE	FALSE
ILMN_1684346	TNFAIP8L1	FALSE	FALSE
ILMN_2319344	APEX1	FALSE	FALSE

ILMN_3187530	CENPI	FALSE	FALSE
ILMN_2077886	C1orf109	FALSE	FALSE
ILMN_1739101	RBBP8	FALSE	FALSE
ILMN_3249366	JMJD8	FALSE	FALSE
ILMN_1702140	RACGAP1	FALSE	FALSE
ILMN_2149400	SPC25	FALSE	FALSE
ILMN_3286312	NEK2	FALSE	FALSE
ILMN_1676905	TIGD7	FALSE	FALSE
ILMN_1759983	DR1	FALSE	FALSE
ILMN_1677239	CCDC14	FALSE	FALSE
ILMN_1792078	RNF114	TRUE	TRUE
ILMN_1764729	JAG2	TRUE	FALSE
ILMN_1679800	BRIX1	FALSE	FALSE
ILMN_1698677	C4orf27	FALSE	FALSE
ILMN_1720996	SLC12A2	FALSE	FALSE
ILMN_1673073	WDR62	FALSE	FALSE
ILMN_1656628	WDR4	FALSE	FALSE
ILMN_2375032	BEND3	FALSE	FALSE
ILMN_1901198	LINC00339	FALSE	FALSE
ILMN_1774890	LAS1L	TRUE	FALSE
ILMN_1708954	GAR1	FALSE	FALSE
ILMN_1661599	DDIT4	TRUE	FALSE
ILMN_1782439	CNN3	TRUE	FALSE
ILMN_1675695	PDS5B	FALSE	FALSE
ILMN_1792748	CPS1	TRUE	TRUE
ILMN_2399769	WLS	FALSE	FALSE
ILMN_1660549	WLS	FALSE	FALSE
ILMN_1790603	ATPAF1	FALSE	FALSE
ILMN_1807044	UBAC1	FALSE	FALSE
ILMN_1736005	NCAPH2	FALSE	FALSE
ILMN_2372200	ZNF586	FALSE	FALSE
ILMN_1683664	FAM60A	FALSE	FALSE
ILMN_2100458	RFESD	TRUE	FALSE
ILMN_1661439	FLOT1	FALSE	FALSE
ILMN_3308663	MIR1228	FALSE	FALSE
ILMN_1687921	JMJD8	FALSE	FALSE
ILMN_2373515	HSP90AA1	FALSE	FALSE
ILMN_1758339	CSTF1	FALSE	TRUE
ILMN_3310113	MIR586	FALSE	FALSE
ILMN_1748926	TMEM209	FALSE	FALSE
ILMN_2411076	MATR3	FALSE	FALSE
ILMN_1815190	METTL1	TRUE	FALSE
ILMN_2392352	CTPS2	FALSE	FALSE
ILMN_1671004	POLG2	FALSE	FALSE
ILMN_1669940	TMEM38B	FALSE	FALSE
ILMN_1711514	COCH	FALSE	FALSE

ILMN_1678934	POLR1E	FALSE	FALSE
ILMN_2145997	SP4	FALSE	FALSE
ILMN_1652163	DVL2	FALSE	FALSE
ILMN_1700604	RBM14	FALSE	FALSE
ILMN_1743373	DLL1	FALSE	FALSE
ILMN_2044617	MTERF3	FALSE	FALSE
ILMN_1730809	SLC29A2	FALSE	FALSE
ILMN_1792435	STAG1	FALSE	FALSE
ILMN_1739429	HS6ST2	FALSE	FALSE
ILMN_3283680	HSPD1	FALSE	FALSE
ILMN_1730077	RPUSD2	FALSE	TRUE
ILMN_2324056	GNL3	TRUE	TRUE
ILMN_1676026	MRPS26	FALSE	TRUE
ILMN_1812777	MRPL35	FALSE	FALSE
ILMN_3243986	FAM72B	FALSE	FALSE
ILMN_1684746	IPO11	FALSE	FALSE
ILMN_1669905	DCP2	FALSE	FALSE
ILMN_1681634	PXMP2	FALSE	FALSE
ILMN_2215545	CMSS1	FALSE	FALSE
ILMN_1765990	KCNK2	FALSE	FALSE
ILMN_2149053	RIF1	TRUE	FALSE
ILMN_1715540	TRAIP	FALSE	FALSE
ILMN_1795336	PTER	TRUE	FALSE
ILMN_2172202	NUDT15	FALSE	FALSE
ILMN_2223380	PPWD1	FALSE	FALSE
ILMN_1790625	CBX3	TRUE	FALSE
ILMN_3177285	HNRNPR	FALSE	FALSE
ILMN_1783075	MMS22L	FALSE	FALSE
ILMN_1789775	WDR74	TRUE	FALSE
ILMN_3249240	C4orf46	FALSE	FALSE
ILMN_1675007	ZSCAN5A	FALSE	TRUE
ILMN_3236713	SNHG1	FALSE	FALSE
ILMN_1800220	KCTD3	FALSE	FALSE
ILMN_1685954	HMBS	FALSE	FALSE
ILMN_1754220	SF3A2	FALSE	FALSE
ILMN_1809477	CARHSP1	FALSE	FALSE
ILMN_1747943	NA	FALSE	FALSE
ILMN_1724497	ABI2	FALSE	FALSE
ILMN_1689800	MRTO4	TRUE	FALSE
ILMN_1684771	PGRMC1	TRUE	FALSE
ILMN_1784880	TAF9B	FALSE	FALSE
ILMN_1712075	SYNM	FALSE	FALSE
ILMN_1689665	NAE1	FALSE	FALSE
ILMN_1803997	SDCCAG3	FALSE	FALSE
ILMN_1797055	LIN52	FALSE	FALSE
ILMN_1725642	SUMO3	FALSE	FALSE

ILMN_1661264	SHMT2	FALSE	FALSE
ILMN_1707720	SLC1A5	FALSE	FALSE
ILMN_2101526	GGCT	FALSE	FALSE
ILMN_1716553	KIF23	FALSE	FALSE
ILMN_2319825	ACYP1	FALSE	FALSE
ILMN_1797372	C3orf58	FALSE	FALSE
ILMN_1666739	RBM15	FALSE	FALSE
ILMN_1792076	TRERF1	FALSE	FALSE
ILMN_1774661	SNRPB	FALSE	FALSE
ILMN_2334042	THYN1	FALSE	FALSE
ILMN_1684321	CYB5B	FALSE	FALSE
ILMN_1777318	C9orf64	FALSE	FALSE
ILMN_1763390	ISL1	FALSE	FALSE
ILMN_1740005	SERPINF2	TRUE	FALSE
ILMN_1763326	SIMC1	FALSE	FALSE
ILMN_1727001	DDX46	FALSE	TRUE
ILMN_2315964	PSRC1	FALSE	FALSE
ILMN_1701512	KIAA0391	FALSE	FALSE
ILMN_1787879	ARL2	TRUE	FALSE
ILMN_1725121	XPO1	FALSE	FALSE
ILMN_1655622	PRKRIR	TRUE	FALSE
ILMN_1734702	MANEA	FALSE	FALSE
ILMN_1772743	PIGK	TRUE	FALSE
ILMN_1721081	SP4	FALSE	FALSE
ILMN_2225577	POC5	FALSE	FALSE
ILMN_1663113	TTLL12	FALSE	FALSE
ILMN_2046003	ZDHHC6	FALSE	FALSE
ILMN_1741406	HOOK1	FALSE	FALSE
ILMN_1712556	ZW10	FALSE	FALSE
ILMN_1743208	NEDD1	FALSE	FALSE
ILMN_1781516	SUPT16H	FALSE	FALSE
ILMN_2146372	KCTD6	FALSE	FALSE
ILMN_2345015	PTGES2	FALSE	FALSE
ILMN_1766499	HSPA2	FALSE	FALSE
ILMN_2082324	SLC36A4	TRUE	FALSE
ILMN_1805590	LSM8	FALSE	FALSE
ILMN_2358202	SRRT	FALSE	FALSE
ILMN_1701374	NUP35	TRUE	FALSE
ILMN_1723211	L2HGDH	FALSE	FALSE
ILMN_2333319	PTBP1	FALSE	FALSE
ILMN_1695491	WDYHV1	FALSE	FALSE
ILMN_1655165	RNF138	FALSE	FALSE
ILMN_1732705	HCFC1	FALSE	TRUE
ILMN_1658437	SFXN4	TRUE	FALSE
ILMN_1718069	MIS12	FALSE	TRUE
ILMN_2134765	NA	FALSE	FALSE

ILMN_1682404	SETMAR	FALSE	FALSE
ILMN_1670801	MTR	FALSE	FALSE
ILMN_2228938	ECM2	FALSE	FALSE
ILMN_1741585	SFMBT1	FALSE	TRUE
ILMN_1773964	H1FX	FALSE	FALSE
ILMN_1758827	RTN4IP1	TRUE	FALSE
ILMN_1770911	NRSN2	FALSE	TRUE
ILMN_2366972	NUDT6	FALSE	FALSE
ILMN_1761044	GNB1L	FALSE	FALSE
ILMN_1718718	MKKS	FALSE	FALSE
ILMN_1775192	BCLAF1	FALSE	FALSE
ILMN_1700685	S100PBP	FALSE	FALSE
ILMN_2175075	SRSF4	FALSE	FALSE
ILMN_1786379	SYNGR3	TRUE	FALSE
ILMN_2364535	SNUPN	FALSE	FALSE
ILMN_3272603	FAM60A	FALSE	FALSE
ILMN_1782688	THNSL1	FALSE	FALSE
ILMN_1755354	YBX2	FALSE	FALSE
ILMN_2412549	GAR1	FALSE	FALSE
ILMN_1742145	ESPL1	FALSE	FALSE
ILMN_1806123	MRPL23	FALSE	FALSE
ILMN_1686626	NA	FALSE	FALSE
ILMN_2072091	HNRNPUL2	FALSE	FALSE
ILMN_1654583	CHD1	FALSE	FALSE
ILMN_1756657	TRIM7	FALSE	FALSE
ILMN_2373689	DIAPH3	FALSE	FALSE
ILMN_1694479	WDR18	FALSE	FALSE
ILMN_1796210	PPRC1	FALSE	FALSE
ILMN_1719158	CTBP1	FALSE	FALSE
ILMN_2253648	PRKDC	TRUE	FALSE
ILMN_1670638	PITPNC1	FALSE	FALSE
ILMN_3306997	METTL1	TRUE	FALSE
ILMN_1741957	RABEPK	FALSE	FALSE
ILMN_1667417	RAB23	FALSE	FALSE
ILMN_1684563	SPIN4	FALSE	FALSE
ILMN_1710207	FAM178A	FALSE	FALSE
ILMN_2275803	LRRC45	FALSE	FALSE
ILMN_2381138	SEH1L	FALSE	FALSE
ILMN_1729144	EBAG9	FALSE	FALSE
ILMN_1748476	NOP58	TRUE	FALSE
ILMN_1711878	ENOPH1	FALSE	FALSE
ILMN_1765860	DOCK11	FALSE	TRUE
ILMN_1746158	HOXD11	FALSE	FALSE
ILMN_3236680	PPAT	FALSE	FALSE
ILMN_2343563	ANAPC11	FALSE	FALSE
ILMN_2346997	RAB23	FALSE	FALSE

ILMN_1787345	FKBP11	FALSE	FALSE
ILMN_1772957	FOXRED2	FALSE	FALSE
ILMN_1667239	INPP1	FALSE	FALSE
ILMN_3238269	NDUFA6	FALSE	TRUE
ILMN_1803775	NA	FALSE	FALSE
ILMN_1734194	EXOSC3	FALSE	FALSE
ILMN_1740493	TRAF5	FALSE	FALSE
ILMN_1769158	ISOC2	FALSE	TRUE
ILMN_3301824	TSEN15	FALSE	FALSE
ILMN_1694106	GPD1L	TRUE	FALSE
ILMN_1669928	ARHGEF16	FALSE	FALSE
ILMN_2054362	SLC25A40	TRUE	FALSE
ILMN_1766637	GLA	TRUE	FALSE
ILMN_1677452	REXO4	FALSE	TRUE
ILMN_3273047	POLR3G	TRUE	FALSE
ILMN_1732509	LRRC40	FALSE	FALSE
ILMN_1704842	ARL4A	TRUE	FALSE
ILMN_2180827	MEPCE	FALSE	FALSE
ILMN_3284119	NA	FALSE	FALSE
ILMN_1701753	HNRNPK	FALSE	FALSE
ILMN_1668498	CWF19L2	TRUE	FALSE
ILMN_1696087	PHB2	FALSE	FALSE
ILMN_1898682	PHIP	FALSE	FALSE
ILMN_1691966	NA	FALSE	FALSE
ILMN_1682572	C2CD5	FALSE	FALSE
ILMN_1705141	CACYBP	FALSE	FALSE
ILMN_2381397	HSPD1	FALSE	FALSE
ILMN_1670379	ANTXR1	FALSE	FALSE
ILMN_1756443	INHA	FALSE	FALSE
ILMN_1691444	UHRF2	FALSE	FALSE
ILMN_1750203	NA	FALSE	FALSE
ILMN_1688971	NOL11	FALSE	FALSE
ILMN_2400292	MAPK9	FALSE	FALSE
ILMN_1665865	IGFBP4	FALSE	FALSE
ILMN_1807807	SKA2	FALSE	FALSE
ILMN_2178226	NA	TRUE	FALSE
ILMN_1765044	CUTC	FALSE	FALSE
ILMN_1792305	ZNF318	FALSE	FALSE
ILMN_1656920	CRIP1	FALSE	FALSE
ILMN_2251375	ZFP64	FALSE	FALSE
ILMN_3289650	NA	FALSE	FALSE
ILMN_2335398	CECR5	FALSE	FALSE
ILMN_2119421	NA	FALSE	FALSE
ILMN_1770692	WDR12	TRUE	FALSE
ILMN_1659463	APAF1	FALSE	FALSE
ILMN_1796013	PYCR1	TRUE	FALSE

ILMN_2379734	CTBP1	FALSE	FALSE
ILMN_3194087	LINC00339	FALSE	TRUE
ILMN_1730304	NA	FALSE	FALSE
ILMN_1783253	NA	FALSE	FALSE
ILMN_2356311	SMIM11	FALSE	FALSE
ILMN_1797499	PRKDC	TRUE	FALSE
ILMN_1698996	SLC19A1	TRUE	FALSE
ILMN_1769545	PRPF19	FALSE	FALSE
ILMN_1699987	MANEA	FALSE	TRUE
ILMN_2323508	AIF1L	FALSE	FALSE
ILMN_1879857	NA	FALSE	FALSE
ILMN_1766435	WBP11	FALSE	FALSE
ILMN_1705151	SF3A3	FALSE	FALSE
ILMN_3279219	SNRPG	FALSE	FALSE
ILMN_2357272	BCLAF1	FALSE	FALSE
ILMN_1762426	DHFRL1	FALSE	FALSE
ILMN_3273854	HNRNPA2B1	FALSE	FALSE
ILMN_1740737	DCPS	FALSE	FALSE
ILMN_1769566	ATG3	FALSE	FALSE
ILMN_1786139	VKORC1	FALSE	FALSE
ILMN_1718734	MLLT6	FALSE	FALSE
ILMN_1672878	ABR	FALSE	FALSE
ILMN_1810235	ATP6V1E2	FALSE	FALSE
ILMN_1764321	ACOT4	FALSE	FALSE
ILMN_2327203	POT1	FALSE	FALSE
ILMN_1727553	ZBED8	FALSE	FALSE
ILMN_1725105	EEF1E1	FALSE	FALSE
ILMN_2224031	CETN3	FALSE	FALSE
ILMN_1712918	NQO2	FALSE	FALSE
ILMN_1809662	MZT1	FALSE	FALSE
ILMN_1695868	TOMM6	FALSE	FALSE
ILMN_1707088	DENND2D	FALSE	FALSE
ILMN_2413041	TEAD4	FALSE	FALSE
ILMN_1733932	SNUPN	FALSE	FALSE
ILMN_1708627	SRP9	FALSE	FALSE
ILMN_2189424	MRPL20	FALSE	FALSE
ILMN_1800855	PPTC7	FALSE	FALSE
ILMN_1707169	AGMAT	FALSE	FALSE
ILMN_1750167	PRR3	FALSE	FALSE
ILMN_1733164	NA	FALSE	FALSE
ILMN_1662318	CCDC59	FALSE	FALSE
ILMN_3243011	SDHD	FALSE	FALSE
ILMN_3262936	LCLAT1	TRUE	FALSE
ILMN_1779399	SNRPA	FALSE	FALSE
ILMN_1791296	DPY19L1	FALSE	FALSE
ILMN_1805481	TRMT11	TRUE	FALSE

ILMN_3275936	NA	FALSE	FALSE
ILMN_1763264	MRPL2	FALSE	FALSE
ILMN_1713178	DENND6A	FALSE	FALSE
ILMN_1726030	GPX7	FALSE	FALSE
ILMN_1767475	CERK	FALSE	FALSE
ILMN_1745806	PEMT	FALSE	FALSE
ILMN_1762787	RNF26	FALSE	FALSE
ILMN_2352609	OGG1	FALSE	FALSE
ILMN_1656016	DHX9	FALSE	FALSE
ILMN_2372379	MGA	FALSE	FALSE
ILMN_1749253	TUBD1	FALSE	FALSE
ILMN_1708881	RAB20	FALSE	FALSE
ILMN_1711414	MRPS27	FALSE	FALSE
ILMN_2317618	TCF19	FALSE	FALSE
ILMN_1703279	CXorf57	FALSE	FALSE
ILMN_1784292	ANKMY2	FALSE	FALSE
ILMN_1797074	EMG1	FALSE	FALSE
ILMN_1653896	ATRIP	FALSE	TRUE
ILMN_1728626	WDR5	FALSE	TRUE
ILMN_1723235	DUS3L	FALSE	FALSE
ILMN_1799280	BDH1	FALSE	FALSE
ILMN_1669584	ILF3	FALSE	FALSE
ILMN_2234229	PRMT6	FALSE	FALSE
ILMN_1753286	MYO19	FALSE	FALSE
ILMN_1692517	SORD	FALSE	FALSE
ILMN_2393573	RASSF1	FALSE	FALSE
ILMN_1676528	BTN3A2	FALSE	FALSE
ILMN_1729417	GNE	FALSE	FALSE
ILMN_1775085	ZNF232	FALSE	FALSE
ILMN_1769451	ILVBL	TRUE	FALSE
ILMN_1775573	DPY19L4	FALSE	FALSE
ILMN_2041327	MRPL37	FALSE	FALSE
ILMN_3235397	SNORD110	FALSE	FALSE
ILMN_1735367	HSD17B11	FALSE	FALSE
ILMN_1719646	ZIK1	FALSE	FALSE
ILMN_1726986	AADAT	FALSE	FALSE
ILMN_2413330	TMEM107	FALSE	FALSE
ILMN_3263702	VPS9D1-AS1	TRUE	FALSE
ILMN_1726391	MRPL39	FALSE	FALSE
ILMN_1680091	POP7	FALSE	FALSE
ILMN_1786326	KCTD15	FALSE	FALSE
ILMN_1736234	CHTOP	TRUE	TRUE
ILMN_3289346	NA	FALSE	FALSE
ILMN_1698189	AASDHPPT	TRUE	FALSE
ILMN_2076640	KHDRBS1	FALSE	FALSE
ILMN_3244893	BAG2	FALSE	FALSE

ILMN_2121437	NCL	FALSE	FALSE
ILMN_1815107	MATR3	FALSE	FALSE
ILMN_1679995	MPP6	FALSE	FALSE
ILMN_1673773	GALNT18	FALSE	FALSE
ILMN_3264828	METTL15	FALSE	FALSE
ILMN_1714965	NFKB1	FALSE	FALSE
ILMN_2363361	SFXN4	FALSE	FALSE
ILMN_1741736	DDX28	TRUE	TRUE
ILMN_1772302	NA	FALSE	FALSE
ILMN_2092850	HPSE	FALSE	FALSE
ILMN_2144401	GLB1L2	FALSE	FALSE
ILMN_1667551	NA	FALSE	FALSE
ILMN_2086238	SMYD4	FALSE	FALSE
ILMN_1718830	ICE2	FALSE	FALSE
ILMN_1767892	DUSP12	FALSE	FALSE
ILMN_3240022	SNORA73A	FALSE	FALSE
ILMN_1723874	MRPS6	FALSE	FALSE
ILMN_1774028	MTFR1	FALSE	FALSE
ILMN_1663002	STOML2	FALSE	FALSE
ILMN_1810486	RAB34	FALSE	FALSE
ILMN_1763627	TNPO1	FALSE	FALSE
ILMN_1727281	TAF6L	TRUE	FALSE
ILMN_1735735	KATNB1	FALSE	FALSE
ILMN_1691156	MT1A	FALSE	FALSE
ILMN_1737413	MSH2	TRUE	FALSE
ILMN_1746252	POLD2	TRUE	FALSE
ILMN_1730698	ODF2	FALSE	FALSE
ILMN_2359627	BCL2L11	FALSE	FALSE
ILMN_1674694	LINC00669	FALSE	FALSE
ILMN_1764127	NA	FALSE	FALSE
ILMN_1697665	CBX3	FALSE	FALSE
ILMN_1774974	CLUAP1	FALSE	FALSE
ILMN_1739325	LOC284023	FALSE	FALSE
ILMN_1692545	NPM1	FALSE	FALSE
ILMN_1778478	CCDC28B	FALSE	FALSE
ILMN_3301042	FLVCR1-AS1	FALSE	FALSE
ILMN_1708006	MICB	FALSE	FALSE
ILMN_1686319	USP37	FALSE	FALSE
ILMN_2286106	PAICS	FALSE	FALSE
ILMN_1726064	PAK1IP1	FALSE	FALSE
ILMN_1715179	SNRPA1	FALSE	FALSE
ILMN_1787808	CEP63	FALSE	FALSE
ILMN_2140207	NA	FALSE	FALSE
ILMN_1696407	SRSF2	FALSE	FALSE
ILMN_1766408	CBFB	FALSE	FALSE
ILMN_1703949	KPNB1	TRUE	FALSE

ILMN_3186473	LCLAT1	FALSE	FALSE
ILMN_1691165	CLSPN	FALSE	FALSE
ILMN_1676191	DARS2	FALSE	FALSE
ILMN_2370907	RAD51D	FALSE	FALSE
ILMN_1793966	BOLA1	TRUE	FALSE
ILMN_1707484	GEMIN6	FALSE	FALSE
ILMN_1669142	NAA15	FALSE	TRUE
ILMN_3207233	ANP32B	FALSE	FALSE
ILMN_2231242	HMGB1	FALSE	FALSE
ILMN_1680618	MYC	FALSE	FALSE
ILMN_1751393	ZNF684	FALSE	FALSE
ILMN_1739032	TMEM70	FALSE	FALSE
ILMN_1678423	SPA17	FALSE	FALSE
ILMN_1752947	COPRS	FALSE	FALSE
ILMN_1665483	KIAA0020	FALSE	FALSE
ILMN_1723358	SCARA3	FALSE	FALSE
ILMN_3235326	SNHG17	FALSE	FALSE
ILMN_3284063	VDAC3	FALSE	FALSE
ILMN_1656691	FBXO4	FALSE	FALSE
ILMN_1741477	SMAD4	FALSE	FALSE
ILMN_1856634	NFIA	TRUE	FALSE
ILMN_1758548	NEK7	FALSE	FALSE
ILMN_3181420	HMGXB4	FALSE	FALSE
ILMN_1694539	MAP3K6	FALSE	FALSE
ILMN_1685824	B4GALT5	FALSE	FALSE
ILMN_2143487	TAF1B	FALSE	FALSE
ILMN_1757415	COA7	TRUE	FALSE
ILMN_1727855	PEMT	FALSE	FALSE
ILMN_1679754	ADRA1B	FALSE	FALSE
ILMN_1708936	EXOSC3	FALSE	FALSE
ILMN_1755811	FBXO4	FALSE	FALSE
ILMN_1664602	NA	FALSE	FALSE
ILMN_1769634	STRA13	TRUE	FALSE
ILMN_1815141	MTR	FALSE	FALSE
ILMN_2301722	PDE8B	FALSE	FALSE
ILMN_1697491	PRR5L	FALSE	FALSE
ILMN_1657087	ZNF385B	FALSE	FALSE
ILMN_1811006	E2F8	FALSE	FALSE
ILMN_1712505	KDELC1	FALSE	FALSE
ILMN_1759991	NOL12	FALSE	TRUE
ILMN_1669722	LRRC61	FALSE	FALSE
ILMN_1759008	ZNF689	FALSE	FALSE
ILMN_1708081	LCLAT1	FALSE	FALSE
ILMN_1761411	MCMBP	FALSE	FALSE
ILMN_3236825	RAPGEF5	FALSE	TRUE
ILMN_2412927	GMPPB	FALSE	FALSE

ILMN_1656274	PRPF38A	FALSE	FALSE
ILMN_2242937	ARSB	FALSE	FALSE
ILMN_2363668	YIF1B	FALSE	FALSE
ILMN_1654671	SLMO1	FALSE	FALSE
ILMN_1658460	SRSF10	FALSE	FALSE
ILMN_1701711	BRD8	FALSE	FALSE
ILMN_3238797	FAM72A	TRUE	FALSE
ILMN_1731648	FOXJ2	FALSE	FALSE
ILMN_1673026	CHCHD3	FALSE	FALSE
ILMN_1721921	BLMH	FALSE	FALSE
ILMN_3210491	TOMM40	FALSE	FALSE
ILMN_1690268	HNRNPUL1	FALSE	TRUE
ILMN_3287583	UBB	FALSE	FALSE
ILMN_1804277	SPRED1	FALSE	FALSE
ILMN_1779584	UTP18	FALSE	FALSE
ILMN_3208140	NA	FALSE	FALSE
ILMN_2116594	GPR89B	FALSE	FALSE
ILMN_1739798	MALSU1	FALSE	FALSE
ILMN_1808374	SNTB2	FALSE	FALSE
ILMN_2278235	CTBP1	FALSE	FALSE
ILMN_3235104	KIAA1731	FALSE	FALSE
ILMN_1660754	MIR17HG	FALSE	FALSE
ILMN_1767142	ZNF280D	FALSE	FALSE
ILMN_2411116	FGFR1OP	TRUE	FALSE
ILMN_1730940	KLHDC3	FALSE	FALSE
ILMN_1801378	COQ3	TRUE	FALSE
ILMN_1691798	ZNF26	FALSE	FALSE
ILMN_1722066	ARMC1	FALSE	FALSE
ILMN_2380999	RECQL	FALSE	FALSE
ILMN_1721029	RHEBL1	FALSE	FALSE
ILMN_1754858	PASK	FALSE	FALSE
ILMN_1698209	AGPS	TRUE	FALSE
ILMN_1670542	AK2	TRUE	FALSE
ILMN_1708537	RBPJ	FALSE	FALSE
ILMN_1724986	WDR53	FALSE	FALSE
ILMN_2209515	SNORD56	FALSE	FALSE
ILMN_3259509	C11orf86	FALSE	FALSE
ILMN_2194649	TADA1	FALSE	FALSE
ILMN_1736623	NCKIPSD	FALSE	FALSE
ILMN_1666670	RBX1	FALSE	FALSE
ILMN_1784709	GNPDA1	FALSE	FALSE
ILMN_1749096	BCL2L10	TRUE	FALSE
ILMN_1680193	PAXIP1	FALSE	FALSE
ILMN_1751589	NUDCD2	FALSE	FALSE
ILMN_1697440	PRPF4	FALSE	FALSE
ILMN_2226955	VOPP1	FALSE	FALSE

ILMN_3268880	LINC00263	FALSE	FALSE
ILMN_2215119	SYNJ2	FALSE	FALSE
ILMN_1676302	PCED1A	FALSE	FALSE
ILMN_2409793	MAZ	FALSE	FALSE
ILMN_1806432	NT5C	FALSE	FALSE
ILMN_1655504	IFT81	FALSE	FALSE
ILMN_1705737	IMPDH2	FALSE	FALSE
ILMN_3251232	HMGN2	FALSE	FALSE
ILMN_1741133	NME1	TRUE	FALSE
ILMN_1744308	DHX33	TRUE	FALSE
ILMN_1673369	SEPHS1	FALSE	FALSE
ILMN_2328972	DNMT3B	FALSE	FALSE
ILMN_2099594	SRP9	FALSE	FALSE
ILMN_1668179	HNRNPF	FALSE	FALSE
ILMN_1685781	C14orf142	FALSE	FALSE
ILMN_1687201	APOL6	FALSE	FALSE
ILMN_1659166	SLC6A15	FALSE	FALSE
ILMN_1689972	RRP9	FALSE	FALSE
ILMN_1726840	B3GALNT1	FALSE	FALSE
ILMN_1675626	PRPF38A	FALSE	FALSE
ILMN_1690576	NA	FALSE	FALSE
ILMN_1661399	ARL4A	FALSE	FALSE
ILMN_1755911	ING5	TRUE	FALSE
ILMN_1697024	STYX	FALSE	FALSE
ILMN_1652749	ERF	FALSE	FALSE
ILMN_2214278	ANKRD32	FALSE	FALSE
ILMN_1756793	PAPD7	FALSE	FALSE
ILMN_1790650	FOPNL	FALSE	FALSE
ILMN_3247018	SNORA67	FALSE	TRUE
ILMN_1780298	FAM86A	FALSE	FALSE
ILMN_1662617	PPP2R3C	FALSE	FALSE
ILMN_1760933	PEX10	TRUE	FALSE
ILMN_3181328	NA	FALSE	FALSE
ILMN_1812545	DMC1	FALSE	FALSE
ILMN_1750051	AMER1	FALSE	TRUE
ILMN_2173611	MT1E	FALSE	TRUE
ILMN_1660063	POLE4	FALSE	FALSE
ILMN_2138435	MRPS27	FALSE	FALSE
ILMN_1683059	SIRT5	FALSE	TRUE
ILMN_1665859	RAB27A	FALSE	FALSE
ILMN_3210917	SET	FALSE	FALSE
ILMN_1730523	FAM195A	TRUE	FALSE
ILMN_3202863	Sep-10	FALSE	FALSE
ILMN_1668960	MID1IP1	FALSE	FALSE
ILMN_1723117	IPO9	FALSE	FALSE
ILMN_3206827	HNRNPA3	FALSE	FALSE

ILMN_2316104	IQCB1	FALSE	FALSE
ILMN_3235472	WDYHV1	FALSE	FALSE
ILMN_1732566	TMTC3	FALSE	FALSE
ILMN_1812973	RRP1B	FALSE	FALSE
ILMN_1666727	ZNF586	FALSE	TRUE
ILMN_2205245	GPN2	FALSE	FALSE
ILMN_1765770	SYCP2	FALSE	TRUE
ILMN_1690138	PHF10	TRUE	FALSE
ILMN_1733045	RAB36	FALSE	FALSE
ILMN_1696330	GUF1	FALSE	FALSE
ILMN_1785831	ZDHHC13	FALSE	FALSE
ILMN_2343010	BOLA3	FALSE	FALSE
ILMN_2054442	ZNF146	FALSE	FALSE
ILMN_1739083	SIRT1	TRUE	FALSE
ILMN_2332105	WRNIP1	FALSE	FALSE
ILMN_1716053	AK2	FALSE	FALSE
ILMN_1796407	SS18L2	FALSE	FALSE
ILMN_3239861	SKIDA1	FALSE	FALSE
ILMN_1660938	TOE1	FALSE	FALSE
ILMN_1701933	SNCA	FALSE	FALSE
ILMN_1815346	TMEM136	FALSE	FALSE
ILMN_2341952	MRPL35	TRUE	FALSE
ILMN_1742031	IFRD2	FALSE	FALSE
ILMN_3236061	ZNF783	FALSE	TRUE
ILMN_1654289	ELK1	FALSE	TRUE
ILMN_1810327	HNRNPUL2	FALSE	FALSE
ILMN_1657993	ADNP	FALSE	FALSE
ILMN_1656682	AZIN1	FALSE	FALSE
ILMN_3238845	SMIM11	FALSE	FALSE
ILMN_1904785	NA	FALSE	FALSE
ILMN_1682747	FANCA	FALSE	FALSE
ILMN_1665290	NA	FALSE	FALSE
ILMN_2356895	MRPL42	FALSE	FALSE
ILMN_1740861	DTWD1	FALSE	FALSE
ILMN_3207933	ABCE1	TRUE	FALSE
ILMN_1752270	SLC25A40	TRUE	FALSE
ILMN_2352326	COASY	FALSE	FALSE
ILMN_3307683	ZNF695	FALSE	FALSE
ILMN_1784847	CREBZF	TRUE	FALSE
ILMN_1726222	FLOT2	FALSE	FALSE
ILMN_1667405	ARHGEF39	FALSE	FALSE
ILMN_1735438	GPM6B	FALSE	FALSE
ILMN_1748916	TIMM21	TRUE	FALSE
ILMN_2389528	MTL5	TRUE	FALSE
ILMN_1738572	USP48	FALSE	FALSE
ILMN_1790757	ADSL	FALSE	FALSE

ILMN_1718295	STAC2	FALSE	FALSE
ILMN_1651684	KIAA0586	FALSE	FALSE
ILMN_1751403	NUDT15	FALSE	FALSE
ILMN_1789510	STIP1	TRUE	FALSE
ILMN_1744647	CAND1	FALSE	FALSE
ILMN_1810992	CAD	FALSE	FALSE
ILMN_1777765	C12orf10	FALSE	FALSE
ILMN_1662483	NA	FALSE	FALSE
ILMN_1772482	SLC6A10P	FALSE	FALSE
ILMN_1683635	C11orf70	FALSE	TRUE
ILMN_1782257	METTL17	FALSE	FALSE
ILMN_2410772	KEAP1	FALSE	TRUE
ILMN_1748034	KLHDC4	FALSE	FALSE
ILMN_2354649	SRSF10	TRUE	FALSE
ILMN_3290800	SDHD	FALSE	FALSE
ILMN_1657332	CCDC85B	FALSE	FALSE
ILMN_1806487	FAM109B	FALSE	FALSE
ILMN_1815882	HNRNPA1	TRUE	FALSE
ILMN_2189842	SNORA10	FALSE	FALSE
ILMN_2153916	HSPA2	FALSE	FALSE
ILMN_1697802	SLC25A33	FALSE	FALSE
ILMN_1671791	PCK2	TRUE	FALSE
ILMN_1756862	APOL3	FALSE	FALSE
ILMN_1718607	TSPAN4	FALSE	FALSE
ILMN_1801262	RAD51B	FALSE	FALSE
ILMN_1657873	XPO4	FALSE	FALSE
ILMN_2322935	MAPKAPK5	TRUE	FALSE
ILMN_1733396	CDC25A	FALSE	FALSE
ILMN_2243697	ZDHHC13	FALSE	FALSE
ILMN_1738712	GPR180	FALSE	FALSE
ILMN_1746819	C5	FALSE	FALSE
ILMN_1730351	TEX19	FALSE	FALSE
ILMN_1664646	NSUN6	FALSE	FALSE
ILMN_1709257	RIPPLY3	FALSE	FALSE
ILMN_1663751	CYCS	FALSE	FALSE
ILMN_1728163	CTDSP1	FALSE	FALSE
ILMN_1672660	MBP	FALSE	FALSE
ILMN_1744795	TBL1X	FALSE	FALSE
ILMN_1712530	AKAP1	TRUE	FALSE
ILMN_1770020	PPIL3	FALSE	FALSE
ILMN_1656248	CLMP	FALSE	FALSE
ILMN_1804610	NFX1	FALSE	FALSE
ILMN_1671288	ODF2	FALSE	FALSE
ILMN_2251978	PAXBP1	FALSE	FALSE
ILMN_1797332	NARS2	TRUE	FALSE
ILMN_1691487	TRAF2	FALSE	FALSE

ILMN_2373177	PANK2	FALSE	FALSE
ILMN_1762436	UBB	FALSE	FALSE
ILMN_1657884	NME2	FALSE	FALSE
ILMN_1671257	DKC1	TRUE	FALSE
ILMN_2128293	APIP	FALSE	FALSE
ILMN_1704305	NIP7	FALSE	FALSE
ILMN_2065822	MCM3AP-AS1	FALSE	FALSE
ILMN_2083833	CNOT6L	FALSE	FALSE
ILMN_1803376	AEBP2	FALSE	FALSE
ILMN_1700923	ATG4C	FALSE	FALSE
ILMN_2166686	NUFIP1	FALSE	TRUE
ILMN_1780806	ANKRD36B	FALSE	FALSE
ILMN_2120965	NPAT	FALSE	FALSE
ILMN_2341690	ELP5	FALSE	FALSE
ILMN_2056002	ANP32AP1	FALSE	FALSE
ILMN_1791593	DENND5B	FALSE	FALSE
ILMN_1749081	AUTS2	FALSE	FALSE
ILMN_1667932	ANKRD36B	FALSE	FALSE
ILMN_3235113	TOMM6	FALSE	FALSE
ILMN_2274923	MOCS2	FALSE	FALSE
ILMN_1716089	KANK2	FALSE	FALSE
ILMN_1811997	RNF115	FALSE	FALSE
ILMN_1659682	GALK2	FALSE	FALSE
ILMN_1789828	VPS13A	FALSE	FALSE
ILMN_2342240	MGAT2	FALSE	FALSE
ILMN_1738784	PPP2R5A	FALSE	FALSE
ILMN_1747195	PSMB8	FALSE	FALSE
ILMN_1712985	C17orf58	TRUE	FALSE
ILMN_3290380	XRCC6	FALSE	FALSE
ILMN_1712257	PPP2R3B	FALSE	FALSE
ILMN_3241139	SNORD57	FALSE	FALSE
ILMN_1706901	HOXD13	FALSE	FALSE
ILMN_2052871	TMEM116	FALSE	FALSE
ILMN_1752639	SLC25A24	FALSE	FALSE
ILMN_1740269	WNT2B	FALSE	FALSE
ILMN_1805111	BRWD1	FALSE	FALSE
ILMN_1655403	ERI2	FALSE	FALSE
ILMN_1729976	CHAMP1	FALSE	FALSE
ILMN_1669674	CNPY3	FALSE	FALSE
ILMN_2393296	GK	FALSE	FALSE
ILMN_1741398	TTI1	FALSE	TRUE
ILMN_2140510	KLHL13	FALSE	FALSE
ILMN_3239217	TOMM40	FALSE	TRUE
ILMN_1730999	TPR	FALSE	FALSE
ILMN_1856315	NA	FALSE	FALSE
ILMN_1749752	GAR1	FALSE	FALSE

ILMN_1655154	PTBP1	FALSE	FALSE
ILMN_1713088	MSI2	FALSE	FALSE
ILMN_1789007	APOC1	FALSE	FALSE
ILMN_2211003	GAS2L3	FALSE	FALSE
ILMN_1810127	ZNF789	FALSE	FALSE
ILMN_1712708	TRIM47	FALSE	FALSE
ILMN_1783681	MRPL34	FALSE	FALSE
ILMN_1695226	DIAPH3	FALSE	FALSE
ILMN_1679209	HSPA9	FALSE	FALSE
ILMN_3251526	USP13	FALSE	FALSE
ILMN_3199609	TCP1	FALSE	FALSE
ILMN_2296422	SMC2	FALSE	FALSE
ILMN_1787595	B3GALNT1	FALSE	FALSE
ILMN_2325394	NA	TRUE	FALSE
ILMN_2390299	PSMB8	FALSE	FALSE
ILMN_2069446	SAFB2	FALSE	FALSE
ILMN_1652753	PAAF1	FALSE	FALSE
ILMN_1766154	MRPL30	FALSE	FALSE
ILMN_1743097	XRCC6	FALSE	FALSE
ILMN_1663220	MRPL22	FALSE	FALSE
ILMN_1673991	ATIC	TRUE	FALSE
ILMN_2163070	KHDC1	FALSE	FALSE
ILMN_3248773	SNHG15	FALSE	FALSE
ILMN_1742238	SET	FALSE	FALSE
ILMN_1738796	PITPNC1	FALSE	FALSE
ILMN_1748254	ZNF714	FALSE	FALSE
ILMN_2330170	MYBPC1	FALSE	FALSE
ILMN_1786015	CTCF	FALSE	FALSE
ILMN_3250592	FAM86A	FALSE	FALSE
ILMN_1791949	PGBD1	FALSE	FALSE
ILMN_2350607	NDUFAF5	FALSE	FALSE
ILMN_2166506	XRCC6	FALSE	FALSE
ILMN_1751143	TMEM243	FALSE	FALSE
ILMN_1707634	CMC1	FALSE	FALSE
ILMN_2206716	JTB	FALSE	FALSE
ILMN_1780292	NA	TRUE	FALSE
ILMN_2196097	PPP2CA	FALSE	FALSE
ILMN_1718033	LYPD5	FALSE	FALSE
ILMN_1716596	NSMAF	FALSE	FALSE
ILMN_1803317	C9orf114	FALSE	FALSE
ILMN_3179371	HNRNPK	FALSE	FALSE
ILMN_2371147	BCL7C	FALSE	FALSE
ILMN_1716862	PPM1B	FALSE	FALSE
ILMN_1664367	COQ6	FALSE	FALSE
ILMN_1686664	MT2A	FALSE	FALSE
ILMN_3247906	RNF114	TRUE	TRUE

ILMN_1762224	HPS3	FALSE	FALSE
ILMN_1775901	PHF5A	FALSE	FALSE
ILMN_1779855	HSD17B6	FALSE	FALSE
ILMN_1705774	TIGD5	FALSE	FALSE
ILMN_1762095	TMTC4	FALSE	FALSE
ILMN_1746393	TSEN2	FALSE	FALSE
ILMN_2383964	CDCA4	FALSE	FALSE
ILMN_1774589	IQCC	FALSE	TRUE
ILMN_1803312	DIMT1	TRUE	FALSE
ILMN_2092756	TMEM109	FALSE	FALSE
ILMN_2369403	PALM2	FALSE	FALSE
ILMN_1682896	PAXBP1	FALSE	FALSE
ILMN_1804737	RAVER2	FALSE	FALSE
ILMN_1773968	SERBP1	FALSE	FALSE
ILMN_1714428	FBXO17	FALSE	FALSE
ILMN_1687724	RAP1GDS1	FALSE	FALSE
ILMN_1680782	PATL1	FALSE	FALSE
ILMN_1669484	WDR6	FALSE	TRUE
ILMN_2398926	C17orf58	TRUE	FALSE
ILMN_1731518	PLD6	FALSE	FALSE
ILMN_1680850	RECQL	FALSE	FALSE
ILMN_1715886	CNOT7	FALSE	FALSE
ILMN_1664641	MED4	FALSE	FALSE
ILMN_1722059	SAFB	FALSE	FALSE
ILMN_3304200	ZNF385B	FALSE	FALSE
ILMN_1768393	SNRPD1	FALSE	FALSE
ILMN_1792173	TUBGCP4	FALSE	FALSE
ILMN_2082209	TOX2	FALSE	FALSE
ILMN_1680134	CARM1	FALSE	FALSE
ILMN_2216918	SHPK	FALSE	FALSE
ILMN_1801246	IFITM1	FALSE	FALSE
ILMN_1696510	GPR89B	FALSE	FALSE
ILMN_1703430	CCDC94	FALSE	FALSE
ILMN_2345016	PTGES2	FALSE	FALSE
ILMN_2346831	MGAT2	FALSE	FALSE
ILMN_3245707	RIMKLB	FALSE	FALSE
ILMN_1812580	YDJC	FALSE	FALSE
ILMN_1691181	TMX1	FALSE	FALSE
ILMN_1806867	PPM1G	FALSE	FALSE
ILMN_1767747	HDAC2	TRUE	FALSE
ILMN_1670821	TXLNGY	TRUE	FALSE
ILMN_1680220	GEN1	FALSE	FALSE
ILMN_1703015	ZRANB2	FALSE	FALSE
ILMN_1734833	NBN	FALSE	FALSE
ILMN_1704619	VPS29	FALSE	FALSE
ILMN_1764410	GUCD1	FALSE	FALSE

ILMN_1785914	CDC6	FALSE	FALSE
ILMN_2397627	UPF3B	FALSE	FALSE
ILMN_1665730	ABCB10	FALSE	FALSE
ILMN_1682197	NFXL1	TRUE	FALSE
ILMN_2282959	DNA2	FALSE	FALSE
ILMN_1662049	AGPAT5	FALSE	FALSE
ILMN_1803652	C9orf91	FALSE	FALSE
ILMN_1749210	BUD13	FALSE	FALSE
ILMN_1815043	MRPS2	FALSE	FALSE
ILMN_1697670	SRRM1	FALSE	FALSE
ILMN_1756942	SP3	FALSE	FALSE
ILMN_1736568	CASP2	FALSE	FALSE
ILMN_1675462	LSM6	FALSE	FALSE
ILMN_1676946	AP3M2	FALSE	FALSE
ILMN_1770031	ABHD10	FALSE	FALSE
ILMN_1690386	NA	FALSE	FALSE
ILMN_1774086	CBX3	FALSE	FALSE
ILMN_1740819	STARD7	FALSE	FALSE
ILMN_2324998	NGDN	FALSE	FALSE
ILMN_1757627	ZMYND19	FALSE	FALSE
ILMN_1728132	LDHB	FALSE	FALSE
ILMN_2051867	PTCD2	FALSE	FALSE
ILMN_1677723	ANGPT1	FALSE	FALSE
ILMN_2336130	NA	FALSE	FALSE
ILMN_1691430	GSTCD	FALSE	FALSE
ILMN_2214603	PPP2R3C	FALSE	FALSE
ILMN_2346460	ICE2	FALSE	FALSE
ILMN_1689336	HOXA10	FALSE	FALSE
ILMN_1803853	NOL7	FALSE	FALSE
ILMN_1780756	RBM23	FALSE	FALSE
ILMN_1665775	Mar-02	TRUE	FALSE
ILMN_1653129	CSTF2	FALSE	FALSE
ILMN_2054392	PPIL1	FALSE	FALSE
ILMN_1698512	NA	FALSE	FALSE
ILMN_1718706	ERAL1	FALSE	FALSE
ILMN_2115434	RAB32	FALSE	FALSE
ILMN_1736130	RBFA	TRUE	FALSE
ILMN_1701855	PPP1CC	FALSE	FALSE
ILMN_1675131	PIH1D1	FALSE	FALSE
ILMN_2186925	FOXE1	FALSE	FALSE
ILMN_1795856	ELF2	FALSE	FALSE
ILMN_1691843	RNPS1	FALSE	FALSE
ILMN_2301163	SFR1	FALSE	FALSE
ILMN_1714809	RPIA	FALSE	FALSE
ILMN_2413158	PODXL	FALSE	TRUE
ILMN_3285198	SET	FALSE	FALSE

ILMN_1698487	SDHD	FALSE	FALSE
ILMN_1669901	NA	FALSE	FALSE
ILMN_1763404	SRP9	FALSE	FALSE
ILMN_3270626	NA	FALSE	FALSE
ILMN_1688621	INIP	FALSE	FALSE
ILMN_1731165	SLC16A8	FALSE	TRUE
ILMN_1756220	DDX18	TRUE	FALSE
ILMN_2133534	NA	FALSE	FALSE
ILMN_3242038	GPX8	FALSE	FALSE
ILMN_1706652	OGG1	FALSE	FALSE
ILMN_2388425	EXTL2	FALSE	FALSE
ILMN_1727043	COLGALT1	FALSE	FALSE
ILMN_3240997	ARAP3	FALSE	FALSE
ILMN_1761479	ZC3HC1	FALSE	FALSE
ILMN_3239168	FAM86C2P	FALSE	FALSE
ILMN_1783168	NETO1	FALSE	FALSE
ILMN_1666634	FAF1	FALSE	FALSE
ILMN_3228700	POLD2	TRUE	FALSE
ILMN_1679891	NAF1	FALSE	FALSE
ILMN_3247608	ABCB10	FALSE	FALSE
ILMN_1689445	POLR2H	FALSE	FALSE
ILMN_1670796	EXOSC10	TRUE	FALSE
ILMN_1685112	TACO1	FALSE	FALSE
ILMN_2398587	ZNRD1	FALSE	FALSE
ILMN_3176790	UBXN2B	FALSE	FALSE
ILMN_1758090	BCCIP	FALSE	FALSE
ILMN_1735055	TEAD1	FALSE	FALSE
ILMN_2127605	LRP3	FALSE	FALSE
ILMN_2414014	RBM10	FALSE	FALSE
ILMN_2120340	RUVBL2	FALSE	TRUE
ILMN_3269405	HNRNPM	FALSE	FALSE
ILMN_1815933	FTSJ2	FALSE	FALSE
ILMN_2219351	CENPO	FALSE	FALSE
ILMN_1806106	GNL3	FALSE	TRUE
ILMN_1742147	UBL4A	FALSE	FALSE
ILMN_1670948	TSR2	TRUE	TRUE
ILMN_3238511	HCG11	FALSE	FALSE
ILMN_2330267	ABCE1	FALSE	FALSE
ILMN_2049727	OSER1	FALSE	FALSE
ILMN_1711314	NUDT5	TRUE	FALSE
ILMN_1898124	SNHG15	FALSE	FALSE
ILMN_1681324	MRPL49	FALSE	FALSE
ILMN_2346727	MTUS1	FALSE	TRUE
ILMN_1711492	PTGES3	FALSE	FALSE
ILMN_1771326	VWA9	FALSE	FALSE
ILMN_2085446	ZNF670	FALSE	TRUE

ILMN_1680643	G2E3	FALSE	FALSE
ILMN_2078334	CNOT10	FALSE	FALSE
ILMN_1730794	SERTAD4	FALSE	FALSE
ILMN_1655382	DCLRE1B	FALSE	FALSE
ILMN_3299905	RNFT2	FALSE	FALSE
ILMN_1693045	TMED1	FALSE	FALSE
ILMN_1787762	HEATR1	FALSE	FALSE
ILMN_1659156	ANKRD16	TRUE	FALSE
ILMN_1761131	ECI2	TRUE	FALSE
ILMN_1704672	NABP2	FALSE	FALSE
ILMN_3238613	SNORA26	FALSE	FALSE
ILMN_1684663	ZDHHC13	FALSE	FALSE
ILMN_1764082	MBOAT1	FALSE	FALSE
ILMN_1657509	TSEN54	FALSE	FALSE
ILMN_1724009	SETD6	TRUE	FALSE
ILMN_1730957	NA	FALSE	FALSE
ILMN_1698139	TCEA1	FALSE	FALSE
ILMN_1722390	CHRAC1	FALSE	FALSE
ILMN_1671693	TPGS2	FALSE	FALSE
ILMN_2140799	FAM24B	FALSE	FALSE
ILMN_1685856	FAM92A1	FALSE	FALSE
ILMN_1691294	CTBP2	FALSE	FALSE
ILMN_1776653	SCML1	FALSE	FALSE
ILMN_1730630	CXorf56	FALSE	FALSE
ILMN_1737087	WDR5	FALSE	FALSE
ILMN_2383707	ALDH1A2	FALSE	FALSE
ILMN_2136635	ISCA2	FALSE	FALSE
ILMN_2165473	MID1IP1	FALSE	FALSE
ILMN_1652123	HMGN1	FALSE	FALSE
ILMN_2082762	SNORD68	FALSE	FALSE
ILMN_1654141	ZDHHC20	FALSE	FALSE
ILMN_1808238	RBPMS2	FALSE	FALSE
ILMN_1657283	ALKBH5	FALSE	FALSE
ILMN_2313158	MBNL1	FALSE	FALSE
ILMN_1808768	ROCK1	FALSE	FALSE
ILMN_1687922	RP9	FALSE	FALSE
ILMN_2410864	RAB28	FALSE	FALSE
ILMN_1782954	UBE2K	TRUE	FALSE
ILMN_1761594	FAM35A	FALSE	FALSE
ILMN_2266005	SMIM11	FALSE	FALSE
ILMN_1659620	NA	FALSE	FALSE
ILMN_1777526	MED20	FALSE	FALSE
ILMN_1694504	RNF220	FALSE	TRUE
ILMN_1660582	LIG3	FALSE	FALSE
ILMN_1773645	GMPPB	FALSE	FALSE
ILMN_3240781	SNORD17	FALSE	FALSE

ILMN_1658110	FAM210A	TRUE	FALSE
ILMN_1755352	TUB	FALSE	TRUE
ILMN_1789558	ZC2HC1A	FALSE	FALSE
ILMN_3238712	SNRNP25	FALSE	FALSE
ILMN_1651506	TGS1	FALSE	FALSE
ILMN_1712357	HNRNPK	FALSE	FALSE
ILMN_3208881	SETD8	FALSE	FALSE
ILMN_1749821	MED28	FALSE	FALSE
ILMN_1744316	TATDN3	FALSE	FALSE
ILMN_2186597	NA	FALSE	FALSE
ILMN_1898692	NA	TRUE	FALSE
ILMN_2085722	ING2	FALSE	FALSE
ILMN_3249624	PKD1P1	FALSE	FALSE
ILMN_1669273	PPT1	FALSE	FALSE
ILMN_1796165	GLRX5	FALSE	FALSE
ILMN_1662198	RANGAP1	FALSE	FALSE
ILMN_1683273	SNAPC5	FALSE	FALSE
ILMN_3248057	NA	TRUE	TRUE
ILMN_1674390	PRKAR1B	FALSE	FALSE
ILMN_1784630	KBTBD11	FALSE	FALSE
ILMN_1776552	FUBP1	FALSE	FALSE
ILMN_1700515	C17orf58	TRUE	FALSE
ILMN_1808779	CSTF3	FALSE	FALSE
ILMN_1751571	RAD23A	FALSE	FALSE
ILMN_1733107	NOC4L	FALSE	FALSE
ILMN_1703369	HNRNPA3	FALSE	FALSE
ILMN_1798459	NA	TRUE	FALSE
ILMN_1676846	ABCE1	TRUE	FALSE
ILMN_1669070	MIPEP	FALSE	FALSE
ILMN_1782247	KAT2A	FALSE	FALSE
ILMN_2368292	TSEN34	FALSE	FALSE
ILMN_1806946	UBTF	FALSE	FALSE
ILMN_1764770	OXNAD1	FALSE	FALSE
ILMN_1652085	MPHOSPH10	FALSE	FALSE
ILMN_1705985	PIGA	FALSE	FALSE
ILMN_2075189	SLC35F2	FALSE	FALSE
ILMN_3293681	PINLYP	FALSE	FALSE
ILMN_1729157	PGAM5	FALSE	FALSE
ILMN_1664776	EFR3A	FALSE	FALSE
ILMN_1781281	B9D1	TRUE	FALSE
ILMN_1749612	IMMP1L	FALSE	FALSE
ILMN_1797482	GCDH	FALSE	FALSE
ILMN_1673960	MAT2B	FALSE	FALSE
ILMN_1792951	ZHX2	FALSE	FALSE
ILMN_1711543	C14orf169	TRUE	FALSE
ILMN_2263144	TMEM223	TRUE	FALSE

ILMN_1811367	MAT2B	FALSE	FALSE
ILMN_1791896	EBAG9	TRUE	FALSE
ILMN_1743784	SHMT1	FALSE	FALSE
ILMN_3209358	NA	FALSE	FALSE
ILMN_1698258	DNAJC8	FALSE	FALSE
ILMN_1667796	NA	FALSE	FALSE
ILMN_1704055	NA	FALSE	FALSE
ILMN_1782730	ZNF473	FALSE	TRUE
ILMN_1716790	APBB2	FALSE	FALSE
ILMN_1806328	PDIK1L	FALSE	FALSE
ILMN_3260618	APIP	FALSE	FALSE
ILMN_1815682	HMCES	FALSE	FALSE
ILMN_2373495	H2AFY	FALSE	FALSE
ILMN_2097421	MRPL51	FALSE	FALSE
ILMN_1667374	L3HYPDH	FALSE	FALSE
ILMN_2156982	IMP4	FALSE	FALSE
ILMN_1796235	CIRH1A	FALSE	FALSE
ILMN_1667016	FAF1	FALSE	FALSE
ILMN_2181089	VPRBP	FALSE	FALSE
ILMN_1663684	NA	FALSE	FALSE
ILMN_1660986	PER3	FALSE	FALSE
ILMN_3200378	LOC401127	FALSE	FALSE
ILMN_2066124	AFG3L2	FALSE	FALSE
ILMN_1667561	IFRD1	FALSE	FALSE
ILMN_3224204	PSMG4	FALSE	FALSE
ILMN_3309534	MIR25	FALSE	FALSE
ILMN_1715583	BOP1	FALSE	FALSE
ILMN_1730685	MRPL16	FALSE	FALSE
ILMN_2406892	URI1	FALSE	FALSE
ILMN_1696591	RB1	FALSE	FALSE
ILMN_1704313	GSTCD	FALSE	FALSE
ILMN_3240117	AIDA	TRUE	FALSE
ILMN_1679881	WRN	TRUE	FALSE
ILMN_1786665	ZNF519	FALSE	FALSE
ILMN_1678680	DBR1	FALSE	FALSE
ILMN_2344130	PSMD4	FALSE	FALSE
ILMN_1731644	SETDB2	FALSE	FALSE
ILMN_1780700	GUSBP2	FALSE	FALSE
ILMN_1784753	PAIP2	FALSE	FALSE
ILMN_1660757	SRSF6	FALSE	FALSE
ILMN_1789018	ILF3	FALSE	FALSE
ILMN_1738173	METTL4	FALSE	FALSE
ILMN_1694596	FAR2	FALSE	FALSE
ILMN_2223805	CEP41	FALSE	FALSE
ILMN_1761147	GABPB1	FALSE	FALSE
ILMN_1704383	TRIM37	FALSE	FALSE

ILMN_1667977	TAF1B	TRUE	FALSE
ILMN_1747020	NA	FALSE	FALSE
ILMN_1784227	MCRS1	FALSE	TRUE
ILMN_1662065	WDR92	FALSE	FALSE
ILMN_1690099	ITGB1BP1	FALSE	FALSE
ILMN_1710170	PPAP2C	FALSE	FALSE
ILMN_1712936	PAXBP1	FALSE	FALSE
ILMN_1703036	WRNIP1	FALSE	FALSE
ILMN_1670172	WDR33	FALSE	FALSE
ILMN_1718769	ITSN1	FALSE	FALSE
ILMN_1804679	KAT8	FALSE	FALSE
ILMN_3192411	LINC00263	FALSE	FALSE
ILMN_1776674	SAC3D1	TRUE	FALSE
ILMN_1657288	GUSBP4	FALSE	FALSE
ILMN_3200830	CCT6P3	FALSE	FALSE
ILMN_1729269	LOC285074	FALSE	FALSE
ILMN_1789364	ZNF789	FALSE	FALSE
ILMN_1791400	ATE1	FALSE	FALSE
ILMN_1654313	NA	FALSE	FALSE
ILMN_2122952	CISD1	FALSE	FALSE
ILMN_2296369	NA	FALSE	FALSE
ILMN_1678605	CDC123	FALSE	FALSE
ILMN_1770673	AKNA	FALSE	FALSE
ILMN_1711189	EXOSC10	FALSE	FALSE
ILMN_1787081	TADA2A	FALSE	FALSE
ILMN_1773117	BCOR	FALSE	FALSE
ILMN_1813685	RAB29	FALSE	FALSE
ILMN_3255144	LOC100129104	TRUE	FALSE
ILMN_1666564	SUMO2	FALSE	FALSE
ILMN_1679318	ARSJ	FALSE	FALSE
ILMN_3240144	NA	FALSE	FALSE
ILMN_1706784	H2AFV	FALSE	FALSE
ILMN_1685413	ALG8	TRUE	FALSE
ILMN_2087528	CPSF3	FALSE	FALSE
ILMN_1735275	WDSUB1	FALSE	FALSE
ILMN_1803846	EIF1	FALSE	FALSE
ILMN_1656361	ARHGAP27	FALSE	FALSE
ILMN_1784328	SNORD25	TRUE	FALSE
ILMN_1708077	DAXX	FALSE	FALSE
ILMN_1894172	NA	FALSE	FALSE
ILMN_1797107	SCLT1	FALSE	FALSE
ILMN_1737084	TXLNA	FALSE	FALSE
ILMN_2370464	ATRIP	FALSE	TRUE
ILMN_1723439	ZYG11A	FALSE	FALSE
ILMN_2198393	KATNA1	FALSE	TRUE
ILMN_1716237	ACOT2	FALSE	FALSE

ILMN_1708427	KPNA3	FALSE	FALSE
ILMN_1762407	CABLES2	FALSE	FALSE
ILMN_1690965	DHX9	FALSE	FALSE
ILMN_2226324	MPC1	FALSE	FALSE
ILMN_2384536	ECI2	TRUE	FALSE
ILMN_3247008	SNORD96B	FALSE	FALSE
ILMN_1771620	SNRPB2	FALSE	FALSE
ILMN_1714805	ZC3H14	FALSE	FALSE
ILMN_1682062	RYR1	FALSE	TRUE
ILMN_1702759	TMX4	FALSE	FALSE
ILMN_1749243	BANF1	TRUE	FALSE
ILMN_1880113	NA	FALSE	FALSE
ILMN_1691747	KHDRBS3	FALSE	FALSE
ILMN_1788738	ZNRF3	FALSE	FALSE
ILMN_3244154	NA	FALSE	FALSE
ILMN_2410771	KEAP1	FALSE	TRUE
ILMN_1759729	NDUFA8	FALSE	FALSE
ILMN_1702198	HMGN1	FALSE	TRUE
ILMN_1661945	SLIRP	FALSE	FALSE
ILMN_1802162	RFESD	FALSE	FALSE
ILMN_2130838	UTP11L	TRUE	FALSE
ILMN_1676763	PIPSL	FALSE	FALSE
ILMN_2380850	SDCCAG3	FALSE	FALSE
ILMN_2408938	LRRC20	FALSE	FALSE
ILMN_1736888	SAR1B	FALSE	FALSE
ILMN_2082489	PRMT7	FALSE	FALSE
ILMN_1750596	CLUAP1	FALSE	FALSE
ILMN_1683927	ITGAE	FALSE	FALSE
ILMN_1735474	R3HCC1	FALSE	FALSE
ILMN_1728984	PA2G4	FALSE	FALSE
ILMN_2107068	HOXA2	FALSE	FALSE
ILMN_1790530	HECTD2	FALSE	FALSE
ILMN_1738656	GLOD4	TRUE	FALSE
ILMN_2124523	METTL14	FALSE	FALSE
ILMN_1752273	KIAA1143	FALSE	FALSE
ILMN_1729767	TARBP2	FALSE	FALSE
ILMN_2357809	G3BP1	FALSE	FALSE
ILMN_1746696	PDS5B	FALSE	FALSE
ILMN_1697614	NHP2L1	FALSE	FALSE
ILMN_1686194	CWC27	FALSE	FALSE
ILMN_1800612	VBP1	FALSE	FALSE
ILMN_1745779	TCTEX1D2	FALSE	FALSE
ILMN_1814657	TFAP4	FALSE	FALSE
ILMN_3243302	NA	FALSE	FALSE
ILMN_1855797	TMEM194B	FALSE	FALSE
ILMN_1745807	TMEM62	FALSE	FALSE

ILMN_1799320	ARRDC1-AS1	FALSE	FALSE
ILMN_3217262	API5	FALSE	FALSE
ILMN_1671028	THRAP3	FALSE	FALSE
ILMN_1800276	RCN1	FALSE	FALSE
ILMN_3231952	ARL17B	FALSE	FALSE
ILMN_1656196	E2F6	FALSE	FALSE
ILMN_3248575	SNORA80E	TRUE	FALSE
ILMN_1685663	CYP24A1	FALSE	TRUE
ILMN_1655635	METTL3	FALSE	FALSE
ILMN_1761560	PHF13	FALSE	FALSE
ILMN_1697975	PHF14	FALSE	FALSE
ILMN_3236945	PTPMT1	FALSE	FALSE
ILMN_1699206	FHDC1	FALSE	FALSE
ILMN_2378316	NUPL1	FALSE	FALSE
ILMN_1742782	GPANK1	FALSE	FALSE
ILMN_1812080	PARP16	FALSE	FALSE
ILMN_1715416	NUP188	FALSE	FALSE
ILMN_1770378	ANAPC10	FALSE	FALSE
ILMN_1711838	SLC25A24	FALSE	FALSE
ILMN_2415776	WWOX	FALSE	FALSE
ILMN_1658728	ZBTB25	FALSE	FALSE
ILMN_1765621	HDGF	FALSE	FALSE
ILMN_1813746	CORO2A	FALSE	FALSE
ILMN_1717727	HMSD	FALSE	FALSE
ILMN_1671911	MTA1	FALSE	FALSE
ILMN_1695422	NCL	FALSE	FALSE
ILMN_1752520	SLFN11	FALSE	FALSE
ILMN_1708382	ELP6	FALSE	FALSE
ILMN_1744725	BTBD6	FALSE	FALSE
ILMN_1660368	TRRAP	FALSE	FALSE
ILMN_1678165	LSM7	FALSE	FALSE
ILMN_2374293	DYRK1A	FALSE	FALSE
ILMN_1705679	SAFB2	FALSE	FALSE
ILMN_1812934	DIDO1	FALSE	FALSE
ILMN_3273885	C8orf88	FALSE	FALSE
ILMN_1701749	UQCRFS1	FALSE	FALSE
ILMN_1793302	WDR4	FALSE	FALSE
ILMN_1673711	HSP90AB1	FALSE	FALSE
ILMN_1728380	PHOSPHO2	FALSE	FALSE
ILMN_2097954	TSFM	FALSE	FALSE
ILMN_1740430	SLC2A4RG	FALSE	TRUE
ILMN_3191636	NA	FALSE	FALSE
ILMN_1696038	LOC150051	FALSE	FALSE
ILMN_1797698	RBM12	FALSE	TRUE
ILMN_1696004	LRRK1	FALSE	FALSE
ILMN_1698402	NFX1	FALSE	FALSE

ILMN_2311497	RCC1	FALSE	FALSE
ILMN_2363106	RBM23	FALSE	FALSE
ILMN_1685682	PCNA	FALSE	FALSE
ILMN_3306028	NA	FALSE	FALSE
ILMN_1729816	VDAC3	FALSE	FALSE
ILMN_1804539	NA	FALSE	FALSE
ILMN_2153485	NMNAT3	FALSE	TRUE
ILMN_2331348	TCOF1	FALSE	FALSE
ILMN_2121282	MRPS18B	FALSE	FALSE
ILMN_1793643	MRM1	FALSE	FALSE
ILMN_1682857	NDUFAF2	FALSE	FALSE
ILMN_2193591	UNC93B1	FALSE	FALSE
ILMN_2344956	ACP1	FALSE	FALSE
ILMN_1775943	BRMS1L	FALSE	FALSE
ILMN_1840273	STX18-AS1	FALSE	FALSE
ILMN_2088612	XPO4	FALSE	FALSE
ILMN_1852384	ERC1	FALSE	FALSE
ILMN_1706583	DLAT	FALSE	FALSE
ILMN_1673522	MOCOS	TRUE	TRUE
ILMN_3305304	POLD2	TRUE	FALSE
ILMN_1740490	ZFP82	FALSE	FALSE
ILMN_1680682	TADA2A	TRUE	FALSE
ILMN_1714738	SCMH1	FALSE	FALSE
ILMN_1780940	PPP2R5D	FALSE	FALSE
ILMN_3243457	ANKLE1	FALSE	FALSE
ILMN_1745813	KIAA1279	TRUE	FALSE
ILMN_1797425	DDX55	FALSE	FALSE
ILMN_1727618	NDUFAF6	FALSE	FALSE
ILMN_1748819	MRPL22	FALSE	FALSE
ILMN_1726815	HIST1H3G	FALSE	FALSE
ILMN_1815134	PI4K2B	FALSE	FALSE
ILMN_2102580	UTP20	FALSE	FALSE
ILMN_1813836	DARS	TRUE	FALSE
ILMN_3238751	PMS2P4	FALSE	FALSE
ILMN_2189406	ARPIN	TRUE	FALSE
ILMN_1804479	MRPL18	TRUE	FALSE
ILMN_1805028	THOC7	FALSE	FALSE
ILMN_1799579	CCDC51	FALSE	FALSE
ILMN_1750160	FASTKD3	FALSE	FALSE
ILMN_1793686	LOC399815	FALSE	FALSE
ILMN_3226392	RPF2	FALSE	FALSE
ILMN_1800942	KCTD6	FALSE	FALSE
ILMN_1742738	LURAP1	FALSE	FALSE
ILMN_1751338	NUP133	FALSE	FALSE
ILMN_1737635	RAD1	FALSE	FALSE
ILMN_1788024	PCID2	FALSE	FALSE

ILMN_1690802	TRMT112	TRUE	FALSE
ILMN_1726913	RBPJ	FALSE	FALSE
ILMN_1664537	USP11	FALSE	FALSE
ILMN_3251298	THNSL1	FALSE	FALSE
ILMN_2305544	DBI	FALSE	FALSE
ILMN_2116299	FAM110D	FALSE	FALSE
ILMN_1722858	PPP2CA	FALSE	FALSE
ILMN_1785268	CD58	FALSE	FALSE
ILMN_1667022	PASK	FALSE	FALSE
ILMN_1662334	DNAJA3	FALSE	TRUE
ILMN_1849192	LOC100506691	FALSE	FALSE
ILMN_1781419	C11orf73	TRUE	FALSE
ILMN_1652735	RFXAP	FALSE	FALSE
ILMN_1725366	SLC27A5	TRUE	FALSE
ILMN_1747052	ITGA4	FALSE	FALSE
ILMN_1739659	ZDHHC6	FALSE	FALSE
ILMN_1654812	UNC93B1	FALSE	FALSE
ILMN_1671621	PCMT1	FALSE	FALSE
ILMN_3232219	NA	FALSE	FALSE
ILMN_1743579	WDR4	FALSE	FALSE
ILMN_1757730	TTC27	FALSE	FALSE
ILMN_2383754	GTPBP10	FALSE	FALSE
ILMN_1782993	DHODH	FALSE	FALSE
ILMN_1763852	ACACB	FALSE	FALSE
ILMN_1683112	FANCC	FALSE	FALSE
ILMN_1740395	RAVER1	FALSE	FALSE
ILMN_2200915	RIPPLY2	FALSE	FALSE
ILMN_3251565	RNASEH2C	FALSE	FALSE
ILMN_3250850	RFESD	FALSE	FALSE
ILMN_1712636	NVL	FALSE	FALSE
ILMN_1696962	PDZD8	FALSE	FALSE
ILMN_3274671	FGF14-AS2	FALSE	FALSE
ILMN_2122953	CISD1	FALSE	FALSE
ILMN_1798728	URI1	FALSE	FALSE
ILMN_2124816	ZNF34	TRUE	FALSE
ILMN_1729319	USP7	FALSE	FALSE
ILMN_1669508	PAF1	FALSE	FALSE
ILMN_1730631	C2orf44	FALSE	TRUE
ILMN_1693664	POMGNT1	FALSE	FALSE
ILMN_3306742	SIGMAR1	FALSE	FALSE
ILMN_1800530	AGAP1	FALSE	FALSE
ILMN_1720542	POLR2I	FALSE	FALSE
ILMN_1725471	GK	FALSE	FALSE
ILMN_3307930	RAN	TRUE	FALSE
ILMN_1655924	TRNT1	FALSE	FALSE
ILMN_1676555	TTC26	FALSE	FALSE

ILMN_1682402	SNORD46	TRUE	FALSE
ILMN_1784678	PBX1	FALSE	FALSE
ILMN_1781526	PPP1R8	FALSE	FALSE
ILMN_1759117	XK	FALSE	FALSE
ILMN_2055477	EXOSC7	FALSE	FALSE
ILMN_1808661	TOMM5	FALSE	FALSE
ILMN_1654385	ASB13	TRUE	FALSE
ILMN_2207328	TPGS2	FALSE	FALSE
ILMN_1672378	ZP3	FALSE	FALSE
ILMN_1679389	NA	FALSE	FALSE
ILMN_3237419	NA	FALSE	FALSE
ILMN_1795524	VWA9	FALSE	FALSE
ILMN_2293992	RAB28	FALSE	FALSE
ILMN_1792672	POLR2D	FALSE	FALSE
ILMN_1655684	SARS2	FALSE	FALSE
ILMN_1782897	CAPRIN1	FALSE	FALSE
ILMN_1714522	CLMN	FALSE	FALSE
ILMN_1808148	SMCHD1	FALSE	FALSE
ILMN_2147306	PNRC2	FALSE	FALSE
ILMN_1756669	POGK	FALSE	FALSE
ILMN_2161508	PHTF2	FALSE	FALSE
ILMN_1671292	LOC390705	FALSE	FALSE
ILMN_1788022	CDKAL1	FALSE	FALSE
ILMN_1686432	ACYP1	FALSE	FALSE
ILMN_1705414	ELP5	FALSE	FALSE
ILMN_1655844	TAF1A	FALSE	FALSE
ILMN_1682501	CNOT1	FALSE	FALSE
ILMN_1750008	SUPV3L1	FALSE	TRUE
ILMN_3242211	TMEM187	FALSE	FALSE
ILMN_1699476	RPE	FALSE	FALSE
ILMN_1793201	HAGHL	FALSE	FALSE
ILMN_1806757	MYBBP1A	FALSE	FALSE
ILMN_1902929	NA	FALSE	FALSE
ILMN_1762622	FAM111B	FALSE	FALSE
ILMN_1676833	CYB5RL	FALSE	FALSE
ILMN_1732550	NA	FALSE	FALSE
ILMN_2291083	SLC6A15	FALSE	FALSE
ILMN_1768930	U2AF2	FALSE	TRUE
ILMN_2232936	UQCRH	FALSE	FALSE
ILMN_1679476	GART	FALSE	FALSE
ILMN_3207406	LOC727896	FALSE	FALSE
ILMN_1735360	SDAD1	FALSE	FALSE
ILMN_2377025	TCOF1	FALSE	FALSE
ILMN_1799688	CDC23	FALSE	FALSE
ILMN_1810474	UBE2I	TRUE	FALSE
ILMN_2147114	PSPH	FALSE	FALSE

ILMN_1803813	ASTE1	FALSE	FALSE
ILMN_1722838	MRPL46	FALSE	FALSE
ILMN_1753980	LRRC37B	FALSE	TRUE
ILMN_1697735	EWSR1	FALSE	TRUE
ILMN_1771411	ALG6	FALSE	FALSE
ILMN_3241985	SNORA13	TRUE	FALSE
ILMN_2155516	QTRTD1	FALSE	FALSE
ILMN_1761086	VPS54	FALSE	FALSE
ILMN_1658259	DRG1	FALSE	FALSE
ILMN_1857265	NA	FALSE	FALSE
ILMN_2080751	ADNP2	FALSE	FALSE
ILMN_3307799	PSMD4	FALSE	FALSE
ILMN_1726659	THOP1	FALSE	FALSE
ILMN_1808260	VWA8	FALSE	FALSE
ILMN_2187718	COX17	FALSE	FALSE
ILMN_1756308	NAE1	FALSE	FALSE
ILMN_1664167	RPF2	TRUE	FALSE
ILMN_1786766	SNTB2	FALSE	FALSE
ILMN_1709611	PSMA1	FALSE	FALSE
ILMN_1651513	SKIV2L2	FALSE	FALSE
ILMN_2092664	ADSS	FALSE	FALSE
ILMN_1697117	TBP	FALSE	FALSE
ILMN_3236049	SNORD12	FALSE	FALSE
ILMN_1662243	ING1	FALSE	FALSE
ILMN_2293167	LRR1	FALSE	FALSE
ILMN_1772713	BMS1	TRUE	FALSE
ILMN_2124361	LEAP2	FALSE	TRUE
ILMN_3291778	ABCB10	FALSE	FALSE
ILMN_1682339	C19orf57	FALSE	FALSE
ILMN_2368535	FBXO17	FALSE	FALSE
ILMN_1772522	ZBTB14	FALSE	FALSE
ILMN_2132809	ARHGEF10	FALSE	FALSE
ILMN_3280847	AK2	FALSE	FALSE
ILMN_1803570	BRI3BP	FALSE	FALSE
ILMN_1738407	ULBP1	FALSE	FALSE
ILMN_3307648	CS	FALSE	TRUE
ILMN_1706502	EIF2AK2	FALSE	FALSE
ILMN_3238478	SNORD11B	FALSE	FALSE
ILMN_1702177	GLO1	FALSE	FALSE
ILMN_1657139	ADAT1	FALSE	FALSE
ILMN_2357471	CASC5	FALSE	FALSE
ILMN_1756355	NDUFS3	FALSE	FALSE
ILMN_1779951	ANKRD7	FALSE	FALSE
ILMN_3182893	NA	FALSE	FALSE
ILMN_1660199	ACAA2	FALSE	FALSE
ILMN_3243366	KIAA1211L	FALSE	FALSE

ILMN_3237209	TOMM40	FALSE	TRUE
ILMN_1765520	MTIF2	FALSE	FALSE
ILMN_2408450	UBA5	FALSE	FALSE
ILMN_2151579	HMGN1	FALSE	FALSE
ILMN_3246247	PKD1P1	FALSE	FALSE
ILMN_2174729	ZNF318	FALSE	FALSE
ILMN_2352042	MRPL4	FALSE	FALSE
ILMN_1792092	ZCCHC8	FALSE	FALSE
ILMN_2360184	INVS	FALSE	FALSE
ILMN_2120072	FAM161A	FALSE	FALSE
ILMN_1784799	TRIM59	FALSE	FALSE
ILMN_1677887	PMS2	FALSE	FALSE
ILMN_3205264	NA	FALSE	FALSE
ILMN_1765146	IFNAR2	FALSE	FALSE
ILMN_1844611	NA	FALSE	TRUE
ILMN_1706706	DCAF7	FALSE	FALSE
ILMN_1702171	LPCAT1	FALSE	FALSE
ILMN_1812571	RAB35	FALSE	FALSE
ILMN_2129859	FASTKD3	FALSE	FALSE
ILMN_1750052	NOP14	FALSE	FALSE
ILMN_1674051	CCHCR1	FALSE	FALSE
ILMN_1757317	LARS	FALSE	FALSE
ILMN_1789616	NUPL2	FALSE	FALSE
ILMN_1681124	C19orf48	FALSE	FALSE
ILMN_2182198	ICT1	TRUE	FALSE
ILMN_2131880	DPY30	FALSE	FALSE
ILMN_1695362	ZNF32	TRUE	FALSE
ILMN_1674859	NA	FALSE	FALSE
ILMN_1659075	HLA-DOA	FALSE	FALSE
ILMN_2398039	TCERG1	FALSE	FALSE
ILMN_1688753	PTDSS1	TRUE	FALSE
ILMN_3275696	SNX5	FALSE	FALSE
ILMN_1657632	ZMYM6NB	FALSE	FALSE
ILMN_1718152	DEPDC7	FALSE	FALSE
ILMN_1698404	NA	FALSE	TRUE
ILMN_1781077	CEP120	FALSE	FALSE
ILMN_1802627	PSMG3	FALSE	FALSE
ILMN_1796005	TERT	FALSE	FALSE
ILMN_1751075	SETD4	FALSE	FALSE
ILMN_2189605	FAM122B	FALSE	FALSE
ILMN_2273103	ELK4	FALSE	FALSE
ILMN_1812289	CDK5RAP1	FALSE	FALSE
ILMN_3208027	NA	FALSE	FALSE
ILMN_1705871	DDHD2	FALSE	FALSE
ILMN_1744835	MRPL21	TRUE	FALSE
ILMN_2342121	PSMD13	FALSE	FALSE

ILMN_1677483	EXOSC1	FALSE	FALSE
ILMN_2055930	FARSB	FALSE	FALSE
ILMN_3250899	NA	FALSE	FALSE
ILMN_1673215	PCBP1	FALSE	FALSE
ILMN_1664177	ATXN7L2	FALSE	FALSE
ILMN_1742250	CCNH	FALSE	FALSE
ILMN_1768449	PRPSAP1	FALSE	FALSE
ILMN_2298860	NA	FALSE	FALSE
ILMN_1680856	MAMLD1	FALSE	FALSE
ILMN_1656145	GOT1	FALSE	TRUE
ILMN_2323302	SON	FALSE	FALSE
ILMN_1671158	MRPL13	FALSE	FALSE
ILMN_1781121	TWISTNB	FALSE	FALSE
ILMN_1809883	CCDC134	TRUE	FALSE
ILMN_3248026	RBM27	FALSE	FALSE
ILMN_1732187	TMEM143	FALSE	FALSE
ILMN_1784785	COPS7B	FALSE	FALSE
ILMN_1657836	PLEKHG2	FALSE	FALSE
ILMN_1857017	SNX8	FALSE	FALSE
ILMN_1699606	NA	FALSE	FALSE
ILMN_1659517	WDR89	FALSE	FALSE
ILMN_1689342	NUBP1	FALSE	FALSE
ILMN_1705390	KLHL22	FALSE	FALSE
ILMN_2193717	RAB42	FALSE	FALSE
ILMN_1743432	DGUOK	FALSE	FALSE
ILMN_1721337	MRPS18B	TRUE	FALSE
ILMN_1679322	SH2D4A	FALSE	FALSE
ILMN_1814820	NA	FALSE	FALSE
ILMN_1697268	EMILIN2	FALSE	FALSE
ILMN_1782795	SMPD4	FALSE	FALSE
ILMN_3290136	LOC643733	FALSE	FALSE
ILMN_1681899	NA	FALSE	FALSE
ILMN_2356890	MRPL42	FALSE	FALSE
ILMN_1855325	NA	FALSE	FALSE
ILMN_1733696	IMP3	FALSE	FALSE
ILMN_1689234	ZFP62	FALSE	FALSE
ILMN_1794349	XYLB	FALSE	FALSE
ILMN_1669972	NCR3LG1	FALSE	FALSE
ILMN_1726743	MRPS30	FALSE	FALSE
ILMN_1676848	NA	FALSE	FALSE
ILMN_1790410	DROSHA	FALSE	FALSE
ILMN_1740345	THYN1	FALSE	FALSE
ILMN_1803217	GPRIN2	FALSE	FALSE
ILMN_1657949	RHEB	FALSE	FALSE
ILMN_2357062	IL1RAP	FALSE	FALSE
ILMN_1763638	BCAR3	FALSE	FALSE

ILMN_1795678	POLR3C	FALSE	FALSE
ILMN_1766176	RAP1A	FALSE	FALSE
ILMN_1724479	NR2C2	FALSE	FALSE
ILMN_1728355	PSMD4	FALSE	FALSE
ILMN_2055165	MRFAP1	TRUE	FALSE
ILMN_1759870	NA	FALSE	FALSE
ILMN_1700766	ZNF324B	FALSE	FALSE
ILMN_1738263	PIGU	FALSE	TRUE
ILMN_3239621	SNRNP27	FALSE	FALSE
ILMN_1795007	C2orf47	FALSE	FALSE
ILMN_1699384	PPP2R5D	FALSE	FALSE
ILMN_2290686	SNX7	FALSE	FALSE
ILMN_1786426	IGFLR1	TRUE	FALSE
ILMN_2328666	CD83	FALSE	FALSE
ILMN_1763745	CCNJL	FALSE	FALSE
ILMN_1737096	CLDN10	FALSE	FALSE
ILMN_1776516	ITPKA	FALSE	FALSE
ILMN_3276203	NA	FALSE	FALSE
ILMN_1689774	MRFAP1L1	TRUE	FALSE
ILMN_1709605	MANEAL	FALSE	FALSE
ILMN_1714087	DGKH	FALSE	FALSE
ILMN_1726239	TBCA	FALSE	FALSE
ILMN_1715133	NA	FALSE	FALSE
ILMN_2214144	TWSG1	FALSE	FALSE
ILMN_1689070	COQ7	FALSE	FALSE
ILMN_2387090	CGGBP1	FALSE	FALSE
ILMN_2290618	SEC22C	FALSE	FALSE
ILMN_3247679	SNORA52	FALSE	FALSE
ILMN_1658909	OSGEPL1	FALSE	FALSE
ILMN_2404454	NLE1	FALSE	FALSE
ILMN_1696846	MIR4435-1HG	FALSE	FALSE
ILMN_1783728	TBRG4	FALSE	FALSE
ILMN_1785177	DNAJC14	TRUE	FALSE
ILMN_1782178	FLVCR1-AS1	FALSE	FALSE
ILMN_1678775	NA	FALSE	FALSE
ILMN_2396648	EXOSC1	FALSE	FALSE
ILMN_3299583	CKS1B	FALSE	FALSE
ILMN_1660343	NA	FALSE	FALSE
ILMN_1795976	SFXN2	FALSE	FALSE
ILMN_1794473	JADE1	FALSE	FALSE
ILMN_1712320	DDX50	TRUE	FALSE
ILMN_1697218	MED22	FALSE	TRUE
ILMN_1736460	MDM1	FALSE	FALSE
ILMN_1704398	FZD9	FALSE	TRUE
ILMN_1803939	YIPF6	FALSE	FALSE
ILMN_1759154	NA	FALSE	FALSE

ILMN_1798543	STK17B	TRUE	FALSE
ILMN_1718424	MRPS28	FALSE	FALSE
ILMN_3260715	CRTC3	FALSE	FALSE
ILMN_1696375	TTC31	FALSE	FALSE
ILMN_1730201	DTNA	FALSE	FALSE
ILMN_1762712	HHEX	FALSE	FALSE
ILMN_1665571	LINC01184	FALSE	FALSE
ILMN_2383163	DEPDC7	FALSE	FALSE
ILMN_3226082	RPL7L1	FALSE	FALSE
ILMN_1729314	PRG2	FALSE	TRUE
ILMN_1682176	CLEC3B	FALSE	FALSE
ILMN_2242068	NA	FALSE	TRUE
ILMN_1786658	BOLA3	FALSE	FALSE
ILMN_2227385	SLC16A14	FALSE	FALSE
ILMN_2397230	USP16	FALSE	FALSE
ILMN_1720242	NA	FALSE	FALSE
ILMN_1733667	DHX35	FALSE	TRUE
ILMN_1715672	HPCAL1	FALSE	FALSE
ILMN_1765522	PPM1G	FALSE	FALSE
ILMN_1796243	SLC13A3	FALSE	FALSE
ILMN_2341363	ATP5A1	FALSE	FALSE
ILMN_1783996	EXO5	FALSE	FALSE
ILMN_1726199	ZNF594	FALSE	FALSE
ILMN_2180997	GTF2IRD2B	FALSE	FALSE
ILMN_1663816	CCDC148	FALSE	FALSE
ILMN_1874506	NA	FALSE	FALSE
ILMN_1654571	FCHO1	FALSE	FALSE
ILMN_1728711	RGS3	FALSE	FALSE
ILMN_2395055	LPPR5	FALSE	FALSE
ILMN_1777726	USP48	FALSE	FALSE
ILMN_1667257	SDHB	FALSE	FALSE
ILMN_1814661	PHLPP1	FALSE	FALSE
ILMN_1811555	SWI5	FALSE	FALSE
ILMN_1658746	PTPLAD1	FALSE	FALSE
ILMN_1757636	SETD9	FALSE	FALSE
ILMN_2326793	NUP98	FALSE	FALSE
ILMN_1689747	NOL8	FALSE	FALSE
ILMN_1701477	CCDC101	FALSE	FALSE
ILMN_1820787	NA	FALSE	FALSE
ILMN_1709162	SHCBP1	TRUE	FALSE
ILMN_1880086	NA	FALSE	FALSE
ILMN_1766851	TMEM126B	FALSE	FALSE
ILMN_1757408	ZNF256	FALSE	FALSE
ILMN_1700487	TOP3A	FALSE	FALSE
ILMN_2300636	HMGXB4	FALSE	FALSE
ILMN_1686478	NA	FALSE	FALSE

ILMN_2090782	GCSH	TRUE	FALSE
ILMN_1760796	PDE4DIP	FALSE	FALSE
ILMN_2270015	AADAT	FALSE	FALSE
ILMN_1807919	TNS1	FALSE	FALSE
ILMN_1703573	DNAJC17	FALSE	FALSE
ILMN_1781454	RAE1	FALSE	TRUE
ILMN_3236536	IMMP1L	FALSE	FALSE
ILMN_1665601	THAP4	TRUE	FALSE
ILMN_1677292	C5orf30	FALSE	FALSE
ILMN_1653324	IL22RA2	FALSE	FALSE
ILMN_1809193	PSEN1	FALSE	FALSE
ILMN_1764323	METTL23	TRUE	TRUE
ILMN_3245413	DENND5A	FALSE	FALSE
ILMN_1742547	NRP1	FALSE	FALSE
ILMN_2388539	OGFOD3	FALSE	FALSE
ILMN_2088825	ACAP2	FALSE	FALSE
ILMN_2405156	PPAP2C	FALSE	FALSE
ILMN_1787511	THUMPD2	TRUE	FALSE
ILMN_2246956	BCL2	FALSE	FALSE
ILMN_1811966	PGGT1B	FALSE	FALSE
ILMN_1657041	NA	FALSE	FALSE
ILMN_1693107	MLH3	FALSE	FALSE
ILMN_3249598	RGS21	FALSE	FALSE
ILMN_2147920	BRD7P3	FALSE	FALSE
ILMN_2250048	KIF18B	FALSE	FALSE
ILMN_1702763	ZMYM1	FALSE	FALSE
ILMN_1725346	SNAPC1	FALSE	FALSE
ILMN_1777663	TOP2B	FALSE	FALSE
ILMN_2376416	UBXN11	FALSE	FALSE
ILMN_1692996	NA	FALSE	FALSE
ILMN_1742256	NA	FALSE	FALSE
ILMN_1787186	NOV	FALSE	FALSE
ILMN_1661650	SMEK2	FALSE	FALSE
ILMN_1665581	HAUS6	FALSE	FALSE
ILMN_1737738	NDUFA12	FALSE	FALSE
ILMN_2198575	C5orf34	FALSE	FALSE
ILMN_1689807	NA	FALSE	FALSE
ILMN_1760400	TTI2	FALSE	FALSE
ILMN_1678140	NA	FALSE	FALSE
ILMN_1784540	KBTBD2	FALSE	TRUE
ILMN_2381769	ATE1	FALSE	FALSE
ILMN_1779428	CCDC184	FALSE	FALSE
ILMN_1813120	MAP4K3	FALSE	FALSE
ILMN_3251881	LOC100130503	FALSE	FALSE
ILMN_1661307	JRK	FALSE	TRUE
ILMN_2334587	HNRNPC	FALSE	FALSE

ILMN_1714093	RMND5A	FALSE	FALSE
ILMN_1877756	NA	FALSE	FALSE
ILMN_3255978	FAM35A	FALSE	FALSE
ILMN_1664292	ZNF415	FALSE	FALSE
ILMN_1714219	FAM106A	FALSE	FALSE
ILMN_1658427	NA	FALSE	FALSE
ILMN_1801869	WDR75	FALSE	FALSE
ILMN_1702419	SLC35D3	FALSE	FALSE
ILMN_1694487	NA	FALSE	FALSE
ILMN_1672741	ITSN1	FALSE	FALSE
ILMN_1659926	ST7L	FALSE	FALSE
ILMN_1712314	DUSP9	FALSE	FALSE
ILMN_1764714	C19orf80	FALSE	FALSE
ILMN_1794987	C17orf100	TRUE	FALSE
ILMN_2085362	FUCA2	FALSE	FALSE
ILMN_1674782	LINC01116	FALSE	FALSE
ILMN_1669057	NA	FALSE	FALSE
ILMN_3205604	FAM185A	FALSE	FALSE
ILMN_1776267	CLN6	FALSE	FALSE
ILMN_2053567	FASTKD2	FALSE	TRUE
ILMN_1692072	CENPBD1	FALSE	FALSE
ILMN_2092118	FPR1	FALSE	FALSE
ILMN_1781225	MORC2-AS1	FALSE	FALSE
ILMN_1651486	COX11	FALSE	FALSE
ILMN_1746027	GNAL	FALSE	FALSE
ILMN_1662147	MANEAL	FALSE	FALSE
ILMN_1706718	TMEM80	FALSE	FALSE
ILMN_1745764	ESYT3	FALSE	FALSE
ILMN_2399503	UBN1	FALSE	FALSE
ILMN_1776121	KIAA1211L	TRUE	FALSE
ILMN_1719826	LOC441956	FALSE	FALSE
ILMN_1722045	ARSB	FALSE	FALSE
ILMN_1732336	RFC2	FALSE	FALSE
ILMN_1670901	COX10	FALSE	FALSE
ILMN_1724183	CDKL1	FALSE	FALSE
ILMN_1654436	NA	FALSE	FALSE
ILMN_1773247	ZNF410	FALSE	FALSE
ILMN_1793578	ZFP37	FALSE	FALSE
ILMN_1708950	ASPHD1	FALSE	FALSE
ILMN_1687403	MRPL40	FALSE	FALSE
ILMN_1658399	KLRG1	FALSE	FALSE
ILMN_1745798	GTF2F2	FALSE	FALSE
ILMN_3305339	UBA5	FALSE	FALSE
ILMN_2115154	NUPL2	FALSE	FALSE
ILMN_1763641	ZNF614	FALSE	FALSE
ILMN_3268535	HEXA-AS1	FALSE	FALSE

ILMN_1686151	NA	FALSE	FALSE
ILMN_1807981	SIGIRR	FALSE	FALSE
ILMN_1770087	MOCS2	FALSE	FALSE
ILMN_3179565	NA	FALSE	FALSE
ILMN_1698191	SCML2	FALSE	FALSE
ILMN_1715096	FAXC	FALSE	FALSE
ILMN_3193999	NA	FALSE	FALSE
ILMN_2394552	WNK3	FALSE	FALSE
ILMN_1666757	NA	FALSE	FALSE
ILMN_2387712	AK5	FALSE	FALSE
ILMN_1666939	NA	FALSE	FALSE
ILMN_1653666	KLHL7	FALSE	FALSE
ILMN_1794692	DNMT3B	FALSE	FALSE
ILMN_1904931	KCNQ5	FALSE	FALSE
ILMN_1788813	RASL10B	FALSE	FALSE
ILMN_2059549	SYK	FALSE	FALSE
ILMN_1680967	SARNP	FALSE	FALSE
ILMN_1806502	ZNF165	FALSE	FALSE
ILMN_2139125	LRFN5	FALSE	FALSE
ILMN_3257884	HMGXB4	FALSE	FALSE
ILMN_1663562	NA	FALSE	FALSE
ILMN_1805827	PPA1	TRUE	FALSE
ILMN_3237971	SNORD11	FALSE	FALSE
ILMN_2223010	VBP1	FALSE	FALSE
ILMN_2046611	MCOLN3	FALSE	FALSE
ILMN_1874577	LOC440117	FALSE	FALSE
ILMN_1716546	NA	FALSE	FALSE
ILMN_2369785	SNRPD2	FALSE	TRUE
ILMN_1686360	ZNF124	FALSE	FALSE
ILMN_3206475	HMGN1	FALSE	FALSE
ILMN_1775962	MCOLN3	FALSE	TRUE
ILMN_2375973	ALKBH1	FALSE	FALSE
ILMN_1749372	GGT5	FALSE	FALSE
ILMN_1744941	TCOF1	FALSE	FALSE
ILMN_1708095	PANK2	FALSE	FALSE
ILMN_2274180	MKKS	FALSE	FALSE
ILMN_3247462	GLRX3	FALSE	FALSE
ILMN_2315289	PEX10	FALSE	FALSE
ILMN_3236122	NA	FALSE	FALSE
ILMN_2381206	DHODH	FALSE	FALSE
ILMN_1747934	ISYNA1	FALSE	FALSE
ILMN_1689621	RNF17	FALSE	FALSE
ILMN_1812482	NA	FALSE	FALSE
ILMN_1805474	C1orf131	FALSE	FALSE
ILMN_1782983	NA	FALSE	FALSE
ILMN_2387553	PSMA3	FALSE	FALSE

ILMN_1903877	NA	FALSE	FALSE
ILMN_1672287	MYNN	FALSE	FALSE
ILMN_1748438	POLR2G	TRUE	FALSE
ILMN_1774799	NA	FALSE	FALSE
ILMN_1652594	ACTR5	FALSE	TRUE
ILMN_2081813	PCSK1	FALSE	FALSE
ILMN_2198376	PSMA4	FALSE	FALSE
ILMN_1765993	DMBX1	FALSE	FALSE
ILMN_1690520	GOLGA2	FALSE	FALSE
ILMN_3306944	CASC2	FALSE	FALSE
ILMN_1722995	NA	FALSE	FALSE
ILMN_1662438	SOD1	FALSE	FALSE
ILMN_1681193	EPS8L1	FALSE	FALSE
ILMN_1782429	TMEM56	FALSE	FALSE
ILMN_1696110	TUBGCP3	FALSE	FALSE
ILMN_2130514	FAM76B	FALSE	FALSE
ILMN_2318459	TMEM8B	FALSE	FALSE
ILMN_1738420	TMEM201	FALSE	TRUE
ILMN_3236970	LINC00667	FALSE	FALSE
ILMN_1911968	NA	FALSE	FALSE
ILMN_2186715	CENPH	FALSE	FALSE
ILMN_3195401	GABPB1	FALSE	FALSE
ILMN_1749583	ZNF777	FALSE	FALSE
ILMN_2056760	MKRN2	FALSE	FALSE
ILMN_1749675	WNT8B	FALSE	FALSE
ILMN_2411658	HPS1	FALSE	FALSE
ILMN_1882284	NA	FALSE	FALSE
ILMN_2132082	NA	FALSE	FALSE
ILMN_1731688	KLB	FALSE	FALSE
ILMN_1698680	ARL17A	FALSE	TRUE
ILMN_1656372	PES1	FALSE	FALSE
ILMN_3235969	SNORA9	FALSE	FALSE
ILMN_1724306	CACNG1	FALSE	FALSE
ILMN_1730795	NA	FALSE	FALSE
ILMN_2103024	RBP5	FALSE	FALSE
ILMN_1667830	NA	FALSE	FALSE
ILMN_1740263	P2RX3	FALSE	FALSE
ILMN_2189222	KLHL8	FALSE	FALSE
ILMN_1716468	NUDT16P1	FALSE	FALSE
ILMN_1658621	NA	FALSE	FALSE
ILMN_3191596	NA	FALSE	FALSE
ILMN_1676159	STK26	TRUE	TRUE
ILMN_1745501	DNALI1	FALSE	FALSE
ILMN_1693650	FES	FALSE	FALSE
ILMN_1875729	LINC00111	FALSE	FALSE
ILMN_1665423	ZFP91	FALSE	FALSE

ILMN_1755138	NEK8	FALSE	FALSE
ILMN_1746763	ECM2	FALSE	FALSE
ILMN_1753745	HDDC2	FALSE	FALSE
ILMN_1743095	CCDC151	FALSE	FALSE
ILMN_3244954	TMEM194B	FALSE	FALSE
ILMN_1682383	NA	FALSE	FALSE
ILMN_1716246	FRZB	FALSE	FALSE
ILMN_1716734	CNOT7	FALSE	FALSE
ILMN_3251418	ARHGEF39	FALSE	FALSE
ILMN_1733515	LOXL3	FALSE	FALSE
ILMN_1893010	NA	FALSE	FALSE
ILMN_1684875	LOC100288637	FALSE	FALSE
ILMN_2274605	FRMPD2B	FALSE	FALSE
ILMN_2336970	MUTYH	FALSE	FALSE
ILMN_1825660	CCDC15	FALSE	FALSE
ILMN_1680354	NA	FALSE	FALSE
ILMN_3190834	ELMOD3	FALSE	FALSE
ILMN_1717453	NA	FALSE	FALSE
ILMN_1680393	SNORD55	FALSE	FALSE
ILMN_3215374	LOC257396	FALSE	FALSE
ILMN_1654217	MPP2	FALSE	FALSE
ILMN_1774983	IL17A	FALSE	FALSE
ILMN_1666409	PSMB6	FALSE	FALSE
ILMN_1711599	ZFP62	FALSE	FALSE
ILMN_3308828	MIR1253	FALSE	FALSE
ILMN_1775944	ABI2	FALSE	FALSE
ILMN_1666179	NA	FALSE	FALSE
ILMN_1659536	NA	FALSE	FALSE
ILMN_1806683	LCE4A	FALSE	FALSE
ILMN_2082810	BRD7	FALSE	FALSE
ILMN_1798602	PCF11	FALSE	FALSE
ILMN_2226628	ZNF184	FALSE	FALSE
ILMN_1792960	NA	FALSE	FALSE
ILMN_1686454	TIFA	FALSE	FALSE
ILMN_1739368	ZSCAN25	FALSE	FALSE
ILMN_1751241	TCOF1	FALSE	FALSE
ILMN_1732329	NA	FALSE	FALSE
ILMN_1747172	ZNF670	FALSE	FALSE
ILMN_1771334	EML5	FALSE	FALSE
ILMN_1811598	ADH1B	FALSE	FALSE
ILMN_1671217	RPS15	FALSE	FALSE
ILMN_1722387	SCAI	FALSE	FALSE
ILMN_3239937	NUDT16P1	FALSE	FALSE
ILMN_1693886	NA	FALSE	FALSE
ILMN_1671209	SYTL2	FALSE	FALSE
ILMN_1682298	ERMN	FALSE	FALSE

ILMN_1723793	PALB2	FALSE	FALSE
ILMN_1701101	NA	FALSE	FALSE
ILMN_3211833	CSPG4	FALSE	FALSE
ILMN_1760683	SRSF9	FALSE	FALSE
ILMN_1739274	PDHB	FALSE	FALSE
ILMN_2224230	NA	FALSE	FALSE
ILMN_3211641	NA	FALSE	FALSE
ILMN_1671981	NA	FALSE	FALSE
ILMN_2171471	SLC1A6	FALSE	FALSE
ILMN_1689515	CPLX3	FALSE	FALSE
ILMN_2353732	CD8A	FALSE	FALSE
ILMN_1787540	NA	FALSE	FALSE
ILMN_1907847	NA	FALSE	FALSE
ILMN_1675788	ZNF175	FALSE	FALSE
ILMN_2323848	PARD6A	FALSE	FALSE
ILMN_1700408	TRIM50	FALSE	FALSE
ILMN_3215392	LOC100131514	FALSE	FALSE
ILMN_1670959	CEACAM5	FALSE	FALSE
ILMN_1815874	NANS	FALSE	FALSE
ILMN_1777905	NA	FALSE	FALSE
ILMN_1661788	WNT5B	FALSE	FALSE
ILMN_2314208	S100PBP	FALSE	FALSE
ILMN_1666631	NA	FALSE	FALSE
ILMN_1764795	FMN2	FALSE	FALSE
ILMN_3274380	NA	FALSE	FALSE
ILMN_1763333	NA	FALSE	FALSE
ILMN_1775345	SRSF12	FALSE	FALSE
ILMN_1815405	NA	FALSE	FALSE
ILMN_1785795	METAP1	FALSE	FALSE
ILMN_2296776	PTPRA	FALSE	FALSE
ILMN_1728802	SDCCAG8	FALSE	TRUE
ILMN_3246845	VPS35	FALSE	FALSE
ILMN_1752646	NA	FALSE	FALSE
ILMN_1771000	NA	FALSE	FALSE
ILMN_1694774	OR52J3	FALSE	FALSE
ILMN_1690561	GZMM	FALSE	FALSE
ILMN_3180960	NA	FALSE	FALSE
ILMN_1789176	PSMB1	FALSE	FALSE
ILMN_1668069	SMPD5	FALSE	FALSE
ILMN_1671626	DLGAP3	FALSE	FALSE
ILMN_3243534	NA	FALSE	FALSE
ILMN_3235133	MYO5B	FALSE	FALSE
ILMN_3211047	BASP1P1	FALSE	FALSE
ILMN_1691334	NME8	FALSE	FALSE
ILMN_3235954	NA	FALSE	FALSE
ILMN_3296267	NA	FALSE	FALSE

ILMN_2086952	NA	FALSE	FALSE
ILMN_3237381	TMEM217	FALSE	FALSE
ILMN_1656383	SOHLH1	FALSE	FALSE
ILMN_1676907	NA	FALSE	FALSE
ILMN_1699091	GPATCH2L	FALSE	FALSE
ILMN_1676992	NA	FALSE	FALSE
ILMN_1704154	TNFRSF19	FALSE	FALSE
ILMN_1662792	NA	FALSE	FALSE
ILMN_1674541	KIRREL3	FALSE	FALSE
ILMN_1762529	SLC12A8	TRUE	FALSE
ILMN_1784728	NA	FALSE	FALSE
ILMN_3240433	GSTT2B	FALSE	FALSE
ILMN_1764414	SOS2	FALSE	FALSE
ILMN_1871306	NA	FALSE	FALSE
ILMN_1742962	CLEC6A	TRUE	FALSE
ILMN_3298671	GUSBP10	FALSE	FALSE
ILMN_1684377	NA	FALSE	FALSE
ILMN_1774823	RPL34	FALSE	FALSE
ILMN_1723732	NA	FALSE	FALSE
ILMN_1770460	FOXR1	FALSE	FALSE
ILMN_1694864	NA	FALSE	FALSE
ILMN_1759493	NA	FALSE	FALSE
ILMN_1740351	IST1	FALSE	FALSE
ILMN_3275012	TUBB8	FALSE	FALSE
ILMN_3289594	NA	FALSE	FALSE
ILMN_2308903	WFDC3	FALSE	FALSE
ILMN_1727241	NA	FALSE	FALSE
ILMN_1668646	NA	FALSE	FALSE
ILMN_3269108	NA	FALSE	FALSE
ILMN_1686562	KIF13B	FALSE	FALSE
ILMN_1910893	NA	FALSE	FALSE
ILMN_1786823	ICAM2	FALSE	FALSE
ILMN_1719149	NA	FALSE	FALSE
ILMN_1718636	NA	FALSE	FALSE
ILMN_1717494	NA	FALSE	FALSE
ILMN_1785592	DUSP13	FALSE	FALSE
ILMN_1755120	MAN1A2	FALSE	FALSE
ILMN_1859439	NA	FALSE	FALSE
ILMN_3240267	LOC728323	FALSE	FALSE
ILMN_1804895	LSMEM1	FALSE	FALSE
ILMN_1714118	CD164L2	FALSE	FALSE
ILMN_2185728	SFRP4	FALSE	FALSE
ILMN_1704598	HYAL4	FALSE	FALSE
ILMN_1798395	PIGH	FALSE	FALSE
ILMN_3225634	NA	FALSE	FALSE
ILMN_1870681	NA	FALSE	FALSE

ILMN_1760889	CARF	FALSE	FALSE
ILMN_1656266	FLJ38379	FALSE	FALSE
ILMN_3278835	NA	FALSE	FALSE
ILMN_1759732	RFNG	FALSE	FALSE
ILMN_1738759	PIGT	FALSE	TRUE
ILMN_1663569	FTCD	FALSE	FALSE
ILMN_1678312	ARL17A	FALSE	FALSE
ILMN_1736655	LCE1B	FALSE	FALSE
ILMN_1712776	KCNK7	FALSE	FALSE
ILMN_1709725	PAPL	FALSE	FALSE
ILMN_3226045	IST1	FALSE	FALSE
ILMN_1719735	NA	FALSE	FALSE
ILMN_1742382	RIMS3	FALSE	FALSE
ILMN_3237665	COX7A2L	FALSE	FALSE
ILMN_1730416	CYCS	FALSE	FALSE
ILMN_1694535	PDZK1	FALSE	FALSE
ILMN_1727809	STK35	FALSE	FALSE
ILMN_1664960	CEP170B	FALSE	FALSE
ILMN_2257833	BBS7	FALSE	FALSE
ILMN_1728371	LOC643201	FALSE	FALSE
ILMN_1715301	FXYD2	FALSE	FALSE
ILMN_2274420	SPTLC1	FALSE	FALSE
ILMN_1738632	PRKAR1A	FALSE	FALSE
ILMN_3300353	ISPD	FALSE	FALSE
ILMN_2214355	RAB30	TRUE	FALSE
ILMN_3235789	NA	FALSE	FALSE
ILMN_1755589	DIP2B	FALSE	FALSE
ILMN_1810727	SLC25A45	FALSE	FALSE
ILMN_2395496	KLK7	FALSE	FALSE
ILMN_1666191	KLK13	FALSE	FALSE
ILMN_2052686	LCA5	FALSE	FALSE
ILMN_3261594	NA	FALSE	FALSE
ILMN_1770245	EPB41L5	FALSE	FALSE
ILMN_1915629	NA	FALSE	FALSE
ILMN_1855510	NA	FALSE	FALSE
ILMN_2205050	PRKX	FALSE	FALSE
ILMN_1836309	MCF2L-AS1	FALSE	FALSE
ILMN_1671895	ZNF613	TRUE	TRUE
ILMN_3245204	NA	FALSE	FALSE
ILMN_1751793	PCNXL2	FALSE	FALSE
ILMN_1678080	FKBP2	FALSE	FALSE
ILMN_1693345	CPEB1	FALSE	FALSE
ILMN_2333440	TM9SF1	FALSE	FALSE
ILMN_1796281	MYF5	FALSE	FALSE
ILMN_1781980	CFC1B	FALSE	FALSE
ILMN_1665554	BRF2	TRUE	FALSE

ILMN_1699525	SRI	FALSE	FALSE
ILMN_1776861	HAP1	FALSE	FALSE
ILMN_1685547	ZXDB	FALSE	FALSE
ILMN_1871488	NA	FALSE	FALSE
ILMN_1819223	NA	FALSE	FALSE
ILMN_1859942	NA	FALSE	FALSE
ILMN_1660111	UCHL3	FALSE	FALSE
ILMN_1876203	NA	FALSE	FALSE
ILMN_1788793	KLF8	FALSE	FALSE
ILMN_2166972	BBS12	FALSE	FALSE
ILMN_2215639	TUBA3D	FALSE	FALSE
ILMN_1782037	IGSF3	FALSE	FALSE
ILMN_1846282	NA	FALSE	FALSE
ILMN_1775822	PDGFB	FALSE	FALSE
ILMN_1899886	NA	FALSE	FALSE
ILMN_1782385	POLR2A	FALSE	FALSE
ILMN_3238772	TAS2R30	FALSE	FALSE
ILMN_1692077	MXRA7	FALSE	FALSE
ILMN_1733831	PGBD5	FALSE	FALSE
ILMN_1774844	MAPKAPK2	FALSE	FALSE
ILMN_1669553	UBE2E3	FALSE	FALSE
ILMN_1652602	LMNTD2	FALSE	FALSE
ILMN_1762100	NA	FALSE	FALSE
ILMN_2393763	ARPC4	FALSE	FALSE
ILMN_1910676	NA	FALSE	FALSE
ILMN_1723115	CLEC4F	FALSE	FALSE
ILMN_1774806	SLC9A7	FALSE	FALSE
ILMN_1655446	XKR6	FALSE	FALSE
ILMN_1675770	SEC14L3	FALSE	FALSE
ILMN_1698427	TMUB2	FALSE	FALSE
ILMN_3287814	ST13	FALSE	FALSE
ILMN_1655433	BCKDHB	FALSE	FALSE
ILMN_2356838	CEPT1	FALSE	FALSE
ILMN_1659000	XPNPEP3	FALSE	FALSE
ILMN_1660541	NA	FALSE	FALSE
ILMN_1769218	NA	FALSE	FALSE
ILMN_1711289	GYS1	FALSE	FALSE
ILMN_1807759	TAS2R31	FALSE	FALSE
ILMN_3263243	LOC100128653	FALSE	FALSE
ILMN_1798488	GPR144	FALSE	FALSE
ILMN_1908135	NA	FALSE	FALSE
ILMN_1655949	NA	FALSE	FALSE
ILMN_2398865	VPS13C	FALSE	FALSE
ILMN_1727592	NA	FALSE	FALSE
ILMN_3308315	MIR1269A	FALSE	FALSE
ILMN_2358628	ADK	FALSE	FALSE

ILMN_1652952	RASAL2	FALSE	FALSE
ILMN_2067813	OR1L4	FALSE	FALSE
ILMN_1744539	NA	FALSE	FALSE
ILMN_1711203	C6orf15	FALSE	FALSE
ILMN_1678075	CDYL	FALSE	FALSE
ILMN_1740549	NA	FALSE	FALSE
ILMN_1789790	NA	FALSE	FALSE
ILMN_1705874	NA	FALSE	FALSE
ILMN_2415170	VPS8	FALSE	FALSE
ILMN_3226214	LOC728755	FALSE	FALSE
ILMN_1749212	NA	TRUE	FALSE
ILMN_1714252	SLC16A13	FALSE	FALSE
ILMN_1673896	MAP3K13	FALSE	FALSE
ILMN_2152402	ABHD16A	FALSE	FALSE
ILMN_1739128	NA	FALSE	FALSE
ILMN_1744299	GPR110	FALSE	FALSE
ILMN_1666852	TRIM62	FALSE	FALSE
ILMN_1795158	FGR	FALSE	FALSE
ILMN_2313672	IL1RL1	FALSE	FALSE
ILMN_1793433	RAB10	FALSE	FALSE
ILMN_1718514	PSG6	FALSE	FALSE
ILMN_1764022	FXYD1	FALSE	FALSE
ILMN_1815519	EPN2	FALSE	FALSE
ILMN_2157932	FRMD8	FALSE	FALSE
ILMN_1809708	KCTD21	FALSE	FALSE
ILMN_1699931	HCST	FALSE	FALSE
ILMN_1663263	EPHA10	FALSE	FALSE
ILMN_1810805	HEATR5B	FALSE	FALSE
ILMN_1755850	ZNF350	FALSE	TRUE
ILMN_2134723	OR7E91P	FALSE	FALSE
ILMN_1769633	CTSO	FALSE	FALSE
ILMN_3310306	MIR1224	FALSE	FALSE
ILMN_2279961	LAMP2	FALSE	FALSE
ILMN_2094875	NA	FALSE	FALSE
ILMN_3260026	HIGD1A	FALSE	FALSE
ILMN_1665065	SERINC3	TRUE	FALSE
ILMN_1745737	DIS3L2	FALSE	FALSE
ILMN_2129015	AFF1	FALSE	FALSE
ILMN_1782558	XG	FALSE	FALSE
ILMN_1696682	ABLIM1	FALSE	FALSE
ILMN_1729650	PEX7	FALSE	FALSE
ILMN_1765979	VSIG10L	FALSE	FALSE
ILMN_3310890	MIR1206	FALSE	FALSE
ILMN_1663281	ZNF702P	FALSE	FALSE
ILMN_1796113	BCDIN3D	FALSE	FALSE
ILMN_1709877	TRIML1	FALSE	FALSE

ILMN_2118129	ITLN2	FALSE	FALSE
ILMN_1702496	STX17-AS1	FALSE	FALSE
ILMN_1740185	TPMT	TRUE	FALSE
ILMN_1759501	TNFSF15	FALSE	FALSE
ILMN_2075507	MAN1A2	FALSE	FALSE
ILMN_1694943	CAST	FALSE	FALSE
ILMN_1662950	EPS8L1	FALSE	FALSE
ILMN_1783060	NA	FALSE	FALSE
ILMN_1732767	PSMD9	FALSE	FALSE
ILMN_1795704	KIAA0232	TRUE	FALSE
ILMN_1684266	LIMD1-AS1	FALSE	FALSE
ILMN_1703153	GLYR1	FALSE	FALSE
ILMN_1724324	PLD3	FALSE	FALSE
ILMN_1782015	FCRLB	FALSE	FALSE
ILMN_1752027	UBE3B	TRUE	FALSE
ILMN_2409596	RAB11FIP1	FALSE	FALSE
ILMN_2047599	TMEM50B	FALSE	FALSE
ILMN_1654653	KLC1	FALSE	TRUE
ILMN_1670757	NA	FALSE	FALSE
ILMN_1659761	SNX29	FALSE	FALSE
ILMN_1789642	DNAJC5	FALSE	TRUE
ILMN_1760027	WAS	FALSE	FALSE
ILMN_1862217	APH1B	FALSE	FALSE
ILMN_1760335	ADPRHL1	FALSE	FALSE
ILMN_1770038	LAMA1	FALSE	FALSE
ILMN_1795835	LINC00936	FALSE	FALSE
ILMN_1720034	PKHD1	FALSE	FALSE
ILMN_2175474	MTRF1L	FALSE	FALSE
ILMN_1660856	ALG11	FALSE	FALSE
ILMN_1702787	SEMA4A	FALSE	FALSE
ILMN_1757847	C11orf68	FALSE	TRUE
ILMN_1765355	NOX1	FALSE	FALSE
ILMN_2180315	ATG4D	FALSE	FALSE
ILMN_1653599	ATP5D	FALSE	TRUE
ILMN_1796912	ARHGEF7	FALSE	FALSE
ILMN_1746232	KITLG	FALSE	FALSE
ILMN_1806713	ZNF18	FALSE	FALSE
ILMN_1750250	ABCA12	FALSE	FALSE
ILMN_1724581	EDC3	FALSE	FALSE
ILMN_1661611	NA	FALSE	FALSE
ILMN_3211677	FGD5P1	FALSE	FALSE
ILMN_1714741	NA	FALSE	TRUE
ILMN_1793632	TMEM222	FALSE	FALSE
ILMN_1741389	B3GNT8	FALSE	FALSE
ILMN_3239263	RPL23AP53	FALSE	FALSE
ILMN_1700770	PSKH1	FALSE	FALSE

ILMN_2316278	NA	FALSE	FALSE
ILMN_1690352	ADO	TRUE	FALSE
ILMN_2139989	ACTBL2	FALSE	FALSE
ILMN_3242176	UBR3	FALSE	FALSE
ILMN_1797021	TMEM86A	FALSE	FALSE
ILMN_1729851	ODAM	FALSE	FALSE
ILMN_1695717	RBM41	FALSE	FALSE
ILMN_1661138	GON4L	FALSE	FALSE
ILMN_3248122	MED1	FALSE	FALSE
ILMN_1733538	RGS10	FALSE	FALSE
ILMN_1791875	NA	FALSE	FALSE
ILMN_1660815	CAB39L	FALSE	FALSE
ILMN_3237627	LOC154761	FALSE	FALSE
ILMN_1691436	BLVRA	FALSE	TRUE
ILMN_1792799	NA	FALSE	FALSE
ILMN_1683872	NA	FALSE	FALSE
ILMN_1756590	SYS1	FALSE	FALSE
ILMN_1802257	PCTP	FALSE	FALSE
ILMN_1709265	RNF170	FALSE	FALSE
ILMN_2102069	SLC16A4	FALSE	FALSE
ILMN_1763670	TM2D1	FALSE	FALSE
ILMN_1669064	ZNF823	FALSE	FALSE
ILMN_1915188	MID2	FALSE	FALSE
ILMN_1737949	GALNS	FALSE	FALSE
ILMN_2388955	FAM212B	FALSE	FALSE
ILMN_2228180	MSRA	FALSE	FALSE
ILMN_1778673	GOLGA7	FALSE	FALSE
ILMN_1753597	FKRP	FALSE	FALSE
ILMN_1793829	TMCO1	FALSE	FALSE
ILMN_1858668	NA	FALSE	FALSE
ILMN_2297315	CDRT1	FALSE	FALSE
ILMN_1738229	NDRG3	FALSE	TRUE
ILMN_1721741	NA	TRUE	FALSE
ILMN_1706758	SEC24C	TRUE	FALSE
ILMN_1792014	TMEM255B	FALSE	FALSE
ILMN_1798472	UBA6-AS1	FALSE	FALSE
ILMN_1658689	NA	FALSE	FALSE
ILMN_1705114	NUMB	FALSE	FALSE
ILMN_1674758	NA	FALSE	FALSE
ILMN_3240829	ZSCAN12P1	FALSE	FALSE
ILMN_1743966	BCL9L	FALSE	FALSE
ILMN_1656706	LCE2D	FALSE	TRUE
ILMN_3308245	MIR524	FALSE	FALSE
ILMN_2046730	S100A10	FALSE	FALSE
ILMN_1676194	KRT17	FALSE	FALSE
ILMN_1652490	MANSC1	FALSE	FALSE

ILMN_1652687	TMEM198B	FALSE	FALSE
ILMN_2203729	HCG4	FALSE	FALSE
ILMN_1670377	NA	TRUE	TRUE
ILMN_2372011	SCAND1	FALSE	TRUE
ILMN_1751500	ADAM15	FALSE	FALSE
ILMN_1666022	TNFRSF10D	FALSE	FALSE
ILMN_1787199	NA	TRUE	FALSE
ILMN_1747285	NA	FALSE	FALSE
ILMN_1880387	AAK1	FALSE	FALSE
ILMN_1712312	RAB11A	FALSE	FALSE
ILMN_1917290	LAMP2	FALSE	FALSE
ILMN_1747347	MILR1	FALSE	FALSE
ILMN_2207419	DDX26B	FALSE	FALSE
ILMN_1776325	UBE2Q1	TRUE	FALSE
ILMN_1685327	NA	FALSE	FALSE
ILMN_1795495	NA	FALSE	FALSE
ILMN_1813091	ARL1	FALSE	FALSE
ILMN_1800465	NA	FALSE	FALSE
ILMN_1790216	NA	FALSE	FALSE
ILMN_1680899	NA	FALSE	FALSE
ILMN_1666078	HLA-H	TRUE	TRUE
ILMN_1705892	SH2D1A	FALSE	FALSE
ILMN_2154115	PSD4	FALSE	FALSE
ILMN_3224939	NA	FALSE	FALSE
ILMN_2402806	TRPC4AP	TRUE	TRUE
ILMN_1732182	FBXO44	FALSE	FALSE
ILMN_1743582	NUDT22	FALSE	FALSE
ILMN_1786734	EIF5	FALSE	FALSE
ILMN_2163790	NA	TRUE	FALSE
ILMN_1806705	ASB6	TRUE	FALSE
ILMN_1653185	PCSK7	FALSE	FALSE
ILMN_2149935	CC2D1B	FALSE	FALSE
ILMN_3258356	RPSAP52	FALSE	FALSE
ILMN_2053829	CBLN3	FALSE	FALSE
ILMN_1799198	OTUB2	FALSE	FALSE
ILMN_1796180	CRY2	TRUE	FALSE
ILMN_1776522	SLC50A1	FALSE	FALSE
ILMN_1754315	MMAA	FALSE	FALSE
ILMN_3246673	SLC35G6	FALSE	FALSE
ILMN_1773643	SLC38A9	FALSE	FALSE
ILMN_1795428	WDR59	FALSE	FALSE
ILMN_2368318	FGR	FALSE	FALSE
ILMN_1907100	NA	FALSE	FALSE
ILMN_1667086	AP3M1	FALSE	FALSE
ILMN_1754757	SCNN1D	FALSE	FALSE
ILMN_1752086	TRAPPC11	FALSE	FALSE

ILMN_1688515	ZNF195	FALSE	TRUE
ILMN_1678805	POMT2	FALSE	FALSE
ILMN_1657129	SKAP2	FALSE	TRUE
ILMN_1800082	B3GNT3	FALSE	FALSE
ILMN_1681631	NA	FALSE	FALSE
ILMN_1671005	IRF2BP2	FALSE	TRUE
ILMN_2392370	MYH14	FALSE	TRUE
ILMN_1669394	EI24	FALSE	FALSE
ILMN_1661424	THAP6	FALSE	FALSE
ILMN_1661519	NA	FALSE	FALSE
ILMN_1789909	TBC1D9B	FALSE	FALSE
ILMN_1713679	MREG	FALSE	FALSE
ILMN_1707077	SORT1	FALSE	FALSE
ILMN_3239950	KHDC1L	FALSE	FALSE
ILMN_1743427	SCYL3	FALSE	FALSE
ILMN_1694470	FOXL2NB	FALSE	FALSE
ILMN_1788347	CIPC	FALSE	FALSE
ILMN_1687375	ATP2A2	FALSE	FALSE
ILMN_1786606	NA	FALSE	FALSE
ILMN_1805225	LPCAT3	FALSE	FALSE
ILMN_1796075	VPS41	FALSE	FALSE
ILMN_1726387	NF1	FALSE	FALSE
ILMN_1765558	NPAS2	TRUE	FALSE
ILMN_1781636	VWA1	FALSE	FALSE
ILMN_2298365	PPP2R2B	FALSE	FALSE
ILMN_1745702	ACO2	FALSE	FALSE
ILMN_1656537	NA	FALSE	FALSE
ILMN_2142752	MANSC1	FALSE	FALSE
ILMN_1805833	FAM90A27P	FALSE	FALSE
ILMN_1783998	STARD5	FALSE	FALSE
ILMN_1655418	CAPNS1	FALSE	FALSE
ILMN_1825067	NA	FALSE	FALSE
ILMN_1741711	DOPEY2	FALSE	FALSE
ILMN_1651378	AUP1	FALSE	FALSE
ILMN_3234993	ROMO1	FALSE	FALSE
ILMN_1776925	PRSS22	FALSE	FALSE
ILMN_2394381	CLN3	FALSE	FALSE
ILMN_1726906	AKT1S1	FALSE	FALSE
ILMN_1673953	HRH1	FALSE	FALSE
ILMN_1807359	CLEC11A	FALSE	FALSE
ILMN_1675982	GGA3	FALSE	FALSE
ILMN_1756877	IFT43	FALSE	FALSE
ILMN_1752351	LAMP2	FALSE	FALSE
ILMN_2161746	TRIP10	FALSE	FALSE
ILMN_1651719	MBTPS1	FALSE	FALSE
ILMN_3257475	EI24	FALSE	FALSE

ILMN_1798346	KIAA1468	FALSE	FALSE
ILMN_1780898	PRKCH	FALSE	FALSE
ILMN_3258628	INPP5K	FALSE	FALSE
ILMN_1810467	PPP2R1A	FALSE	TRUE
ILMN_2045994	SEPW1	TRUE	TRUE
ILMN_1684168	GSTZ1	FALSE	FALSE
ILMN_1662961	GRHL3	FALSE	FALSE
ILMN_1809695	CAMK2G	TRUE	FALSE
ILMN_1812249	C15orf54	FALSE	FALSE
ILMN_1787378	ADD3	FALSE	FALSE
ILMN_3309699	MIR29B1	FALSE	FALSE
ILMN_1738821	GOLGA2	FALSE	FALSE
ILMN_2412807	DCTN1	FALSE	FALSE
ILMN_2189859	TMEM192	FALSE	FALSE
ILMN_1804938	TPRA1	FALSE	FALSE
ILMN_1679460	PPFIBP1	FALSE	FALSE
ILMN_1754114	FLJ20021	FALSE	FALSE
ILMN_1796106	MGAT4B	FALSE	FALSE
ILMN_1753819	NA	FALSE	FALSE
ILMN_2203463	SPATS2	FALSE	FALSE
ILMN_3242362	LINC00950	FALSE	FALSE
ILMN_1781987	CDK5	FALSE	FALSE
ILMN_1731248	NA	FALSE	FALSE
ILMN_1651819	GALNT11	FALSE	FALSE
ILMN_1656042	KIAA0319L	FALSE	FALSE
ILMN_2225595	ACAD8	FALSE	FALSE
ILMN_2217601	ANXA9	FALSE	FALSE
ILMN_2271894	ZNF654	FALSE	FALSE
ILMN_1660837	CLCN3	FALSE	FALSE
ILMN_1689059	ZNF329	TRUE	FALSE
ILMN_1787103	NA	FALSE	FALSE
ILMN_1808590	GUCY1A3	TRUE	FALSE
ILMN_1693397	PSG4	FALSE	FALSE
ILMN_2096372	ALDH1A1	TRUE	TRUE
ILMN_1706118	HN1L	FALSE	FALSE
ILMN_1795666	SNX24	FALSE	FALSE
ILMN_1769751	PIGG	TRUE	FALSE
ILMN_2225348	ZNF805	FALSE	FALSE
ILMN_1736863	TMEM140	TRUE	FALSE
ILMN_1660629	NBEAL2	FALSE	FALSE
ILMN_1707308	IKBKG	FALSE	FALSE
ILMN_1687303	ACAD10	FALSE	FALSE
ILMN_1730054	GSTT1	FALSE	FALSE
ILMN_3238221	FLJ42627	FALSE	FALSE
ILMN_3244456	NA	FALSE	FALSE
ILMN_1783709	RRAGA	FALSE	FALSE

ILMN_3281627	NA	FALSE	FALSE
ILMN_2359601	CAMK2G	FALSE	FALSE
ILMN_1700549	ERLIN2	TRUE	FALSE
ILMN_3242048	NA	FALSE	FALSE
ILMN_1795893	TMEM167B	TRUE	FALSE
ILMN_1692276	GGPS1	FALSE	FALSE
ILMN_2133784	PATE2	FALSE	FALSE
ILMN_1692267	B4GALT3	FALSE	FALSE
ILMN_1814606	GALNT2	FALSE	FALSE
ILMN_1693233	KIAA0513	FALSE	FALSE
ILMN_2362122	AP3M1	FALSE	FALSE
ILMN_1793563	DCTN1	FALSE	FALSE
ILMN_1804798	BEX4	FALSE	FALSE
ILMN_1831098	NA	FALSE	FALSE
ILMN_1751969	LOC440292	FALSE	FALSE
ILMN_1661220	TRIM16	FALSE	FALSE
ILMN_1859843	NA	FALSE	FALSE
ILMN_3310080	MIR614	FALSE	FALSE
ILMN_1715698	NA	FALSE	FALSE
ILMN_1683660	EIF3H	FALSE	FALSE
ILMN_1719286	CTSA	FALSE	TRUE
ILMN_1669281	CLN3	FALSE	FALSE
ILMN_1854861	NA	FALSE	FALSE
ILMN_1746031	RIMS4	FALSE	FALSE
ILMN_1651656	PSG7	FALSE	FALSE
ILMN_1810652	LMBRD2	FALSE	FALSE
ILMN_1698324	AVPR2	FALSE	FALSE
ILMN_2399140	RAB5C	FALSE	FALSE
ILMN_3255124	ATL1	FALSE	FALSE
ILMN_1764826	TFE3	FALSE	FALSE
ILMN_1685156	ADCY6	FALSE	FALSE
ILMN_1664698	UNC119	FALSE	FALSE
ILMN_1767811	ZNF416	FALSE	FALSE
ILMN_3248848	MLF2	FALSE	FALSE
ILMN_2409167	ANXA2	FALSE	FALSE
ILMN_1656393	PPP2R3A	FALSE	FALSE
ILMN_1743847	ACAP3	FALSE	FALSE
ILMN_1685709	TMEM125	FALSE	FALSE
ILMN_2251279	WDR45	FALSE	FALSE
ILMN_1785265	PLS3	FALSE	FALSE
ILMN_1805228	LRG1	FALSE	FALSE
ILMN_1716821	GORASP1	FALSE	TRUE
ILMN_2355463	CYFIP1	FALSE	FALSE
ILMN_1790810	HCN4	FALSE	FALSE
ILMN_1711909	EDEM2	FALSE	TRUE
ILMN_1682818	NA	TRUE	FALSE

ILMN_2297864	MTMR14	FALSE	FALSE
ILMN_1739821	EIF2S1	FALSE	FALSE
ILMN_1803988	MCL1	FALSE	TRUE
ILMN_1734880	RPL23AP53	FALSE	FALSE
ILMN_2399627	AP1G1	FALSE	FALSE
ILMN_1723542	ZNF137P	FALSE	FALSE
ILMN_1684368	SMIM3	FALSE	FALSE
ILMN_1673795	HSD17B4	FALSE	FALSE
ILMN_1689585	C20orf194	FALSE	FALSE
ILMN_1772702	SRSF8	TRUE	FALSE
ILMN_1704621	WDR44	FALSE	FALSE
ILMN_1755504	CALCOCO2	FALSE	FALSE
ILMN_1793729	C15orf39	FALSE	FALSE
ILMN_2413259	SOCS4	FALSE	FALSE
ILMN_2144574	CTBS	FALSE	FALSE
ILMN_2377991	ZSCAN31	FALSE	FALSE
ILMN_1687971	CAPN3	TRUE	FALSE
ILMN_1708303	CYP4F22	FALSE	FALSE
ILMN_1766045	SH3GLB1	FALSE	FALSE
ILMN_1795317	NA	FALSE	FALSE
ILMN_1743142	CERS3	FALSE	FALSE
ILMN_2362902	RASSF5	FALSE	FALSE
ILMN_1824192	NA	FALSE	FALSE
ILMN_1744822	BECN1	FALSE	FALSE
ILMN_1794038	FAM49A	FALSE	FALSE
ILMN_1738383	EEF2	FALSE	FALSE
ILMN_1759670	AMACR	FALSE	FALSE
ILMN_2331062	CBFA2T2	FALSE	FALSE
ILMN_1683300	BAX	FALSE	FALSE
ILMN_3249142	ZG16	FALSE	FALSE
ILMN_2397776	ASB6	FALSE	FALSE
ILMN_1655312	HPS5	FALSE	FALSE
ILMN_2119535	RIPK1	FALSE	FALSE
ILMN_1671748	NA	FALSE	FALSE
ILMN_3262542	LINC01011	FALSE	FALSE
ILMN_1708103	NA	FALSE	FALSE
ILMN_1904238	NA	FALSE	FALSE
ILMN_2128750	PTTG1IP	TRUE	FALSE
ILMN_1735432	ISCU	FALSE	FALSE
ILMN_1696391	LEPR	FALSE	FALSE
ILMN_1789418	NA	FALSE	FALSE
ILMN_2404539	TMEM230	FALSE	FALSE
ILMN_1757427	NA	FALSE	FALSE
ILMN_2307455	UBE2A	FALSE	FALSE
ILMN_1741572	AKAP8	FALSE	FALSE
ILMN_3282321	CDC42	FALSE	FALSE

ILMN_1665887	WDR61	FALSE	FALSE
ILMN_1799996	DHH	FALSE	FALSE
ILMN_1772894	TMEM27	TRUE	FALSE
ILMN_1779854	CARD6	FALSE	FALSE
ILMN_1760855	OCRL	FALSE	FALSE
ILMN_1898938	NA	FALSE	FALSE
ILMN_2370091	NGFRAP1	FALSE	FALSE
ILMN_3237507	ATXN7L3B	FALSE	FALSE
ILMN_1669352	LY6G6C	FALSE	FALSE
ILMN_1667519	RRAS2	FALSE	FALSE
ILMN_1686261	TOM1L2	FALSE	FALSE
ILMN_1735792	RHBDF2	FALSE	TRUE
ILMN_3241524	ZBTB22	FALSE	FALSE
ILMN_2408683	PPAP2B	FALSE	FALSE
ILMN_1799289	MRPL55	FALSE	FALSE
ILMN_2363273	ZNF226	FALSE	TRUE
ILMN_2399208	SCAMP3	FALSE	FALSE
ILMN_1771697	VRK3	FALSE	FALSE
ILMN_1739156	DOPEY1	FALSE	FALSE
ILMN_1682449	ZNF518B	TRUE	FALSE
ILMN_2333865	DNAJB12	TRUE	FALSE
ILMN_1713978	SDF2	FALSE	FALSE
ILMN_1705861	AP1M2	FALSE	FALSE
ILMN_2234016	FTH1	FALSE	FALSE
ILMN_3236774	NA	FALSE	FALSE
ILMN_1667319	LPPR2	TRUE	FALSE
ILMN_1712755	LRRC41	FALSE	FALSE
ILMN_2343097	NCALD	FALSE	FALSE
ILMN_1915616	NA	FALSE	FALSE
ILMN_3242967	NA	FALSE	FALSE
ILMN_1744912	CTTN	FALSE	FALSE
ILMN_1657429	SMAGP	FALSE	FALSE
ILMN_1764609	PWWP2B	FALSE	FALSE
ILMN_1703041	IDUA	TRUE	FALSE
ILMN_3253304	BRI3	FALSE	FALSE
ILMN_1683980	PLEKHM2	FALSE	FALSE
ILMN_1709227	CCDC84	FALSE	FALSE
ILMN_1768751	MTA3	FALSE	FALSE
ILMN_1916702	LINC00672	FALSE	FALSE
ILMN_1784286	NDUFA1	FALSE	FALSE
ILMN_2341548	MYO5B	FALSE	FALSE
ILMN_2366246	SEC23B	FALSE	FALSE
ILMN_1758542	BMP1	FALSE	FALSE
ILMN_1661888	MEF2A	FALSE	FALSE
ILMN_3225300	DYNC1I2	FALSE	TRUE
ILMN_2199313	NPDC1	FALSE	FALSE

ILMN_1789233	VPS37C	FALSE	FALSE
ILMN_1761242	COMMD1	FALSE	FALSE
ILMN_3257030	FTH1	FALSE	FALSE
ILMN_1730568	ZDHHC7	FALSE	FALSE
ILMN_1757561	MGC16025	FALSE	FALSE
ILMN_1796458	GABARAPL2	FALSE	FALSE
ILMN_1691476	MYLK	TRUE	FALSE
ILMN_1785060	TSPAN14	FALSE	FALSE
ILMN_1675674	UBE4B	FALSE	FALSE
ILMN_1756874	FBXO42	FALSE	FALSE
ILMN_2113340	AIM1L	FALSE	FALSE
ILMN_2201413	SLC37A2	FALSE	FALSE
ILMN_1756152	MFSD11	FALSE	FALSE
ILMN_1681812	HIF1AN	FALSE	FALSE
ILMN_1831826	NA	FALSE	FALSE
ILMN_2258363	KLC4	FALSE	FALSE
ILMN_1776119	ABCC10	FALSE	FALSE
ILMN_1740010	PCNX	FALSE	FALSE
ILMN_2163051	NA	FALSE	FALSE
ILMN_1777129	CENPT	FALSE	FALSE
ILMN_1781672	GAB1	FALSE	TRUE
ILMN_3268717	LOC146880	FALSE	FALSE
ILMN_2364110	GBA	FALSE	FALSE
ILMN_1750563	CERCAM	FALSE	FALSE
ILMN_2204876	FLVCR2	FALSE	FALSE
ILMN_2391150	FILIP1L	FALSE	FALSE
ILMN_1774708	ORMDL2	FALSE	FALSE
ILMN_3245452	FAM149B1	FALSE	FALSE
ILMN_1688452	LCMT1	FALSE	FALSE
ILMN_1804328	WWP1	FALSE	FALSE
ILMN_1895334	NA	FALSE	FALSE
ILMN_1689989	CDC42BPB	FALSE	FALSE
ILMN_1801377	SLC29A4	FALSE	FALSE
ILMN_2339627	COPE	FALSE	FALSE
ILMN_1744932	NA	FALSE	FALSE
ILMN_1652533	POM121L8P	FALSE	FALSE
ILMN_1802456	DCTD	FALSE	FALSE
ILMN_1651949	SCYL3	FALSE	FALSE
ILMN_1685365	ZNF773	FALSE	FALSE
ILMN_1654013	HID1	FALSE	FALSE
ILMN_1793854	INTS1	FALSE	FALSE
ILMN_1763265	CHMP1B	FALSE	FALSE
ILMN_1798061	ZFYVE26	FALSE	FALSE
ILMN_2345837	CLTA	FALSE	FALSE
ILMN_3236344	BMS1P4	FALSE	FALSE
ILMN_1788604	WBP2	FALSE	FALSE

ILMN_1668748	MED15	FALSE	FALSE
ILMN_1680388	LINC00989	FALSE	FALSE
ILMN_1670875	PPM1D	FALSE	FALSE
ILMN_1778951	C6orf203	FALSE	FALSE
ILMN_2111237	MN1	FALSE	FALSE
ILMN_1659437	TXNDC17	FALSE	FALSE
ILMN_1727135	FIBCD1	FALSE	FALSE
ILMN_2345564	TGM5	FALSE	FALSE
ILMN_1814856	CACFD1	FALSE	FALSE
ILMN_1765204	ST13	FALSE	FALSE
ILMN_1726666	GPX3	FALSE	FALSE
ILMN_2415011	DCTD	FALSE	FALSE
ILMN_1791222	GLYCTK	FALSE	FALSE
ILMN_2173294	THNSL2	FALSE	TRUE
ILMN_3235216	IFT20	FALSE	FALSE
ILMN_1798129	TRMT44	FALSE	FALSE
ILMN_1679194	UGT2B7	FALSE	FALSE
ILMN_2181125	NAPB	TRUE	FALSE
ILMN_1672526	LOC389834	FALSE	FALSE
ILMN_2331010	TNFRSF10B	FALSE	FALSE
ILMN_1778803	ZFAND6	FALSE	TRUE
ILMN_3307742	CAPS2	FALSE	FALSE
ILMN_1722809	NRCAM	FALSE	FALSE
ILMN_1697793	SYNJ2BP	FALSE	FALSE
ILMN_1657746	BPHL	FALSE	FALSE
ILMN_1706426	DSTN	FALSE	FALSE
ILMN_1737146	TRAM1	FALSE	FALSE
ILMN_1787885	NUDT18	FALSE	FALSE
ILMN_1752923	IFNAR1	FALSE	FALSE
ILMN_2402581	NA	FALSE	FALSE
ILMN_1697227	NA	FALSE	FALSE
ILMN_2390338	UBE2E3	FALSE	FALSE
ILMN_2106994	RABIF	FALSE	FALSE
ILMN_1669607	PHKG2	FALSE	FALSE
ILMN_1680054	LAMB3	FALSE	FALSE
ILMN_1691790	DACT2	FALSE	FALSE
ILMN_3236080	FAM26E	FALSE	FALSE
ILMN_2258004	METRNL	FALSE	FALSE
ILMN_2119692	CSAD	FALSE	FALSE
ILMN_3180557	CYB561D1	FALSE	TRUE
ILMN_1765523	TOLLIP	TRUE	FALSE
ILMN_2053536	RHBDL2	FALSE	FALSE
ILMN_2109536	NA	FALSE	FALSE
ILMN_2264029	KLK5	FALSE	FALSE
ILMN_3274339	NA	FALSE	FALSE
ILMN_1894388	NA	FALSE	FALSE

ILMN_1680693	ZNF419	FALSE	FALSE
ILMN_2371251	MPG	FALSE	FALSE
ILMN_1671149	MEG3	FALSE	FALSE
ILMN_2405628	TOP1MT	FALSE	FALSE
ILMN_1803313	NA	FALSE	FALSE
ILMN_2103362	ARHGAP27	FALSE	FALSE
ILMN_1801941	C1orf50	FALSE	FALSE
ILMN_2392286	IP6K1	FALSE	FALSE
ILMN_2138589	MERTK	FALSE	FALSE
ILMN_1800871	RAB6A	FALSE	FALSE
ILMN_1678579	CPT2	FALSE	FALSE
ILMN_1746704	TRIM8	FALSE	FALSE
ILMN_3271587	NA	FALSE	FALSE
ILMN_2105966	SLC35A4	FALSE	FALSE
ILMN_1688702	PJA2	FALSE	FALSE
ILMN_1722329	CASQ2	FALSE	FALSE
ILMN_2396148	HIP1R	FALSE	FALSE
ILMN_1683231	FAM83F	FALSE	FALSE
ILMN_1726434	UNC45A	FALSE	FALSE
ILMN_1731433	AOC1	TRUE	FALSE
ILMN_1757262	ZBTB5	FALSE	FALSE
ILMN_1662896	WDR11	FALSE	FALSE
ILMN_3188106	CYTH2	FALSE	FALSE
ILMN_1652762	HIC2	FALSE	FALSE
ILMN_1701457	FAHD1	FALSE	FALSE
ILMN_1769721	SP6	FALSE	FALSE
ILMN_1661594	C2orf42	FALSE	FALSE
ILMN_1711862	RNF7	FALSE	FALSE
ILMN_2064694	STIM1	FALSE	FALSE
ILMN_3240187	EMC3	FALSE	TRUE
ILMN_2363127	MROH8	FALSE	FALSE
ILMN_1718171	MED26	FALSE	FALSE
ILMN_1671154	TMPRSS13	FALSE	FALSE
ILMN_1667594	KLF10	FALSE	TRUE
ILMN_2406267	MARVELD2	FALSE	FALSE
ILMN_1657744	FAM219B	FALSE	FALSE
ILMN_1670652	CYP2C9	FALSE	FALSE
ILMN_2384181	DHRS9	FALSE	FALSE
ILMN_3263575	NA	FALSE	FALSE
ILMN_1687896	PIK3C3	FALSE	FALSE
ILMN_1658498	SLC44A3	FALSE	FALSE
ILMN_2388800	PPAP2B	FALSE	FALSE
ILMN_1705403	CYP2S1	TRUE	FALSE
ILMN_1709348	ALDH1A1	FALSE	TRUE
ILMN_1686929	GPATCH3	FALSE	FALSE
ILMN_1731014	HEMK1	FALSE	FALSE

ILMN_1753265	C12orf76	TRUE	FALSE
ILMN_1698225	MYO5A	FALSE	FALSE
ILMN_3276016	WWP1	FALSE	FALSE
ILMN_1709809	NHP2L1	FALSE	FALSE
ILMN_1697200	MON2	FALSE	FALSE
ILMN_1678730	NOMO1	FALSE	FALSE
ILMN_1667429	SLC25A20	FALSE	FALSE
ILMN_3238375	CENPT	FALSE	FALSE
ILMN_2093231	NA	FALSE	FALSE
ILMN_1771832	PARP6	FALSE	FALSE
ILMN_1698072	PITRM1	FALSE	FALSE
ILMN_1909770	NA	FALSE	FALSE
ILMN_2096405	WDR37	FALSE	FALSE
ILMN_2198893	LOC407835	FALSE	FALSE
ILMN_2128623	NA	FALSE	FALSE
ILMN_1854015	NA	FALSE	FALSE
ILMN_3243972	SNORA70B	FALSE	FALSE
ILMN_1768480	VGLL4	FALSE	FALSE
ILMN_3201614	NA	FALSE	FALSE
ILMN_2187533	CSRNP2	FALSE	FALSE
ILMN_1734830	MTHFR	TRUE	FALSE
ILMN_1735788	TRIOBP	FALSE	FALSE
ILMN_1723768	NLRX1	FALSE	FALSE
ILMN_1805104	ABAT	TRUE	FALSE
ILMN_1792671	C12orf29	FALSE	FALSE
ILMN_1711766	SKP1	FALSE	FALSE
ILMN_1671728	CARD14	FALSE	FALSE
ILMN_1720771	STX11	FALSE	FALSE
ILMN_1668559	RGS10	FALSE	FALSE
ILMN_2330845	NSF	FALSE	FALSE
ILMN_3243414	NA	FALSE	FALSE
ILMN_1804908	NA	FALSE	FALSE
ILMN_1672356	NA	FALSE	FALSE
ILMN_1747146	TSG101	FALSE	FALSE
ILMN_2366714	UQCR10	FALSE	TRUE
ILMN_1772329	LRRFIP2	FALSE	FALSE
ILMN_1792587	VPS4B	FALSE	FALSE
ILMN_1789732	TMEM189	TRUE	FALSE
ILMN_2410262	MTMR14	FALSE	FALSE
ILMN_2413572	MARK2	FALSE	FALSE
ILMN_3293972	NA	FALSE	FALSE
ILMN_1796749	KIF1C	FALSE	FALSE
ILMN_2109994	NA	FALSE	FALSE
ILMN_3302919	MYOF	FALSE	FALSE
ILMN_1798838	CTTN	FALSE	FALSE
ILMN_1794659	FUT2	FALSE	FALSE

ILMN_1737517	RPL29	FALSE	FALSE
ILMN_1759023	WFS1	TRUE	FALSE
ILMN_1706813	SLC6A14	FALSE	FALSE
ILMN_2374115	TFAP2A	FALSE	TRUE
ILMN_1748803	ZDHHC9	FALSE	FALSE
ILMN_1657983	TERF2IP	TRUE	FALSE
ILMN_1836469	NA	FALSE	FALSE
ILMN_1791069	ZNF513	FALSE	FALSE
ILMN_1869119	NA	FALSE	FALSE
ILMN_2289924	TRAK1	FALSE	FALSE
ILMN_1676689	PPT2	FALSE	FALSE
ILMN_2390227	TBC1D9B	FALSE	FALSE
ILMN_1661755	FAM129B	FALSE	FALSE
ILMN_1696702	NEO1	FALSE	FALSE
ILMN_1699852	CES4A	FALSE	FALSE
ILMN_1731180	EXOC2	FALSE	FALSE
ILMN_1728349	TMEM63B	FALSE	FALSE
ILMN_1907467	NA	FALSE	FALSE
ILMN_3247645	LINC01420	FALSE	FALSE
ILMN_1713990	TRIP6	FALSE	FALSE
ILMN_1768577	PCSK6	FALSE	FALSE
ILMN_3236599	LINC00452	FALSE	FALSE
ILMN_1774828	VEZT	FALSE	FALSE
ILMN_2367671	LIMK2	FALSE	FALSE
ILMN_1789243	VPS33B	FALSE	FALSE
ILMN_1751492	NA	FALSE	FALSE
ILMN_1773407	C16orf72	FALSE	FALSE
ILMN_2389376	DCAF11	FALSE	FALSE
ILMN_2379226	CCNL2	FALSE	FALSE
ILMN_3310730	MIR1277	FALSE	FALSE
ILMN_2372398	ALDH5A1	FALSE	FALSE
ILMN_2152502	MGC72080	FALSE	FALSE
ILMN_1813139	ANKDD1A	FALSE	FALSE
ILMN_1739847	EIF3D	FALSE	FALSE
ILMN_1796663	B4GALNT4	FALSE	FALSE
ILMN_3239378	TADA2B	FALSE	FALSE
ILMN_1693039	ZFPL1	FALSE	FALSE
ILMN_1712236	PPP1R3B	FALSE	FALSE
ILMN_2100815	TMEM9B	FALSE	FALSE
ILMN_1743621	CRHR1-IT1	FALSE	FALSE
ILMN_2187487	HEATR5B	FALSE	FALSE
ILMN_1875510	NA	FALSE	FALSE
ILMN_1739882	SDR9C7	FALSE	FALSE
ILMN_1661733	FOLR1	FALSE	FALSE
ILMN_3271092	PPP1R21	FALSE	FALSE
ILMN_1728698	GDE1	FALSE	FALSE

ILMN_1724666	NA	FALSE	FALSE
ILMN_1657483	SEC23B	FALSE	FALSE
ILMN_1792182	RDH12	FALSE	FALSE
ILMN_1749009	REXO2	FALSE	FALSE
ILMN_2336186	LCMT1	FALSE	FALSE
ILMN_1813423	NAA60	FALSE	FALSE
ILMN_3293146	TMEM230	FALSE	FALSE
ILMN_1692260	MAFG	FALSE	FALSE
ILMN_1770030	C7orf43	FALSE	FALSE
ILMN_1776953	MYL9	FALSE	FALSE
ILMN_1756006	ATG2A	FALSE	FALSE
ILMN_1663173	STAG3L2	FALSE	FALSE
ILMN_1675684	APOBEC3C	FALSE	FALSE
ILMN_1658773	PIGF	FALSE	FALSE
ILMN_1807600	NPLOC4	FALSE	FALSE
ILMN_1771051	RPL29	FALSE	FALSE
ILMN_2192385	TTC19	FALSE	FALSE
ILMN_1868866	NA	FALSE	FALSE
ILMN_1785919	NA	FALSE	FALSE
ILMN_1727393	NA	FALSE	FALSE
ILMN_1735553	MAP3K9	FALSE	FALSE
ILMN_3209180	NA	FALSE	FALSE
ILMN_1685005	TNFRSF1A	TRUE	FALSE
ILMN_1676005	KPNA1	FALSE	FALSE
ILMN_1774427	CALCOCO1	TRUE	FALSE
ILMN_1764500	BRK1	FALSE	FALSE
ILMN_1683916	PEX13	FALSE	TRUE
ILMN_1679809	GSTP1	FALSE	FALSE
ILMN_1655876	TMEM159	TRUE	FALSE
ILMN_1796712	S100A10	FALSE	FALSE
ILMN_2190414	ZNF83	FALSE	FALSE
ILMN_1668514	PIP5K1C	FALSE	FALSE
ILMN_1756525	KLHL20	FALSE	FALSE
ILMN_1753439	RPTN	FALSE	FALSE
ILMN_1704253	C6orf106	TRUE	FALSE
ILMN_1803624	TMEM147	FALSE	FALSE
ILMN_1720430	PPDPF	FALSE	FALSE
ILMN_1788118	SLC23A3	FALSE	FALSE
ILMN_2124386	RGL2	FALSE	FALSE
ILMN_1697559	G6PD	FALSE	FALSE
ILMN_1703593	BAIAP2L1	FALSE	FALSE
ILMN_1832425	NA	FALSE	FALSE
ILMN_1343295	GAPDH	FALSE	FALSE
ILMN_1776188	MAP1LC3A	FALSE	FALSE
ILMN_3236858	NYNRIN	TRUE	FALSE
ILMN_2332795	ZNF16	FALSE	FALSE

ILMN_1781431	GLCCI1	FALSE	FALSE
ILMN_2379718	RAB24	FALSE	FALSE
ILMN_1651826	BASP1	FALSE	FALSE
ILMN_2352009	ACADVL	FALSE	FALSE
ILMN_1651254	LPP	FALSE	FALSE
ILMN_1686082	C14orf79	FALSE	FALSE
ILMN_1909073	NA	FALSE	FALSE
ILMN_1784863	CD36	FALSE	FALSE
ILMN_1767992	SLC12A6	FALSE	FALSE
ILMN_1727815	CFI	FALSE	FALSE
ILMN_1803256	STOX2	FALSE	FALSE
ILMN_1808356	FAM3A	FALSE	FALSE
ILMN_2347805	EXOC1	FALSE	FALSE
ILMN_1677919	GMPR2	FALSE	TRUE
ILMN_1804148	TMED4	FALSE	FALSE
ILMN_1651967	TP53I3	FALSE	FALSE
ILMN_1750805	ARHGAP30	FALSE	FALSE
ILMN_2153373	LRBA	FALSE	FALSE
ILMN_1755897	UGT2B7	FALSE	FALSE
ILMN_2349071	GPR64	FALSE	FALSE
ILMN_1748983	RTN4	FALSE	FALSE
ILMN_1793743	DIRC2	FALSE	TRUE
ILMN_1745655	PEX16	FALSE	FALSE
ILMN_1671809	DUSP22	TRUE	FALSE
ILMN_1717809	RNF24	TRUE	TRUE
ILMN_1739792	RHOG	FALSE	FALSE
ILMN_3213098	NA	FALSE	FALSE
ILMN_1773561	RAP2C	FALSE	FALSE
ILMN_1732873	IPPK	FALSE	FALSE
ILMN_3244516	HECTD4	FALSE	FALSE
ILMN_1702430	SOX30	FALSE	FALSE
ILMN_1752197	PDDC1	FALSE	FALSE
ILMN_1679178	ATP5D	FALSE	FALSE
ILMN_1722218	MBOAT7	FALSE	TRUE
ILMN_1754658	NA	FALSE	FALSE
ILMN_1768271	SMAP1	FALSE	FALSE
ILMN_1784967	EPB41L4B	FALSE	FALSE
ILMN_2206126	RAET1L	FALSE	FALSE
ILMN_1679614	SGSM3	FALSE	FALSE
ILMN_1744006	GFOD2	FALSE	FALSE
ILMN_1751345	AP1S1	FALSE	FALSE
ILMN_2317730	ELMO2	TRUE	TRUE
ILMN_1762718	CMTM4	FALSE	FALSE
ILMN_1913021	NA	FALSE	FALSE
ILMN_3206429	CTBP2	FALSE	FALSE
ILMN_3243351	LOC646214	FALSE	FALSE

ILMN_1807423	IGF2BP3	FALSE	FALSE
ILMN_1694514	ZDHHC11	TRUE	FALSE
ILMN_1669747	UNC13B	FALSE	FALSE
ILMN_2133090	SDR9C7	FALSE	FALSE
ILMN_2404182	DUOX1	TRUE	FALSE
ILMN_1716524	RAB7A	TRUE	FALSE
ILMN_1745112	FAM102A	FALSE	FALSE
ILMN_1713402	FAM160A2	FALSE	FALSE
ILMN_1692121	USO1	FALSE	FALSE
ILMN_1803953	LINC00493	FALSE	FALSE
ILMN_1679232	KIDINS220	FALSE	FALSE
ILMN_1763640	NCKAP5L	FALSE	FALSE
ILMN_1773847	DYNC1I2	FALSE	FALSE
ILMN_1810431	NA	FALSE	FALSE
ILMN_3282436	EIF3F	TRUE	FALSE
ILMN_1801632	KRT5	FALSE	FALSE
ILMN_1813491	SPTLC1	FALSE	FALSE
ILMN_2353143	FBLIM1	FALSE	FALSE
ILMN_1713174	TCP11L1	FALSE	FALSE
ILMN_1799604	OCIAD1	TRUE	FALSE
ILMN_1798254	ACTR10	FALSE	FALSE
ILMN_1680353	NSF	FALSE	FALSE
ILMN_2383975	PRDX5	FALSE	FALSE
ILMN_1826531	ATXN7L3B	FALSE	FALSE
ILMN_1841620	NA	FALSE	FALSE
ILMN_1672728	KCTD5	FALSE	FALSE
ILMN_3307944	PHLDB3	FALSE	FALSE
ILMN_2225537	PTGR1	FALSE	FALSE
ILMN_1789419	EXOC3	FALSE	FALSE
ILMN_1775114	ENTPD3	TRUE	FALSE
ILMN_1679838	WBP5	FALSE	FALSE
ILMN_1711166	WRAP73	FALSE	FALSE
ILMN_1745784	ZNF324	FALSE	TRUE
ILMN_1706434	YBX3P1	FALSE	FALSE
ILMN_1727332	ATPIF1	FALSE	FALSE
ILMN_1793203	MIEF1	FALSE	TRUE
ILMN_1724946	SPINT1	FALSE	FALSE
ILMN_1680687	NSF	FALSE	FALSE
ILMN_1803219	TMC4	FALSE	FALSE
ILMN_2326273	CHI3L2	FALSE	FALSE
ILMN_1794588	DYRK2	FALSE	FALSE
ILMN_1708340	DAPK1	FALSE	FALSE
ILMN_3187357	NA	FALSE	FALSE
ILMN_1743476	LOC729609	FALSE	FALSE
ILMN_1739199	WASH3P	TRUE	FALSE
ILMN_1757370	SMPD1	FALSE	FALSE

ILMN_1789702	GBE1	FALSE	FALSE
ILMN_1773153	SHC1	FALSE	FALSE
ILMN_1756238	TMEM217	FALSE	FALSE
ILMN_1672940	ZNF562	FALSE	FALSE
ILMN_1678477	C9orf85	FALSE	FALSE
ILMN_1769027	CDC42SE1	FALSE	FALSE
ILMN_1758963	NADK	FALSE	FALSE
ILMN_1693341	NA	FALSE	TRUE
ILMN_2230566	RAB40B	FALSE	FALSE
ILMN_3236239	LOC100129550	TRUE	TRUE
ILMN_1677314	MUC1	FALSE	FALSE
ILMN_1725534	ACTN4	FALSE	FALSE
ILMN_2319588	OSGIN1	FALSE	FALSE
ILMN_1892638	NA	FALSE	FALSE
ILMN_1678546	PEX11B	TRUE	FALSE
ILMN_2140389	TMEM185A	FALSE	FALSE
ILMN_2403946	FEZ2	FALSE	FALSE
ILMN_1801852	SEC61B	FALSE	FALSE
ILMN_1676358	RALB	FALSE	FALSE
ILMN_1788961	PPP2R2A	FALSE	FALSE
ILMN_2095133	SPTAN1	TRUE	TRUE
ILMN_3235723	TMPRSS11E	FALSE	FALSE
ILMN_1718866	C5orf46	FALSE	FALSE
ILMN_1707336	ARPC4	FALSE	FALSE
ILMN_2122420	HMGCL	FALSE	FALSE
ILMN_2318638	TGIF1	FALSE	TRUE
ILMN_1679693	TMEM87B	FALSE	FALSE
ILMN_1700159	NIPSNAP3A	FALSE	FALSE
ILMN_2384561	TJP2	FALSE	FALSE
ILMN_1734229	SPPL2A	FALSE	FALSE
ILMN_1808202	R3HDM4	FALSE	FALSE
ILMN_3240962	DDRGK1	FALSE	TRUE
ILMN_1789751	MFSD1	FALSE	FALSE
ILMN_1679600	ACOT8	FALSE	FALSE
ILMN_1700660	RNF135	FALSE	FALSE
ILMN_1738642	CMPK1	FALSE	FALSE
ILMN_3273340	AGAP2-AS1	FALSE	FALSE
ILMN_1780268	C1orf56	FALSE	FALSE
ILMN_1751984	PRKAG1	TRUE	FALSE
ILMN_2321292	WIPI2	FALSE	FALSE
ILMN_1763386	BID	FALSE	FALSE
ILMN_1741942	STX16	TRUE	TRUE
ILMN_1675055	MUL1	FALSE	TRUE
ILMN_3244963	DCAF8	FALSE	FALSE
ILMN_2354381	PON2	FALSE	FALSE
ILMN_1808095	RNF41	FALSE	FALSE

ILMN_1719344	NRBF2	TRUE	FALSE
ILMN_1658351	FIS1	FALSE	FALSE
ILMN_1811195	ZNF211	TRUE	TRUE
ILMN_1900110	NA	FALSE	FALSE
ILMN_1786843	KCTD13	FALSE	FALSE
ILMN_1746135	PHF23	FALSE	FALSE
ILMN_1815812	CDIP1	FALSE	FALSE
ILMN_1684440	PXN	FALSE	FALSE
ILMN_1769934	GRHL1	FALSE	FALSE
ILMN_1753805	PRKD2	FALSE	FALSE
ILMN_1739586	FEZ2	FALSE	FALSE
ILMN_1743187	C6orf120	FALSE	FALSE
ILMN_1660341	LRPAP1	TRUE	TRUE
ILMN_1712721	GAST	FALSE	FALSE
ILMN_1759915	ARPC1A	FALSE	FALSE
ILMN_3251317	RER1	FALSE	FALSE
ILMN_1703866	SUPT5H	FALSE	FALSE
ILMN_1711124	NA	FALSE	FALSE
ILMN_1685012	EAF1	FALSE	FALSE
ILMN_1723847	CILP	FALSE	FALSE
ILMN_1679277	CMTM3	FALSE	FALSE
ILMN_1690826	TNKS1BP1	FALSE	FALSE
ILMN_3253126	GAS6-AS1	FALSE	FALSE
ILMN_1763491	CKMT1B	FALSE	FALSE
ILMN_1775327	PKM	FALSE	FALSE
ILMN_3301749	SPNS2	FALSE	FALSE
ILMN_2055634	TIAF1	FALSE	FALSE
ILMN_1654441	OAT	FALSE	FALSE
ILMN_1782389	LAD1	FALSE	FALSE
ILMN_2233050	PLA2G2D	FALSE	FALSE
ILMN_1656621	CHMP2A	FALSE	FALSE
ILMN_2056032	CD99	FALSE	FALSE
ILMN_1656656	COX19	FALSE	FALSE
ILMN_1667432	HYAL3	FALSE	FALSE
ILMN_3252665	C6orf132	FALSE	FALSE
ILMN_2230902	CTNNA1	FALSE	FALSE
ILMN_1689306	TMC7	FALSE	FALSE
ILMN_1781745	C9orf152	FALSE	FALSE
ILMN_1697880	PLD2	FALSE	FALSE
ILMN_1800733	MANBA	FALSE	FALSE
ILMN_1736700	ALDOA	FALSE	FALSE
ILMN_2059535	PPM1F	FALSE	FALSE
ILMN_1752582	RAB5B	FALSE	FALSE
ILMN_2358382	ZFYVE1	FALSE	FALSE
ILMN_1675354	NA	FALSE	FALSE
ILMN_1722294	CPNE8	FALSE	TRUE

ILMN_3308728	MIR635	FALSE	FALSE
ILMN_1679051	PTPRR	FALSE	FALSE
ILMN_1697971	TRIM38	FALSE	FALSE
ILMN_2173004	RAB8B	FALSE	FALSE
ILMN_1739751	SLC26A11	FALSE	FALSE
ILMN_1683827	NA	FALSE	FALSE
ILMN_1774265	C2orf82	FALSE	FALSE
ILMN_1684051	WASF2	FALSE	FALSE
ILMN_1797367	TSC1	TRUE	FALSE
ILMN_1695962	SLC12A9	TRUE	FALSE
ILMN_1870457	NA	FALSE	FALSE
ILMN_1854349	NA	FALSE	FALSE
ILMN_1703288	TMEM259	FALSE	FALSE
ILMN_1661537	LEPROT	FALSE	FALSE
ILMN_3251269	SRP14	FALSE	FALSE
ILMN_1728965	C11orf57	FALSE	FALSE
ILMN_1875342	NA	TRUE	FALSE
ILMN_1889229	NA	FALSE	FALSE
ILMN_2112524	RNF149	FALSE	FALSE
ILMN_1760718	ZMIZ2	FALSE	FALSE
ILMN_1807596	UBAP1	FALSE	FALSE
ILMN_2068747	OAT	FALSE	FALSE
ILMN_1663618	STAT3	FALSE	FALSE
ILMN_1726169	EDF1	FALSE	FALSE
ILMN_1714352	DMWD	TRUE	TRUE
ILMN_2064655	CXorf40A	FALSE	FALSE
ILMN_2046896	ESRRA	FALSE	FALSE
ILMN_2206344	NDST2	TRUE	FALSE
ILMN_2393254	CAPNS1	FALSE	FALSE
ILMN_1763127	ACKR2	FALSE	FALSE
ILMN_1699112	COPB1	FALSE	FALSE
ILMN_2068991	NA	FALSE	FALSE
ILMN_2226304	ANKRD50	TRUE	FALSE
ILMN_2311779	TMUB2	FALSE	FALSE
ILMN_1742618	XAF1	FALSE	FALSE
ILMN_3283141	LOC643802	FALSE	FALSE
ILMN_1769259	ANO9	FALSE	FALSE
ILMN_1789575	GJB3	FALSE	TRUE
ILMN_1733970	BAG1	FALSE	FALSE
ILMN_1682783	TUG1	FALSE	FALSE
ILMN_3296181	NA	FALSE	FALSE
ILMN_1808584	COX20	FALSE	FALSE
ILMN_2399392	SIL1	FALSE	FALSE
ILMN_1803819	IQGAP1	FALSE	TRUE
ILMN_1701455	FBXO6	FALSE	FALSE
ILMN_1685095	BLOC1S4	FALSE	FALSE

ILMN_1686623	CSF1R	FALSE	FALSE
ILMN_1687626	ZDHHC24	FALSE	FALSE
ILMN_1750158	ACOX1	FALSE	FALSE
ILMN_1710954	FBXL19-AS1	FALSE	FALSE
ILMN_1674394	APMAP	FALSE	TRUE
ILMN_1809099	IL33	FALSE	FALSE
ILMN_1672461	SPPL2B	FALSE	FALSE
ILMN_2339779	ATP6V1E1	FALSE	FALSE
ILMN_1766261	SLC2A12	FALSE	FALSE
ILMN_1826471	NA	FALSE	FALSE
ILMN_1696021	KPNA6	FALSE	FALSE
ILMN_2124082	PPP2R5B	FALSE	FALSE
ILMN_1759460	TAF7	FALSE	FALSE
ILMN_3247256	RPL7L1	FALSE	FALSE
ILMN_1661173	TRIP4	FALSE	FALSE
ILMN_1758895	CTSK	TRUE	FALSE
ILMN_1746948	MYL5	TRUE	FALSE
ILMN_1706015	FAM43A	TRUE	FALSE
ILMN_1729208	NGFRAP1	FALSE	FALSE
ILMN_1801040	SPN	FALSE	FALSE
ILMN_1773742	DNAJB9	FALSE	FALSE
ILMN_2377019	CORO1B	FALSE	FALSE
ILMN_2119224	KIFAP3	FALSE	FALSE
ILMN_1815130	MICALL1	FALSE	FALSE
ILMN_1663160	ZNF337	FALSE	FALSE
ILMN_1802252	GAPDH	FALSE	FALSE
ILMN_1757646	UFM1	FALSE	FALSE
ILMN_1779965	AK1	FALSE	FALSE
ILMN_1719303	P4HB	FALSE	FALSE
ILMN_2143261	CXorf40B	FALSE	FALSE
ILMN_1786105	PCBD1	FALSE	FALSE
ILMN_1784105	SLC38A7	FALSE	FALSE
ILMN_1788315	SIN3B	FALSE	FALSE
ILMN_1799614	PNPLA6	FALSE	FALSE
ILMN_1689712	RPLP1	FALSE	FALSE
ILMN_1695432	TPST2	FALSE	FALSE
ILMN_1765801	GAA	FALSE	FALSE
ILMN_1749368	HIST1H3H	FALSE	FALSE
ILMN_1675523	PPAP2C	FALSE	FALSE
ILMN_2041788	PLS3	FALSE	FALSE
ILMN_1667356	PPP1R21	FALSE	FALSE
ILMN_1741727	QPCT	FALSE	FALSE
ILMN_1788942	GGT6	FALSE	FALSE
ILMN_1782618	C9orf16	FALSE	FALSE
ILMN_1658261	MAPKBP1	FALSE	FALSE
ILMN_1752333	SLC35E1	FALSE	FALSE

ILMN_1866887	NA	FALSE	FALSE
ILMN_1730032	BOK	FALSE	FALSE
ILMN_1725620	GGNBP2	FALSE	FALSE
ILMN_3249101	SDR9C7	FALSE	FALSE
ILMN_1773906	NCOA4	TRUE	FALSE
ILMN_2401873	DUSP10	TRUE	TRUE
ILMN_3249658	LOC100134868	FALSE	FALSE
ILMN_1769787	SELO	FALSE	FALSE
ILMN_1701466	PEX16	FALSE	FALSE
ILMN_1701487	TAOK2	FALSE	FALSE
ILMN_1674560	GBA2	FALSE	FALSE
ILMN_1719517	CTTNBP2NL	FALSE	FALSE
ILMN_2399622	AP1G1	FALSE	FALSE
ILMN_2355462	CYFIP1	FALSE	FALSE
ILMN_3300198	NA	TRUE	FALSE
ILMN_1855230	NA	FALSE	FALSE
ILMN_1715647	VANGL2	FALSE	FALSE
ILMN_3245907	FAM160B2	FALSE	FALSE
ILMN_1793831	TRADD	FALSE	FALSE
ILMN_3244521	LINC00294	TRUE	FALSE
ILMN_1673548	LGALSL	FALSE	FALSE
ILMN_2057768	SLC35D1	FALSE	FALSE
ILMN_1809467	VAMP5	FALSE	FALSE
ILMN_1756146	WDR45	FALSE	FALSE
ILMN_1683277	KIAA0319L	FALSE	FALSE
ILMN_3262439	NA	FALSE	FALSE
ILMN_1684956	ARSD	FALSE	FALSE
ILMN_1702322	ALS2CL	TRUE	FALSE
ILMN_2211950	SRP14	FALSE	FALSE
ILMN_1786469	FBXO22	FALSE	FALSE
ILMN_2388874	GRHL3	FALSE	FALSE
ILMN_1657624	NSFL1C	FALSE	FALSE
ILMN_1789839	GTF3C1	FALSE	FALSE
ILMN_1688639	FBXL2	FALSE	FALSE
ILMN_1767651	TECPR1	FALSE	FALSE
ILMN_1742456	OSTF1	FALSE	FALSE
ILMN_2359453	ERGIC3	TRUE	TRUE
ILMN_1770425	CDIPT	FALSE	FALSE
ILMN_1704713	CSNK1G1	FALSE	FALSE
ILMN_1663407	SURF1	FALSE	FALSE
ILMN_3246783	TXLNGY	FALSE	FALSE
ILMN_1719543	MAF	TRUE	FALSE
ILMN_1652128	LMBRD1	FALSE	FALSE
ILMN_1701967	SHC1	FALSE	FALSE
ILMN_1690920	SP100	FALSE	FALSE
ILMN_2402805	TRPC4AP	FALSE	TRUE

ILMN_1777683	ADAMTSL4	FALSE	FALSE
ILMN_1692199	RNF103	FALSE	FALSE
ILMN_1746494	FNTA	FALSE	TRUE
ILMN_2381197	RNF19A	FALSE	TRUE
ILMN_1807277	IFI30	FALSE	FALSE
ILMN_1755383	LRRC1	FALSE	FALSE
ILMN_1762764	SH3BGRL2	FALSE	FALSE
ILMN_2093748	ZNF669	FALSE	FALSE
ILMN_1659490	NA	FALSE	FALSE
ILMN_1695679	NWD2	FALSE	TRUE
ILMN_1672843	FBXO8	FALSE	TRUE
ILMN_3199438	NA	FALSE	FALSE
ILMN_3295735	FRAT1	FALSE	FALSE
ILMN_3275771	FTH1	FALSE	FALSE
ILMN_1786396	ZZEF1	FALSE	FALSE
ILMN_1795838	C4orf19	FALSE	FALSE
ILMN_3251379	SLC35E1	FALSE	FALSE
ILMN_2341815	TFG	FALSE	FALSE
ILMN_1804673	SLC16A4	FALSE	FALSE
ILMN_1719883	CYP4F11	FALSE	FALSE
ILMN_3307158	ATG4A	FALSE	FALSE
ILMN_1807873	SNX6	FALSE	FALSE
ILMN_1654016	MYL12B	FALSE	FALSE
ILMN_1769665	RAB5C	FALSE	FALSE
ILMN_1756126	STUB1	FALSE	FALSE
ILMN_1762876	DSCR4	FALSE	FALSE
ILMN_1780504	C19orf26	FALSE	FALSE
ILMN_1776109	ZNF622	FALSE	FALSE
ILMN_1787265	ZNF503	TRUE	FALSE
ILMN_1776088	NAT9	FALSE	FALSE
ILMN_1714945	ITPKC	FALSE	FALSE
ILMN_1729175	FBXO3	FALSE	FALSE
ILMN_1803995	TM7SF3	FALSE	FALSE
ILMN_1808938	PIGF	FALSE	FALSE
ILMN_1651347	SERTAD2	TRUE	FALSE
ILMN_1751328	FAM83H	FALSE	FALSE
ILMN_1745772	ASCC3	FALSE	FALSE
ILMN_1683044	PPP1R2	FALSE	TRUE
ILMN_1665831	CLPTM1	FALSE	TRUE
ILMN_1806010	CUL9	FALSE	TRUE
ILMN_2400407	CNTN1	FALSE	FALSE
ILMN_2346573	PSME3	FALSE	FALSE
ILMN_1746378	GHDC	FALSE	FALSE
ILMN_1785765	TM9SF2	FALSE	FALSE
ILMN_3241979	TMEM179B	TRUE	FALSE
ILMN_1777061	ZSWIM6	FALSE	FALSE

ILMN_1697529	RNF10	FALSE	FALSE
ILMN_1917341	PTPN14	FALSE	FALSE
ILMN_3238707	SNORA8	TRUE	FALSE
ILMN_1751887	PREP	FALSE	FALSE
ILMN_1677963	TMCC1	FALSE	FALSE
ILMN_1717594	AP5B1	FALSE	FALSE
ILMN_1912083	NA	FALSE	FALSE
ILMN_2157951	STX6	FALSE	FALSE
ILMN_2401878	DUSP10	TRUE	TRUE
ILMN_2324561	SLC7A6	FALSE	FALSE
ILMN_1722726	ARHGAP32	FALSE	FALSE
ILMN_1775823	POFUT2	TRUE	FALSE
ILMN_1655748	ZSCAN31	FALSE	FALSE
ILMN_3264466	MTFR1L	FALSE	FALSE
ILMN_3230241	NA	FALSE	FALSE
ILMN_2268068	MAPKAP1	FALSE	FALSE
ILMN_1730487	CALD1	FALSE	FALSE
ILMN_1681890	DYNLT3	FALSE	FALSE
ILMN_2074773	KCNK6	FALSE	FALSE
ILMN_1760950	SLC23A1	FALSE	FALSE
ILMN_1764628	LYPLA2	FALSE	FALSE
ILMN_1680774	LOC728392	FALSE	FALSE
ILMN_1789410	ZSCAN21	FALSE	FALSE
ILMN_1767470	SCPEP1	FALSE	FALSE
ILMN_2374244	DYRK2	FALSE	FALSE
ILMN_2394561	IRF2BP2	FALSE	FALSE
ILMN_1669931	TM9SF3	FALSE	FALSE
ILMN_1798485	ATP6V1E1	FALSE	FALSE
ILMN_3247802	PRRC2B	FALSE	FALSE
ILMN_1685602	TMEM41A	FALSE	FALSE
ILMN_1713380	EIF2B2	FALSE	FALSE
ILMN_1849494	EFR3B	FALSE	FALSE
ILMN_1670609	ATOX1	FALSE	FALSE
ILMN_1751886	REC8	FALSE	TRUE
ILMN_1815656	SERINC3	FALSE	TRUE
ILMN_2406043	NA	FALSE	FALSE
ILMN_1704091	DGAT1	FALSE	FALSE
ILMN_1778876	FAM179B	FALSE	FALSE
ILMN_1667306	RANBP10	FALSE	FALSE
ILMN_1726466	HDHD3	FALSE	FALSE
ILMN_1658759	PEX19	FALSE	FALSE
ILMN_1779401	CHP1	FALSE	FALSE
ILMN_1679979	PLK3	FALSE	FALSE
ILMN_1716547	NAGK	FALSE	FALSE
ILMN_2374770	TAX1BP1	FALSE	FALSE
ILMN_1804306	LYPLA2	FALSE	FALSE

ILMN_1786021	PRKAB2	FALSE	FALSE
ILMN_1696757	TTC14	FALSE	FALSE
ILMN_1702065	MFSD5	FALSE	FALSE
ILMN_1697561	FBXL16	FALSE	FALSE
ILMN_1656482	OSBPL2	TRUE	FALSE
ILMN_2246510	TSC1	TRUE	FALSE
ILMN_1754279	FBXW7	FALSE	FALSE
ILMN_1665964	GAB2	FALSE	FALSE
ILMN_2388484	MAP2	FALSE	FALSE
ILMN_1703142	Mar-02	FALSE	TRUE
ILMN_1761941	FAM198B	FALSE	TRUE
ILMN_1808220	LCE3E	FALSE	FALSE
ILMN_2386982	PRKCZ	FALSE	FALSE
ILMN_1862521	DENND1B	FALSE	FALSE
ILMN_1724718	NCK2	FALSE	FALSE
ILMN_1653404	NKIRAS2	FALSE	FALSE
ILMN_1688205	KLK10	FALSE	FALSE
ILMN_1857081	NA	FALSE	FALSE
ILMN_1680403	SSR4	TRUE	FALSE
ILMN_3262345	MROH1	FALSE	FALSE
ILMN_1793290	WDR60	FALSE	FALSE
ILMN_1810055	ITFG3	FALSE	FALSE
ILMN_2159453	STXBP2	FALSE	TRUE
ILMN_2256359	ZNF823	FALSE	FALSE
ILMN_2098643	FADS3	FALSE	FALSE
ILMN_1804988	MOAP1	TRUE	FALSE
ILMN_2174081	ZNF133	FALSE	TRUE
ILMN_1800543	SCFD1	FALSE	FALSE
ILMN_1662524	CXCR1	FALSE	FALSE
ILMN_1658247	OAS1	FALSE	FALSE
ILMN_1791508	MMP28	TRUE	FALSE
ILMN_1686735	LYPLA2	FALSE	FALSE
ILMN_2341595	KITLG	FALSE	FALSE
ILMN_1748915	S100A12	FALSE	FALSE
ILMN_1681016	SPNS1	FALSE	FALSE
ILMN_1739618	ZNF408	FALSE	FALSE
ILMN_1779374	AMMECR1	FALSE	FALSE
ILMN_1653266	DNAJB14	FALSE	FALSE
ILMN_1797342	FNBP1	FALSE	FALSE
ILMN_1719316	TMED3	FALSE	FALSE
ILMN_3242004	NA	TRUE	FALSE
ILMN_1911605	FRS2	FALSE	FALSE
ILMN_2103761	TLE4	FALSE	FALSE
ILMN_1713301	DGCR2	FALSE	FALSE
ILMN_2383107	VPS41	FALSE	FALSE
ILMN_1728498	PCBP4	FALSE	FALSE

ILMN_2087303	XKR8	FALSE	FALSE
ILMN_1773313	NA	FALSE	FALSE
ILMN_1755721	ANXA9	FALSE	FALSE
ILMN_1794230	SCAND1	FALSE	FALSE
ILMN_2366634	PKM	FALSE	FALSE
ILMN_1772124	ATRN	TRUE	TRUE
ILMN_2156953	ZFAND6	FALSE	TRUE
ILMN_1677098	YPEL2	TRUE	FALSE
ILMN_1802251	PTTG1IP	TRUE	FALSE
ILMN_1810591	PUS10	FALSE	FALSE
ILMN_2397721	GLB1	FALSE	FALSE
ILMN_2325337	APOL2	FALSE	FALSE
ILMN_1756715	RUNDC3A	FALSE	FALSE
ILMN_1714587	NA	FALSE	FALSE
ILMN_1783771	UBE2Z	FALSE	FALSE
ILMN_1806312	TMEM230	FALSE	FALSE
ILMN_1703246	SBF1	FALSE	FALSE
ILMN_2411731	HPS5	FALSE	FALSE
ILMN_2313782	ATG4A	FALSE	FALSE
ILMN_3270473	C6orf132	FALSE	FALSE
ILMN_2149566	VPS25	FALSE	FALSE
ILMN_1654516	TMEM120A	FALSE	FALSE
ILMN_1670130	ARID3A	FALSE	FALSE
ILMN_1800975	PSME3	FALSE	FALSE
ILMN_1701869	FBXO22	FALSE	FALSE
ILMN_3247023	CASC15	FALSE	FALSE
ILMN_1785988	CSNK1A1	FALSE	FALSE
ILMN_1721575	VPS18	FALSE	FALSE
ILMN_1735415	NUDT16L1	FALSE	TRUE
ILMN_1753608	TMEM131	FALSE	FALSE
ILMN_1778203	CLN5	FALSE	FALSE
ILMN_1813386	CORO6	FALSE	FALSE
ILMN_3307827	DNAJC4	FALSE	FALSE
ILMN_3203444	CERCAM	FALSE	FALSE
ILMN_2337955	ZMAT5	FALSE	FALSE
ILMN_1789186	OBFC1	FALSE	FALSE
ILMN_1716195	HIST1H2BG	FALSE	FALSE
ILMN_1652918	RHOF	FALSE	FALSE
ILMN_1745697	MGRN1	FALSE	FALSE
ILMN_1763828	MTF1	FALSE	FALSE
ILMN_2263466	ACADVL	FALSE	FALSE
ILMN_2352097	GPR56	FALSE	FALSE
ILMN_2354547	TUSC3	FALSE	FALSE
ILMN_1815308	SDC1	FALSE	FALSE
ILMN_1776993	COG2	FALSE	FALSE
ILMN_2161286	STRIP2	FALSE	FALSE

ILMN_2406557	ALDH4A1	FALSE	FALSE
ILMN_2140700	CRIPAK	TRUE	FALSE
ILMN_1733869	OGDH	FALSE	FALSE
ILMN_1742731	SLC35A2	FALSE	TRUE
ILMN_1734290	MAPRE3	TRUE	FALSE
ILMN_1753426	KIAA0556	FALSE	FALSE
ILMN_1774659	NA	FALSE	FALSE
ILMN_3268165	RNF7	FALSE	FALSE
ILMN_1680104	SLC35C1	FALSE	FALSE
ILMN_1867439	NA	FALSE	FALSE
ILMN_1836776	MPRIP	FALSE	FALSE
ILMN_2396571	GPR108	FALSE	FALSE
ILMN_1677843	RAB24	TRUE	FALSE
ILMN_1720865	OSBPL7	FALSE	FALSE
ILMN_1739161	PPAP2A	FALSE	FALSE
ILMN_1763228	MEF2D	FALSE	FALSE
ILMN_1660000	NA	FALSE	TRUE
ILMN_3210946	SELT	FALSE	FALSE
ILMN_1719165	BRMS1	FALSE	FALSE
ILMN_1812327	RNF19A	FALSE	TRUE
ILMN_1815306	AP2A1	TRUE	TRUE
ILMN_1664154	NA	FALSE	FALSE
ILMN_2089175	SYAP1	FALSE	FALSE
ILMN_1712806	AP1S1	FALSE	FALSE
ILMN_1891857	RAB2A	FALSE	FALSE
ILMN_1757604	TPM2	FALSE	FALSE
ILMN_1780302	DYNC1H1	FALSE	FALSE
ILMN_1691499	TJP1	FALSE	FALSE
ILMN_1807767	GSE1	FALSE	FALSE
ILMN_1673950	NA	FALSE	FALSE
ILMN_1786972	SARS	FALSE	FALSE
ILMN_1795905	ZBTB4	FALSE	FALSE
ILMN_1681777	SHROOM2	FALSE	FALSE
ILMN_1813517	DISP2	FALSE	FALSE
ILMN_1692785	KLHL21	FALSE	FALSE
ILMN_2197519	ZNF627	FALSE	FALSE
ILMN_1678268	VPS8	TRUE	FALSE
ILMN_1789436	DENND1B	FALSE	FALSE
ILMN_2155480	NA	FALSE	FALSE
ILMN_1785528	NA	FALSE	FALSE
ILMN_1676600	SEC24C	TRUE	FALSE
ILMN_1682919	PAFAH2	FALSE	FALSE
ILMN_2052163	YIPF1	FALSE	TRUE
ILMN_1750497	HCAR2	FALSE	FALSE
ILMN_2317580	SHANK3	FALSE	FALSE
ILMN_1805345	MOSPD3	FALSE	FALSE

ILMN_1726512	ZSCAN2	TRUE	FALSE
ILMN_2325338	APOL2	FALSE	FALSE
ILMN_3241136	NA	FALSE	FALSE
ILMN_1676665	NA	FALSE	FALSE
ILMN_1739558	CRELD1	FALSE	FALSE
ILMN_1803510	NA	FALSE	FALSE
ILMN_2367469	CARS	FALSE	FALSE
ILMN_1741491	ZNHIT1	FALSE	FALSE
ILMN_1806408	ACADVL	FALSE	FALSE
ILMN_1733757	TMEM179B	TRUE	FALSE
ILMN_2347949	G6PD	FALSE	FALSE
ILMN_1750429	MKNK1	FALSE	FALSE
ILMN_2144573	CTBS	FALSE	TRUE
ILMN_1694491	CCNG1	FALSE	FALSE
ILMN_1781983	AP1B1	FALSE	FALSE
ILMN_1666192	DCTN5	FALSE	FALSE
ILMN_2374383	TSPAN17	FALSE	FALSE
ILMN_1730639	SLC22A15	FALSE	FALSE
ILMN_1811489	OXSR1	FALSE	FALSE
ILMN_1852022	PLIN4	FALSE	FALSE
ILMN_2353161	MSLN	FALSE	FALSE
ILMN_1695899	RPS3A	FALSE	FALSE
ILMN_2169761	CPNE8	FALSE	TRUE
ILMN_3240524	MFSD6	FALSE	FALSE
ILMN_2195236	PGRMC2	FALSE	FALSE
ILMN_1668228	RPL18	FALSE	FALSE
ILMN_1669848	FAM25A	FALSE	FALSE
ILMN_2157099	CCNA1	FALSE	FALSE
ILMN_2256953	CASZ1	FALSE	FALSE
ILMN_2359456	ERGIC3	FALSE	TRUE
ILMN_2241124	GPR110	FALSE	TRUE
ILMN_1787212	CDKN1A	FALSE	FALSE
ILMN_1669557	AIM1L	FALSE	FALSE
ILMN_1798123	ELOVL1	FALSE	FALSE
ILMN_1714586	VGLL3	FALSE	FALSE
ILMN_1812474	TFG	FALSE	FALSE
ILMN_1652445	RAC1	FALSE	FALSE
ILMN_1753890	IFT20	FALSE	FALSE
ILMN_1664233	TMC6	FALSE	FALSE
ILMN_3272378	EZR	FALSE	FALSE
ILMN_1757388	OCEL1	FALSE	FALSE
ILMN_1687351	ANKRA2	FALSE	TRUE
ILMN_1764769	VWA5A	FALSE	FALSE
ILMN_1666076	ARHGEF4	FALSE	FALSE
ILMN_1812403	BCAP31	TRUE	FALSE
ILMN_1652631	GLIPR2	FALSE	FALSE

ILMN_1659240	MTMR14	FALSE	FALSE
ILMN_1705261	CAPN1	FALSE	FALSE
ILMN_1725130	FAM50A	FALSE	FALSE
ILMN_2384544	ADAM15	FALSE	FALSE
ILMN_1761797	CSTB	FALSE	FALSE
ILMN_1800354	CST3	FALSE	FALSE
ILMN_1752837	ARL8B	FALSE	FALSE
ILMN_3231638	FAM160B1	TRUE	FALSE
ILMN_1814726	SCARB2	FALSE	FALSE
ILMN_1679401	TRPM4	FALSE	TRUE
ILMN_1794122	ZNF79	TRUE	FALSE
ILMN_1674050	COL8A2	FALSE	FALSE
ILMN_1754179	AP1G2	TRUE	FALSE
ILMN_2380243	SMAGP	FALSE	FALSE
ILMN_3233930	SYNGR2	FALSE	FALSE
ILMN_2113490	NTN4	FALSE	FALSE
ILMN_2356654	LGALS8	FALSE	FALSE
ILMN_1735155	GLB1	FALSE	FALSE
ILMN_1653771	WDR63	FALSE	FALSE
ILMN_3307791	FAM69A	FALSE	FALSE
ILMN_1654392	KHNYN	FALSE	FALSE
ILMN_1837167	NA	FALSE	FALSE
ILMN_2281810	PSG4	FALSE	FALSE
ILMN_1808508	KITLG	FALSE	FALSE
ILMN_1727080	MYO6	FALSE	FALSE
ILMN_1809813	PGF	FALSE	FALSE
ILMN_1752932	MPZL2	FALSE	FALSE
ILMN_1753101	VTCN1	FALSE	FALSE
ILMN_1757639	SPTSSB	FALSE	FALSE
ILMN_3216365	NA	FALSE	FALSE
ILMN_1749662	GPX1	FALSE	FALSE
ILMN_1659415	LAMTOR3	FALSE	FALSE
ILMN_1678957	WDR55	FALSE	FALSE
ILMN_1789136	NA	FALSE	FALSE
ILMN_1777096	NA	FALSE	FALSE
ILMN_1807283	NDST1	FALSE	FALSE
ILMN_1732060	ARHGAP1	FALSE	FALSE
ILMN_1702168	HSD17B12	FALSE	FALSE
ILMN_1784333	SECISBP2L	FALSE	FALSE
ILMN_1789005	ATP6V0C	FALSE	FALSE
ILMN_1750409	RAB9A	FALSE	FALSE
ILMN_3251501	SERF2	FALSE	FALSE
ILMN_1764571	ARHGAP23	FALSE	TRUE
ILMN_2366445	KRT80	FALSE	FALSE
ILMN_1731181	TEX2	FALSE	FALSE
ILMN_1792837	CIAO1	FALSE	FALSE

ILMN_1718063	LIPA	FALSE	FALSE
ILMN_1655637	UPK1A	FALSE	FALSE
ILMN_2071937	ATP6V0E1	FALSE	FALSE
ILMN_2203588	MYL5	TRUE	FALSE
ILMN_2208777	NHLH2	FALSE	TRUE
ILMN_1758497	TTYH1	FALSE	FALSE
ILMN_1715635	ATP6V0E1	FALSE	FALSE
ILMN_1660079	RNF44	FALSE	FALSE
ILMN_1801516	GPC1	FALSE	FALSE
ILMN_1803889	CCDC64B	FALSE	FALSE
ILMN_2286400	CAPS	FALSE	FALSE
ILMN_2091846	FTH1	FALSE	FALSE
ILMN_1711715	NA	FALSE	FALSE
ILMN_1749915	RSRP1	FALSE	FALSE
ILMN_1728426	INPPL1	FALSE	FALSE
ILMN_1784554	NA	FALSE	FALSE
ILMN_1735180	NCSTN	FALSE	FALSE
ILMN_1738268	IP6K3	FALSE	FALSE
ILMN_1773901	STX12	FALSE	FALSE
ILMN_3256325	CYB561D1	TRUE	TRUE
ILMN_1803564	YIPF1	FALSE	TRUE
ILMN_1720965	TULP4	TRUE	FALSE
ILMN_1772706	NA	TRUE	FALSE
ILMN_3307877	C21orf58	FALSE	FALSE
ILMN_2285802	SEC14L1	FALSE	FALSE
ILMN_1790014	METRNL	FALSE	FALSE
ILMN_1662839	PLEKHA1	FALSE	FALSE
ILMN_1807455	DHRS7	FALSE	FALSE
ILMN_2126344	SEC16A	FALSE	FALSE
ILMN_1662973	CD82	FALSE	FALSE
ILMN_1701237	SH2D1B	FALSE	FALSE
ILMN_1659610	TJP3	FALSE	FALSE
ILMN_2162989	TMEM189	FALSE	FALSE
ILMN_1775016	MPZL2	FALSE	FALSE
ILMN_1658911	AP3S1	FALSE	FALSE
ILMN_1681542	HIST1H4E	FALSE	FALSE
ILMN_1653358	FUT3	FALSE	TRUE
ILMN_2410986	STAT3	FALSE	FALSE
ILMN_1677607	SC5D	FALSE	FALSE
ILMN_1756849	HIST1H2AE	FALSE	FALSE
ILMN_1793522	PRKAB1	FALSE	FALSE
ILMN_1664012	CANT1	FALSE	FALSE
ILMN_1657864	TMUB2	FALSE	FALSE
ILMN_1798177	CHURC1	TRUE	FALSE
ILMN_2339234	TRIM16	FALSE	FALSE
ILMN_2105253	PTGR2	FALSE	FALSE

ILMN_1694084	CYTH1	FALSE	FALSE
ILMN_1666004	WASL	FALSE	FALSE
ILMN_2312228	FUT6	FALSE	FALSE
ILMN_1741613	SERINC1	FALSE	FALSE
ILMN_1696065	SDF4	FALSE	FALSE
ILMN_1870041	NA	FALSE	FALSE
ILMN_1775692	EIF4G3	FALSE	FALSE
ILMN_3303295	NA	FALSE	FALSE
ILMN_3297945	UBA1	FALSE	FALSE
ILMN_1697409	TNFRSF14	FALSE	FALSE
ILMN_3307659	SFT2D2	FALSE	FALSE
ILMN_2161556	C12orf49	FALSE	TRUE
ILMN_1774083	TRIAP1	FALSE	FALSE
ILMN_1710427	VPS37B	FALSE	FALSE
ILMN_3274790	LOC283693	FALSE	FALSE
ILMN_2352303	RASSF2	FALSE	FALSE
ILMN_2398432	BRMS1	FALSE	FALSE
ILMN_1693310	ITFG1	FALSE	FALSE
ILMN_1691048	SLC22A18AS	FALSE	FALSE
ILMN_1699254	PLEKHH1	FALSE	FALSE
ILMN_1860638	ST3GAL1	FALSE	FALSE
ILMN_1741356	PRICKLE1	FALSE	FALSE
ILMN_1721704	FNTA	FALSE	TRUE
ILMN_1790533	PHACTR2	FALSE	FALSE
ILMN_3247223	TPBG	FALSE	FALSE
ILMN_2299795	CPM	FALSE	FALSE
ILMN_1788931	DOCK8	TRUE	FALSE
ILMN_2157421	STUB1	FALSE	FALSE
ILMN_2071641	KCNK1	FALSE	FALSE
ILMN_1692168	UBE2Z	FALSE	FALSE
ILMN_1679782	BLOC1S2	TRUE	FALSE
ILMN_1746883	SAT2	FALSE	FALSE
ILMN_3228294	RPL28	FALSE	FALSE
ILMN_1769390	ABHD15	FALSE	FALSE
ILMN_3276861	RPS2	FALSE	FALSE
ILMN_1795300	ARHGEF37	FALSE	FALSE
ILMN_1772768	PSG7	FALSE	FALSE
ILMN_3251545	CHMP5	FALSE	FALSE
ILMN_1660554	VWA1	FALSE	FALSE
ILMN_1651950	TPST1	FALSE	FALSE
ILMN_1682894	NA	FALSE	FALSE
ILMN_1720838	DECR1	TRUE	TRUE
ILMN_1687266	NA	FALSE	FALSE
ILMN_1797604	CAP1	FALSE	FALSE
ILMN_1786722	ZNF385A	FALSE	FALSE
ILMN_1684210	NIPAL3	FALSE	FALSE

ILMN_1787326	SNORA65	FALSE	FALSE
ILMN_2180352	DIP2B	FALSE	FALSE
ILMN_1764177	JARID2	FALSE	FALSE
ILMN_1759341	MAN2B1	FALSE	FALSE
ILMN_1719064	KCTD10	FALSE	FALSE
ILMN_1697652	PLEKHB2	FALSE	FALSE
ILMN_2108735	EEF1A2	FALSE	TRUE
ILMN_1711073	NA	FALSE	FALSE
ILMN_2159152	NA	FALSE	FALSE
ILMN_1771264	ELL3	FALSE	FALSE
ILMN_1757631	DBNDD1	FALSE	FALSE
ILMN_1748675	TMPRSS11B	FALSE	FALSE
ILMN_1734543	PTPRE	FALSE	FALSE
ILMN_1657871	RSAD2	FALSE	FALSE
ILMN_1679920	RPS12	FALSE	FALSE
ILMN_1773741	GOLGA5	FALSE	FALSE
ILMN_2337263	PKIB	FALSE	FALSE
ILMN_1746517	KYNU	FALSE	FALSE
ILMN_1651229	IPO13	FALSE	FALSE
ILMN_1781580	BRI3	FALSE	FALSE
ILMN_1697420	TINF2	FALSE	FALSE
ILMN_1720829	ZFP36	FALSE	FALSE
ILMN_2356111	SLC41A3	FALSE	FALSE
ILMN_3292008	RPL13AP3	FALSE	FALSE
ILMN_2398274	PYCARD	FALSE	FALSE
ILMN_1716869	GPM6A	TRUE	FALSE
ILMN_1736154	LZTS3	FALSE	FALSE
ILMN_1651538	NUMBL	FALSE	FALSE
ILMN_1704431	NA	FALSE	FALSE
ILMN_1710482	APLP2	FALSE	FALSE
ILMN_1782863	FAM83B	FALSE	FALSE
ILMN_3240222	SGK223	FALSE	FALSE
ILMN_1862018	ATXN7L3	FALSE	FALSE
ILMN_2184064	ARRDC4	FALSE	FALSE
ILMN_1679358	ZDHHC5	FALSE	FALSE
ILMN_1744963	ERO1L	FALSE	FALSE
ILMN_1694486	TCIRG1	TRUE	TRUE
ILMN_2082314	TOM1	FALSE	FALSE
ILMN_1654060	MKNK2	FALSE	TRUE
ILMN_2097185	PUS3	TRUE	FALSE
ILMN_2061327	TAF13	FALSE	FALSE
ILMN_1656335	RIT1	FALSE	FALSE
ILMN_1752579	ATP6V0A1	FALSE	FALSE
ILMN_2403730	ATP6V1H	FALSE	FALSE
ILMN_1815673	DKK3	FALSE	FALSE
ILMN_1710209	MFSD6	FALSE	FALSE

ILMN_2315694	STRADA	FALSE	FALSE
ILMN_3283772	GAPDH	FALSE	FALSE
ILMN_1739876	RAB3GAP1	TRUE	FALSE
ILMN_1750674	SDSL	FALSE	FALSE
ILMN_1747281	EVI5L	FALSE	TRUE
ILMN_2161330	SPDEF	FALSE	FALSE
ILMN_1761912	MGAT1	FALSE	FALSE
ILMN_1746408	MIDN	FALSE	FALSE
ILMN_3241091	TMEM164	FALSE	FALSE
ILMN_1812570	SHC1	FALSE	FALSE
ILMN_1739335	OST4	FALSE	FALSE
ILMN_1743836	MXRA7	FALSE	FALSE
ILMN_1673604	YIPF3	FALSE	FALSE
ILMN_1777261	FAM3C	FALSE	FALSE
ILMN_1692219	RAB11FIP1	FALSE	FALSE
ILMN_3197732	RPL7A	FALSE	FALSE
ILMN_1873620	TSPAN14	FALSE	FALSE
ILMN_2094106	HSD17B12	FALSE	FALSE
ILMN_2081087	HSPA12A	FALSE	FALSE
ILMN_2125374	CMAS	FALSE	FALSE
ILMN_1721563	TMEM127	FALSE	FALSE
ILMN_3194248	NA	FALSE	FALSE
ILMN_1802628	PPAPDC2	FALSE	FALSE
ILMN_3243381	MLEC	FALSE	FALSE
ILMN_1722089	RNF217	TRUE	TRUE
ILMN_1663787	KLK3	FALSE	FALSE
ILMN_1803452	CRCT1	FALSE	FALSE
ILMN_1692731	TTYH3	FALSE	FALSE
ILMN_1731418	SP110	FALSE	FALSE
ILMN_1715476	NA	FALSE	TRUE
ILMN_1786976	RAB22A	TRUE	TRUE
ILMN_1711408	ANXA4	FALSE	FALSE
ILMN_1747935	GOLGB1	TRUE	FALSE
ILMN_1669390	PPP1R13L	FALSE	TRUE
ILMN_2299221	RABGGTA	FALSE	FALSE
ILMN_1700733	FAM214A	FALSE	TRUE
ILMN_1800412	BMP1	TRUE	FALSE
ILMN_1787541	SPSB2	TRUE	FALSE
ILMN_1750256	ALS2	FALSE	FALSE
ILMN_1802905	PIAS4	FALSE	FALSE
ILMN_1810025	SLC39A2	FALSE	FALSE
ILMN_1781285	DUSP1	TRUE	TRUE
ILMN_1807093	NA	FALSE	FALSE
ILMN_1768505	IL13RA1	FALSE	FALSE
ILMN_3244019	KANK1	FALSE	FALSE
ILMN_2089977	FKBP9P1	FALSE	FALSE

ILMN_3205656	GAPDH	FALSE	FALSE
ILMN_1757872	SGK223	FALSE	FALSE
ILMN_2331266	NUMB	FALSE	FALSE
ILMN_1707475	UBE2E2	FALSE	FALSE
ILMN_2126957	NOMO1	FALSE	FALSE
ILMN_1714158	PON2	FALSE	FALSE
ILMN_1751351	C6orf132	FALSE	FALSE
ILMN_2375360	COL4A5	FALSE	TRUE
ILMN_1769071	XKRX	FALSE	FALSE
ILMN_1732575	SEC14L1	FALSE	FALSE
ILMN_1731113	ZBTB43	FALSE	FALSE
ILMN_1814213	PQLC3	FALSE	FALSE
ILMN_3204312	ATP5G1	FALSE	FALSE
ILMN_1706571	SLC35D2	TRUE	FALSE
ILMN_1755909	GID8	FALSE	TRUE
ILMN_1686906	TP53INP2	TRUE	FALSE
ILMN_1738075	CMIP	FALSE	FALSE
ILMN_1839647	LINC00323	FALSE	FALSE
ILMN_1747589	HIST2H2AB	FALSE	FALSE
ILMN_1746588	TALDO1	FALSE	FALSE
ILMN_1690921	STAT2	FALSE	FALSE
ILMN_1770977	TMEM134	FALSE	TRUE
ILMN_1795937	EZR	FALSE	FALSE
ILMN_3243598	PDZK1P2	FALSE	FALSE
ILMN_1681695	DNASE1L1	FALSE	FALSE
ILMN_1655922	NA	FALSE	FALSE
ILMN_1701621	SCO2	FALSE	FALSE
ILMN_1758626	IDS	FALSE	FALSE
ILMN_1729225	SORCS2	TRUE	TRUE
ILMN_2260991	TSPO	FALSE	FALSE
ILMN_1752249	PIEZO1	FALSE	FALSE
ILMN_1791569	PLXNA1	TRUE	TRUE
ILMN_2363058	PAOX	FALSE	FALSE
ILMN_1787657	CLDN12	FALSE	FALSE
ILMN_1846771	NA	TRUE	FALSE
ILMN_1674151	NA	FALSE	FALSE
ILMN_2359907	CD68	FALSE	FALSE
ILMN_3245236	FBRS	FALSE	FALSE
ILMN_1717674	PEPD	TRUE	FALSE
ILMN_1713952	C1orf106	FALSE	FALSE
ILMN_1659836	RAET1G	FALSE	FALSE
ILMN_1714527	VAMP3	TRUE	FALSE
ILMN_2099783	ATP6V1F	FALSE	FALSE
ILMN_3263225	LOC100288911	FALSE	FALSE
ILMN_1673282	LAMP2	FALSE	FALSE
ILMN_1709549	PLEKHM1	FALSE	FALSE

ILMN_1778202	NA	FALSE	FALSE
ILMN_1773849	ATP6V0C	FALSE	FALSE
ILMN_1674759	LPCAT4	FALSE	FALSE
ILMN_3247452	OST4	FALSE	FALSE
ILMN_1665877	RNF149	FALSE	FALSE
ILMN_1781386	WIPI1	TRUE	FALSE
ILMN_2184184	ANXA1	FALSE	FALSE
ILMN_1713688	DHX32	FALSE	FALSE
ILMN_1695946	TRNP1	TRUE	FALSE
ILMN_1724863	TICAM1	FALSE	FALSE
ILMN_1746928	TMPRSS11D	FALSE	FALSE
ILMN_3249244	TMEM106A	FALSE	FALSE
ILMN_2340065	UBL5	FALSE	FALSE
ILMN_1793770	DNAJB6	FALSE	FALSE
ILMN_2375992	SPINT1	FALSE	FALSE
ILMN_1658830	WBP1L	TRUE	FALSE
ILMN_2121068	ADAM17	FALSE	FALSE
ILMN_1679797	ADARB1	FALSE	FALSE
ILMN_3273229	LOC100129781	FALSE	FALSE
ILMN_1716913	TRAPPC1	FALSE	FALSE
ILMN_1659749	DSP	FALSE	FALSE
ILMN_2096322	ADIPOR1	FALSE	FALSE
ILMN_1733655	NA	FALSE	FALSE
ILMN_2337058	PORCN	FALSE	FALSE
ILMN_1798679	ANO9	FALSE	FALSE
ILMN_1810962	PTPRK	FALSE	FALSE
ILMN_1806758	C9orf85	FALSE	FALSE
ILMN_2380967	DNASE1L1	FALSE	FALSE
ILMN_1859127	LOC100129781	FALSE	FALSE
ILMN_1673380	GNG12	FALSE	FALSE
ILMN_1903021	NA	TRUE	FALSE
ILMN_1751627	TRAPPC3	FALSE	FALSE
ILMN_1698752	NLRX1	FALSE	FALSE
ILMN_1664016	ARHGEF18	FALSE	FALSE
ILMN_1718699	RAP1B	FALSE	FALSE
ILMN_3244157	SNORD83B	FALSE	FALSE
ILMN_1722798	PLCD3	TRUE	FALSE
ILMN_1678729	SIL1	FALSE	FALSE
ILMN_1670096	NRBP1	FALSE	FALSE
ILMN_1775883	IVL	TRUE	FALSE
ILMN_1775549	PRSS27	FALSE	FALSE
ILMN_1659753	LAMP2	FALSE	FALSE
ILMN_2374865	ATF3	FALSE	TRUE
ILMN_1797522	DUSP3	TRUE	FALSE
ILMN_1662578	C1GALT1	FALSE	FALSE
ILMN_1752526	RNF144B	FALSE	FALSE

ILMN_1686750	MGEA5	FALSE	FALSE
ILMN_1730007	MPZL2	FALSE	FALSE
ILMN_2061950	RABGAP1	FALSE	FALSE
ILMN_1801043	GSN	FALSE	FALSE
ILMN_3251145	LDHA	FALSE	FALSE
ILMN_2406532	F11R	FALSE	FALSE
ILMN_1733288	C1RL	FALSE	FALSE
ILMN_1703123	CSRNP1	FALSE	TRUE
ILMN_1705922	BAIAP2	FALSE	FALSE
ILMN_1763447	PLXNB2	FALSE	FALSE
ILMN_1666924	PINK1	FALSE	FALSE
ILMN_1702247	CCNDBP1	FALSE	FALSE
ILMN_1734153	GDI1	TRUE	FALSE
ILMN_1777446	NA	FALSE	FALSE
ILMN_1736939	UGCG	FALSE	FALSE
ILMN_2316173	AP1S1	FALSE	FALSE
ILMN_1745079	TRIM2	FALSE	FALSE
ILMN_3213925	GPX1	FALSE	FALSE
ILMN_1717234	CAST	FALSE	FALSE
ILMN_2094166	CHMP5	FALSE	FALSE
ILMN_1707156	LRRFIP2	FALSE	FALSE
ILMN_1764230	GNPTG	FALSE	FALSE
ILMN_1678922	HERC4	TRUE	FALSE
ILMN_1812721	HIP1R	FALSE	FALSE
ILMN_1767111	ANO10	FALSE	TRUE
ILMN_2103547	GOLGA8B	FALSE	FALSE
ILMN_1711208	CELSR2	FALSE	FALSE
ILMN_2413779	SEZ6L2	FALSE	FALSE
ILMN_2398159	DKK3	FALSE	FALSE
ILMN_1762561	PLA2G10	FALSE	FALSE
ILMN_1693090	CROT	FALSE	FALSE
ILMN_2401978	STAT3	FALSE	FALSE
ILMN_1683658	FKBP1A	FALSE	FALSE
ILMN_1721316	TNFRSF10A	FALSE	FALSE
ILMN_1699022	ENDOD1	FALSE	FALSE
ILMN_2237428	SCD5	FALSE	FALSE
ILMN_1682165	NT5C2	TRUE	FALSE
ILMN_1815392	ACTRT1	FALSE	FALSE
ILMN_2046470	DAAM1	FALSE	FALSE
ILMN_1734184	PLBD2	FALSE	FALSE
ILMN_2117323	PIK3C2B	FALSE	FALSE
ILMN_1815578	ZNF223	FALSE	FALSE
ILMN_2371724	CEACAM1	FALSE	FALSE
ILMN_1702738	KLC3	FALSE	FALSE
ILMN_1684211	SEC14L2	FALSE	FALSE
ILMN_1815319	CMTM4	FALSE	FALSE

ILMN_1757072	FKBP1A	FALSE	FALSE
ILMN_1685934	UPK2	FALSE	FALSE
ILMN_2347807	EXOC1	FALSE	FALSE
ILMN_1809437	RHBDD2	FALSE	FALSE
ILMN_1720053	ZFAND3	FALSE	FALSE
ILMN_1695285	CAPN14	FALSE	FALSE
ILMN_1721833	IER5	FALSE	FALSE
ILMN_1706531	ABCC5	FALSE	FALSE
ILMN_1774547	MPRIP	FALSE	FALSE
ILMN_1721651	UBE2H	FALSE	FALSE
ILMN_1882590	NACC2	FALSE	FALSE
ILMN_1701361	LURAP1L	FALSE	FALSE
ILMN_1853167	NA	FALSE	FALSE
ILMN_1752927	FAM160B1	FALSE	FALSE
ILMN_3251550	PHLDA1	FALSE	FALSE
ILMN_1656902	HECTD3	FALSE	FALSE
ILMN_3225534	RNF216P1	FALSE	FALSE
ILMN_3300663	NA	FALSE	FALSE
ILMN_1674034	H2AFY	FALSE	TRUE
ILMN_2374352	DBNDD1	FALSE	FALSE
ILMN_1674038	CTSD	FALSE	FALSE
ILMN_2334242	CREB1	FALSE	FALSE
ILMN_1687035	ADAMTSL4	FALSE	FALSE
ILMN_1671928	PROS1	FALSE	FALSE
ILMN_1680579	ATP2B4	TRUE	FALSE
ILMN_2095610	NA	TRUE	FALSE
ILMN_1698554	AACS	FALSE	FALSE
ILMN_1660544	ARRDC4	FALSE	FALSE
ILMN_1738523	MYD88	FALSE	FALSE
ILMN_1673305	RHOC	FALSE	FALSE
ILMN_2342066	METRNL	FALSE	FALSE
ILMN_1671893	CHMP2A	FALSE	FALSE
ILMN_2359742	CTSB	FALSE	FALSE
ILMN_1776519	RAP1GAP	FALSE	FALSE
ILMN_1711994	TCIRG1	TRUE	FALSE
ILMN_1691410	BAMBI	FALSE	FALSE
ILMN_1752968	LAMB2	FALSE	FALSE
ILMN_2065783	EXOC2	FALSE	TRUE
ILMN_2149766	APPBP2	FALSE	FALSE
ILMN_1653708	CORO1B	FALSE	FALSE
ILMN_1732534	CHMP5	FALSE	FALSE
ILMN_1700001	TCTA	FALSE	FALSE
ILMN_1784364	STARD5	FALSE	FALSE
ILMN_2399896	SEC31A	FALSE	FALSE
ILMN_1653220	PITPNM1	FALSE	FALSE
ILMN_1719696	PLD1	FALSE	FALSE

ILMN_1733937	MMD	TRUE	FALSE
ILMN_1798372	ANXA2P3	FALSE	FALSE
ILMN_1699265	TNFRSF10B	FALSE	FALSE
ILMN_2061318	TAF13	FALSE	FALSE
ILMN_1772991	NA	FALSE	FALSE
ILMN_2091347	IDH1	FALSE	FALSE
ILMN_1740276	CLDN9	FALSE	FALSE
ILMN_3302499	IDS	FALSE	FALSE
ILMN_2388605	ACTR2	FALSE	FALSE
ILMN_1765132	LACTB	FALSE	FALSE
ILMN_1702127	SPRR2G	FALSE	TRUE
ILMN_1805853	PHLDB3	FALSE	FALSE
ILMN_1719753	VGLL1	FALSE	FALSE
ILMN_1735052	ULK1	TRUE	FALSE
ILMN_1703335	LACTB	FALSE	FALSE
ILMN_1678454	CASP4	TRUE	TRUE
ILMN_1741371	TMEM8A	FALSE	TRUE
ILMN_2387731	NDUFV3	FALSE	FALSE
ILMN_1795359	SPRR2A	FALSE	FALSE
ILMN_1782292	LAMP1	FALSE	FALSE
ILMN_2311761	AP3S1	FALSE	FALSE
ILMN_1674522	HIGD1A	FALSE	FALSE
ILMN_2367753	ATP2B4	TRUE	FALSE
ILMN_1653828	CHFR	FALSE	FALSE
ILMN_1737644	TMEM219	FALSE	FALSE
ILMN_1722834	RGS12	TRUE	FALSE
ILMN_1768469	TCN1	FALSE	FALSE
ILMN_1659564	SEC61A1	TRUE	FALSE
ILMN_1746561	BCL2L2	TRUE	FALSE
ILMN_1814789	UBAP2L	FALSE	FALSE
ILMN_1815937	TMEM184A	FALSE	FALSE
ILMN_2180239	DOPEY2	FALSE	FALSE
ILMN_1776115	GSDMA	FALSE	FALSE
ILMN_1682658	EPM2AIP1	FALSE	FALSE
ILMN_1799098	NA	TRUE	FALSE
ILMN_1724700	RIOK3	FALSE	FALSE
ILMN_1789830	CFLAR	FALSE	FALSE
ILMN_1721629	ZNF654	FALSE	FALSE
ILMN_1651438	ZFPM1	FALSE	FALSE
ILMN_1667418	TMEM114	FALSE	FALSE
ILMN_1712298	ANKRD46	FALSE	FALSE
ILMN_1810533	SLC6A15	FALSE	FALSE
ILMN_2387078	MPZL2	FALSE	FALSE
ILMN_1763144	NEU1	FALSE	FALSE
ILMN_2101651	UBE2G2	FALSE	FALSE
ILMN_1756595	SH3TC1	TRUE	FALSE

ILMN_1797005	PGLS	TRUE	FALSE
ILMN_1664750	TMBIM4	FALSE	FALSE
ILMN_1802799	AKIRIN1	FALSE	FALSE
ILMN_1769394	PLCD1	FALSE	FALSE
ILMN_1729987	SRC	TRUE	TRUE
ILMN_1750394	SLC39A6	FALSE	FALSE
ILMN_2078599	ACP5	FALSE	FALSE
ILMN_1745148	ZNFX1	FALSE	FALSE
ILMN_1724959	SEC31A	FALSE	FALSE
ILMN_1699925	TGM5	FALSE	FALSE
ILMN_1764508	PTK6	FALSE	FALSE
ILMN_2373062	RHBDF2	FALSE	FALSE
ILMN_1771120	TMEM45B	FALSE	FALSE
ILMN_1705116	SLC22A23	FALSE	FALSE
ILMN_1751956	MGST3	FALSE	FALSE
ILMN_1706051	PLD5	TRUE	FALSE
ILMN_2353642	ATP6V0B	FALSE	FALSE
ILMN_1803811	TRIB1	FALSE	TRUE
ILMN_1762294	ADAMTSL4	FALSE	FALSE
ILMN_1692133	ZNF226	FALSE	FALSE
ILMN_2223903	PPIC	FALSE	FALSE
ILMN_2152429	C12orf76	FALSE	FALSE
ILMN_1688322	ADIPOR1	FALSE	FALSE
ILMN_1680770	UBQLN2	FALSE	FALSE
ILMN_1668194	LMTK3	FALSE	FALSE
ILMN_1738921	ACAA1	FALSE	FALSE
ILMN_1793517	RASAL1	FALSE	FALSE
ILMN_1685699	PRSS3	FALSE	FALSE
ILMN_2382505	SLC22A18	TRUE	FALSE
ILMN_1696675	CES2	FALSE	FALSE
ILMN_1743078	NA	FALSE	FALSE
ILMN_1714082	CMAS	FALSE	FALSE
ILMN_3176146	C6orf132	FALSE	FALSE
ILMN_1787308	PIP4K2C	TRUE	FALSE
ILMN_2339863	VPS28	FALSE	FALSE
ILMN_1711699	HIP1R	FALSE	FALSE
ILMN_1808115	ATP7A	FALSE	FALSE
ILMN_1796639	UBQLN3	FALSE	FALSE
ILMN_1721022	SHC1	TRUE	TRUE
ILMN_1719938	FGF11	TRUE	FALSE
ILMN_2415179	CLSTN1	FALSE	FALSE
ILMN_2367233	ZNF654	FALSE	FALSE
ILMN_1733333	CALML3	FALSE	FALSE
ILMN_1694147	PUS3	FALSE	FALSE
ILMN_1691717	RHBDF2	FALSE	FALSE
ILMN_3237396	AAGAB	FALSE	FALSE

ILMN_2075643	ANKRD29	FALSE	FALSE
ILMN_1886769	NA	FALSE	FALSE
ILMN_1658425	DAG1	FALSE	TRUE
ILMN_3238452	FAM21C	TRUE	FALSE
ILMN_1667460	SULF2	FALSE	FALSE
ILMN_1788223	RSPH3	FALSE	FALSE
ILMN_1795826	ATP6V0D1	FALSE	FALSE
ILMN_2368773	FAM3C	FALSE	FALSE
ILMN_1671885	MLF2	FALSE	FALSE
ILMN_1781791	PRRG1	FALSE	TRUE
ILMN_1725079	TSPAN31	FALSE	TRUE
ILMN_1734288	DUSP18	FALSE	FALSE
ILMN_2244841	ALDH4A1	FALSE	FALSE
ILMN_1778242	CALM1	FALSE	TRUE
ILMN_1773427	KANK1	FALSE	FALSE
ILMN_1700310	NA	TRUE	FALSE
ILMN_1744471	ZNF654	FALSE	FALSE
ILMN_1761259	EXT2	FALSE	FALSE
ILMN_2143566	SLC39A6	FALSE	FALSE
ILMN_1671600	EPS8L1	FALSE	FALSE
ILMN_1738237	HS1BP3	FALSE	FALSE
ILMN_2170595	RRM2B	FALSE	FALSE
ILMN_1793537	MUC15	FALSE	FALSE
ILMN_1717165	IGBP1	FALSE	FALSE
ILMN_2396546	IGSF3	FALSE	FALSE
ILMN_1719518	ARF4	FALSE	FALSE
ILMN_1659106	PHLDA3	FALSE	FALSE
ILMN_2230016	HIGD1A	FALSE	FALSE
ILMN_2404085	CLIP1	FALSE	FALSE
ILMN_2214678	MXD1	TRUE	FALSE
ILMN_1693630	VPS9D1	TRUE	FALSE
ILMN_1750549	PI4K2A	FALSE	FALSE
ILMN_1694810	PANX2	FALSE	FALSE
ILMN_1740604	RAB11FIP5	FALSE	FALSE
ILMN_2362681	CES2	FALSE	FALSE
ILMN_1890614	FAM212B	FALSE	FALSE
ILMN_1719972	PLXNA3	FALSE	FALSE
ILMN_2121816	GPR137B	TRUE	FALSE
ILMN_1777325	STAT1	FALSE	FALSE
ILMN_2164242	UBE2F	FALSE	FALSE
ILMN_1657495	MLEC	FALSE	FALSE
ILMN_1717052	STARD10	FALSE	FALSE
ILMN_2158336	SH3GLB2	FALSE	FALSE
ILMN_1729288	C1QTNF6	FALSE	FALSE
ILMN_2153495	WNT7B	FALSE	FALSE
ILMN_2075927	STK40	FALSE	FALSE

ILMN_1699545	PCSK7	FALSE	FALSE
ILMN_1788540	PHLDB3	FALSE	FALSE
ILMN_1697694	ATP6AP1	FALSE	FALSE
ILMN_2151056	C10orf32	TRUE	FALSE
ILMN_2301624	MACF1	FALSE	FALSE
ILMN_1688480	CCND1	FALSE	FALSE
ILMN_2104106	XPR1	FALSE	FALSE
ILMN_1712786	AHCYL2	FALSE	FALSE
ILMN_2232166	CCDC90B	FALSE	TRUE
ILMN_1794017	SERTAD1	FALSE	FALSE
ILMN_1803772	POLD4	FALSE	FALSE
ILMN_1790471	CIDECP	FALSE	FALSE
ILMN_1759003	SNX12	FALSE	FALSE
ILMN_2196588	NA	FALSE	FALSE
ILMN_2191803	MYPN	FALSE	FALSE
ILMN_1802706	IDH3G	FALSE	FALSE
ILMN_1728049	S100A16	FALSE	FALSE
ILMN_1719661	MSRB1	FALSE	FALSE
ILMN_1663092	CITED2	FALSE	FALSE
ILMN_2366864	JUP	FALSE	FALSE
ILMN_1730660	LGALS3	TRUE	FALSE
ILMN_1760792	KLHL7	FALSE	FALSE
ILMN_1815012	EXOC7	TRUE	FALSE
ILMN_1753472	SUMF1	FALSE	FALSE
ILMN_1755937	ANXA2	FALSE	FALSE
ILMN_1675640	OAS1	FALSE	TRUE
ILMN_1782331	NA	FALSE	FALSE
ILMN_1800341	WDR66	FALSE	FALSE
ILMN_1698243	C1orf85	FALSE	FALSE
ILMN_2374159	HERPUD1	FALSE	FALSE
ILMN_1676611	PHPT1	FALSE	FALSE
ILMN_1735156	SLC4A11	FALSE	TRUE
ILMN_1795711	LCE2B	FALSE	TRUE
ILMN_1708016	FAM210B	FALSE	FALSE
ILMN_1719343	WDR26	FALSE	FALSE
ILMN_1811574	MAPK8IP3	TRUE	FALSE
ILMN_3235171	CCDC64B	FALSE	FALSE
ILMN_1726516	SCRIB	FALSE	FALSE
ILMN_1765578	TIPARP	FALSE	FALSE
ILMN_1789095	BMPR2	FALSE	FALSE
ILMN_1694530	DDA1	FALSE	FALSE
ILMN_2111932	SERINC2	FALSE	FALSE
ILMN_2233454	SPTLC3	FALSE	FALSE
ILMN_1742534	COL4A5	FALSE	FALSE
ILMN_1749044	PVRL4	FALSE	FALSE
ILMN_1749834	SMIM1	FALSE	FALSE

ILMN_1791826	RAB25	FALSE	FALSE
ILMN_1725241	GSTK1	FALSE	FALSE
ILMN_1793859	ALDH2	FALSE	FALSE
ILMN_1671565	RNASET2	FALSE	FALSE
ILMN_1737988	PRNP	FALSE	FALSE
ILMN_1779828	EDEM1	FALSE	FALSE
ILMN_1804117	FAM89B	FALSE	FALSE
ILMN_3284114	CAP1	FALSE	FALSE
ILMN_1775522	MAGED1	TRUE	TRUE
ILMN_1769550	SLFN5	FALSE	FALSE
ILMN_1685312	NA	FALSE	FALSE
ILMN_1722025	CPEB4	FALSE	FALSE
ILMN_1749109	PSAP	TRUE	FALSE
ILMN_1803810	RRBP1	FALSE	FALSE
ILMN_2407389	GPNMB	FALSE	TRUE
ILMN_1741970	JUP	FALSE	FALSE
ILMN_2382717	OPN3	FALSE	FALSE
ILMN_1729453	TSPAN9	FALSE	FALSE
ILMN_1699071	MAP3K7CL	FALSE	FALSE
ILMN_1737561	N4BP2L2	TRUE	FALSE
ILMN_3237385	NRBF2	TRUE	FALSE
ILMN_2380688	B4GALT4	FALSE	FALSE
ILMN_1680223	PNPLA8	FALSE	FALSE
ILMN_1677440	ATP6AP2	TRUE	FALSE
ILMN_1804901	IL36RN	FALSE	FALSE
ILMN_2361400	ABCA12	FALSE	FALSE
ILMN_2393712	CTTN	FALSE	FALSE
ILMN_1723035	OLR1	FALSE	FALSE
ILMN_2151441	FAM103A1	FALSE	FALSE
ILMN_1697597	EFCAB14	FALSE	FALSE
ILMN_1805410	C15orf48	FALSE	FALSE
ILMN_1741392	SLC25A20	FALSE	FALSE
ILMN_2169025	JOSD2	FALSE	FALSE
ILMN_1765258	HLA-E	TRUE	FALSE
ILMN_1745256	NA	FALSE	FALSE
ILMN_1743396	ACOX3	FALSE	FALSE
ILMN_1803211	FBXO2	FALSE	FALSE
ILMN_1758852	ENTPD7	FALSE	FALSE
ILMN_1708223	PAK6	FALSE	FALSE
ILMN_1679311	STK40	FALSE	FALSE
ILMN_2330787	FRMD6	FALSE	FALSE
ILMN_1664802	WSB1	FALSE	FALSE
ILMN_1796177	GIPC1	FALSE	FALSE
ILMN_1731353	CHPF	FALSE	FALSE
ILMN_1759676	HOXC13	FALSE	FALSE
ILMN_2097793	ZBTB4	FALSE	FALSE

ILMN_1766221	B4GALT1	FALSE	FALSE
ILMN_1682938	ARF3	FALSE	FALSE
ILMN_1773389	PLTP	FALSE	FALSE
ILMN_2343278	PPAP2A	FALSE	FALSE
ILMN_2390974	DNAJB2	TRUE	FALSE
ILMN_1783287	S100A14	FALSE	FALSE
ILMN_1661799	HIGD1A	FALSE	FALSE
ILMN_2342033	F11R	FALSE	FALSE
ILMN_3227263	SLC22A23	FALSE	TRUE
ILMN_1778087	NA	TRUE	FALSE
ILMN_1714861	CD68	FALSE	FALSE
ILMN_1800512	HMOX1	FALSE	FALSE
ILMN_1807972	MICAL1	FALSE	FALSE
ILMN_1814966	PIAS3	FALSE	TRUE
ILMN_2113535	PCYOX1	FALSE	FALSE
ILMN_1764361	DUSP16	FALSE	FALSE
ILMN_3241870	FRMD8	FALSE	FALSE
ILMN_1698666	CST6	FALSE	FALSE
ILMN_1739450	NFE2L1	TRUE	FALSE
ILMN_2376822	NA	FALSE	FALSE
ILMN_2352131	ERBB2	FALSE	FALSE
ILMN_3247578	FAT1	FALSE	FALSE
ILMN_1779677	ZCCHC6	TRUE	FALSE
ILMN_1800739	SPINT2	FALSE	FALSE
ILMN_1715715	CEBPA	FALSE	FALSE
ILMN_2355559	PSAP	TRUE	FALSE
ILMN_1760556	NA	FALSE	FALSE
ILMN_2277676	ERCC1	FALSE	FALSE
ILMN_1732066	CKMT1A	FALSE	FALSE
ILMN_1778360	PYGB	TRUE	TRUE
ILMN_1716249	OR1K1	FALSE	FALSE
ILMN_1656194	TSPAN10	FALSE	TRUE
ILMN_3251605	KLHL28	FALSE	FALSE
ILMN_1794677	TMC6	FALSE	FALSE
ILMN_2188722	NA	FALSE	FALSE
ILMN_1697548	LPHN2	FALSE	FALSE
ILMN_1733248	NRBP2	FALSE	FALSE
ILMN_1744534	LYRM5	FALSE	FALSE
ILMN_1716988	OPN3	FALSE	TRUE
ILMN_1670145	DFNA5	FALSE	FALSE
ILMN_2071809	MGP	FALSE	FALSE
ILMN_1796094	CD36	FALSE	FALSE
ILMN_1684653	GPR115	FALSE	FALSE
ILMN_1720303	OSTM1	FALSE	FALSE
ILMN_1754795	FAT1	FALSE	FALSE
ILMN_3307874	AIM1L	FALSE	FALSE

ILMN_1810420	DYSF	FALSE	FALSE
ILMN_1727479	TPRG1L	TRUE	FALSE
ILMN_1803838	CNFN	FALSE	FALSE
ILMN_2390586	SP100	FALSE	TRUE
ILMN_1656938	SLC37A2	FALSE	FALSE
ILMN_1795963	OSGIN1	FALSE	FALSE
ILMN_1712577	FAM174A	FALSE	FALSE
ILMN_1651429	SELM	FALSE	FALSE
ILMN_1810560	NUPR1	FALSE	TRUE
ILMN_1770454	AGRN	TRUE	FALSE
ILMN_1679725	PCYOX1	FALSE	FALSE
ILMN_1673769	KCNG1	FALSE	FALSE
ILMN_1765109	TNFRSF25	TRUE	FALSE
ILMN_1677200	CYFIP2	FALSE	FALSE
ILMN_2375484	CPEB2	FALSE	FALSE
ILMN_1769810	ARL6IP5	FALSE	FALSE
ILMN_1735365	GJB5	FALSE	FALSE
ILMN_2394250	PLEKHA1	FALSE	FALSE
ILMN_2322806	CAST	FALSE	FALSE
ILMN_1746917	NA	FALSE	FALSE
ILMN_1753468	CD63	FALSE	FALSE
ILMN_3245194	NA	FALSE	FALSE
ILMN_2133675	SGSH	FALSE	FALSE
ILMN_1749011	NECAP2	FALSE	FALSE
ILMN_1810289	MYOF	FALSE	FALSE
ILMN_1898723	NA	FALSE	FALSE
ILMN_1705144	ULK1	FALSE	FALSE
ILMN_1813704	CEMIP	FALSE	FALSE
ILMN_1788250	LDOC1	FALSE	FALSE
ILMN_1721127	HIST1H3D	FALSE	FALSE
ILMN_2401779	FAM102A	FALSE	FALSE
ILMN_1777915	STX6	FALSE	FALSE
ILMN_2404135	RIOK3	FALSE	FALSE
ILMN_1810852	LAMC1	FALSE	FALSE
ILMN_1739594	ACOT11	FALSE	FALSE
ILMN_1718960	SERPINB8	FALSE	FALSE
ILMN_1653283	APP	TRUE	FALSE
ILMN_1714820	ITGB1	FALSE	FALSE
ILMN_1900520	NA	FALSE	FALSE
ILMN_1768510	MAN2B2	FALSE	FALSE
ILMN_1808501	SH3KBP1	FALSE	FALSE
ILMN_1669820	NA	FALSE	FALSE
ILMN_1794914	UBTD1	TRUE	FALSE
ILMN_1659027	SLC2A1	FALSE	FALSE
ILMN_2381697	P4HA2	FALSE	FALSE
ILMN_1717326	SLC29A3	TRUE	FALSE

ILMN_2349138	CDC42SE1	FALSE	FALSE
ILMN_1708728	H2AFJ	FALSE	FALSE
ILMN_1803824	ZDHHC9	FALSE	FALSE
ILMN_1702835	SH3BGRL	FALSE	TRUE
ILMN_1804329	TUSC2	FALSE	TRUE
ILMN_2064150	PRRG2	FALSE	FALSE
ILMN_1675612	BLCAP	FALSE	TRUE
ILMN_1660021	PLIN3	FALSE	FALSE
ILMN_1704753	EPAS1	FALSE	FALSE
ILMN_1746396	CTNNB1	FALSE	FALSE
ILMN_1724533	LY96	FALSE	FALSE
ILMN_1757350	CTNNB1	FALSE	FALSE
ILMN_1746618	PAQR7	FALSE	FALSE
ILMN_1725912	KLK12	FALSE	FALSE
ILMN_3309349	SNHG8	FALSE	FALSE
ILMN_1778625	CD44	FALSE	FALSE
ILMN_1778575	WNT7A	FALSE	FALSE
ILMN_1802053	ZNF91	FALSE	FALSE
ILMN_1677396	NDFIP2	FALSE	FALSE
ILMN_1701173	KCNK6	FALSE	FALSE
ILMN_1757644	UBE2H	FALSE	FALSE
ILMN_1801610	METRNL	FALSE	FALSE
ILMN_1767816	APH1B	FALSE	FALSE
ILMN_1730611	RTN4	FALSE	FALSE
ILMN_1763520	SULT2B1	FALSE	FALSE
ILMN_1674367	SPRR2F	FALSE	FALSE
ILMN_1787251	DAAM1	FALSE	FALSE
ILMN_1810785	RNF11	FALSE	FALSE
ILMN_1655429	TNFAIP1	FALSE	FALSE
ILMN_2383611	PTPRE	FALSE	FALSE
ILMN_1711566	TIMP1	FALSE	FALSE
ILMN_1701731	AKR1B1	FALSE	TRUE
ILMN_1802603	RFNG	FALSE	FALSE
ILMN_1790807	XPC	FALSE	FALSE
ILMN_2299612	TMEM150A	FALSE	FALSE
ILMN_1665982	AKTIP	FALSE	FALSE
ILMN_1703433	NA	FALSE	FALSE
ILMN_1780711	NA	FALSE	FALSE
ILMN_1750785	SYTL1	FALSE	FALSE
ILMN_1660624	LIMK2	FALSE	FALSE
ILMN_1802888	ZNF185	FALSE	FALSE
ILMN_2190084	VAMP8	FALSE	FALSE
ILMN_1708341	PDZK1	FALSE	FALSE
ILMN_1712748	GSKIP	FALSE	FALSE
ILMN_1672124	FAM198B	FALSE	FALSE
ILMN_2370976	MYOF	FALSE	FALSE

ILMN_2328029	EXT2	FALSE	FALSE
ILMN_1714710	CCDC120	TRUE	FALSE
ILMN_2075800	PTK6	FALSE	FALSE
ILMN_1723843	CSNK2A2	TRUE	FALSE
ILMN_1798030	XPR1	FALSE	FALSE
ILMN_3289745	NA	FALSE	FALSE
ILMN_2317543	ITGB4	FALSE	FALSE
ILMN_3236481	HIGD1A	FALSE	FALSE
ILMN_1708143	FAM127A	FALSE	FALSE
ILMN_2345142	SULF2	FALSE	FALSE
ILMN_1805693	GMIP	FALSE	FALSE
ILMN_1658469	TP53TG1	FALSE	FALSE
ILMN_1705064	NDEL1	FALSE	FALSE
ILMN_1764380	GLTP	FALSE	FALSE
ILMN_2329735	ECM1	FALSE	FALSE
ILMN_1669433	ZSWIM8	TRUE	FALSE
ILMN_2102960	FAM214A	FALSE	TRUE
ILMN_1717262	PROCR	FALSE	FALSE
ILMN_3263099	NA	FALSE	FALSE
ILMN_1688295	ZNF219	TRUE	FALSE
ILMN_2333367	FKBP1A	FALSE	FALSE
ILMN_2399174	TRAK1	FALSE	FALSE
ILMN_1809344	BTBD10	TRUE	FALSE
ILMN_2397028	SERPINB8	FALSE	FALSE
ILMN_1777170	FAM183A	FALSE	FALSE
ILMN_1737653	KRT78	FALSE	FALSE
ILMN_1791912	SIDT2	TRUE	FALSE
ILMN_1706013	FTH1	FALSE	FALSE
ILMN_1791147	YPEL3	FALSE	FALSE
ILMN_3307729	CXXC5	FALSE	FALSE
ILMN_1754842	DLGAP4	TRUE	TRUE
ILMN_1664265	EPHA1	FALSE	FALSE
ILMN_1787567	TSC22D1	FALSE	FALSE
ILMN_1759513	RND3	TRUE	TRUE
ILMN_1687947	HIST1H2BE	FALSE	FALSE
ILMN_1696360	CTSB	FALSE	FALSE
ILMN_1683146	FTH1	FALSE	FALSE
ILMN_1651958	MGP	FALSE	FALSE
ILMN_2095597	CDH16	FALSE	FALSE
ILMN_1786470	C1orf74	FALSE	FALSE
ILMN_1700690	VAT1	FALSE	FALSE
ILMN_3307841	AGR2	FALSE	FALSE
ILMN_1723884	ARF4	FALSE	FALSE
ILMN_1695246	KLHDC8B	FALSE	FALSE
ILMN_1696099	ALDH4A1	FALSE	FALSE
ILMN_1762284	ASPRV1	FALSE	TRUE

ILMN_1757660	CAPS	FALSE	FALSE
ILMN_1759792	CLIP4	FALSE	FALSE
ILMN_2252160	UBC	FALSE	FALSE
ILMN_1718387	LOR	FALSE	TRUE
ILMN_2133205	GPX2	TRUE	FALSE
ILMN_1739840	LRRC8A	FALSE	FALSE
ILMN_1790797	VPS28	FALSE	FALSE
ILMN_1815445	IDS	FALSE	FALSE
ILMN_2125346	MUC16	TRUE	FALSE
ILMN_2098418	GATSL3	FALSE	FALSE
ILMN_2070896	BMPR2	FALSE	FALSE
ILMN_1658094	ZNF365	FALSE	FALSE
ILMN_2313901	PAM	FALSE	FALSE
ILMN_1812441	FAM222B	TRUE	TRUE
ILMN_1793017	DGKQ	TRUE	FALSE
ILMN_1800626	SESN1	FALSE	FALSE
ILMN_1779648	HIST3H2A	FALSE	FALSE
ILMN_1704369	LIMA1	FALSE	FALSE
ILMN_3248591	LTBP2	FALSE	FALSE
ILMN_1730906	FILIP1L	FALSE	TRUE
ILMN_1784523	ATP6V1G1	FALSE	FALSE
ILMN_1690017	SPINK5	TRUE	FALSE
ILMN_3202024	FTL	FALSE	FALSE
ILMN_1696494	CMTM6	FALSE	FALSE
ILMN_2377240	AKTIP	FALSE	FALSE
ILMN_1737308	GLRX	FALSE	FALSE
ILMN_1714567	AHNAK	FALSE	FALSE
ILMN_1801205	GPNMB	FALSE	TRUE
ILMN_1744268	PLEC	TRUE	FALSE
ILMN_1739001	TACSTD2	FALSE	FALSE
ILMN_2134974	RAB38	TRUE	FALSE
ILMN_2198413	MYEOV	FALSE	FALSE
ILMN_1733811	JUP	FALSE	FALSE
ILMN_1691572	TST	FALSE	FALSE
ILMN_1803197	RAB3IP	FALSE	FALSE
ILMN_2061435	MEG3	FALSE	FALSE
ILMN_2234526	NA	FALSE	FALSE
ILMN_1764654	NA	FALSE	FALSE
ILMN_1798303	NA	FALSE	FALSE
ILMN_1751464	TNFSF9	FALSE	TRUE
ILMN_1698231	RRM2B	FALSE	FALSE
ILMN_3307782	FBXL18	FALSE	FALSE
ILMN_2232854	FAP	FALSE	FALSE
ILMN_1678692	MPRIP	FALSE	FALSE
ILMN_1775587	STX19	FALSE	FALSE
ILMN_3233442	RPL13A	FALSE	FALSE

ILMN_2370872	GRINA	FALSE	FALSE
ILMN_1656910	TRIM6	FALSE	FALSE
ILMN_2116127	NPEPPS	FALSE	FALSE
ILMN_1690289	DUOX1	TRUE	FALSE
ILMN_1745471	IRF9	FALSE	TRUE
ILMN_2340721	TMEM134	FALSE	TRUE
ILMN_1695435	NA	FALSE	FALSE
ILMN_2327860	MAL	FALSE	FALSE
ILMN_1798006	ANKRD35	TRUE	FALSE
ILMN_2044453	LPAR5	FALSE	FALSE
ILMN_1713636	S100A6	FALSE	FALSE
ILMN_1705213	TMBIM1	TRUE	FALSE
ILMN_1766054	ABCA1	TRUE	FALSE
ILMN_1659029	DENND6B	FALSE	FALSE
ILMN_1881081	NA	FALSE	FALSE
ILMN_2051684	C4orf3	FALSE	FALSE
ILMN_1712678	RPS27L	FALSE	FALSE
ILMN_1815024	PRDX5	TRUE	TRUE
ILMN_1796461	PRSS8	FALSE	FALSE
ILMN_1699243	MSMB	FALSE	FALSE
ILMN_3305614	ZNF812	TRUE	FALSE
ILMN_1683969	FKBP1A	FALSE	FALSE
ILMN_1698766	PYCARD	FALSE	FALSE
ILMN_1757351	S100A7	FALSE	FALSE
ILMN_2392274	CD82	FALSE	FALSE
ILMN_2061446	NCEH1	FALSE	FALSE
ILMN_1692177	TSC22D1	FALSE	FALSE
ILMN_1867664	SPTLC3	FALSE	FALSE
ILMN_3247261	RAPGEF2	FALSE	FALSE
ILMN_2186983	ANXA8L1	TRUE	FALSE
ILMN_1670844	OPN3	FALSE	FALSE
ILMN_1755727	KDM5B	FALSE	FALSE
ILMN_2377496	ERCC1	FALSE	FALSE
ILMN_1720282	NQO1	FALSE	FALSE
ILMN_2278335	AKR1B15	FALSE	FALSE
ILMN_1826921	NA	FALSE	FALSE
ILMN_1666976	PLD3	FALSE	FALSE
ILMN_1797310	ATP6V1D	FALSE	FALSE
ILMN_1782069	TRAK1	FALSE	FALSE
ILMN_1790227	IGFL2	TRUE	FALSE
ILMN_1747546	TSPAN1	FALSE	FALSE
ILMN_1751708	ITM2B	TRUE	FALSE
ILMN_2243687	LAMP2	FALSE	FALSE
ILMN_1737426	PCMTD1	FALSE	TRUE
ILMN_1727288	EVPL	FALSE	FALSE
ILMN_1814106	CYSRT1	FALSE	FALSE

ILMN_1672004	TOB1	FALSE	TRUE
ILMN_1729234	TPP1	FALSE	FALSE
ILMN_1682929	SYTL2	FALSE	FALSE
ILMN_1745964	IRAK2	FALSE	FALSE
ILMN_1799105	COL17A1	TRUE	FALSE
ILMN_1807169	TINAGL1	FALSE	FALSE
ILMN_2129505	CYB561A3	FALSE	FALSE
ILMN_1761946	PROM2	TRUE	FALSE
ILMN_1690040	TM7SF2	FALSE	FALSE
ILMN_1784661	TMEM2	FALSE	FALSE
ILMN_1803788	LGALS3	FALSE	FALSE
ILMN_1676278	ABCC2	FALSE	FALSE
ILMN_1666546	DUSP14	FALSE	FALSE
ILMN_1679428	CHIC2	FALSE	FALSE
ILMN_1791792	C12orf5	FALSE	FALSE
ILMN_3237991	NA	FALSE	FALSE
ILMN_2124187	TSC22D2	FALSE	FALSE
ILMN_1670272	LRP10	FALSE	FALSE
ILMN_1704294	CDH3	FALSE	FALSE
ILMN_1808404	RHBDF1	FALSE	FALSE
ILMN_1729237	CYB5R1	FALSE	FALSE
ILMN_1701461	TIMP3	FALSE	FALSE
ILMN_1701239	SPRR2E	FALSE	FALSE
ILMN_1750101	S100A11	FALSE	FALSE
ILMN_1803429	CD44	FALSE	FALSE
ILMN_2408576	FAM129B	FALSE	FALSE
ILMN_1679133	SERPINB1	FALSE	FALSE
ILMN_1748352	CTSV	TRUE	FALSE
ILMN_2089167	RHOD	FALSE	FALSE
ILMN_2320330	MAL	FALSE	FALSE
ILMN_1666122	HEG1	FALSE	FALSE
ILMN_2221046	GM2A	FALSE	FALSE
ILMN_1913510	NA	FALSE	TRUE
ILMN_1795342	MLPH	FALSE	FALSE
ILMN_1781374	TUFT1	FALSE	FALSE
ILMN_1782141	GRHL3	FALSE	FALSE
ILMN_1667081	CCND2	FALSE	FALSE
ILMN_1667948	SPATA18	FALSE	FALSE
ILMN_1688103	CTNNBIP1	TRUE	FALSE
ILMN_1659544	STX3	FALSE	FALSE
ILMN_1803018	KIFC2	TRUE	FALSE
ILMN_1809783	FAM25A	FALSE	FALSE
ILMN_1682599	GPRC5A	FALSE	FALSE
ILMN_1811702	GRN	FALSE	FALSE
ILMN_1658926	NOTCH3	FALSE	FALSE
ILMN_1747759	NA	FALSE	FALSE

ILMN_3238676	ULBP2	FALSE	FALSE
ILMN_2334210	ITGB4	FALSE	FALSE
ILMN_2145116	TMEM173	FALSE	FALSE
ILMN_1710622	DUOXA1	TRUE	FALSE
ILMN_2360415	PRNP	FALSE	FALSE
ILMN_1655614	DSP	FALSE	FALSE
ILMN_1685574	TSC22D2	FALSE	FALSE
ILMN_1754576	KRT6C	FALSE	FALSE
ILMN_1673356	FAM83C	FALSE	FALSE
ILMN_2228732	CCNG2	FALSE	FALSE
ILMN_1796490	GRINA	FALSE	FALSE
ILMN_1710325	SCEL	FALSE	FALSE
ILMN_1655468	DSG3	FALSE	FALSE
ILMN_1713638	NIPAL4	FALSE	FALSE
ILMN_2207363	RABAC1	FALSE	FALSE
ILMN_2150465	C5orf28	FALSE	FALSE
ILMN_1721732	GSDMC	FALSE	FALSE
ILMN_1804663	THBS3	FALSE	FALSE
ILMN_1780861	NA	FALSE	FALSE
ILMN_1758272	MYPN	FALSE	FALSE
ILMN_1674609	CLTB	FALSE	FALSE
ILMN_2149971	SPINK6	FALSE	FALSE
ILMN_1667626	EGLN3	FALSE	FALSE
ILMN_1655261	ERP27	FALSE	FALSE
ILMN_2062468	IGFBP7	FALSE	FALSE
ILMN_2169439	ITGAV	FALSE	FALSE
ILMN_2380418	BICD2	FALSE	FALSE
ILMN_1699809	CAPNS2	FALSE	FALSE
ILMN_2134538	FTH1	FALSE	FALSE
ILMN_2073235	FTH1	FALSE	FALSE
ILMN_1716815	CEACAM1	FALSE	FALSE
ILMN_1653161	SNCG	FALSE	FALSE
ILMN_1792265	TRIM4	FALSE	FALSE
ILMN_2313730	RHOC	TRUE	FALSE
ILMN_1684873	ARSD	FALSE	TRUE
ILMN_2073446	C1orf116	FALSE	FALSE
ILMN_1746525	FTH1	FALSE	FALSE
ILMN_1790228	FURIN	FALSE	FALSE
ILMN_1750278	FTH1	FALSE	FALSE
ILMN_1785852	NABP1	FALSE	FALSE
ILMN_2309156	PMEPA1	FALSE	TRUE
ILMN_2184612	C3orf52	FALSE	FALSE
ILMN_2232463	ARL14	FALSE	FALSE
ILMN_2319910	DGKA	TRUE	FALSE
ILMN_1698772	NA	FALSE	FALSE
ILMN_1651832	EHD1	FALSE	FALSE

ILMN_2344373	MVP	FALSE	FALSE
ILMN_1704353	IGSF3	FALSE	FALSE
ILMN_1700268	QPRT	FALSE	FALSE
ILMN_1684306	S100A4	FALSE	FALSE
ILMN_1774901	GDPD3	FALSE	FALSE
ILMN_1663454	PKP1	TRUE	FALSE
ILMN_1725312	RASA1	FALSE	FALSE
ILMN_2376313	MSMB	FALSE	FALSE
ILMN_1723912	IFI44L	FALSE	FALSE
ILMN_1764690	NTS	FALSE	FALSE
ILMN_2381257	DSC2	FALSE	FALSE
ILMN_1724250	GRN	FALSE	FALSE
ILMN_1695924	KLK11	FALSE	FALSE
ILMN_2358919	TP53I3	FALSE	FALSE
ILMN_2384122	GPR56	FALSE	FALSE
ILMN_1790906	MSMB	FALSE	FALSE
ILMN_2167416	MR1	FALSE	TRUE
ILMN_1698100	ANXA2P1	FALSE	FALSE
ILMN_1699695	TNFRSF21	FALSE	FALSE
ILMN_2180885	CWH43	FALSE	FALSE
ILMN_1738558	RGS20	FALSE	FALSE
ILMN_1661500	B4GALT4	FALSE	FALSE
ILMN_2348788	CD44	FALSE	FALSE
ILMN_1795778	P4HA2	FALSE	FALSE
ILMN_3243644	RNA28S5	FALSE	FALSE
ILMN_1711069	YPEL5	TRUE	FALSE
ILMN_1803236	CLCA2	FALSE	FALSE
ILMN_1781010	ARHGEF3	FALSE	FALSE
ILMN_1771261	SYNC	FALSE	FALSE
ILMN_1737163	SH3BGRL3	FALSE	FALSE
ILMN_1676336	NCEH1	FALSE	FALSE
ILMN_1652280	FBXO32	FALSE	FALSE
ILMN_2173835	FTH1P3	FALSE	FALSE
ILMN_2384745	PSG4	FALSE	FALSE
ILMN_1757406	HIST1H1C	FALSE	FALSE
ILMN_1744937	PTPRM	FALSE	FALSE
ILMN_1776936	KANK4	FALSE	TRUE
ILMN_2353633	EMR2	FALSE	FALSE
ILMN_1661743	LINC00263	FALSE	FALSE
ILMN_2411282	QSOX1	FALSE	FALSE
ILMN_1793118	TAX1BP1	FALSE	FALSE
ILMN_1718268	GJB4	FALSE	FALSE
ILMN_1687384	IFI6	FALSE	FALSE
ILMN_3241234	S100A11	FALSE	FALSE
ILMN_1712095	FOXO4	FALSE	FALSE
ILMN_2136495	A2ML1	FALSE	FALSE

ILMN_1655077	PRDM1	FALSE	TRUE
ILMN_1756541	MXD4	TRUE	FALSE
ILMN_1676213	SRPX2	FALSE	FALSE
ILMN_2132599	ANKRD22	FALSE	FALSE
ILMN_1838767	NA	FALSE	FALSE
ILMN_1656368	ALDH4A1	FALSE	FALSE
ILMN_1676563	HTRA1	FALSE	FALSE
ILMN_3183838	FAM25A	FALSE	FALSE
ILMN_1705107	NA	TRUE	FALSE
ILMN_2415748	WSB1	FALSE	FALSE
ILMN_1655930	ELL2	FALSE	FALSE
ILMN_1736190	NA	FALSE	FALSE
ILMN_1664922	FLNB	FALSE	TRUE
ILMN_1745570	KLK7	FALSE	FALSE
ILMN_2090802	TMEM79	FALSE	FALSE
ILMN_1805466	SOX9	FALSE	FALSE
ILMN_1803392	NA	FALSE	FALSE
ILMN_1779252	TRIM22	FALSE	FALSE
ILMN_2381899	OPTN	FALSE	FALSE
ILMN_1666893	TRIML2	TRUE	FALSE
ILMN_1694075	GADD45A	FALSE	TRUE
ILMN_1696911	FTH1	FALSE	FALSE
ILMN_1721134	TGM1	FALSE	FALSE
ILMN_2319913	DGKA	TRUE	FALSE
ILMN_2317923	TMEM132A	FALSE	FALSE
ILMN_1659215	CYP4B1	FALSE	FALSE
ILMN_1705750	TGM2	TRUE	TRUE
ILMN_1718731	KLK5	FALSE	FALSE
ILMN_1736829	MDM2	FALSE	FALSE
ILMN_1691376	JAG1	FALSE	FALSE
ILMN_1799848	ANKRD22	FALSE	FALSE
ILMN_1709870	HES2	FALSE	FALSE
ILMN_1712522	CEACAM6	FALSE	FALSE
ILMN_1731215	MYBPHL	FALSE	FALSE
ILMN_1803277	MVP	FALSE	FALSE
ILMN_1747451	PLCXD1	FALSE	FALSE
ILMN_1688780	S100A4	FALSE	FALSE
ILMN_2060413	CD24	FALSE	FALSE
ILMN_1769388	GJB2	FALSE	FALSE
ILMN_1689211	NA	FALSE	FALSE
ILMN_2363880	ALDH3B2	FALSE	FALSE
ILMN_1712632	XDH	FALSE	FALSE
ILMN_1808713	HSD17B2	FALSE	TRUE
ILMN_1733998	DHRS9	FALSE	FALSE
ILMN_1811479	SPINK6	FALSE	FALSE
ILMN_2052208	GADD45A	FALSE	TRUE

ILMN_1680937	HIST1H2BC	FALSE	FALSE
ILMN_2061565	PLCH2	TRUE	FALSE
ILMN_1796179	HIST1H2BK	FALSE	FALSE
ILMN_3241554	KANK4	FALSE	FALSE
ILMN_1743412	SIAE	FALSE	FALSE
ILMN_3227315	FTH1	FALSE	FALSE
ILMN_3248032	NCCRP1	FALSE	FALSE
ILMN_2329927	ABCG1	FALSE	FALSE
ILMN_1791280	HSPB8	FALSE	FALSE
ILMN_1657111	AHNAK2	TRUE	FALSE
ILMN_1713397	NCCRP1	FALSE	FALSE
ILMN_1672148	AKR1B10	FALSE	FALSE
ILMN_2376723	CDKN2B	TRUE	FALSE
ILMN_2347798	IFI6	FALSE	FALSE
ILMN_1743103	SH3PXD2A	TRUE	FALSE
ILMN_1667295	VASN	TRUE	FALSE
ILMN_1723198	CDKN2B	FALSE	FALSE
ILMN_1702503	ALDH3A1	FALSE	FALSE
ILMN_1742410	BCL2L1	FALSE	FALSE
ILMN_1714592	CDA	FALSE	FALSE
ILMN_2173291	CYP4B1	FALSE	FALSE
ILMN_1813314	HIST1H2BK	FALSE	FALSE
ILMN_1689037	LIPG	FALSE	FALSE
ILMN_2396875	IGFBP3	FALSE	FALSE
ILMN_1807206	DHRS1	FALSE	FALSE
ILMN_2063168	MALL	FALSE	FALSE
ILMN_1655348	GPR1	FALSE	FALSE
ILMN_1709307	GPSM1	TRUE	FALSE
ILMN_1658706	ST6GALNAC2	FALSE	TRUE
ILMN_1717793	C19orf33	FALSE	FALSE
ILMN_1779034	NADSYN1	FALSE	TRUE
ILMN_2197365	RGS2	FALSE	TRUE
ILMN_1734276	PMEPA1	FALSE	TRUE
ILMN_1663042	SDC4	TRUE	TRUE
ILMN_1758938	SLC31A2	FALSE	FALSE
ILMN_1785202	STAT4	FALSE	FALSE
ILMN_1767685	SERPINB7	FALSE	FALSE
ILMN_1806030	PPL	FALSE	FALSE
ILMN_2404688	NUPR1	FALSE	TRUE
ILMN_2083946	TGFA	FALSE	FALSE
ILMN_1712545	S100A3	FALSE	FALSE
ILMN_1805175	TGFA	FALSE	FALSE
ILMN_1793888	SERPINB5	FALSE	FALSE
ILMN_1732609	FAM214B	TRUE	FALSE
ILMN_1775829	PERP	FALSE	FALSE
ILMN_1770940	CDH1	FALSE	FALSE

ILMN_2353054	KLK5	FALSE	FALSE
ILMN_2188862	GDF15	FALSE	FALSE
ILMN_1735700	KLK8	FALSE	FALSE
ILMN_1661197	CLCF1	FALSE	FALSE
ILMN_1730454	FOLR3	FALSE	FALSE
ILMN_1805519	CD24	FALSE	FALSE
ILMN_2094266	HES2	FALSE	FALSE
ILMN_2395139	SERPINB7	FALSE	FALSE
ILMN_1812968	SOX18	FALSE	FALSE
ILMN_1699989	BNIPL	TRUE	FALSE
ILMN_1671703	ACTA2	FALSE	FALSE
ILMN_2390310	MIR22HG	FALSE	FALSE
ILMN_1763666	ALDH3B2	FALSE	FALSE
ILMN_1794782	ABCG1	FALSE	FALSE
ILMN_1688775	NA	FALSE	FALSE
ILMN_1865764	ZMAT3	TRUE	FALSE
ILMN_1797776	PRSS23	FALSE	FALSE
ILMN_1711087	NA	TRUE	FALSE
ILMN_1739605	LYPD3	FALSE	FALSE
ILMN_2211018	SPRR2E	FALSE	FALSE
ILMN_1814305	SAMD9	FALSE	FALSE
ILMN_2404063	APP	TRUE	FALSE
ILMN_1676712	NA	FALSE	FALSE
ILMN_1735712	KRT1	FALSE	FALSE
ILMN_1775501	IL1B	FALSE	FALSE
ILMN_1806607	SFN	FALSE	FALSE
ILMN_3243156	AHNAK2	TRUE	FALSE
ILMN_1791545	KRT23	FALSE	FALSE
ILMN_1746085	IGFBP3	FALSE	FALSE
ILMN_2336609	SYTL2	FALSE	FALSE
ILMN_2148913	TMEM45A	FALSE	FALSE
ILMN_2412336	AKR1C2	FALSE	FALSE
ILMN_2149292	TMEM40	FALSE	FALSE
ILMN_1725852	S100A2	FALSE	FALSE
ILMN_1763000	ADAP2	FALSE	FALSE
ILMN_1753342	SAT1	FALSE	FALSE
ILMN_1651282	COL17A1	TRUE	FALSE
ILMN_1687757	NA	FALSE	FALSE
ILMN_1770085	BTG2	TRUE	FALSE
ILMN_1654262	ZMAT3	TRUE	FALSE
ILMN_2078592	ADTRP	FALSE	FALSE
ILMN_1697733	CST6	FALSE	FALSE
ILMN_1738546	NA	FALSE	FALSE
ILMN_2404065	APP	TRUE	FALSE
ILMN_2191967	SPRR2D	FALSE	FALSE
ILMN_1772627	NSG1	TRUE	FALSE

ILMN_1651574	AQP3	FALSE	FALSE
ILMN_1744212	INPP5D	FALSE	FALSE
ILMN_1673191	S100A7A	FALSE	FALSE
ILMN_1665035	KRT14	FALSE	FALSE
ILMN_1680110	ADIRF	TRUE	FALSE
ILMN_1805665	FLRT3	TRUE	FALSE
ILMN_1779147	ENC1	FALSE	FALSE
ILMN_2406035	LAMA3	FALSE	FALSE
ILMN_1700583	ZNF750	TRUE	FALSE
ILMN_1713124	AKR1C3	FALSE	FALSE
ILMN_2139970	ALDH1A3	FALSE	FALSE
ILMN_1796423	CLIC3	FALSE	FALSE
ILMN_1701424	LAMC2	FALSE	FALSE
ILMN_1712082	GCNT3	FALSE	FALSE
ILMN_2214197	TP53INP1	TRUE	FALSE
ILMN_1654118	BCL2L1	FALSE	FALSE
ILMN_1770922	TMEM45A	FALSE	FALSE
ILMN_1797704	NA	FALSE	FALSE
ILMN_1751120	HIST1H4H	FALSE	FALSE
ILMN_1727589	SULT2B1	FALSE	FALSE
ILMN_1666845	KRT17	FALSE	FALSE
ILMN_1771538	PSCA	FALSE	FALSE
ILMN_1769759	SERPINB13	FALSE	FALSE
ILMN_1714108	TP53INP1	TRUE	FALSE
ILMN_1658483	IL1A	FALSE	FALSE
ILMN_1729216	CRYAB	FALSE	FALSE
ILMN_1774874	IL1RN	FALSE	FALSE
ILMN_1724686	CLDN1	FALSE	FALSE
ILMN_1758623	HIST1H2BD	FALSE	FALSE
ILMN_1807439	ALDH1A3	FALSE	FALSE
ILMN_2121408	HBEGF	FALSE	TRUE
ILMN_1686573	DEFB1	FALSE	FALSE
ILMN_1780255	KLK6	FALSE	FALSE
ILMN_1692223	LCN2	FALSE	FALSE
ILMN_3242166	KRT16	FALSE	FALSE
ILMN_2067656	CCND2	FALSE	TRUE
ILMN_1784602	CDKN1A	FALSE	FALSE
ILMN_1653934	KRT17	FALSE	FALSE
ILMN_1814270	LY6D	FALSE	FALSE
ILMN_1792689	HIST1H2AC	FALSE	FALSE
ILMN_1784294	CPA4	FALSE	FALSE
ILMN_1706483	C1orf116	FALSE	FALSE
ILMN_1661708	NA	FALSE	FALSE
ILMN_3243690	NA	FALSE	FALSE
ILMN_1763011	SUGCT	FALSE	FALSE
ILMN_1732071	HIST2H2BE	FALSE	FALSE

ILMN_3195198	KRT17	FALSE	FALSE
ILMN_2064860	NA	FALSE	FALSE
ILMN_1768973	HIST2H2AC	FALSE	FALSE
ILMN_1651496	HIST1H2BD	TRUE	FALSE
ILMN_1775330	C15orf52	FALSE	FALSE
ILMN_2058782	IFI27	FALSE	FALSE
ILMN_2228162	KRT16	FALSE	FALSE
ILMN_3242900	NA	FALSE	FALSE
ILMN_1659047	NA	FALSE	FALSE
ILMN_1715684	LAMB3	TRUE	FALSE
ILMN_1658569	NA	FALSE	FALSE
ILMN_1740938	APOE	FALSE	FALSE
ILMN_1750748	KRT16P3	FALSE	FALSE
ILMN_2144426	NA	FALSE	FALSE
ILMN_1713813	KRT16P3	FALSE	FALSE
ILMN_1711174	SPRR1B	FALSE	FALSE
ILMN_1693192	PI3	FALSE	TRUE
ILMN_1736760	KRT16	FALSE	FALSE
ILMN_1721354	KRT6B	FALSE	FALSE

Supplementary Table S6. Primers and conditions used for qRT-PCR.

Gene symbol	Forward Primer (5' to 3')	Reverse Primer (5' to 3')	Reference/Supplier
ATL3	ACAGCCAGTCAACTGTGAAAG	CCAGACGACCGTATTCTGTGA	PrimerBank ID 45827805c1
PA2G4	ACAGGGTACTTCGGTCCTTG	ATGGCATCACCTTTCTCACAC	PrimerBank ID 124494253c2
GAPDH	TGCACCACCAACTGCTTAGC	GGCATGGACTGTGGTCATGAG	Vandesompele et al. [47]
YWHAZ	ACTTTTGGTACATTGTGGCTTCAA	CCGCCAGGACAAACCAGTAT	Vandesompele et al. [47]
RPL13	CCTGGAGGAGAAGAGGAAAGAGA	TTGAGGACCTCTGTGTATTTGTCAA	Vandesompele et al. [47]

Conditions used: 95°C for 2min; 40/45 cycles of 95°C for 15sec, 58°C for 20sec, 72°C for 15sec, 76°C for 5sec and read; final extension 78°C for 8min; followed by melting curve analysis from 65°C to 90°C to confirm product-specific amplification.

Supplementary Table S7. Antibodies used for western blotting.

Antibody	Manufacturer	Band size (kD)	Dilution used
Rabbit anti-ATL3	ABGENT	59	1:2000
Rabbit anti-PA2G4	GeneTex	48	1:20000
Mouse anti-β-actin	Thermo Scientific	42	1:150000
Polyclonal goat anti-rabbit	Dako		1:1000
Polyclonal goat anti-mouse	Dako		1:1000
